# Stereoselective [2 + 2 + 2] Cycloaddition of Benzocyclobutanones and Norbornadienes through Nickel-Catalyzed C–C Bond Activation

**DOI:** 10.1021/acs.orglett.5c01324

**Published:** 2025-05-16

**Authors:** Robert C. Richter, Ivo H. Lindenmaier, David Schray, Markus Ströbele, Ivana Fleischer

**Affiliations:** † Institute of Organic Chemistry, Faculty of Science, 9188 Eberhard Karls Universität Tübingen, Auf der Morgenstelle 18, 72076 Tübingen, Germany; ‡ Institute of Inorganic Chemistry, Faculty of Science, Eberhard Karls Universität Tübingen, Auf der Morgenstelle 18, 72076 Tübingen, Germany

## Abstract

We report a nickel-catalyzed stereoselective [2 + 2 + 2] cycloaddition between norbornadienes and benzocyclobutanones via C–C activation. This transformation generates four stereocenters, producing nortricyclane scaffolds that introduce three-dimensional architecture. The reaction proceeds with high stereoselectivity while allowing precise steric control of the reaction site, offering a versatile approach for constructing intricate stereochemical frameworks.

## Graphical abstract

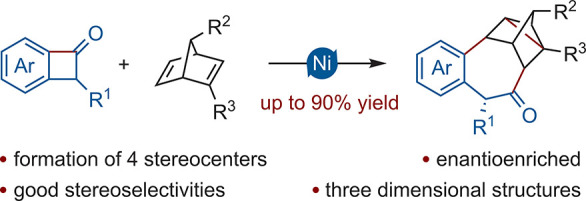

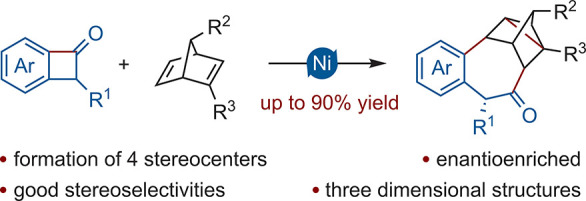

The development of metal-catalyzed C–C bond cleavage reactions has gained exceeding attention in modern synthetic chemistry, enabling atom-efficient and step-economical routes to complex, three-dimensional scaffolds commonly present in natural products.^1^ Ring expansion reactions involving three- and four-membered rings have been particularly instrumental in overcoming the inherently inert nature of the C–C bond, as demonstrated by Huffman and Liebeskind,^2^ Nishimura et al.,^3^ Murakami et al.,^4^ and others.^5^

Benzocyclobutanones (BCBs), represent well-studied compounds in C–C activation reactions, contributing to numerous transformations, including natural product synthesis.^6^ Traditionally, the strain-driven reactivity of BCBs has been harnessed through noble metal catalysis activating the C^1^–C^8^ or C^1^–C^2^ bond.^7^ However, these generally intramolecular transformations often require elevated temperatures, carbonyl preactivation, and precious metals, which limit their economic feasibility.^8^ When these challenges are addressed, nickel catalysis provides a versatile and economically attractive expansion, especially for intermolecular reactions.^7,9^ Seminal work by Martin and colleagues demonstrated nickel-catalyzed C^1^–C^2^ activation of BCBs, enabling the insertion of 1,3-conjugated dienes and alkynes with excellent diastereoselectivity at ambient temperatures (Scheme 1a).^10^ Based on these findings, Shi and co-workers showed that alkynes could also be inserted into the C^1^–C^8^ bond utilizing steric control.^11^

**Scheme 1:**
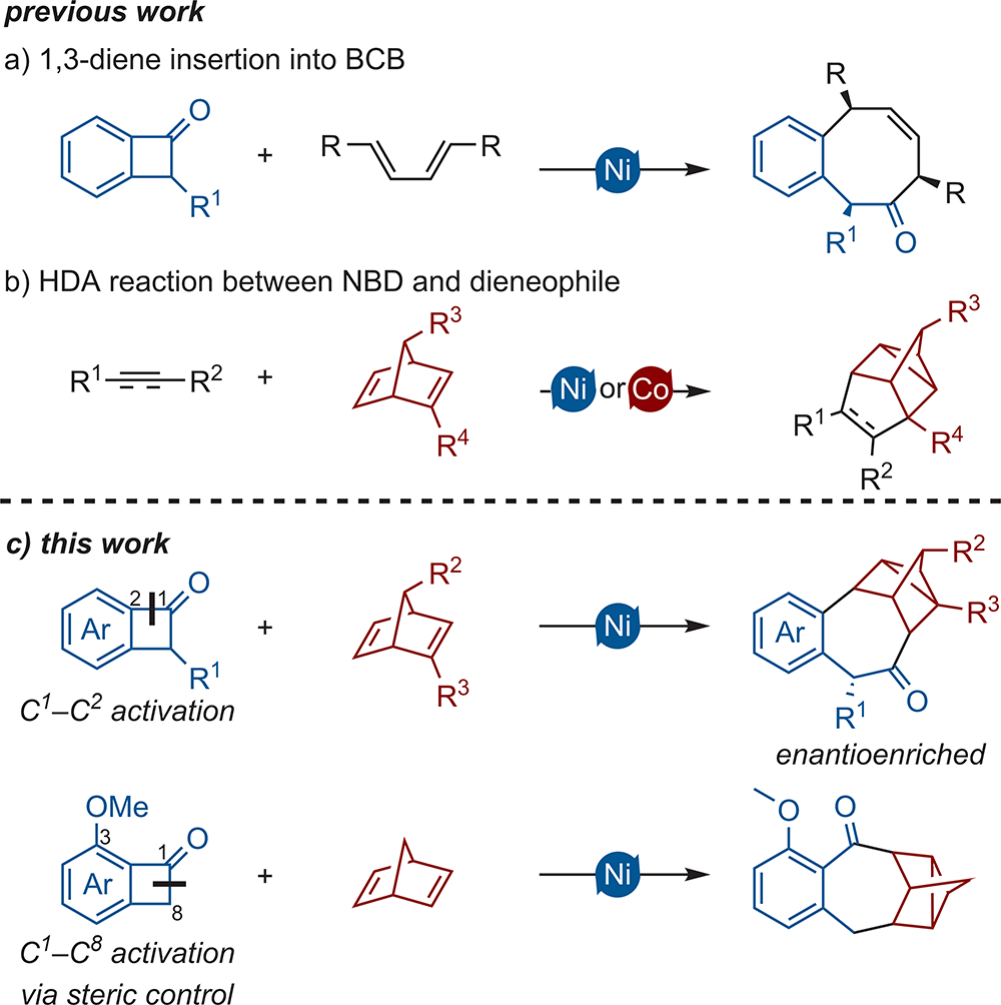
Nickel-Catalyzed Activation of BCB Combined with the HDA of NBD

Inspired by these advances, we sought to explore further substrate classes and report here the intermolecular [2 + 2 + 2] insertion of norbornadiene (NBD) into BCBs. NBD is known to engage in homo-Diels–Alder (HDA) reactions with dieneophiles,^12^ forming nortricyclane frameworks either thermally or catalytically (e.g., Ni and Co; Scheme 1b).^13^ This approach enables the construction of four stereocenters in a single step and can be achieved enantioselectively, offering a powerful pathway to complex carbon frameworks.^14^ Additionally, by introduction of a substituent in the 3-position of BCB, the regioselectivity can be varied, broadening the scope of accessible scaffolds (Scheme 1c).

To begin our investigation, we optimized the nickel-catalyzed reaction between BCB (**1a**) and NBD (**2a**) to yield product **3aa** in 91%, employing (*R*)-MonoPhos as the ligand in 1,3-dimethyl-2-imidazolidinone (DMI) at 80 °C (Table 1, entry 1). Reducing the reaction temperature to 50 °C led to comparable yields (Table 1, entry 2). However, 80 °C was selected as the standard reaction condition due to the shorter reaction time (80 °C, 3 h; 50 °C, 10 h; see the Supporting Information for further details) and increased robustness. Attempts to reduce the catalyst loading to 5 mol % resulted in inconsistent yields. Similarly, varying the ligand-to-catalyst ratio proved unfavorable (Table 1, entry 3). The solvent appeared to play a crucial role in stabilizing the catalytic species, as increased reaction concentrations led to slightly lower yields (Table 1, entry 4), and for some solvents, the formation of a black precipitate was observed, indicating catalyst decomposition. Dimethylimidazolidinone (DMI) proved to be the most robust solvent, although various others, including apolar and etherical solvents, afforded fair to good yields (Table 1, entries 5–8) (see the Supporting Information for details).

**Table 1:**
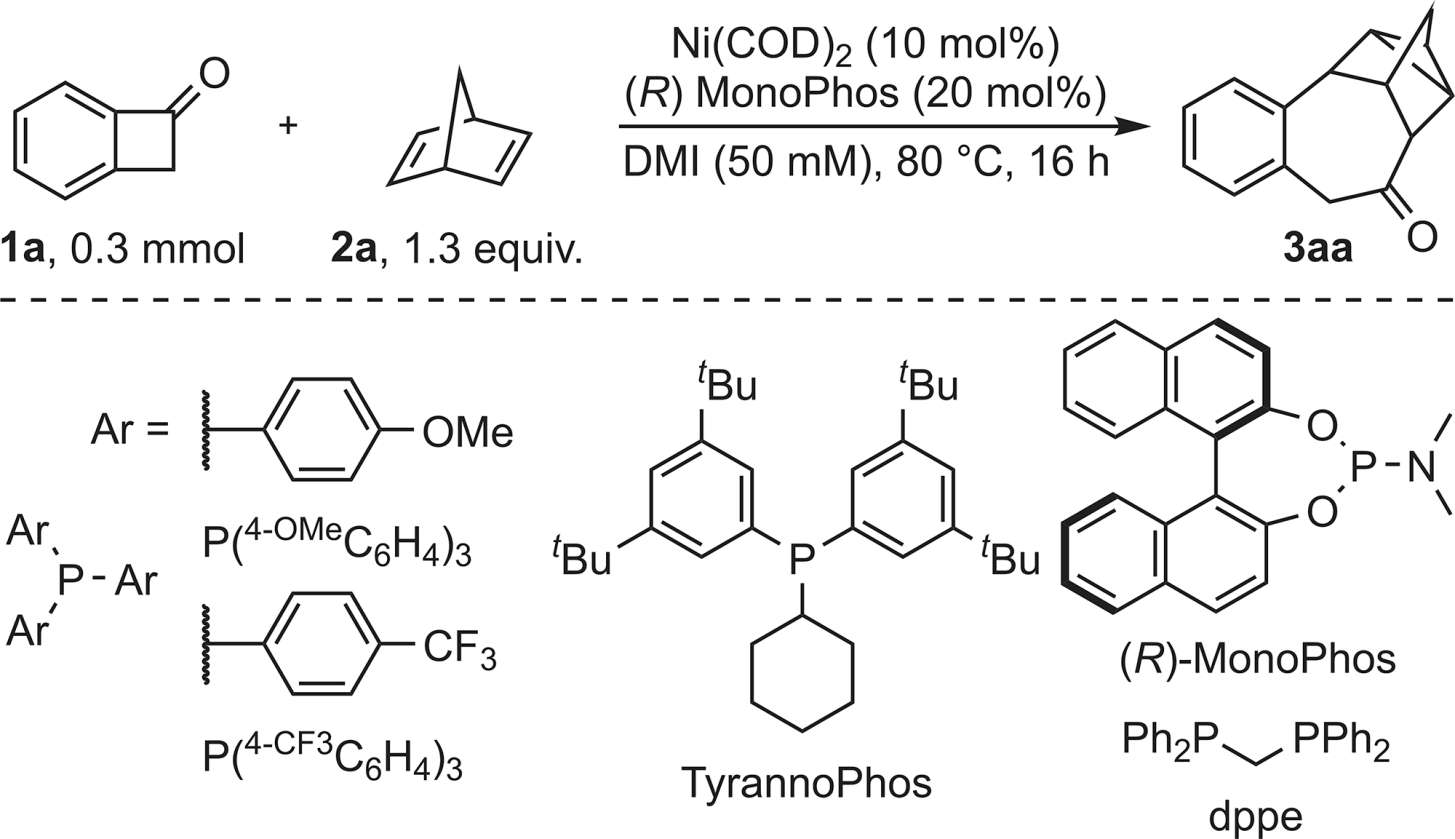
Optimization of Reaction Conditions

entry	variation from standard conditions^a^	yield (%)^b^
1	none	91
2	50 °C	96
3	12 mol % (*R*)-MonoPhos	63
4	150 mM	78
5	toluene, 150 mM	55
6	*n*-hexane, 150 mM	73
7	1,4-dioxane, 150 mM	63
8^c^	THF	24
9^c^	P(^4‑OMe^C_6_H_4_)_3_, 40 °C	55
10^c^	dppe	29
11^c^	TyrannoPhos	27
12	toluene, P(^4‑CF_3_^C_6_H_4_)_3_, 50 °C	11
13	toluene, without ligand or nickel, 50 °C	0

a Reaction conditions: **1a** (0.3 mmol), **2a** (1.3 equiv), Ni­(COD)_2_ (10 mol %), and (*R*)-MonoPhos (20 mol %) in DMI (6 mL) at 80 °C for 16 h.

b GC–FID yields utilizing *n*-pentadecane as an internal standard.

c **1a** (0.1 mmol).

Likewise, the developed system accepted various ligands, including electron-rich, bidentate, and sterically demanding phosphines, albeit with reduced yields (Table 1, entries 9 – 11). Electron-poor phosphines performed poorly, especially compared to the literature-known nickel-catalyzed insertion of 1,3-dienes into BCBs (Table 1, entry 12).^10^ Other screened phosphoramidite-type ligands gave inferior yields (see the Supporting Information for further information). Changing the nickel(0) source from Ni­(COD)_2_ to Ni­(^4‑^*t*^Bu^stb)_3_ reduced the yield, and a control reaction in toluene without either ligand or nickel confirmed their essential role (Table 1, entry 13).

With the optimized reaction conditions in hand, the scope of the reaction was investigated (Table 2). Product **3aa** was isolated in 90% yield with 34% enantiomeric excess (ee) at 50 °C. With exploitation of the modularity provided by phosphoramidite-type ligands and employment of Monophos **2**,^15^ product **3aa** was obtained with up to 71% ee at the cost of the yield. Modifying the aromatic ring of **1a**, by extending the π system or adding simple substituents, was tolerated. Electron-donating methoxy and dioxole substituents were also tolerated, requiring elevated temperatures for **1d** (150 °C). Despite the high temperature, 20% ee was still observed. Product **3ea** showcased a minor amount of the regioisomeric C^1^–C^8^ activation due to steric strain imposed by the pseudo-*ortho* substituent. Electron-withdrawing pseudo-*o*-fluorine-substituted **1f** resulted in 39% of **3fa** (29% ee) requiring 100 °C. Similar to **3ea**, minor quantities of the regioisomer were observed on the account of the pseudo-*ortho* substituent. We were pleased to see that, in the benzylic position, substituted BCBs displayed good to excellent diastereoselectivity and furnished moderate to good yields (39–67%). Hereby, a higher catalyst loading of 20 mol % was necessary for **3ha** and **3ia**. Additionally, **1l** derived from ibuprofen, combining a benzylic and aromatic substituent, and spirocyclic **1k** were tolerated.

**Table 2:**
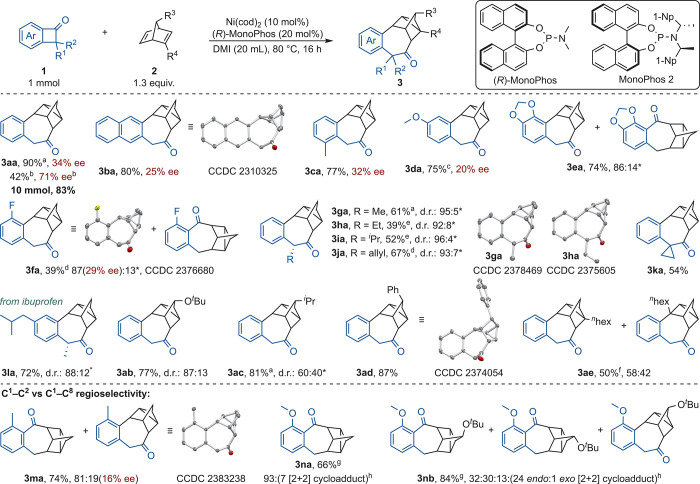
Substrate Scope of BCBs and NBDs^a^

a At 50 °C for 24 h.

b MonoPhos **2** (12 mol %) and *n*-hexane (20 mL) at 70 °C for 16 h.

c At 150 °C.

d At 100 °C.

e **2** (3.0 equiv), Ni­(COD)_2_ (20 mol %), and (*R*)-MonoPhos (40 mol %).

f PhCN (2 equiv) as an additive.

g **1n** (1 mmol), **2** (1.3 equiv), Ni­(COD)_2_ (10 mol %), and PCy_3_ (20 mol %) in toluene (20 mL) at 100 °C for 16 h.

h The isomer ratio was determined by GC–FID assuming identical response factors or ^1^H NMR. The ee values were determined by GC–MS on the chiral stationary phase. Hydrogen atoms of ORTEP structures are omitted for clarity, and the thermal ellipsoids are drawn at 50% probability level.

i Standard reaction conditions: **1** (1 mmol), **2** (1.3 equiv), Ni­(COD)_2_ (10 mol %), and (*R*)-MonoPhos (20 mol %) in DMI (20 mL) at 80 °C for 16 h.

The scope of dienes was investigated as well. NBDs substituted in the 7 position extended the scope with good yields (77–87%). Hereby, the diastereoselectivity strongly depends on the steric bulk (**3a**(**b**,**c**)) and was inverted by a phenyl substituent (**3ad**, *vide infra*). Although the observed selectivities were evident through thorough NMR characterization, we unambiguously could ascribe selected structures (**3**(**b**,**f**,**h**,**i**,**m**)**a** and **3ad**) by X-ray crystallography. Finally, the reaction of 2-hexyl NBD with **1a** furnished two constitutional isomers (*vide infra*) in a fair yield (50%). Hereby, the addition of PhCN was necessary, most likely due to competing coordination of cyclooctadiene (COD) with sterically hindered norbornadiene **2e**.^16^

The influence of structural elements on the regioselectivity was explored in more detail. A methyl group in the 3 position of benzocylobutanone enabled addressing the C^1^–C^8^ bond by sterically encumbering the activation of the C^1^–C^2^ bond.^11^ The product was obtained with good selectivity (81:19). Generally, increasing steric demand in the 3 position resulted in more C^1^–C^8^ activation product (**3ma** > **3ea** > **3fa**). A OMe substituent in the 3 position almost completely inhibited the reaction. After further optimization, Ni­(COD)_2_ with PCy_3_ in toluene was found to be suitable for this transformation, yielding 66% of the [2 + 2 + 2] cycloaddition product (**3na**) with good chemoselectivity ([2 + 2] cycloadduct as the side product). Unfortunately, other 1,4-dienes deviating from the NBD scaffold as well as methyl bicyclo[2.2.1]­hepta-2,5-diene-2-carboxylate were not tolerated. Simple cyclobutanone also did not furnish any product (see the Supporting Information for unsuccessful substrates).^17^

To demonstrate the synthetic utility of the established catalytic system, **3aa** was synthesized on a 10 mmol scale in 83% yield (Table 2). Derivatization reactions could be carried out, showcasing reduction with NaBH_4_ (89%), Johnson–Corey–Chaykovsky epoxidation (81%), 1,2-addition of a Grignard reagent (65%), and Baeyer–Villinger oxidation (54%). To our delight, all reactions displayed excellent diastereo- and chemoselectivities furnishing the corresponding products (Scheme 2).

**Scheme 2:**
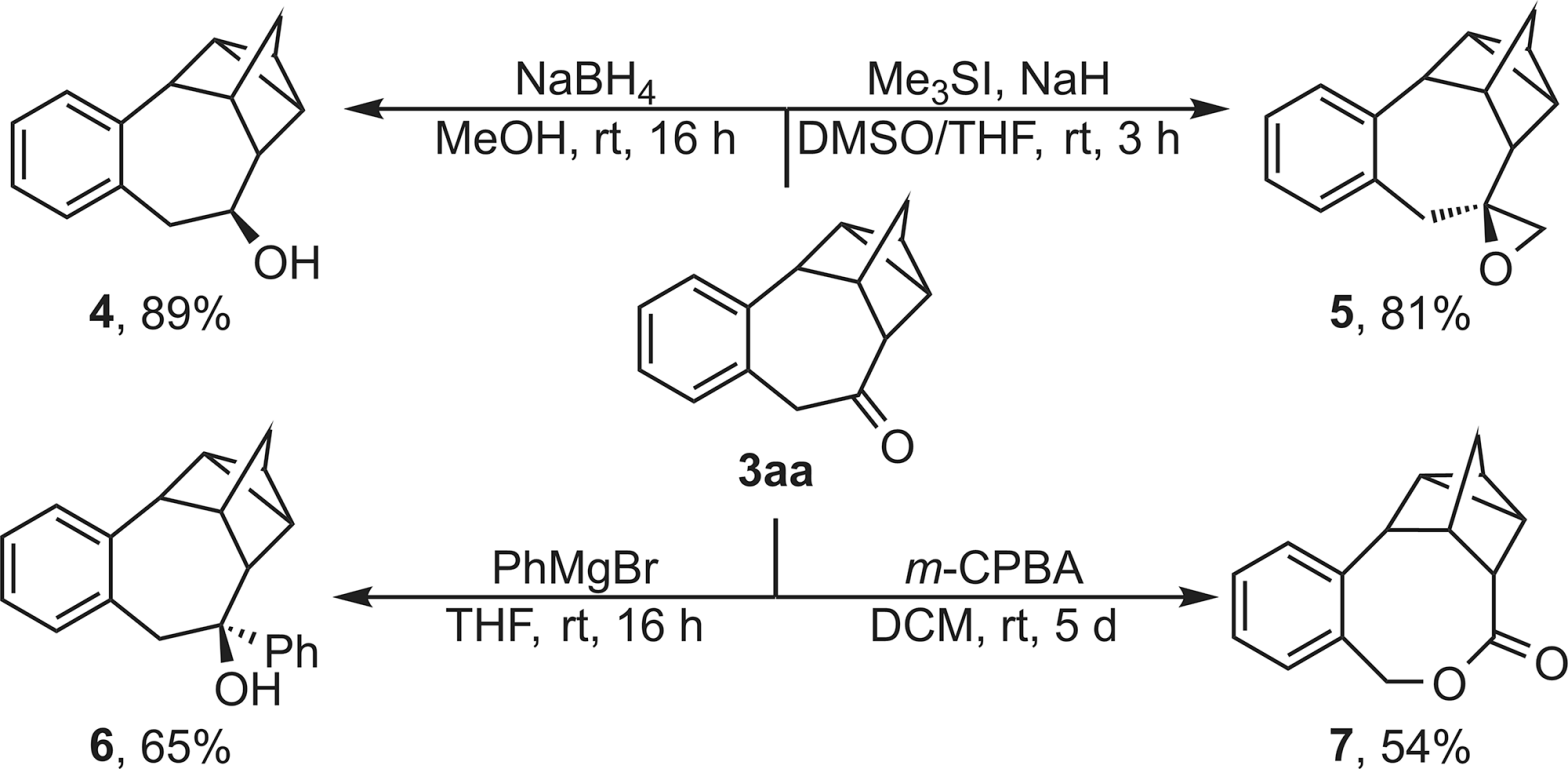
Synthetic Utility of the Cycloaddition Product

The analysis of the observed stereoisomers formed in the [2 + 2 + 2] cycloaddition provided tentative mechanistic insights. Based on previous experimental and computational findings, C–C bond activation of BCB likely proceeds via a Ni(0)­L_2_ complex, resulting in an oxidative addition complex with two ligands.^11,18^ In this scenario, one ligand must dissociate to enable NBD coordination. When a benzylic substituent is present, the ligand on the same side is sterically favored to dissociate, allowing *syn*-NBD insertion relative to the benzylic substituent (Figure 1, I), in agreement with the *syn*-diastereoselectivity observed for conjugated 1,3-diene insertions in BCBs.^10^

**Figure 1:**
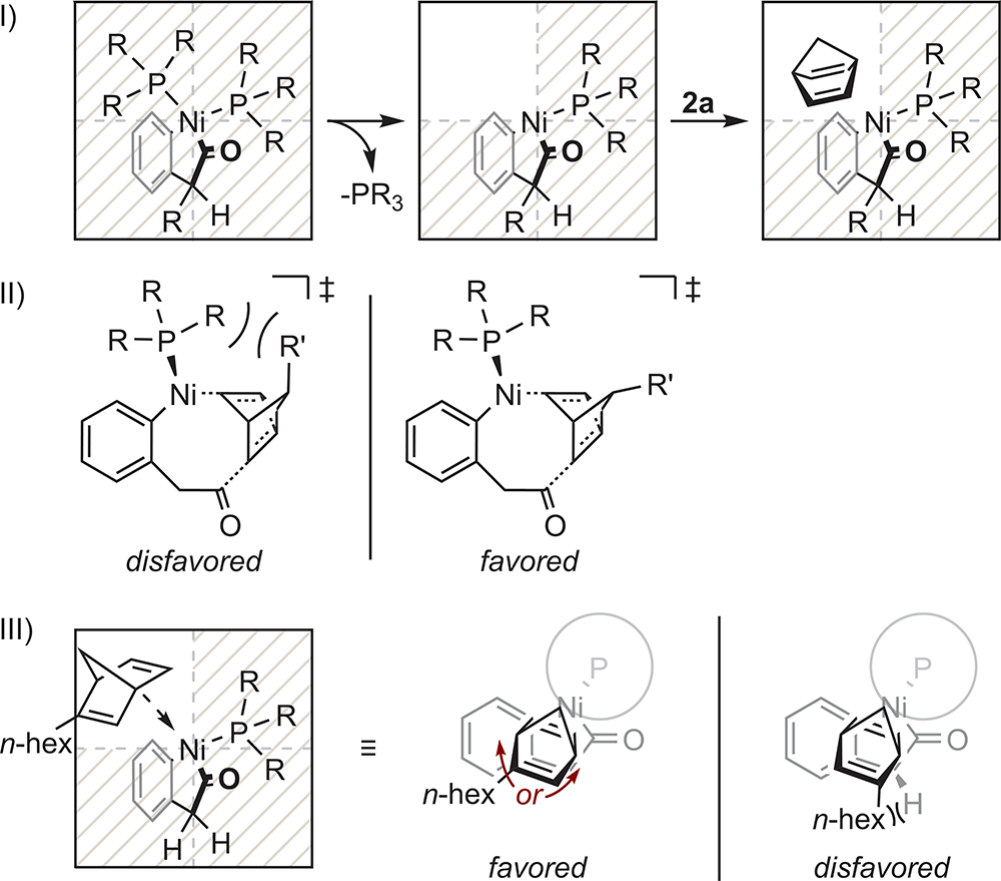
Proposed models, explaining the diastereoselectivity of benzylic-substituted BCBs (I) and NBDs carrying a non-coordinative substituent in the 7 position (II) as well as the constitutional isomer ratio of product **3ae** (III).

In the presence of **2b** or **2c**, the substituent in the 7 position is oriented distal to the nickel center, minimizing strain during insertion (Figure 1, II). For NBD **2d**, the inverted selectivity is explained by a favorable π interaction between the phenyl substituent and the nickel center. These results suggest that the insertion of NBD occurs in the C^1^–Ni bond. The two constitutional isomers observed for **3ae** allowed us to deduce the NBD approach path toward the oxidative addition complex. Upon dissociation of one ligand, **2e** approaches opposite the remaining ligand with the unsubstituted double bond facing nickel. The *n*-hexyl group positions distally from the benzylic CH_2_ group of ketone **1a**. As **2e** is now orthogonal with respect to product orientation, a clockwise or anticlockwise rotation occurs, explaining the observed pseudo-C_2_ axis differentiating the product (**3ae**) constitutional isomers (Figure 1, III).

To gain further insights into the mechanism, we conducted ^31^P NMR studies, indicating that Ni­(**2a**)­((*R*)-MonoPhos)_2_ is likely the resting state. Furthermore, a possible poisoned catalyst species was identified as a Ni(0) carbonyl complex (see the Supporting Information for details), which was observed previously during nickel-catalyzed BCB activation.^11^ Based on these findings and prior studies, a preliminary mechanism was proposed (Figure 2). The reaction initiates with the coordination of **1a** to Ni(0)­L_2_, initiating oxidative addition into the C^1^–C^2^ bond. One ligand is then replaced by **2a**, enabling insertion into the C^1^–Ni bond. After ligand saturation, **3aa** is reductively eliminated, closing the cycle by releasing Ni(0)­L_2_.

**Figure 2:**
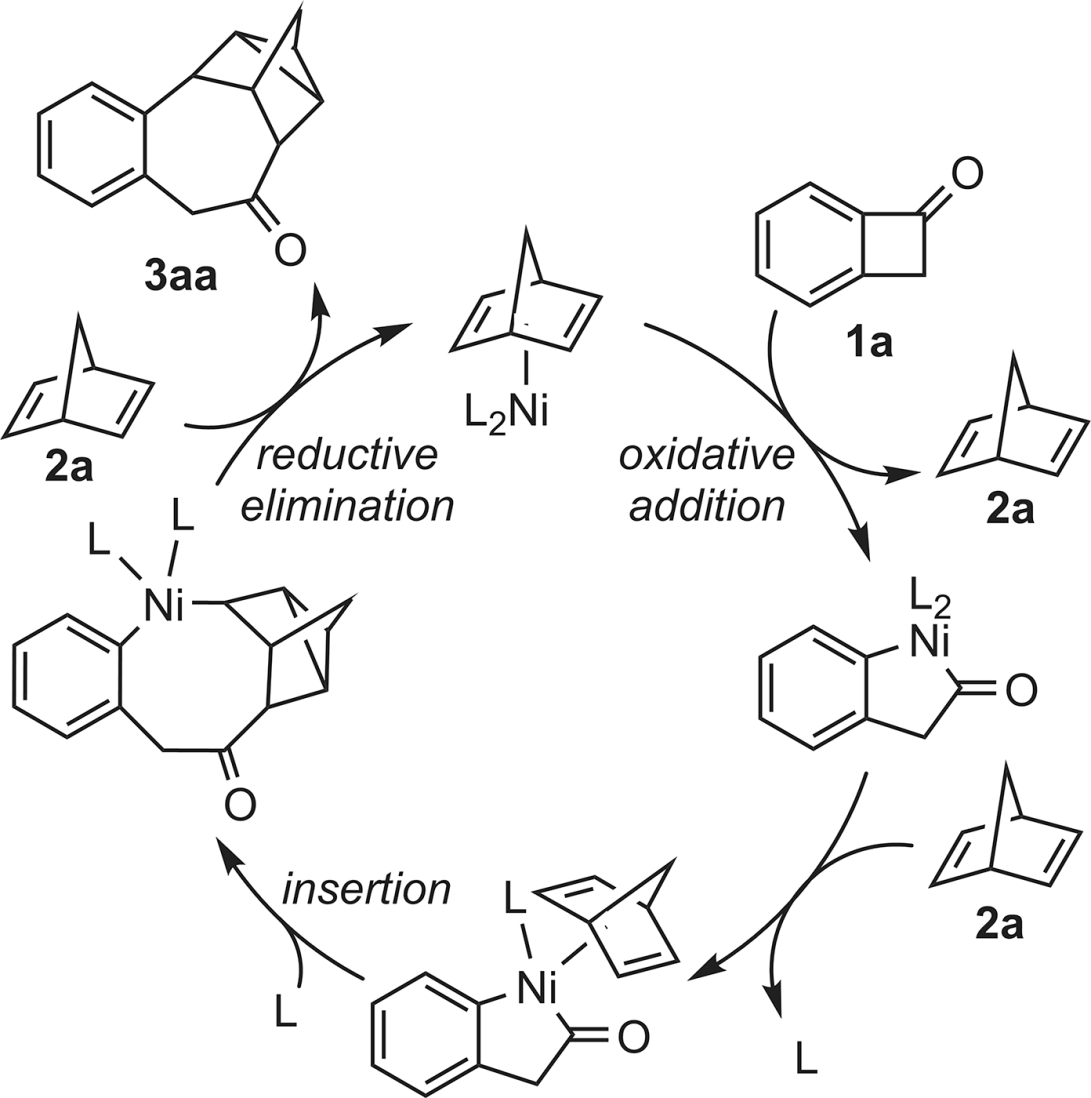
Proposed mechanism for the insertion of **2a** in **1a** subsequent to C^1^–C^2^ activation.

In conclusion, we report the nickel-catalyzed stereoselective intermolecular insertion of norbornadienes into benzocyclobutanones via C–C bond activation. This transformation efficiently constructs complex nortricyclane scaffolds with good stereoselectivity and tunable regioselectivity, broadening the synthetic utility of BCBs. The substrate screening highlights the tolerance of various substitution patterns, providing valuable mechanistic insights. Furthermore, the successful upscale experiment and subsequent derivatization demonstrate the practical applicability of this methodology in complex molecule synthesis.

## Supplementary Material



## Data Availability

The data underlying this study are available in the published article and its Supporting Information.

## Notes

Experimental procedures and compound characterization: ^1^H, ^13^C, ^19^F, and ^31^P NMR spectra, structural assignments made with COSY, HSQC, HMBC, and NOESY experiments, HRMS for all new compounds, IR, and X-ray crystallographic data (PDF)
