# Supplementary material for: Stereoselective [2 + 2 + 2] Cycloaddition of Benzocyclobutanones and Norbornadienes through Nickel-Catalyzed C–C Bond Activation
Source: Org Lett. 2025 May 16;27(21):5385–9. doi: 10.1021/acs.orglett.5c01324 (PMC12131216; doi:10.1021/acs.orglett.5c01324)
Supplement: Supplementary file 1 [file ol5c01324_si_001.pdf]

SUPPORTING INFORMATION

Stereoselective [2+2+2] Cycloaddition of Benzocyclobutanones and Norbornadienes through Nickel Catalyzed C–C Bond Activation

Robert C. Richter,[a] Ivo H. Lindenmaier,[a] David Schray,[a] Markus Ströbele,[b] Ivana Fleischer*[a]

[a] Institute of Organic Chemistry, Faculty of Science, Eberhard Karls Universität Tübingen, Auf der Morgenstelle 18, 72076 Tübingen, Germany. E-mail: ivana.fleischer@uni-tuebingen.de

[b] Institute of Inorganic Chemistry, Faculty of Science, Eberhard Karls Universität Tübingen, Auf der Morgenstelle 18, 72076 Tübingen, Germany.

Contents

1 General information **S2**

1.1 Chemicals and general techniques S2

1.2 Analytical techniques S2

2 General procedures **S4**

3 Optimization **S10**

3.1 Optimization of the standard reaction S10

3.2 Optimization of 3-OMe BCB S18

4 Synthesis **S20**

4.1 Weinreb amides S20

4.2 Kobayashi and ketene precursors S36

4.3 Benzocyclobutanones S44

4.4 Norbornadienes S73

4.5 Catalytic Products S81

4.6 Derivatisation products S190

5 ^{31}P NMR studies **S206**

6 Kinetic measurements **S206**

7 Crystal structures **S208**

8 GC on chiral stationary phase **S217**

9 GC-FID calibration **S221**

10 Literature **S225**

11 IR-Spectra **S227**

1 General information

1.1 Chemicals and general techniques

All reactions requiring an inert atmosphere were carried out under argon employing standard Schlenk technique or a glovebox (GS MEGA E-Line, Glovebox Systemtechnik, <5 ppm O_2, <0.1 ppm H_2O) with pre-dried glassware. Solvents (except DMI) were dried via a commercially available solvent purification system (MB SPS-5). DMI was dried *via* an activated neutral Al_2O_3 column. Solvents for the catalytic reaction were degassed by passing through argon and were stored over 3 Å MS from abcr (activated *in vacuo* at 280 °C for 30 min). Solvents employed during chromatography were HPLC grade or distilled prior to use. Column chromatography was performed with silica (35 – 70 µm, 60A) from Thermo Fischer. Flash chromatography was carried out using the Interchim Puriflash XS420 system with FlashPure EcoFlex Silica 25 g columns (50 µm, irregular). Preparative HPLC was conducted using an Agilent 1260 Infinity II preparative binary pump, a 1260 Infinity II preparative autosampler, a 1290 Infinity II column holder, a 1260 Infinity II DAD and a 1260 Infinity II preparative fraction collector. A Kinetex C18 column from *Phenomenex*, with 5 µm particle size, 100 Å pore size, 250 mm length and a 21.2 mm diameter was used. HPLC grade solvents with 0.1% (volume) formic acid as an additive were used. For separations, the gradients are given as a binary eluent combination X:Y (Y is the more polar solvent) with X + Y = 100. Chemicals including ligands L1 – L17 were purchased from abcr, BLDpharm, Sigma-Aldrich, Thermo Fischer, Acros or TCI and were used without further purification. Ligand L18 was kindly provided by Dielman and co-workers.[1] Low reaction temperatures were achieved employing the Julabo FT902 Cryostat or a liquid nitrogen acetone cooling bath.

1.2 Analytical techniques

NMR spectra were recorded using the Bruker Avance III HDX 700, Bruker Avance III HDX 600 or Bruker Avance 400 spectrometers. ^{13}C NMR and ^{19}F NMR experiments were proton-decoupled (not denoted explicitly). Chemical shifts are reported in parts per million relative to tetramethyl silane using the residual NMR solvent signals[2] (chloroform-d: ^{1}H δ = 7.26 and ^{13}C δ = 77.16; acetone-d_6: ^{1}H δ = 2.06) and the J coupling constants are given in Hertz with the usual designations for splitting patterns (s = singlet, d = doublet, dm = doublet of multiplet, t = triplet, q = quartet, sep = septet, m = multiplet). Structural assignments were made with additional information from gCOSY, gHSQC, and gHMBC experiments.

Information on the crystal structures is found in section 5.

HR-MS (ESI/APCI-TOF) measurements were carried out by the mass spectrometry department of the Institute of Organic Chemistry, University of Tübingen. Measurements were carried out using maXis 4G from Bruker (ESI/APCI-TOF). The molecular ion $[M+H/Na]^+$ is given in m/z units.

GC-LR-MS (EI) analysis was carried out with an Agilent 7820A/5977B GC-system/MSD employing an Agilent 190915-433UI column (30 m × 250 µm × 0.25 µm). Program: 50 °C – 280 °C over 15 minutes.

GC-FID analysis was performed on an Agilent 7820A system with an Agilent 19091J-431 column (30 m × 320 µm × 0.25 µm), utilizing H_2 as the carrier gas. The program used heated from 50 °C to 280 °C within 15 min.

FT-IR spectra were measured by a Cary 630 FTIR by applying the neat sample on a diamond ATR sampler.

Melting points were determined either by visual detection employing a Büchi B-540 (heating rate 5 °C/min).

Thin layer chromatography (TLC) was performed on aluminum plates coated with 0.20 mm silica gel 60 with fluorescence indicator UV_{254} from Macherey-Nagel and compounds were detected under UV light (254 nm) or using an anisaldehyde stain (4 mL anisaldehyde, 3 mL glacial acetic acid, 10 mL H_2SO_4 (96%), 200 mL EtOH).

2 General procedures

General Procedure A (GP-A): Nickel catalyzed insertion of benzocyclobutanones (BCB´s) into norbornadienes (NBD´s) for product isolation:

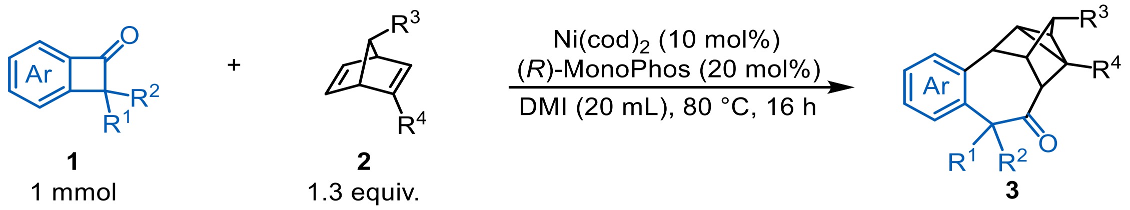

In a glove box, Ni(COD)$_2$ (0.1 mmol, 10 mol%) and (*R*)-(+)-(3,5-dioxa-4-phosphacyclohepta[2,1-a;3,4-a']dinaphthalen-4-yl)dimethylamine ((*R*)-MonoPhos, 0.2 mmol, 20 mol%) were added to a dried 50 mL Schlenk round bottom flask (RBF). 20 mL of 1,3-dimethyl-2-imidazolidinone (DMI) were added and the reaction was stirred for 5 min at rt. Subsequently, first the respective NBD (1.3 mmol, 1.3 equiv.) and then the respective BCB (1.0 mmol, 1.0 equiv.) were added, after which the reaction was transferred to a preheated (80 °C) oil bath and stirred for 16 h. The reaction was diluted with ethyl acetate (EA) (20 mL) and washed once with HCl$_{(aq.)}$ (1M, 50 mL). The aqueous phase was extracted with EA (3 × 20 mL) and the combined organic phases were washed once with brine (50 mL), dried over MgSO$_4$, followed by the removal of solvent *in vacuo*. The crude product was purified by flash column chromatography.

General Procedure B (GP-B): Nickel catalyzed insertion of *ortho* substituted benzocyclobutanones (BCB´s) into norbornadienes (NBD´s) for product isolation:

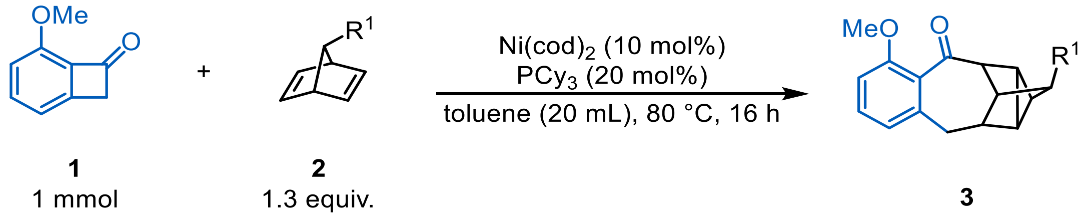

In a glove box, Ni(COD)$_2$ (0.1 mmol, 10 mol%) and PCy$_3$ (0.2 mmol, 20 mol%) were added to a dried 50 mL Schlenk round bottom flask (RBF). 20 mL of toluene were added and the reaction was stirred for 5 min at rt. Subsequently, first the respective NBD (1.3 mmol, 1.3 equiv.) and then **1** (1.0 mmol, 1.0 equiv.) were added, after which the reaction was transferred to a preheated (100 °C) oil bath and stirred for 16 h. The reaction was diluted with ethyl acetate (EA) (20 mL) and washed once with HCl$_{(aq.)}$ (1M, 50 mL). The aqueous phase

was extracted with EA (3 × 20 mL) and the combined organic phases were washed once with brine (50 mL), dried over $MgSO_4$, followed by the removal of solvent *in vacuo*. The crude product was purified by flash column chromatography.

General Procedure C (GP-C): Nickel catalyzed insertion of benzocyclobutanones (BCB´s) into norbornadienes (NBD´s) for screening reactions:

In a glove box, the respective nickel source (5 – 20 µmol, 5 – 20 mol%) and ligand were added to a dried Schlenk tube. The respective solvent (50.0 mM) was added, and the reaction was stirred for 5 min at rt. Subsequently, first NBD (100 – 300 µmol, 1.3 – 3.0 equiv.) and then the respective BCB (100 – 300 µmol, 1.0 equiv.) were added, after which the reaction was transferred to a preheated metal block and stirred for the respective time. *n*-Pentadecane (30 – 50 µL) was added after which the reaction was diluted with EA (2 mL) and quenched with $HCl_{(aq.)}$ (1M, 2 mL). The organic phase was separated and filtered through Celite, Al_2O_3 and $MgSO_4$, before being quantified by GC-FID.

General Procedure D (GP-D): Weinreb amide formation:

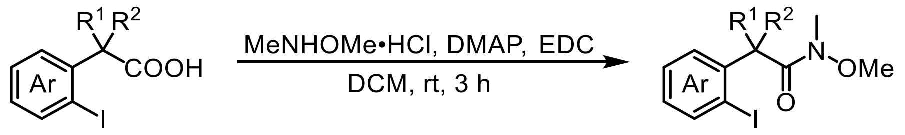

Weinreb amides were synthesized according to literature.[3] 2-(2-Iodophenyl)acetic acid derivatives (2.9 – 75.2 mmol, 1.0 equiv.) and *N*,*O*-dimethylhydroxylamine hydrochloride (4.4 – 113.3 mmol, 1.5 equiv.) were dissolved in DCM (25 mL) in a RBF. Subsequently, DMAP (4.4 – 113.3 mmol, 1.5 equiv.) and EDC (4.4 – 113.3 mmol, 1.5 equiv.) were added and the reaction was stirred for 3 h at rt. Afterwards, the mixture was diluted with 1 M $HCl_{(aq.)}$ (25 mL) and the aqueous phase was extracted with DCM (3 × 20 mL). The combined organic phases were washed with 1 M $HCl_{(aq.)}$ (20 mL), $NaHCO_{3(aq.)}$ (20 mL), and brine (20 mL) and dried over Na_2SO_4. The filtrate was concentrated *in vacuo* and purified *via* column chromatography.

General Procedure E (GP-E): Benzylic substituted Weinreb amide formation:

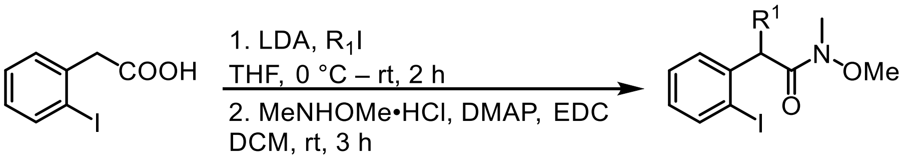

Substitution of the benzylic position was carried out following literature procedure.[3] In a predried Schlenk RBF, a THF (1 M) solution of 2-(2-iodophenyl)acetic acid (10.0 – 11.4 mmol, 1.0 equiv.) was added dropwise into freshly prepared LDA (22.0 – 25.1 mmol, 2.2 equiv., THF, 0.7 M) at 0 °C. The mixture was stirred for 1 h at 0 °C before the alkyl iodide (50.0 – 57.0 mmol, 5.0 equiv.) was slowly added. The reaction was allowed to warm to rt and was stirred for 2 h. Subsequently, the reaction was quenched with 0.5 M $NH_4OAc_{(aq.)}$ (15 mL). Afterwards, 6 M $HCl_{(aq.)}$ (7.5 mL) was added and the aqueous phase was extracted wit EA (3 × 30 mL). The combined organic phases were washed with sat. $NaS_2O_{3(aq.)}$ (45 mL), brine (45 mL) and dried over Na_2SO_4. The solvent was removed *in vacuo* and the product was transformed to the respective Weinreb amide according to **GP-D** without further purification.

General Procedure F (GP-F): Palladium catalyzed *ortho* iodination of phenyl acetic acids followed by Weinreb amide formation:

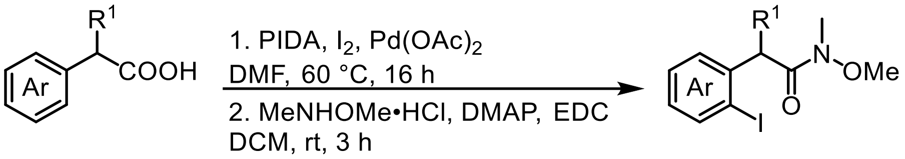

The palladium catalyzed *ortho* iodination of phenyl acetic acids was conducted according to literature.[4] In a 50 mL RBF, the phenylacetic acid derivative (10.0 mmol, 1.0 equiv.), $Pd(OAc)_2$ (0.5 mmol, 5 mol%), (Diacetoxyiodo)benzene (PIDA, 7.5 mmol, 0.75 equiv.), I_2 (7.5 mmol, 0.75 equiv.) were dissolved in anhydrous DMF (0.17 M) under air. The reaction was stirred at 60 °C for 16 h under light exclusion. Subsequently, the mixture was quenched with Na_2SO_3, and the aqueous phase was extracted wit EA (3 × 15 mL), concentrated *in vacuo* and the crude product was directly transformed to the respective Weinreb amide according to **GP-D**.

General Procedure G (GP-G): Synthesis of Kobayashi aryne precursors:

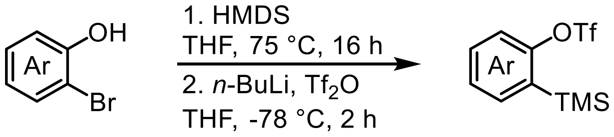

Kobayashi precursors were synthesized according to literature.[5] In a predried Schlenk RBF, Hexamethyldisilazane (HMDS, 24.6 – 28.9 mmol, 1.05 equiv.) was added to a THF (0.3 M)

solution of the *o*-bromo phenol derivate (23.5 – 27.5 mmol, 1.0 equiv.) at rt, after which the solution was stirred at 75 °C for 16 h. Subsequently, all volatiles were removed *in vacuo* and the residue was redissolved in THF (0.15 M) and cooled to -78 °C. *n*-BuLi (35.3 – 41.3 mmol, 2.5 M in *n*-hexane, 1.5 equiv.) was added dropwise over 5 min and the mixture was stirred for 1 h. Afterwards Tf_2O (28.2 – 33.0 mmol, 1.2 equiv.) was added and the mixture was stirred for 1 h. The reaction was quenched with sat. $NaHCO_{3(aq.)}$ (20 mL) and the aqueous phase was extracted with EA (3 × 30 mL). The combined organic extracts were washed with brine (1 × 30 mL), dried over Na_2SO_4 and the solvent was removed *in vacuo*. The crude material was purified *via* column chromatography.

General Procedure H (GP-H): Synthesis of BCB´s from 2-(2-iodophenyl)acetic acid Weinreb amides:

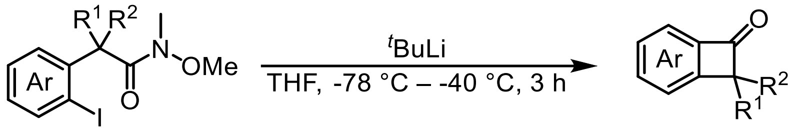

BCB´s were synthesized following a literature procedure with slight modifications.[6] In a predried Schlenk RBF, the respective 2-(2-iodophenyl)acetic acid Weinreb amide (1.9 – 72.5 mmol, 1.0 equiv.) was dissolved in THF (0.3 M) and cooled to -78 °C. *t*BuLi (4.0 – 152.3 mmol, 1.7 M in *n*-pentane, 2.1 equiv.) was added dropwise over 10 min, the mixture was allowed to warm to -40 °C and stirred for 2 h at -40 °C. Afterwards, sat. $NH_4Cl_{(aq.)}$ (12 mL) was added to quench the reaction and the aqueous phase was extracted with EA (3 × 20 mL). The combined organic phases were washed with brine (25 mL) and dried over $MgSO_4$. The filtrate was concentrated *in vacuo* and purified *via* flash chromatography.

General Procedure I (GP-I): Synthesis of BCB´s *via* [2+2] cycloaddition:

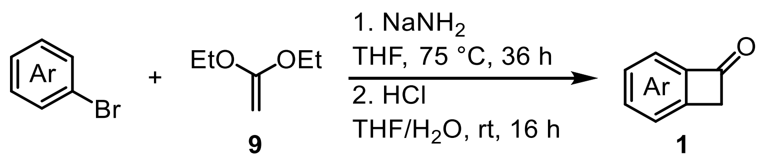

BCB´s were synthesized following a literature procedure with slight modifications.[7] In a predried Schlenk RBF, aryl bromide (20.0 mmol, 1.0 equiv.) and $NaNH_2$ (40.0 mmol, 2.0 equiv.) were suspended in THF (1 M). **9** (80.0 mmol, 4.0 equiv.) was added, and the reaction mixture was heated to 75 °C for 16 h. Reaction progress was monitored by GC-MS. If aryl bromide was still present $NaNH_2$ (20.0 mmol, 2.0 equiv.) was added, and the reaction

was stirred at 75 °C for further 24 h. The cooled mixture was poured on ice, and the organic phase was separated. The aqueous phase was extracted with DCM (3 × 30 mL) and the combined organic phases were washed with brine (1 × 30 mL), after which the solvent was removed *in vacuo*. The obtained black tar was dissolved in equal parts THF and H_2O (0.5 M). $HCl_{(aq.)}$ (conc., 80 mmol, 4 equiv.) was added and the reaction mixture was stirred at rt for 16 h. The organic phase was separated, and the aqueous phase was extracted with DCM (3 × 30 mL). The combined organic extracts were washed with brine (1 × 30 mL), dried over $MgSO_4$ and the solvent was removed *in vacuo*. The crude product was purified *via* column chromatography.

Note: After the first aqueous workup, solids might be present, which can be filtered off via silica plug.

General Procedure J (GP-J): Synthesis of BCB´s *via* [2+2] cycloaddition employing Kobayashi precursors:

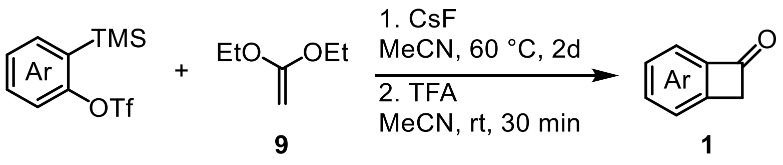

BCB´s were synthesized following a literature procedure.[5] In a Schlenk RBF, CsF (14.4 – 17.3 mmol, 1.5 equiv.) was suspended in MeCN (0.2 M). Subsequently, Kobayashi precursor (9.6 – 11.5 mmol, 1.0 equiv.), and **9** (19.2 – 23 mmol, 2.0 equiv.) were added, and the reaction was stirred at 60 °C for 2 d. The reaction mixture was cooled to rt, TFA (19.2 – 23 mmol, 2.0 equiv.) was added and the mixture was stirred for 30 min. Subsequently, EA (15 mL) and H_2O (10 mL) were added. The organic phase was separated, and the aqueous phase was extracted with EA (3 × 30 mL). The combined organic extracts were dried over $MgSO_4$ and the solvent was removed *in vacuo*. The crude product was purified *via* column chromatography.

General Procedure K (GP-K): Synthesis of 7-substituted NBD´s:

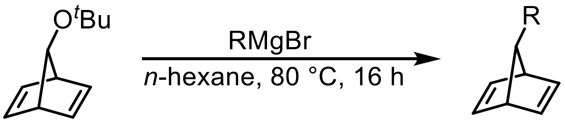

2b **2**

7-substituted NBD´s were synthesized according to literature with slight modifications.[8] In a Schlenk RBF RMgBr (24.0 mmol, 2.0 equiv., 2 M in Et_2O) was added to a solution of **2b**

(12.0 mmol, 1.0 equiv.) in *n*-hexane. At 40 °C with a N_2 stream, the Et_2O was removed (2 h). Subsequently, *n*-hexane (4 mL) was added and the reaction was heated to 80 °C for 16 h. The cooled reaction was quenched with sat. $NH_4Cl_{(aq.)}$ (5 mL) and the aqueous phase was extracted with Et_2O (3 × 5 mL). The combined organic phases were washed with brine (10 mL), dried over $MgSO_4$ and the solvent was carefully removed *in vacuo*. Subsequently the product was purified by vacuum distillation.

3 Optimization

3.1 Optimization of the standard reaction

Ligand screening

Table S 1. Ligand screening for the nickel catalyzed NBD insertion into **1a**.

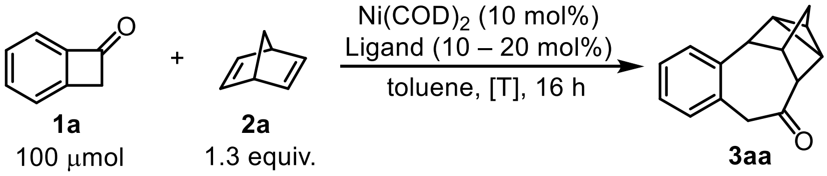

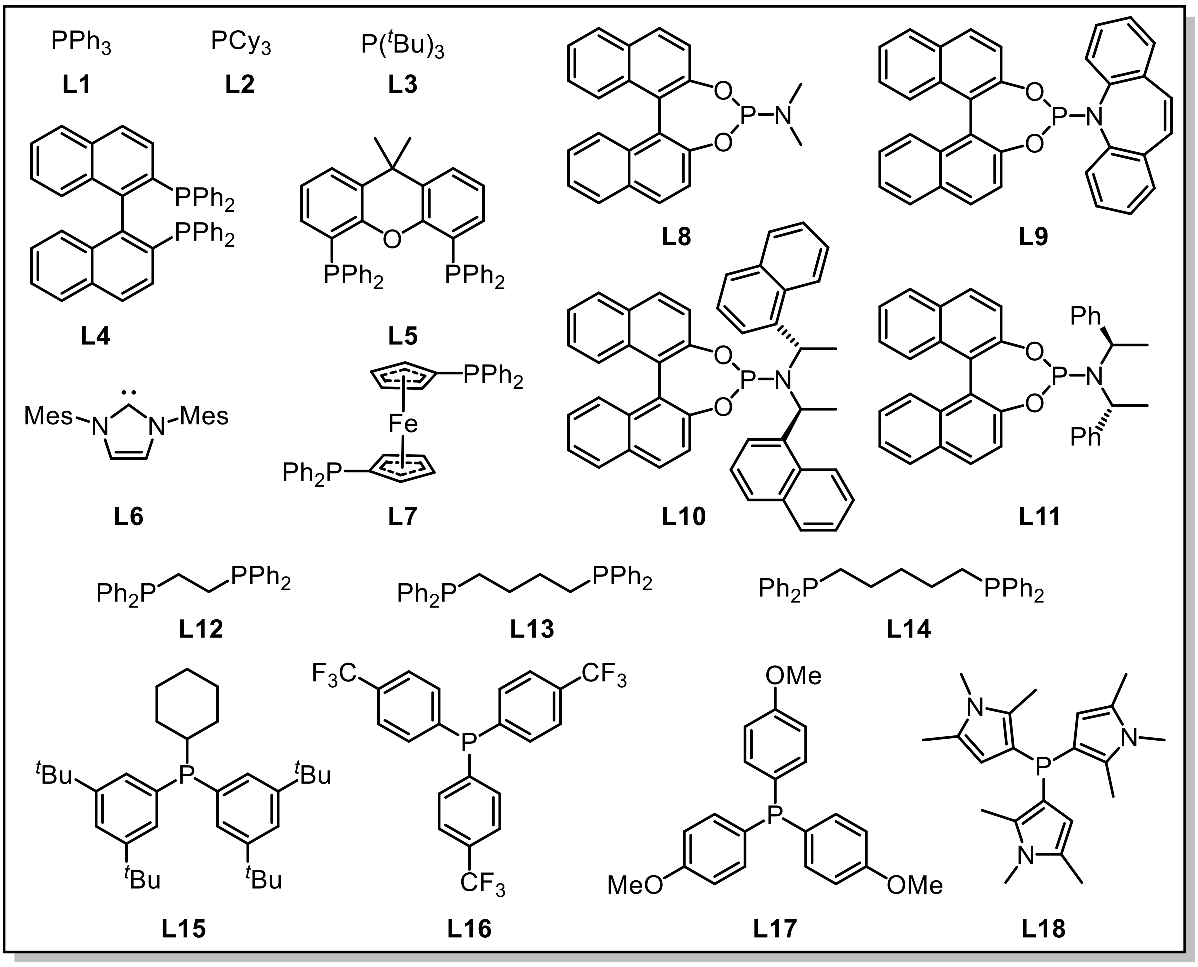

Entry	Ligand	T (°C)	Yield[a] (%)	Conversion[a] (%)
1	dppb (**L13**)	50	44	83
2	dppb (**L13**)	80	55	100
3	P(*p*-OMeC_6H_4)$_3$ (**L17**)	50	33	100
4	P(*p*-OMeC_6H_4)$_3$ (**L17**)	40	36	91
5	Tyrannophos (**L15**)	50	30	81

6	Tyrannophos (**L15**)	80	51	100
7	MonoPhos (**L8**)	50	30	70
8	MonoPhos (**L8**)	80	61	100
9	**L9**	80	12	36
10	**L10**	80	28	100
11	**L11**	80	23	47
12	PPh_3 (**L1**)	50	28	100
13	dpppe (**L14**)	50	28	76
14	dpppe (**L14**)	80	50	100
15	dppf (**L7**)	50	22	64
16	BINAP (**L4**)	50	16	70
17	dppe (**L12**)	50	12	95
18	P(*p*-$CF_3C_6H_4)_3$ (**L16**)	50	11	56
19	tmp (**L18**)	50	8	59
20	PCy_3 (**L2**)	50	4	79
21	Xantphos (**L5**)	50	0	51
22	$P(^tBu)_3$ (**L3**)	50	0	31
23	IMes (**L6**)	50	0	35
24	/	50	0	36

Reaction conditions: **1a** (100 µmol, 1.0 equiv.), NBD (1.3 equiv.), $Ni(COD)_2$ (10 mol%), monodentate ligand (20 mol%) / bidentate ligand (10 mol%), toluene (2 mL). [a]GC-FID yields.

A total of 18 ligands were evaluated over a temperature range of 40 – 80 °C. Among these, dppb (**L13**), P(*p*-$OMeC_6H_4)_3$ (**L17**), Tyrannophos (**L15**), MonoPhos (**L8**) and dpppe (**L14**) demonstrated moderate performance, achieving yields of 28 – 44%) (Table S 1, entries 1, 3, 5, 7 and 13). While PPh_3 (**L1**, Table S 1, entry 12) provided a comparable yield of 28%, it displayed complete conversion, indicating the formation of decomposition products. Consequently, ligands **L8**, **13** – **15** were selected to be investigated at a higher temperature of 80 °C, resulting in enhanced yields of 50 – 60% (Table S 1, entries 2, 6, 8 and 14). In contrast, **L17**, which showed full conversion at 50 °C (Table S 1, entry 3), was screened at 40 °C, achieving a yield of 36% (Table S 1, entry 4).

The highest yield (60%) was obtained using the phosphonamidite ligand MonoPhos (Table S 1, entry 8). This prompted the screening of additional phosphonamidite ligands (Table S 1, entries 9 – 11), though all exhibited diminished yields compared to MonoPhos.

Aliphatic bidentate ligands showed promising yields, reaching up to 44% at 50 °C (Table S 1, entry 1). The longer-chain ligands dppb and dpppe (Table S 1, entries 1 and 13) outperformed the short-bridged dppe ligand (Table S 1, entry 17), potentially due to increased flexibility enabling monodentate-like coordination. In contrast, other bidentate ligands such as dppf, BINAP or Xantphos yielded poor results (Table S 1, entries 15, 16 and 21).

Monodentate electron-rich ligands generally afforded poor or negligible yields (Table S 1, entries 19, 20, 22, 23). Similarly, the electron-deficient ligand **L16** provided only a low yield (Table S 1, entry 18). Finally, in the absence of a ligand, no insertion product was observed (Table S 1, entry 24).

Temperature – ligand and catalyst loading screening

As an increased yield was observed by increasing the temperature, a temperature screening was conducted employing the MonoPhos ligand (Table S 2, entries 1 – 5). Hereby, the optimal temperature range at 16 h reaction time was identified as 80 – 90 °C.

Variation of catalyst loading showed minimal impact on the reaction outcome (Table S 2, entries 6 and 7). However, reproducibility issues prompted the selection of a standard catalyst loading of 10 mol% for subsequent experiments. In contrast, altering the ligand loading substantially influenced the catalytic performance.

When the Ni(0) source was replaced with either Ni(^{t}BuStb)$_3$ or $Ni(CO)_2(PPh_3)_2$, yields decreased substantially to 28% and 11%, respectively (Table S 2, entries 10, 11). Attempts to enhance the yield by employing a large excess of NBD (5.0 equiv.) or adjusting the substrate-to NBD ratio (1.3 equiv. of substrate to 1.0 equiv. of NBD) resulted in only modest improvements (Table S 2, entries 12, 13).

Table S 2. Temperature – ligand and catalyst loading screening for the nickel catalyzed NBD insertion into **1a**.

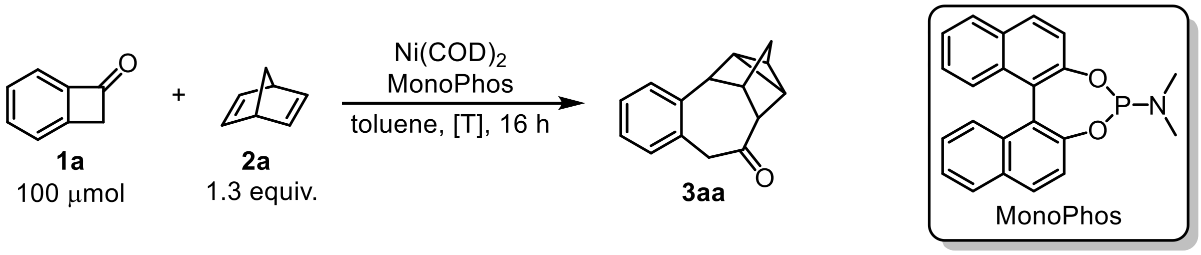

Entry	Catalyst loading (mol%)	Ligand loading (mol%)	T (°C)	Yield[a] (%)	Conversion[a] (%)
1	10	20	60	45	79

2	10	20	70	53	99
3	10	20	80	61	100
4	10	20	90	59	100
5	10	20	100	48	100
6	20	40	80	64	100
7[b]	5	10	80	63	100
8	10	10	80	44	82
9	10	30	80	14	47
10[c]	10	20	80	28	57
11[d]	10	0	80	11	33
12[e]	10	20	80	63	100
13[f]	10	20	80	74	n. d.

Reaction conditions: **1a** (100 µmol, 1.0 equiv.), NBD (1.3 equiv.), toluene (2 mL). [a]GC-FID yields. [b] **1a** (200 µmol, 1.0 equiv.). [c] $Ni(^tBuStb)_3$ instead of $Ni(COD)_2$. [d] $Ni(CO)_2(PPh_3)_2$ instead of $Ni(COD)_2$. [e] 3 equiv. NBD. [f] 1.3 equiv. **1a**.

Solvent screening

17 solvents were evaluated, with the most promising ones being screened at several temperatures and applied to the most promising ligands.

Table S 3. Solvent screening for the nickel catalyzed NBD insertion into **1a**.

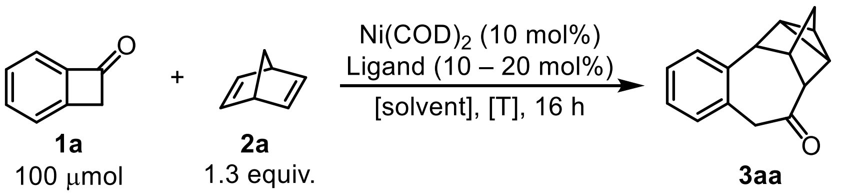

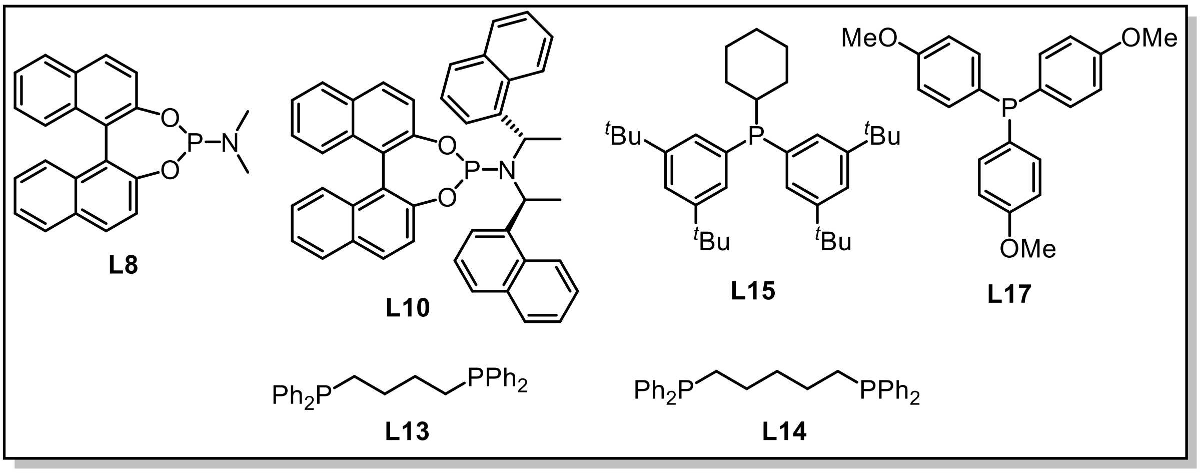

Entry	Solvent	Ligand	T (°C)	Yield (%)[a]	Conversion (%)[a]

1	DMI	MonoPhos (**L8**)	80	85	n. d.
2	DMI	Tyrannophos (**L15**)	80	25	n. d.
3	DMI	dpppe (**L14**)	80	27	n. d.
4	DMI	P(*p*-OMeC$_6$H$_4$)$_3$ (**L17**)	40	52	n. d.
5	DMI	MonoPhos (**L8**)	60	72	n. d.
6	DMI	MonoPhos (**L8**)	100	28	n. d.
7	DMPU	MonoPhos (**L8**)	80	37	68
8	DMF	MonoPhos (**L8**)	80	78	100
9	DMF	Tyrannophos (**L15**)	80	74	100
10	DMF	dppb (**L13**)	80	62	100
11	DMF	dpppe (**L14**)	80	74	100
12	DMF	**L10**[b]	80	17	100
13	DMF	P(*p*-OMeC$_6$H$_4$)$_3$ (**L17**)	40	61	100
14	DMF	P(*p*-OMeC$_6$H$_4$)$_3$ (**L17**)	25	11	31
15	DMA	MonoPhos (**L8**)	80	71	100
16	*n*-hexane	MonoPhos (**L8**)	80	73	100
17	*n*-hexane	Tyrannophos (**L15**)	80	62	100
18	*n*-hexane	dppb (**L13**)	80	60	100
19	*n*-hexane	dpppe (**L14**)	80	63	100
20	*n*-hexane	**L10**[b]	80	50	100
21	*n*-hexane	P(*p*-OMeC$_6$H$_4$)$_3$ (**L17**)	40	52	100
22	*n*-hexane	MonoPhos (**L8**)	30	41	58
23	*n*-hexane	MonoPhos (**L8**)	60	71	100
24	*n*-hexane	MonoPhos (**L8**)	70	66	100
25	*n*-hexane	MonoPhos (**L8**)	90	72	100
26	*n*-hexane	MonoPhos (**L8**)	100	71	100
27[c]	*n*-octane	MonoPhos (**L8**)	80	80	100
28	*n*-octane	**L10**[b]	80	48	100
29	*n*-octane	MonoPhos (**L8**)	60	52	78
30	*n*-octane	MonoPhos (**L8**)	100	51	83
31	*n*-pentane	MonoPhos (**L8**)	80	63	100
32	1,4-dioxane	MonoPhos (**L8**)	80	70	100
33	1,4-dioxane	Tyrannophos (**L15**)	80	62	100

34	1,4-dioxane	dppb (**L13**)	80	55	100
35	1,4-dioxane	dpppe (**L14**)	80	66	100
36	1,4-dioxane	**L10**[b]	80	29	49
37	1,4-dioxane	P(*p*-OMeC_6H_4)$_3$ (**L17**)	40	61	100
38	1,4-dioxane	MonoPhos (**L8**)	60	68	100
39	1,4-dioxane	MonoPhos (**L8**)	70	69	100
40	1,4-dioxane	MonoPhos (**L8**)	90	70	100
41	1,4-dioxane	MonoPhos (**L8**)	100	70	100
42	2-MeTHF	MonoPhos (**L8**)	80	50	73
43	THF	MonoPhos (**L8**)	80	23	32
44	Et_2O	MonoPhos (**L8**)	80	49	100
45	MTBE	MonoPhos (**L8**)	80	46	100
46	DME	MonoPhos (**L8**)	80	0	0
47	EtOH	MonoPhos (**L8**)	80	72	100
48	Toluene	MonoPhos (**L8**)	80	61	100
49[d]	MeCN	MonoPhos (**L8**)	80	25	51
50	DCE	MonoPhos (**L8**)	80	6	20

Reaction conditions: **1a** (100 µmol, 1.0 equiv.), NBD (1.3 equiv.), Ni(COD)$_2$ (10 mol%), Ligand (modentate: 10 mol%, bidentate: 20 mol%), solvent (2 mL). [a]GC-FID yields. [b] **L10** (12 mol%). [c] **1** (85 µmol, 1.0 equiv.), NBD (1.3 equiv.), Ni(COD)$_2$ (10 mol%), **L8** (20 mol%), *n*-octane (2 mL). [d] **1** (100 µmol, 1.0 equiv.), NBD (1.3 equiv.), Ni(COD)$_2$ (10 mol%), **L8** (20 mol%), MeCN (2.5 mL).

The highest yield of 85% was achieved using DMI as the solvent with the MonoPhos ligand (Table S 3, entry 1). Attempts to vary the temperature resulted in decreased yields (Table S 3, entries 5, 6). Interestingly, substituting DMI with the structurally similar solvent DMPU led to a substantial drop in yield to 37% (Table S 3, entry 7). Using DMF, another solvent with an amide group, resulted in 78% yield (Table S 3, entry 8). Comparable yields were obtained with other promising ligands in DMF (Table S 3, entries 9–11, 13). The structurally similar solvent **DMA** provided analoglous results to DMF for the MonoPhos ligand (Table S 3, entry 15).

When the solvent was changed to the nonpolar *n*-hexane, MonoPhos afforded a yield of 73% (Table S 3, entry 16). Other ligands produced slightly lower yields in *n*-hexane (Table S 3, entries 17–21), though the 50% yield observed with **L10** was notable, as this ligand holds potential for improved enantioselectivity.[9] Varying the reaction temperature between 30 °C and 100 °C with MonoPhos (Table S 3, entries 22–26) revealed consistent yields between

60 °C and 100 °C, while decreasing the temperature to 30 °C led to a decline in yield, in correspondence with lower conversion rates.

While screening etheric solvents, 1,4-dioxane emerged as the most promising, yielding 70% of the product at 80 °C with MonoPhos (Table S 3, entry **32**). Other promising ligands showed slightly reduced yields under the same conditions (Table S 3, entries 33–37). Temperature variation between 60 °C and 100 °C in 1,4-dioxane did not affect the yield (Table S 3, entries 38–41) substantially. In contrast, switching to 2-MeTHF reduced the yield to 50% (Table S 3, entry 42), while THF resulted in a much lower yield of 23% (23%, Table S 3, entry 43). Solvents such as Et_2O and MTBE yielded approximately 50% (Table S 3, entries 44, 45), whereas DME completely inhibited the reaction (Table S 3, entry 46).

Polar protic EtOH, performed surprisingly well, yielding 72% (Table S 3, entry 47). In comparison, MeCN and DCE gave poor results, with yields of only 25% and 6%, respectively (Table S 3, entries 49, 50).

Table S 4. Screening for the Ni catalyzed NBD insertion into **1a** at 300 µmol scale.

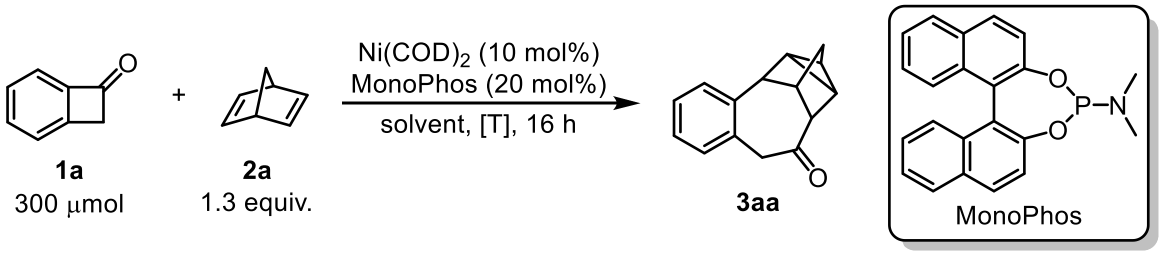

**Entry**	**Solvent**	**T [°C]**	**Yield (%)**[a]	**Conversion (%)**[a]
1	DMI	80	91	n. d
2	DMI	70	83	n. d
3	DMI	60	85	n. d
4[b]	DMI	50	96	n. d
5[b]	DMI	40	45	n. d
6[c]	DMI	80	86	n. d
7[d]	DMI	80	74	n. d
8[d]	*n*-hexane	80	69	100
9[d]	DMF	80	68	100
10[d]	1,4-dioxane	80	59	90
11[d]	toluene	80	55	100
12[e]	DMI	80	89	n. d

13[f]	DMI	80	67	n. d
14[g]	DMI	80	69	n. d
15[h]	DMI	80	63	n. d
16[i]	DMI	80	43	n. d
17	*n*-hexane/DMF	80	78	100
18	*n*-hexane/1,4-dioxane	80	77	100
19	*n*-hexane/DMI	80	69	n. d

Reaction conditions: **1a** (300 µmol, 1.0 equiv.), NBD (1.3 equiv.), $Ni(COD)_2$ (10 mol%), MonoPhos (20 mol%), solvent (6 mL). [a]GC-FID yields. [b] 24 h. [c] 5 mol% $Ni(COD)_2$ [d] solvent (2 mL). [e] slow addition of **1a**. [f] slow addition of NBD. [g] slow addition of **1a** and NBD. [h] 12 mol% MonoPhos. [i] 5 mol% MonoPhos.

Final screening reactions were performed on a 300 µmol scale to confirming the standard conditions (Table S 4, entry 1). Additionally, it was demonstrated that a lower reaction temperature could be employed if the reaction time was extended (Table S 4, entry 4). Modifications such as increasing the reaction concentration or reducing the catalyst loading resulted in a slight decrease in product yield (Table S 4, entries 6, 7). Consequently, these conditions were deemed unsuitable for the substrate screening. Attempts to optimize the reaction through adjustments in the mode of compound addition or the use of solvent mixtures also furnished unfavourable yields (Table S 4, entries 12 – 19).

3.2 Optimization of 3-OMe BCB

Table S 5. Ligand screening for the nickel catalyzed NBD insertion into **1n**.

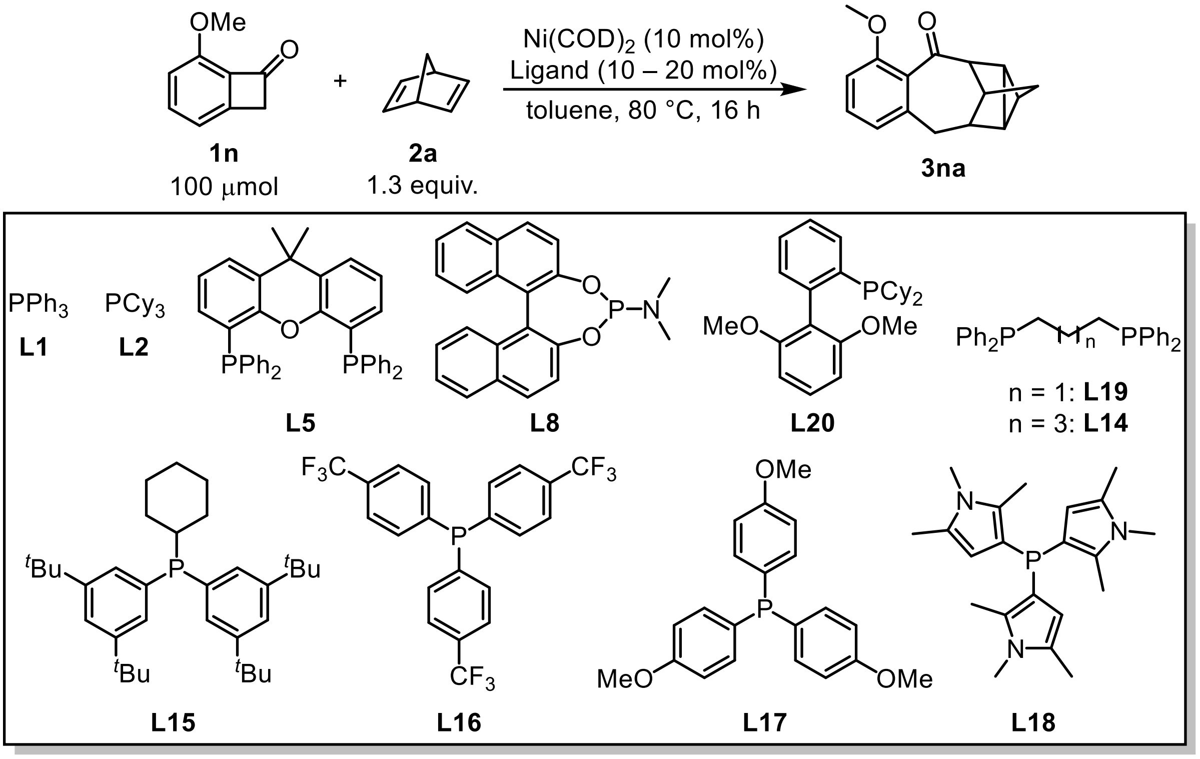

Entry	Ligand	Yield[a] (%)
1	PCy_3 (**L2**)	56
2	Tyrannophos (**L15**)	48
3	PPh_3 (**L1**)	47
4	P(*p*-$OMeC_6H_4)_3$ (**L17**)	46
5	dpppe (**L14**)	31
6	tmp (**L18**)	17
7[b]	MonoPhos (**L8**)	13
8[b]	P(*p*-$CF_3C_6H_4)_3$ (**L16**)	8
9[b]	Xantphos (**L5**)	7
10[b]	dppp (**L19**)	5
11[b]	SPhos (**L20**)	2
12	/	0

Reaction conditions: **1n** (100 μmol, 1.0 equiv.), NBD (1.3 equiv.), $Ni(COD)_2$ (10 mol%), monodentate ligand (20 mol%) / bidentate ligand (10 mol%), toluene (2 mL). [a]GC-FID yields. [b] NBD (3.0 equiv.).

The screening revealed that electron-rich ligands provided the best performance, with PCy_3 delivering the highest yield (Table S 5, entry 1). Thus, subsequent optimization efforts focused on reactions employing PCy_3 as the ligand.

Table S 6. Solvent and temperature screening for the Ni catalyzed NBD insertion into **1n**.

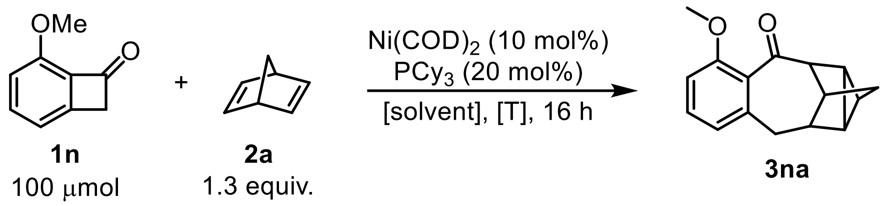

Entry	Solvent	T (°C)	Yield (%)[a]
1	toluene	80	56
2[b]	toluene	40	32
3	toluene	100	74
4[c]	toluene	100	78
5	THF	80	57
6	*n*-hexane	80	53
7	1,4-dioxane	80	51
8	DMI	80	46
9	DMF	80	44
10	EtOH	80	7

Reaction conditions: **1n** (100 µmol, 1.0 equiv.), NBD (1.3 equiv.), $Ni(COD)_2$ (10 mol%), PCy_3 (20 mol%), solvent (2 mL). [a]GC-FID yields. [b] 64 h. [c] NBD (3.0 equiv.).

A correlation between reaction temperature and yield was observed, with lower temperatures resulting in reduced, and higher temperatures in enhanced yields (Table S 6, entries 2, 3). Increasing the excess of NBD provided only a marginal improvement in yield (Table S 6, entry 4). Based on these findings, the standard reaction conditions were established as 1.3 equiv. of NBD at 100 °C in toluene (Table S 6, entry 3).

Similar to the solvent screening conducted for **1a**, several solvents were found to be tolerated with the reaction, delivering yields comparable to toluene (Table S 6, entries 5 – 9). Only, the use of the polar protic solvent EtOH resulted in a significantly lower yield, contrasting with the results observed during the optimization of the reaction with **1a**.

4 Synthesis

4.1 Weinreb amides

2-(2-iodophenyl)-*N*-methoxy-*N*-methylacetamide

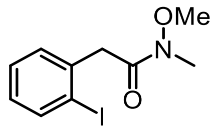

8a

8a was synthesized according to **GP-D** employing 2-(2-iodophenyl)acetic acid (19.7 g, 75.2 mmol, 1.0 equiv.), *N*,*O*-dimethylhydroxylamine hydrochloride (11.0 g, 113mmol, 1.50 equiv.), DMAP (13.8 g, 113mmol, 1.50 equiv.), and EDC (17.5 g, 113 mmol, 1.5 equiv.). Purification *via* column chromatography (80:20 *n*-hexane/EA) afforded **8a** (22.9 g, 75.2 mmol, 97%) as a yellow oil. The analytical data is in accordance with literature.[10]

$C_{10}H_{12}NO_2I$ (305.12 $\frac{\text{g}}{\text{mol}}$)

R$_f$: 0.41 (*n*-hexane/EA = 80:20) [UV]

^{1}H NMR(700.21 MHz, $CDCl_3$): δ = 7.84 (d, ^3J = 7.9 Hz, 1H), 7.31 (m, 1H), 7.28 (dd, ^3J = 7.6 Hz, ^4J = 1.7 Hz, 1H), 6.94 (m, 1H), 3.93 (s, 2H), 3.72 (s, 3H), 3.23 (s, 3H).

^{13}C NMR(176.08 MHz, $CDCl_3$): δ = 171.5, 139.4, 138.5, 130.5, 128.6, 128.4, 101.4, 61.5, 44.5, 32.5.

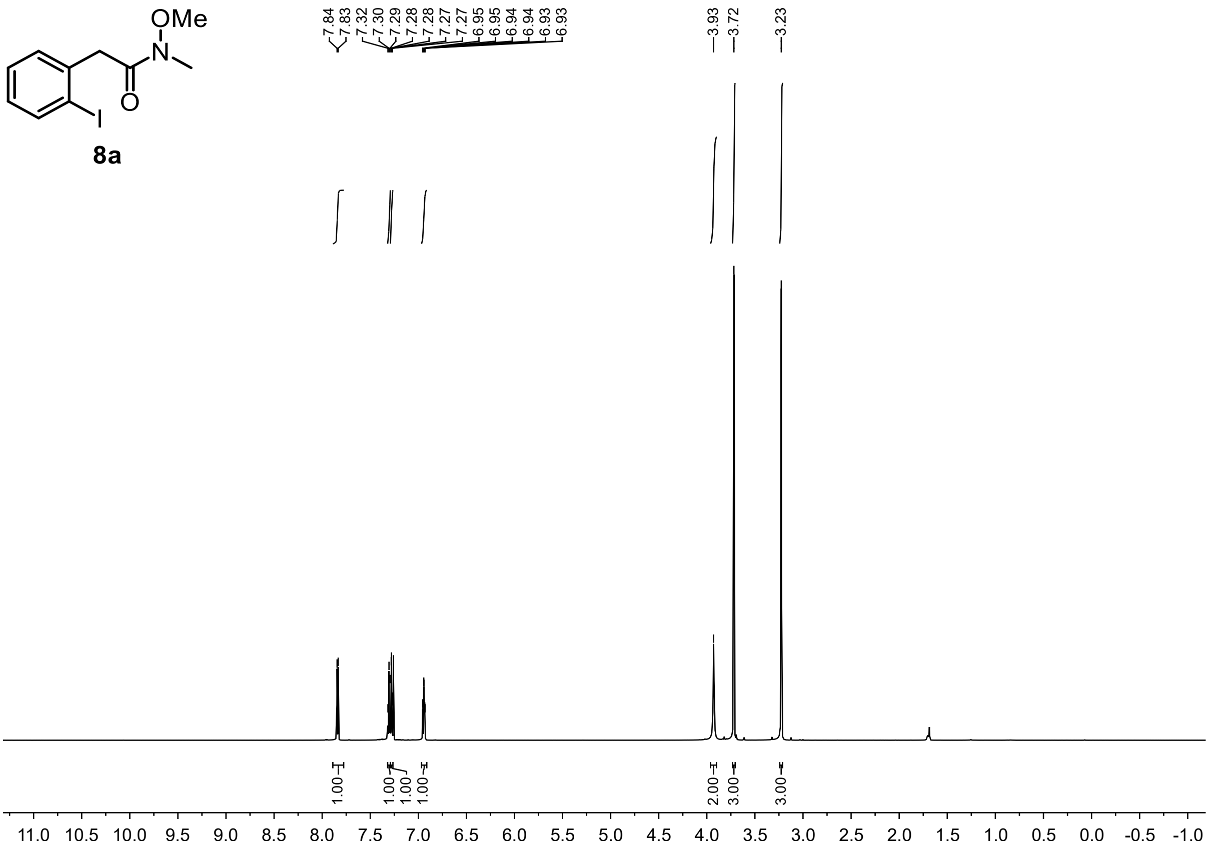

Figure S 1. ^{1}H NMR of **8a** in $CDCl_3$ measured at 700.21 MHz.

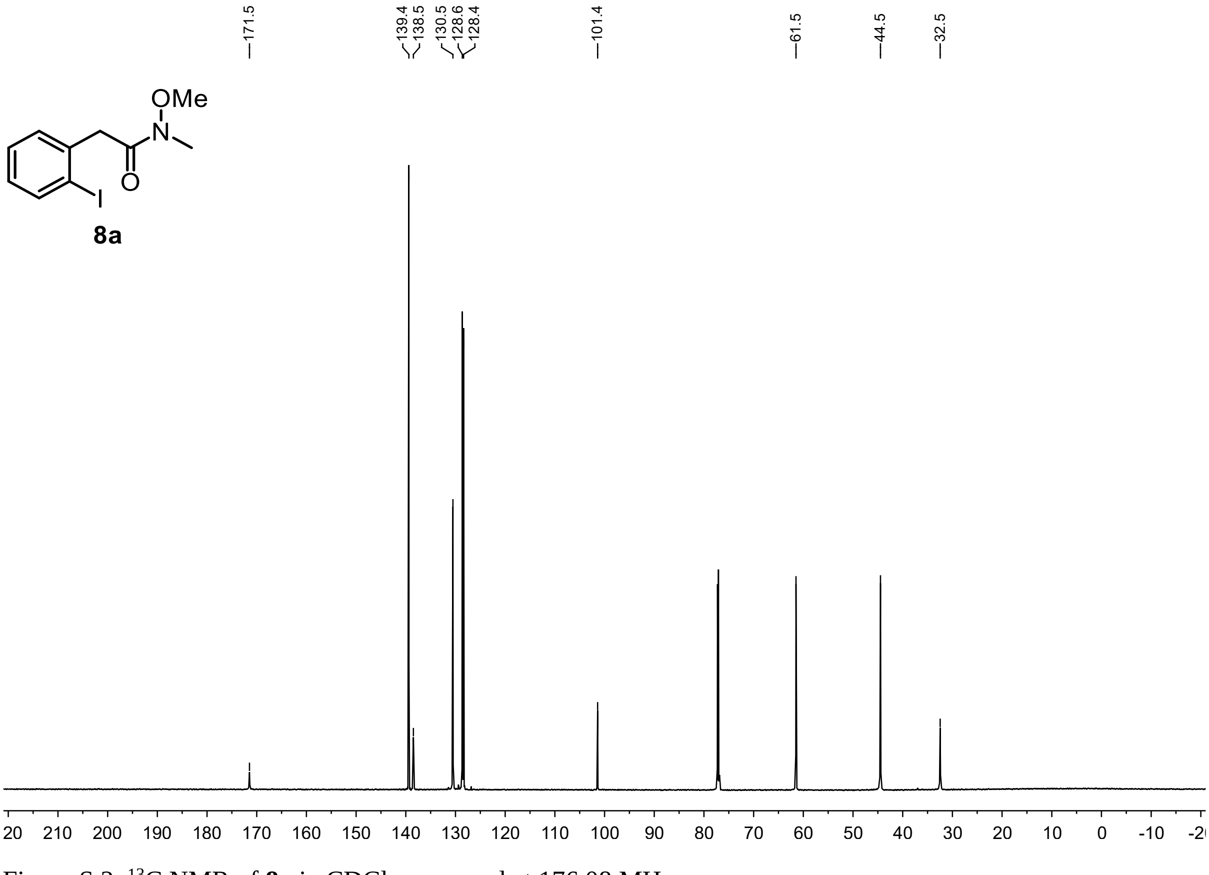

Figure S 2. ^{13}C NMR of **8a** in $CDCl_3$ measured at 176.08 MHz.

2-(2-iodo-6-methylphenyl)-*N*-methoxy-*N*-methylacetamide

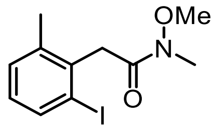

8c

8c was synthesized according to **GP-F** employing *o*-tolylacedic acid (1.50 g, 10.0 mmol, 1.00 equiv.), $Pd(OAc)_2$ (113 mg, 500 µmol, 5 mol%), PIDA (2.42 g, 7.50 mmol, 0.75 equiv.), I_2 (1.90 g, 7.50 mmol, 0.75 equiv.), N,O-dimethylhydroxylamine hydrochloride (1.50 g, 15.0 mmol, 1.50 equiv.), DMAP (1.83 g, 15.0 mmol, 1.50 equiv.), and EDC (2.33 g, 15.0 mmol, 1.50 equiv.). Purification *via* column chromatography (70:30 *n*-hexane/EA) afforded **8c** (1.18 g, 3.70 mmol, 37%) as a yellow oil over two steps.

$C_{11}H_{14}NO_2I$ (319.14 $\frac{\mathrm{g}}{\mathrm{mol}}$)

mp: 75.0 °C.

R_f: 0.27 (*n*-hexane/EA = 80:20) [UV]

^{1}H NMR(400.16 MHz, $CDCl_3$): δ = 7.70 (d, ^3J = 7.9 Hz, 1H), 7.14 (d, ^3J = 7.5 Hz, 1H), 6.85 (m, 1H), 4.05 (s, 2H), 3.80 (s, 3H), 3.24 (s, 3H), 2.33 (s, 3H).

^{13}C NMR(100.62 MHz, $CDCl_3$): δ = 171.0, 139.0, 137.3, 137.0, 130.4, 128.8, 103.4, 61.5, 42.2, 32.7, 21.7.

HRMS (APCI-TOF) m/z: $[M+H]^+$ Calcd for $C_{11}H_{14}NO_2IH$ 320.0142; Found 320.0143.

IR (ATR, ṽ): 1649 cm^{-1} (s, *CO*NMeOMe).

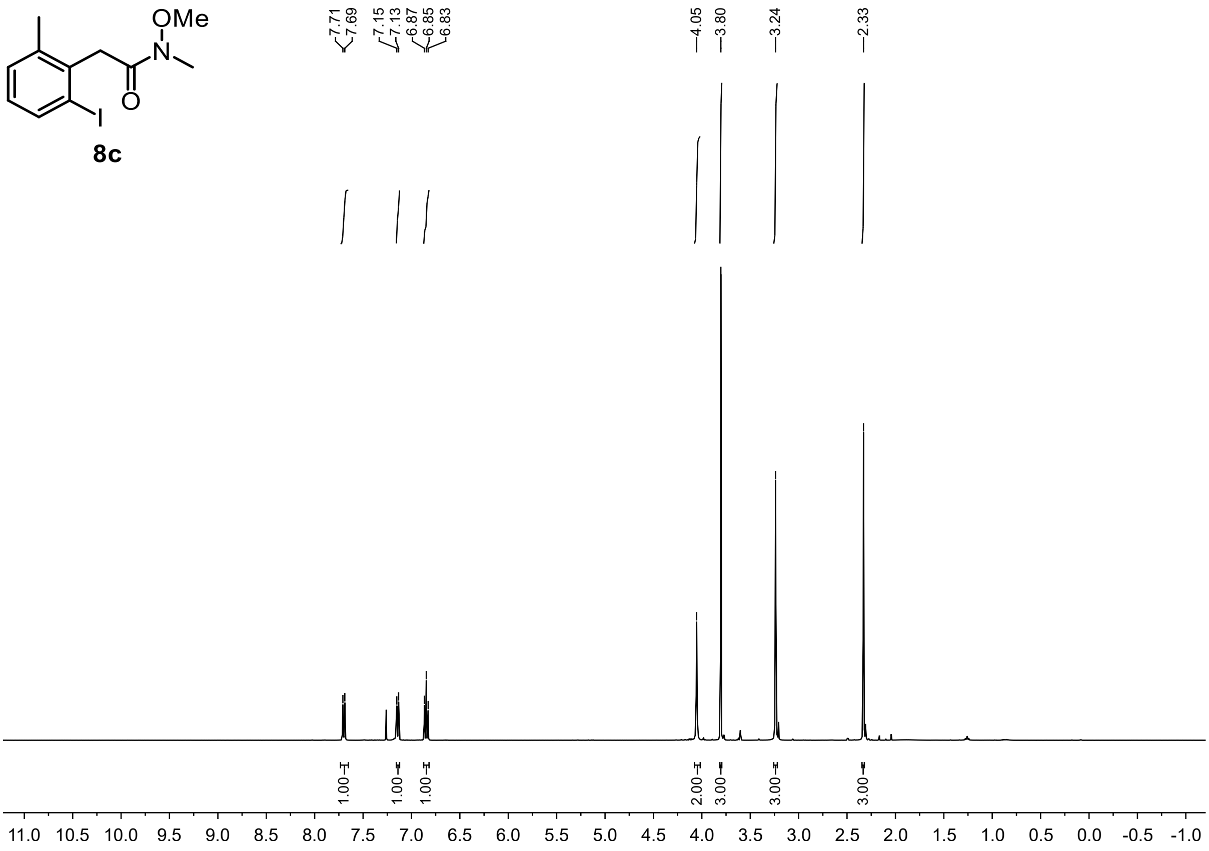

Figure S 3. ^{1}H NMR of **8c** in $CDCl_3$ measured at 400.16 MHz.

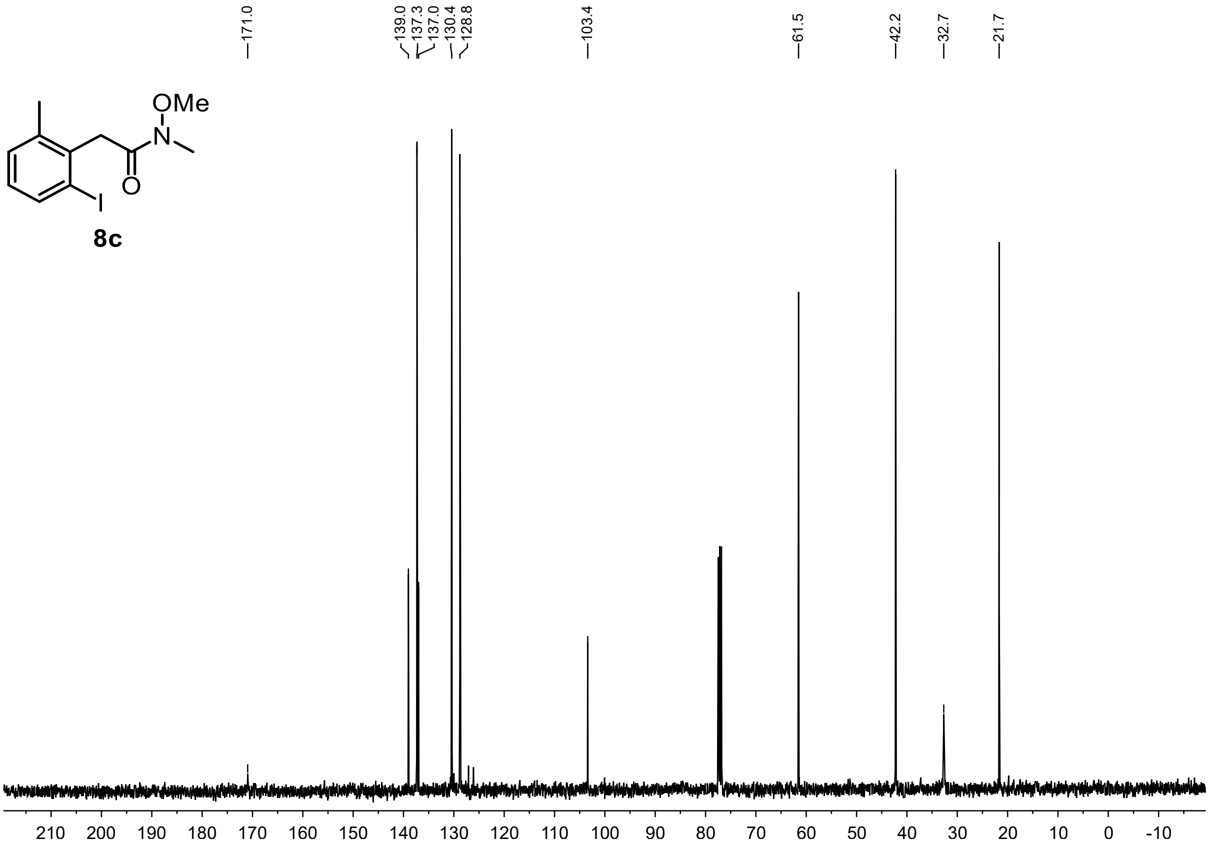

Figure S 4. ^{13}C NMR of **8c** in $CDCl_3$ measured at 100.63 MHz.

2-(2-iodophenyl)-*N*-methoxy-*N*-methylpropanamide

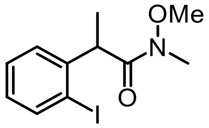

8g

8g was synthesized according to **GP-E** employing 2-(2-iodophenyl)acetic acid (2.99 g, 11.4 mmol, 1.00 equiv.), diisopropylamine (3.20 mL, 22.8 mmol, 2.00 equiv.), *n*-BuLi (9.12 mL, 2.50 M, 2.00 equiv.), methyl iodide (3.55 mL, 57.0 mmol 5.00 equiv.), N,O-dimethylhydroxylamine hydrochloride (1.67 g, 17.1 mmol, 1.50 equiv.), DMAP (2.09 g, 17.1 mmol, 1.50 equiv.), and EDC (2.65 g, 17.1 mmol, 1.50 equiv.). Purification *via* column chromatography (67:33 *n*-hexane/EA) afforded **8g** (1.92 g, 6.02 mmol, 53%) as a yellow oil. The analytical data is in accordance with literature.[11]

$C_{11}H_{14}NO_2I$ (319.14 $\frac{g}{mol}$)

R$_f$: 0.75 (*n*-hexane/EA = 80:20) [UV]

^{1}H NMR(400.16 MHz, CDCl$_3$): δ = 7.84 (d, ^3J = 8.5 Hz, 1H), 7.29 (m, 2H), 6.90 (m, 1H), 4.38 (m, 1H), 3.38 (s, 3H), 3.15 (s, 3H), 1.34 (d, ^3J = 7.0 Hz, 3H).

^{13}C NMR(176.08 MHz, CDCl$_3$): δ = 175.1, 145.0, 139.7, 128.9, 128.5, 127.4, 100.9, 61.3, 47.1, 32.6, 18.5, 1.1.

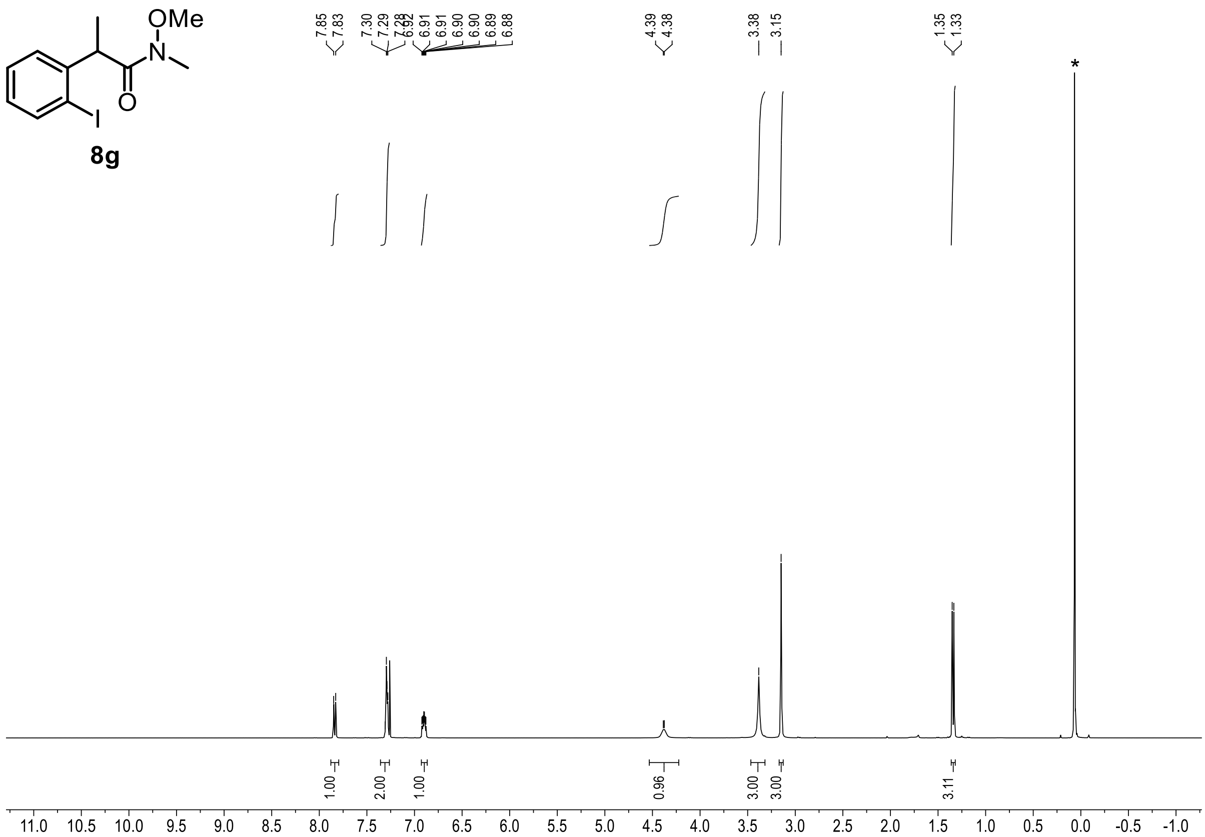

Figure S 5. ^{1}H NMR of **8g** in $CDCl_3$ measured at 700.21 MHz. * Denotes residual grease.

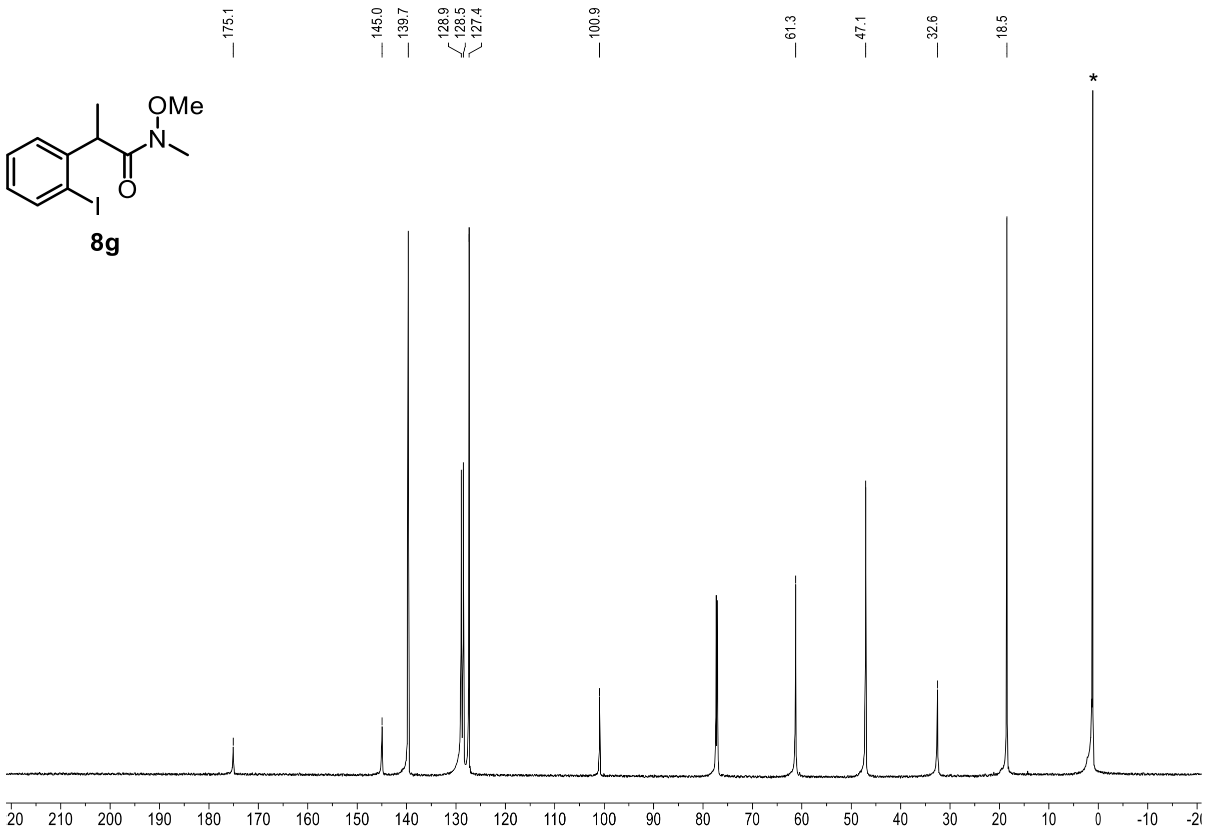

Figure S 6. ^{13}C NMR of **8g** in $CDCl_3$ measured at 176.08 MHz. * Denotes residual grease.

2-(2-iodophenyl)-*N*-methoxy-*N*-methylbutanamide

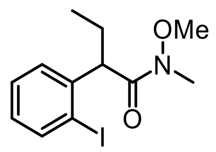

8h

8h was synthesized according to **GP-E** employing 2-(2-iodophenyl)acedic acid (2.62 g, 10.0 mmol, 1.00 equiv.), diisopropylamine (3.09 mL, 22.0 mmol, 2.20 equiv.), *n*-BuLi (13.8 mL, 1.60 M, 2.20 equiv.), ethyl iodide (4.02 mL, 50.0 mmol 5.00 equiv.), N,O-dimethylhydroxylamine hydrochloride (1.46 g, 15.0 mmol, 1.50 equiv.), DMAP (1.83 g, 15.0 mmol, 1.50 equiv.), and EDC (2.33 g, 15.0 mmol, 1.50 equiv.). Purification *via* flash chromatography (23 g SiO_2, gradient from 100:0 to 50:50 *n*-hexane/EA over 10 CV) afforded **8h** (2.53 g, 7.93 mmol, 79%) as a yellow oil. The analytical data is in accordance with literature.[3]

$C_{12}H_{16}NO_2I$ (333.17 $\frac{g}{mol}$)

R_f: 0.48 (*n*-hexane/EA = 80:20) [UV]

^{1}H NMR(400.16 MHz, $CDCl_3$): δ = 7.84 (dd, ^3J = 7.9 Hz, ^4J = 1.2 Hz, 1H), 7.34 (dd, ^3J = 7.8 Hz, ^3J = 1.7 Hz, 1H), 7.27 (m, 1H), 6.89 (m, 1H), 4.23 (m, 1H), 3.46 (s, 3H), 3.15 (s, 3H), 1.99 (m, 1H), 1.65 (m, 1H), 0.95 (t, ^3J = 7.4 Hz, 3H).

^{13}C NMR(100.62 MHz, $CDCl_3$): δ = 174.2, 143.4, 139.6, 128.8, 128.5, 127.9, 102.0, 61.5, 53.3, 32.5, 27.4, 12.5.

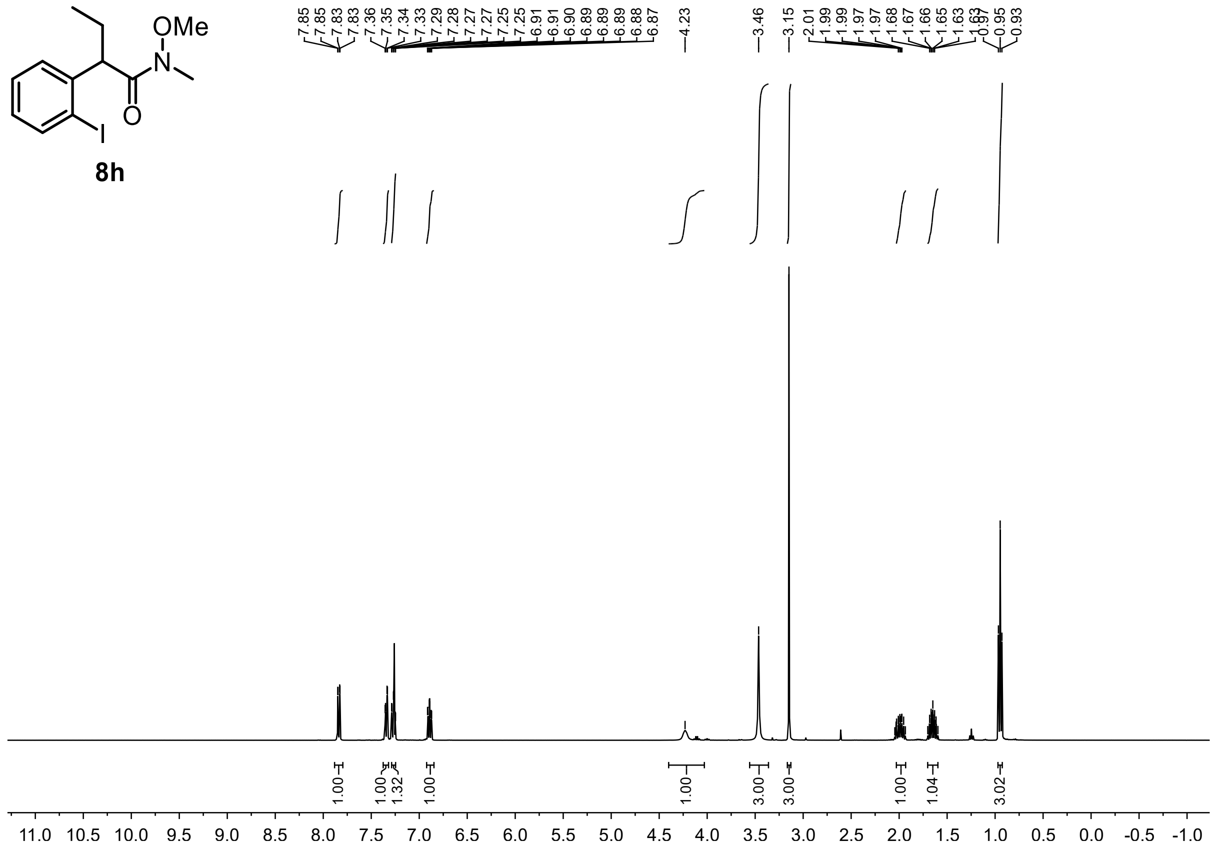

Figure S 7. ^{1}H NMR of **8h** in $CDCl_3$ measured at 400.16 MHz.

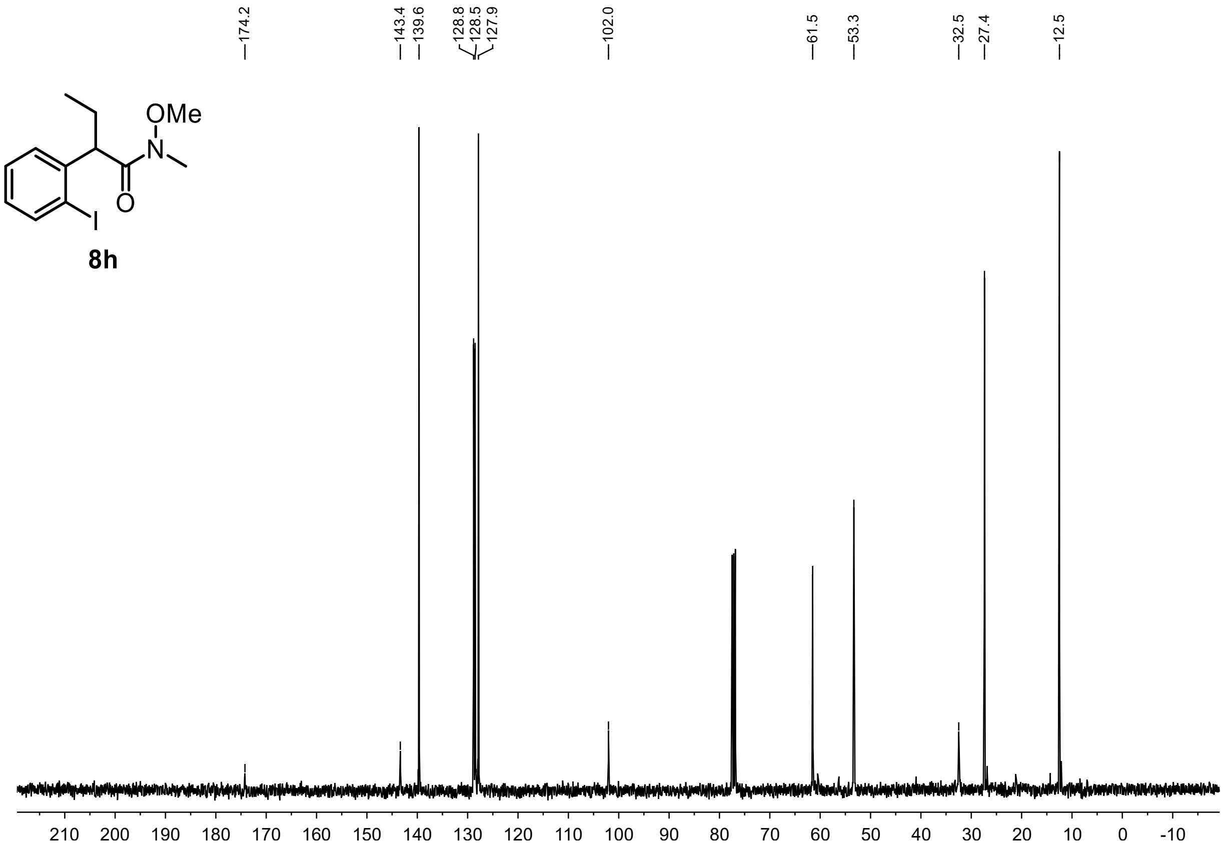

Figure S 8. ^{13}C NMR of **8h** in $CDCl_3$ measured at 100.63 MHz.

2-(2-iodophenyl)-*N*-methoxy-*N*,3-dimethylbutanamide

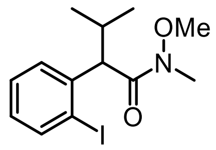

8i

8i was synthesized according to **GP-E** employing 2-(2-iodophenyl)acedic acid (2.99 g, 11.4 mmol, 1.00 equiv.), diisopropylamine (3.20 mL, 22.8 mmol, 2.00 equiv.), *n*-BuLi (9.12 mL, 2.50 M, 2.00 equiv.), isopropyl iodide (5.70 mL, 57.0 mmol 5.00 equiv.), N,O-dimethylhydroxylamine hydrochloride (1.67 g, 17.1 mmol, 1.50 equiv.), DMAP (2.09 g, 17.1 mmol, 1.50 equiv.), and EDC (2.09 g, 17.1 mmol, 1.50 equiv.). Purification *via* column chromatography (67:33 *n*-hexane/EA) afforded **8i** (1.75 g, 5.48 mmol, 48%) as a yellow oil.

$C_{13}H_{18}NO_2I$ (347.20 $\frac{g}{mol}$)

R$_f$: 0.90 (*n*-hexane/EA = 80:20) [UV]

^{1}H NMR(400.16 MHz, $CDCl_3$): δ = 7.84 (dd, ^3J = 8.0 Hz, ^4J = 1.2 Hz, 1H), 7.51 (dd, ^3J = 7.8 Hz, ^4J = 1.5 Hz, 1H), 7.29 (m, 1H), 6.90 (m, 1H), 4.10 (dm, ^3J = 10.1 Hz, 1H), 3.62 (s, 3H), 3.14 (s, 3H), 2.39 (dqq, ^3J = 10.1 Hz, ^3J = 6.9 Hz, ^3J = 6.4 Hz, 1H), 1.07 (d, ^3J = 6.4 Hz, 3H), 0.77 (d, ^3J = 6.9 Hz, 3H).

^{13}C NMR(100.62 MHz, $CDCl_3$): δ = 174.2, 142.2, 139.7, 128.8, 128.7, 128.6, 103.4, 61.9, 57.6, 33.5, 32.4, 22.0, 19.4.

HRMS (ESI-TOF): m/z $[M+Na]^+$ Calcd for $C_{13}H_{18}NO_2INa$ 370.0274; Found 370.0275.

IR (ATR, ṽ): 1653 cm^{-1} (s, *CO*NMeOMe).

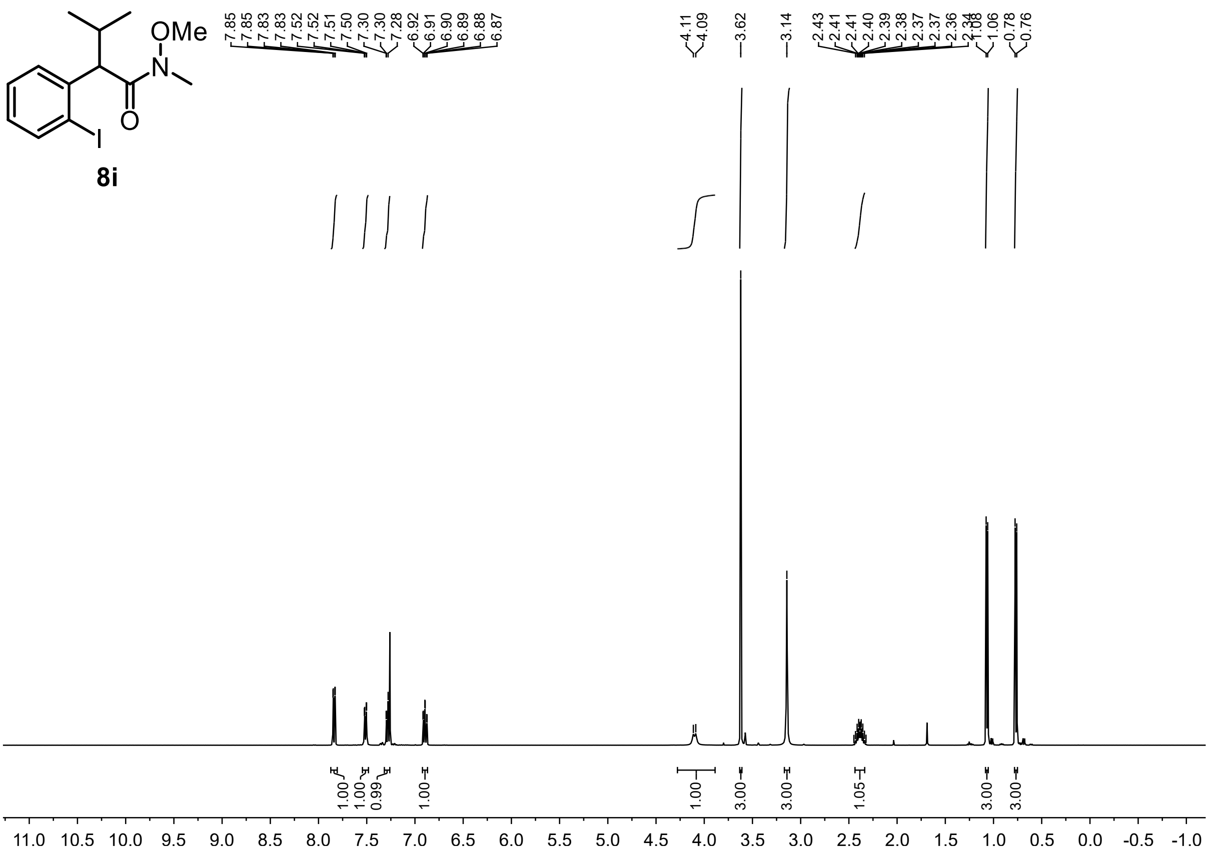

Figure S 9. ^{1}H NMR of **8i** in $CDCl_3$ measured at 400.16 MHz.

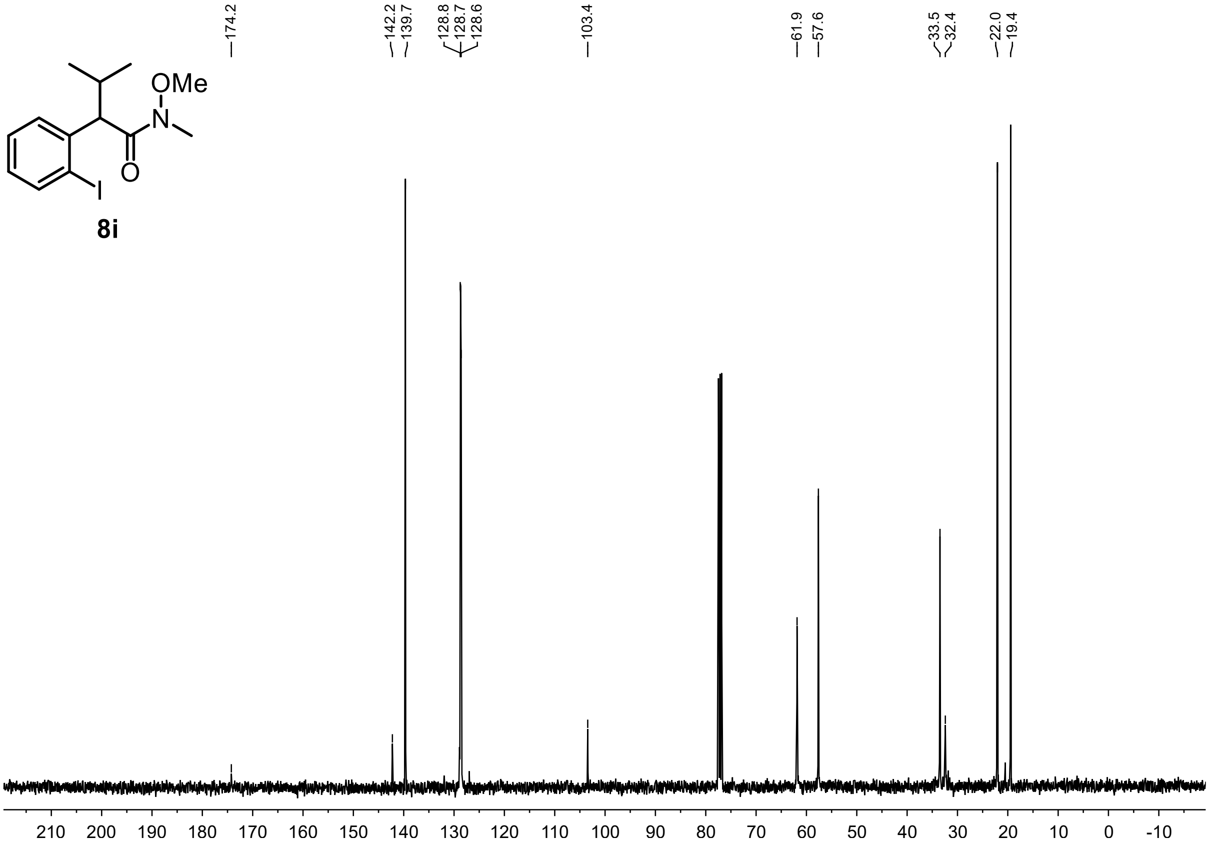

Figure S 10. ^{13}C NMR of **8i** in $CDCl_3$ measured at 100.63 MHz.

2-(2-iodophenyl)-*N*-methoxy-*N*-methylpent-4-enamide

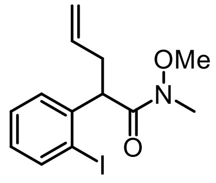

8j

8j was synthesized according to **GP-E** employing 2-(2-iodophenyl)acedic acid (2.99 g, 11.4 mmol, 1.00 equiv.), diisopropylamine (3.20 mL, 22.8 mmol, 2.00 equiv.), *n*-BuLi (9.12 mL, 2.50 M, 2.00 equiv.), allyl iodide (5.20 mL, 57.0 mmol 5.00 equiv.), N,O-dimethylhydroxylamine hydrochloride (1.67 g, 17.1 mmol, 1.50 equiv.), DMAP (2.09 g, 17.1 mmol, 1.50 equiv.), and EDC (2.65 g, 17.1 mmol, 1.50 equiv.). Purification *via* column chromatography (67:33 *n*-hexane/EA) afforded **8j** (768 mg, 2.41 mmol, 21%) as a yellow oil.

$C_{13}H_{16}NO_2I$ (344.18 $\frac{g}{mol}$)

R_f: 0.84 (*n*-hexane/EA = 80:20) [UV]

^{1}H NMR(400.16 MHz, $CDCl_3$): δ = 7.85 (dd, ^3J = 7.9 Hz, ^4J = 1.2 Hz, 1H), 7.36 (dd, ^3J = 7.8 Hz, ^4J = 1.7 Hz, 1H), 7.29 (m, 1H), 6.91 (m, 1H), 5.85 (dddd, ^3J = 17.1 Hz, ^3J = 10.2 Hz, ^3J = 6.9 Hz, ^3J = 6.9 Hz, 1H), 5.09 (dm, ^3J = 17.1 Hz, 1H), 5.00 (dm, ^3J = 10.2 Hz, 1H), 4.42 (m, 1H), 3.46 (s, 3H), 3.15 (s, 3H), 2.72 (m, 1H), 2.34 (m, 1H).

^{13}C NMR(100.62 MHz, $CDCl_3$): δ = 173.7, 142.7, 139.7, 135.9, 128.9, 128.7, 128.0, 116.9, 101.8, 61.5, 51.9, 37.9, 32.5.

HRMS (ESI-TOF) m/z: $[M+Na]^+$ Calcd for $C_{13}H_{16}NO_2INa$ 368.0118; Found 368.0124.

IR (ATR, ṽ): 1657 cm^{-1} (s, *CO*NMeOMe).

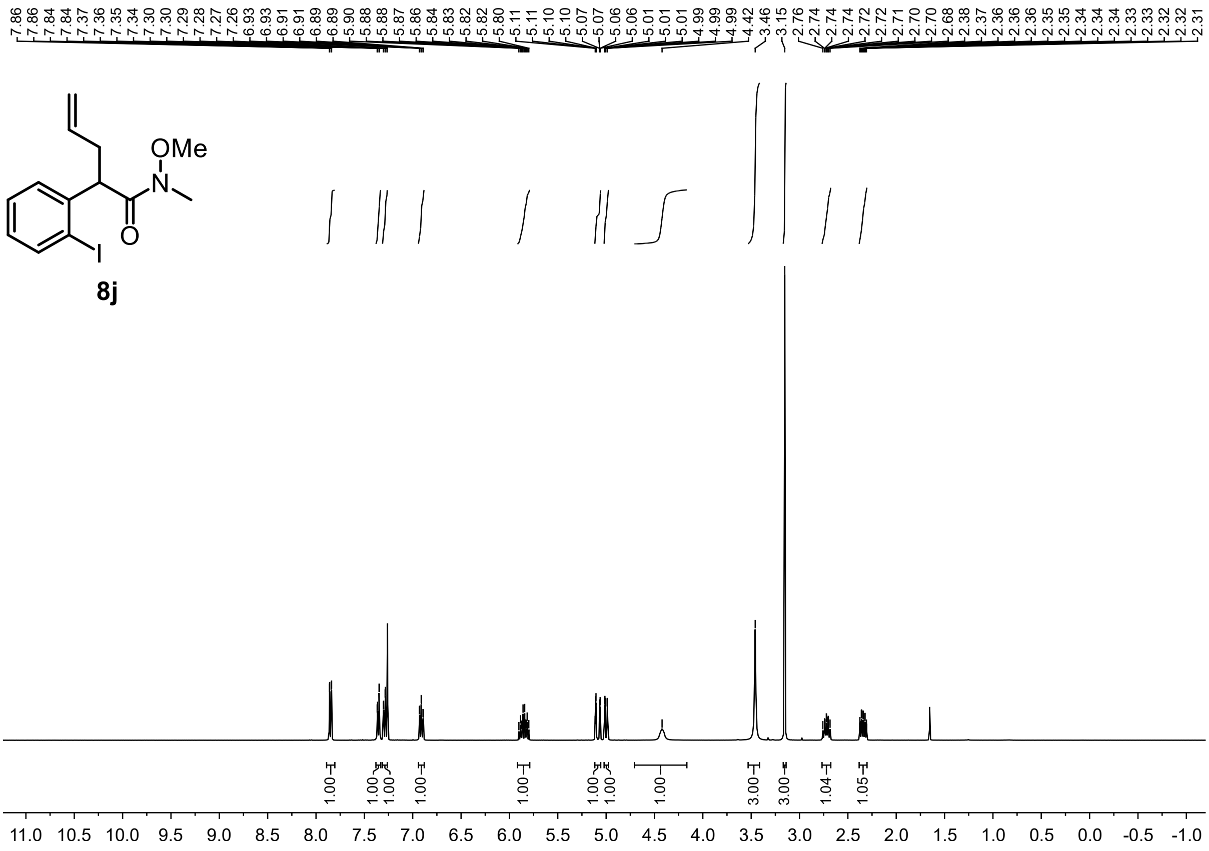

Figure S 11. ^{1}H NMR of **8j** in $CDCl_3$ measured at 400.16 MHz.

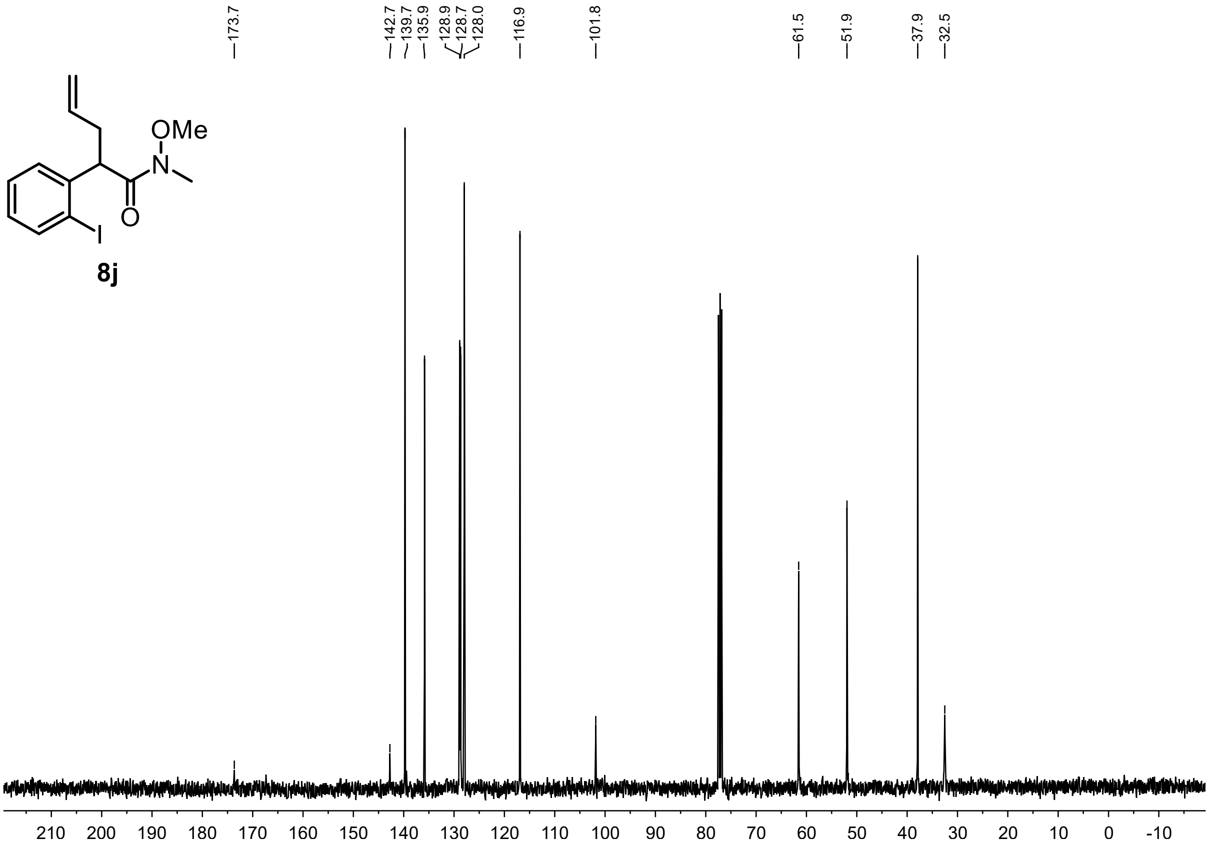

Figure S 12. ^{13}C NMR of **8j** in $CDCl_3$ measured at 100.63 MHz.

1-(2-bromophenyl)-*N*-methoxy-*N*-methylcyclopropane-1-carboxamide

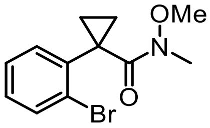

8k

8k was synthesized according to **GP-D** employing 1-(2-bromophenyl)cyclopropanecarboxylic acid (699 mg, 2.90 mmol, 1.00 equiv.), N,O-dimethylhydroxylamine hydrochloride (424 mg, 4.35 mmol, 1.50 equiv.), DMAP (531 mg, 4.35 mmol, 1.50 equiv.), and EDC (675 mg, 4.35 mmol, 1.50 equiv.). Purification *via* column chromatography (90:10 *n*-hexane/EA) afforded **8k** (651 mg, 2.04 mmol, 70%) as a yellow oil. The analytical data is in accordance with literature.

$C_{12}H_{14}NO_2Br$ (284.15 $\frac{g}{mol}$)

mp: 80.0 °C.

R_f: 0.73 (*n*-hexane/EA = 80:20) [UV]

^{1}H NMR(700.21 MHz, $CDCl_3$): δ = 7.54 (dd, ^3J = 7.9 Hz, ^4J = 1.1 Hz, 1H), 7.33 (dd, ^3J = 7.8 Hz, ^3J = 1.6 Hz, 1H), 7.27 (m, 1H), 7.09 (m, 1H), 3.10 (s, 3H), 2.90 (s, 3H), 1.69 (m, 2H), 1.11 (m, 2H).

^{13}C NMR(176.08 MHz, $CDCl_3$): δ = 173.4, 141.5, 132.5, 131.0, 128.0, 127.0, 126.9, 59.8, 33.8, 31.0, 16.1.

HRMS (ESI-TOF) m/z: $[M+Na]^+$ Calcd for $C_{12}H_{14}NO_2BrNa$ 306.0100; Found 306.0100.

IR (ATR, ṽ): 1654 cm^{-1} (s, *CO*NMeOMe).

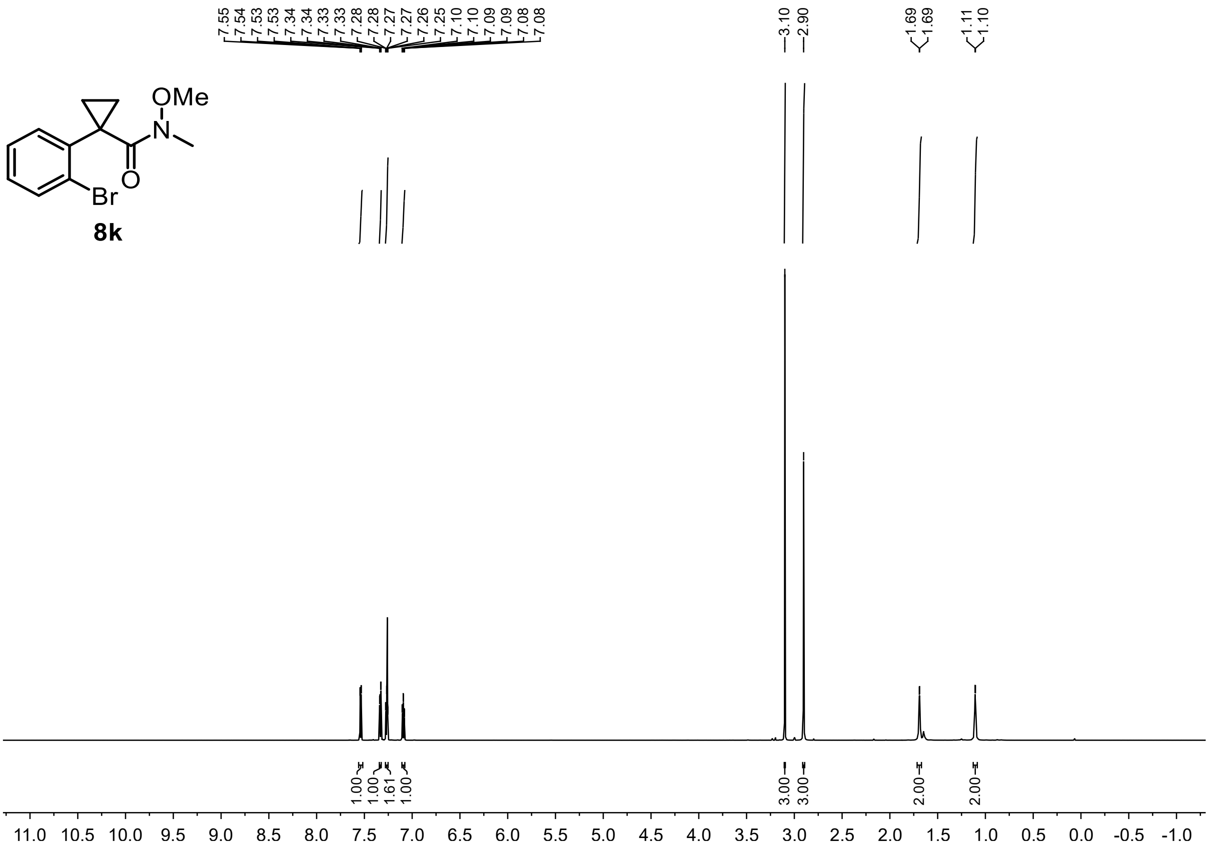

Figure S 13. ^{1}H NMR of **8k** in $CDCl_3$ measured at 700.21 MHz.

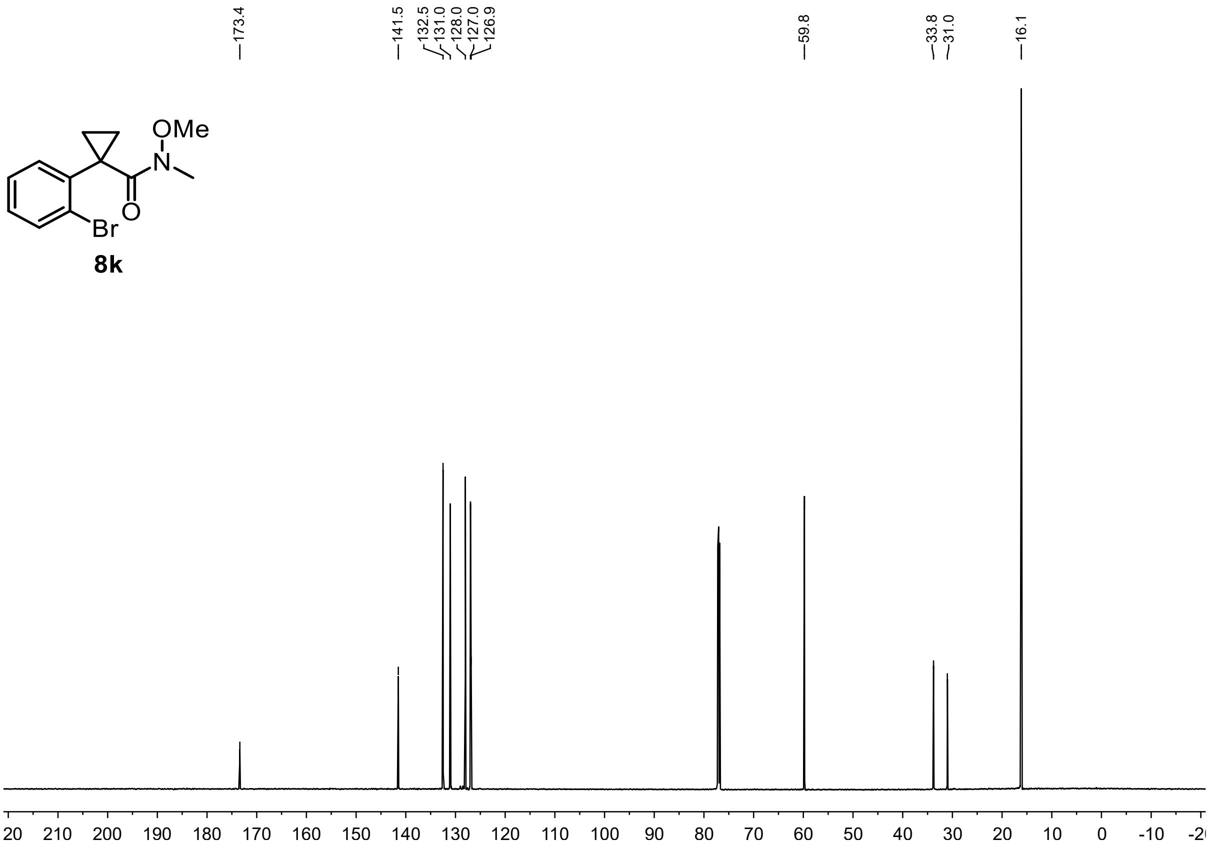

Figure S 14. ^{13}C NMR of **8k** in $CDCl_3$ measured at 176.08 MHz.

2-(2-iodo-4-isobutylphenyl)-*N*-methoxy-*N*-methylpropanamide

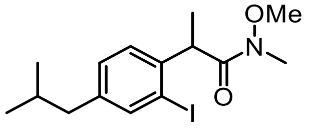

8l

8l was synthesized according to **GP-F** employing ibuprofen (2.06 g, 10.0 mmol, 1.00 equiv.), $Pd(OAc)_2$ (112 mg, 500 µmol, 5 mol%), PIDA (2.42 g, 7.50 mmol, 0.75 equiv.), I_2 (1.90 g, 7.50 mmol, 0.75 equiv.), N,O-dimethylhydroxylamine hydrochloride (1.46 g, 15.0 mmol, 1.50 equiv.), DMAP (1.83 g, 15.0 mmol, 1.50 equiv.), and EDC (2.33 g, 15.0 mmol, 1.50 equiv.). Purification *via* column chromatography (80:20 *n*-hexane/EA) afforded 1.51 g of a mixture of **8l** (1.22 g, 3.30 mmol) and N-methoxy-N-methyl-2-(4-isobutylphenyl)propenamide (78:22) as a yellow oil corresponding to 33% yield over two steps.

$C_{15}H_{22}NO_2I$ (375.25 $\frac{g}{mol}$)

R_f: 0.88 (*n*-hexane/EA = 80:20) [UV]

^{1}H NMR(700.21 MHz, $CDCl_3$): δ = 7.63 (d, ^4J = 1.8 Hz, 1H), 7.19 (d, ^3J = 7.8 Hz, 1H), 7.06 (dd, ^3J = 7.8 Hz, ^4J = 1.8 Hz, 1H), 4.36 (m, 1H), 3.36 (s, 3H), 3.15 (s, 3H), 2.37 (d, ^3J = 7.2 Hz, 2H), 1.82 (m, 1H), 1.33 (d, ^3J = 7.0 Hz, 3H), 0.88 (d, ^3J = 6.6 Hz, 3H), 0.88 (d, ^3J = 6.6 Hz, 3H).

^{13}C NMR(176.08 MHz, $CDCl_3$): δ = 175.4, 142.3, 142.1, 140.0, 129.8, 126.9, 100.8, 61.2, 46.7, 44.4, 32.6, 30.2, 22.4, 22.4, 18.6.

HRMS (APCI-TOF) m/z: $[M+Na]^+$ Calcd for $C_{15}H_{22}NO_2INa$ 398.0588; Found 398.0589.

IR (ATR, ṽ): 1649 cm^{-1} (s, *CO*NMeOMe).

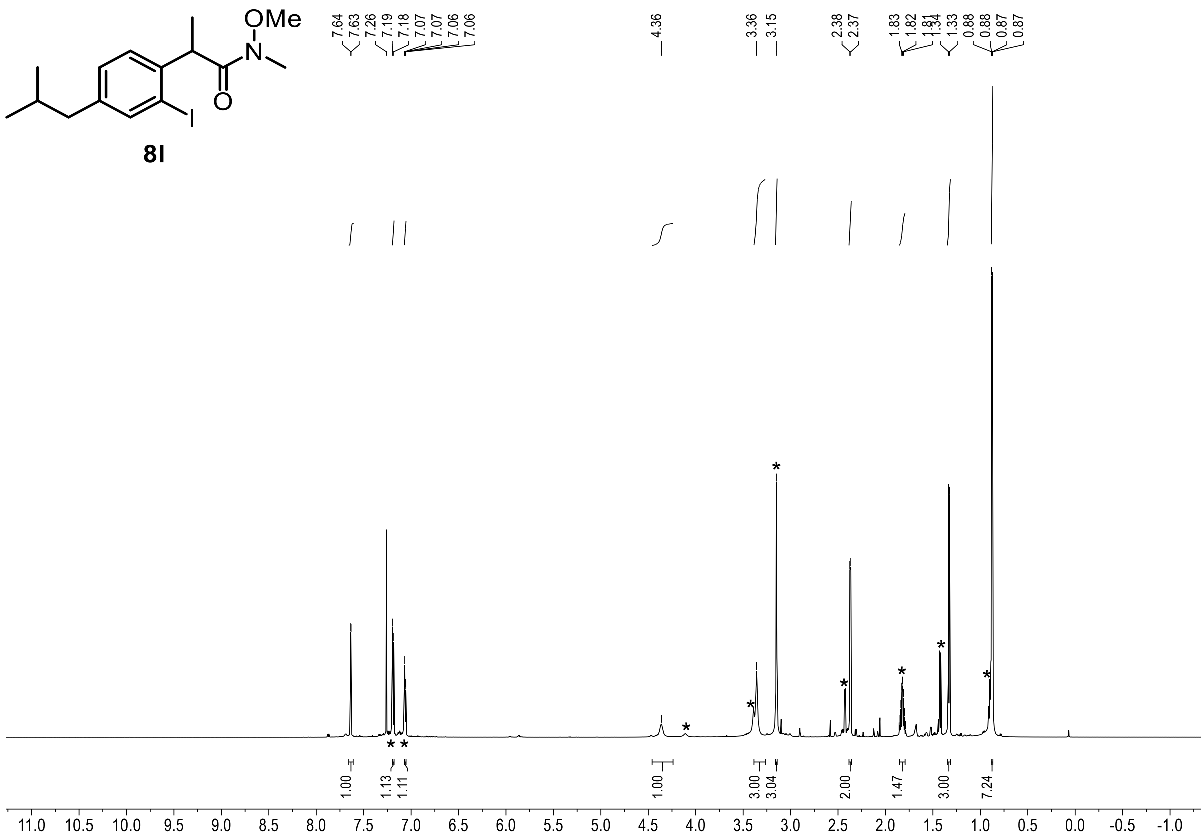

Figure S 15. ^{1}H NMR of **8l** in $CDCl_3$ measured at 700.21 MHz. Signals denoted by * correspond to N-Methoxy-N-methyl-2-(4-isobutylphenyl)propanamide.[12]

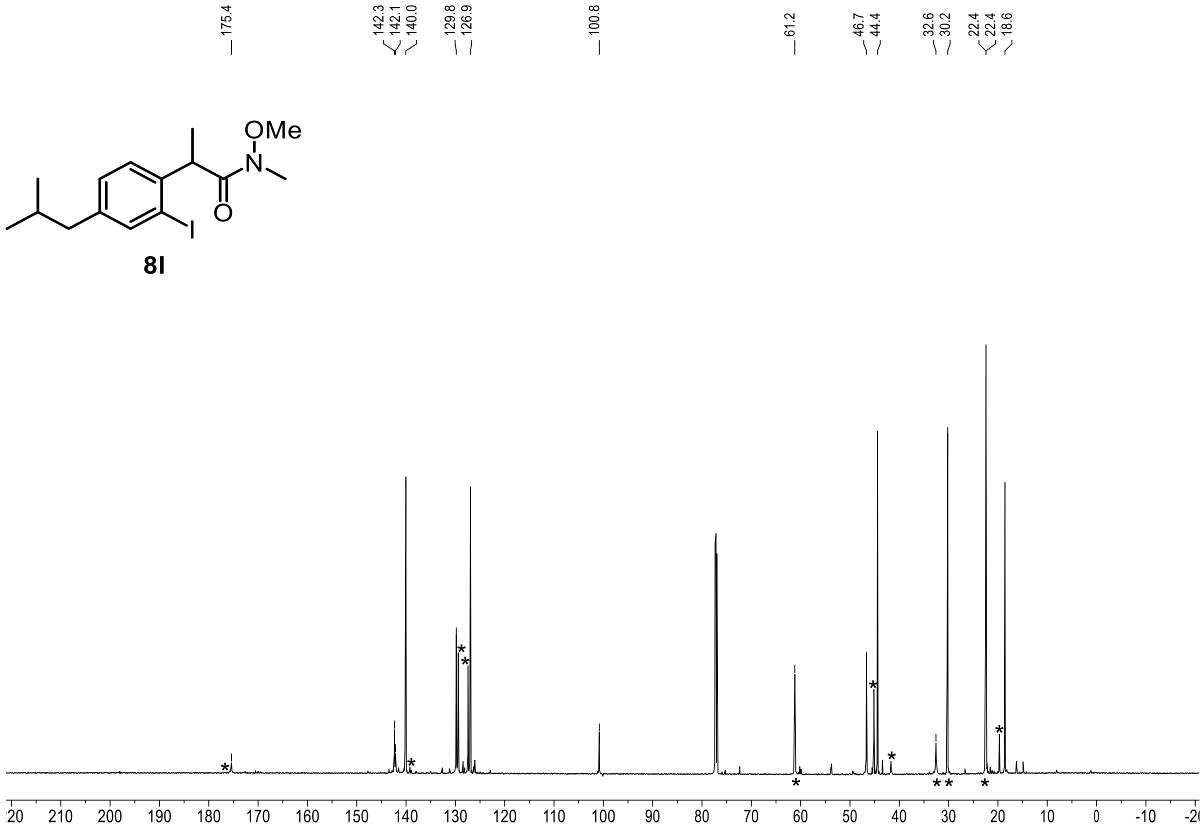

Figure S 16. ^{13}C NMR of **8l** in $CDCl_3$ measured at 176.08 MHz. Signals denoted by * correspond N-Methoxy-N-methyl-2-(4-isobutylphenyl)propanamide.[12]

4.2 Kobayashi and ketene precursors

1,1-diethoxyethene

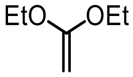

9

9 was synthesized according to literature.[13] In a dried two necked Schlenk RBF (500 mL) KO^{t}Bu (93.0 g, 829 mmol, 1.00 equiv.) was cooled to 0 °C and 2-bromo-1,1-diethoxyethane (129 mL, 829 mmol, 1.00 equiv.) was added dropwise until white mist formed (about 10 mL). The remaining 2-bromo-1,1-diethoxyethane was added dropwise after disappearance of the mist, whilst stirring manually. ^{t}BuOH (50 mL) was added and the mixture was heated to 50 °C and stirred for 1 h. Subsequently, ^{t}BuOH was distilled off (oil bath: 130 – 145 °C, 1.0 atm), after which **9** was obtained *via* vacuum distillation (oil bath: 60 – 120 °C, 30 – 10 mbar) as a colorless liquid (84.8 g, 730 mmol, 88%). The analytical data is in accordance with literature.[13]

$C_6H_{12}O_2$ (116.16 $\frac{g}{mol}$)

b.p. (28 mbar): 35 °C.

^{1}H NMR(400.16 MHz, $CDCl_3$): δ = 3.81 (q, ^3J = 7.1 Hz, 4H), 3.07 (d, ^2J = 4.2 Hz, 2H), 1.31 (t, ^3J = 7.1 Hz, 6H).

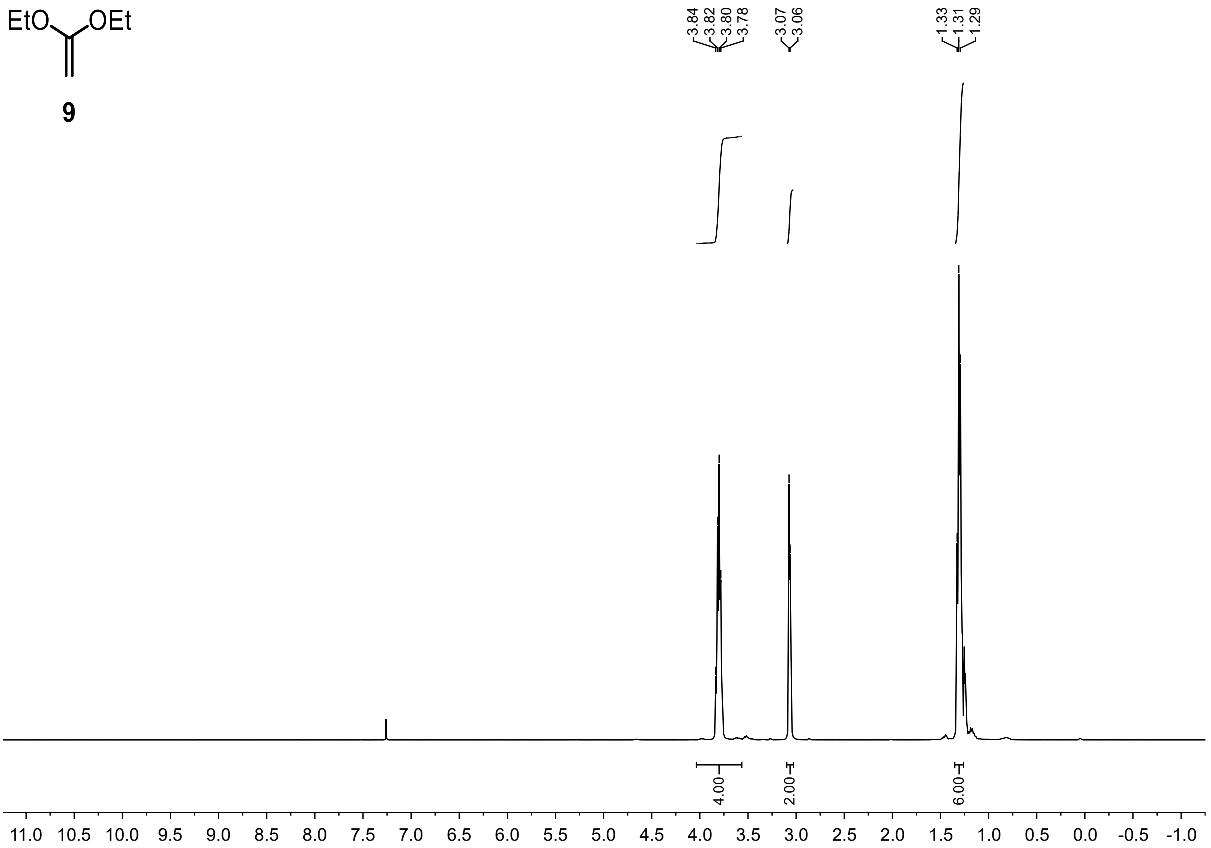

Figure S 17. ^{1}H NMR of **9** in $CDCl_3$ measured at 400.16 MHz.

3-(trimethylsilyl)naphthalen-2-yl trifluoromethanesulfonate

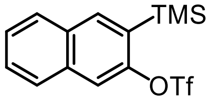

10b

10b was synthesized according to **GP-G** employing 3-bromo-2-naphthol (5.23 g, 23.5 mmol, 1.00 equiv.), HMDS (5.13 mL, 24.6 mmol, 1.05 equiv.), *n*-BuLi (14.1 mL, 2.50 M, 1.50 equiv.) and Tf_2O (4.72 mL, 28.1 mmol, 1.20 equiv.). Purification *via* column chromatography (98:02 *n*-hexane/EA) afforded **10b** (4.20 g, 12.1 mmol, 52%) as a colorless oil. The analytical data is in accordance with literature.[5]

$C_{14}H_{15}O_3F_3SSi$ (348.41 $\frac{g}{mol}$)

R$_f$: 0.56 (*n*-hexane/EA = 98:2) [UV]

^{1}H NMR(700.21 MHz, $CDCl_3$): δ = 8.02 (s, 1H), 7.88 (d, ^3J = 7.8 Hz, 1H), 7.84 (d, ^3J = 7.7 Hz, 1H), 7.82 (s, 1H), 7.56 (m, 2H), 0.46 (m, 9H).

^{13}C NMR(176.08 MHz, $CDCl_3$): δ = 152.7, 137.7, 134.2, 131.9, 131.2, 128.1, 127.9, 127.1, 118.7 (q, ^1J = 320.2 Hz), 116.6, -0.6.

^{19}F NMR(658.79 MHz, $CDCl_3$): δ =-73.7.

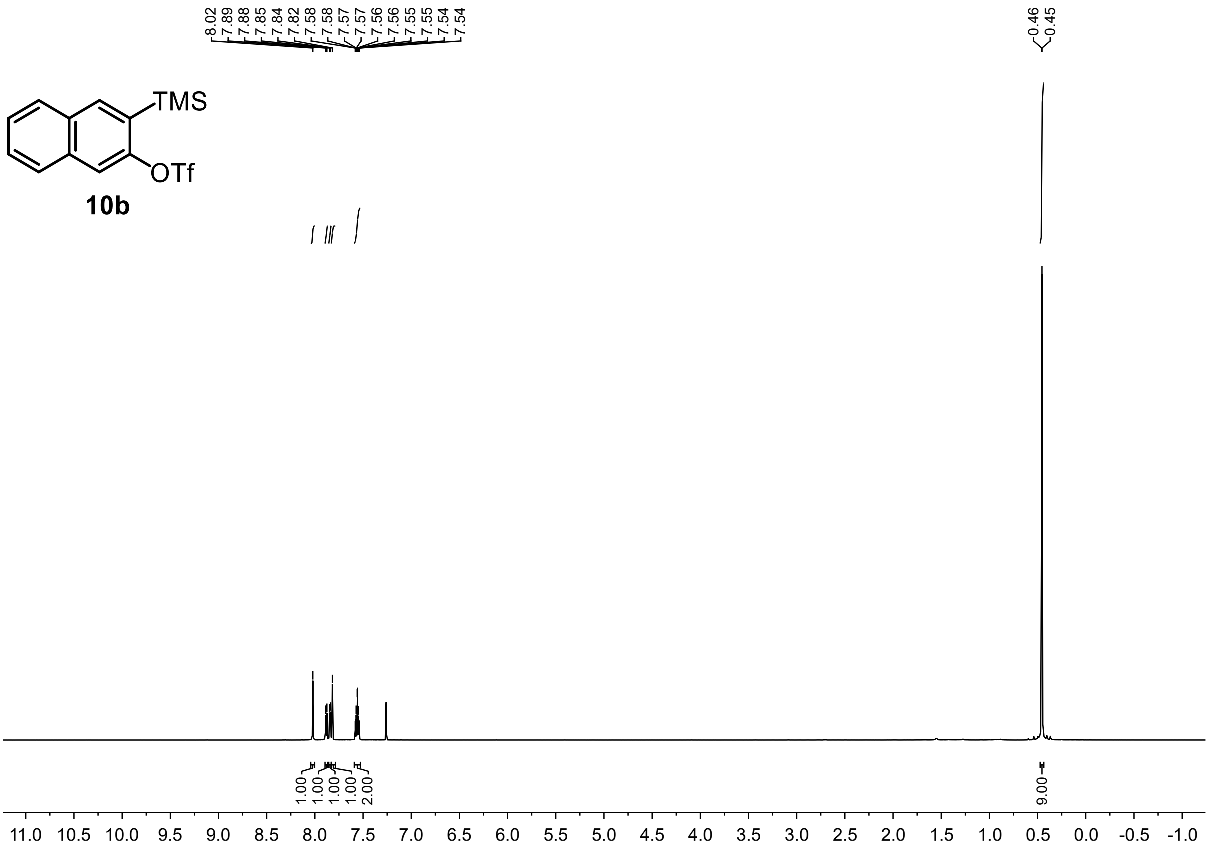

Figure S 18. ^{1}H NMR of **10b** in $CDCl_3$ measured at 700.21 MHz.

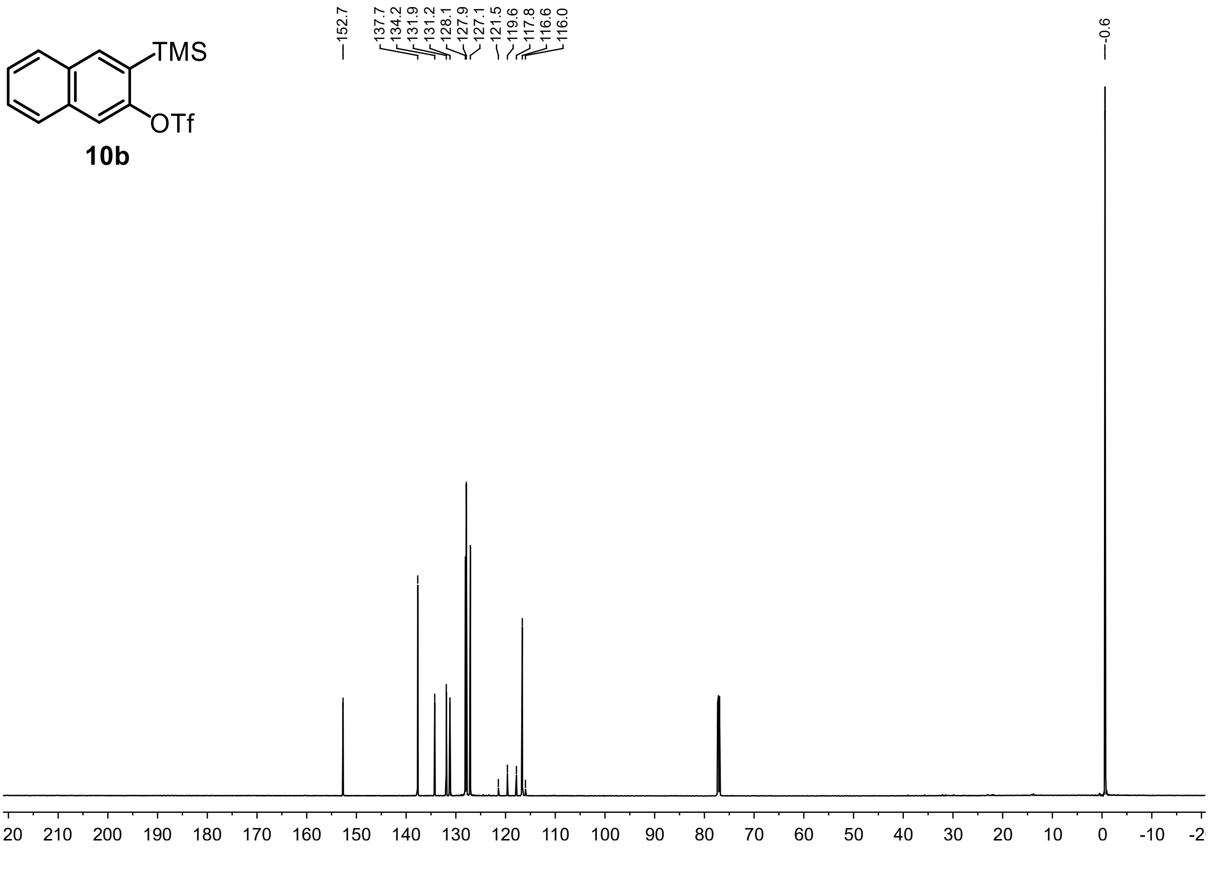

Figure S 19. ^{13}C NMR of **10b** in $CDCl_3$ measured at 176.08 MHz.

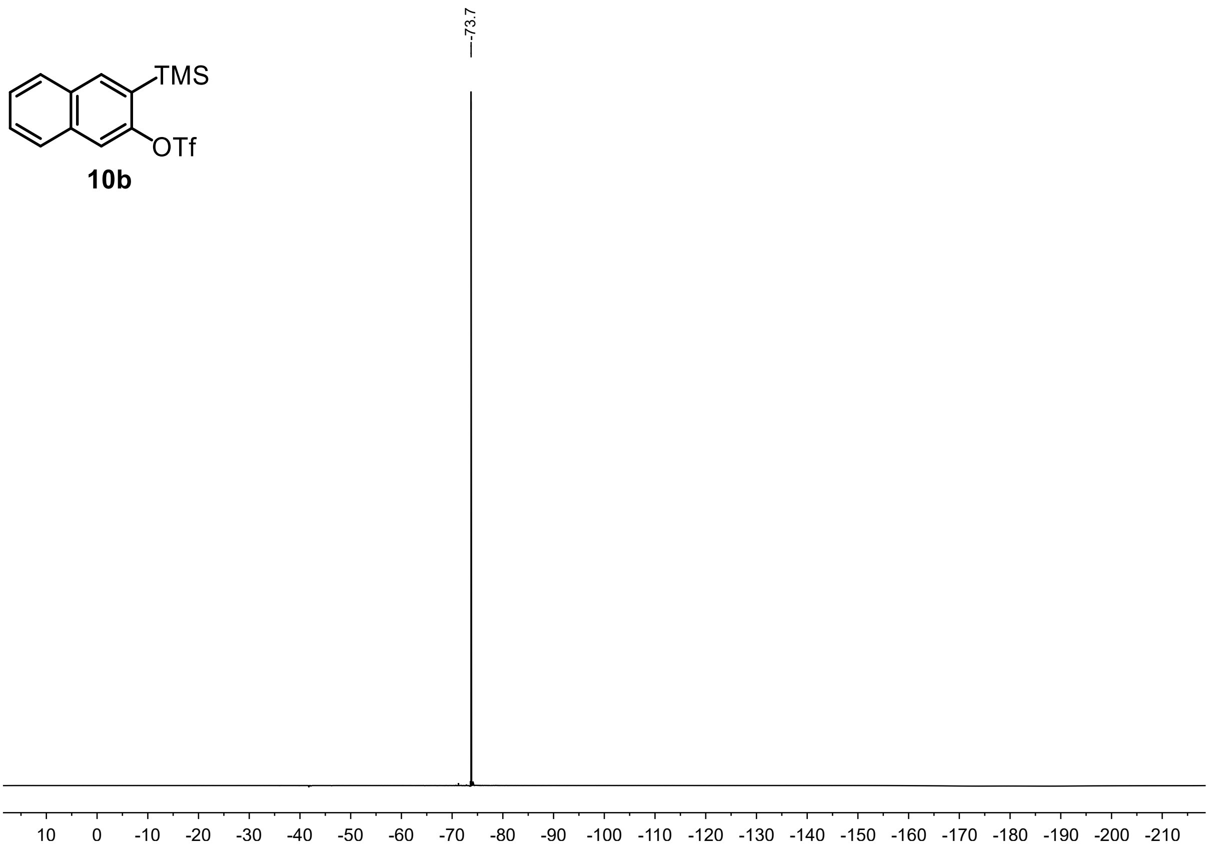

Figure S 20. ^{19}F NMR of **10b** in $CDCl_3$ measured at 658.79 MHz.

3-methyl-2-(trimethylsilyl)phenyl trifluoromethanesulfonate

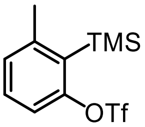

10m

10m was synthesized according to **GP-G** employing 2-bromo-3-methylphenol (5.15 g, 27.5 mmol, 1.00 equiv.), HMDS (6.03 mL, 28.9 mmol, 1.05 equiv.), *n*-BuLi (25.8 mL, 1.60 M, 1.50 equiv.) and Tf_2O (5.55 mL, 33.0 mmol, 1.20 equiv.). Purification *via* column chromatography (98:02 *n*-hexane/EA) afforded **10m** (3.26 g, 10.4 mmol, 38%) as a colorless oil. The analytical data is in accordance with literature.[14]

$C_{11}H_{15}O_3F_3SSi$ (312.38 $\frac{g}{mol}$)

R_f: 0.71 (*n*-hexane/EA = 98:2) [UV]

^{1}H NMR(700.21 MHz, $CDCl_3$): δ = 7.30 (m, 1H), 7.15 (m, 2H), 2.50 (s, 3H), 0.44 (s, 9H).

^{13}C NMR(176.08 MHz, $CDCl_3$): δ = 155.4, 146.9, 131.2, 130.6, 130.2, 118.7 (q, ^1J = 320.4 Hz), 117.6, 24.3, 2.0.

^{19}F NMR(658.79 MHz, $CDCl_3$): δ =-73.1.

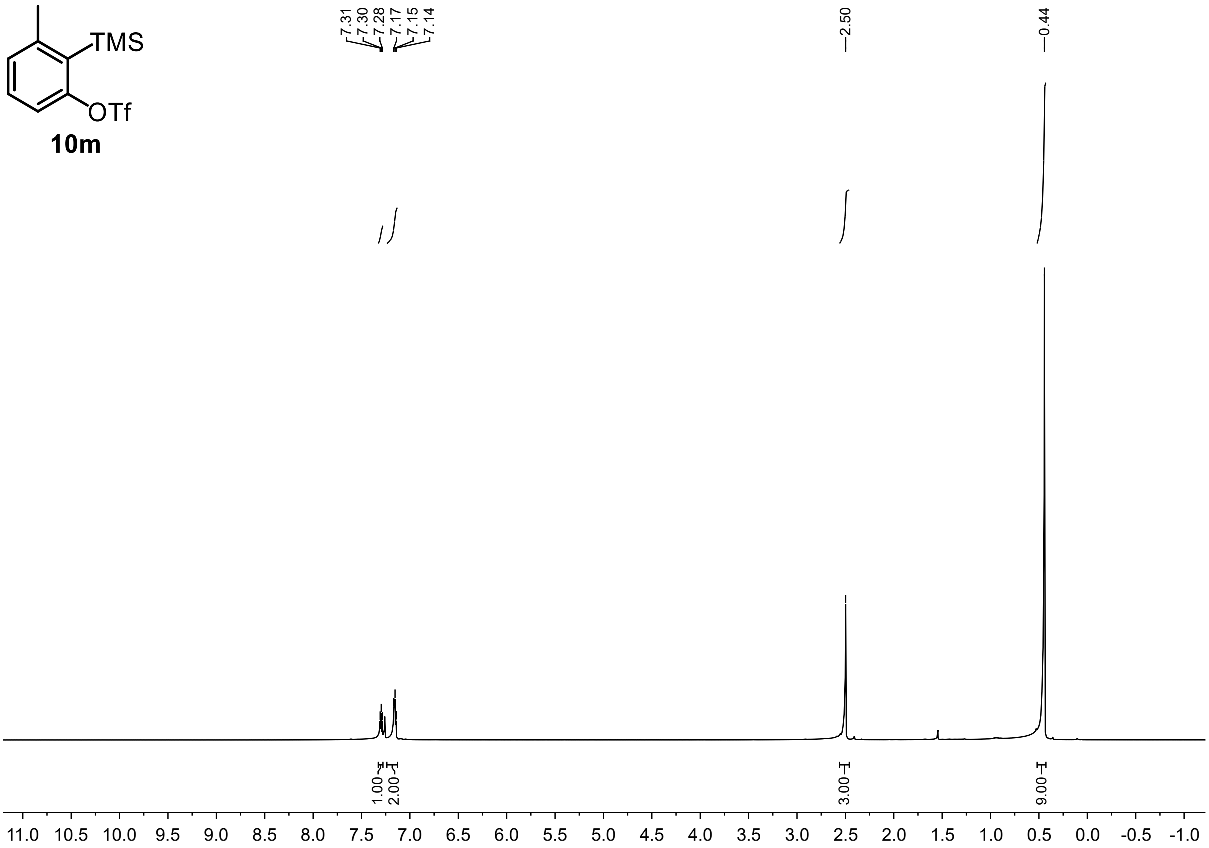

Figure S 21. ^{1}H NMR of **10m** in $CDCl_3$ measured at 700.21 MHz.

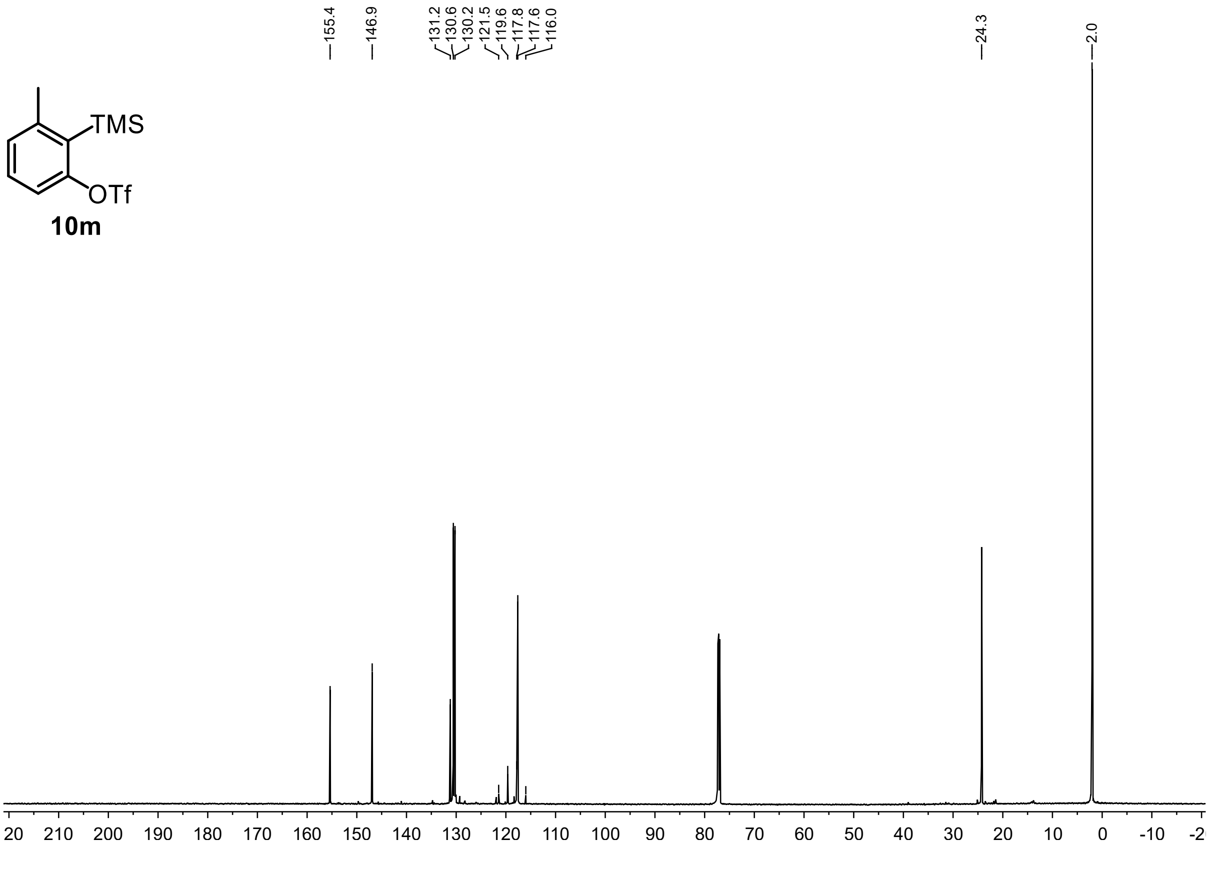

Figure S 22. ^{13}C NMR of **10m** in $CDCl_3$ measured at 176.08 MHz.

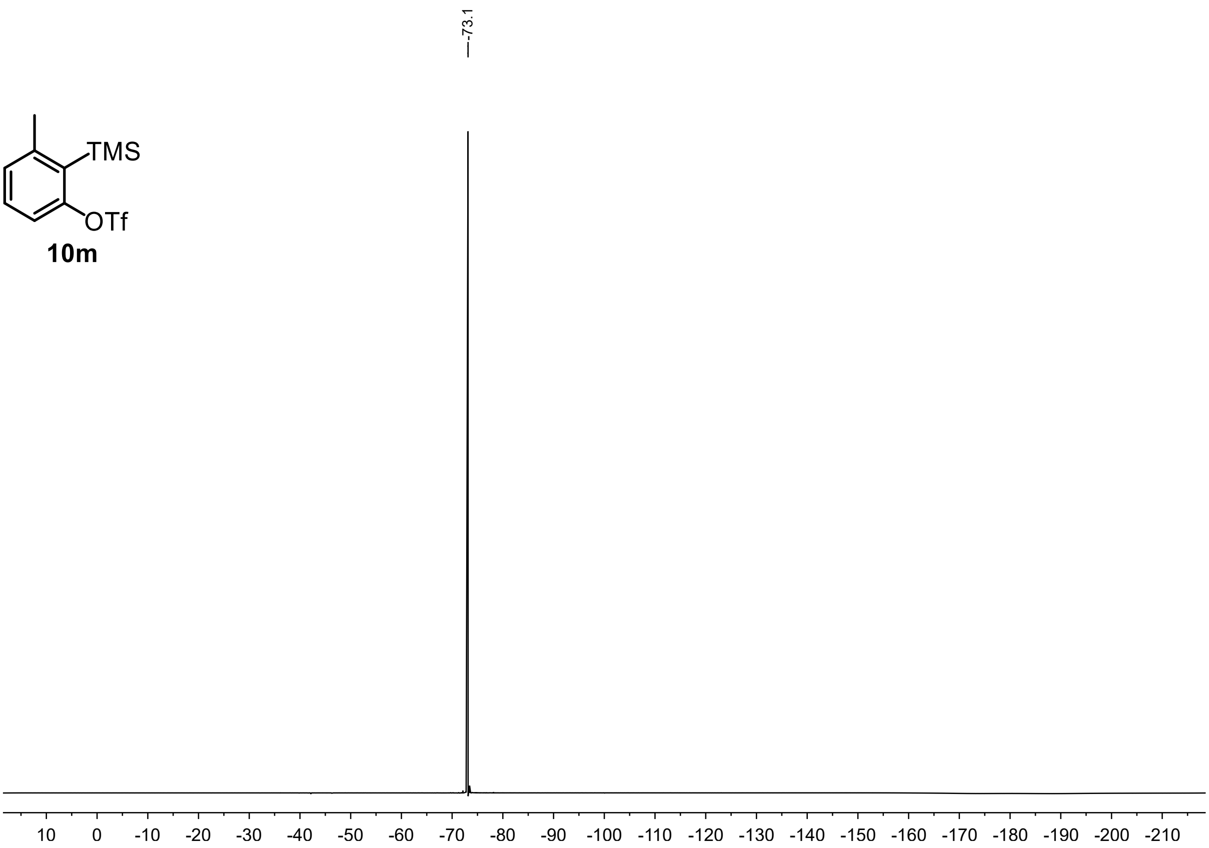

10m

Figure S 23. ^{19}F NMR of **10m** in $CDCl_3$ measured at 658.79 MHz.

4.3 Benzocyclobutanones

bicyclo[4.2.0]octa-1,3,5-trien-7-one

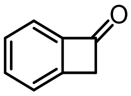

1a

1a was synthesized according to **GP-H** employing 2-(2-iodophenyl)acetic acid (22.1 g, 72.5 mmol, 1.00 equiv.) and ^{t}BuLi (89.6 mL, 1.70 M, 2.10 equiv.). Purification *via* flash chromatography (80 g SiO_2, gradient from 100:0 to 93:07 *n*-hexane/EA over 15 CV) afforded **1a** (2.74 g, 23.2 mmol, 32%) as a pale yellow oil. Subsequent vacuum distillation at 2.8 mbar, 54 °C (70 °C water bath) furnished **1a** (2.32 g, 19.6 mmol, 27%) as a colorless oil. The analytical data is in accordance with literature.[15]

C_8H_6O (118.14 $\frac{g}{mol}$)

b.p. (2.8 mbar): 54 °C.

R$_f$: 0.66 (*n*-hexane/EA = 80:20) [UV]

^{1}H NMR(700.21 MHz, $CDCl_3$): δ = 7.53 (m, 2H), 7.40 (m, 1H), 7.34 (d, ^3J = 7.6 Hz, 1H), 3.98 (s, 2H).

^{13}C NMR(176.08 MHz, $CDCl_3$): δ = 188.9, 151.4, 148.1, 135.3, 128.8, 123.8, 120.7, 52.5.

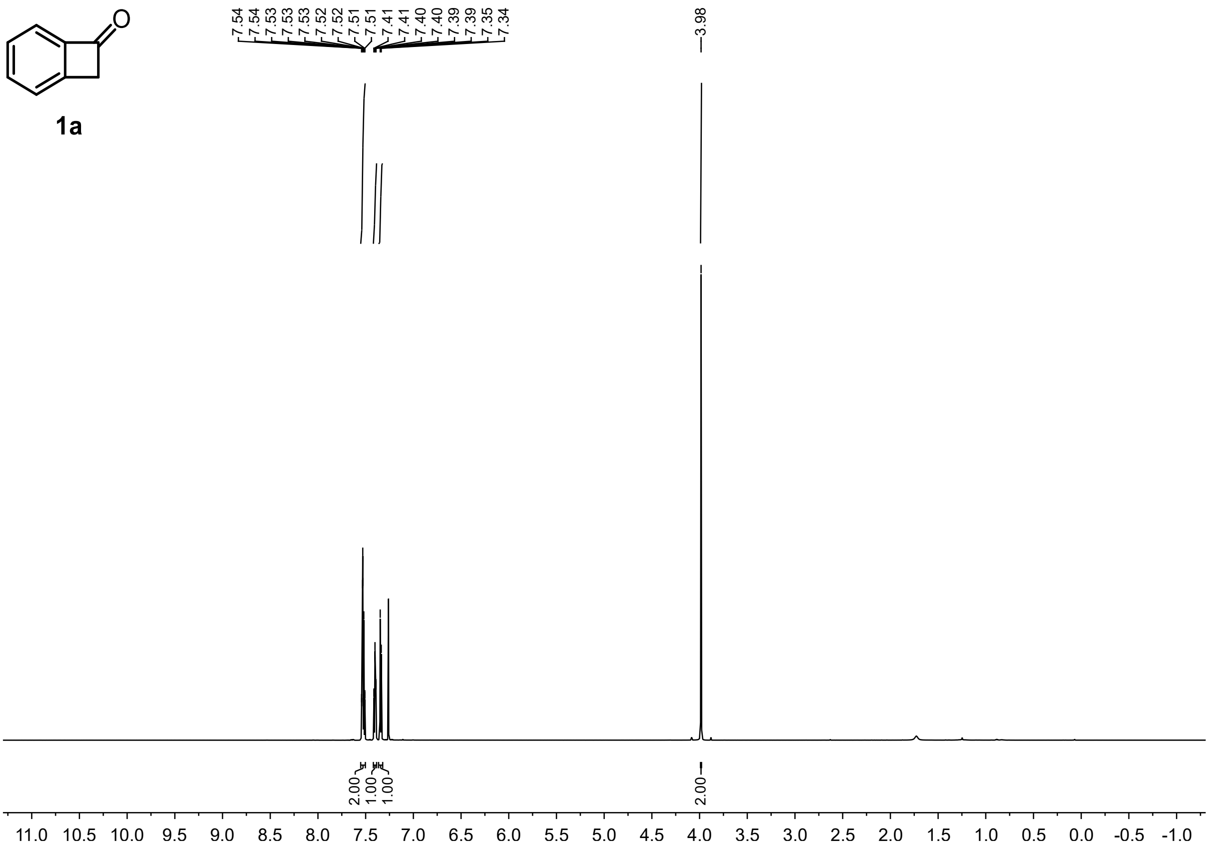

Figure S 24. ^{1}H NMR of **1a** in $CDCl_3$ measured at 700.21 MHz.

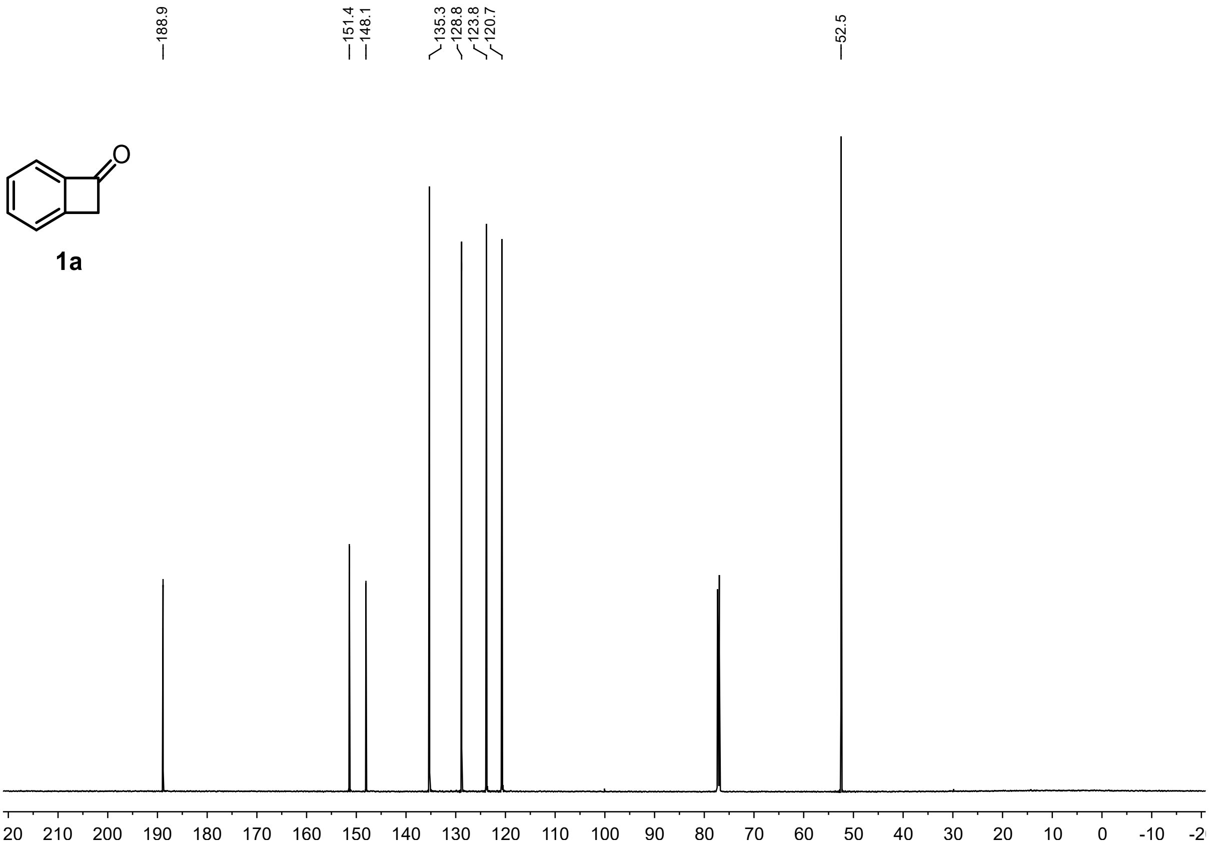

Figure S 25. ^{13}C NMR of **1a** in $CDCl_3$ measured at 176.08 MHz.

cyclobuta[b]naphthalen-1(2H)-one

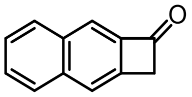

1b

1b was synthesized according to **GP-J** employing **10b** (4.01 g, 11.5 mmol, 1.0 equiv.), **9** (3.41 mL, 23.0 mmol, 2.00 equiv.), CsF (2.62 g, 17.3 mmol, 1.50 equiv.), and TFA (1.76 mL, 23.0 mmol, 2.00 equiv.). Purification *via* column chromatography (gradient from 100:0 to 85:15 *n*-hexane/EA) and recrystallisation from *n*-hexane/EA (80:20) afforded **1b** (630 mg, 3.74 mmol, 33%) as colorless crystals. The analytical data is in accordance with literature.[5]

$C_{12}H_8O$ (168.20 $\frac{g}{mol}$)

R$_f$: 0.50 (*n*-hexane/EA = 90:10) [UV]

^{1}H NMR(400.16 MHz, $CDCl_3$): δ = 7.95 (d, ^3J = 8.5 Hz, 1H), 7.87 (m, 3H), 7.60 (ddd, ^3J = 8.2 Hz, ^3J = 6.9 Hz, ^4J = 1.3 Hz, 1H), 7.50 (m, 1H), 4.19 (s, 2H).

^{13}C NMR(100.62 MHz, $CDCl_3$): δ = 190.7, 146.5, 143.5, 137.8, 133.6, 130.9, 128.6, 128.4, 126.2, 121.6, 120.2, 53.1.

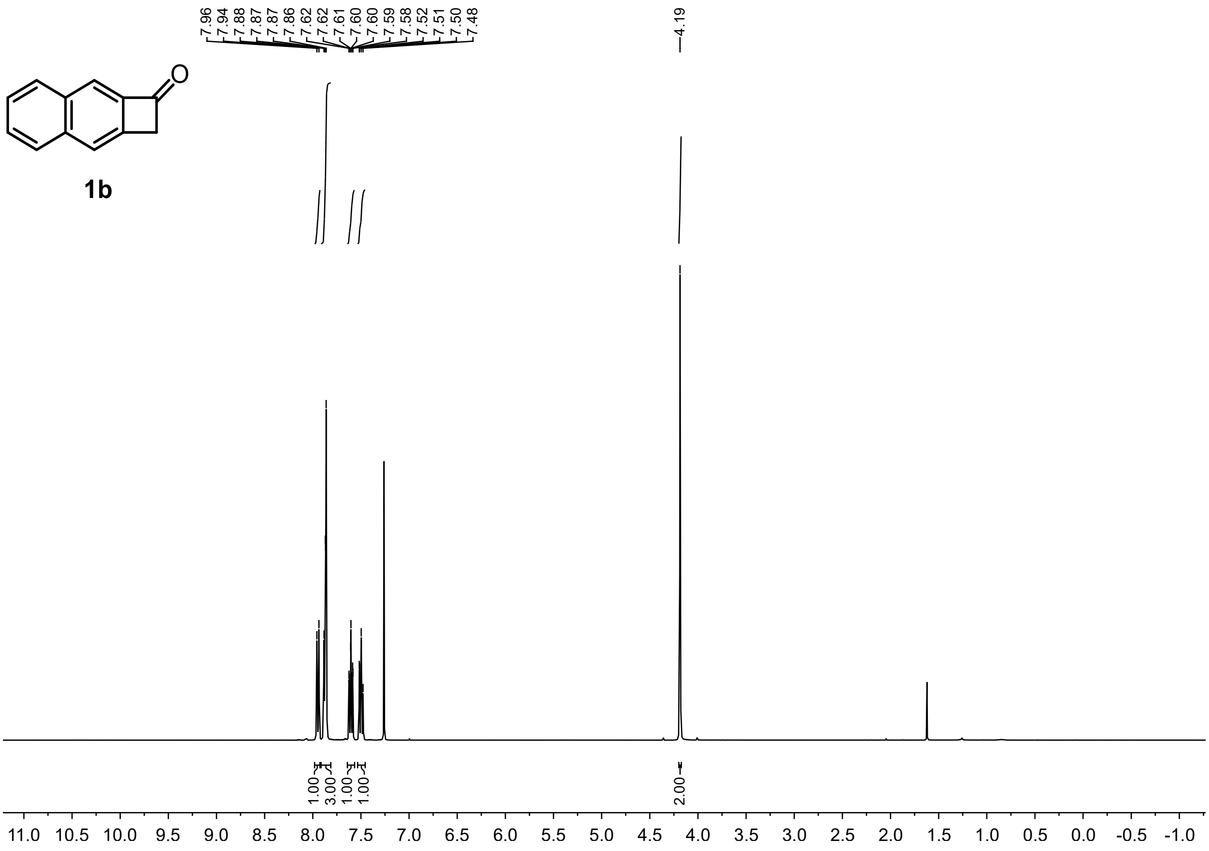

Figure S 26. ^{1}H NMR of **1b** in $CDCl_3$ measured at 400.16 MHz.

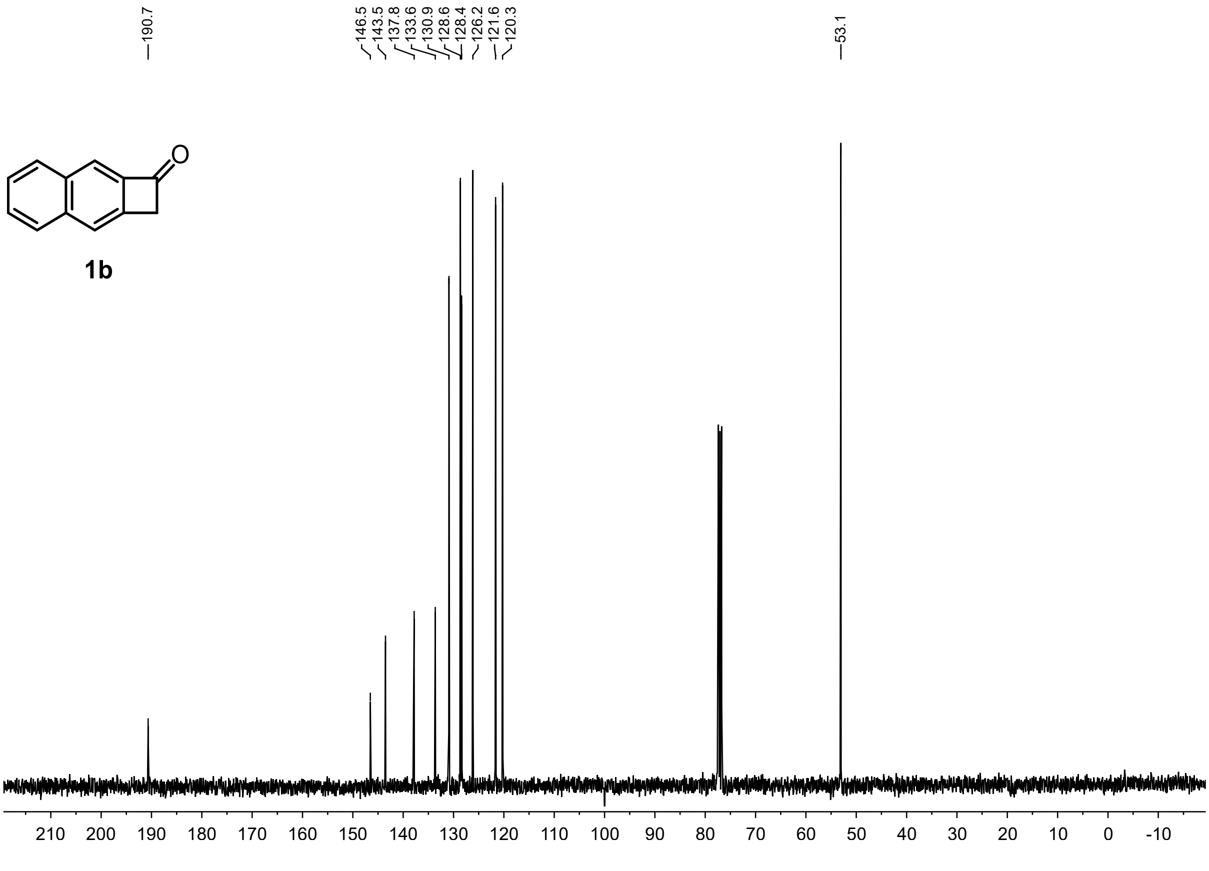

Figure S 27. ^{13}C NMR of **1b** in $CDCl_3$ measured at 100.63 MHz.

2-methylbicyclo[4.2.0]octa-1,3,5-trien-7-one

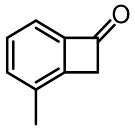

1c

1c was synthesized according to **GP-H** employing **8c** (851 mg, 2.67 mmol, 1.00 equiv.) and ^{t}BuLi (3.14 mL, 1.70 M, 2.00 equiv.). Purification *via* flash chromatography (23 g SiO_2, gradient from 100:0 to 80:20 *n*-hexane/EA over 15 CV) afforded **1c** (281 mg, 2.13 mmol, 80%) as a colorless soid. The analytical data is in accordance with literature**.** The analytical data is in accordance with literature.[6]

C_9H_8O (132.16 $\frac{g}{mol}$)

R$_f$: 0.68 (*n*-hexane/EA = 80:20) [UV]

^{1}H NMR(700.21 MHz, $CDCl_3$): δ = 7.31 (m, 2H), 7.16 (m, 1H), 3.91 (s, 2H), 2.37 (s, 3H).

^{13}C NMR(176.08 MHz, $CDCl_3$): δ = 188.7, 150.5, 147.6, 136.1, 134.2, 129.6, 118.0, 51.2, 17.8.

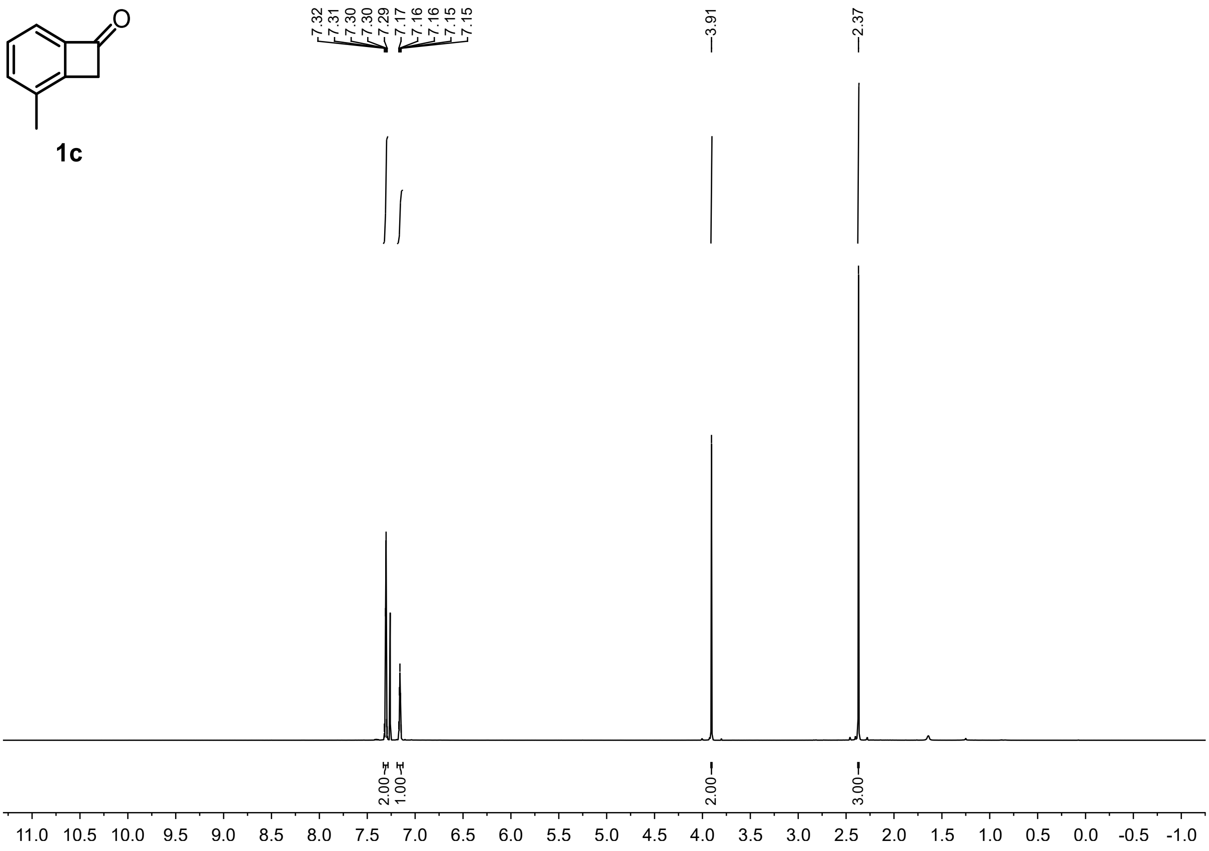

Figure S 28. ^{1}H NMR of **1c** in $CDCl_3$ measured at 700.21 MHz.

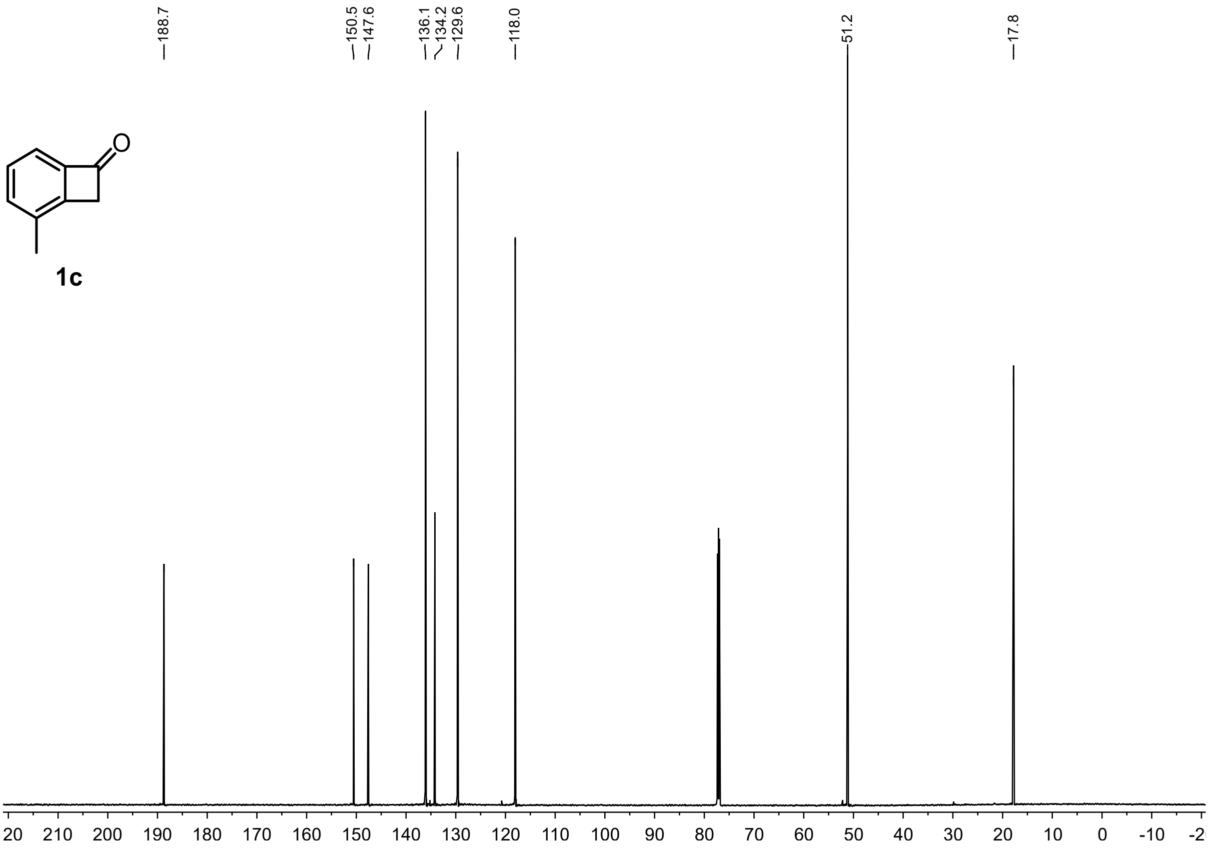

Figure S 29. ^{13}C NMR of **1c** in $CDCl_3$ measured at 176.08 MHz.

4-methoxybicyclo[4.2.0]octa-1,3,5-trien-7-one

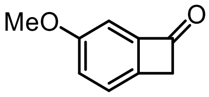

1d

1d was synthesized according to **GP-I** employing 4-bromo anisole (3.74 g, 20.0 mmol, 1.00 equiv.), $NaNH_2$ (3.12 g, 80.0 mmol. 4.00 equiv.), and **9** (10.6 mL, 80.0 mmol, 4.00 equiv.). Purification *via* flash chromatography (40 g SiO_2, gradient from 100:0 to 92:08 *n*-hexane/EA over 11 CV) afforded **1d** (0.633 g, 4.27 mmol, 21%) as a yellow solid. The analytical data is in accordance with literature.[6]

$C_9H_8O_2$ (148.16 $\frac{g}{mol}$)

R$_f$: 0.59 (*n*-hexane/EA = 80:20) [UV]

^{1}H NMR(400.16 MHz, $CDCl_3$): δ = 7.41 (dm, ^3J = 8.1 Hz, 1H), 7.09 (dd, ^3J = 8.1 Hz, ^4J = 2.2 Hz, 1H), 6.82 (d, ^4J = 2.2 Hz, 1H), 3.87 (s, 2H), 3.79 (s, 3H).

^{13}C NMR(100.62 MHz, $CDCl_3$): δ = 188.2, 160.8, 148.8, 144.0, 124.7, 124.1, 103.1, 55.8, 51.1.

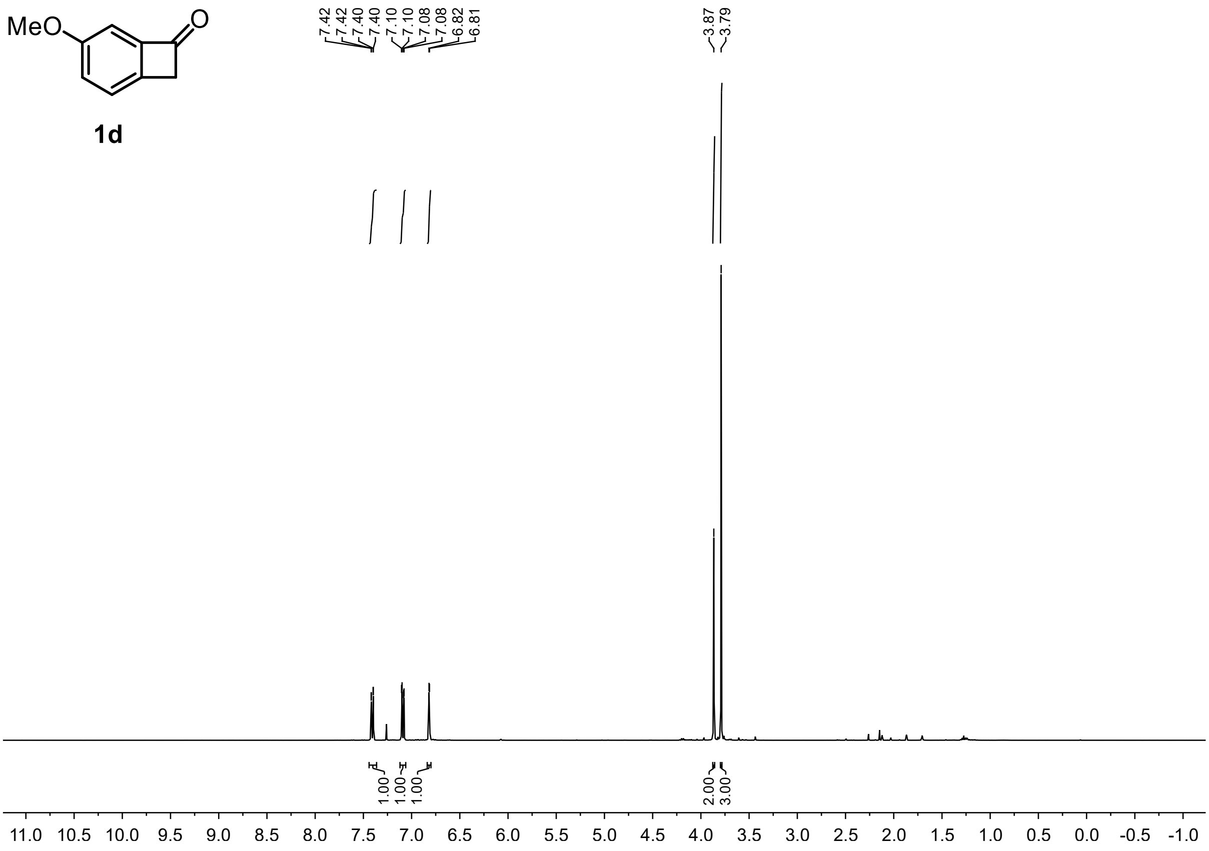

Figure S 30. ^{1}H NMR of **1d** in $CDCl_3$ measured at 400.16 MHz.

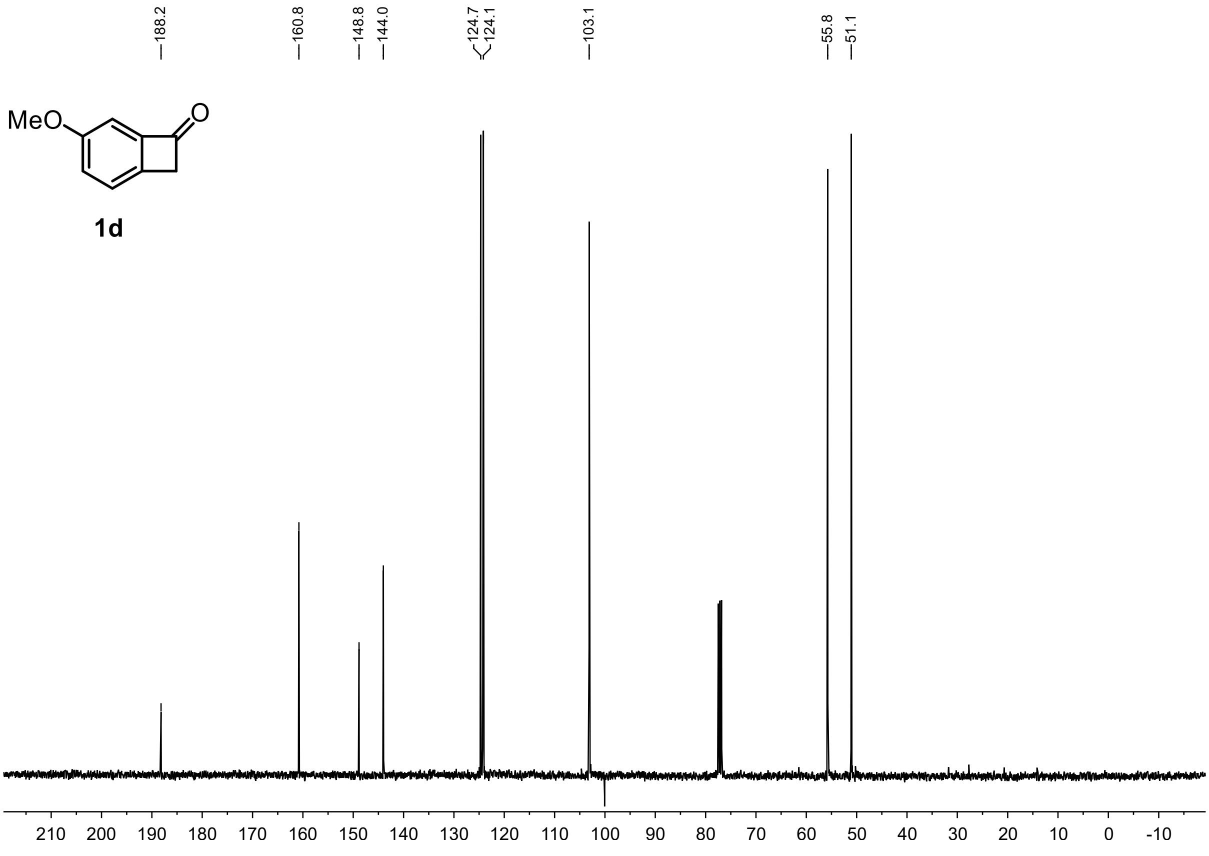

Figure S 31. ^{13}C NMR of **1d** in $CDCl_3$ measured at 100.63 MHz.

cyclobuta[3,4]benzo[1,2-d][1,3]dioxol-7(6H)-one

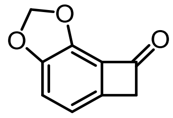

1e

1e was synthesized according to **GP-I** employing 5-bromo 1,3-benzodioxole (4.02 g, 20.0 mmol, 1.00 equiv.), $NaNH_2$ (3.12 g, 80.0 mmol. 4.00 equiv.), and **9** (11.9 mL, 80.0 mmol, 4.00 equiv.). Purification *via* flash chromatography (40 g SiO_2, gradient from 100:0 to 90:10 *n*-hexane/EA over 10 CV) afforded **1e** (1.72 g, 10.61 mmol, 53%) as a pale yellow solid. The analytical data is in accordance with literature.[7]

$C_9H_6O_3$ (162.14 $\frac{g}{mol}$)

R$_f$: 0.52 (*n*-hexane/EA = 80:20) [UV]

^{1}H NMR(400.16 MHz, $CDCl_3$): δ = 6.97 (m, 1H), 6.92 (m, 1H), 6.06 (s, 2H), 3.90 (d, ^4J = 0.8 Hz, 2H).

^{13}C NMR(100.62 MHz, $CDCl_3$): δ = 184.4, 149.1, 141.6, 137.7, 128.2, 115.6, 115.0, 102.6, 52.2.

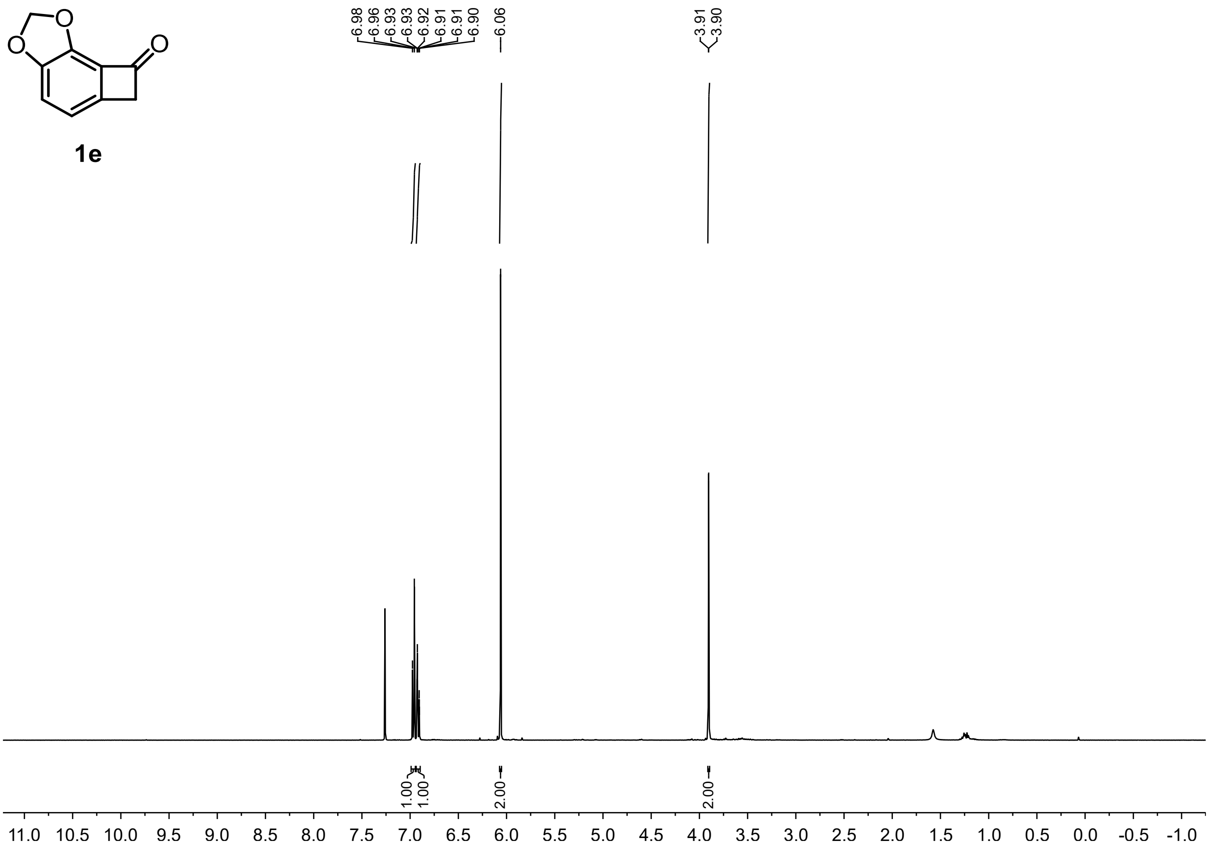

Figure S 32. ^{1}H NMR of **1e** in $CDCl_3$ measured at 400.16 MHz.

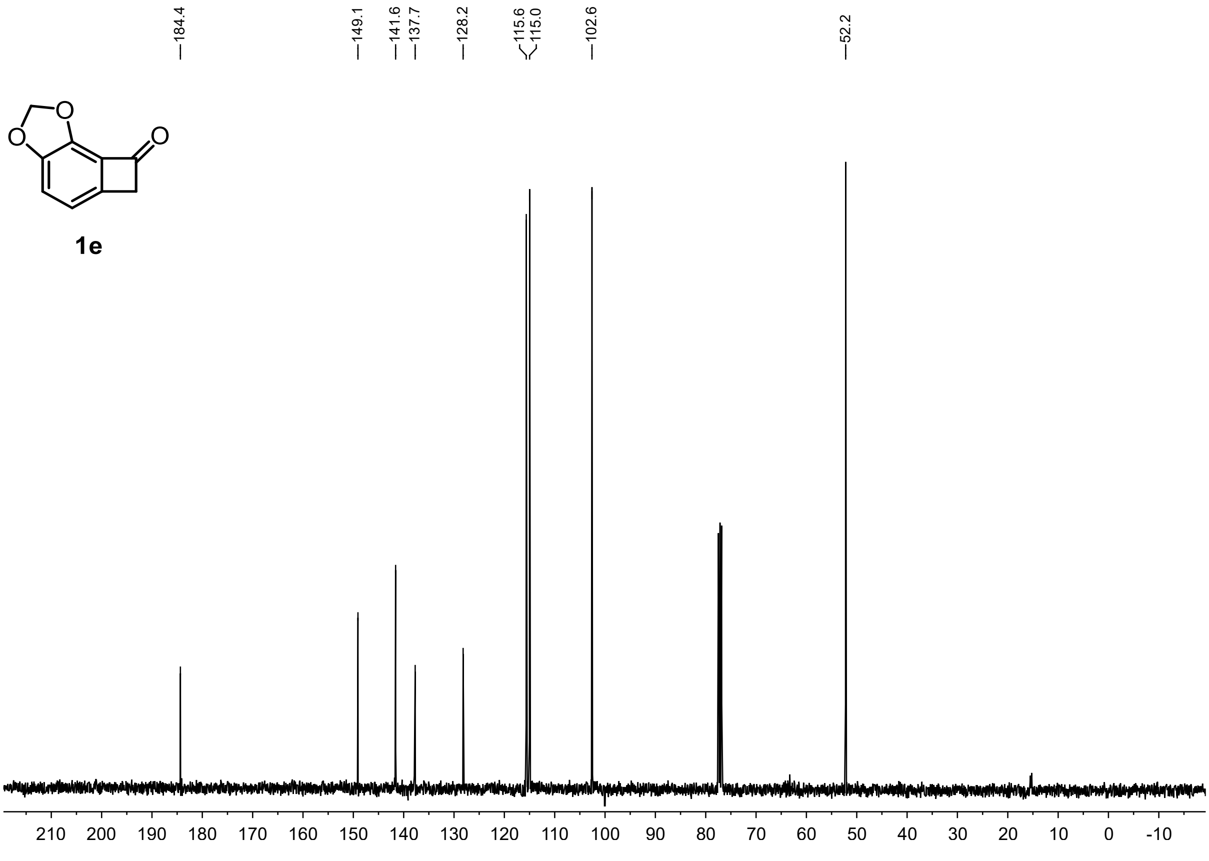

Figure S 33. ^{13}C NMR of **1e** in $CDCl_3$ measured at 100.63 MHz.

5-Fluorobicyclo[4.2.0]octa-1,3,5-trien-7-one

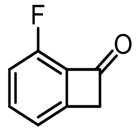

1f

1f was synthesized according to **GP-I** employing 1-bromo-2-fluorobenzene (3.50 g, 20.0 mmol, 1.00 equiv.), $NaNH_2$ (1.56 g, 40.0 mmol. 2.00 equiv.), and **9** (11.9 mL, 80.0 mmol, 4.00 equiv.). Purification *via* flash chromatography (40 g SiO_2, gradient from 100:0 to 85:15 *n*-hexane/EA over 10 CV) afforded **1f** (551 mg, 4.05 mmol, 20%) as a yellow solid.[7]

C_8H_5OF (136.13 $\frac{\mathrm{g}}{\mathrm{mol}}$)

R$_f$: 0.59 (*n*-hexane/EA = 80:20) [UV]

^{1}**H NMR**(700.21 MHz, $CDCl_3$): δ = 7.55 (m, 1H), 7.32 (dm, ^3J = 7.3 Hz, 1H), 6.99 (m, 1H), 3.99 (m, 2H).

^{13}**C NMR**(176.08 MHz, $CDCl_3$): δ = 183.2, 151.9 (d, ^4J = 4 Hz), 151.3 (d, ^1J = 267 Hz), 138.2 (d, ^3J = 7 Hz), 134.3 (d, ^2J = 17 Hz), 120.0 (d, ^3J = 5 Hz), 115.5 (d, ^2J = 20 Hz), 52.8.

^{19}**F NMR**(658.79 MHz, $CDCl_3$): δ =-106.8.

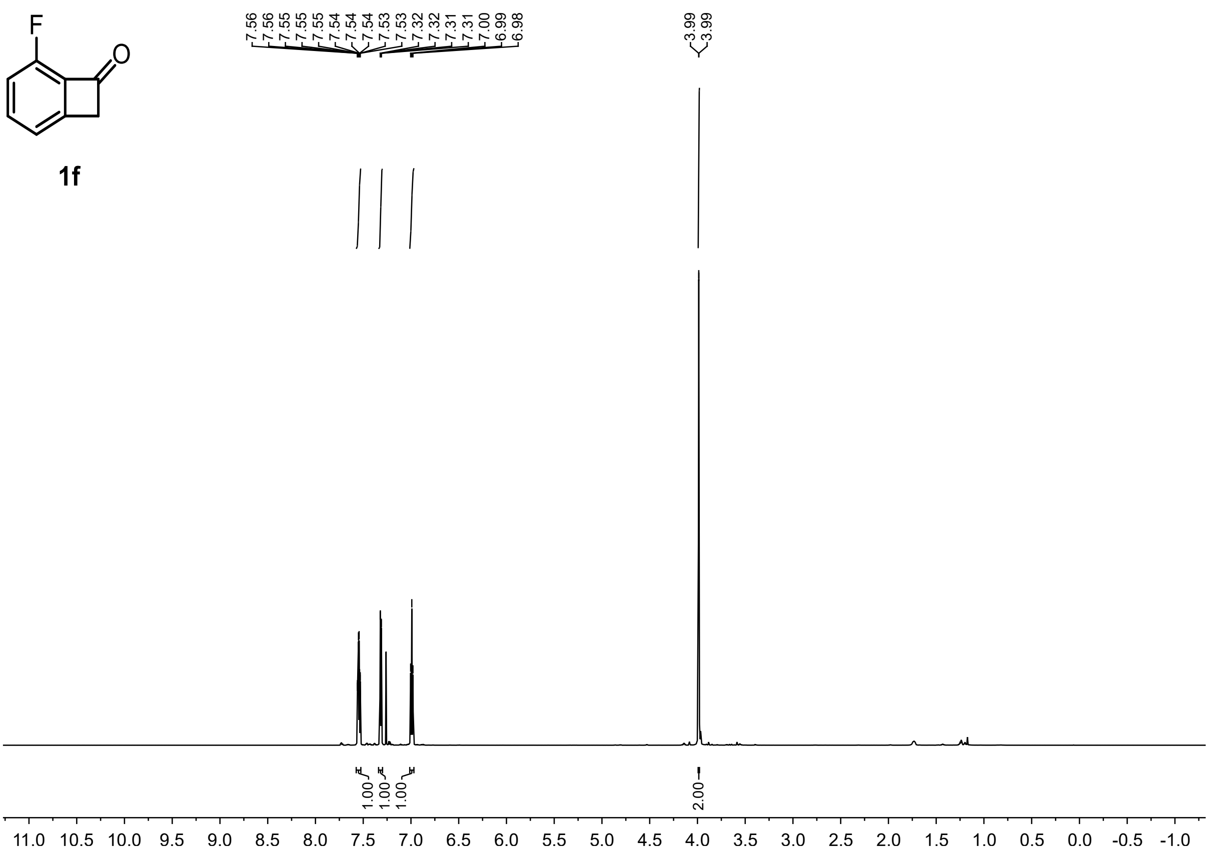

Figure S 34. ^{1}H NMR of **1f** in $CDCl_3$ measured at 700.21 MHz.

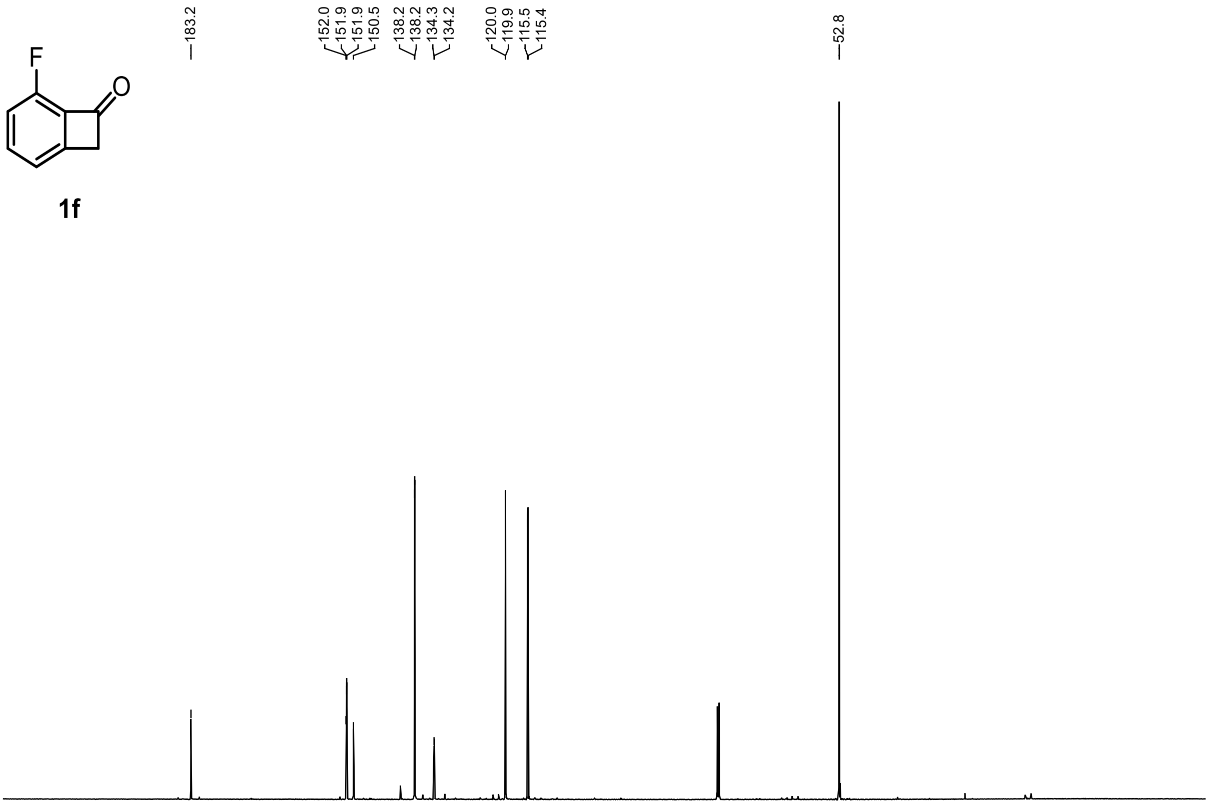

Figure S 35. ^{13}C NMR of **1f** in $CDCl_3$ measured at 176.08 MHz.

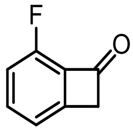

1f

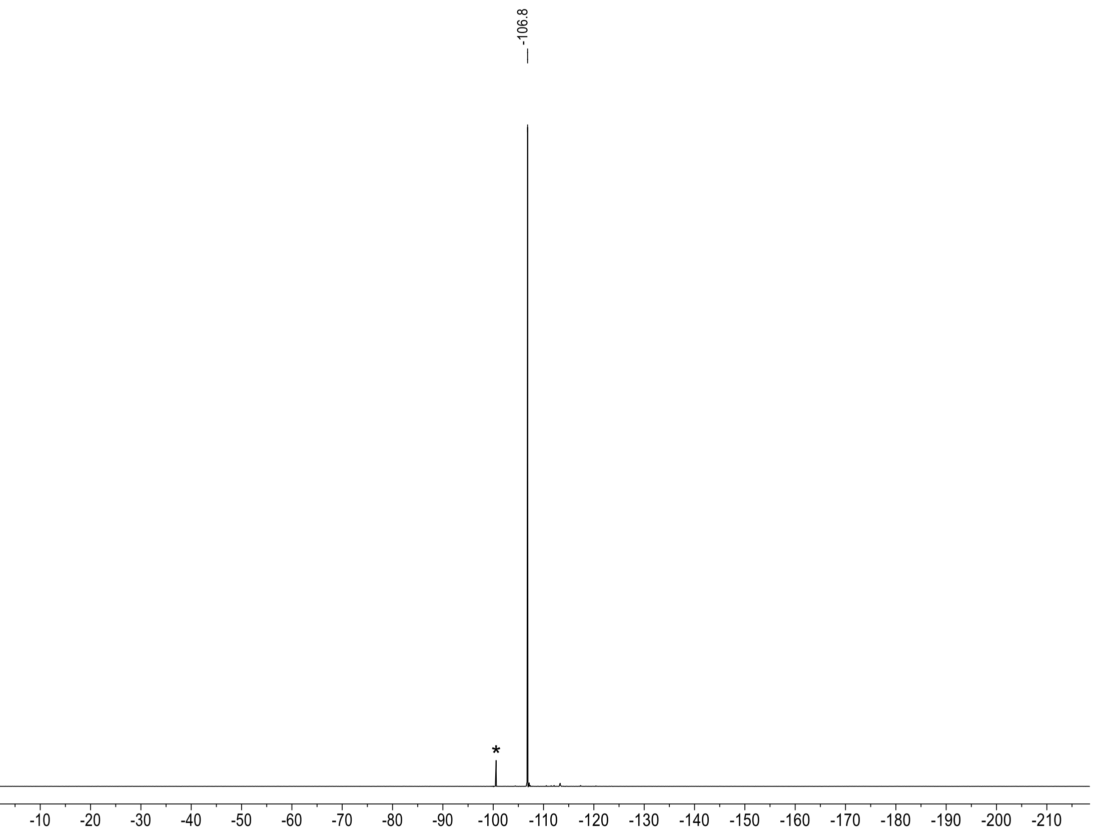

Figure S 36. ^{19}F NMR of **1f** in $CDCl_3$ measured at 658.79 MHz. * Denotes an unknown impurity.

8-methylbicyclo[4.2.0]octa-1,3,5-trien-7-one

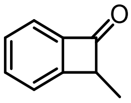

1g

1g was synthesized according to **GP-H** employing **8g** (1.63 g, 5.10 mmol, 1.00 equiv.) and ^{t}BuLi (6.00 mL, 1.7 M, 2.00 equiv.). Purification *via* flash chromatography (23 g SiO_2, gradient from 100:0 to 80:20 *n*-hexane/EA over 10 CV) afforded **1g** (514 mg, 3.89 mmol, 76%) as a pale yellow oil. The analytical data is in accordance with literature.[6]

C_9H_8O (132.16 $\frac{g}{mol}$)

R$_f$: 0.68 (*n*-hexane/EA = 80:20) [UV]

^{1}H NMR(400.16 MHz, $CDCl_3$): δ = 7.51 (m, 2H), 7.41 (m, 1H), 7.35 (dm, ^3J = 7.5 Hz, 1H), 4.27 (q, ^3J = 7.2 Hz, 1H), 1.45 (d, ^3J = 7.2 Hz, 3H).

^{13}C NMR(100.62 MHz, $CDCl_3$): δ = 193.3, 157.7, 146.4, 135.3, 129.2, 122.9, 121.1, 59.7, 15.2.

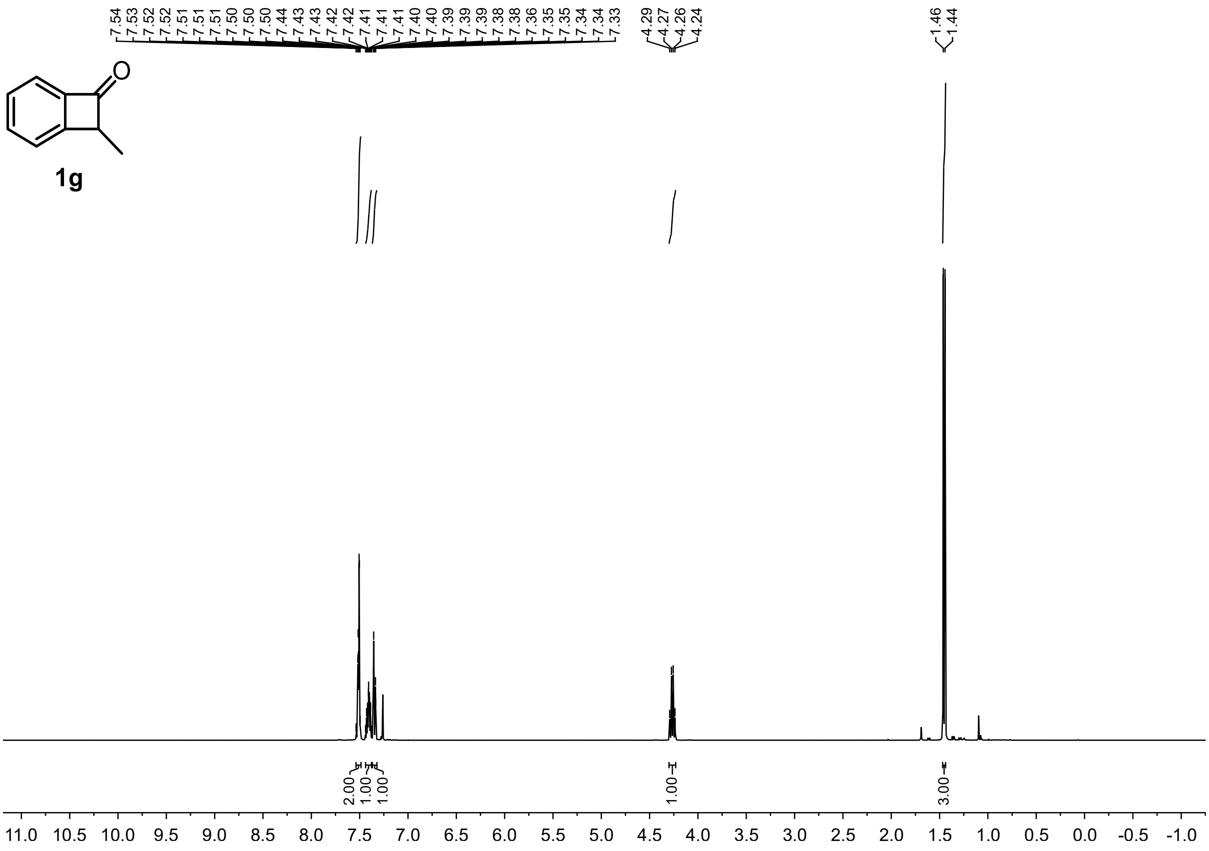

Figure S 37. ^{1}H NMR of **1g** in $CDCl_3$ measured at 400.16 MHz.

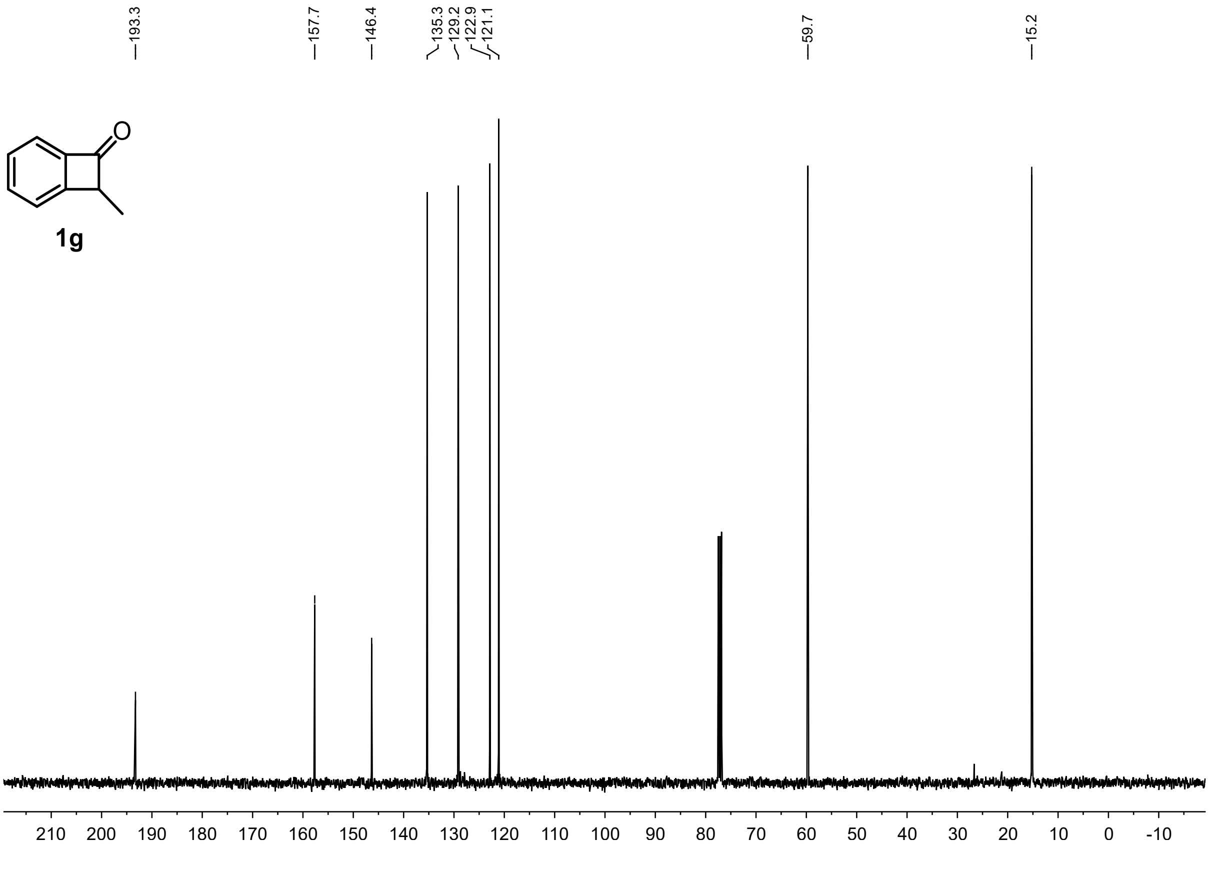

Figure S 38. ^{13}C NMR of **1g** in $CDCl_3$ measured at 100.63 MHz.

8-ethylbicyclo[4.2.0]octa-1,3,5-trien-7-one

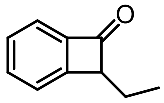

1h

1h was synthesized according to **GP-H** employing **8h** (2.10 g, 6.30 mmol, 1.00 equiv.) and ^{t}BuLi (7.78 mL, 1.70 M, 2.10 equiv.). Purification *via* flash chromatography (23 g SiO_2, gradient from 100:0 to 80:20 *n*-hexane/EA over 10 CV) afforded **1h** (587 mg, 4.01 mmol, 64%) as a pale yellow oil. The analytical data is in accordance with literature.[3]

$C_{10}H_{10}O$ (146.19 $\frac{g}{mol}$)

R$_f$: 0.77 (*n*-hexane/EA = 80:20) [UV]

^{1}H NMR(700.21 MHz, $CDCl_3$): δ = 7.52 (m, 2H), 7.42 (m, 1H), 7.36 (d, ^3J = 7.6 Hz, 1H), 4.21 (t, ^3J = 6.9 Hz, 1H), 1.94 (m, 1H), 1.81 (m, 1H), 1.05 (t, ^3J = 7.5 Hz, 3H).

^{13}C NMR(176.08 MHz, $CDCl_3$): δ = 193.2, 156.7, 146.9, 135.2, 129.2, 123.5, 120.9, 66.5, 23.6, 11.8.

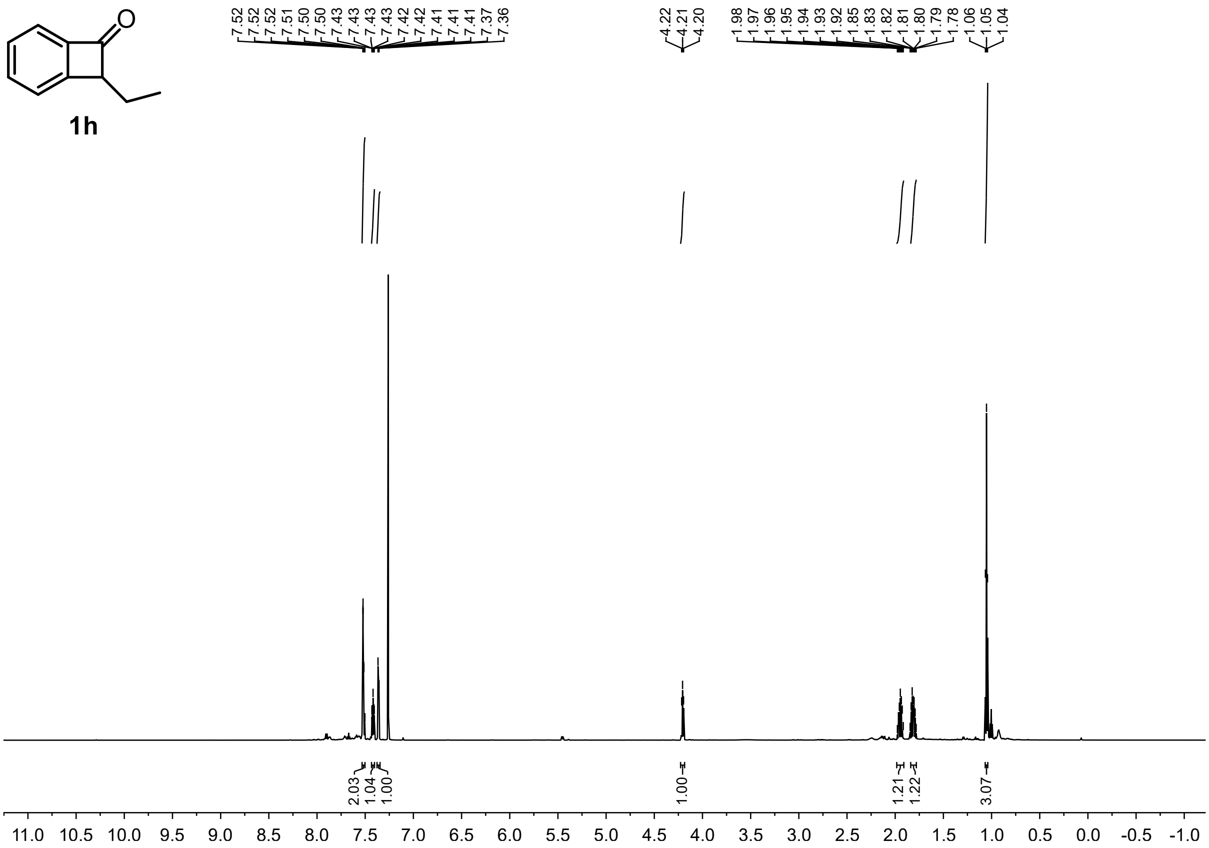

Figure S 39. ^{1}H NMR of **1h** in $CDCl_3$ measured at 700.21 MHz.

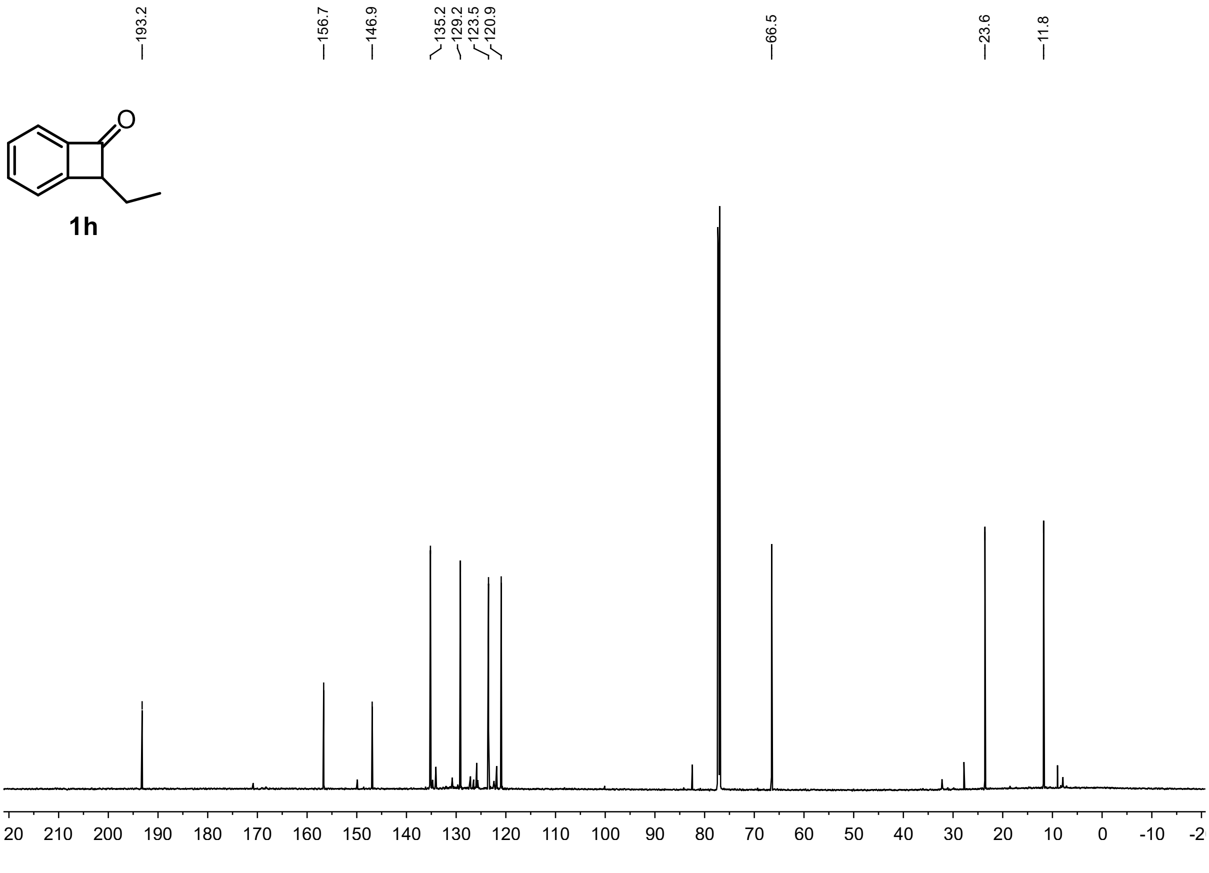

Figure S 40. ^{13}C NMR of **1h** in $CDCl_3$ measured at 176.08 MHz.

8-isopropylbicyclo[4.2.0]octa-1,3,5-trien-7-one

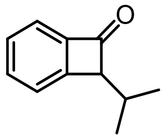

1i

1i was synthesized according to **GP-H** employing **8i** (1.70 g, 4.90 mmol, 1.00 equiv.) and ^{t}BuLi (5.76 mL, 1.70 M, 2.00 equiv.). Purification *via* flash chromatography (23 g SiO_2, gradient from 100:0 to 85:15 *n*-hexane/EA over 10 CV) afforded **1i** (701 mg, 4.38 mmol, 89%) as a pale yellow oil. The analytical data is in accordance with literature.[16]

$C_{11}H_{12}O$ (160.22 $\frac{\text{g}}{\text{mol}}$)

R_f: 0.80 (*n*-hexane/EA = 80:20) [UV]

^{1}H NMR(400.16 MHz, $CDCl_3$): δ = 7.52 (m, 2H), 7.41 (m, 1H), 7.35 (dm, ^3J = 7.5 Hz, 1H), 4.06 (d, ^3J = 7.3 Hz, 1H), 2.14 (dqq, ^3J = 7.3 Hz, ^3J = 6.8 Hz, ^3J = 6.7 Hz, 1H), 1.11 (d, ^3J = 6.8 Hz, 3H), 1.00 (d, ^3J = 6.7 Hz, 3H).

^{13}C NMR(100.62 MHz, $CDCl_3$): δ = 192.9, 155.9, 147.2, 135.0, 129.1, 123.9, 120.6, 71.9, 29.9, 21.0, 20.1.

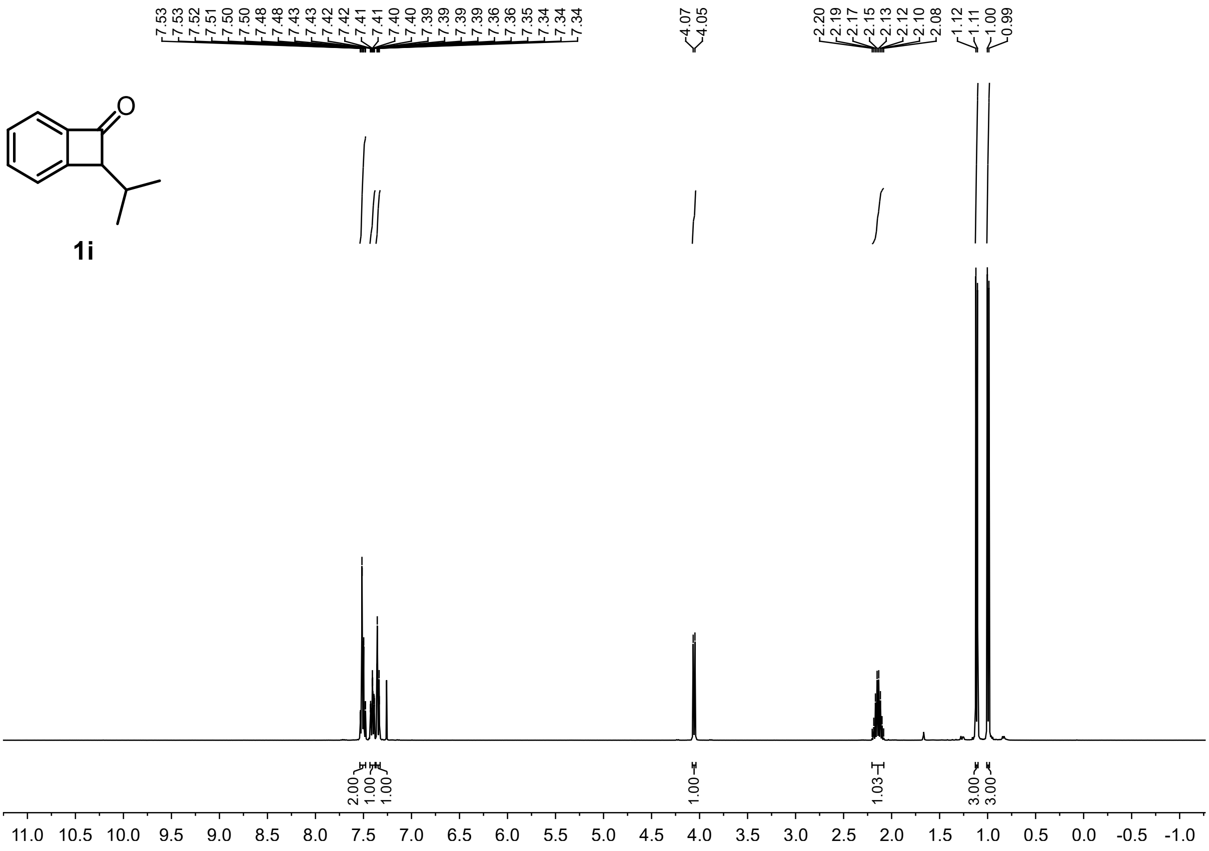

Figure S 41. ^{1}H NMR of **1i** in $CDCl_3$ measured at 400.16 MHz.

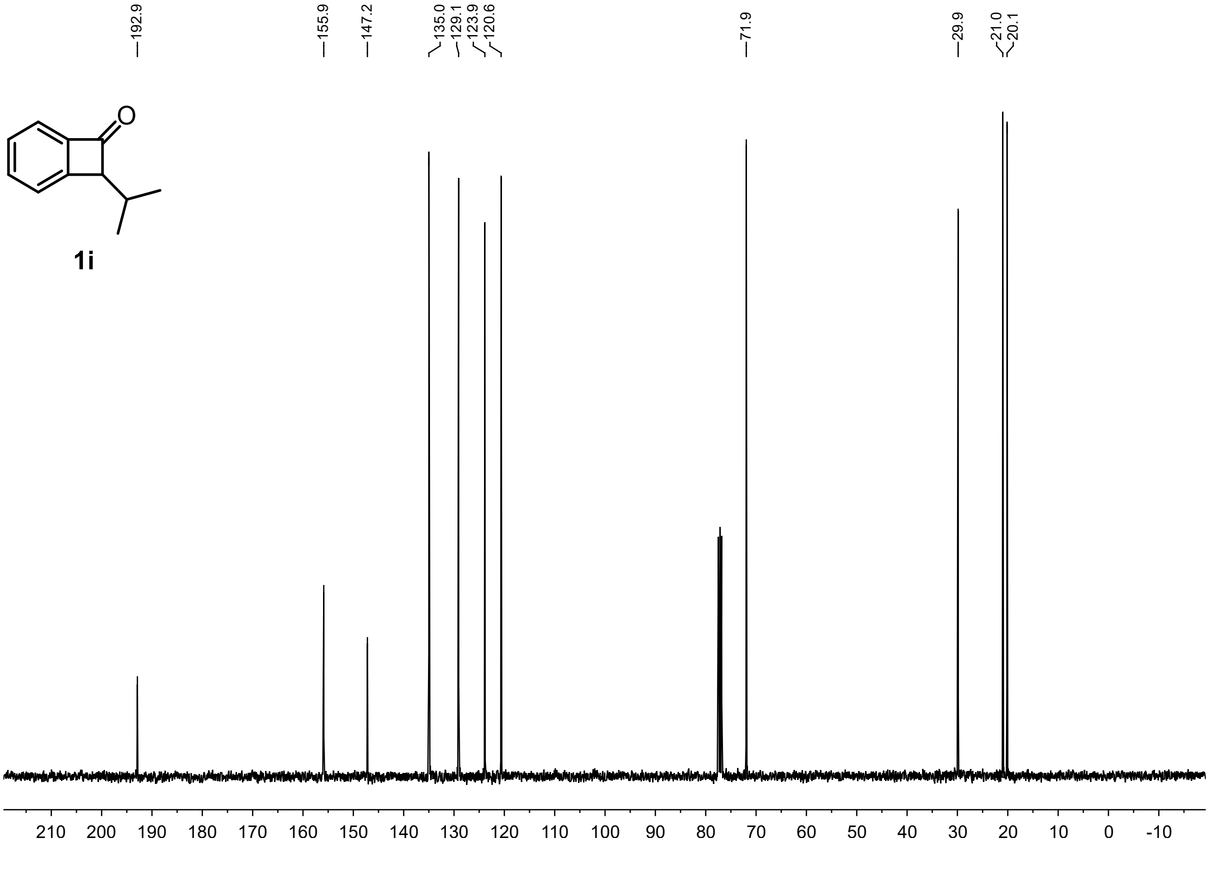

Figure S 42. ^{13}C NMR of **1i** in $CDCl_3$ measured at 100.63 MHz.

8-allylbicyclo[4.2.0]octa-1,3,5-trien-7-one

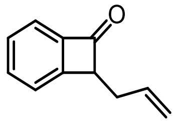

1j

1j was synthesized according to **GP-H** employing **8j** (783 mg, 2.27 mmol, 1.00 equiv.) and ^{t}BuLi (2.67 mL, 1.70 M, 2.00 equiv.). Purification *via* flash chromatography (23 g SiO_2, gradient from 100:0 to 90:10 *n*-hexane/EA over 7 CV) afforded **1j** (283 mg, 1.79 mmol, 79%) as a pale yellow oil. The analytical data is in accordance with literature.[3]

$C_{11}H_{10}O$ (158.20 $\frac{g}{mol}$)

R$_f$: 0.75 (*n*-hexane/EA = 80:20) [UV

^{1}H NMR(400.16 MHz, $CDCl_3$): δ = 7.51 (m, 2H), 7.42 (m, 1H), 7.36 (dm, ^3J = 7.5 Hz, 1H), 5.89 (dddd, ^3J = 17.1 Hz, ^3J = 10.2 Hz, ^3J = 7.6 Hz, ^3J = 6.5 Hz, 1H), 5.13 (dm, ^3J = 17.1 Hz, 1H), 5.09 (dm, ^3J = 10.2 Hz, 1H), 4.30 (dd, ^3J = 8.8 Hz, ^3J = 5.8 Hz, 1H), 2.71 (ddddd, ^2J = 14.6 Hz, ^3J = 6.5 Hz, ^3J = 5.8 Hz, ^4J = 1.5 Hz, ^4J = 1.5 Hz, 1H), 2.46 (ddddd, ^2J = 14.6 Hz, ^3J = 8.8 Hz, ^3J = 7.6 Hz, ^4J = 1.1 Hz, ^4J = 1.1 Hz, 1H).

^{13}C NMR(100.62 MHz, $CDCl_3$): δ = 192., 156.2, 146.9, 135.2, 134.9, 129.3, 123.7, 121.0, 117.1, 64.1, 34.7.

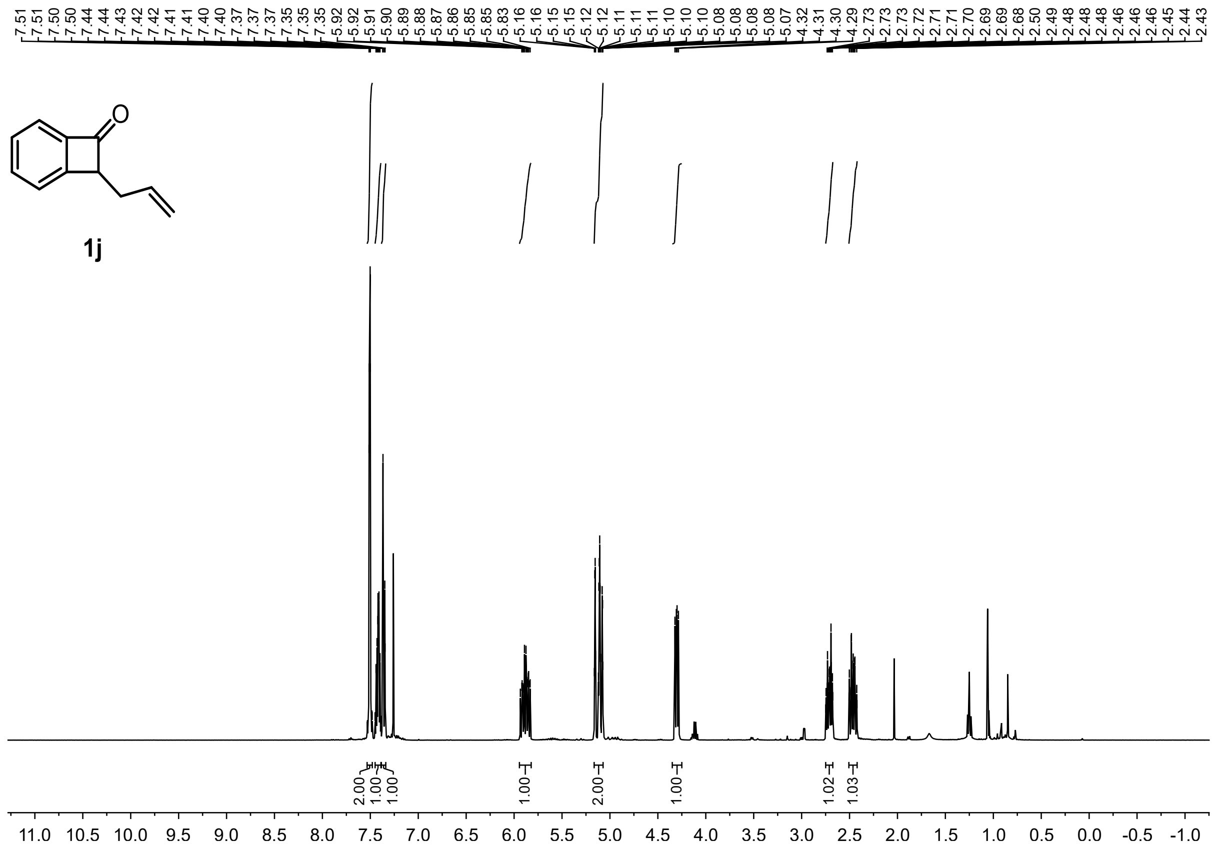

Figure S 43. ^{1}H NMR of **1j** in $CDCl_3$ measured at 400.16 MHz.

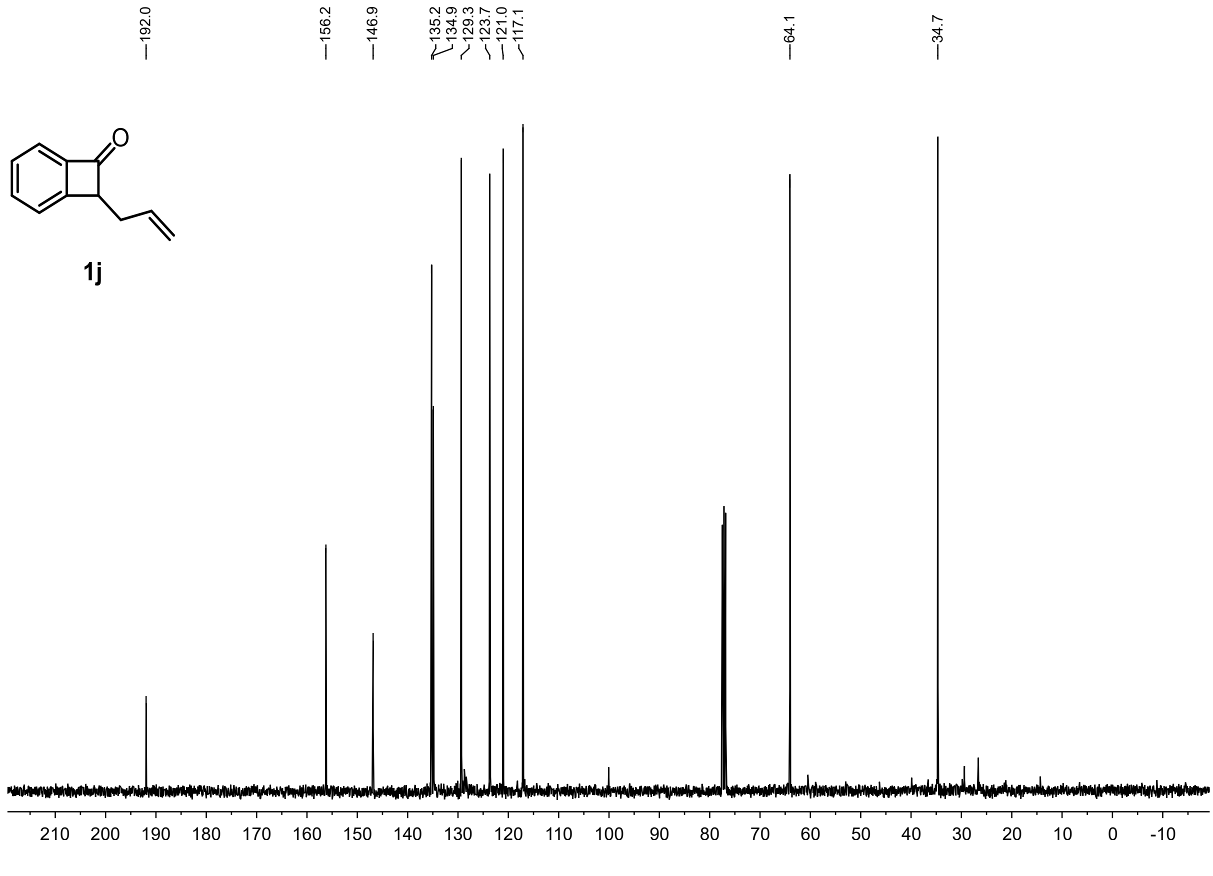

Figure S 44. ^{13}C NMR of **1j** in $CDCl_3$ measured at 100.63 MHz.

spiro[bicyclo[4.2.0]octane-7,1'-cyclopropane]-1,3,5-trien-8-one

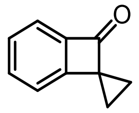

1k

1k was synthesized according to **GP-H** employing **8k** (540 mg, 1.90 mmol, 1.00 equiv.) and ^{t}BuLi (2.35 mL, 1.70 M, 2.10 equiv.). Purification *via* flash chromatography (23 g SiO_2, gradient from 100:0 to 90:10 *n*-hexane/EA over 15 CV) afforded **1k** (262 mg, 1.82 mmol, 96%) as a colorless solid, which was recrystallized from *n*-hexane yielding **1k** (223 mg, 1.54 mmol, 81%) as colorless crystals.

$C_{10}H_8O$ (144.17 $\frac{g}{mol}$)

mp: 86.9 °C.

R$_f$: 0.75 (*n*-hexane/EA = 80:20) [UV]

^{1}H NMR(400.16 MHz, $CDCl_3$): δ = 7.44 (m, 1H), 7.32 (m, 1H), 7.27 (dm, ^3J = 7.5 Hz, 1H), 7.17 (d, ^3J = 7.4 Hz, 1H), 1.47 (m, 2H), 1.24 (m, 2H).

^{13}C NMR(100.62 MHz, $CDCl_3$): δ = 195.0, 158.0, 148.1, 135.3, 128.1, 119.6, 119.3, 47.1, 13.1.

HRMS (ESI-TOF) m/z: $[M+Na]^+$ Calcd for $C_{10}H_8ONa$ 167.0467; Found 167.0466.

IR (ATR, ṽ): 1739 cm^{-1} (s, *CO*).

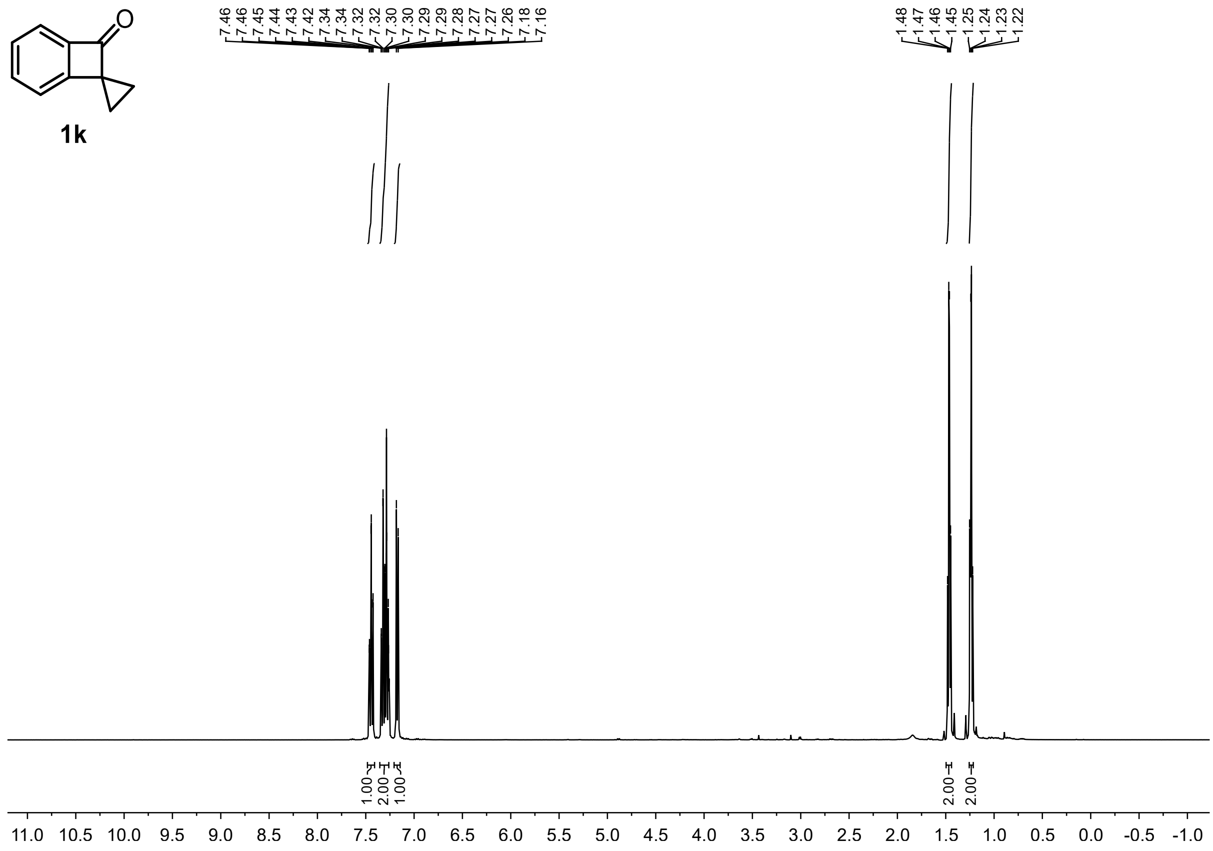

Figure S 45. ^{1}H NMR of **1k** in $CDCl_3$ measured at 400.16 MHz.

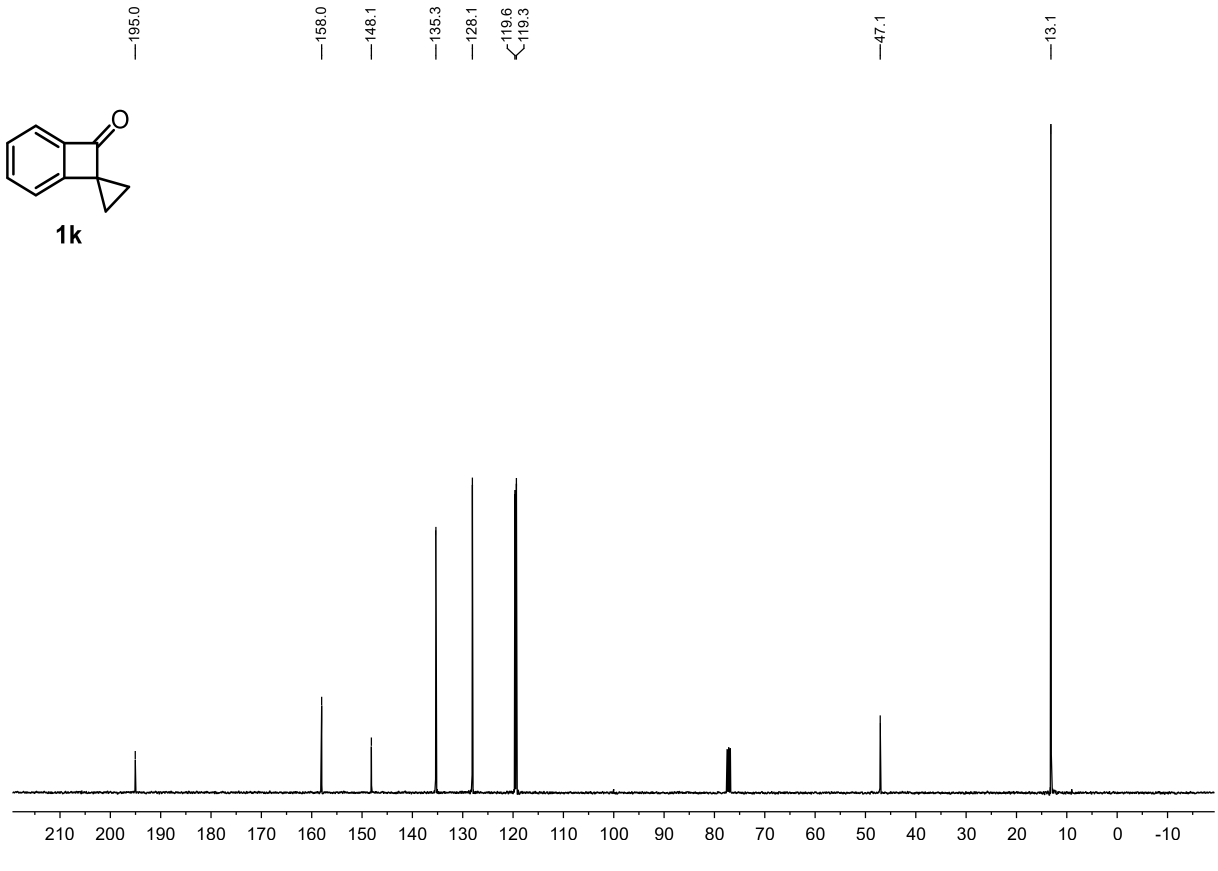

Figure S 46. ^{13}C NMR of **1k** in $CDCl_3$ measured at 100.63 MHz.

4-isobutyl-8-methylbicyclo[4.2.0]octa-1,3,5-trien-7-one

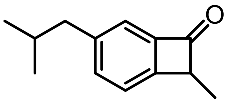

1l

1l was synthesized according to **GP-H** employing **8l** (1.34 g, 3.57 mmol, 1.00 equiv.) and ^{t}BuLi (4.41 mL, 1.70 M, 2.10 equiv.). Purification *via* flash chromatography (23 g SiO_2, gradient from 100:0 to 85:15 *n*-hexane/EA over 15 CV) afforded **1l** (436 mg, .2.32 mmol, 65%) as a pale colorless oil.

$C_{13}H_{16}O$ (188.27 $\frac{g}{mol}$)

R$_f$: 0.61 (*n*-hexane/EA = 80:20) [UV]

^{1}H NMR(700.21 MHz, $CDCl_3$): δ = 7.41 (d, ^3J = 7.6 Hz, 1H), 7.30 (dd, ^3J = 7.6 Hz, ^3J = 1.2 Hz, 1H), 7.12 (s, 1H), 4.20 (q, ^3J = 7.2 Hz, 1H), 2.51 (d, ^3J = 7.2 Hz, 2H), 1.83 (tsep, ^3J = 7.2 Hz, ^3J = 6.7 Hz, 1H), 1.43 (d, ^3J = 7.2 Hz, 3H), 0.89 (d, ^3J = 6.7 Hz, 6H).

^{13}C NMR(176.08 MHz, $CDCl_3$): δ = 193.5, 155.3, 146.5, 143.1, 136.7, 122.4, 121.1, 58.9, 45.6, 30.5, 22.3, 15.4.

HRMS (ESI-TOF) m/z: $[M+Na]^+$ Calcd for $C_{13}H_{16}ONa$ 211.1093; Found 211.1091.

IR (ATR, ṽ): 1757 cm^{-1} (vs, *CO*).

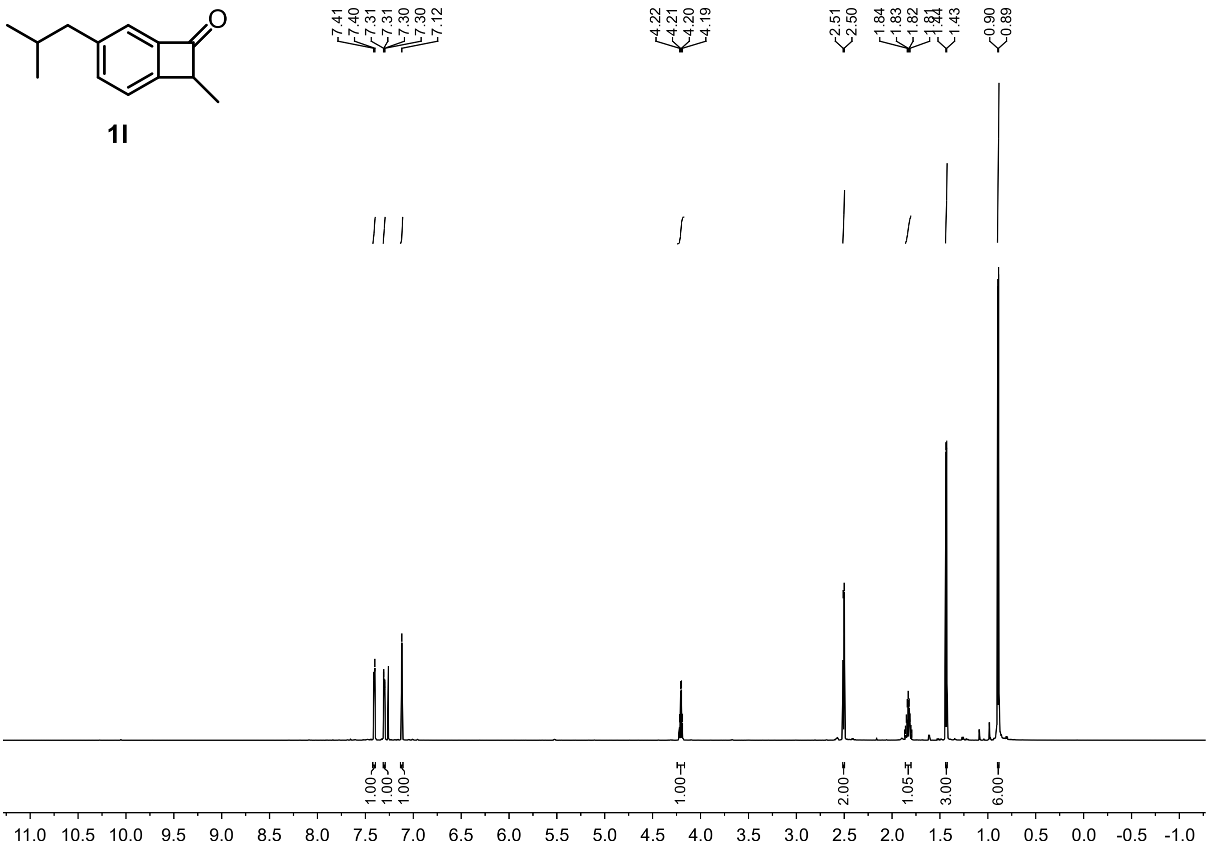

Figure S 47. ^{1}H NMR of **1l** in $CDCl_3$ measured at 700.21 MHz.

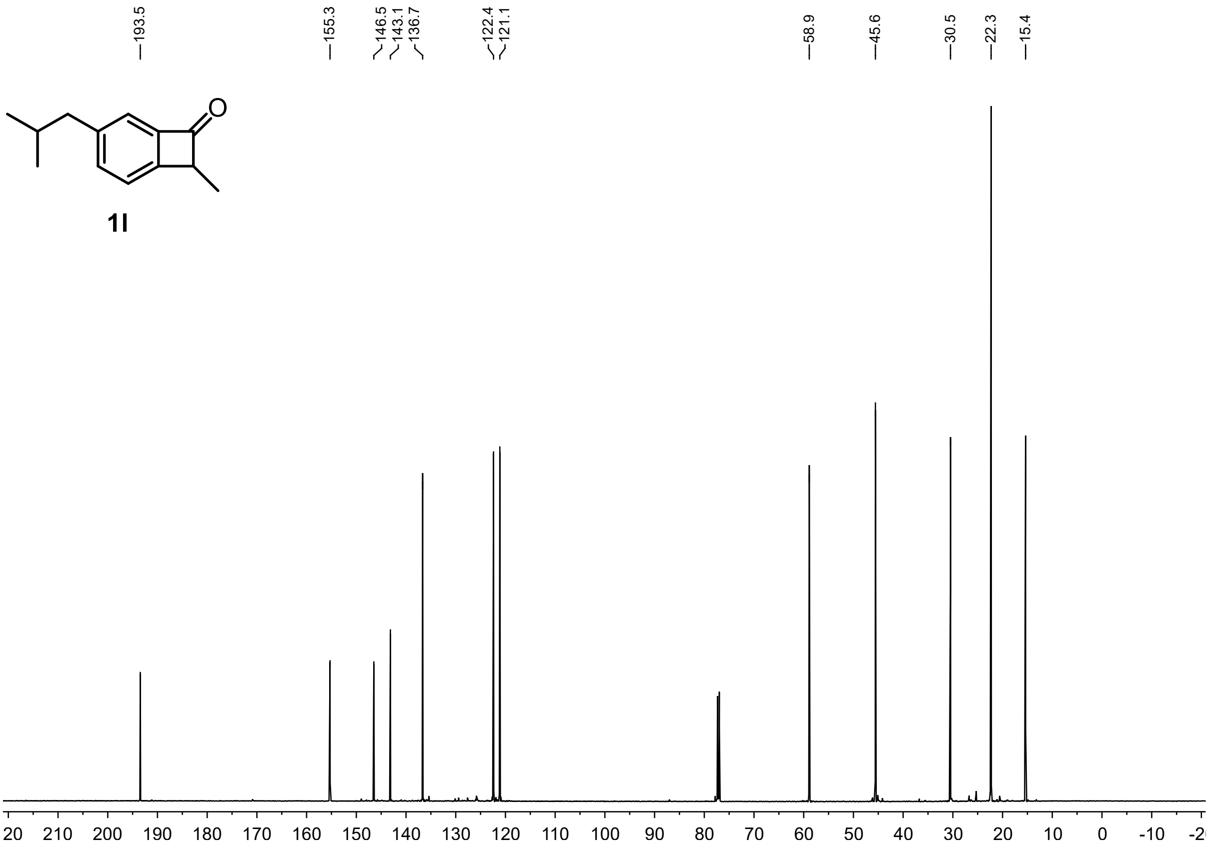

Figure S 48. ^{13}C NMR of **1l** in $CDCl_3$ measured at 176.08 MHz.

5-methylbicyclo[4.2.0]octa-1,3,5-trien-7-one

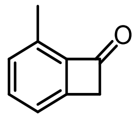

1m

1m was synthesized according to **GP-J** employing **10m** (3.00 g, 9.60 mmol, 1.00 equiv.), **9** (2.85 mL, 19.2 mmol, 2.00 equiv.), CsF (2.19 g, 14.4 mmol, 1.50 equiv.), and TFA (1.47 mL, 19.2 mmol, 2.00 equiv.). Purification *via* flash chromatography (23 g SiO_2, gradient from 100:0 to 88:12 *n*-hexane/EA over 12 CV) and recrystallisation from *n*-hexane afforded **1m** (157 mg, 1.15 mmol, 12%) as a colorless solid. The analytical data is in accordance with literature.[17]

C_9H_8O (132.16 $\frac{g}{mol}$)

R$_f$: 0.55 (*n*-hexane/EA = 90:10) [UV]

^{1}H NMR(600.13 MHz, $CDCl_3$): δ = 7.40 (m, 1H), 7.32 (d, ^3J = 7.3 Hz, 1H), 7.14 (d, ^3J = 7.6 Hz, 1H), 3.93 (s, 2H), 2.41 (s, 3H).

^{13}C NMR(150.90 MHz, $CDCl_3$): δ = 189.3, 151.0, 146.7, 135.1, 133.7, 129.6, 120.6, 52.1, 17.7.

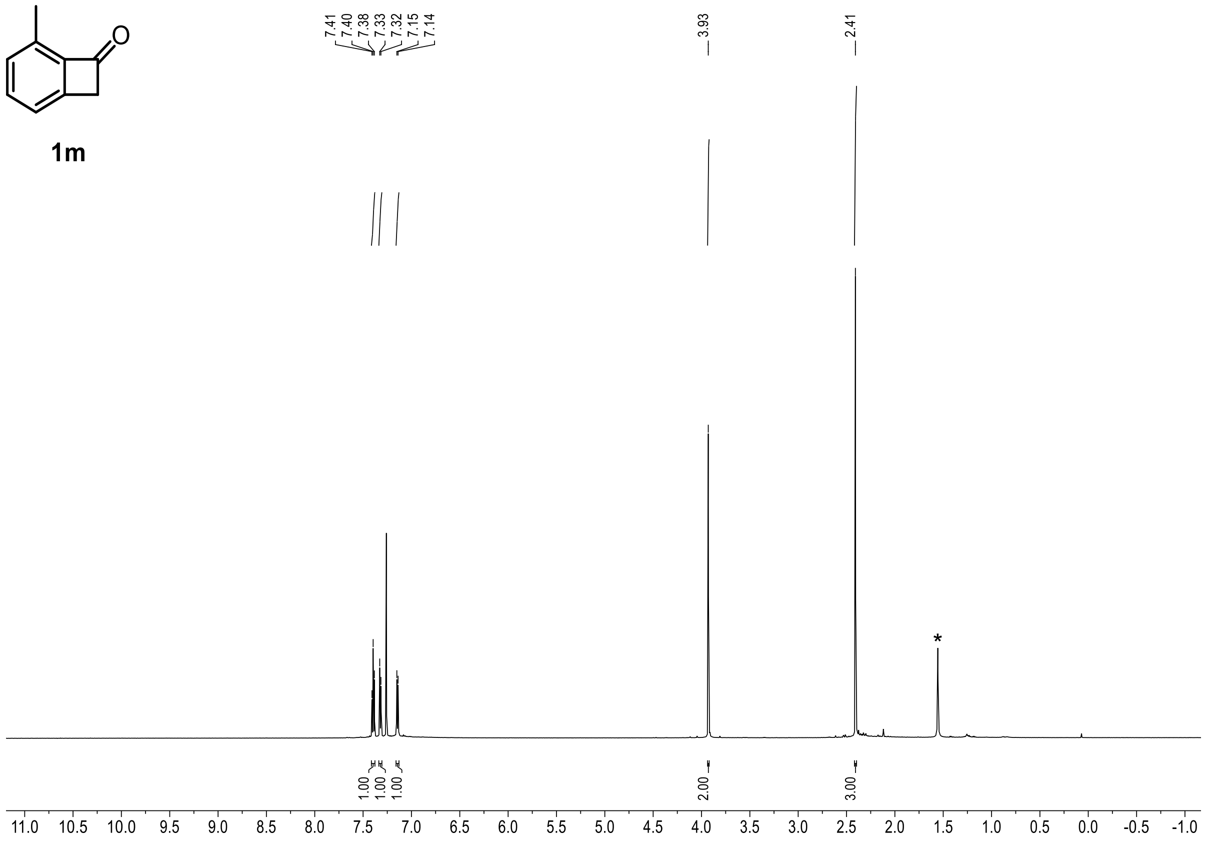

Figure S 49. ¹H NMR of **1m** in $CDCl_3$ measured at 600.13 MHz.

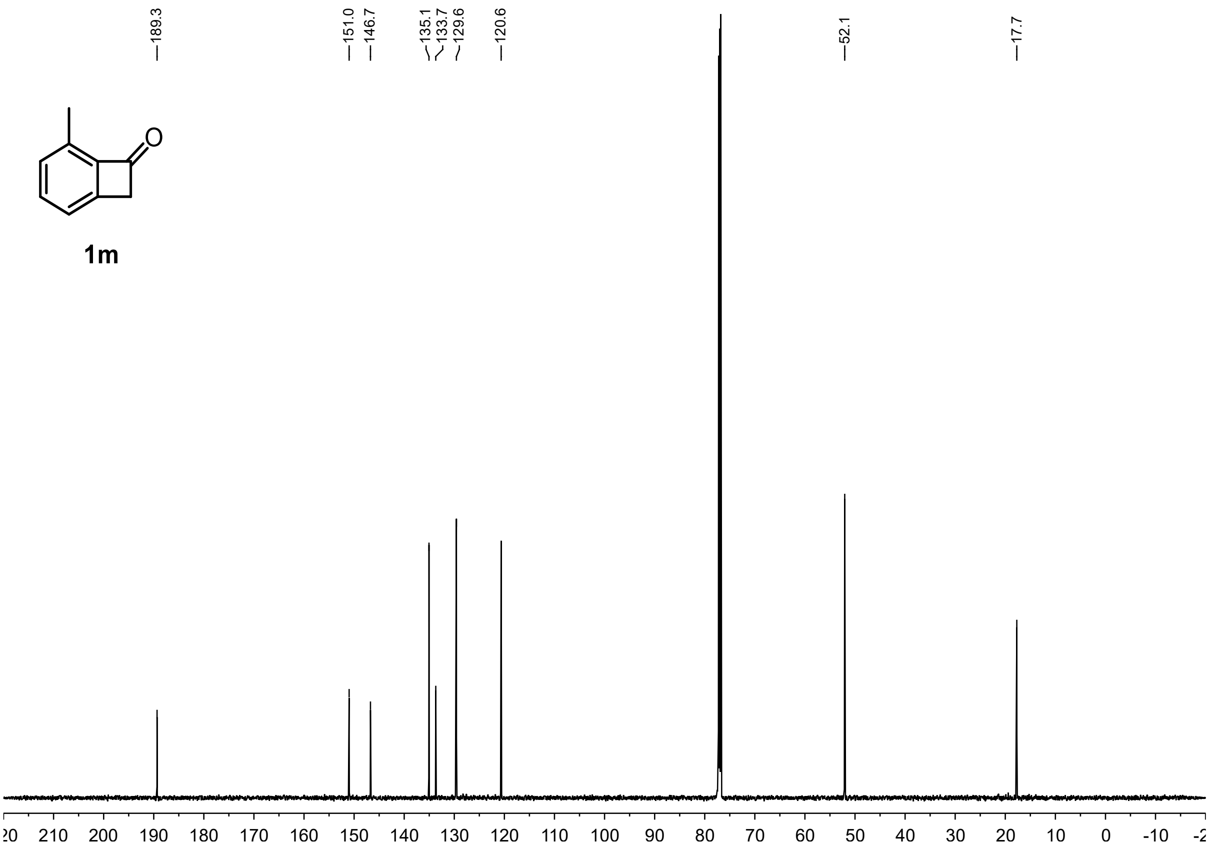

Figure S 50. ¹³C NMR of **1m** in $CDCl_3$ measured at 150.92 MHz.

5-methoxybicyclo[4.2.0]octa-1,3,5-trien-7-one

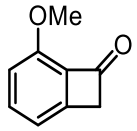

1n

1n was synthesized according to **GP-I** employing 2-bromo anisole (3.74 g, 20.0 mmol, 1.00 equiv.), $NaNH_2$ (3.12 g, 80.0 mmol. 4.00 equiv.), and **9** (11.9 mL, 80.0 mmol, 4.00 equiv.). Purification *via* flash chromatography (40 g SiO_2, gradient from 100:0 to 90:10 *n*-hexane/EA over 10 CV) afforded **1n** (1.53 g, 10.3 mmol, 52%) as a colorless solid. The analytical data is in accordance with literature.[7]

$C_9H_8O_2$ (148.16 $\frac{g}{mol}$)

R_f: 0.25 (*n*-hexane/EA = 80:20) [UV]

^{1}H NMR(700.21 MHz, $CDCl_3$): δ = 7.42 (dd, ^3J = 8.4 Hz, ^3J = 7.1 Hz, 1H), 7.02 (d, 7.0 Hz, 1H), 6.79 (d, ^3J = 8.4 Hz, 1H), 4.10 (s, 3H), 3.91 (s, 2H).

^{13}C NMR(176.08 MHz, $CDCl_3$): δ = 185.0, 153.5, 150.7, 137.7, 132.5, 115.9, 115.2, 59.9, 51.4.

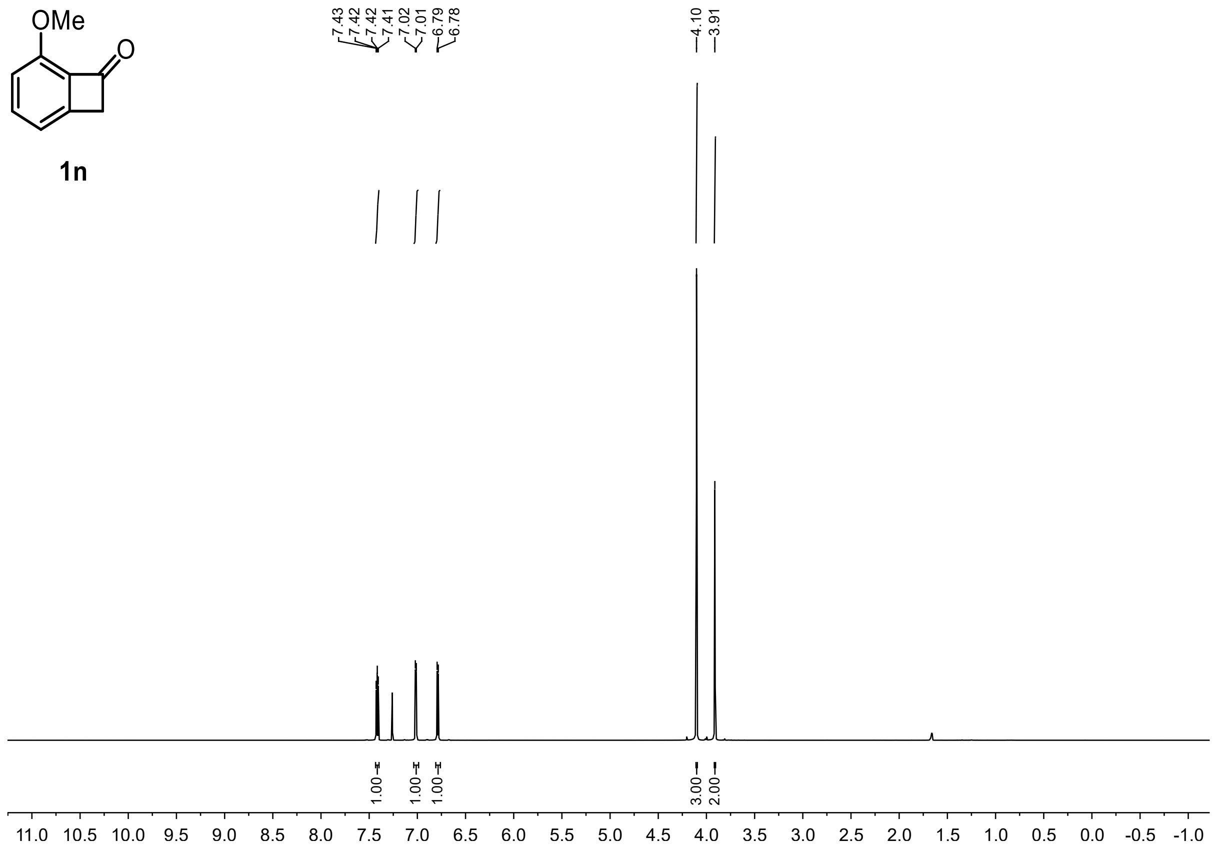

Figure S 51. ^{1}H NMR of **1n** in $CDCl_3$ measured at 700.21 MHz.

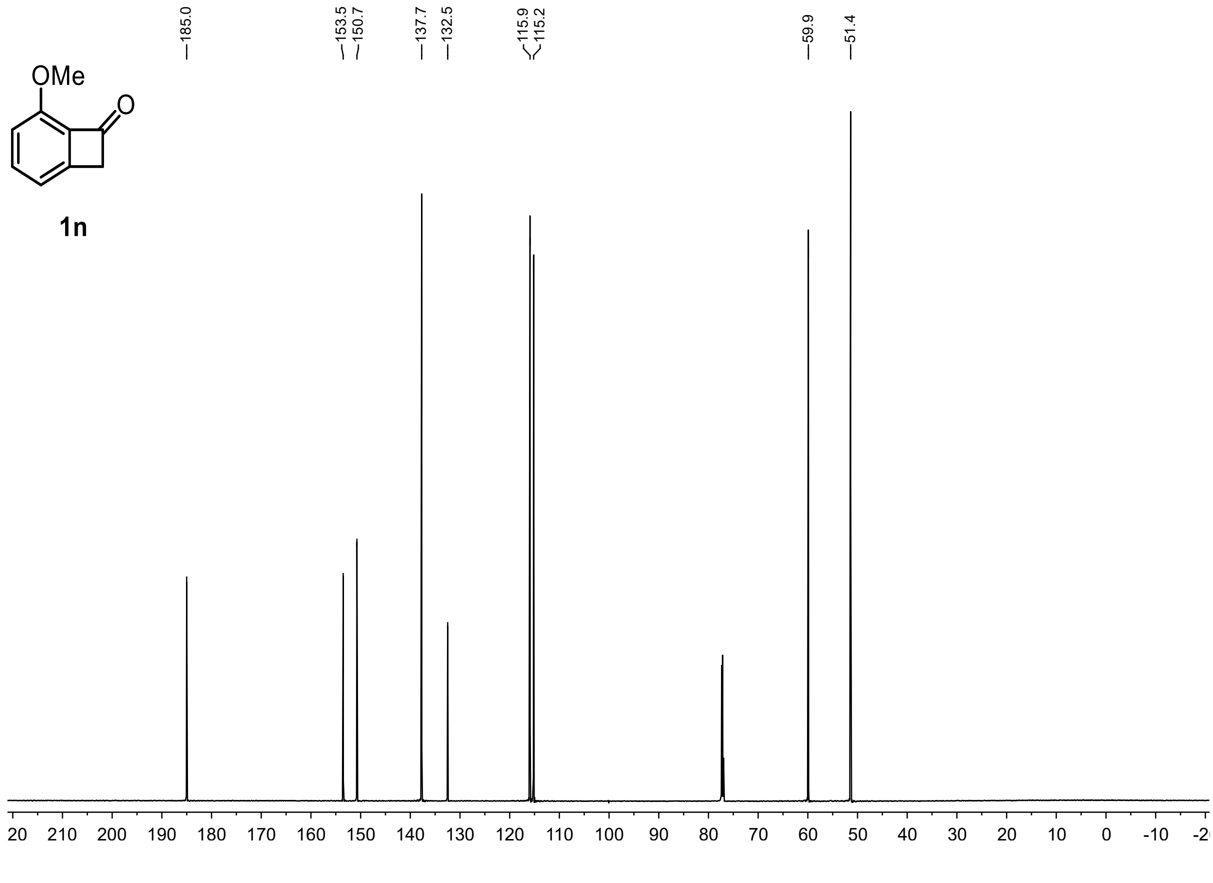

Figure S 52. ^{13}C NMR of **1n** in $CDCl_3$ measured at 176.08 MHz.

4.4 Norbornadienes

7-(*tert*-butoxy)bicyclo[2.2.1]hepta-2,5-diene

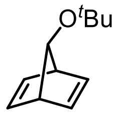

2b

2b was synthesized according to literature.[18] In a RBF NBD (98.9 mL, 0.98 mol, 2.6 equiv.) and CuBr (215 mg, 1.50 mmol, 0.4 mol%) were dissolved in benzene (160 mL) and heated to a reflux. *tert*-Butyl perbenzoate (71.5 mL, 0.38 mol, 1.00 equiv.) dissolved in benzene (25 mL) was added dropwise over 1 h. Afterwards, the mixture was refluxed for 1 h, cooled to rt and washed with brine (3 × 100 mL), $NaOH_{(aq.)}$ (3 × 100 mL, 10%) and again with brine (50 mL). The solution was dried over $MgSO_4$ and carefully concentrated *in vacuo*. **2b** (12.9 g, 78.5 mmol, 21%) was obtained upon vacuum distillation (7 mbar, 62 °C, 80 °C oil bath) as a colorless oil. The analytical data is in accordance with literature.[19]

$C_{11}H_{16}O$ (164.25 $\frac{\mathrm{g}}{\mathrm{mol}}$)

b.p. (7 mbar): 62 °C.

^{1}H NMR(400.16 MHz, $CDCl_3$): δ = 6.64 (dd, ^3J = 2.7 Hz, ^3J = 2.2 Hz, 2H), 6.58 (m, 2H), 3.78 (m, 1H), 3.39 (m, 2H), 1.14 (s, 9H).

^{13}C NMR(100.62 MHz, $CDCl_3$): δ = 140.0, 137.4, 104.5, 73.7, 55.6, 28.5.

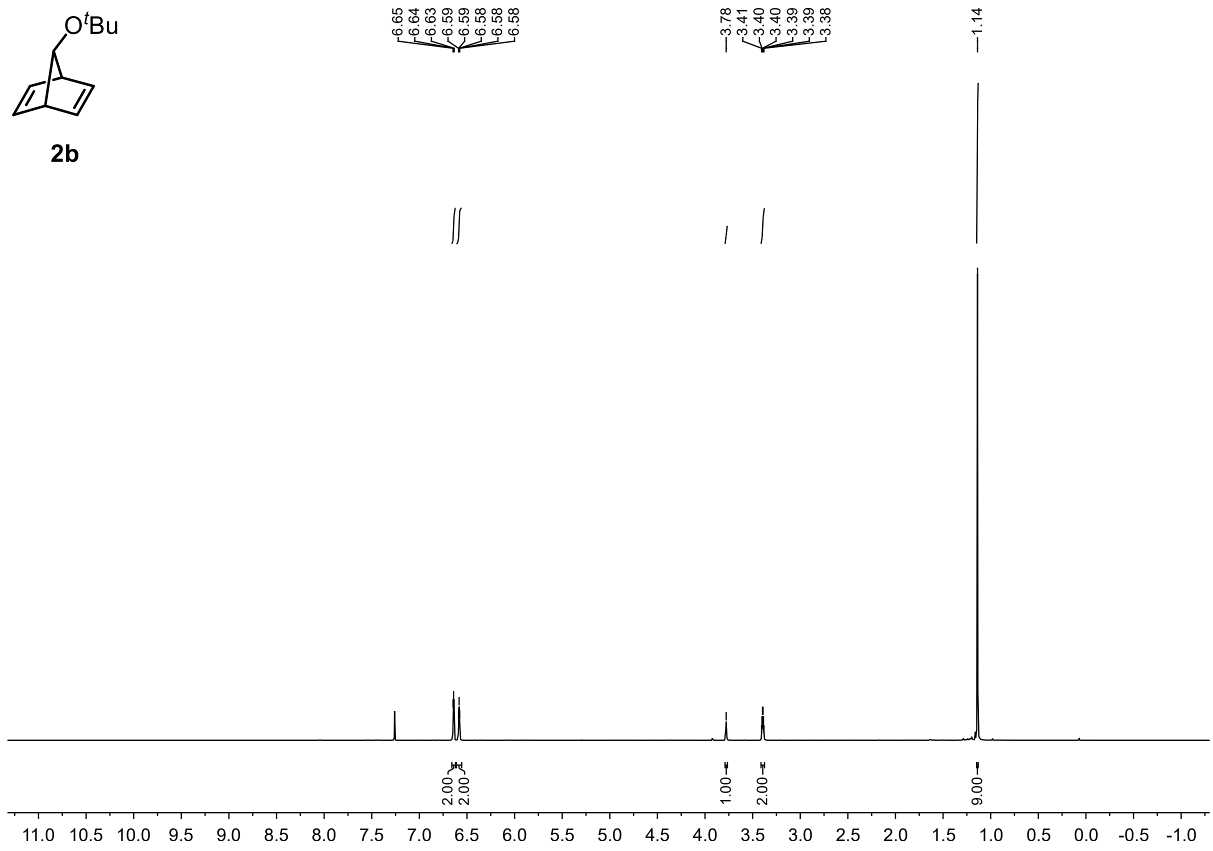

Figure S 53. ^{1}H NMR of **2b** in $CDCl_3$ measured at 400.16 MHz.

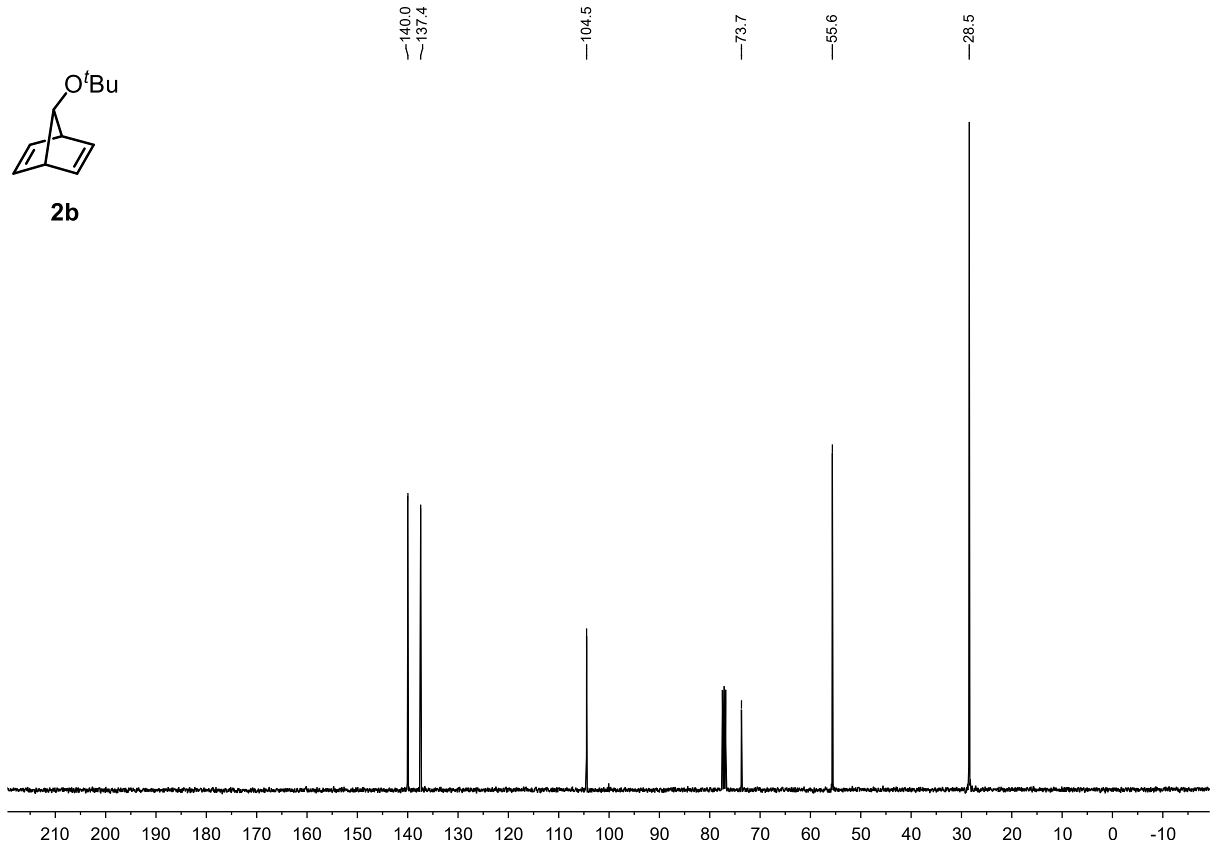

Figure S 54. ^{13}C NMR of **2b** in $CDCl_3$ measured at 100.63 MHz.

7-(isopropyl)bicyclo[2.2.1]hepta-2,5-diene

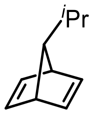

2c

2c was synthesize d according to **GP-K** employing **2b** (1.97 g, 12.0 mmol, 1.00 equiv.), and ^{i}PrMgBr (8.00 mL, 3.00 M, 2.00 equiv.). Vacuum distillation furnished **2c** (0.21 g, 1.56 mmol, 13%) as a colorless oil. The analytical data is in accordance with literature.[20]

$C_{10}H_{14}$ (134.22 $\frac{g}{mol}$)

^{1}H NMR(700.21 MHz, $CDCl_3$): δ = 6.81 (dd, ^{3}J = 2.4 Hz, ^{3}J = 1.9 Hz, 2H), 6.58 (dd, ^{3}J = 2.4 Hz, ^{3}J = 1.9 Hz, 2H), 3.42 (ddd, ^{3}J = 3.8 Hz, ^{3}J = 1.9 Hz, ^{3}J = 1.9 Hz, 2H), 2.09 (d, ^{3}J = 9.8 Hz, 1H), 1.62 (dsep, ^{3}J = 9.8 Hz, ^{3}J = 6.7 Hz, 1H), 0.75 (d, ^{3}J = 6.7 Hz, 6H).

^{13}C NMR(176.08 MHz, $CDCl_3$): δ = 144.9, 140.0, 96.3, 52.7, 27.2, 21.3.

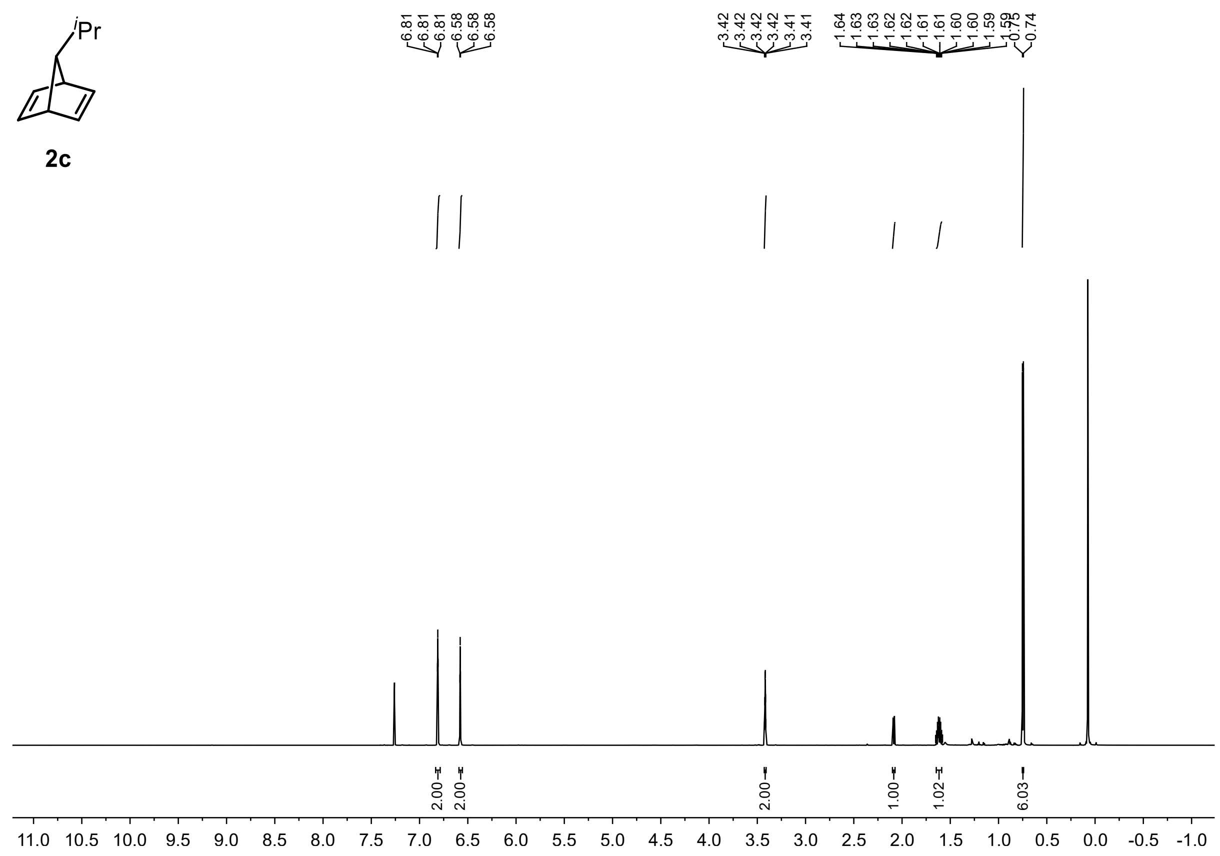

Figure S 55. ^{1}H NMR of **2c** in $CDCl_3$ measured at 700.21 MHz. * Denotes residual grease.

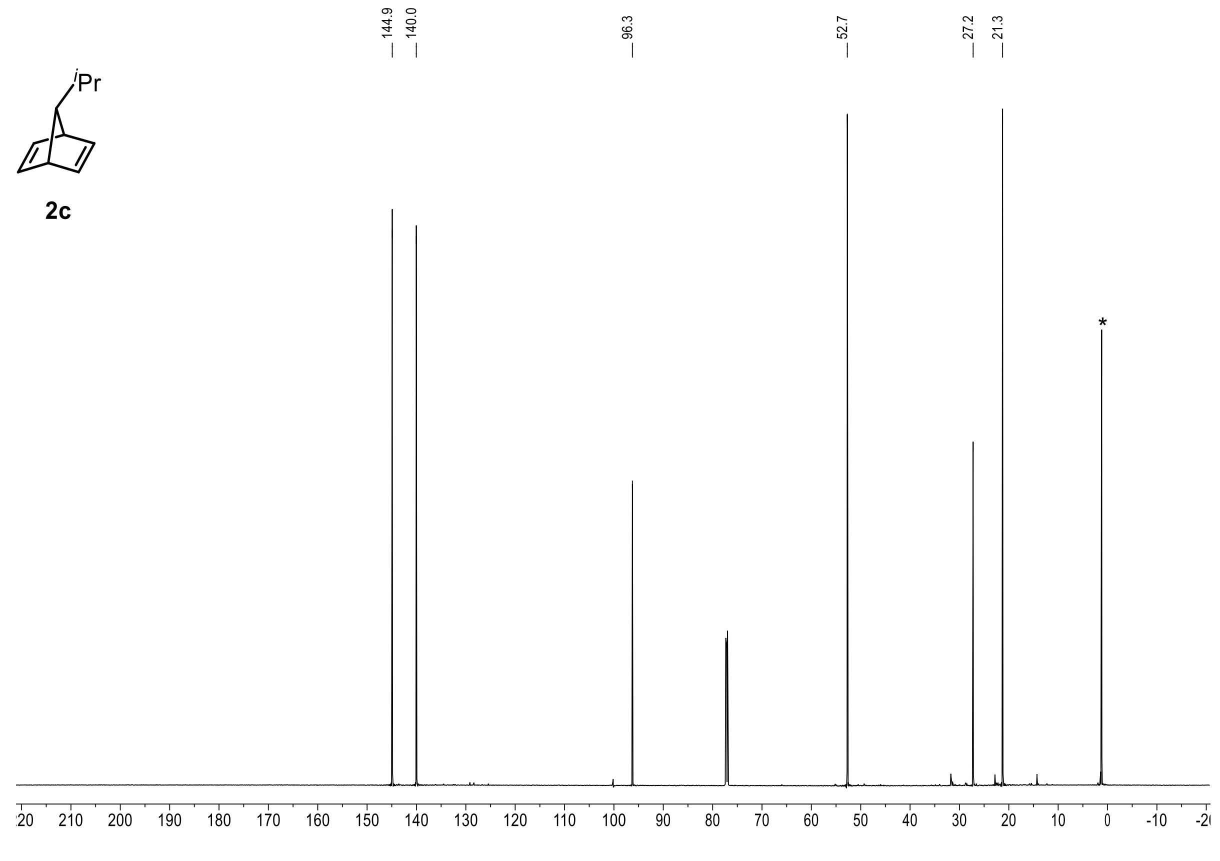

Figure S 56. ^{13}C NMR of **2c** in $CDCl_3$ measured at 176.08 MHz. * Denotes residual grease.

7-(phenyl)bicyclo[2.2.1]hepta-2,5-diene

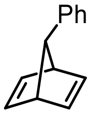

2d

2d was synthesized according to **GP-K** employing **2b** (1.97 g, 12.0 mmol, 1.00 equiv.), and PhMgBr (8.00 mL, 3.00 M, 2.00 equiv.). Vacuum distillation (15 mbar, 110 C) furnished **2d** (1.09 g, 6.48 mmol, 54%) as a colorless oil. The analytical data is in accordance with literature.[20]

$C_{13}H_{12}$ (168.24 $\frac{g}{mol}$)

b.p. (15 mbar): 110 °C.

^{1}H NMR(700.21 MHz, $CDCl_3$): δ = 7.26 (m, 2H), 7.17 (t, ^3J = 7.3 Hz, 1H), 7.08 (d, ^3J = 7.4 Hz, 2H), 6.96 (dd, ^3J = 1.9 Hz, ^3J = 1.9 Hz, 2H), 6.58 (dd, ^3J = 1.9 Hz, ^3J = 1.9 Hz, 2H), 3.87 (m, 2H), 3.83 (s, 1H).

^{13}C NMR(176.08 MHz, $CDCl_3$): δ = 144.4, 141.4, 139.9, 128.2, 127.7, 125.7, 87.7, 53.7.

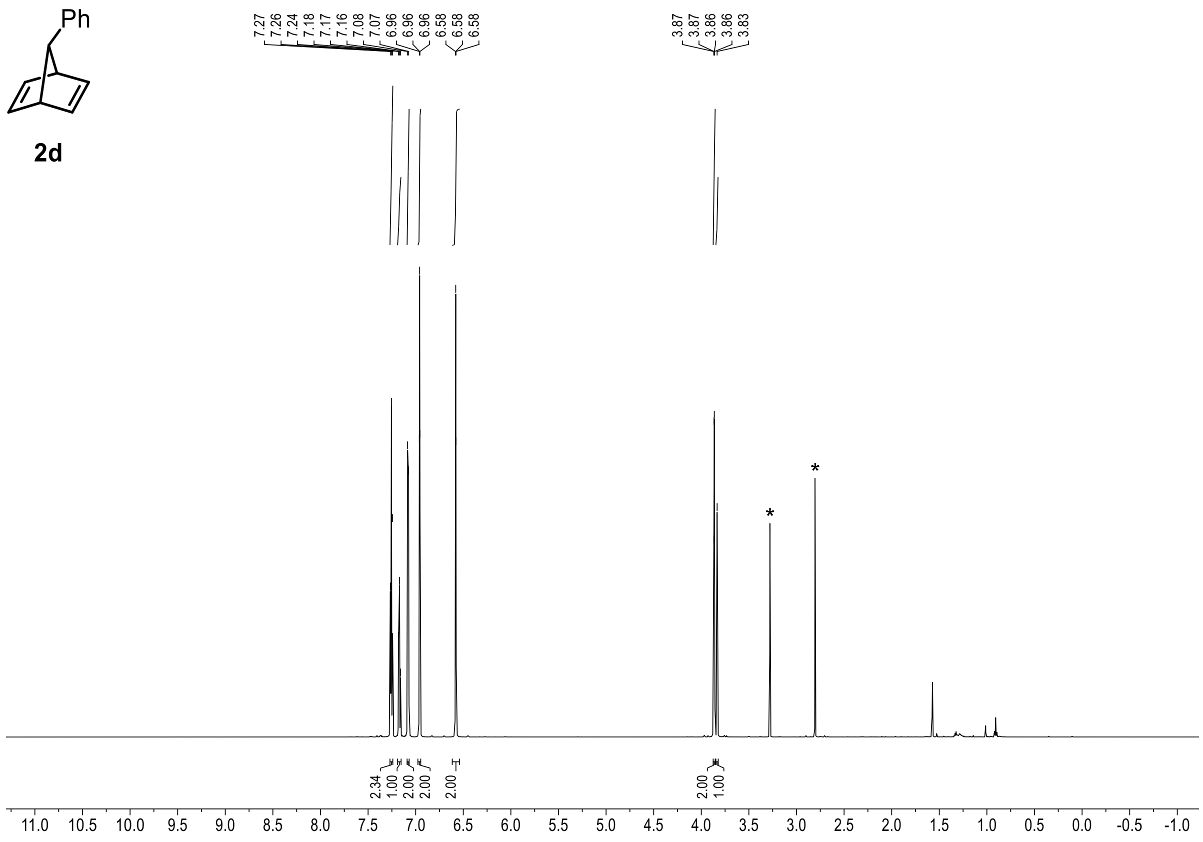

Figure S 57. ^{1}H NMR of **2d** in $CDCl_3$ measured at 700.21 MHz. * Denotes DMI.

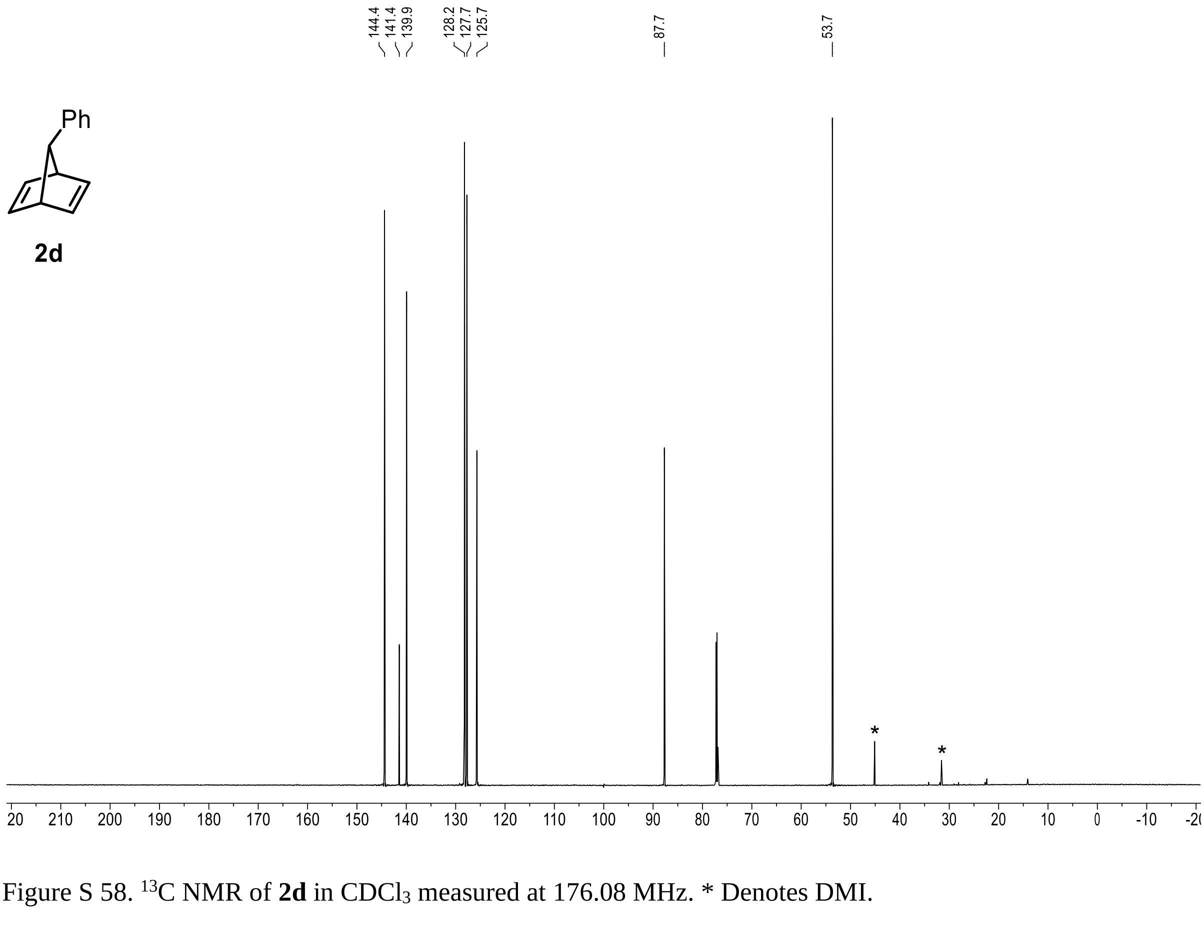

Figure S 58. ^{13}C NMR of **2d** in $CDCl_3$ measured at 176.08 MHz. * Denotes DMI.

2-hexylbicyclo[2.2.1]hepta-2,5-diene

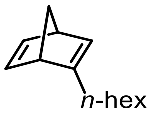

2e

2e was synthesized according to literature.[21] In a predried Schlenk RBF, a solution of NBD (2.03 mL, 20.0 mmol, 1.82 equiv.) in THF (50 mL) was added to a solution of KO^{t}Bu (1.23 g, 11.0 mmol, 1.00 equiv.) in THF (60 mL) at -78 °C. Subsequently, ^{n}BuLi (6.87 mL, 1.60 M, 1.00 equiv.) was added dropwise over 45 min. After complete addition the reaction was first allowed to slowly warm to -35 °C and then cooled down to -70 °C, before 1-bromohexane (2.00 mL, 14.3 mmol, 1.30 equiv.) was added over 2 min. After complete addition the reaction was stirred at -40 °C for 2 h, before stirring at 0 °C for further 2 h. The reaction was allowed to warm to rt and was quenched with $NH_4Cl_{(aq.)}$ (25 mL). The layers were separated, and the aqueous phase was extracted with DCM (3 × 10 mL). The combined organic phases were washed with H_2O (2 × 25 mL), dried over $MgSO_4$ and the solvent was removed *in vacuo*. **2e** was purified by flash chromatography (23 g SiO_2, *n*-hexane over 10 CV) followed by vacuum distillation (5 mbar, 65 °C, 105 °C oil bath) yielding a colorless oil (1.13 g, 6.41 mmol, 58%). The analytical data is in accordance with literature.[22]

$C_{13}H_{20}$ (176.30 $\frac{g}{mol}$)

b.p. (5 mbar): 65 °C.

R_f: 0.63 (*n*-hexane/EA = 80:20) [UV]

^{1}H NMR(400.16 MHz, $CDCl_3$): δ = 6.75 (m, 2H), 6.11 (m, 1H), 3.49 (s, 1H), 3.27 (s, 1H), 2.18 (m, 2H), 1.96 (m, 2H), 1.40 (m, 2H), 1.27 (m, 6H), 0.88 (t, ^2J = 6.8 Hz, 3H).

^{13}C NMR(176.08 MHz, $CDCl_3$): δ = 159.3, 144.0, 142.6, 133.2, 73.6, 53.7, 50.1, 31.9, 31.7, 29.2, 27.4, 22.8, 14.3.

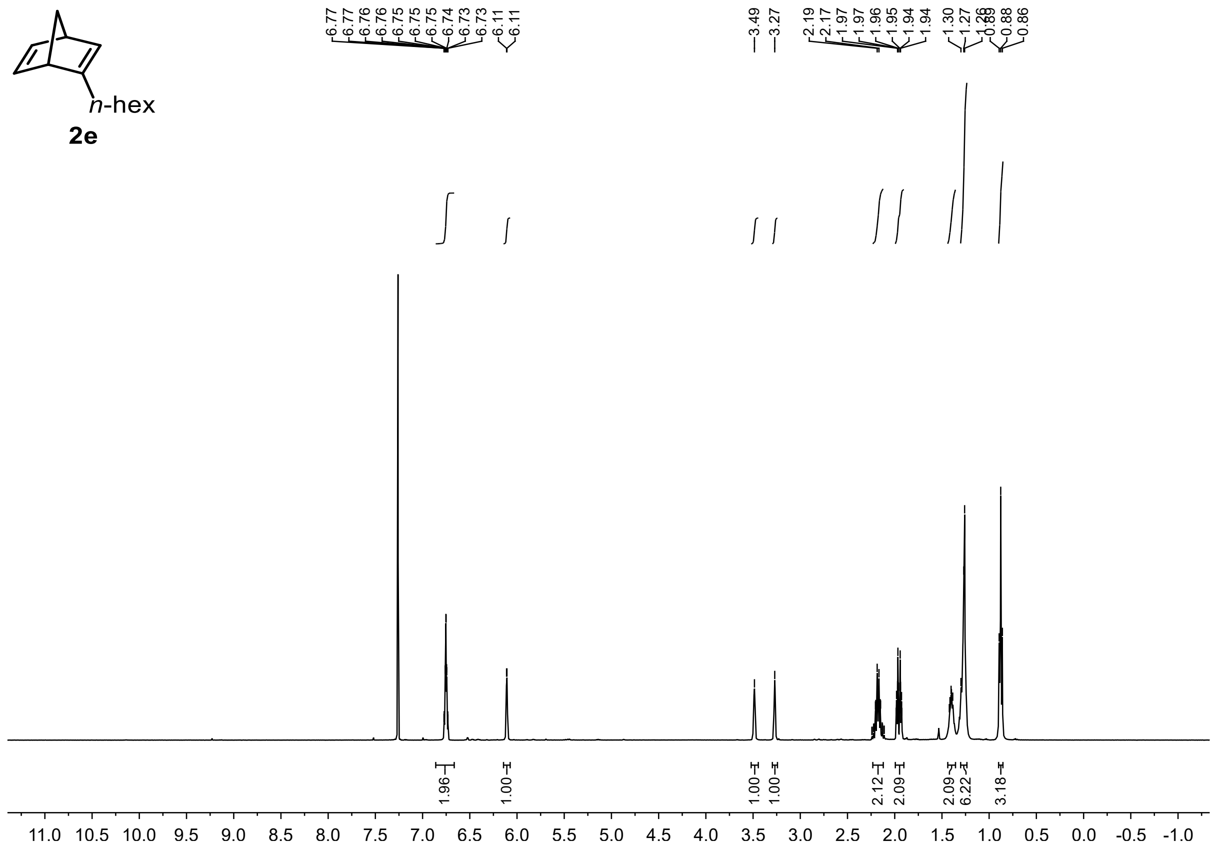

Figure S 59. ^{1}H NMR of **2e** in $CDCl_3$ measured at 400.16 MHz.

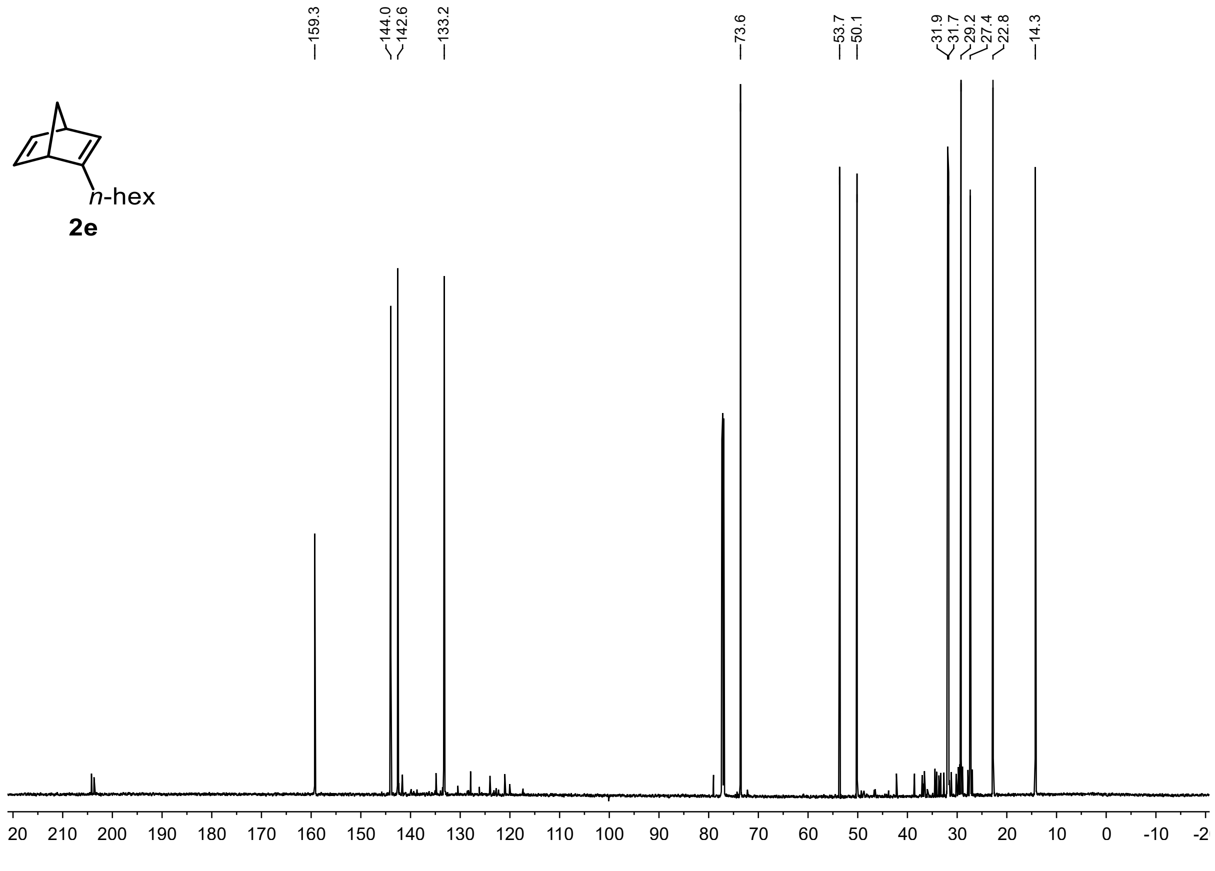

Figure S 60. ^{13}C NMR of **2e** in $CDCl_3$ measured at 176.08 MHz.

4.5 Catalytic Products

***rel*-(1*S*,2*S*,3a*R*,4*R*,10a*S*,11*R*)-2,3,3a,4,9,10a-hexahydro-1,2,4-(epimethanetriyl) benzo[f]azulen-10(1H)-one**

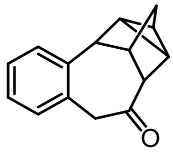

3aa

3aa was synthesized according to **GP-A** employing **1a** (118 mg, 1.00 mmol, 1.00 equiv.) and NBD (132 µL, 1.30 mmol, 1.30 equiv.) at 50 °C instead of 80 °C. Purification *via* flash chromatography (23 g SiO_2, gradient from 100:0 to 70:30 *n*-hexane/EA over 15 CV) afforded **3aa** (190 mg, 904 µmol, 90%, 34% *ee*, $[\alpha]_D^{20}$ +24 (*c* 1.0, $CHCl_3$)) as a pale yellow solid.

Additionally, **3aa** was synthesized in an upscaled reaction according to **GP-A** employing **1a** (1.18 g, 10.0 mmol, 1.00 equiv.) and NBD (1.32 mL, 1.30 mmol, 1.30 equiv.) at 50 °C instead of 80 °C. Hereby all parameters were scaled accordingly. Purification *via* flash chromatography (37 g SiO_2, gradient from 100:0 to 70:30 *n*-hexane/EA over 15 CV) afforded **3aa** (1.75 g, 8.31 mmol, 83%) as a pale yellow solid.

Alternatively, Ni(COD)$_2$ (10 mol%) and 11bS-N,N-bis[(1S)-1-(1-naphthalenyl)ethyl]dinaphtho[2,1-d:1',2'-f][1,3,2]dioxaphosphepin-4-amine (cas: 342813-26-7, 12 mol%) were added to a dried 50 mL Schlenk round bottom flask (RBF) in a glovebox. 20 mL of *n*-hexane were added, and the reaction was stirred for 5 min at rt. Subsequently, first NBD (132 µL, 1.30 mmol, 1.30 equiv.) and then **1a** (118 mg, 1.00 mmol, 1.00 equiv.) were added, after which the reaction was transferred to a preheated (70 °C) oil bath and stirred for 16 h. The workup was conducted according to **GP-A**. Purification *via* flash chromatography (23 g SiO_2, gradient from 100:0 to 70:30 *n*-hexane/EA over 15 CV) afforded **3aa** (87.9 mg, 418 µmol, 42%, 71% *ee*, $[\alpha]_D^{20}$ -60 (*c* 1.0, $CHCl_3$)) as a pale yellow solid.

$C_{15}H_{14}O$ (210.28 $\frac{g}{mol}$)

mp: 65.5 °C.

R$_f$: 0.45 (*n*-hexane/EA = 80:20) [anisaldehyde]

^{1}H NMR(400.16 MHz, $CDCl_3$): δ = 7.18 (m, 1H, H-10), 7.17 (m, 1H, H-11), 7.16 (m, 1H, H-12), 7.06 (m, 1H, H-13), 4.50 (dd, ^2J = 12.3 Hz, ^4J = 0.6 Hz, 1H, H-15b), 3.36 (dd,

${}^2J$ = 12.3 Hz, ${}^4J$ = 1.0 Hz, 1H, H-15a), 2.91 (s, 1H, H-7), 2.65 (m, 1H, H-2), 2.29 (m, 1H, H-8), 1.72 (ddm, ${}^3J$ = 5.0 Hz, ${}^3J$ = 5.0 Hz, 1H, H-6), 1.66 (dm, ${}^2J$ = 10.8 Hz, 1H, H-5a), 1.52 (dm, ${}^2J$ = 10.8 Hz, 1H, H-5b), 1.51 (m, 1H, H-4), 1.49 (m, 1H, H-3).

^{13}C NMR(100.62 MHz, $CDCl_3$): δ = 209.5 (C-1), 139.7 (C-9), 131.8 (C-14), 131.5 (C-10), 130.4 (C-13), 127.6 (C-12), 127.1 (C-11), 59.1 (C-2), 52.9 (C-7), 49.1 (C-15), 42.1 (C-8), 36.3 (C-5), 17.8 (C-6), 14.9 (C-4), 13.2 (C-3).

HRMS (ESI-TOF) m/z: $[M+H]^+$ Calcd for $C_{15}H_{14}OH$ 211.1117; Found 211.1119.

IR (ATR, ṽ): 1687 cm^{-1} (s, *CO*).

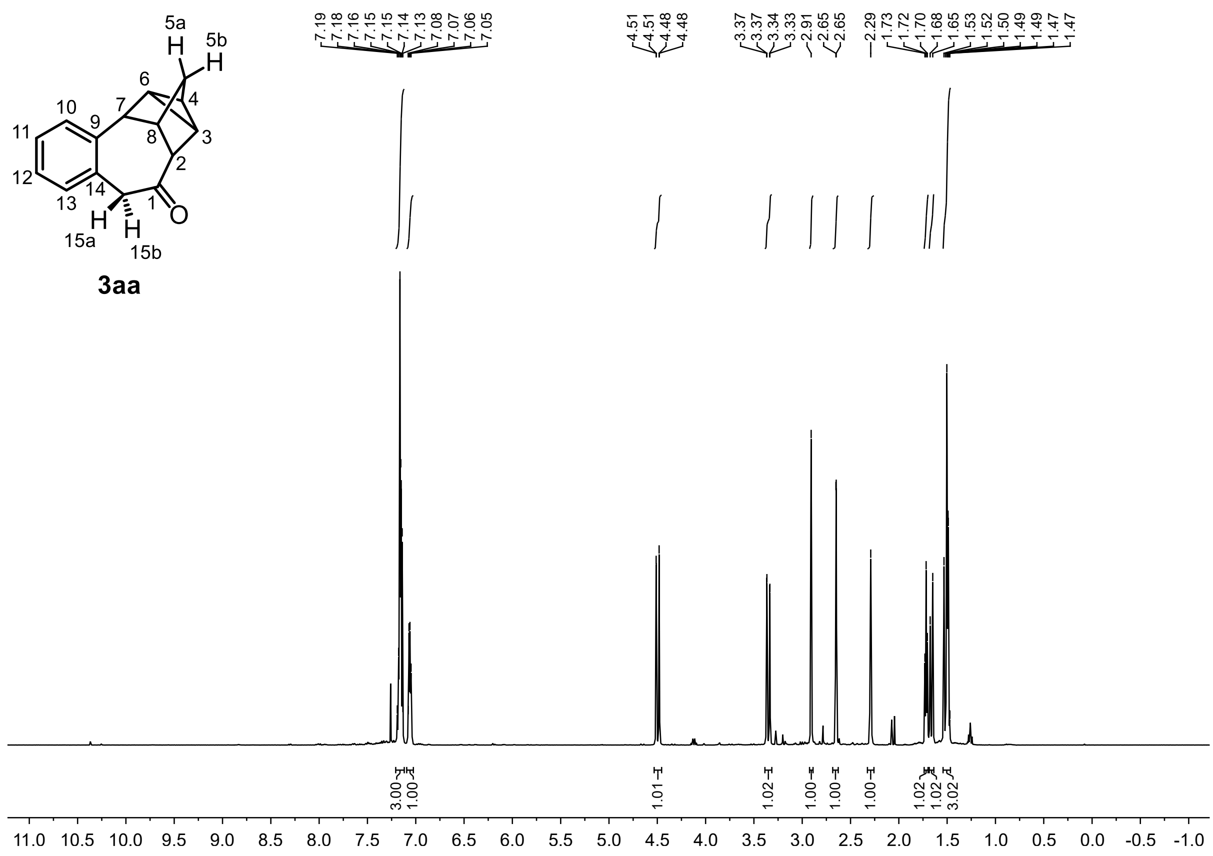

Figure S 61. ^{1}H NMR of **3aa** in $CDCl_3$ measured at 400.16 MHz.

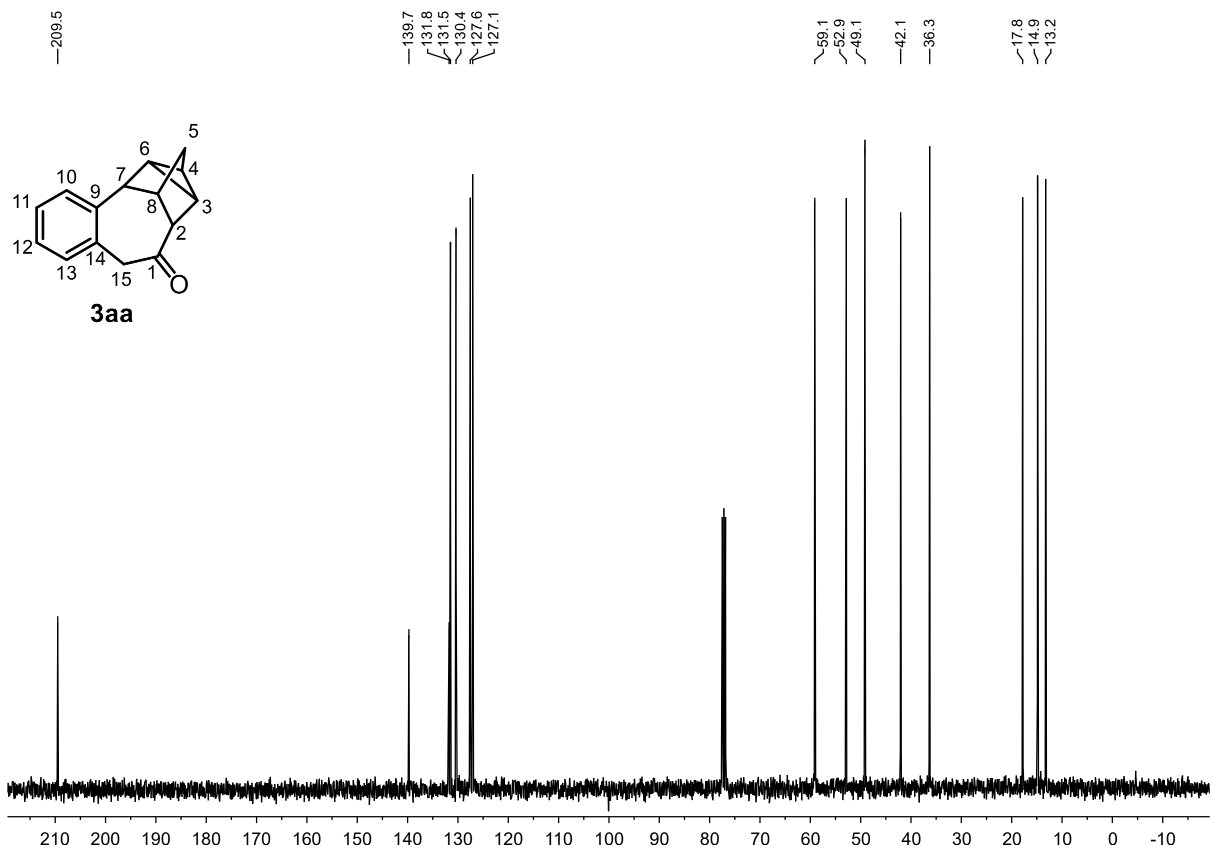

Figure S 62. ^{13}C NMR of **3aa** in $CDCl_3$ measured at 100.63 MHz.

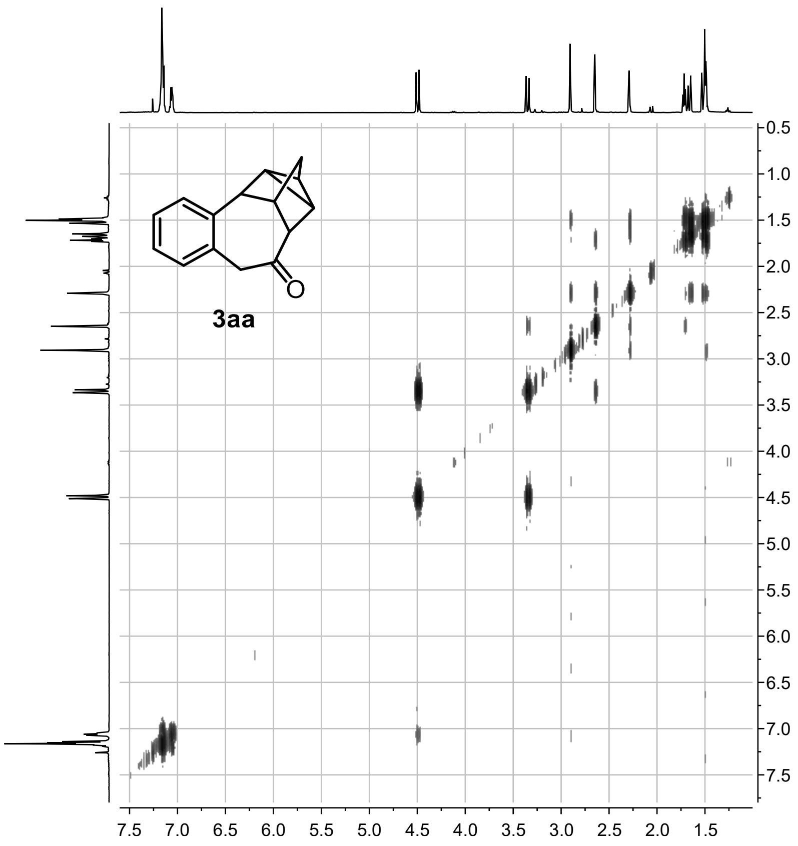

Figure S 63. $^{1}H,^{1}H$-COSY of **3aa** in $CDCl_3$ measured at 400.16 MHz.

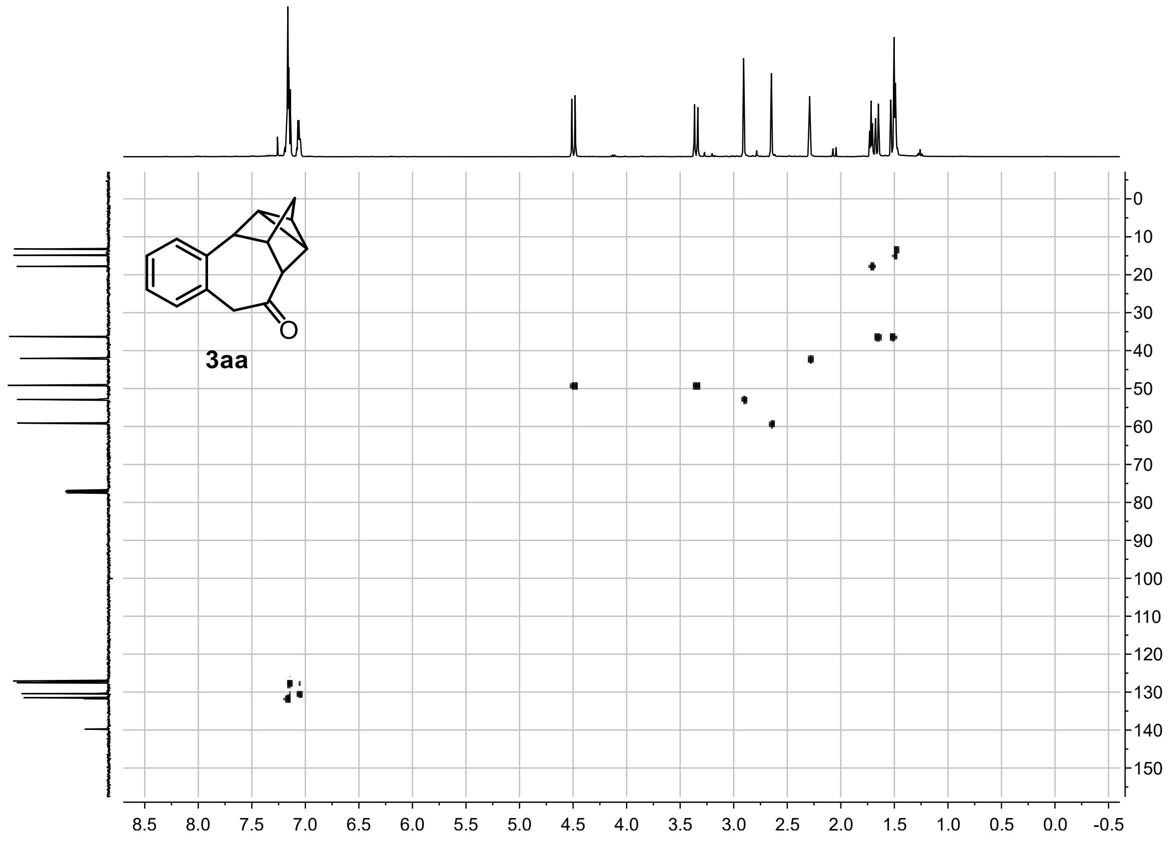

Figure S 64. $^{1}H,^{13}C$-HSQC of **3aa** in $CDCl_3$ measured at ^{1}H: 400.16 MHz; ^{13}C: 100.63 MHz.

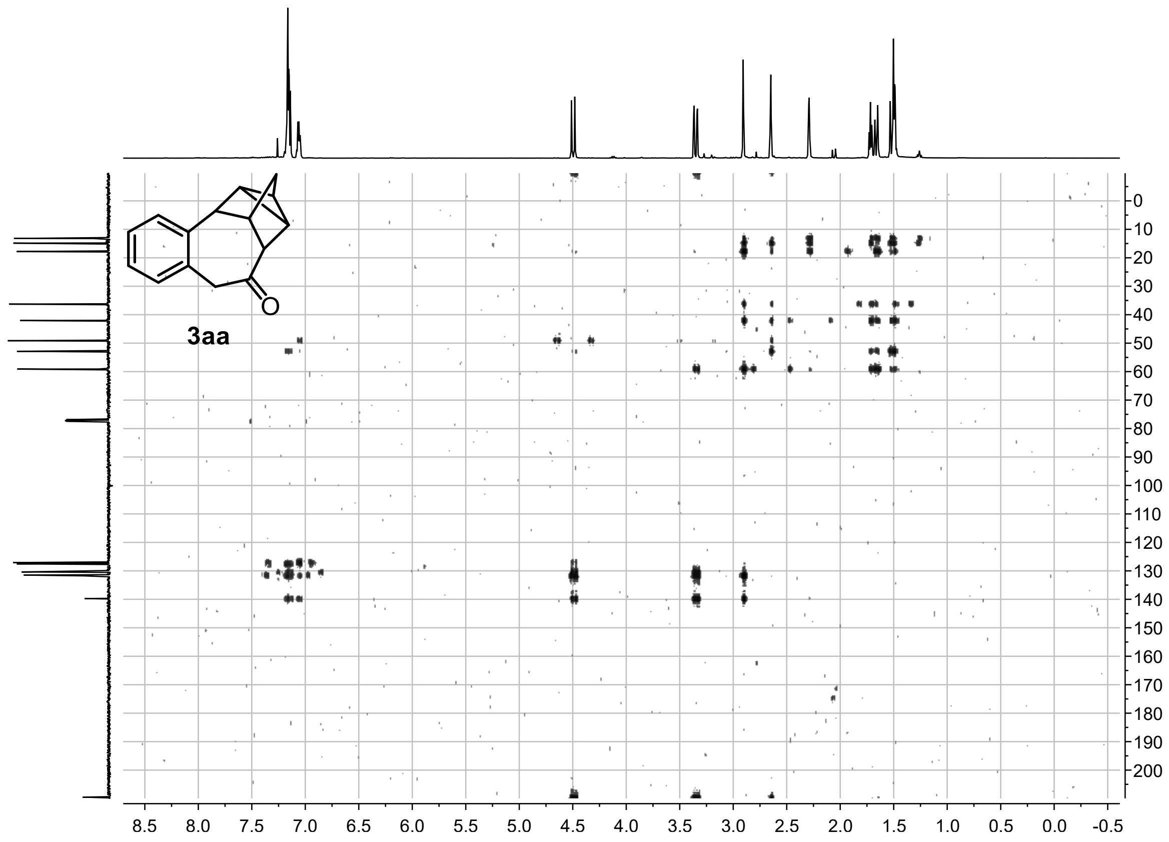

Figure S 65. $^{1}H,^{13}C$-HMBC of **3aa** in $CDCl_3$ measured at ^{1}H: 400.16 MHz; ^{13}C: 100.63 MHz.

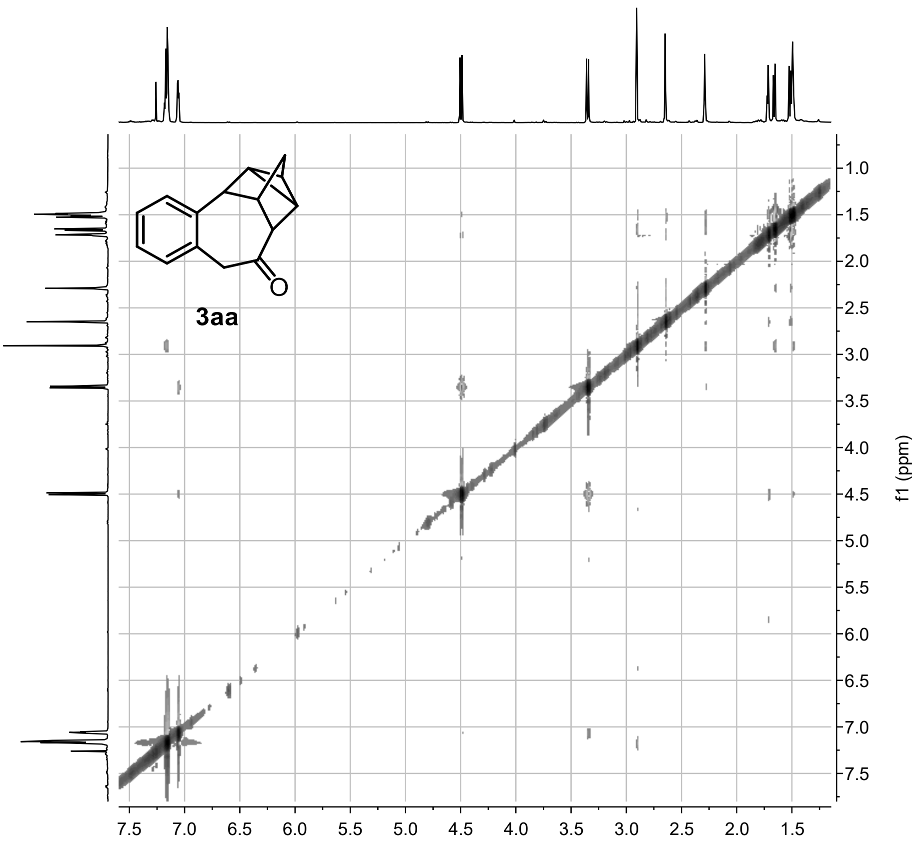

Figure S 66. $^{1}H,^{1}H$-NOESY of **3aa** in $CDCl_3$ measured at 700.21 MHz.

***rel*-(1*S*,2*S*,3a*R*,4*R*,12a*S*,13*R*)-2,3,3a,4,11,12a-hexahydro-1,2,4-(epimethanetriyl) naphtho[2,3-f]azulen-12(1H)-one**

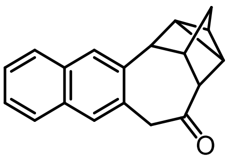

3ba

3ba was synthesized according to **GP-A** employing **1b** (168 mg, 1.00 mmol, 1.00 equiv.) and NBD (132 µL, 1.30 mmol, 1.30 equiv.). Purification *via* flash chromatography (23 g SiO_2, gradient from 98:02 to 80:10 *n*-hexane/EA over 15 CV) afforded **3ba** (209 mg, 802 µmol, 80%, 25% *ee*, $[\alpha]_D^{20}$ -2 (*c* 1.0, $CHCl_3$)) as a pale brown solid.

$C_{19}H_{16}O$ (260.34 $\frac{g}{mol}$)

mp: 144.3 °C.

R_f: 0.57 (*n*-hexane/EA = 80:20) [UV]

^{1}H-NMR (700 MHz, $CDCl_3$) δ = 7.73 (m, 2H, H-12, H-15), 7.67 (s, 1H, H-10), 7.54 (s, 1H, H-17), 7.42 (m, 2H, H-13, H-14), 4.73 (m, 1H, H-19b), 3.48 (m, 1H, H-19a), 3.12 (s, 1H, H-7), 2.69 (s, 1H, H-2), 2.27 (s, 1H, H-8), 1.78 (m, 1H, H-3), 1.73 (m, 1H, H-5a), 1.59 (m, 1H, H-4), 1.56 (m, 2H, H-6, H-5b).

^{13}C-NMR (176 MHz, $CDCl_3$) δ = 210.0 (C-1), 138.1 (C-8), 132.9 (C-11), 132.5 (C-16), 130.2 (C-18), 130.0 (C-10), 128.7 (C-17), 127.2 (C-12), 127.1 (C-15), 126.1 (C-14), 126.1 (C-13), 59.1 (C-2), 53.4 (C-7), 49.3 (C-19), 42.5 (C-8), 36.6 (C-5), 18.3 (C-3), 15.0 (C-4), 13.6 (C-6).

HRMS (ESI-TOF) m/z: $[M+H]^+$ Calcd for $C_{19}H_{16}OH$ 261.1274; Found 261.1273.

IR (ATR, $\tilde{\nu}$): 1687 cm^{-1} (s, *CO*).

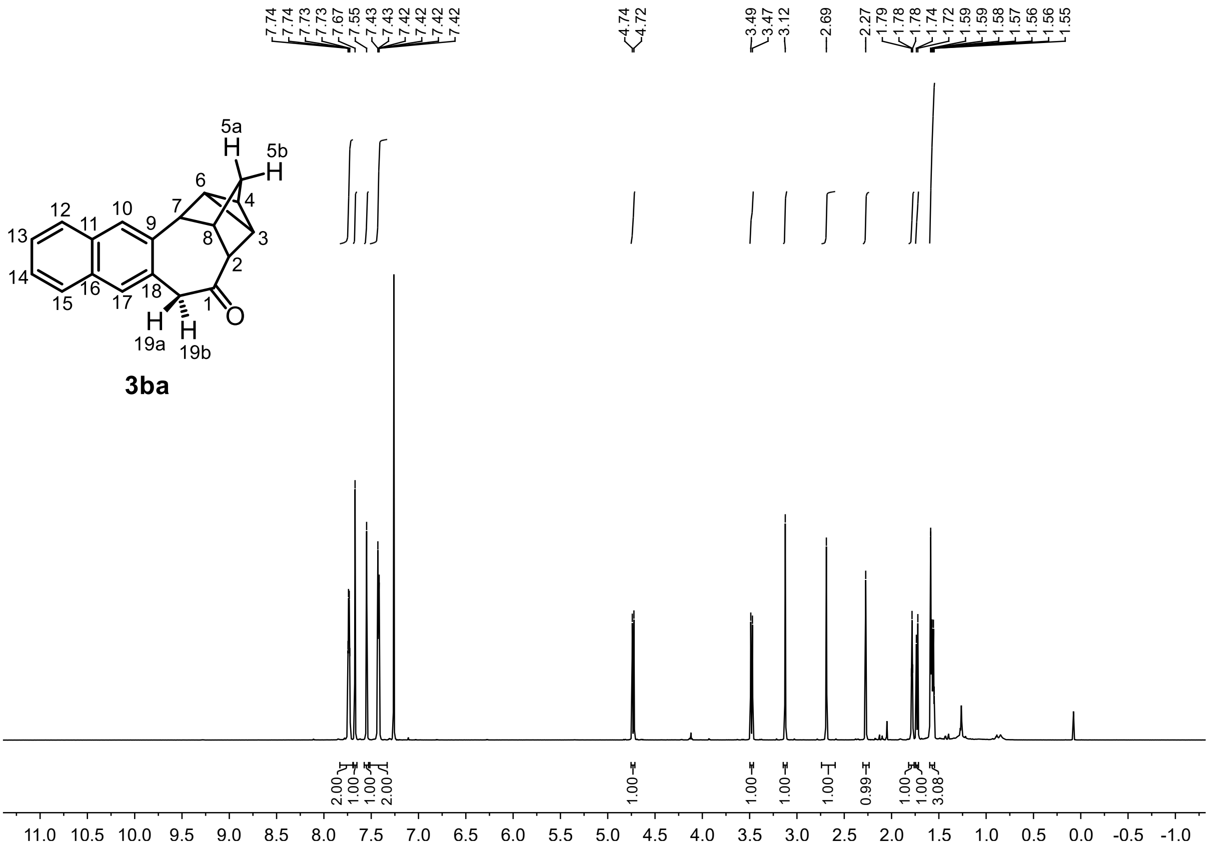

Figure S 67. ^{1}H NMR of **3ba** in $CDCl_3$ measured at 700.21 MHz.

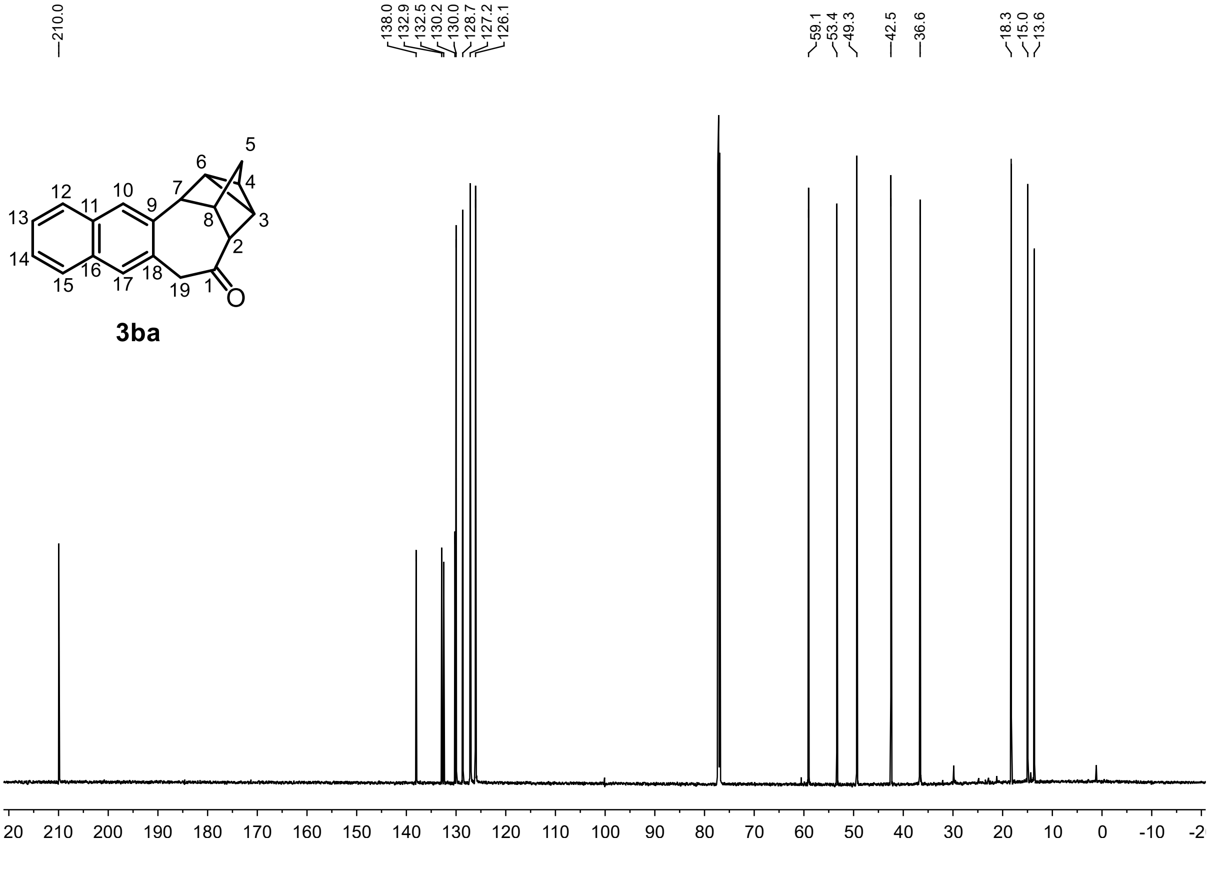

Figure S 68. ^{13}C NMR of **3ba** in $CDCl_3$ measured at 176.08 MHz.

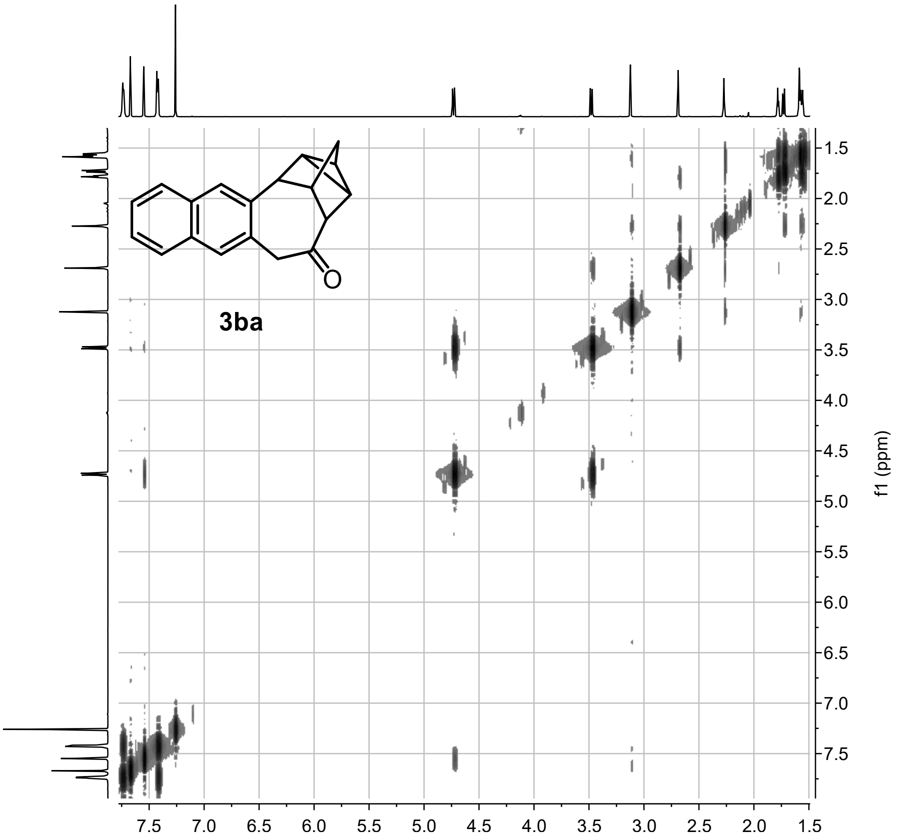

Figure S 69. ^{1}H,^{1}H-COSY of **3ba** in $CDCl_3$ measured at 700.21 MHz.

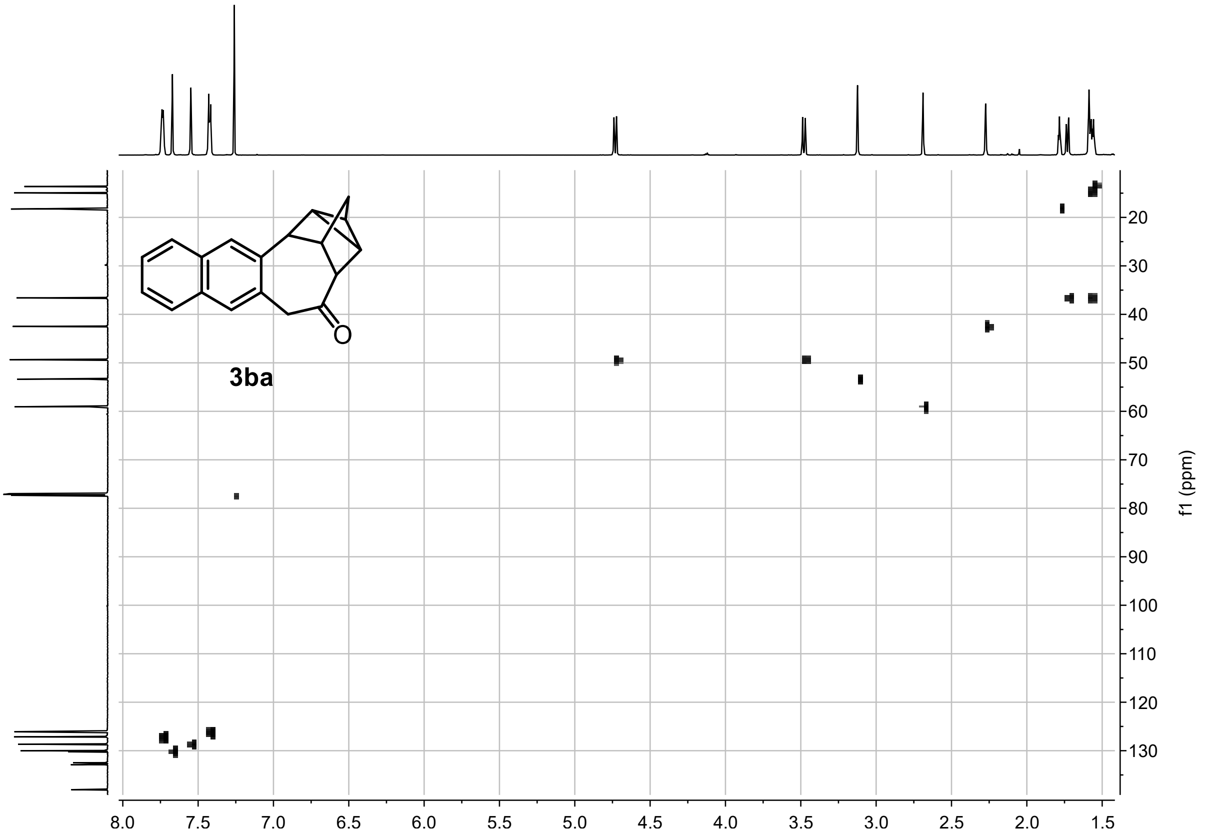

Figure S 70. ^{1}H,^{13}C-HSQC of **3ba** in $CDCl_3$ measured at ^{1}H: 700.21 MHz; ^{13}C: 176.08 MHz.

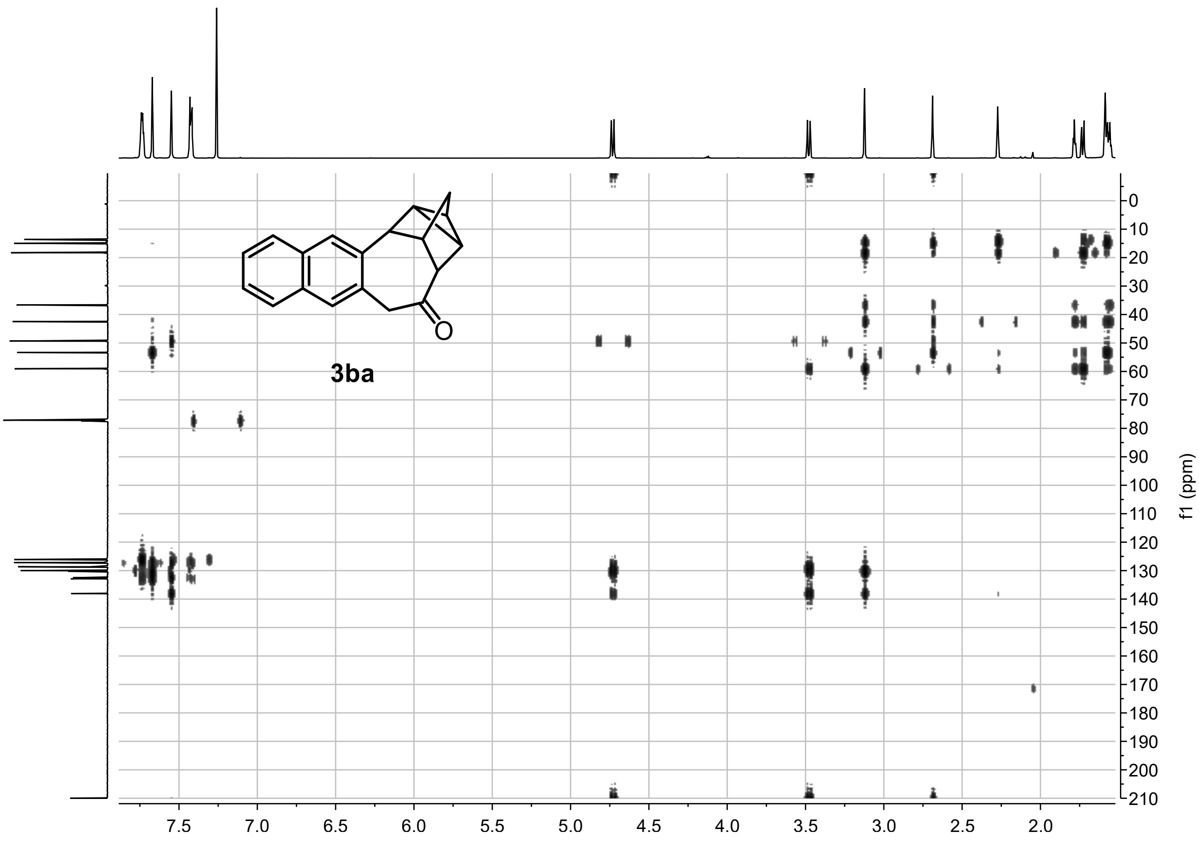

Figure S 71. $^{1}H,^{13}C$-HMBC of **3ba** in $CDCl_3$ measured at ^{1}H: 700.21 MHz; ^{13}C: 176.08 MHz.

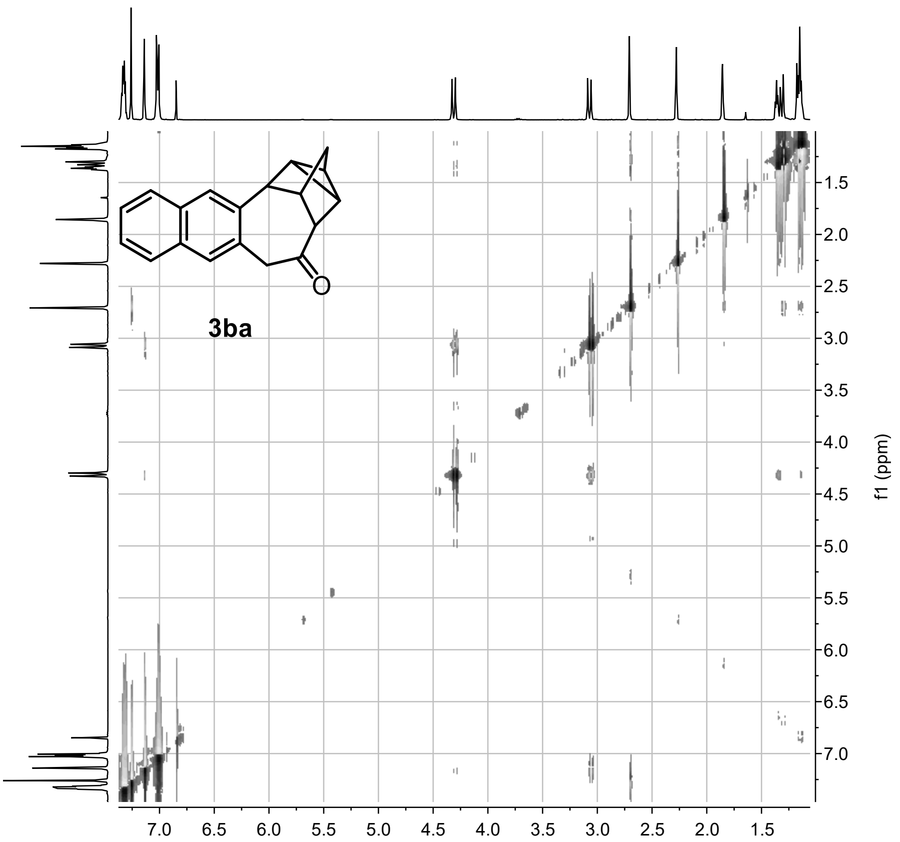

Figure S 72. $^{1}H,^{1}H$-NOESY of **3ba** in $CDCl_3$ measured at 400.16 MHz.

***rel*-(1*S*,2*S*,3a*R*,4*R*,10a*S*,11*R*)-8-methyl-2,3,3a,4,9,10a-hexahydro-1,2,4-(epimethanetriyl) benzo[f]azulen-10(1H)-one**

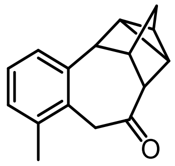

3ca

3ca was synthesized according to **GP-A** employing **1c** (132 mg, 1.00 mmol, 1.00 equiv.) and NBD (132 µL, 1.30 mmol, 1.30 equiv.). Purification *via* flash chromatography (23 g SiO_2, gradient from 98:02 to 75:25 *n*-hexane/EA over 20 CV) afforded **3ca** (174 mg, 774 µmol, 77%, 32% *ee*, $[\alpha]_D^{20}$ +13 (*c* 1.0, $CHCl_3$)) as a colorless oil.

$C_{16}H_{16}O$ (224.30 $\frac{g}{mol}$)

R$_f$: 0.68 (*n*-hexane/EA = 80:20) [anisaldehyde]

^{1}H NMR(400.16 MHz, $CDCl_3$): δ = 7.07 (m, 1H, H-12), 7.03 (m, 1H, H-10), 7.03 (m, 1H, H-11), 4.33 (d, ^2J = 12.4 Hz, 1H, H-16b), 3.64 (dd, ^2J = 12.4 Hz, ^4J = 0.8 Hz, 1H, H-16a), 2.91 (s, 1H, H-7), 2.64 (m, 1H, H-2), 2,37 (s, 3H, H-14), 2.26 (m, 1H, H-8), 1.74 (ddm, ^3J = 5.1 Hz, ^3J = 5.0 Hz, 1H, H-6), 1.65 (dm, ^2J = 11.0 Hz, 1H, H-5a), 1.52 (dm, ^2J = 11.0 Hz, 1H, H-5b), 1.50 (m, 1H, H-4), 1.49 (m, 1H, H-3).

^{13}C NMR(100.62 MHz, $CDCl_3$): δ = 210.3 (C-1), 140.1 (C-9), 136.3 (C-15), 130.8 (C-13), 129.8, 129.7, 126.5, 59.1 (C-2), 53.4 (C-7), 42.8 (C-16), 42.0 (C-8), 36.2 (C-5), 21.4 (C-14), 18.1 (C-6), 14.5 (C-3), 13.5 (C-4).

HRMS (ESI-TOF) m/z: $[M+H]^+$ Calcd for $C_{16}H_{16}OH$ 225.1274; Found 225.1276.

IR (ATR, ṽ): 1693 cm^{-1} (s, *CO*).

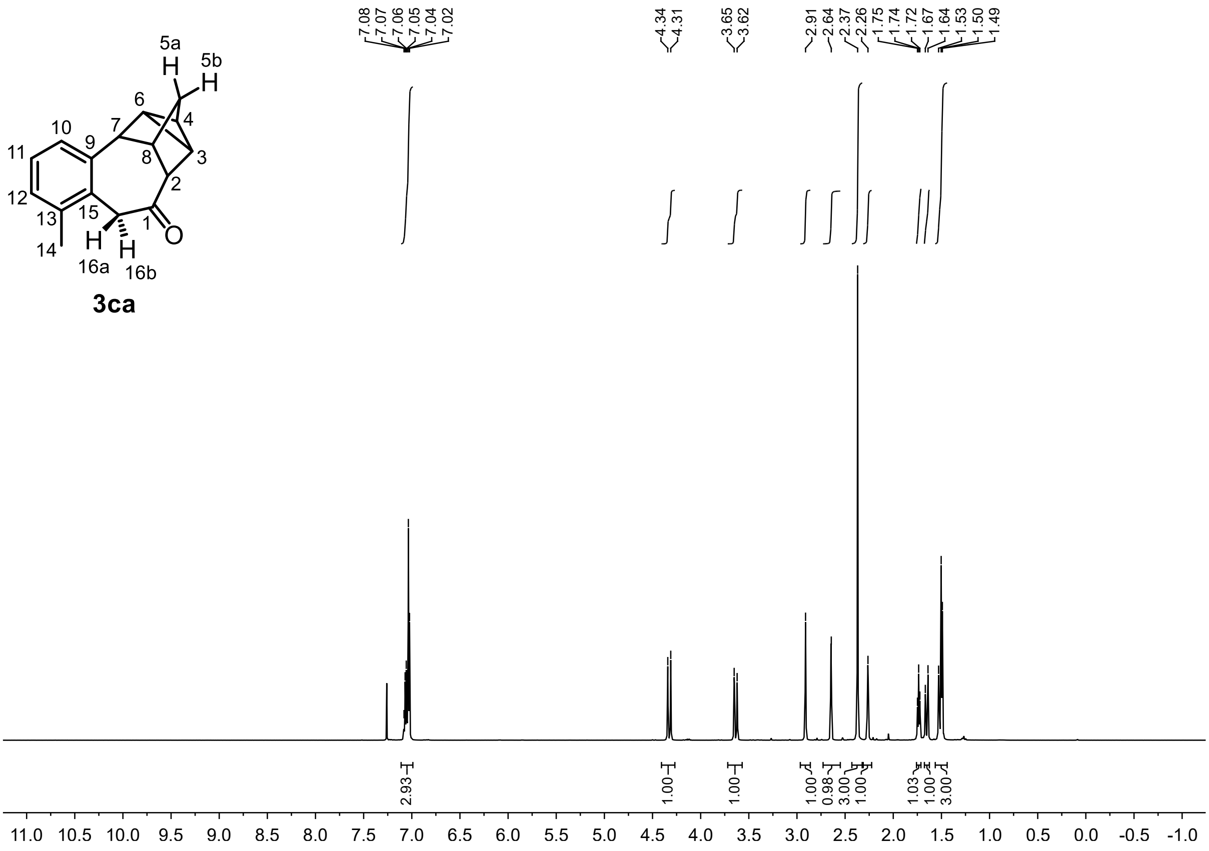

Figure S 73. ^{1}H NMR of **3ca** in $CDCl_3$ measured at 400.16 MHz.

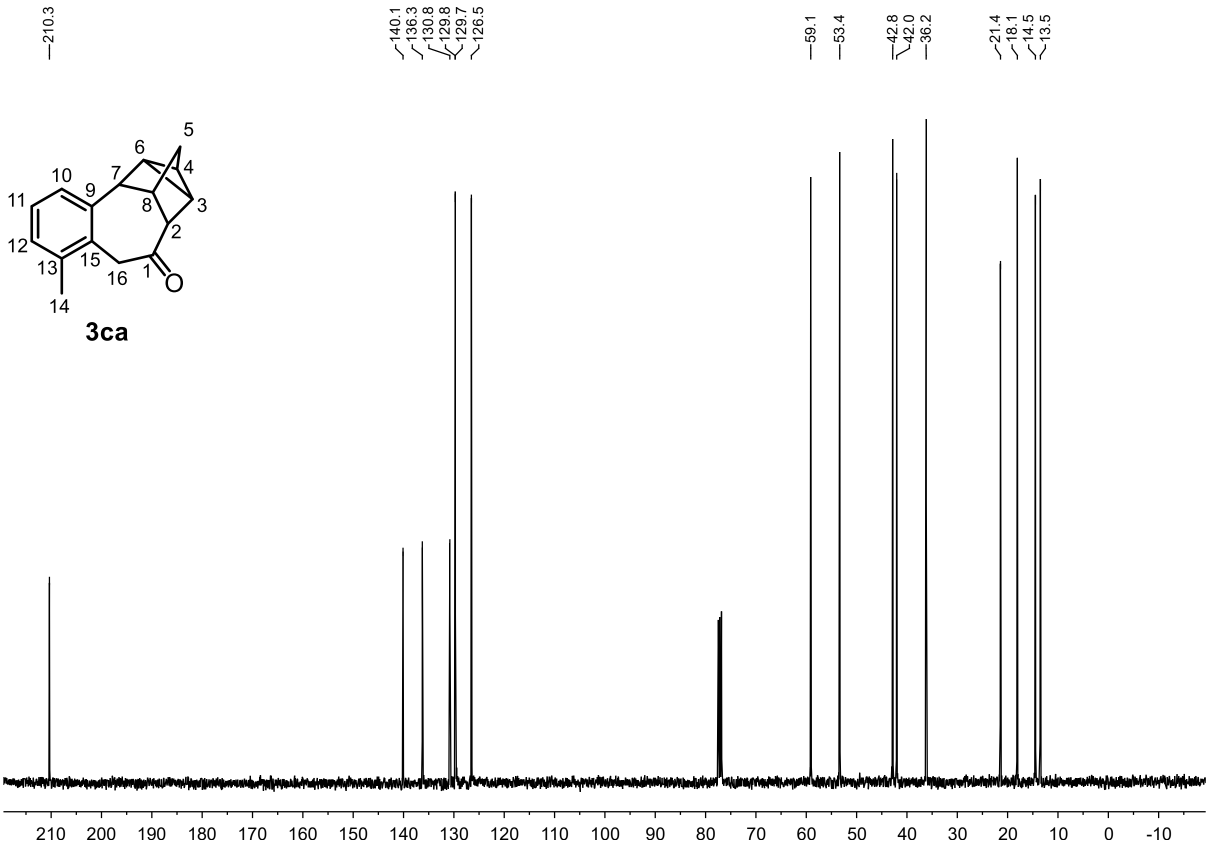

Figure S 74: ^{13}C NMR of **3ca** in $CDCl_3$ measured at 100.63 MHz.

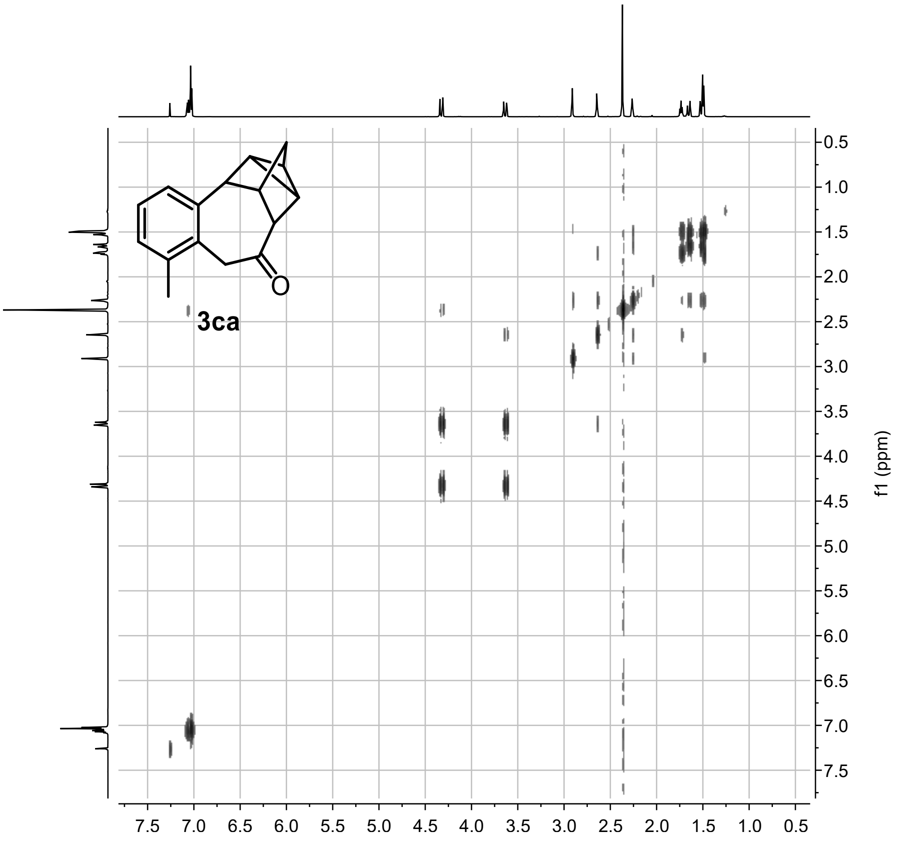

Figure S 75. $^{1}H,^{1}H$-COSY of **3ca** in $CDCl_3$ measured at 400.16 MHz.

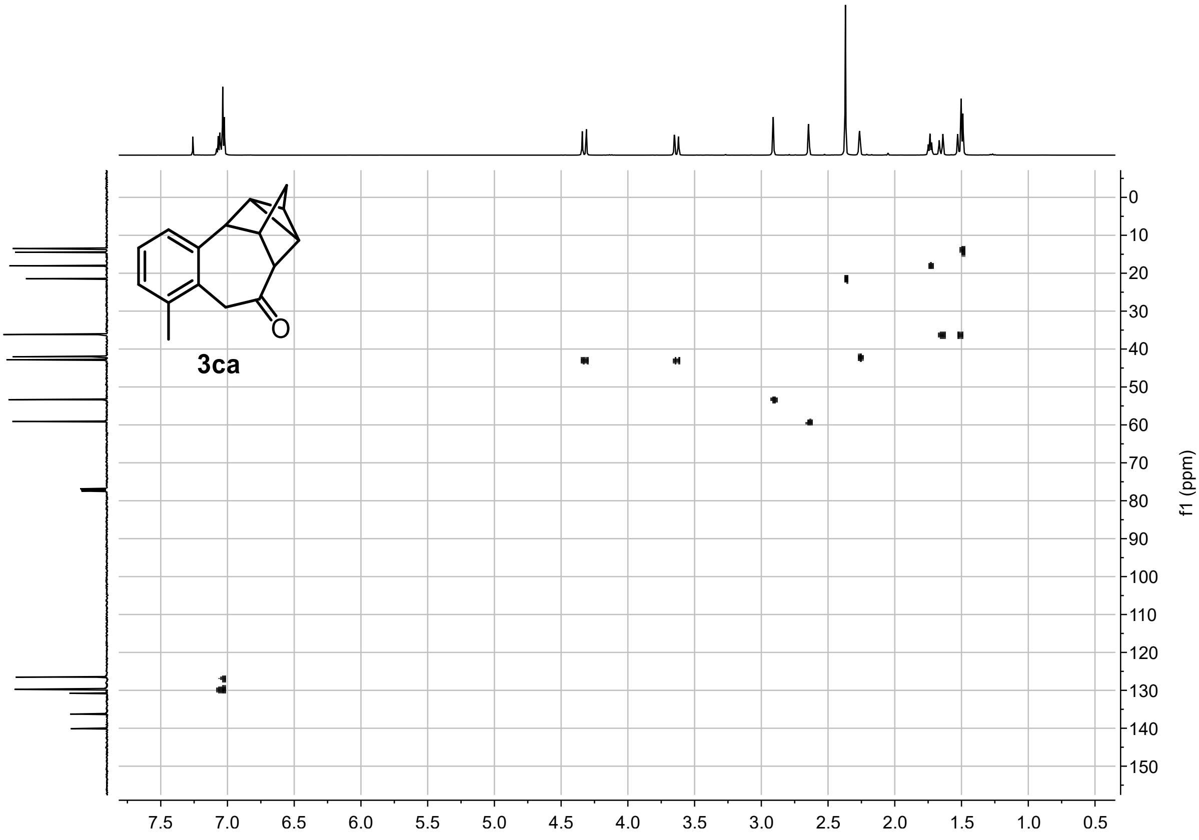

Figure S 76. $^{1}H,^{13}C$-HSQC of **3ca** in $CDCl_3$ measured at ^{1}H: 400.16 MHz; ^{13}C: 100.63 MHz.

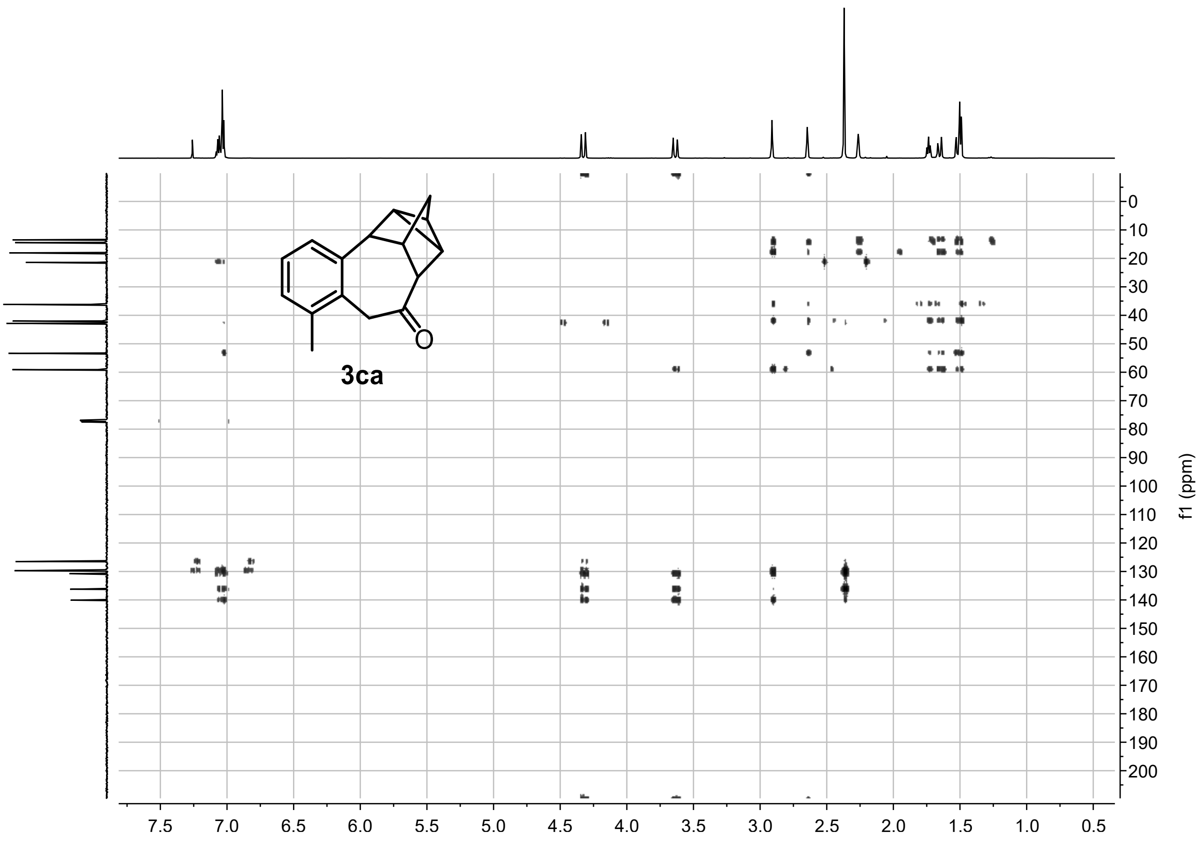

Figure S 77. $^{1}H,^{13}C$-HMBC of **3ca** in $CDCl_3$ measured at ^{1}H: 400.16 MHz; ^{13}C: 100.63 MHz.

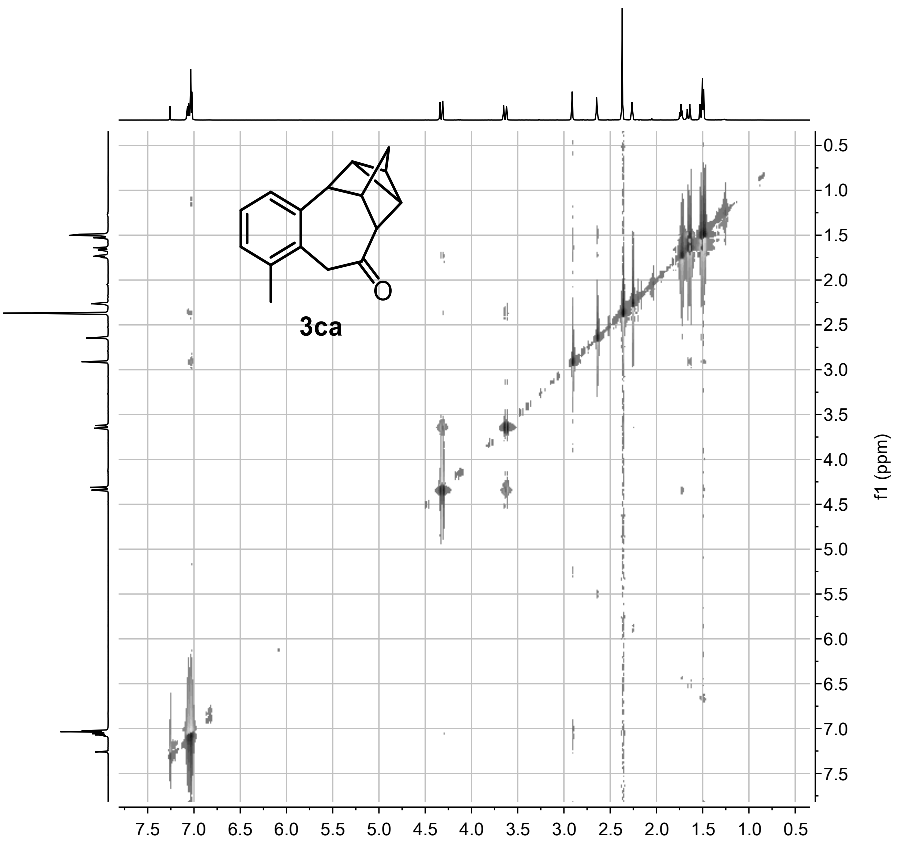

Figure S 78. $^{1}H,^{1}H$-NOESY of **3ca** in $CDCl_3$ measured at 400.16 MHz.

***rel*-7-methoxy-(1*S*,2*S*,3a*R*,4*R*,10a*S*,11*R*)-2,3,3a,4,9,10a-hexahydro-1,2,4-(epimethanetriyl)benzo[f]azulen-10(1H)-one**

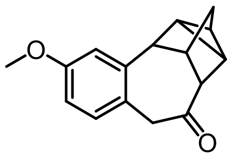

3da

3da was synthesized according to **GP-A** employing **1d** (148 mg, 1.00 mmol, 1.00 equiv.) and NBD (132 µL, 1.30 mmol, 1.30 equiv.) at 150 °C instead of 80 °C. Purification *via* flash chromatography (23 g SiO_2, gradient from 100:0 to 65:35 *n*-hexane/EA over 20 CV) afforded **3da** (179 mg, 746 µmol, 75%, 20% *ee*, $[\alpha]_D^{20}$ +10 (*c* 1.0, $CHCl_3$)) as a colorless solid.

$C_{16}H_{16}O_2$ (240.30 $\frac{g}{mol}$)

mp: 97.3 °C.

R_f: 0.51 (*n*-hexane/EA = 80:20) [anisaldehyde]

^{1}H NMR(400.16 MHz, $CDCl_3$): δ = 6.97 (m, 1H, H-14), 6.74 (m, 1H, H-10), 6.69 (m, 1H, H-13), 4.38 (dd, ^2J = 12.3 Hz, ^4J = 0.5 Hz, 1H, H-16b), 3.78 (s, 3H, H-12) 3.30 (dd, ^2J = 12.3 Hz, ^4J = 1.0 Hz, 1H, H-16a), 2.84 (s, 1H, H-7), 2.62 (m, 1H, H-2), 2.30 (m, 1H, H-8), 1.71 (ddm, ^3J = 5.1 Hz, ^3J = 5.0 Hz, 1H, H-6), 1.65 (dm, ^2J = 10.7 Hz, 1H, H-5a), 1.51 (dm, ^2J = 10.7 Hz, 1H, H-5b), 1.50 (m, 1H, H-3), 1.48 (m, 1H, H-4).

^{13}C NMR(100.62 MHz, $CDCl_3$): δ = 209.8 (C-1), 158.5 (C-11), 141.1 (C-15), 131.4 (C-14), 123.8 (C-9), 117.4 (C-10), 112.2 (C-13), 58.9 (C-2), 55.4 (C-12), 53.1 (C-7), 48.3 (C-16), 42.0 (C-8), 36.3 (C-5), 17.8 (C-6), 15.0 (C-3), 13.2 (C-4).

HRMS (ESI-TOF) m/z: $[M+H]^+$ Calcd for $C_{16}H_{16}O_2H$ 241.1223; Found 241.1226.

IR (ATR, ṽ): 1694 cm^{-1} (s, *CO*).

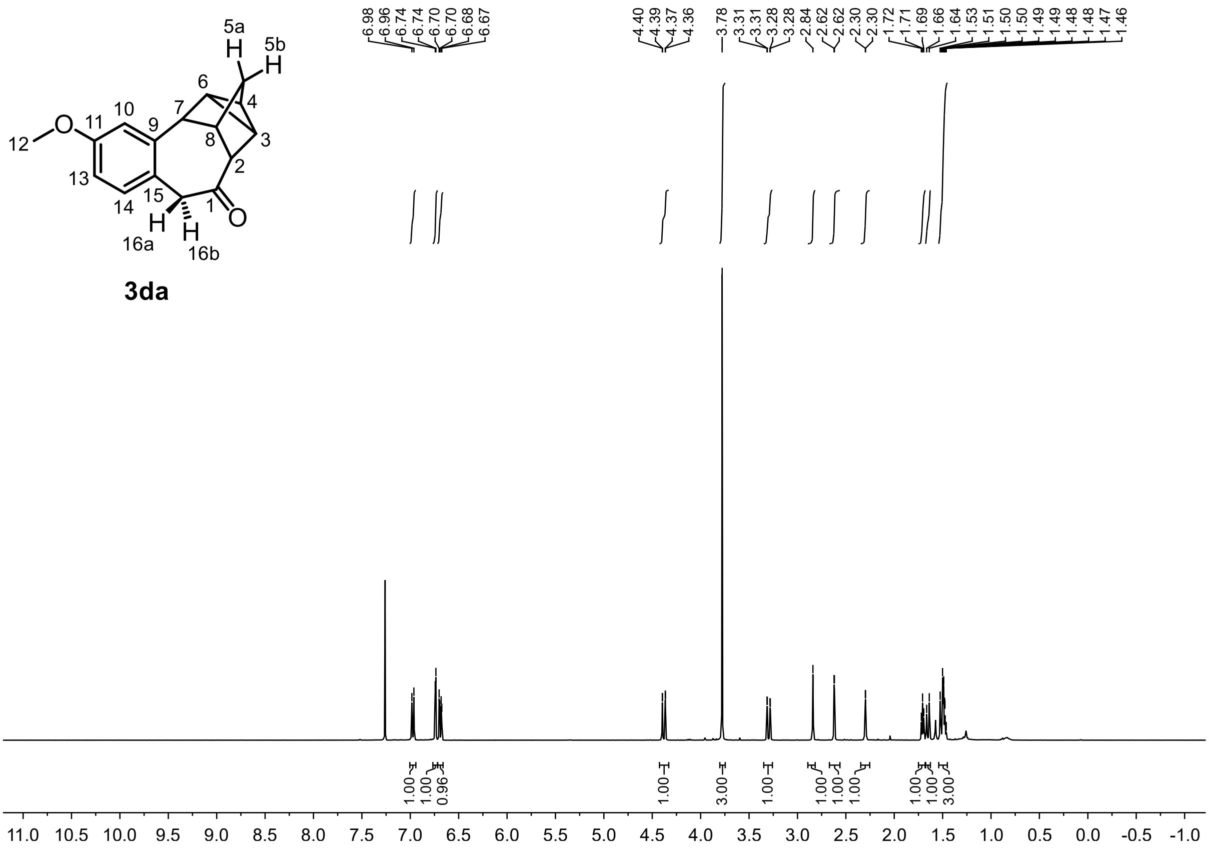

Figure S 79. ^{1}H NMR of **3da** in $CDCl_3$ measured at 400.16 MHz.

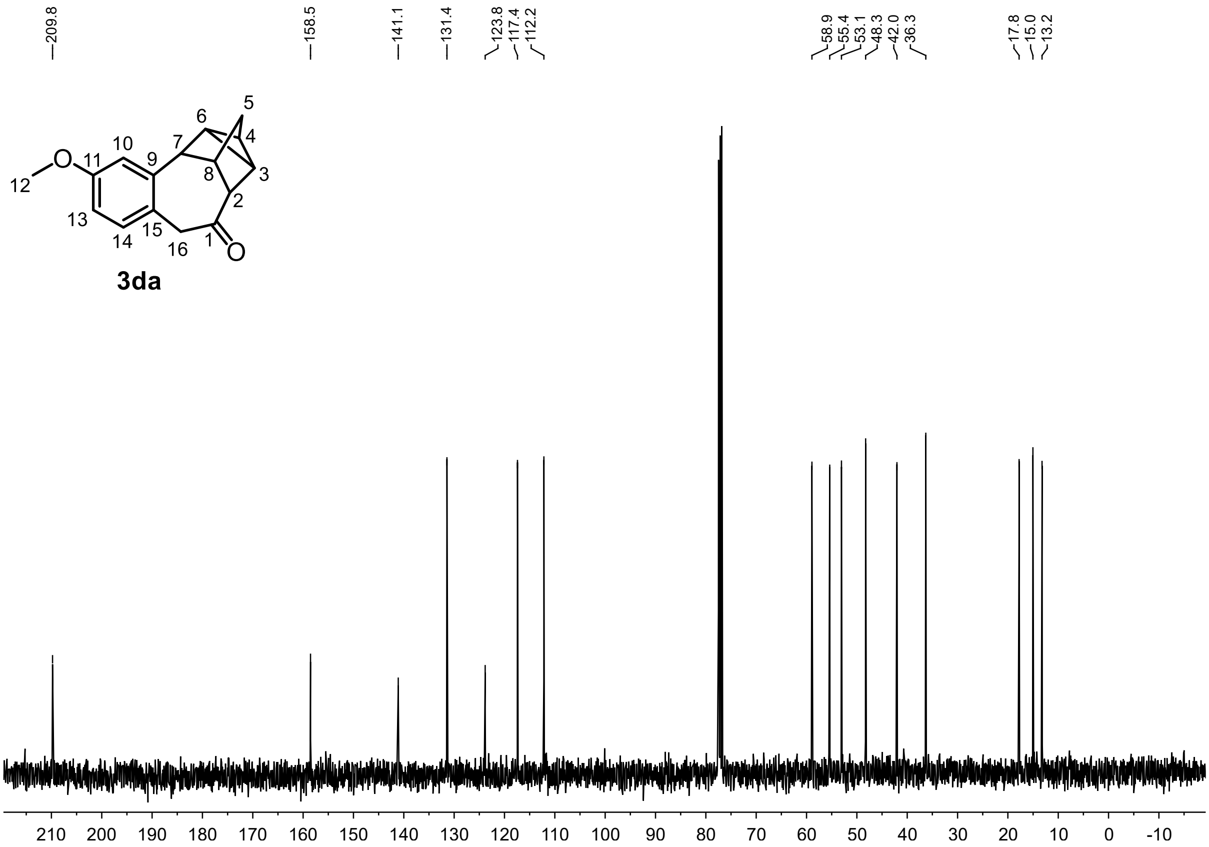

Figure S 80. ^{13}C NMR of **3da** in $CDCl_3$ measured at 100.63 MHz.

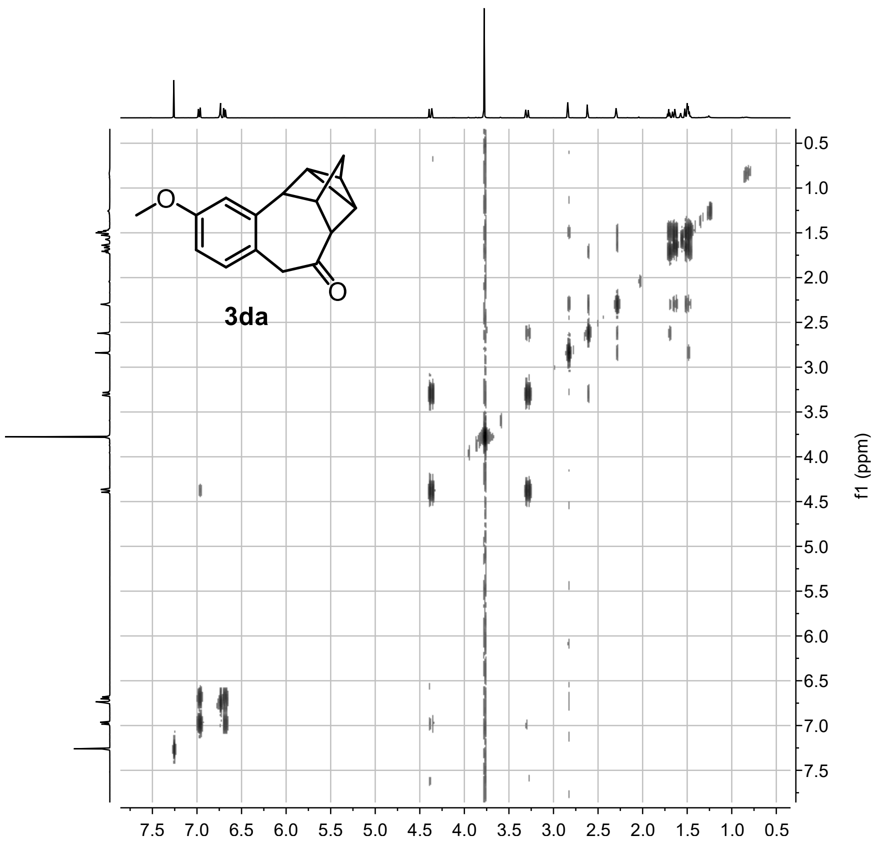

Figure S 81. ^{1}H,^{1}H-COSY of **3da** in $CDCl_3$ measured at 400.16 MHz.

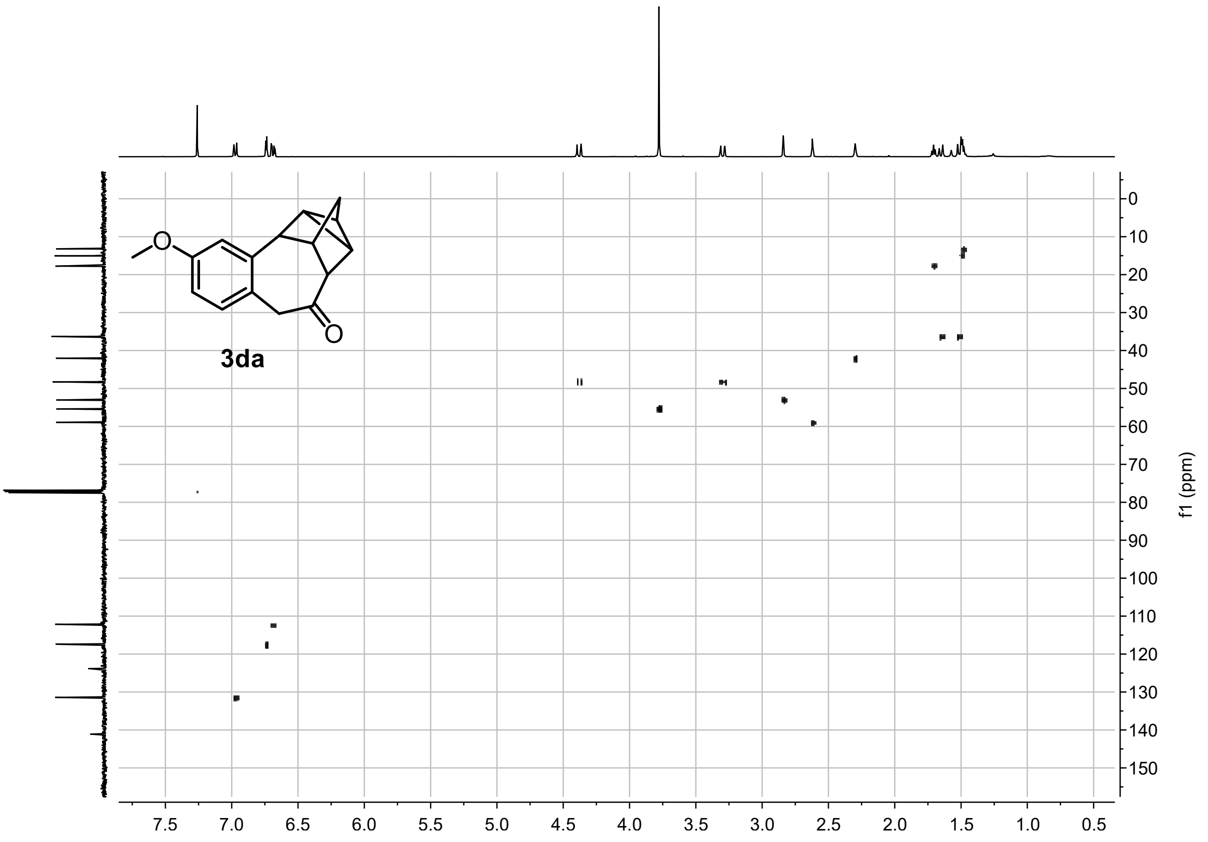

Figure S 82. ^{1}H,^{13}C-HSQC of **3da** in $CDCl_3$ measured at ^{1}H: 400.16 MHz; ^{13}C: 100.63 MHz.

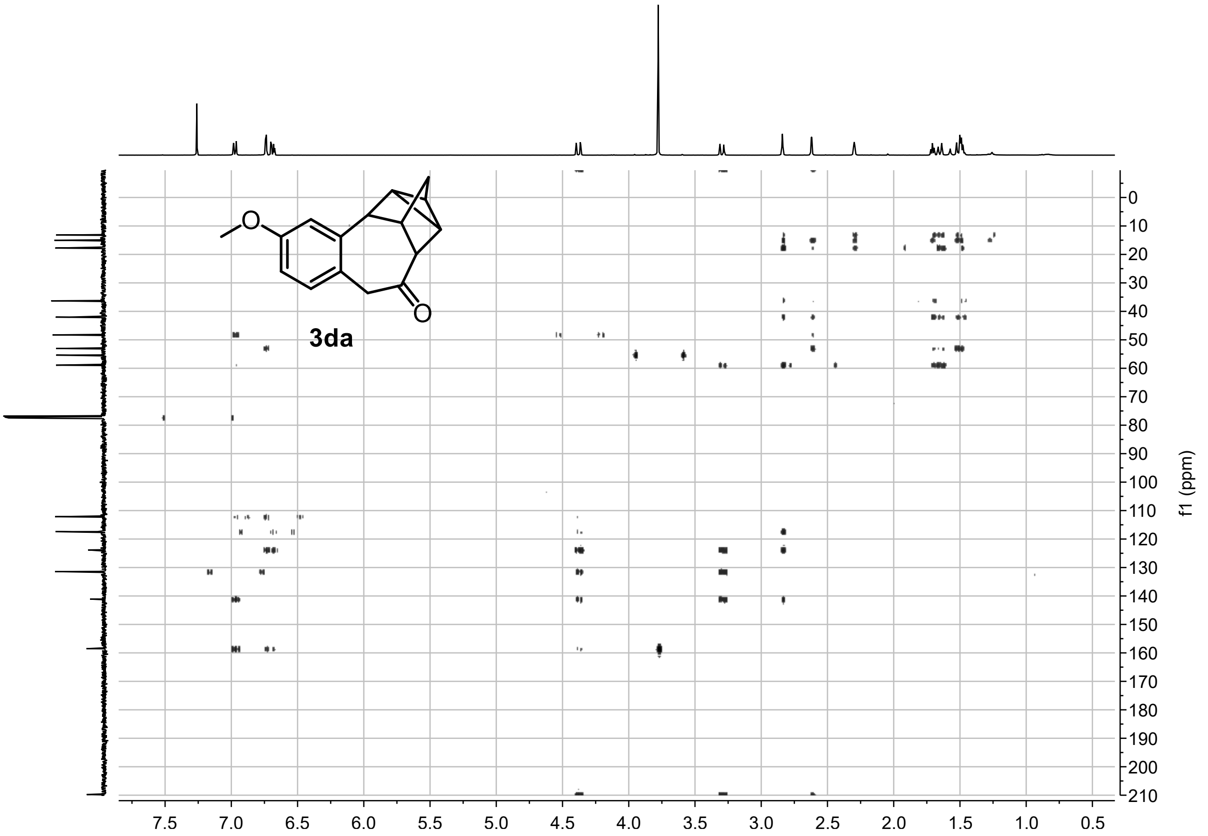

Figure S 83. ^{1}H,^{13}C-HMBC of **3da** in $CDCl_3$ measured at ^{1}H: 400.16 MHz; ^{13}C: 100.63 MHz.

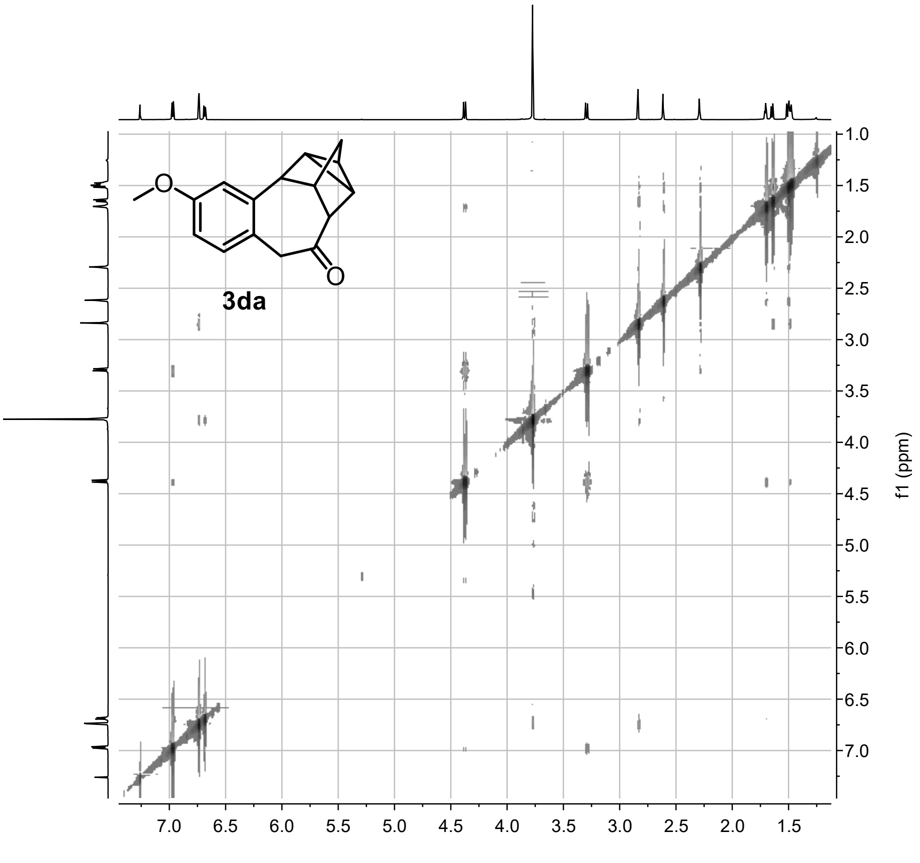

Figure S 84. ^{1}H,^{1}H-NOESY of **3da** in $CDCl_3$ measured at 700.21 MHz.

***rel*-(7a*R*,8*R*,9*R*,10a*S*,11*S*,12*S*)-7a,8,9,10,10a,11-hexahydro-8,9,11-(epimethanetriyl) azuleno[5',6':3,4]benzo[1,2-d][1,3]dioxol-7(6H)-one**

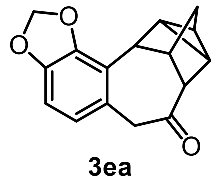

3ea

3ea was synthesized according to **GP-A** employing **1e** (162 mg, 1.00 mmol, 1.00 equiv.) and NBD (132 µL, 1.30 mmol, 1.30 equiv.). Purification *via* flash chromatography (23 g SiO_2, gradient from 90:10 to 70:30 *n*-hexane/EA over 15 CV) afforded **3ea** (187 mg, 737 µmol, 74%, mixture of isomers) as a pale brown solid.

Regioselective ratio: 86:14

$C_{16}H_{14}O_3$ (254.29 $\frac{g}{mol}$)

mp: 59.4 °C.

R$_f$: 0.53 (*n*-hexane/EA = 80:20) [anisaldehyde]

^{1}H NMR(700.21 MHz, CD_2Cl_2): δ = 6.59 (d, ^3J = 7.8 Hz, 1H, H-13), 6.52 (d, ^3J = 7.8 Hz, 1H, H-14), 5.94 (m, 1H, H-11a), 5.93 (m, 1H, H-11b), 4.27 (d, ^2J = 12.3 Hz, 1H, H-16b), 3.37 (d, ^2J = 12.3 Hz, 1H, H-16a), 3.24 (s, 1H, H-7), 2.62 (s, 1H, H-2), 2.31 (s, 1H, H-8), 1.67 (m, 1H, H-3), 1.67 (d, ^2J = 10.8 Hz, 1H, H-5a), 1.52 (d, ^2J = 10.8 Hz, 1H, H-5b), 1.48 (m, 1H, H-4), 1.48 (m, 1H, H-6).

^{13}C NMR(176.08 MHz, CD_2Cl_2): δ = 209.7 (C-1), 146.7 (C-10), 145.8 (C-12), 125.7 (C-15), 122.8 (C-14), 121.7 (C-9), 106.9 (C-13), 100.9 (C-11), 58.9 (C-2), 48.7 (C-15), 42.7 (C-2), 40.9 (C-8), 36.1 (C-5), 17.2 (C-3), 14.7 (C-6), 13.1 (C-4).

HRMS (ESI-TOF) m/z: $[M+H]^+$ Calcd for $C_{16}H_{14}O_3H$ 255.1016; Found 255.1017.

IR (ATR, ṽ): 1688 cm^{-1} (s, *CO*).

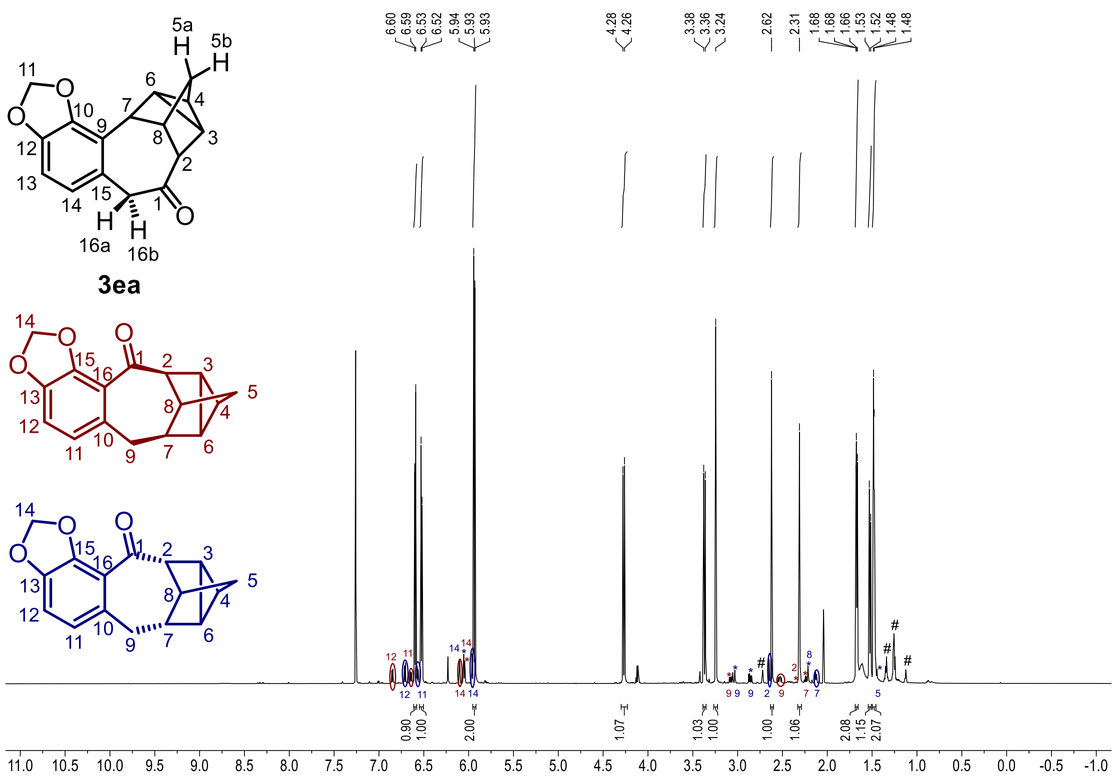

Figure S 85. ^{1}H NMR of **3ea** with the two diastereomers of the respective regioisomer in $CDCl_3$ measured at 700.21 MHz. The assigned signals of the diastereomers are color coded and their ambiguous signals are denoted by #.

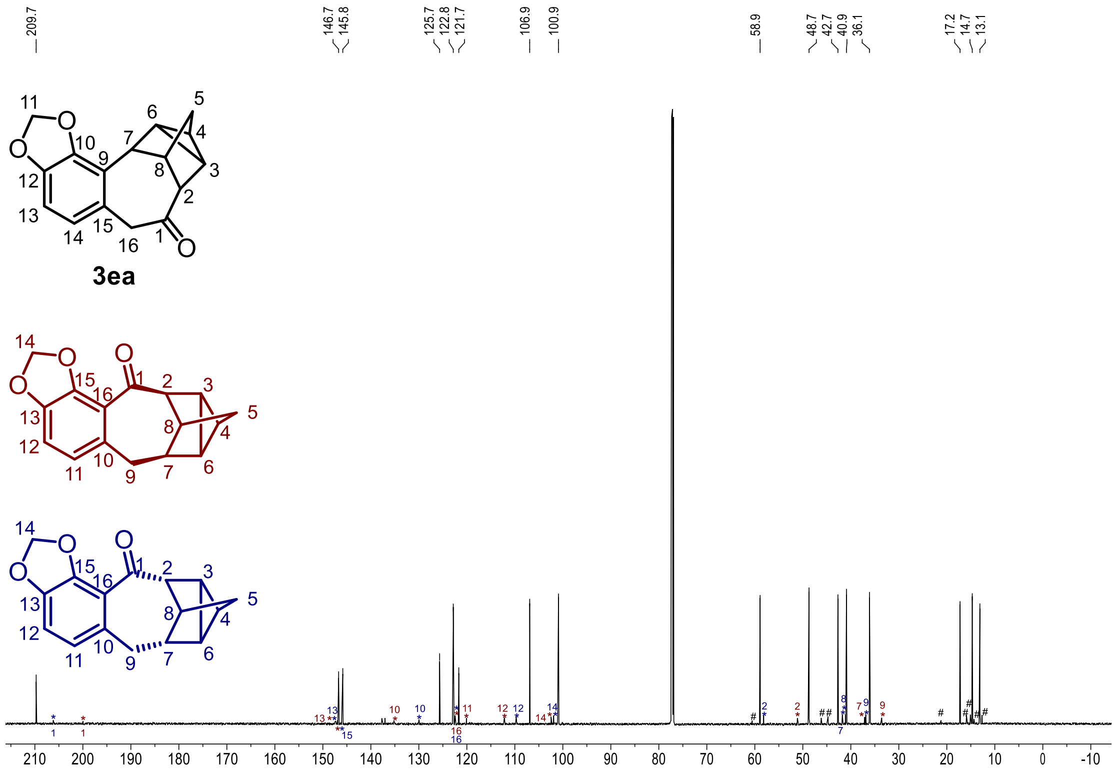

Figure S 86. ^{13}C NMR of **3ea** with the two diastereomers of the respective regioisomer in $CDCl_3$ measured at 176.08 MHz. The assigned signals of the diastereomers are color coded and their ambiguous signals are denoted by #.

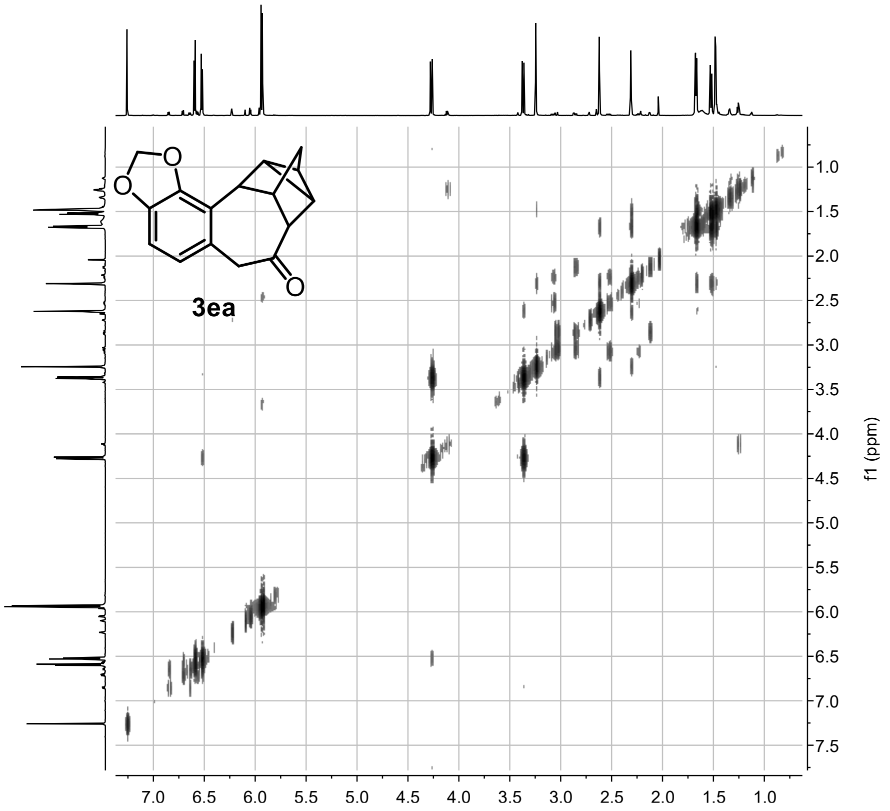

Figure S 87. $^{1}H,^{1}H$-COSY of **3ea** with the two diastereomers of the respective regioisomer in $CDCl_3$ measured at 700.21 MHz.

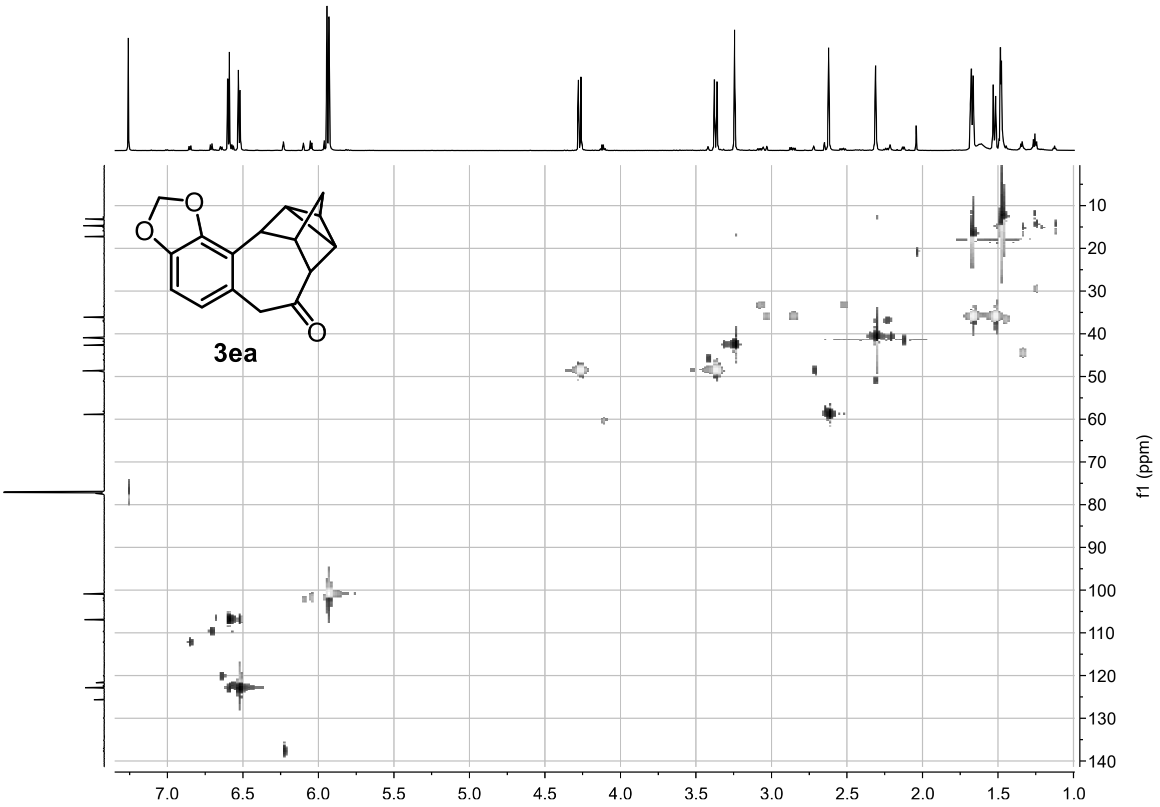

Figure S 88. $^{1}H,^{13}C$-HSQC of **3ea** with the two diastereomers of the respective regioisomer in $CDCl_3$ measured at ^{1}H: 700.21 MHz; ^{13}C: 176.08 MHz.

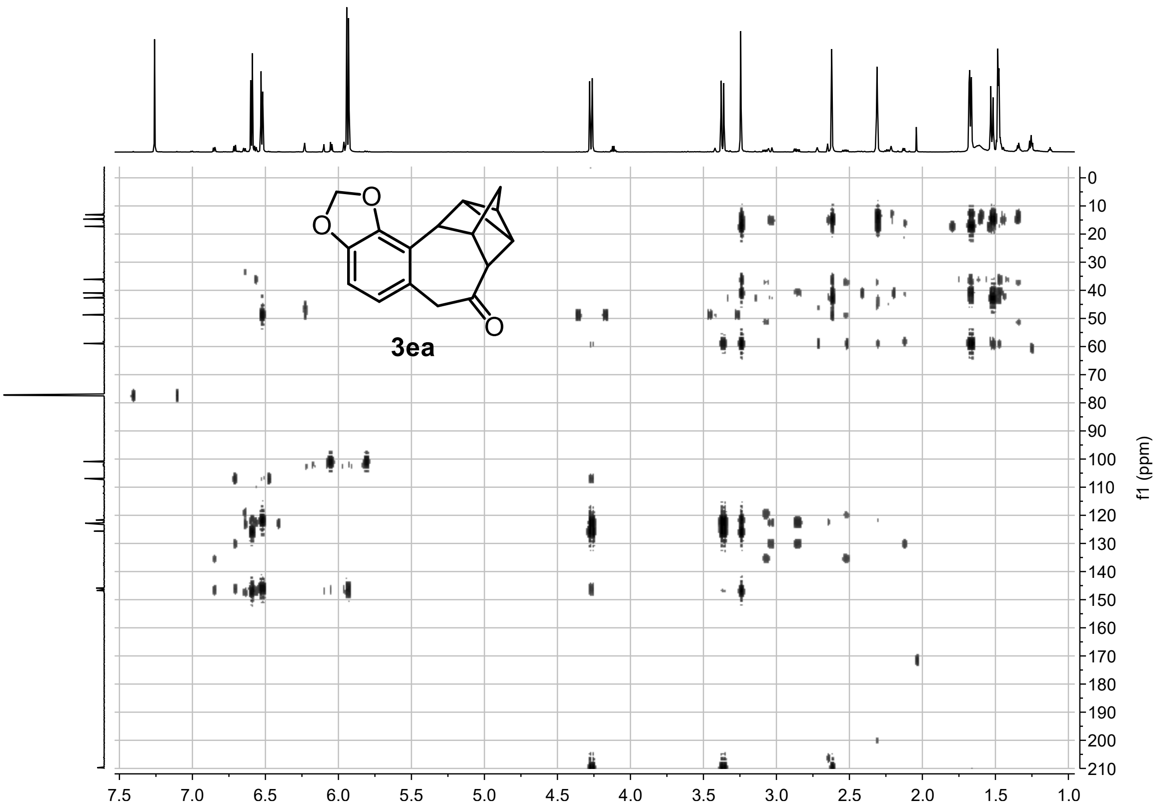

Figure S 89. $^{1}H,^{13}C$-HMBC of **3ea** with the two diastereomers of the respective regioisomer in $CDCl_3$ measured at ^{1}H: 700.21 MHz; ^{13}C: 176.08 MHz.

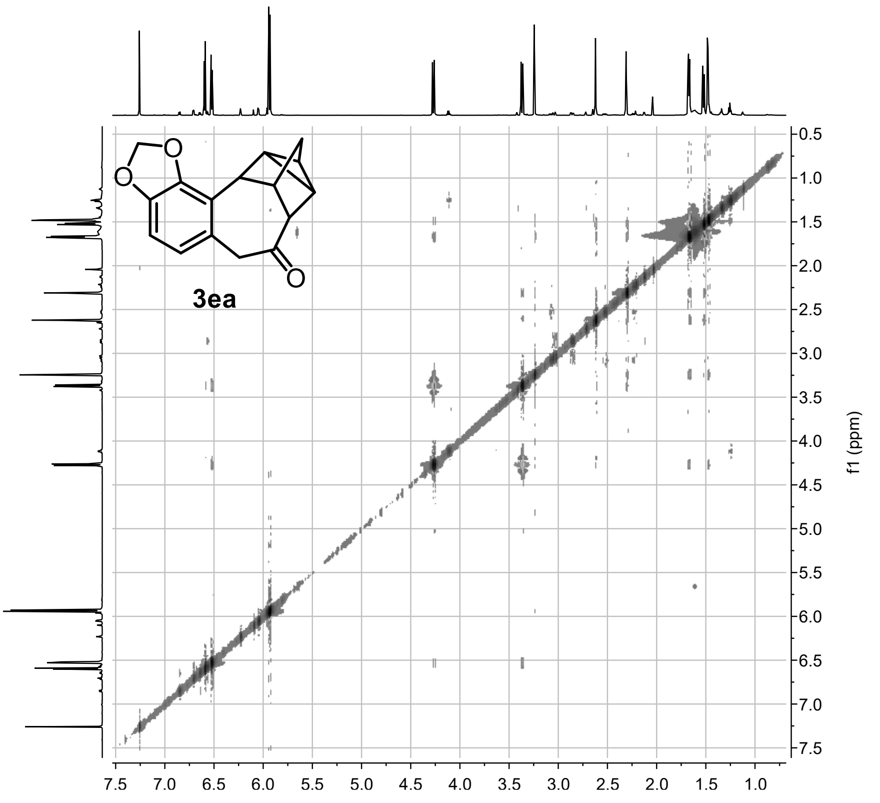

Figure S 90. $^{1}H,^{1}H$-NOESY of **3ea** with the two diastereomers of the respective regioisomer in $CDCl_3$ measured at 700.21 MHz.

***rel*-5-fluoro-(1*S*,2*S*,3a*R*,4*R*,10a*S*,11*R*)-2,3,3a,4,9,10a-hexahydro-1,2,4-(epimethanetriyl) benzo[f]azulen-10(1H)-one**

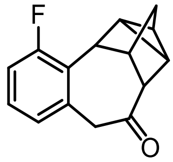

3fa

3fa was synthesized according to **GP-A** employing **1f** (136 mg, 1.00 mmol, 1.00 equiv.) and NBD (132 µL, 1.30 mmol, 1.30 equiv.) at 100 °C instead of 80 °C. Purification *via* flash chromatography (23 g SiO_2, gradient from 100:0 to 60:40 *n*-hexane/EA over 20 CV) afforded **3fa** (89.2 mg, 391 µmol, 39%, 29% *ee*, $[\alpha]_D^{20}$ +8 (*c* 1.0, $CHCl_3$)) as a pale yellow solid.

Regioisomeric ratio: 87:13

$C_{15}H_{13}FO$ (228.27 $\frac{g}{mol}$)

mp: 62.1 °C.

R_f: 0.66 (*n*-hexane/EA = 80:20) [anisaldehyde]

^{1}H NMR(400.16 MHz, $CDCl_3$): δ = 7.09 (m, 1H, H-12), 6.92 (m, 1H, H-11), 6.82 (d, ^3J = 7.6 Hz, 1H, H-13), 4.23 (d, ^2J = 12.4 Hz, 1H, H-15b), 3.58 (d, ^2J = 12.4 Hz, 1H, H-15a), 3.46 (s, 1H, H-7), 2.63 (s, 1H, H-2), 2.35 (s, 1H, H-8), 1.69 (dm, ^2J = 10.7 Hz, 1H, H-5a), 1.66 (ddm, ^3J = 5.5 Hz, ^3J = 5.0 Hz, 1H, H-6), 1.54 (dm, ^2J = 10.7 Hz, 1H, H-5b), 1.48 (m, 1H, H-4), 1.47 (m, 1H, H-3).

^{13}C NMR(100.62 MHz, $CDCl_3$): δ = 208.7 (C-1), 161.4 (d, ^1J = 244 Hz, C-10), 134.0 (d, ^3J = 3 Hz, C-14), 128.1 (d, ^3J = 10 Hz, C-12), 126.8 (d, ^2J = 13 Hz, C-9), 125.9 (d, ^4J = 3 Hz, C-13), 114.1 (d, ^2J = 25 Hz, C-11), 58.6 (C-2), 49.2 (C-15), 41.2 (d, ^3J = 7 Hz, C-7), 39.8 (C-8), 36.0 (C-5), 16.9 (C-6), 15.3 (C-3), 13.1 (C-4).

^{19}F NMR(376.49 MHz, $CDCl_3$): δ = -116.6.

HRMS (ESI-TOF) m/z: $[M+Na]^+$ Calcd for $C_{15}H_{13}FONa$ 251.0843; Found 251.0845.

IR (ATR, ṽ): 1701 cm^{-1} (s, *CO*).

Regiosiomer:

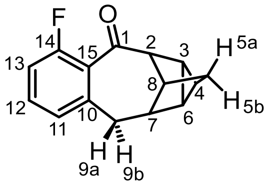

^{1}H NMR(400.16 MHz, $CDCl_3$): δ = 7.23 (ddd, , ^3J = 8.2 Hz, ^3J = 8.2 Hz, ^4J = 5.7 Hz, 1H, H-12), 6.94 (ddm, ^3J = 9.8 Hz, ^3J = 8.6 Hz, 1H, H-11), 6.90 (d, ^3J = 7.5 Hz, 1H, H-13), 3.09 (d, ^2J = 15.9 Hz, 1H, H-9a), 2.90 (ddd, ^2J = 15.9 Hz, ^3J = 7.6 Hz, ^4J = 1.2 Hz, 1H, H-9b), 2.65 (m, 1H, H-2), 2.22 (m, 1H, H-8), 2.14 (dm, ^3J = 7.6 Hz, 1H, H-7), 1.53 (dm, ^2J = 10.7 Hz, 1H, H-5b), 1.47 (dm, ^3J = 10.8 Hz, 1H, H-5a), 1.31 (m, 1H, H-4), 1.28 (m, 1H, H-3), 1.07 (m, 1H, H-6).

^{13}C NMR(100.62 MHz, $CDCl_3$): δ = 205.9 (C-1), 159.2 (d, ^1J = 252 Hz, C-14), 137.1 (d, ^2J = 66 Hz, C-15), 131.2 (d, ^3J = 9 Hz, C-12), 128.5 (d, ^3J = 14 Hz, C-10), 126.3 (d, ^4J = 3 Hz, C-11), 114.4 (d, ^2J = 22 Hz, C-13), 57.3 (C-2), 41.7 (C-8), 41.2 (C-7), 36.8 (C-5), 35.8 (C-9), 15.3 (C-4), 15.1 (C-6), 12.9 (C-3).

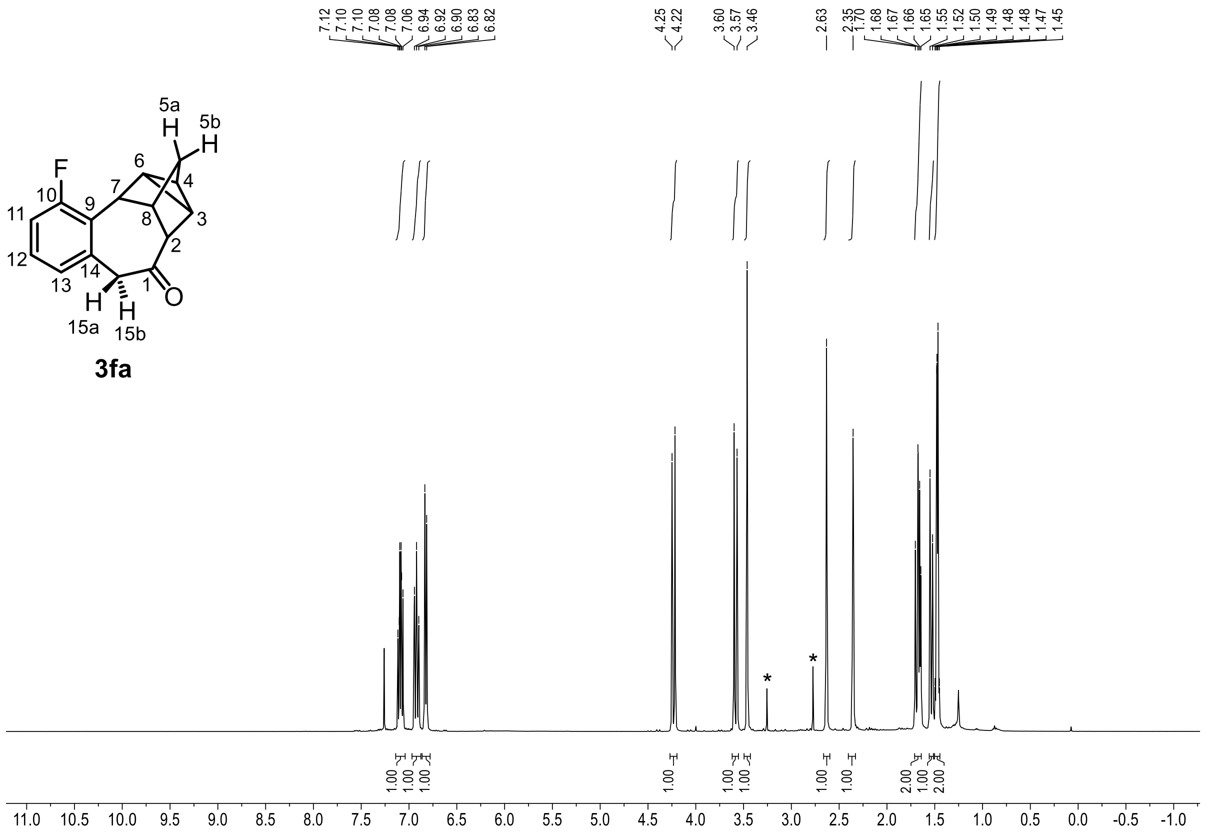

Figure S 91. ^{1}H NMR of **3fa** in $CDCl_3$ measured at 400.16 MHz. * Denotes residual DMI.

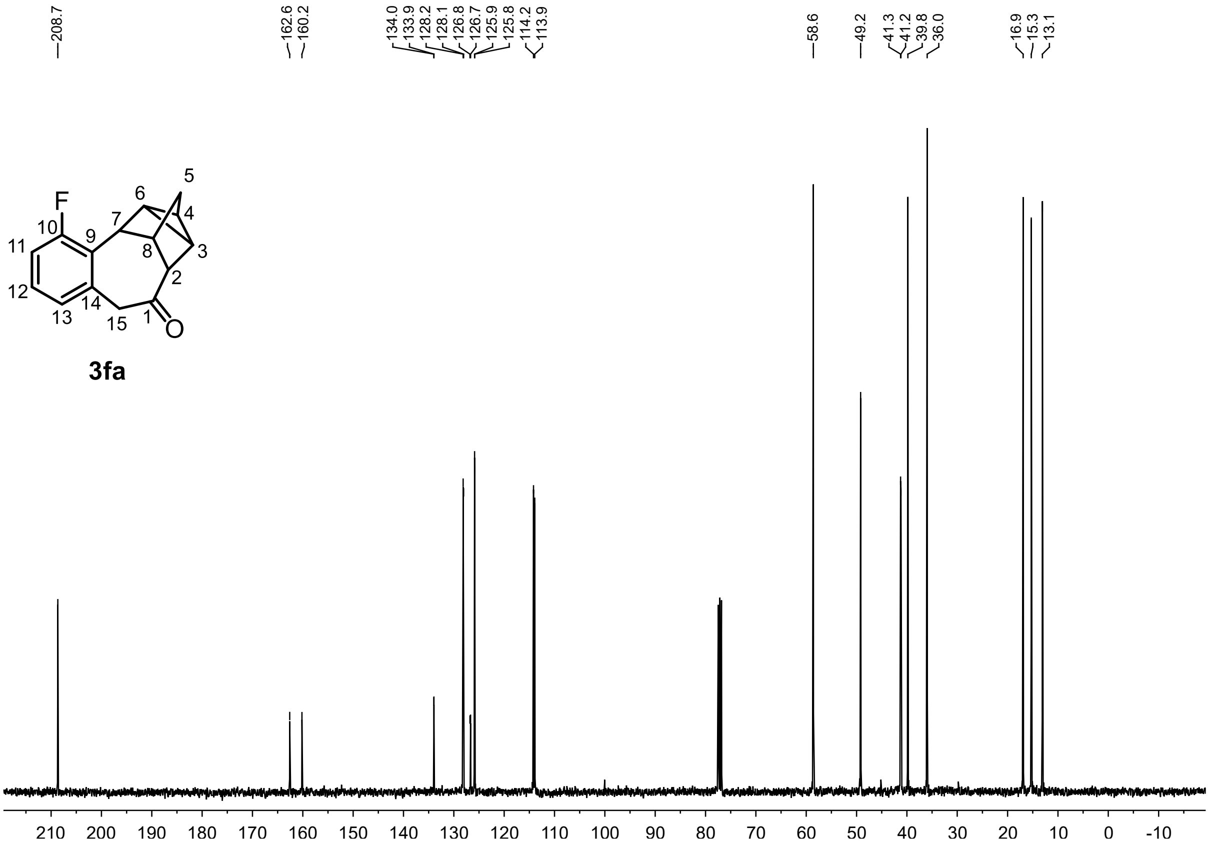

Figure S 92. ^{13}C NMR of **3fa** in $CDCl_3$ measured at 100.63 MHz.

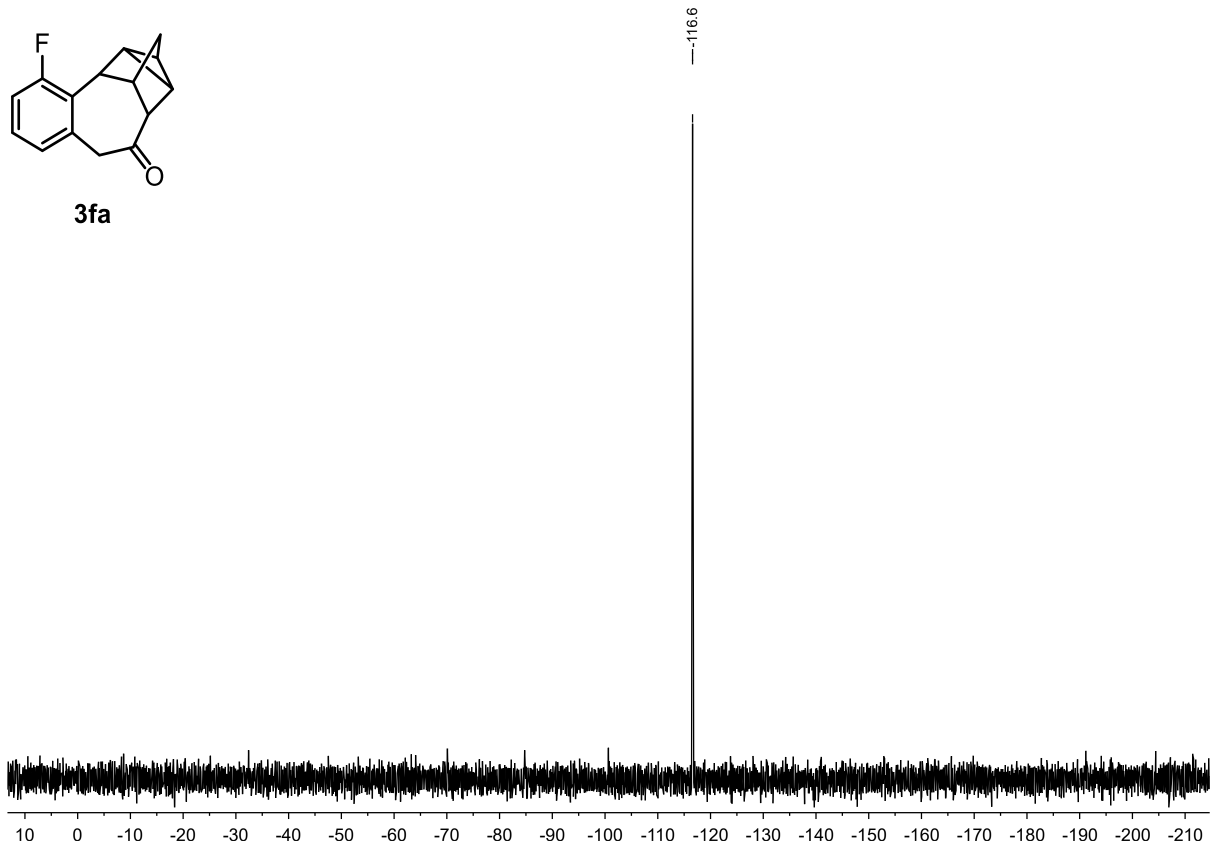

Figure S 93. ^{19}F NMR of **3fa** in $CDCl_3$ measured at 376.49 MHz.

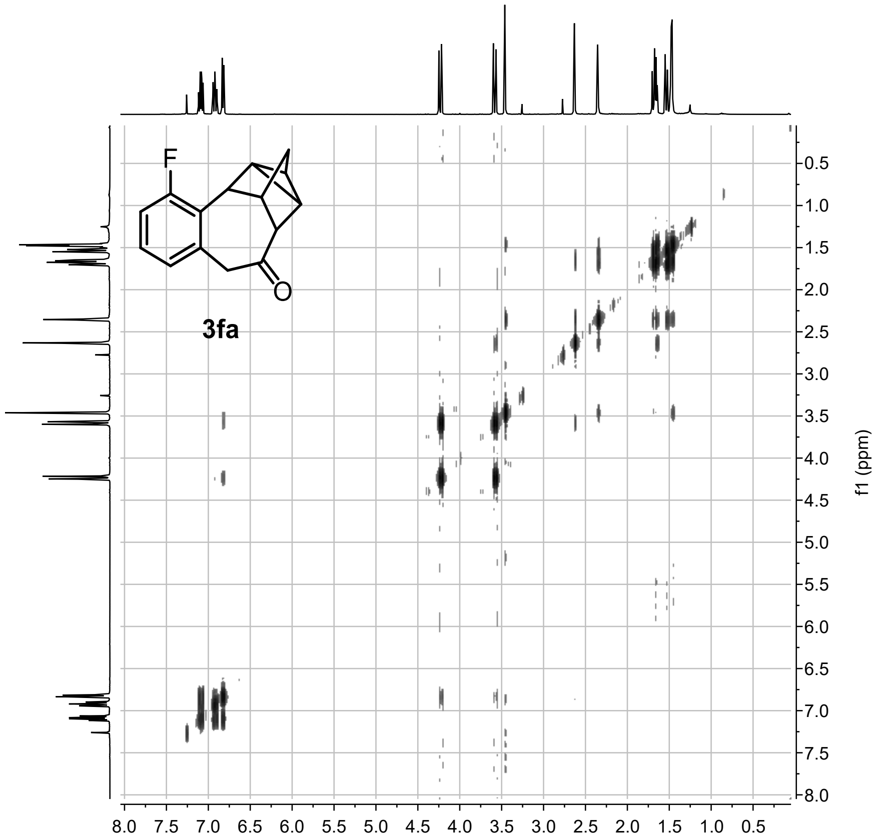

Figure S 94. ^{1}H,^{1}H-COSY of **3fa** in $CDCl_3$ measured at 400.16 MHz.

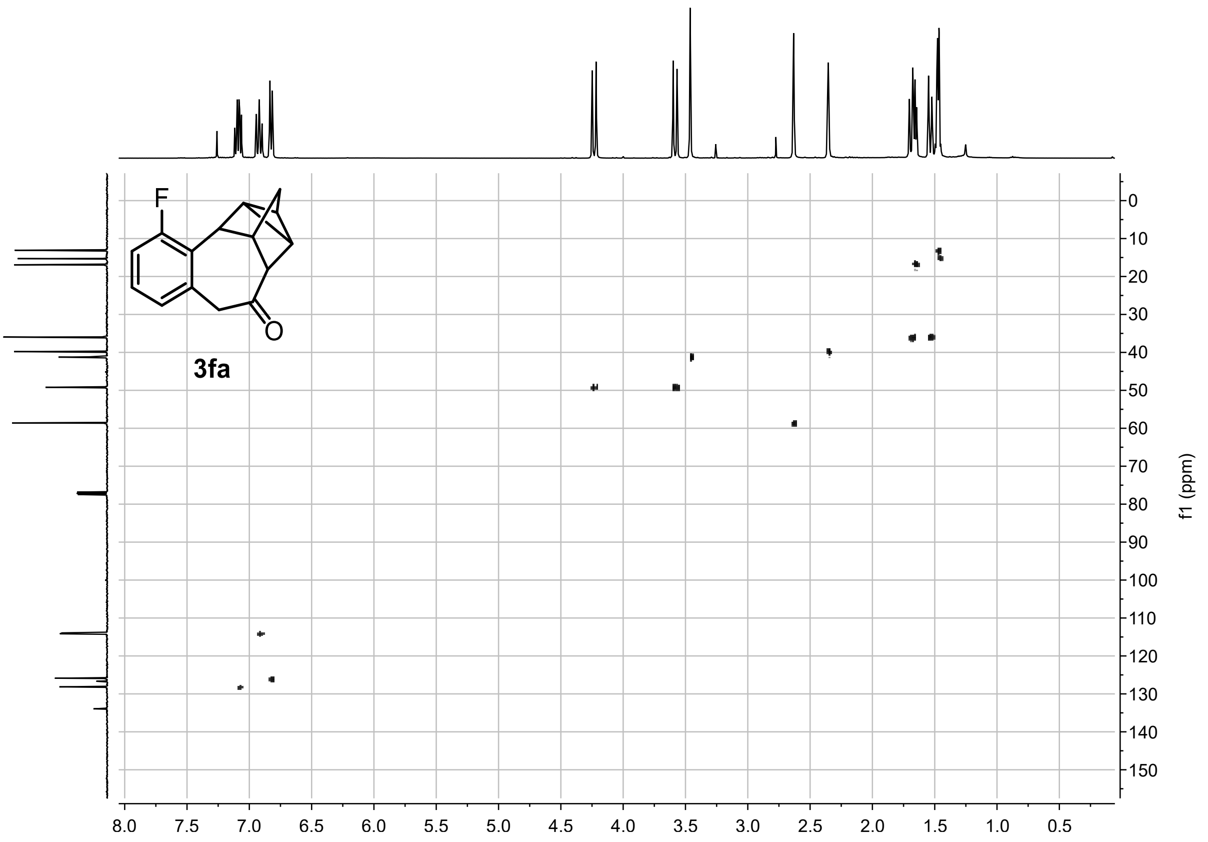

Figure S 95. $^{1}H,^{13}C$-HSQC of **3fa** in $CDCl_3$ measured at ^{1}H: 700.21 MHz; ^{13}C: 176.08 MHz.

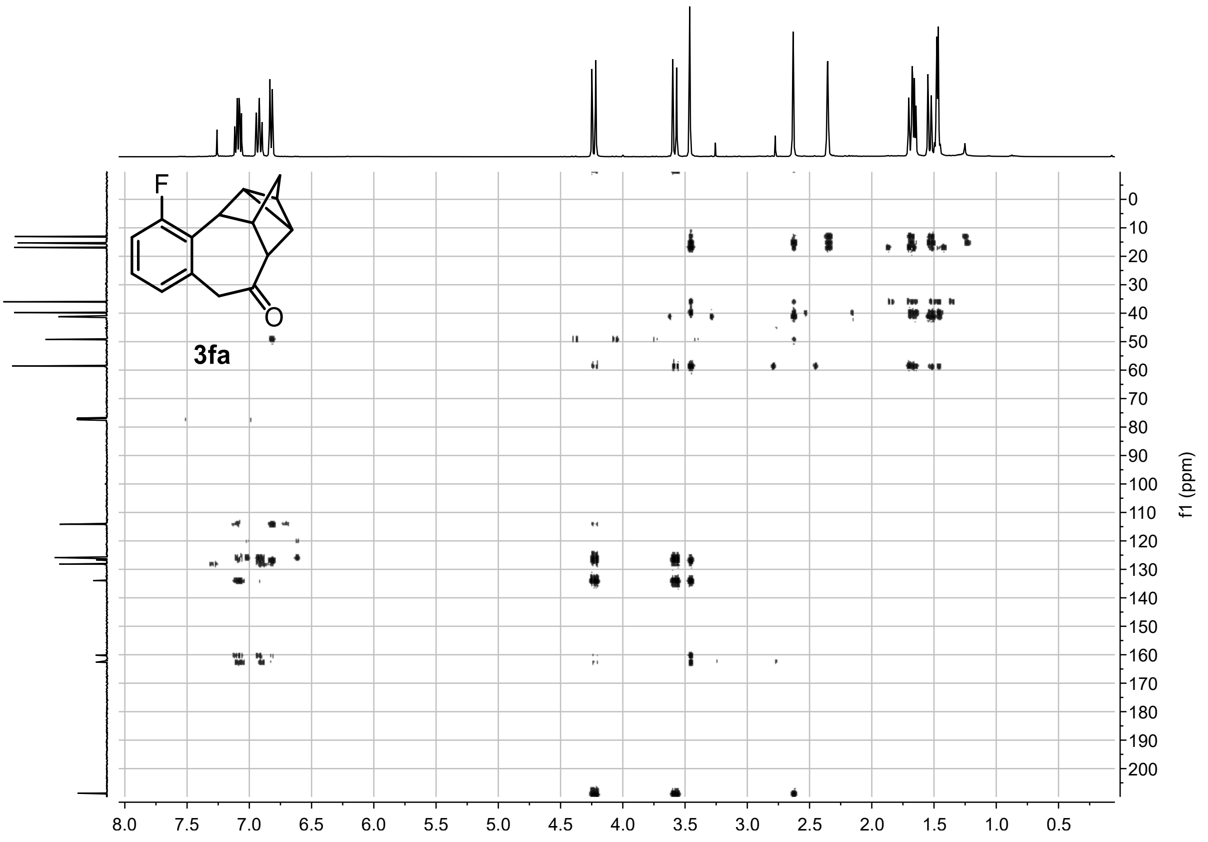

Figure S 96. $^{1}H,^{13}C$-HMBC of **3fa** in $CDCl_3$ measured at ^{1}H: 700.21 MHz; ^{13}C: 176.08 MHz.

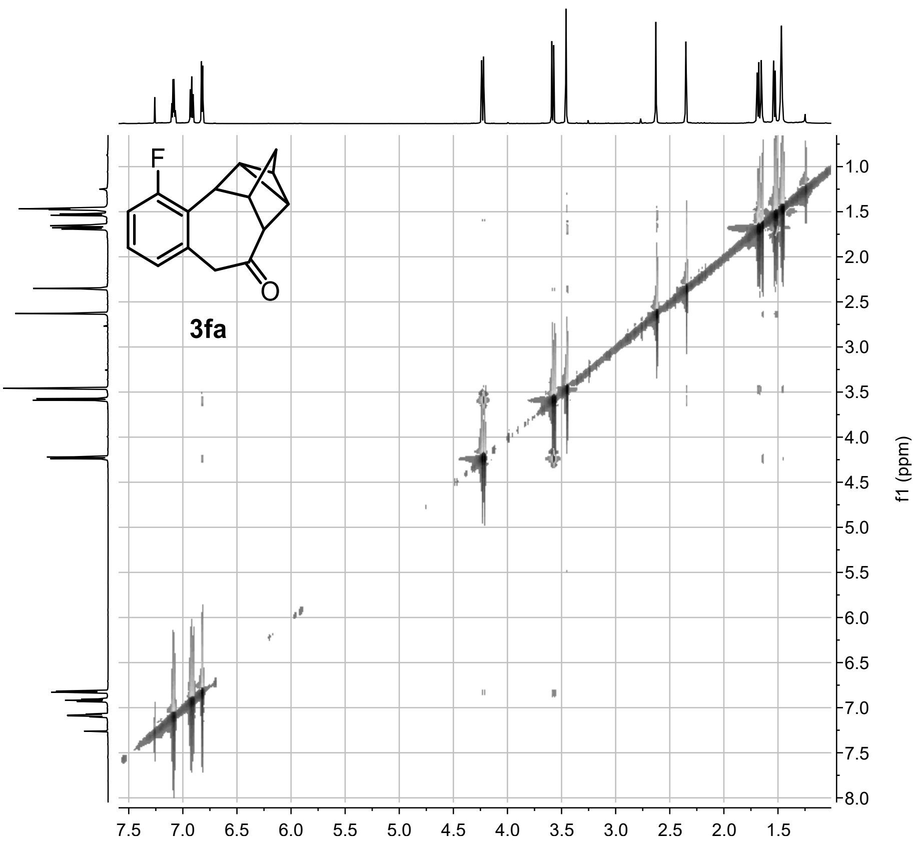

Figure S 97. $^{1}H,^{1}H$-NOESY of **3fa** in $CDCl_3$ measured at 700.21 MHz.

***rel*-(1*R*,2*R*,3a*S*,4*S*,9*R*,10a*R*,11*S*)-9-methyl-2,3,3a,4,9,10a-hexahydro-1,2,4-(epimethanetriyl)benzo[f]azulen-10(1H)-one (mixture of enantiomers)**

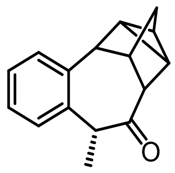

3ga

3ga was synthesized according to **GP-A** employing **1g** (132 mg, 1.00 mmol, 1.00 equiv.) and NBD (132 µL, 1.30 mmol, 1.30 equiv.) at 50 °C instead of 80 °C. Purification *via* flash chromatography (23 g SiO_2, gradient from 100:00 to 90:10 *n*-hexane/EA over 10 CV) afforded **3ga** (137 mg, 610 µmol, 61%, mixture of isomers) as colorless crystals.

d.r.: 95:5

$C_{16}H_{16}O$ (224.30 $\frac{\text{g}}{\text{mol}}$)

mp: 85.6 °C.

R_f: 0.68 (*n*-hexane/EA = 80:20) [anisaldehyde]

^{1}H NMR(400.16 MHz, $CDCl_3$): δ = 7.22 (m, 1H, H-10), 7.20 (m, 2H, H-11/12), 7.14 (m, 1H, H-13), 4.28 (q, ^3J = 6.9 Hz, 1H, H-15), 3.16 (s, 1H, H-7), 2.70 (m, 1H, H-8), 2.63 (m, 1H, H-2), 1.64 (dm, ^2J = 10.6 Hz, 1H, H-5a), 1.56 (dm, ^2J = 10.6 Hz, 1H, H-5b), 1.53 (m, 1H, H-3), 1.48 (d, ^3J = 6.9 Hz, 3H, H-16), 1.40 (m, 1H, H-4), 1.38 (m, 1H, H-6); *diastereomer* δ = 7.22 (m, 1H), 7.20 (m, 2H), 7.16 (m, 1H, H-10), 4.86 (q, ^3J = 6.8 Hz, 1H, H-15), 2.90 (s, 1H, H-2), 2.71 (m, 1H, H-7), 2.24 (m, 1H, H-8), 1.75 (m, 1H, H-6), 1.65 (dm, ^2J = 11.6 Hz, 1H, H-5a), 1.53 (m, 1H, H-3), 1.52 (dm, ^2J = 11.6 Hz, 1H, H-5b), 1.43 (m, 1H, H-4), 1.42 (d, ^3J = 6.8 Hz, 3H, H-16).

^{13}C NMR(100.62 MHz, $CDCl_3$): δ = 210.7 (C-1), 139.4 (C-9), 134.6 (C-14), 131.2 (C-10), 127.2 (C-12), 126.8 (C-11), 126.6 (C-12), 57.7 (C-2), 52.6 (C-7), 48.6 (C-15), 35.4 (C-8), 35.3 (C-5), 21.2 (C-6), 15.0 (C-16), 14.5 (C-3), 13.5 (C-4) *diastereomer* δ = 139.4 (C-9), 134.5 (C-14), 131.5 (C-10), 127.6, 126.8, 125.2, 53.8 (C-2), 44.2 (C-15), 44.1 (C-8), 36.2 (C-7), 36.2 (C-5), 18.2 (C-6), 14.1 (C-16), 13.7 (C-3), 13.0 (C-4).

HRMS (ESI-TOF) m/z: $[M+Na]^+$ Calcd for $C_{16}H_{16}ONa$ 247.1093; Found 247.1095.

IR (ATR, ṽ): 1699 cm^{-1} (s, *CO*).

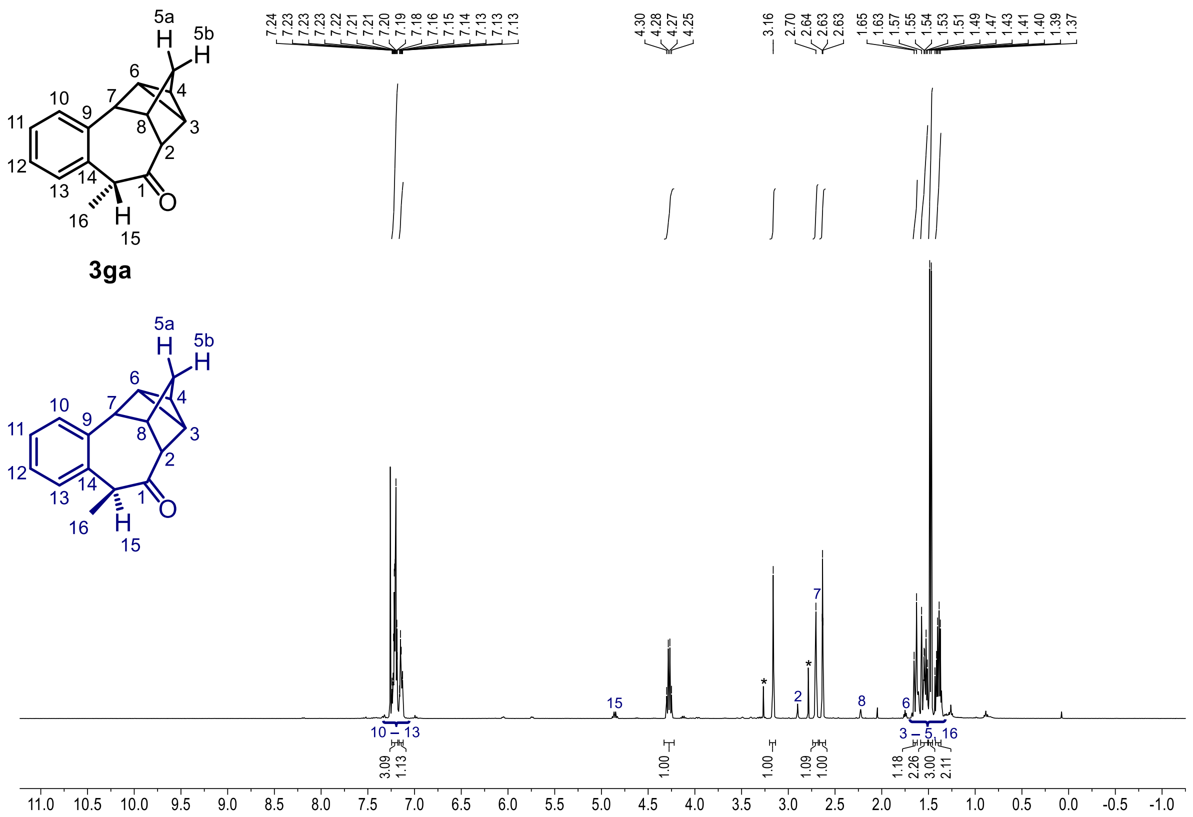

Figure S 98. ^{1}H NMR of **3ga** and the respective diastereomer (blue) in $CDCl_3$ measured at 400.16 MHz. * denotes residual DMI.

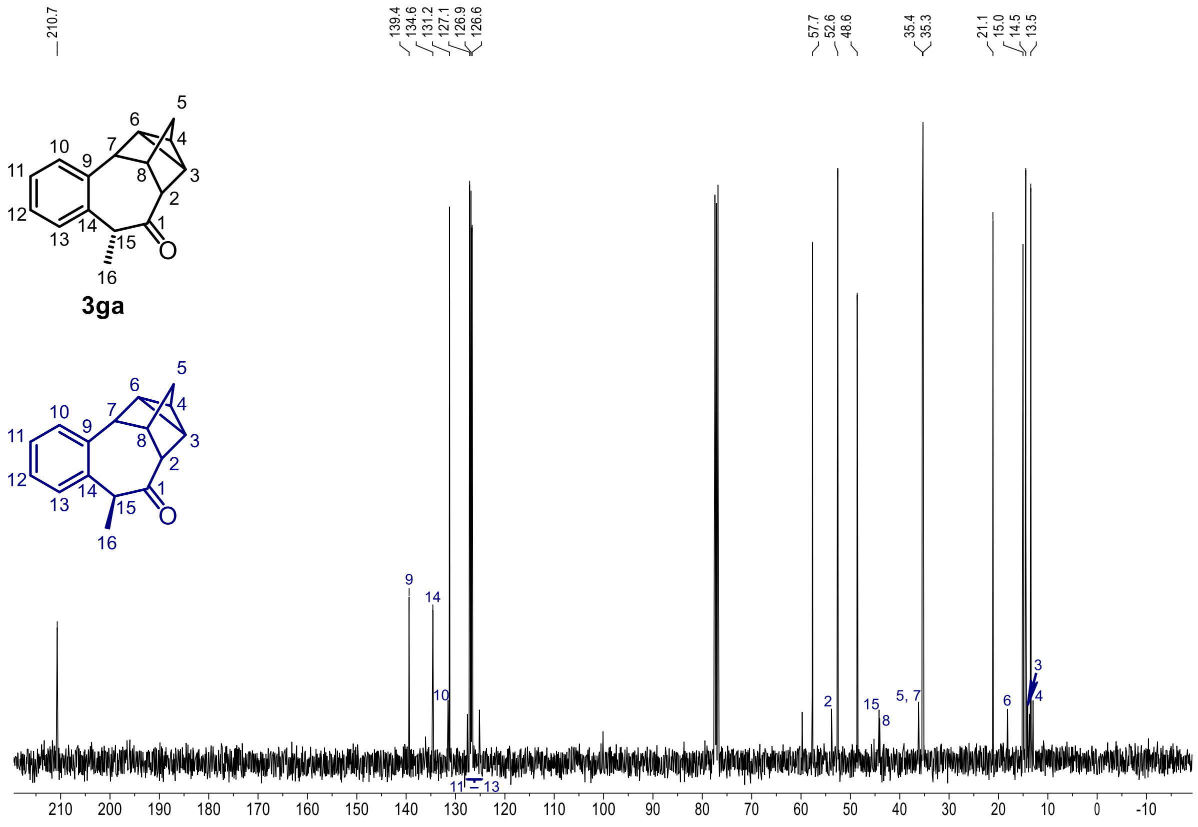

Figure S 99. ^{13}C NMR of **3ga** and the respective diastereomer (blue) in $CDCl_3$ measured at 100.63 MHz.

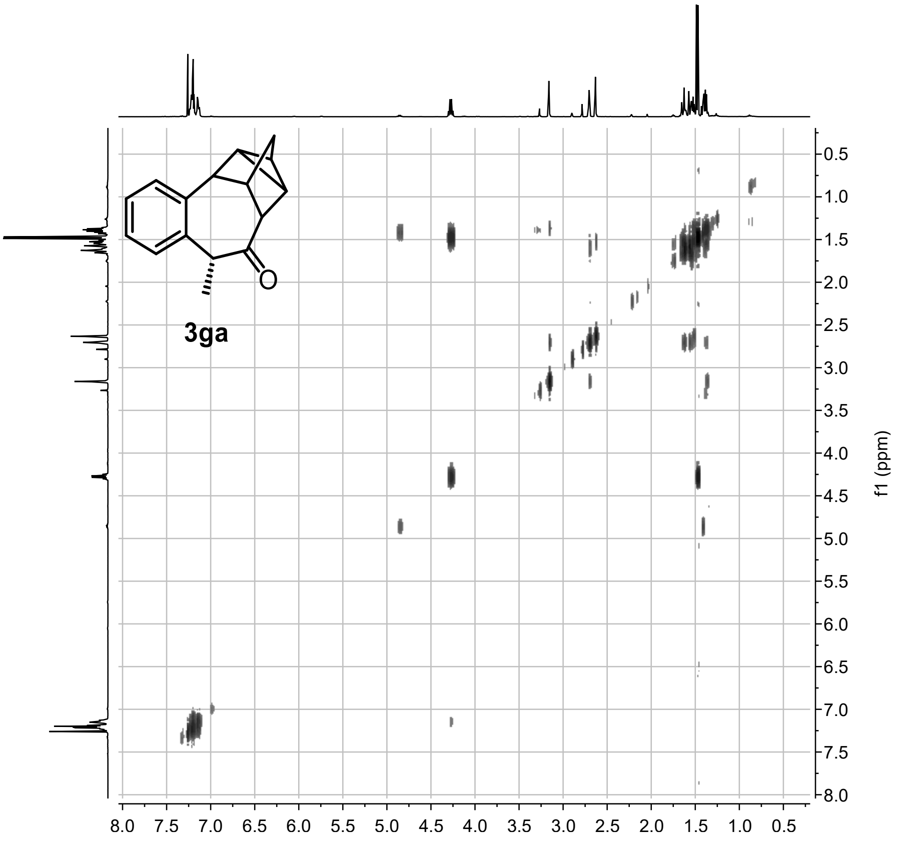

Figure S 100. $^{1}H,^{1}H$-COSY of **3ga** in $CDCl_3$ measured at 400.16 MHz.

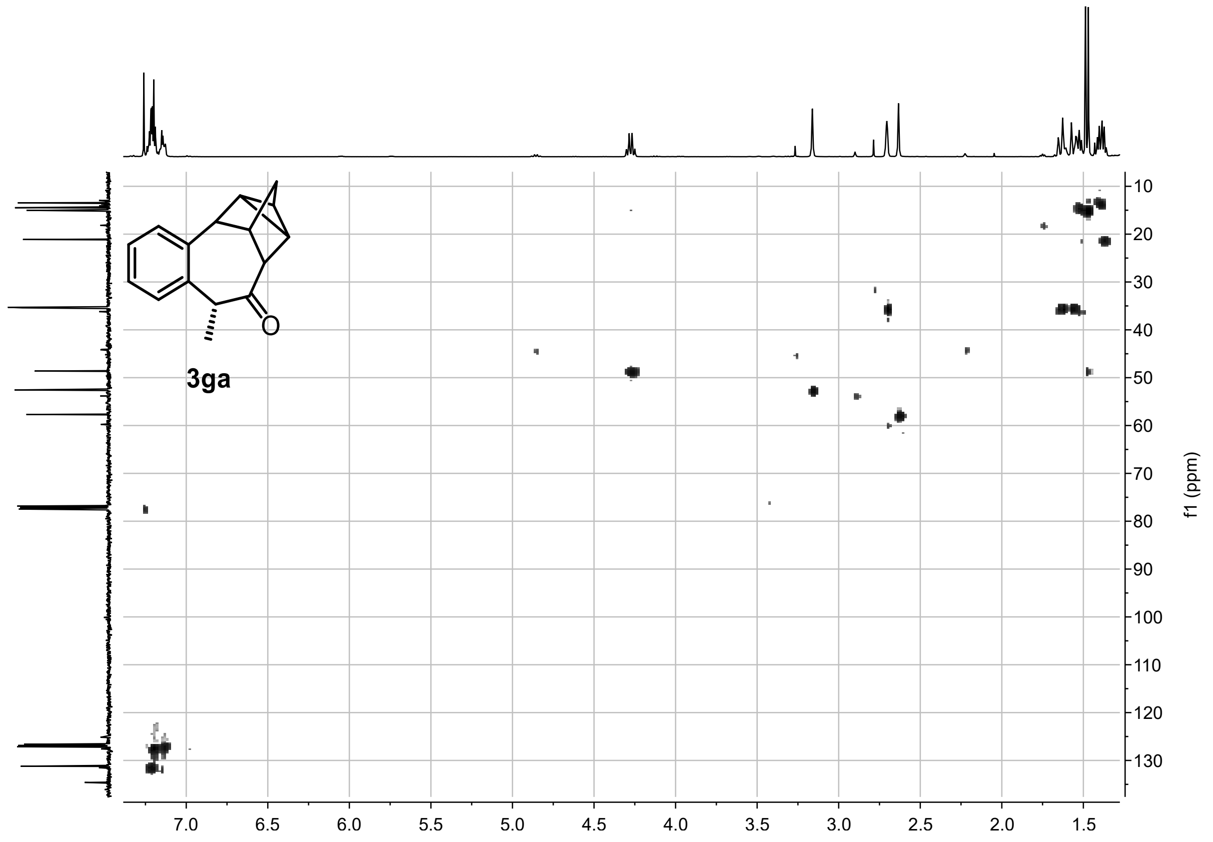

Figure S 101. $^{1}H,^{13}C$-HSQC of **3ga** in $CDCl_3$ measured at ^{1}H: 400.16 MHz; ^{13}C: 100.63 MHz.

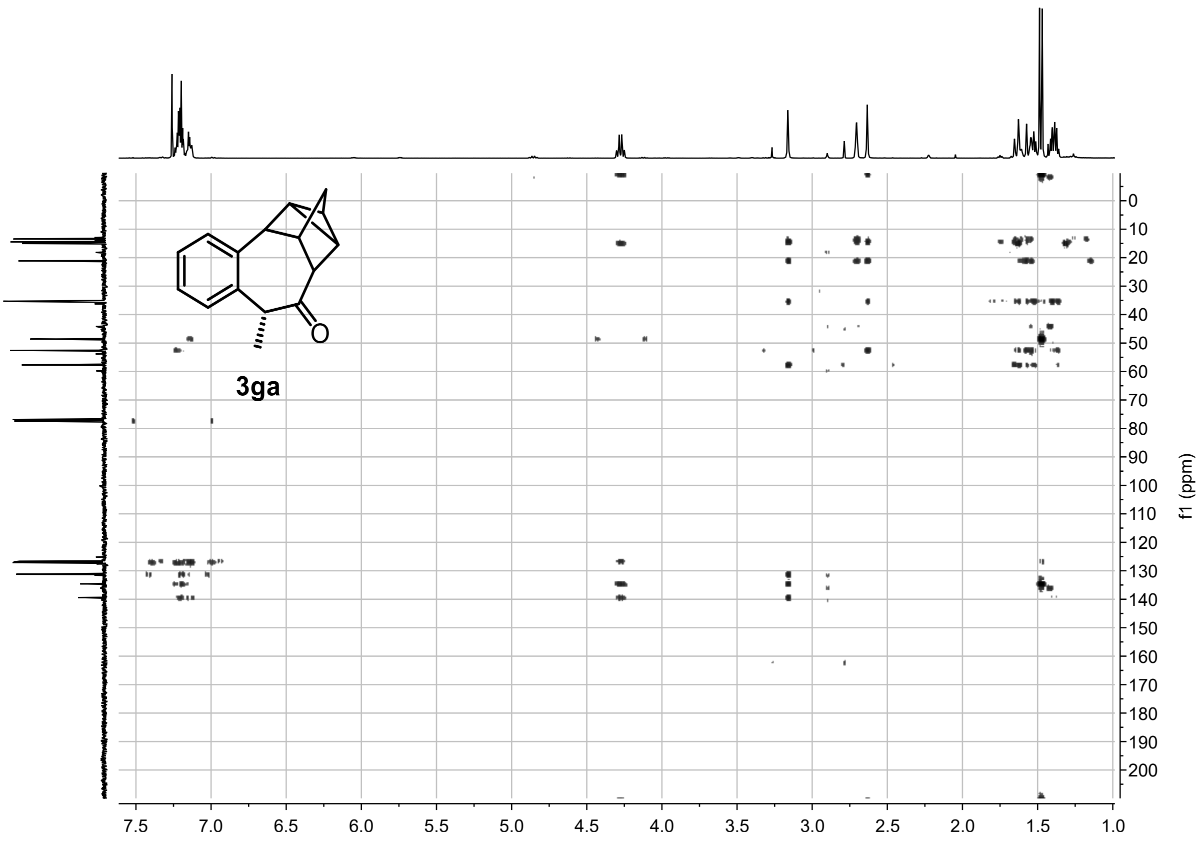

Figure S 102. $^{1}H,^{13}C$-HMBC of **3ga** in $CDCl_3$ measured at ^{1}H: 400.16 MHz; ^{13}C: 100.63 MHz.

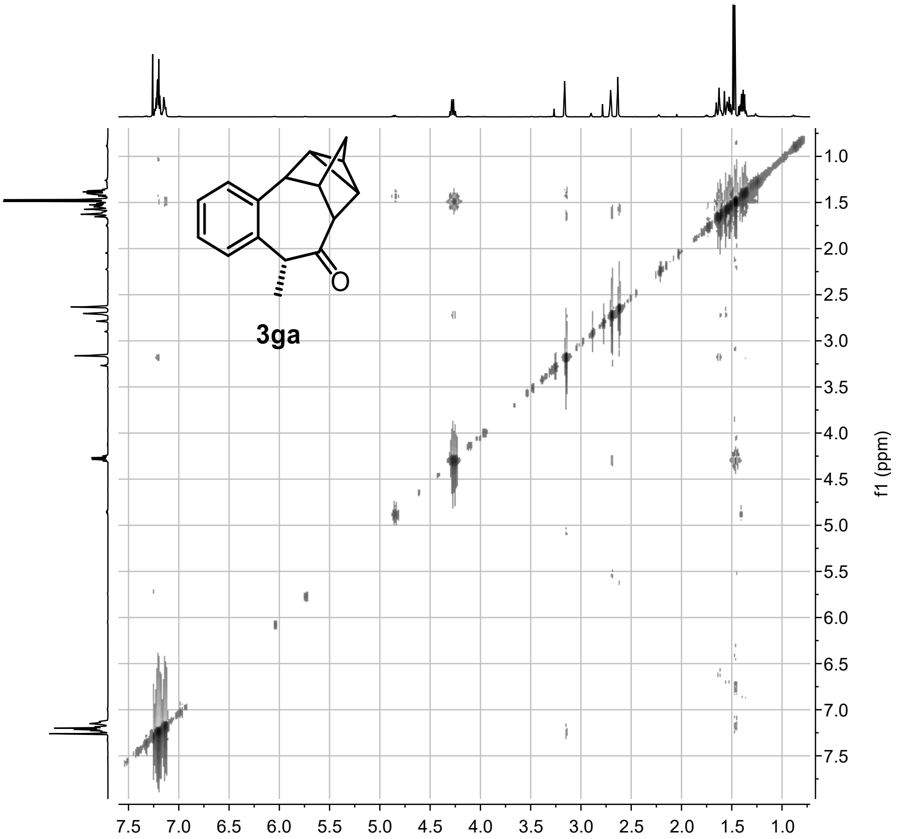

Figure S 103. $^{1}H,^{1}H$-NOESY of **3ga** in $CDCl_3$ measured at 400.16 MHz.

***rel*-(1*R*,2*R*,3a*S*,4*S*,9*R*,10a*R*,11*S*)-9-ethyl-2,3,3a,4,9,10a-hexahydro-1,2,4-(epimethanetriyl)benzo[f]azulen-10(1H)-one (mixture of enantiomers)**

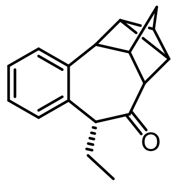

3ha

3ha was synthesized according to **GP-A** employing **1h** (146 mg, 1.00 mmol, 1.00 equiv.), NBD (305 µL, 3.00 mmol, 3.00 equiv.), Ni(COD)$_2$ (20 mol%) and (*R*)-(+)-(3,5-dioxa-4-phosphacyclohepta[2,1-a;3,4-a']dinaphthalen-4-yl)dimethylamine ((*R*)-MonoPhos, 40 mol%). Purification *via* flash chromatography (23 g SiO$_2$, gradient from 98:02 to 80:20 *n*-hexane/EA over 15 CV) afforded **1ha** (92.0 mg, 386 µmol, 39%, mixture of isomers) as colorless crystals.

d.r.: 92:8

$C_{17}H_{18}O$ (238.33 $\frac{g}{mol}$)

R$_f$: 0.71 (*n*-hexane/EA = 80:20) [anisaldehyde]

^{1}H NMR(400.16 MHz, CDCl$_3$): δ = 7.21 (m, 1H, H-10), 7.17 (m, 2H, H-11/12), 7.07 (m, 1H, H-13), 3.88 (dd, ^3J = 9.6 Hz, ^3J = 5.9 Hz, 1H, H-15), 3.06 (s, 1H, H-7), 2.67 (m, 1H, H-2), 2.55 (s, 1H, H-8), 2.24 (ddt, ^2J = 20.7 Hz, ^3J = 9.6 Hz, ^3J = 7.3 Hz, 1H, H-16b), 1.88 (ddt, ^2J = 20.7 Hz, ^3J = 5.9 Hz, ^3J = 7.3 Hz, 1H, H-16a), 1.63 (dm, ^2J = 10.7 Hz, 1H, H-5a), 1.55 (dm, ^2J = 10.7 Hz, 1H, H-5b), 1.57 (m, 1H, H-6), 1.44 (m, 1H, H-4), 1.43 (m, 1H, H-4), 0.99 (t, ^3J = 7.3 Hz, 1H, H-17).

^{13}C NMR(100.62 MHz, CDCl$_3$): δ = 210.8 (C-1), 139.3 (C-9), 135.3 (C-14), 131.7 (C-10), 128.7 (C-13), 127.2 (C-12), 126.9 (C-11), 60.0 (C-15), 59.1 (C-2), 53.0 (C-7), 37.8 (C-8), 35.5 (C-5), 25.4 (C-16), 20.3 (C-4), 15.0 (C-6), 13.9 (C-3), 13.0 (C-17).

HRMS (ESI-TOF) m/z: $[M+H]^+$ Calcd for $C_{17}H_{18}OH$ 239.1430; Found 239.1431.

IR (ATR, ṽ): 1701 cm^{-1} (s, *CO*).

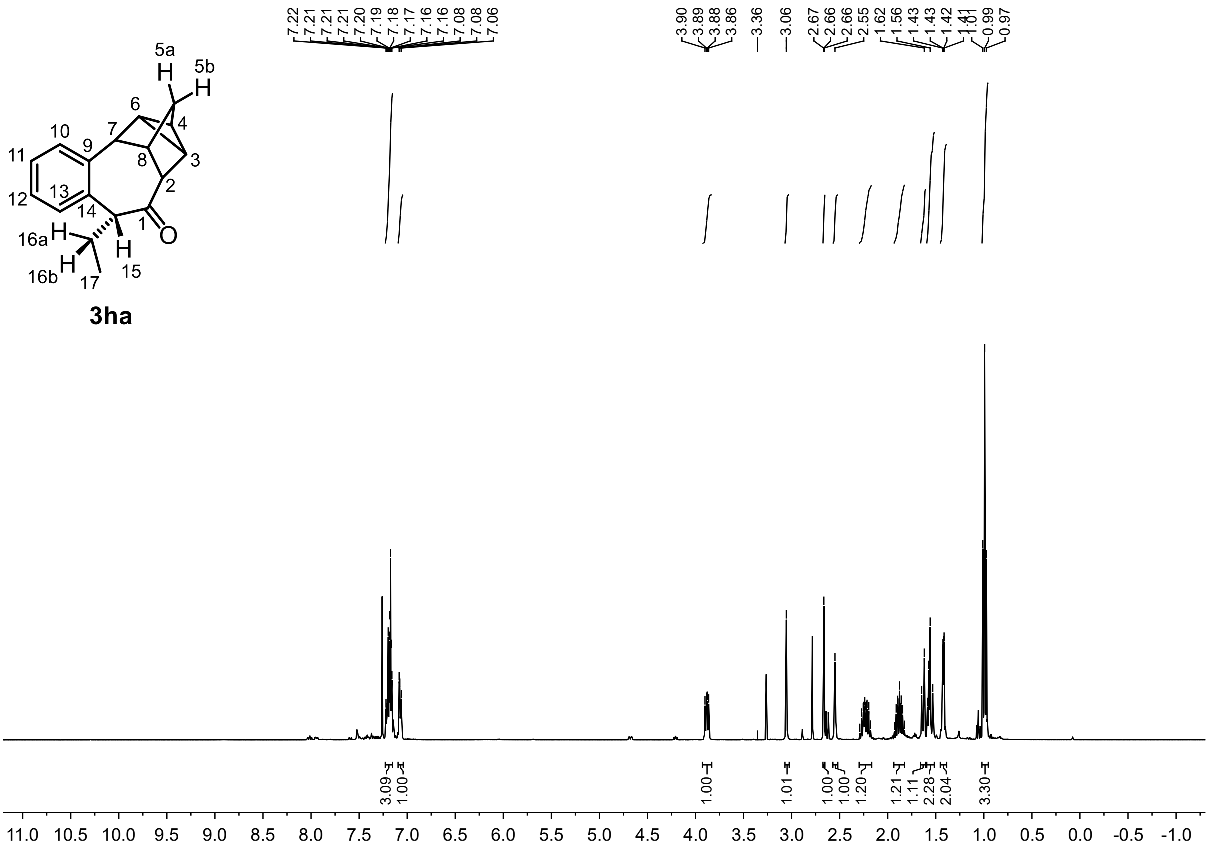

Figure S 104. ^{1}H NMR of **3ha** in $CDCl_3$ measured at 400.16 MHz. * denotes residual DMI.

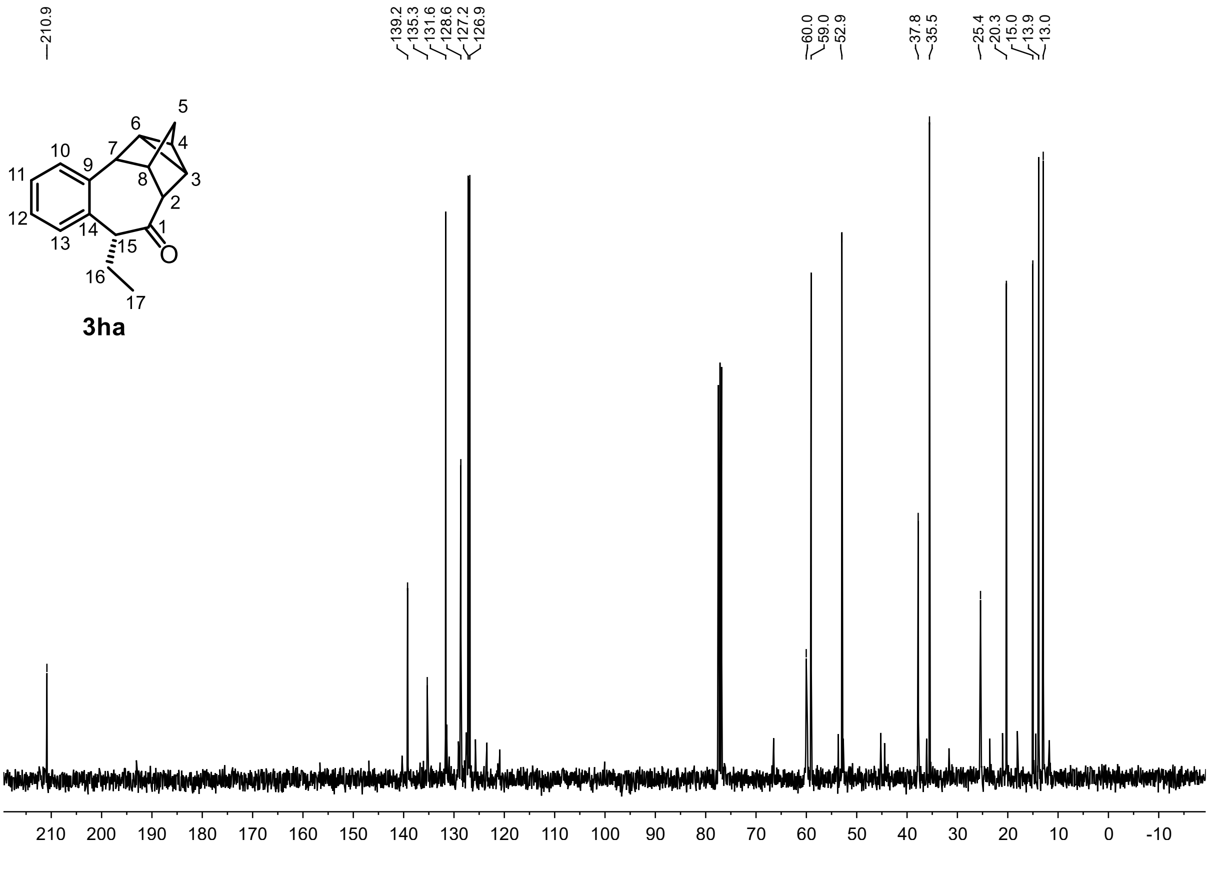

Figure S 105. ^{13}C NMR of **3ha** in $CDCl_3$ measured at 100.63 MHz.

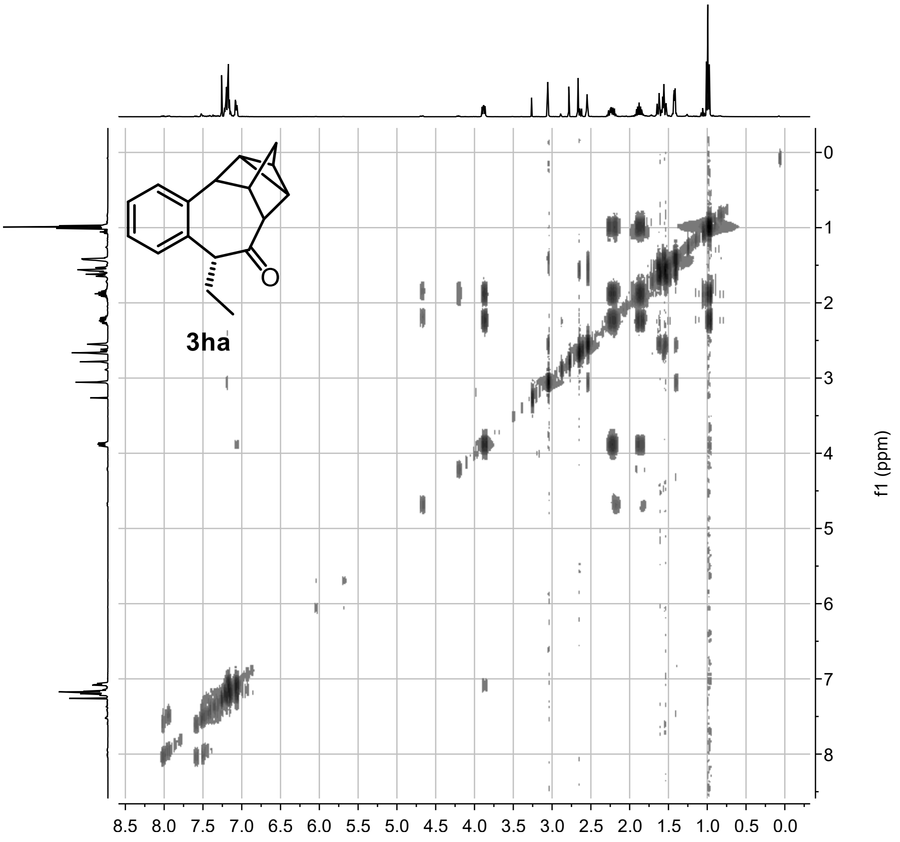

Figure S 106. $^{1}H,^{1}H$-COSY of **3ha** in $CDCl_3$ measured at 400.16 MHz.

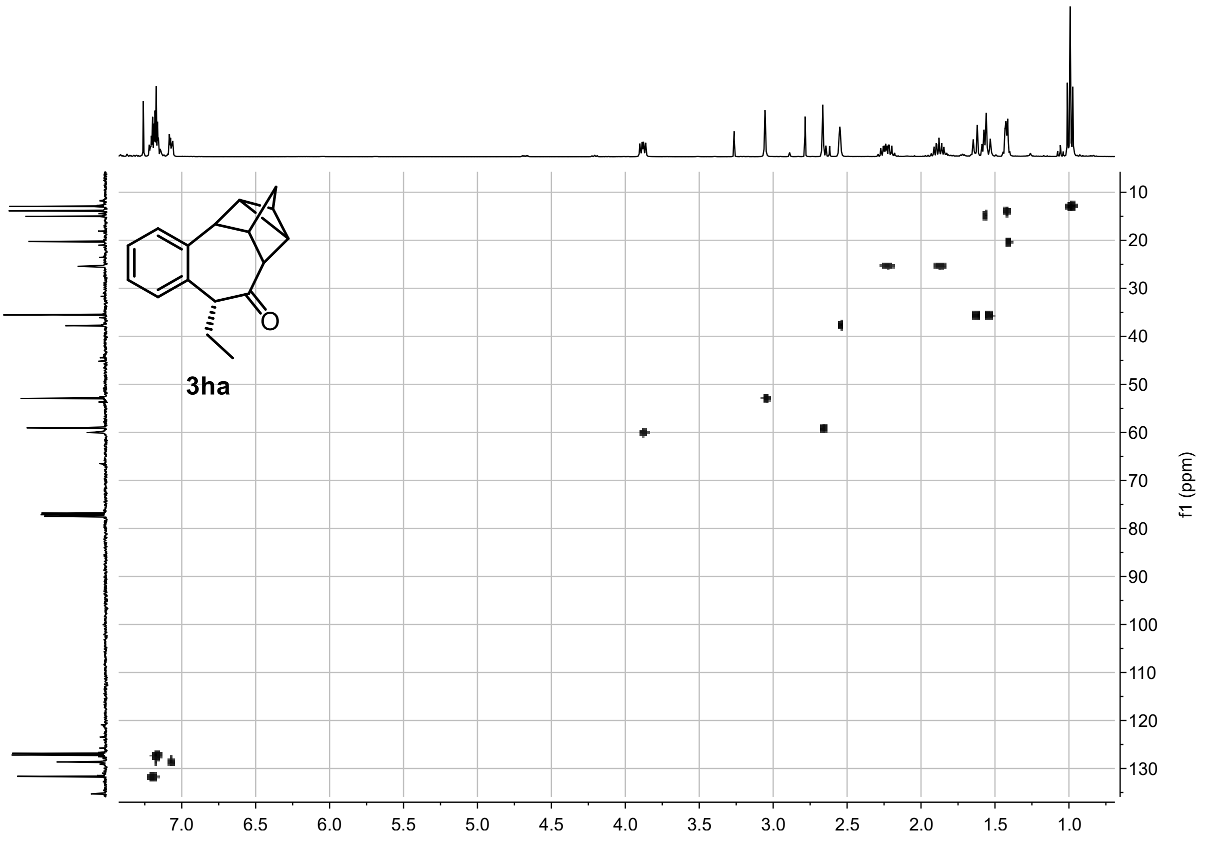

Figure S 107. $^{1}H,^{13}C$-HSQC of **3ha** in $CDCl_3$ measured at ^{1}H: 400.16 MHz; ^{13}C: 100.63 MHz.

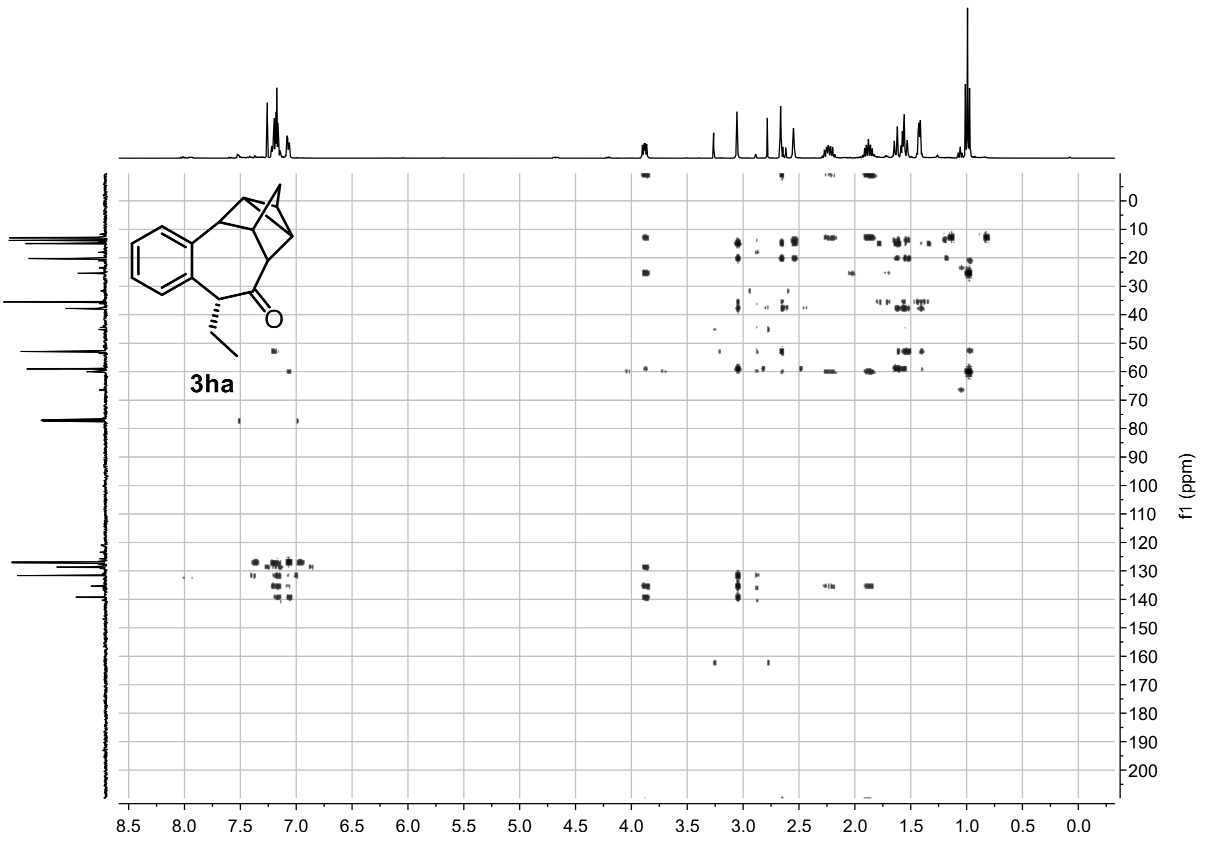

Figure S 108. $^{1}H,^{13}C$-HMBC of **3ha** in $CDCl_3$ measured at ^{1}H: 400.16 MHz; ^{13}C: 100.63 MHz.

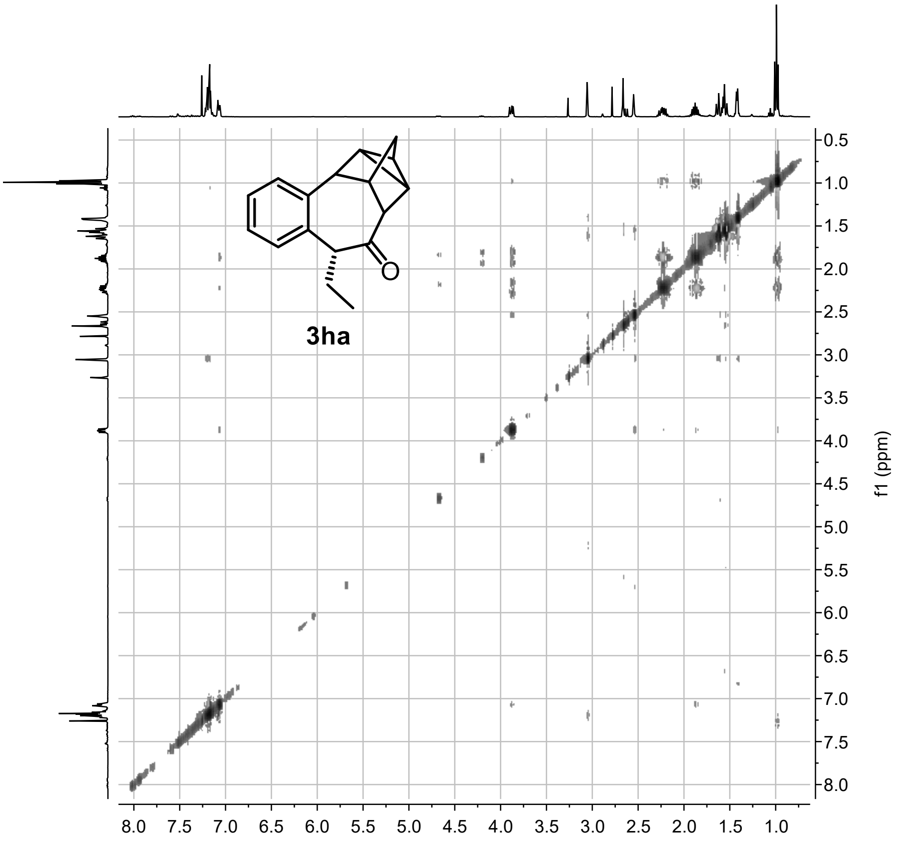

Figure S 109. $^{1}H,^{1}H$-NOESY of **3ha** in $CDCl_3$ measured at 400.16 MHz.

***rel*-(1*R*,2*R*,3a*S*,4*S*,9*R*,10a*R*,11*S*)-9-isopropyl-2,3,3a,4,9,10a-hexahydro-1,2,4-(epimethanetriyl)benzo[f]azulen-10(1H)-one**

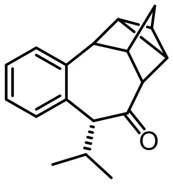

3ia

3ia was synthesized according to **GP-A** employing **1i** (160 mg, 1.00 mmol, 1.00 equiv.), NBD (305 µL, 3.00 mmol, 3.00 equiv.), Ni(COD)$_2$ (20 mol%) and (*R*)-(+)-(3,5-dioxa-4-phosphacyclohepta[2,1-a;3,4-a']dinaphthalen-4-yl)dimethylamine ((*R*)-MonoPhos, 40 mol%). Purification *via* flash chromatography (23 g SiO$_2$, gradient from 100:00 to 90:10 *n*-hexane/EA over 10 CV) afforded **3ia** (131 mg, 518 µmol, 52%, mixture of isomers) as a yellow solid.

d.r.: 96:4

$C_{18}H_{20}O$ (252.36 $\frac{g}{mol}$)

mp: 104.3 °C.

R$_f$: 0.67 (*n*-hexane/EA = 80:20) [anisaldehyde]

^{1}H NMR(400.16 MHz, CDCl$_3$): δ = 7.19 (m, 1H, H-10), 7.15 (m, 2H, H-11/12), 7.00 (m, 1H, H-13), 3.25 (d, ^3J = 11.1 Hz, 1H, H-15), 2.88 (s, 1H, H-7), 2.76 (m, 1H, H-2), 2.67 (dsep, ^3J = 11.1 Hz, ^3J = 6.5 Hz, 1H, H-16), 2.27 (s, 1H, H-8), 1.67 (m, 1H, H-3), 1.61 (dm, ^2J = 10.7 Hz, 1H, H-5a), 1.53 (m, 1H, H-6), 1.51 (dm, ^2J = 10.7 Hz, 1H, H-5b), 1.48 (m, 1H, H-4), 1.06 (d, ^3J = 6.5 Hz, 1H, H-18), 0.81 (d, ^3J = 6.5 Hz, 1H, H-17).

^{13}C NMR(100.62 MHz, CDCl$_3$): δ = 212.5 (C-1), 139.4 (C-9), 135.5 (C-14), 132.6 (C-13), 132.4 (C-10), 127.0 (C-11/12), 126.7 (C-12/11), 72.0 (C-15), 60.4 (C-2), 53.9 (C-7), 42.4 (C-8), 35.8 (C-5), 31.9 (C-16), 22.4 (C-18), 22.0 (C-17), 18.9 (C-6), 16.1 (C-3), 14.3 (C-4).

HRMS (ESI-TOF) m/z: [M+H]$^+$ Calcd for $C_{18}H_{20}OH$ 253.1587; Found 253.1589.

IR (ATR, ṽ): 1675 cm^{-1} (s, *CO*).

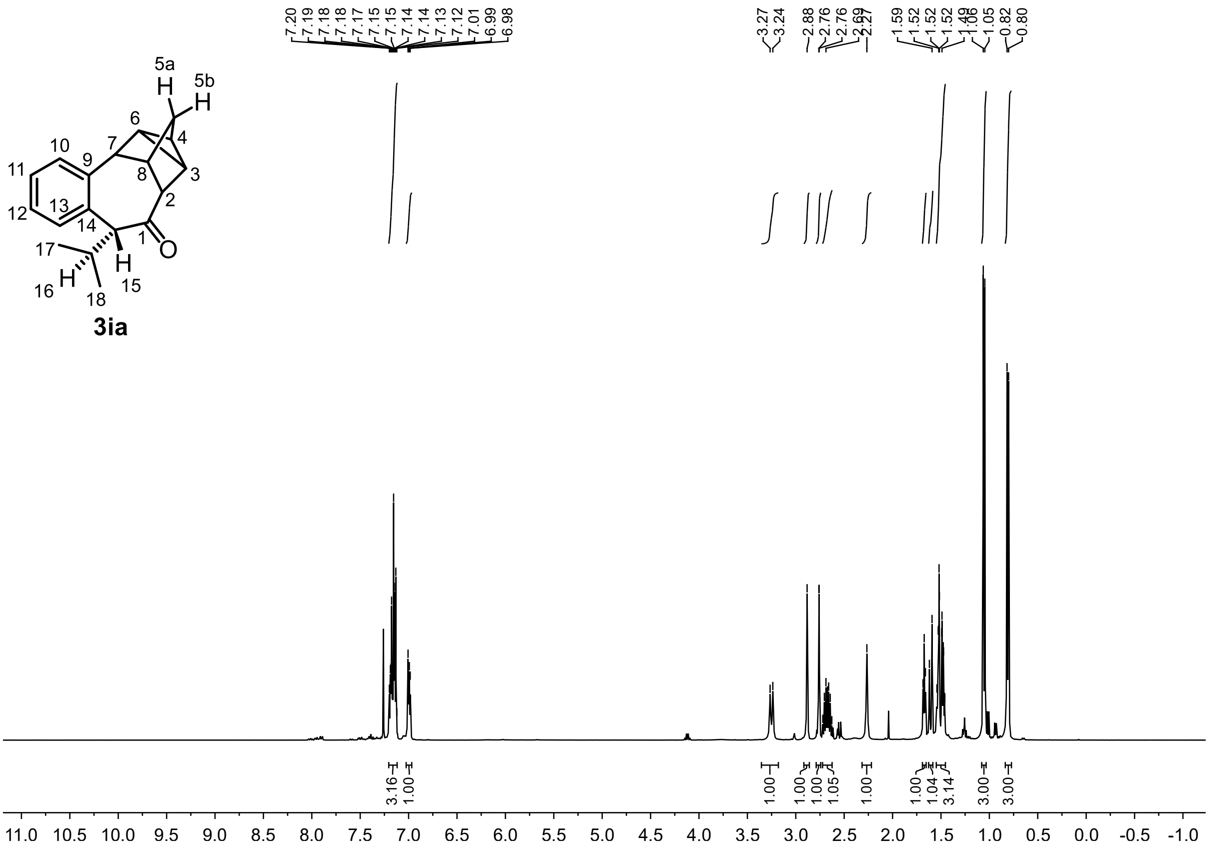

Figure S 110. ^{1}H NMR of **3ia** in $CDCl_3$ measured at 400.16 MHz.

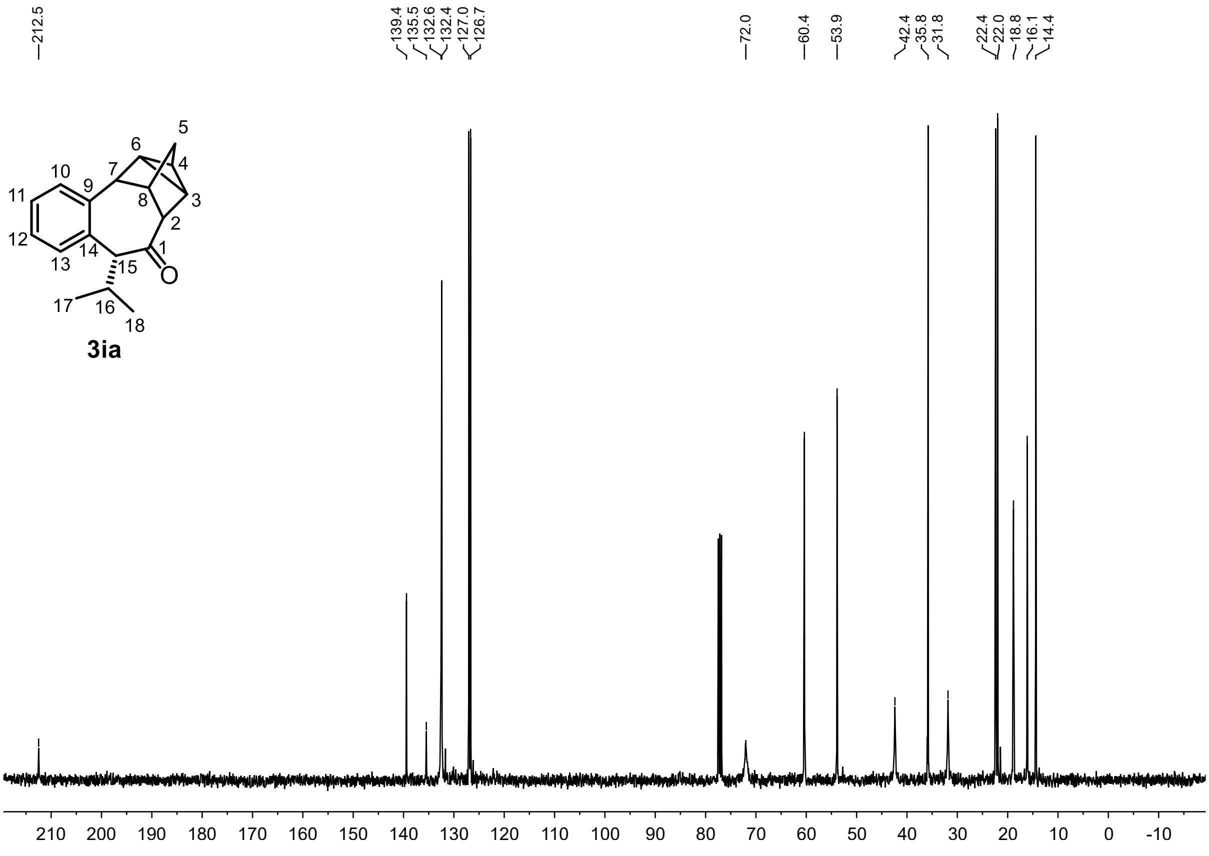

Figure S 111. ^{13}C NMR of **3ia** in $CDCl_3$ measured at 100.63 MHz.

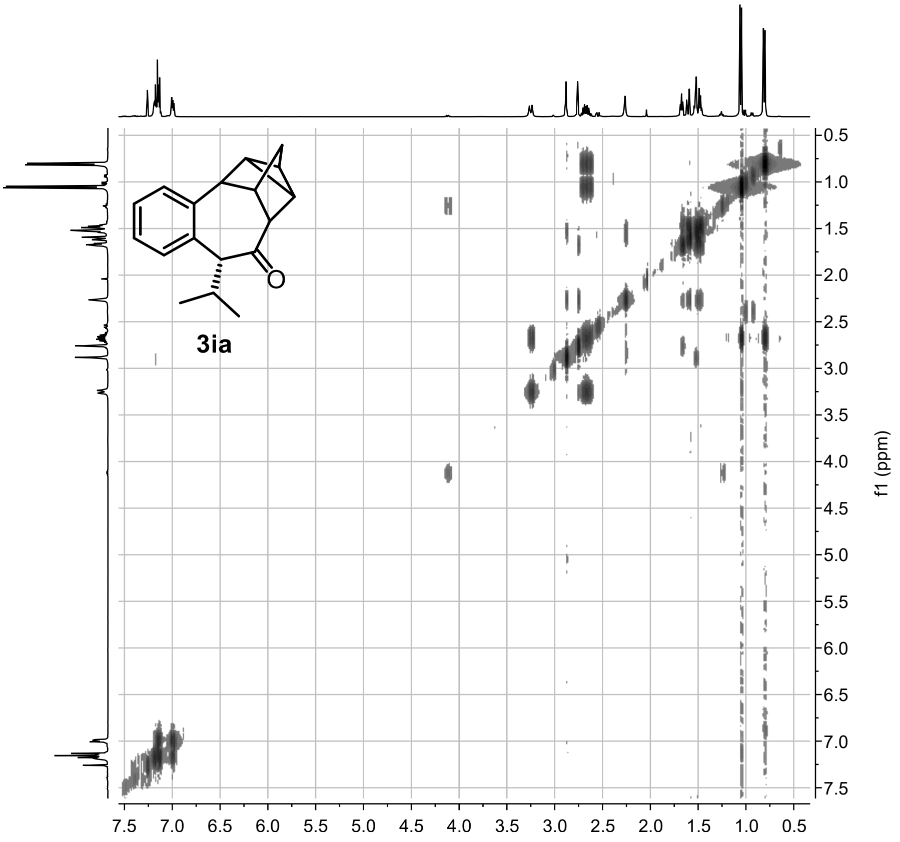

Figure S 112. $^{1}H,^{1}H$-COSY of **3ia** in $CDCl_3$ measured at 400.16 MHz.

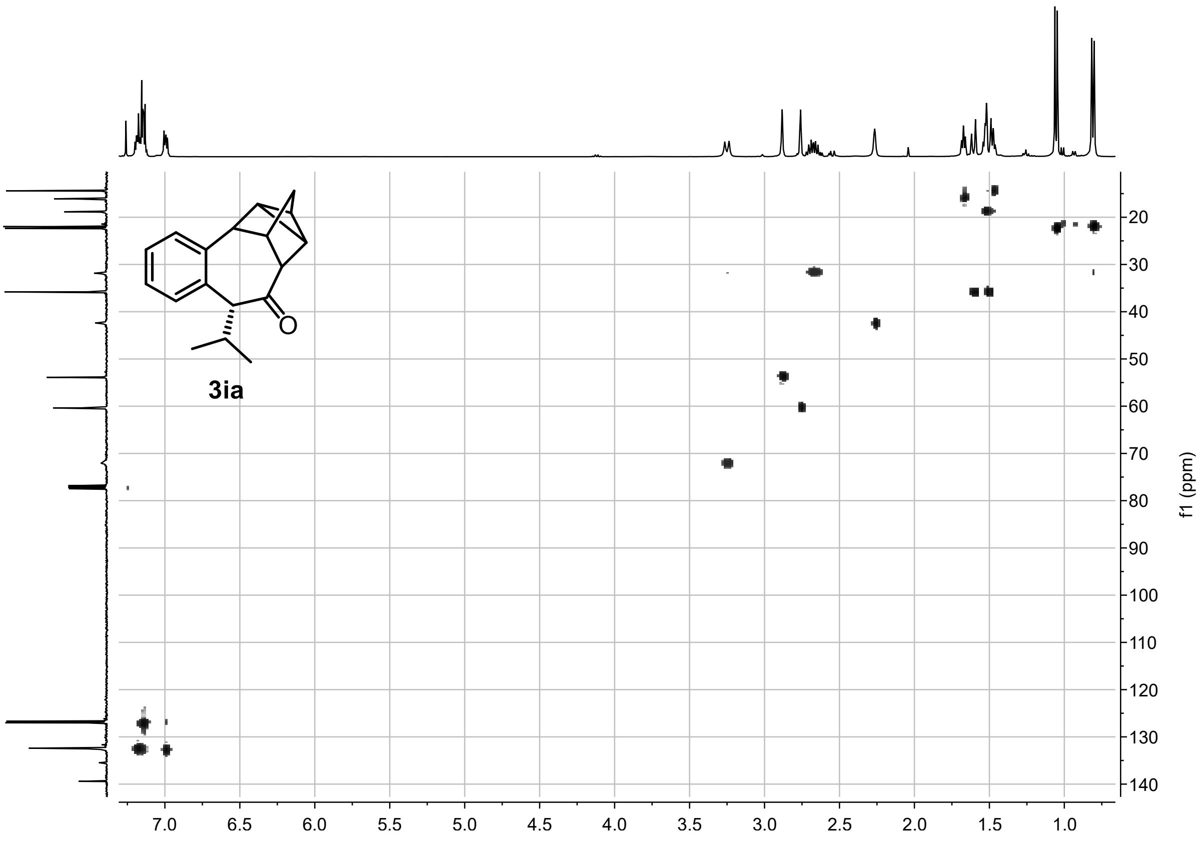

Figure S 113. $^{1}H,^{13}C$-HSQC of **3ia** in $CDCl_3$ measured at ^{1}H: 400.16 MHz; ^{13}C: 100.63 MHz.

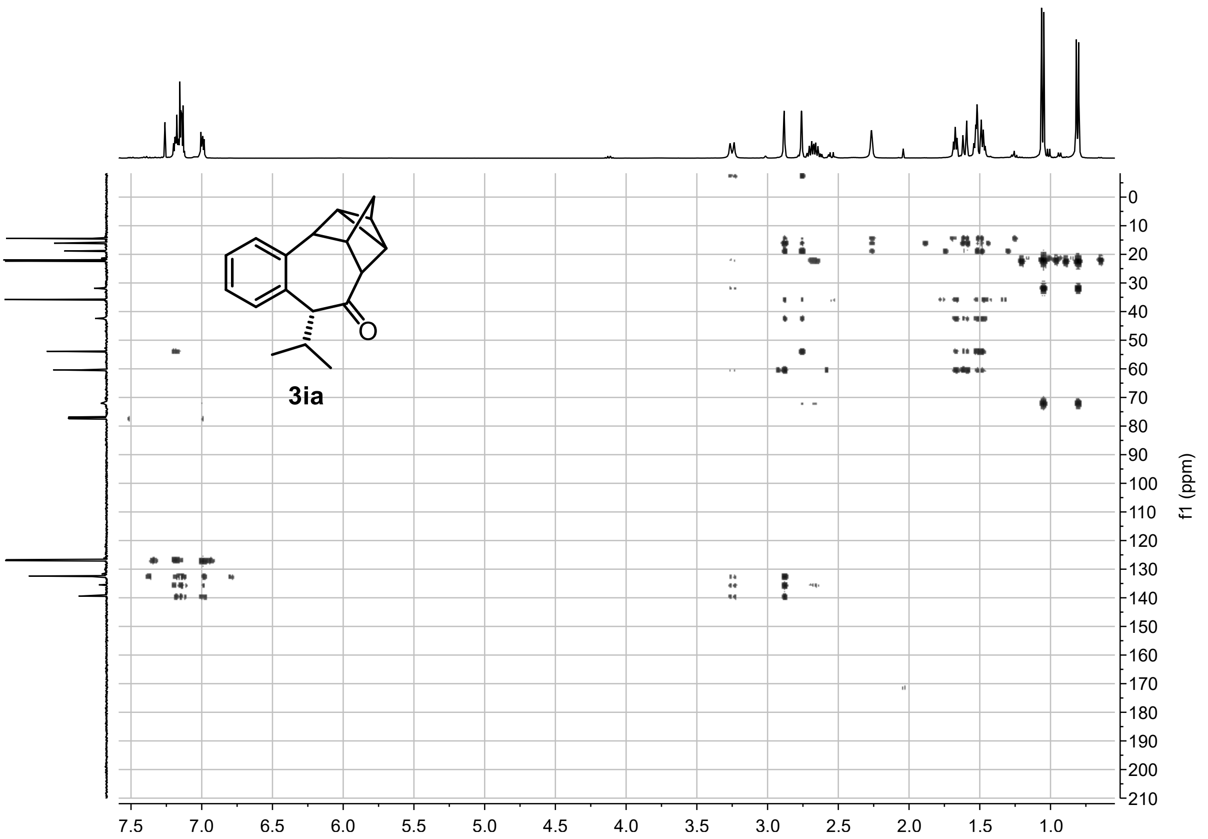

Figure S 114. ^{1}H,^{13}C-HMBC of **3ia** in $CDCl_3$ measured at ^{1}H: 400.16 MHz; ^{13}C: 100.63 MHz.

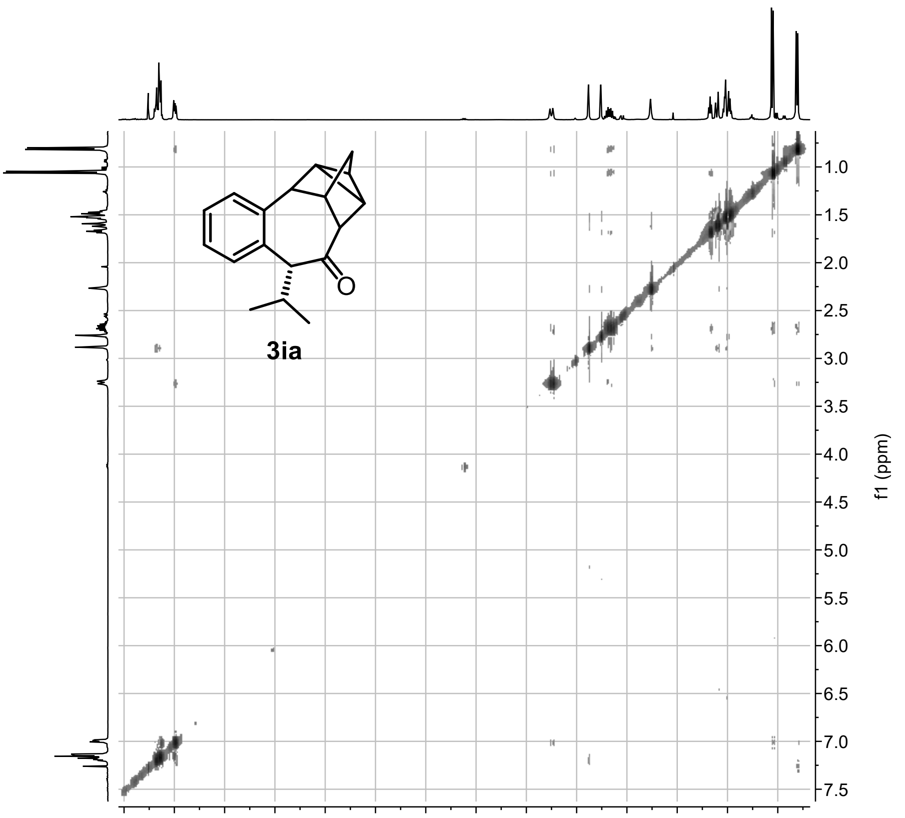

Figure S 115. ^{1}H,^{1}H-NOESY of **3ia** in $CDCl_3$ measured at 400.16 MHz.

***rel*-(1*R*,2*R*,3a*S*,4*S*,9*R*,10a*R*,11*S*)-9-allyl-2,3,3a,4,9,10a-hexahydro-1,2,4-(epimethanetriyl)benzo[f]azulen-10(1H)-one**

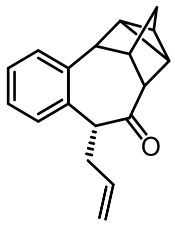

3ja

3ja was synthesized according to **GP-A** employing **1j** (158 mg, 1.00 mmol, 1.00 equiv.) and NBD (132 µL, 1.30 mmol, 1.30 equiv.) at 100 °C instead of 80 °C. Purification *via* flash chromatography (23 g SiO_2, gradient from 100:00 to 90:10 *n*-hexane/EA over 10 CV) afforded **3ja** (168 mg, 670 µmol, 67%, mixture of isomers) as a yellow oil.

d.r.: 93:7

$C_{18}H_{18}O$ (250.33 $\frac{g}{mol}$)

R_f: 0.80 (*n*-hexane/EA = 80:20) [anisaldehyde]

^{1}H NMR(400.16 MHz, $CDCl_3$): δ = 7.23 (m, 1H, H-10), 7.19 (m, 2H, H-11/12), 7.09 (m, 1H, H-13), 5.84 (dddd, ^3J = 17.1 Hz, ^3J = 10.2 Hz, ^3J = 6.7 Hz, ^3J = 6.7 Hz, 1H, H-17), 5.11 (dm, ^3J = 17.1 Hz, 1H, H-18b), 5.07 (dm, ^3J = 10.2 Hz, 1H, H-18a), 4.15 (dd, ^3J = 9.4 Hz, ^3J = 6.0 Hz, 1H, H-15), 3.11 (s, 1H, H-7), 2.95 (dddm, ^2J = 14.2 Hz, ^3J = 9.4 Hz, ^3J = 6.7 Hz, 1H, H-16b), 2.64 (m, 1H, H-2), 2.63 (m, 1H, H-8), 2.63 (ddm, ^2J = 14.2 Hz, ^3J = 6.0 Hz, 1H, H-16a), 1.64 (dm, ^2J = 10.6 Hz, 1H, H-5a), 1.59 (m, 1H, H-3), 1.56 (dm, ^2J = 10.6 Hz, 1H, H-5b), 1.43 (m, 1H, H-4), 1.41 (m, 1H, H-6); *diastereomer* δ = 7.25 – 7.16 (m, 3H, H-10, H-11, H-12), 7.15 (m, 1H, H-13), 5.84 (m, 1H, H-17), 5.15 (m, 1H, H-18b), 5.07 (m, 1H, H-18a), 4.89 (dd, ^3J = 10.0 Hz, ^3J = 5.0 Hz, 1H, H-15), 2.92 (m, 1H, H-16a/b), 2.90 (s, 1H, H-7), 2.67 (m, 1H, H-2), 2.56 (m, 1H, H-16b/a), 2.24 (m, 1H, H-8), 1.74 (m, 1H, H-3) 1.65 (dm, ^2J = 10.7 Hz, 1H, H-5a), 1.58 (m, 1H, H-4/6), 1.51 (dm, ^2J = 10.7 Hz, 1H, H-5b), 1.43 (m, 1H, H-6/4).

^{13}C NMR(100.62 MHz, $CDCl_3$): δ = 209.6 (C-1), 139.3 (C-9), 136.2 (C-17), 134.1 (C-14), 131.5 (C-10), 127.9 (C-13), 127.1 (C-11/12), 127.0 (C-12/11), 116.9 (C-18), 58.5 (C-2), 56.4 (C-15), 52.7 (C-7), 36.8 (C-8), 35.4 (C-5), 35.1 (C-16), 20.6 (C-6), 14.6 (C-3), 13.8 (C-4); *diastereomer* δ = 210.6 (C-1), 140.4 (C-9), 136.5 (C-17), 135.3 (C-14), 131.5 (C-10), 127.6

(C-11/12), 126.8 (C-12/11), 125.5 (C-13), 116.9 (C-18), 59.7 (C-2), 53.7 (C-2), 50.2 (C-15), 44.7 (C-8), 36.1 (C-5), 31.7 (C-16), 18.0 (C-3), 14.4 (C-4/6), 13.7 (C-6/4).

HRMS (ESI-TOF) m/z: $[M+H]^+$ Calcd for $C_{18}H_{18}OH$ 251.1430; Found 251.1433.

IR (ATR, ṽ): 1705 cm^{-1} (s, *CO*).

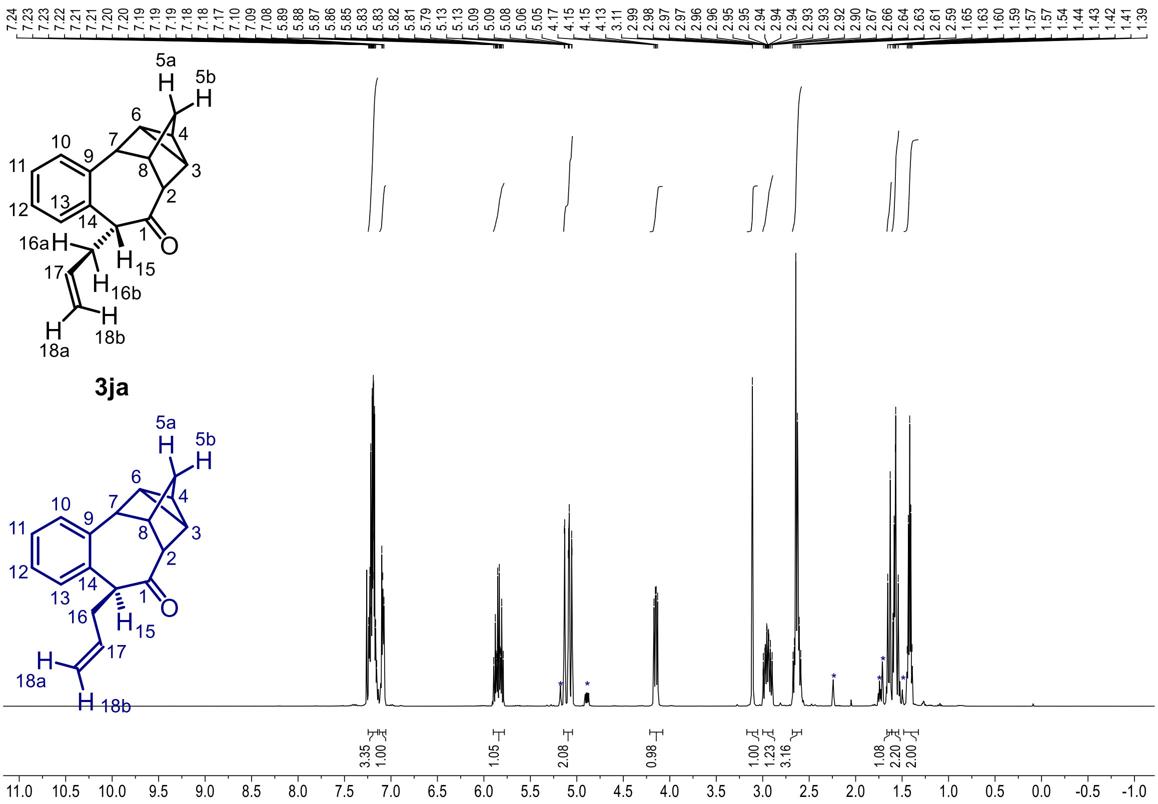

Figure S 116. ^{1}H NMR of **3ja** in $CDCl_3$ measured at 400.16 MHz. * Denotes signals corresponding to the diastereomer marked in blue.

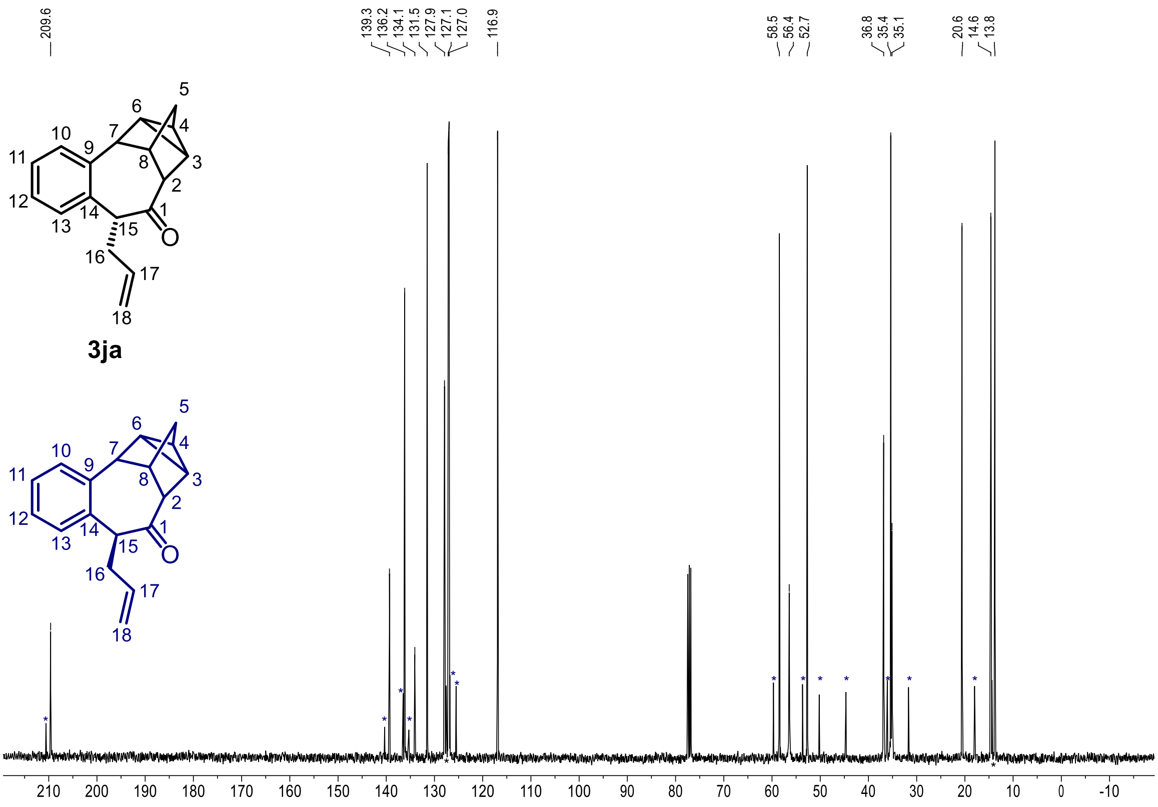

Figure S 117. ^{13}C NMR of **3ja** in $CDCl_3$ measured at 100.63 MHz. * Denotes signals corresponding to the diastereomer marked in blue.

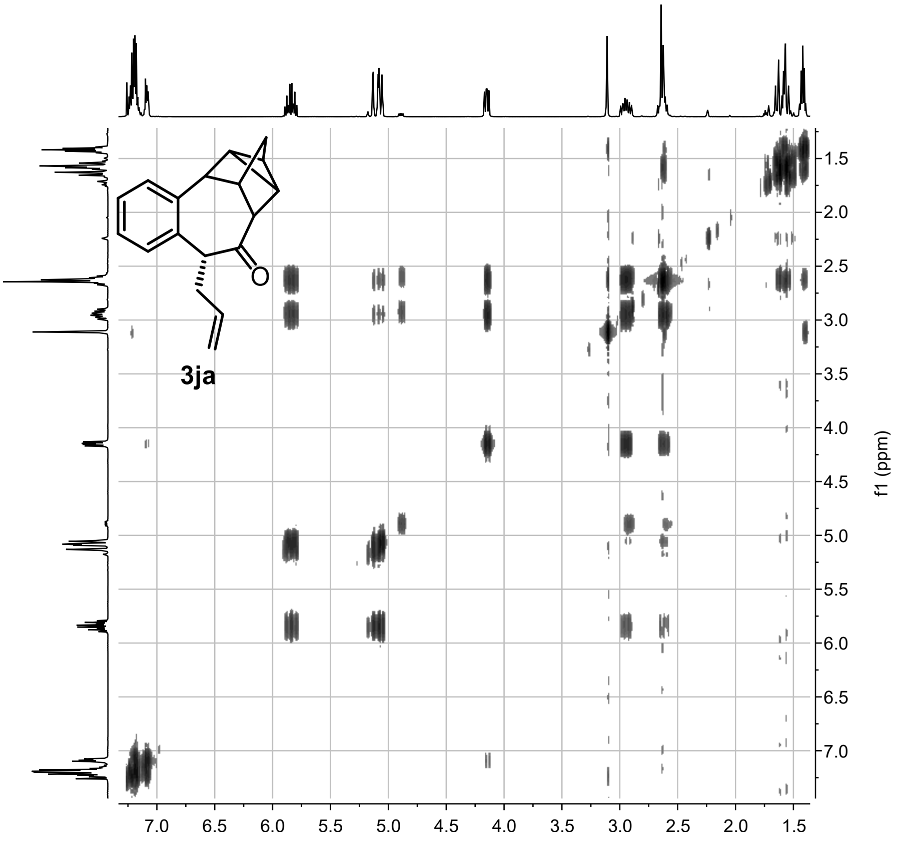

Figure S 118. $^{1}H,^{1}H$-COSY of **3ja** and its respective diastereomer in $CDCl_3$ measured at 400.16 MHz.

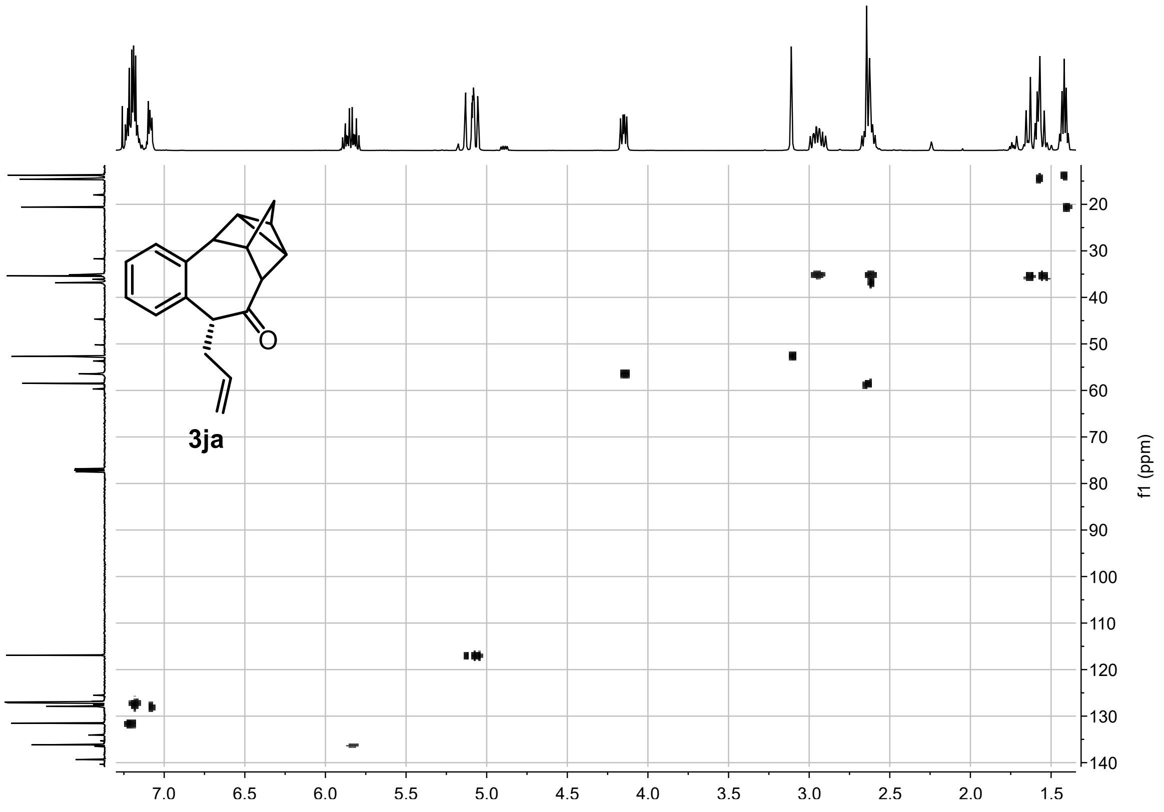

Figure S 119. $^{1}H,^{13}C$-HSQC of **3ja** and its respective diastereomer in $CDCl_3$ measured at ^{1}H: 400.16 MHz; ^{13}C: 100.63 MHz.

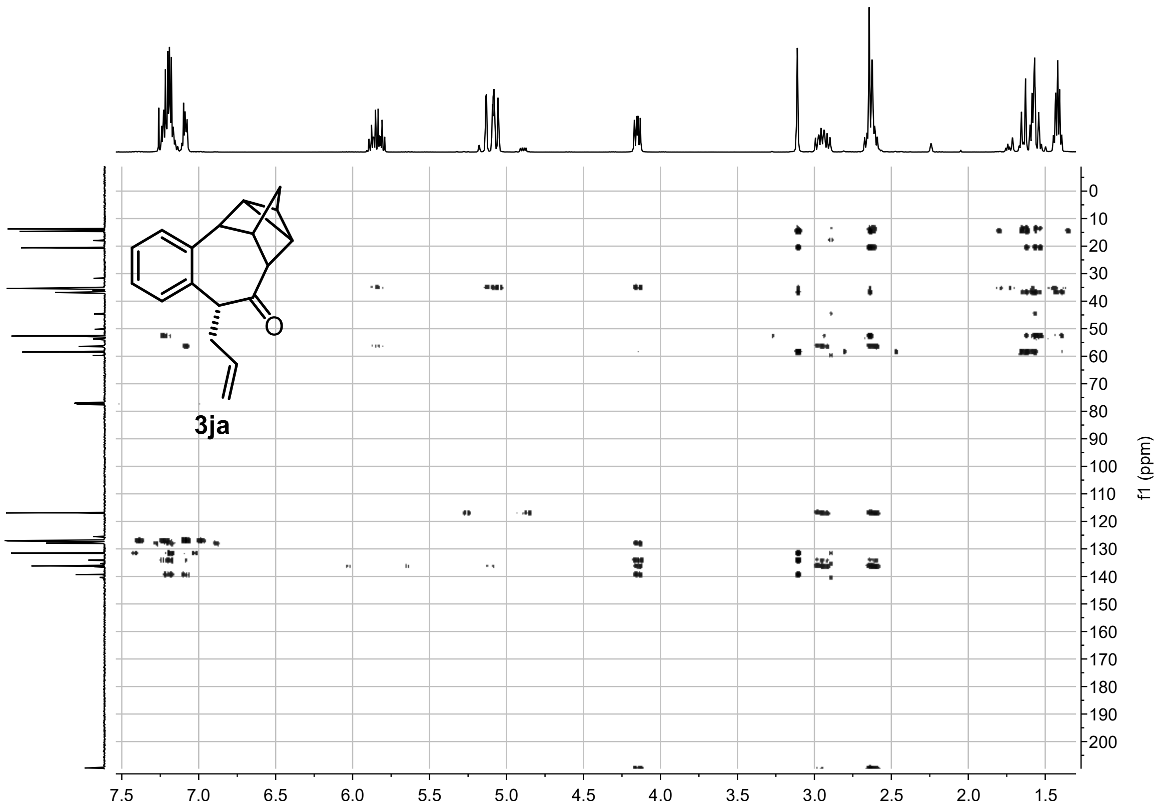

Figure S 120. $^{1}H,^{13}C$-HMBC of **3ja** and its respective diastereomer in $CDCl_3$ measured at ^{1}H: 400.16 MHz; ^{13}C: 100.63 MHz.

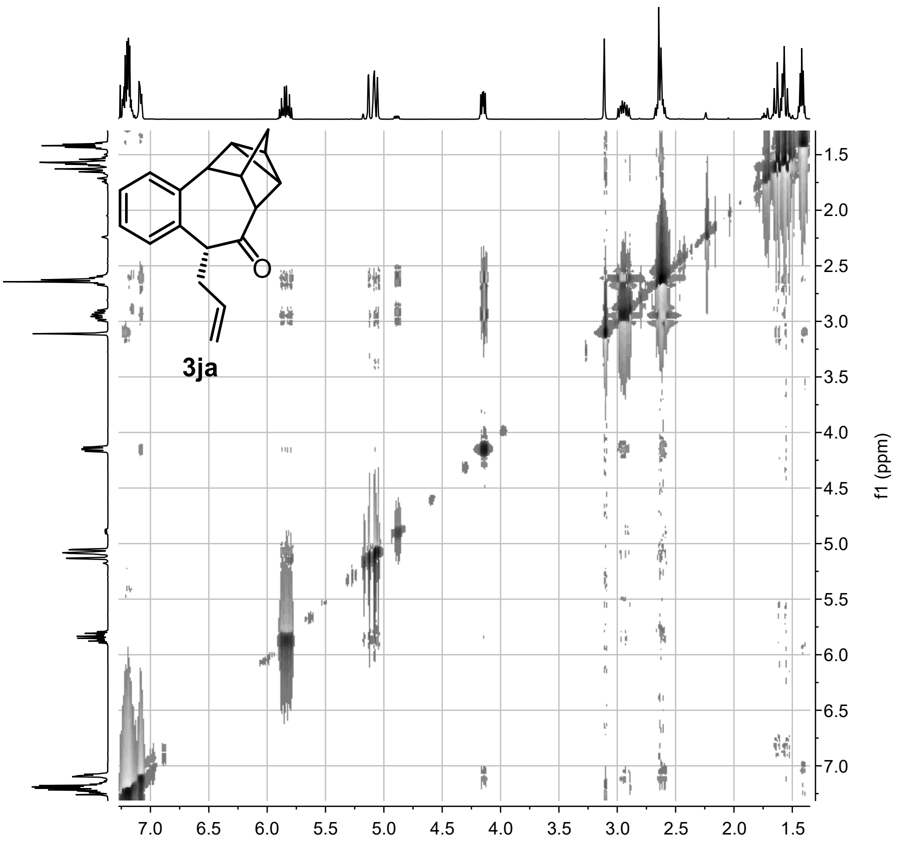

Figure S 121. $^{1}H,^{1}H$-NOESY of **3ja** and its respective diastereomer in $CDCl_3$ measured at 400.16 MHz.

***rel*-(1'*S*,2'*S*,3a'*R*,4'*R*,10a'*S*,11'*R*)-1',2',3',3a',4',10a'-hexahydro-10'H-spiro[cyclopropane-1,9'-[1,2,4](epimethanetriyl) benzo[f]azulen]-10'-one**

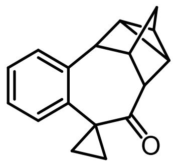

3ka

3ka was synthesized according to **GP-A** employing **1k** (144 mg, 1.00 mmol, 1.00 equiv.) and NBD (132 µL, 1.30 mmol, 1.30 equiv.). Purification *via* flash chromatography (23 g SiO_2, gradient from 100:00 to 90:10 *n*-hexane/EA over 10 CV) afforded **3ka** (127 mg, 537 µmol, 54%) as a pale yellow solid.

$C_{17}H_{16}O$ (236.31 $\frac{g}{mol}$)

mp: 86.1 °C.

R_f: 0.73 (*n*-hexane/EA = 80:20) [anisaldehyde]

^{1}H NMR(600.13 MHz, $CDCl_3$): δ = 7.22 (dd, ^3J = 7.4 Hz, ^4J = 0.7 Hz, 1H, H-10,), 7.03 (td, ^3J = 7.2 Hz, ^4J = 1.3 Hz, 1H, H-11), 7.15 (td, ^3J = 7.6 Hz, ^4J = 1.5 Hz, 1H, H-12), 7.10 (d, ^3J = 7.6 Hz, 1H, H-13), 3.09 (s, 1H, H-7), 2.70 (s, 1H, H-2), 2.60 (s, 1H, H-8), 1.65 (d, ^2J = 10.7 Hz, 1H, H-5a), 1.57 (m, 1H, H-3), 1.53 (d, ^2J = 10.7 Hz, 1H, H-5b), 1.50 (m, 1H, H-6), 1.41 (m, 1H, H-4), 1.37 (m, 1H, H-17b), 1.23 (m, 1H, H-17a), 1.21 (m, 2H, H-16).

^{13}C NMR(150.90 MHz, $CDCl_3$): δ = 209.0 (C-1), 141.6 (C-9), 134.5 (C-14), 131.5 (C-10), 127.4 (C-13), 127.2 (2C, C-11, C-12), 58.8 (C-2), 53.4 (C-7), 38.0 (C-8), 36.5 (C-15), 35.9 (C-5), 18.6 (C-6), 17.3 (C-3), 13.7 (C-17), 13.1 (C-4), 12.9 (C-16).

HRMS (ESI-TOF) m/z: $[M+Na]^+$ Calcd for $C_{17}H_{16}ONa$ 259.1093; Found 259.1098.

IR (ATR, ṽ): 1687 cm^{-1} (s, *CO*).

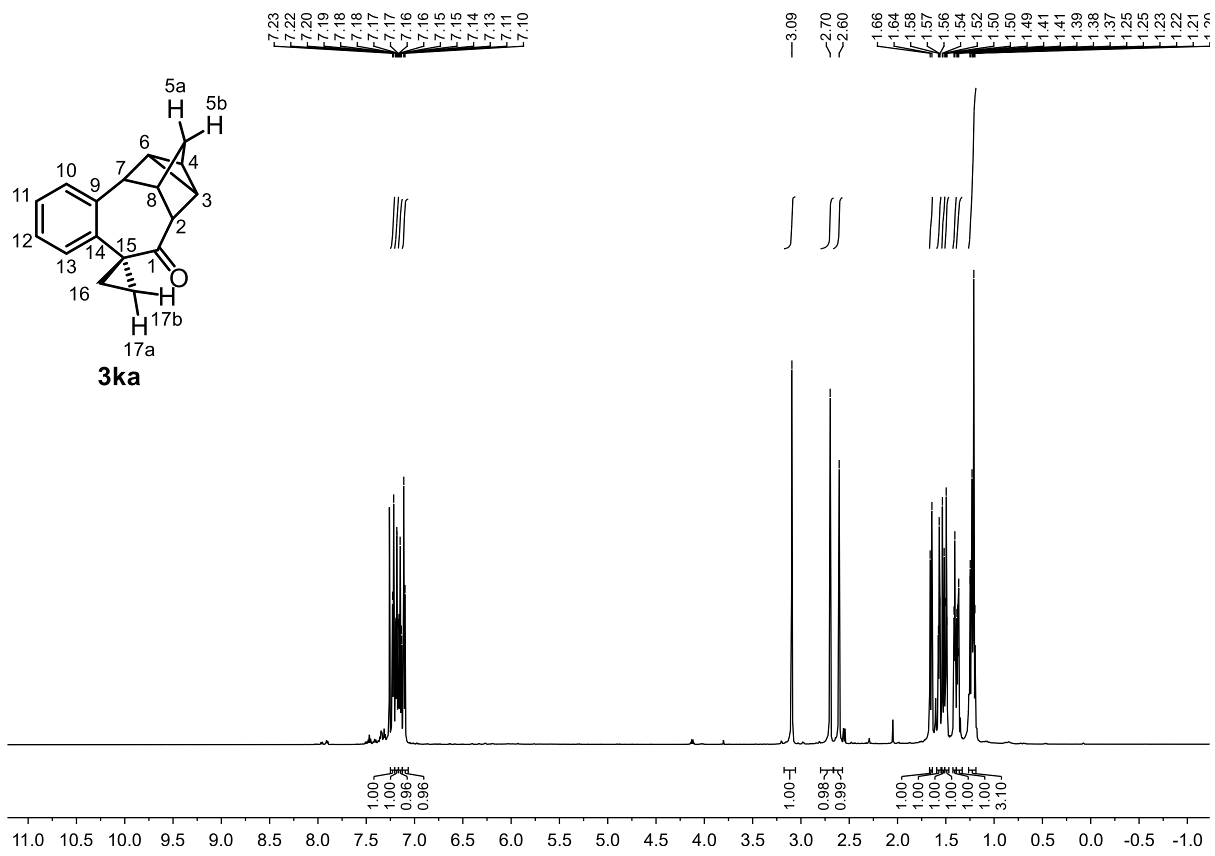

Figure S 122. ^{1}H NMR of **3ka** in $CDCl_3$ measured at 600.13 MHz.

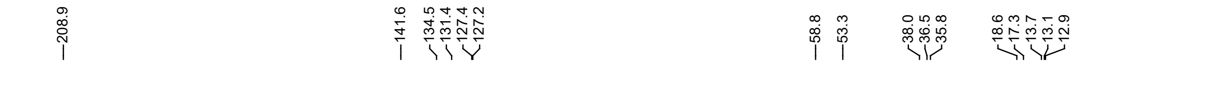

Figure S 123. ^{13}C NMR of **3ka** in $CDCl_3$ measured at 150.92 MHz.

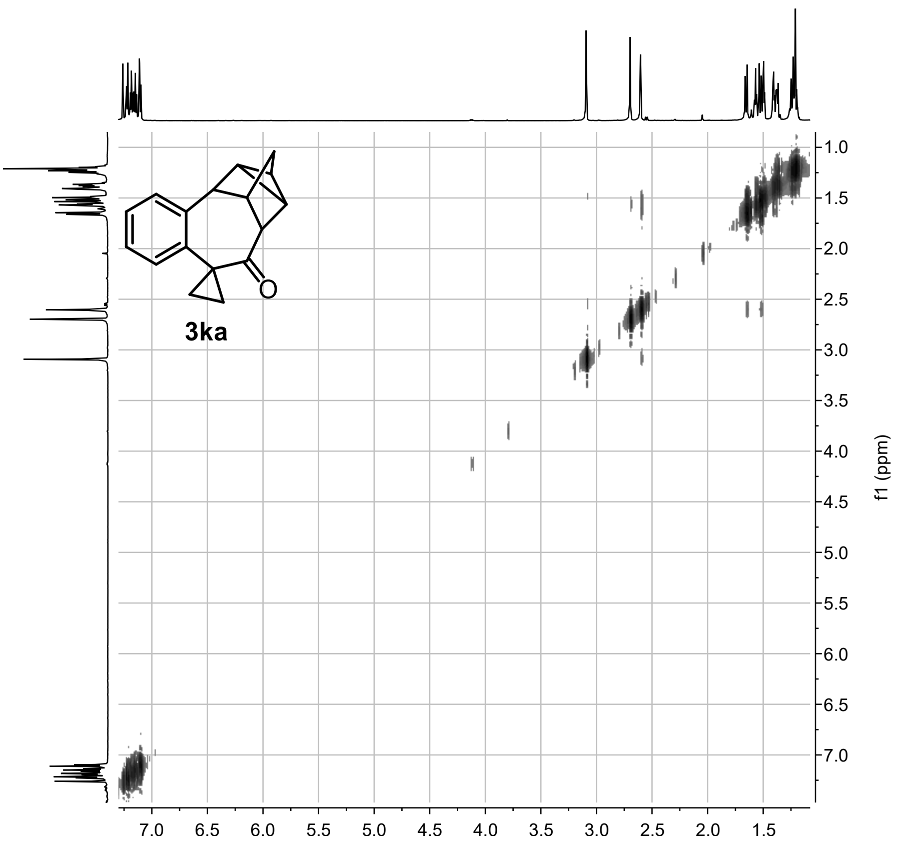

Figure S 124. ^{1}H,^{1}H-COSY of **3ka** in $CDCl_3$ measured at 600.13 MHz.

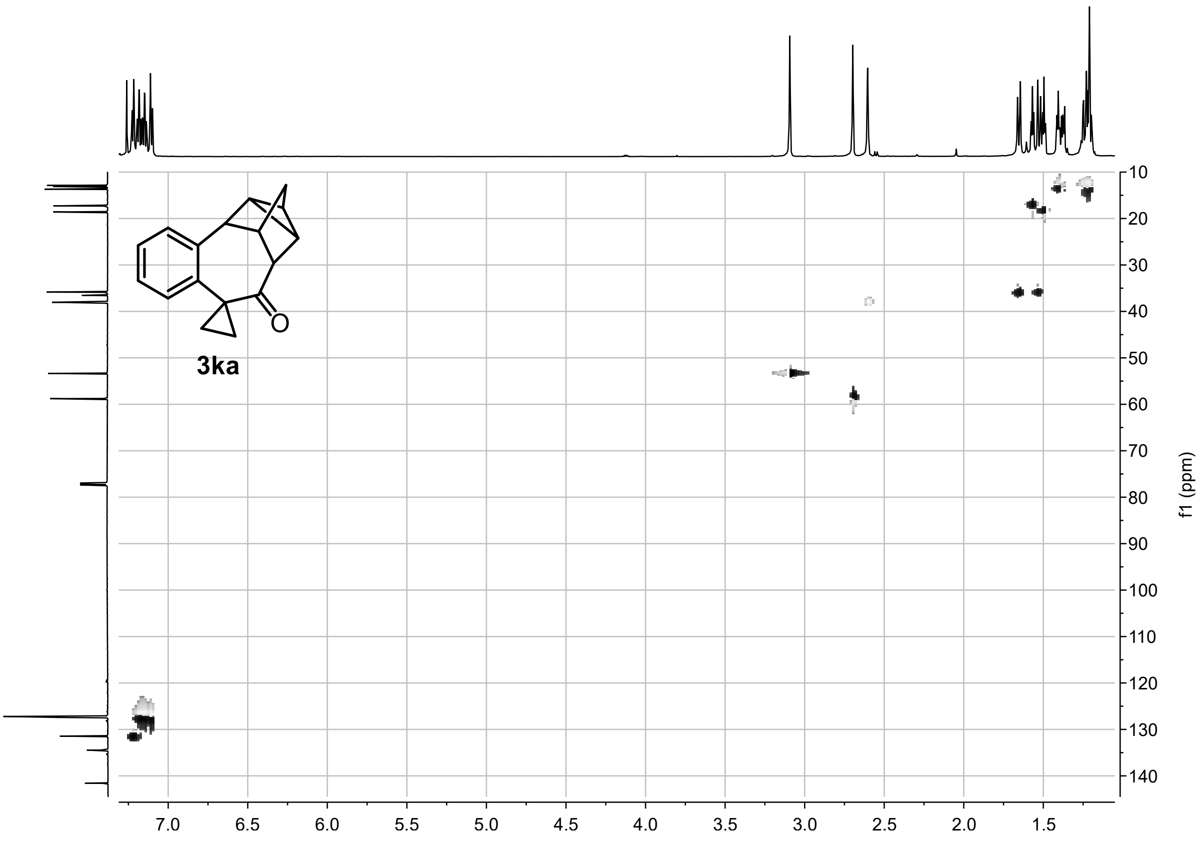

Figure S 125. ^{1}H,^{13}C-HSQC of **3ka** in $CDCl_3$ measured at ^{1}H: 600.13 MHz; ^{13}C: 150.92 MHz..

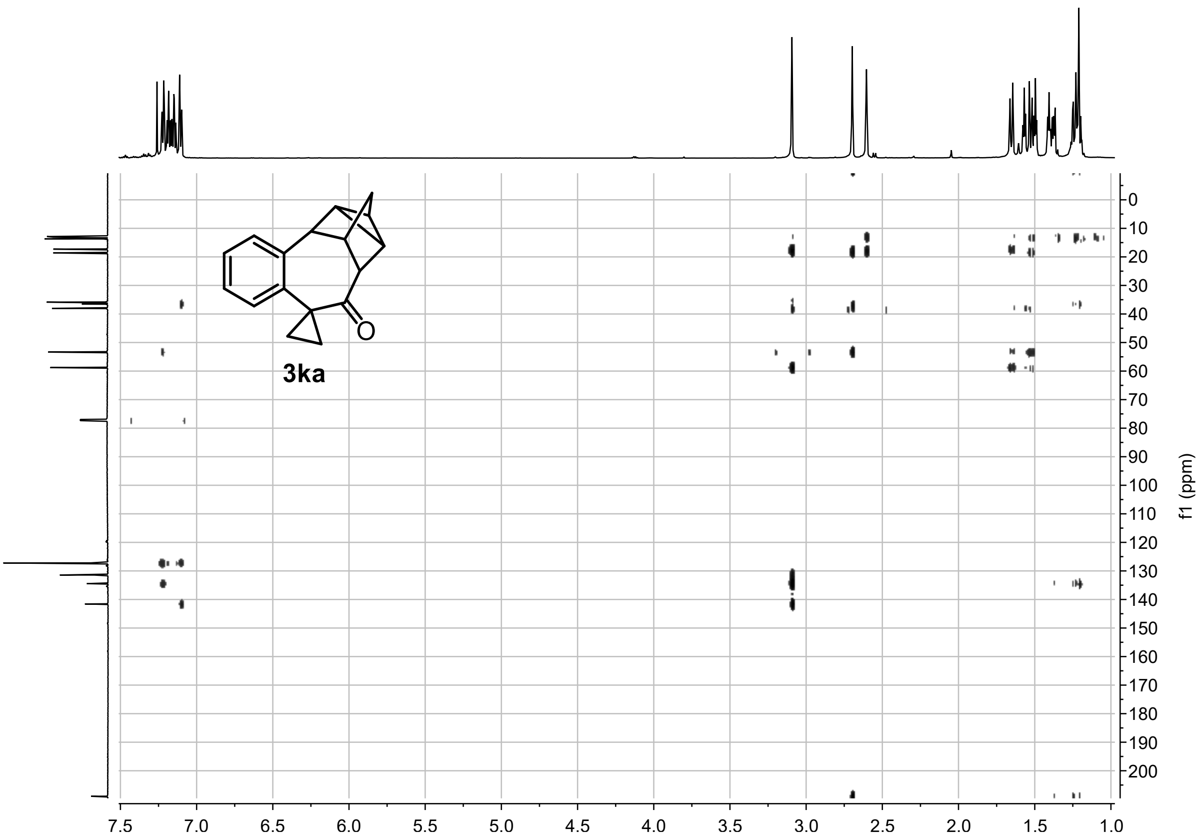

Figure S 126. $^{1}H,^{13}C$-HMBC of **3ka** in $CDCl_3$ measured at ^{1}H: 600.13 MHz; ^{13}C: 150.92 MHz.

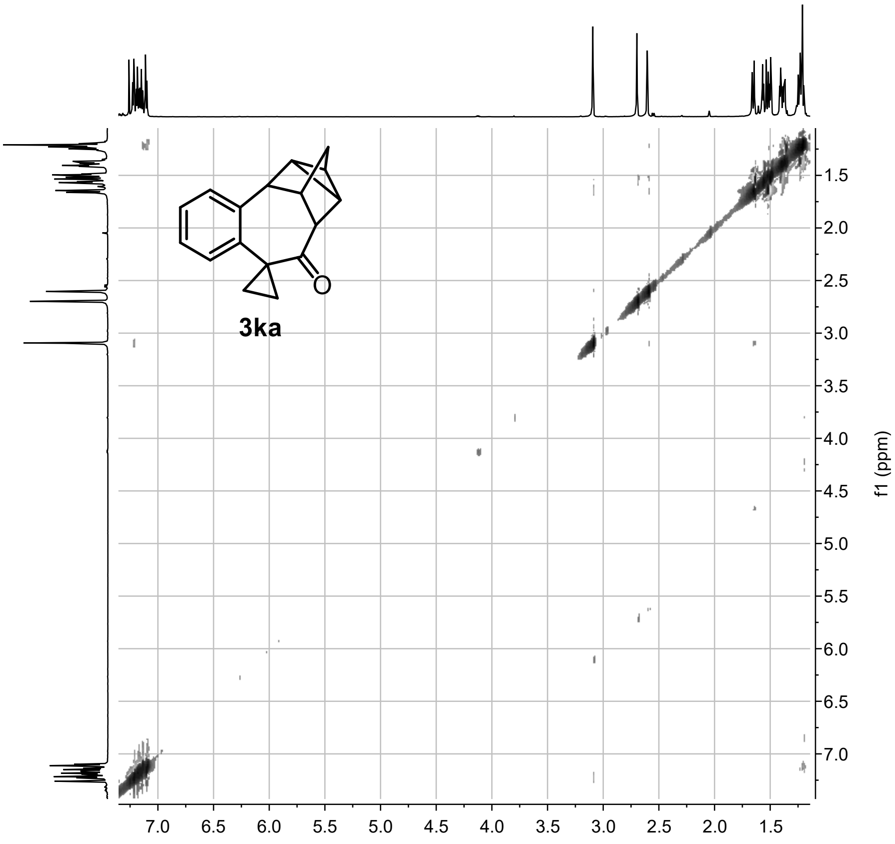

Figure S 127. $^{1}H,^{1}H$-NOESY of **3ka** in $CDCl_3$ measured at 600.13 MHz.

***rel*-(1*R*,2*R*,3a*S*,4*S*,9*R*,10a*R*,11*S*)-6-isobutyl-9-methyl-2,3,3a,4,9,10a-hexahydro-1,2,4-(epimethanetriyl)benzo[f]azulen-10(1H)-one**

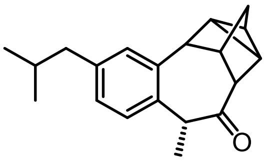

3la

3la was synthesized according to **GP-A** employing **1l** (188 mg, 1.00 mmol, 1.00 equiv.) and NBD (132 µL, 1.30 mmol, 1.30 equiv.). Purification *via* flash chromatography (23 g SiO_2, gradient from 100:00 to 85:15 *n*-hexane/EA over 15 CV) afforded **1la** (202 mg, 721 µmol, 72%, mixture of isomers) as a yellow oil.

d.r.: 88:12

$C_{20}H_{24}O$ (280.41 $\frac{g}{mol}$)

R_f: 0.79 (*n*-hexane/EA = 80:20) [anisaldehyde]

^{1}H NMR(400.16 MHz, $CDCl_3$): δ = 7.05 (m, 1H, H-13), 7.01 (m, 1H, H-10), 6.99 (m, 1H, H-12), 4.25 (q, ^3J = 6.9 Hz, 1H, H-15), 3.14 (s, 1H, H-7), 2.71 (m, 1H, H-8), 2.61 (m, 1H, H-2), 2.43 (m, 2H, H-17), 1.85 (sep, ^3J = 6.7 Hz, 1H, H-18), 1.65 (dm, ^2J = 10.5 Hz, 1H, H-5a), 1.55 (dm, ^2J = 10.5 Hz, 1H, H-5b), 1.52 (m, 1H, H-3), 1.47 (d, ^3J = 6.9 Hz, 3H, H-16), 1.39 (m, 1H, H-4), 1.36 (m, 1H, H-6), 0.92 (d, ^3J = 3.9 Hz, 3H, H-19/20), 0.90 (d, ^3J = 3.9 Hz, 3H, H-20/19); *diastereomer* δ = 7.11 (m, 1H, H-13), 7.00 (m, 1H, H-12), 6.95 (m, 1H, H-10), 4.81 (q, ^3J = 6.8 Hz, 1H, H-15), 2.87 (s, 1H, H-7), 2.69 (m, 1H, H-2), 2.43 (m, 2H, H-17), 2.22 (m, 1H, H-8), 1.83 (m, 1H, H-18), 1.74 (m, 1H, H-3), 1.65 (m, 1H, H-5a), 1.54 (m, 2H, H-4, H-6), 1.51 (m, 1H, H-5b), 1.42 (d, ^3J = 6.9 Hz, 3H, H-16), 0.92 (m, 3H, H-19/20), 0.90 (m, 3H, H-20/19).

^{13}C NMR(100.62 MHz, $CDCl_3$): δ = 210.7 (C-1), 140.1 (C-11), 138.9 (C-9), 131.9 (C-10), 131.7 (C-14), 127.9 (C-12), 126.3 (C-13), 57.5 (C-2), 52.5 (C-7), 48.0 (C-15), 44.8 (C-17), 34.2 (C-8), 35.2 (C-5), 30.2 (C-18), 22.5 (C-19/20), 22.4 (C-20/19), 21.2 (C-6), 14.8 (C-16), 14.4 (C-3), 13.3 (C-4) *diastereomer* δ = 211.9 (C-1), 140.0 (C-11), 139.8 (C-9), 133.2 (C-14), 132.4 (C-10), 128.1 (C-12), 124.9 (C-13), 59.7 (C-2), 53.8 (C-7), 44.1 (C-8), 43.8 (C-15), 43.4 (C-17), 36.2 (C-5), 29.1 (C-18), 22.5 (C-19/20), 22.4 (C-20/19), 18.1 (C-3), 14.0 (C-6), 13.7 (C-4), 13.0 (C-16).

HRMS (ESI-TOF) m/z: $[M+H]^+$ Calcd for $C_{20}H_{24}OH$ 281.1900; Found 281.1903.

IR (ATR, ṽ): 1705 cm^{-1} (s, *CO*).

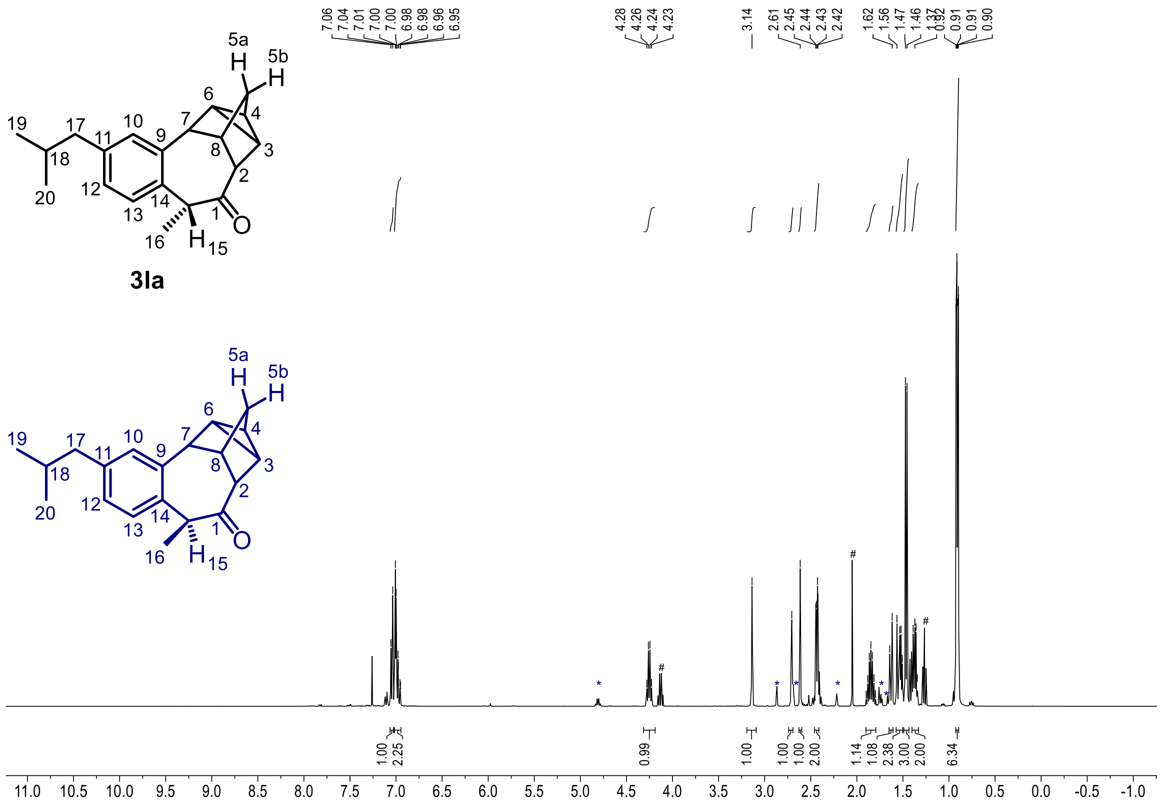

Figure S 128. ^{1}H NMR of **3la** in $CDCl_3$ measured at 400.16 MHz. * Denotes signals of the diastereomer. # Denotes residual EA.

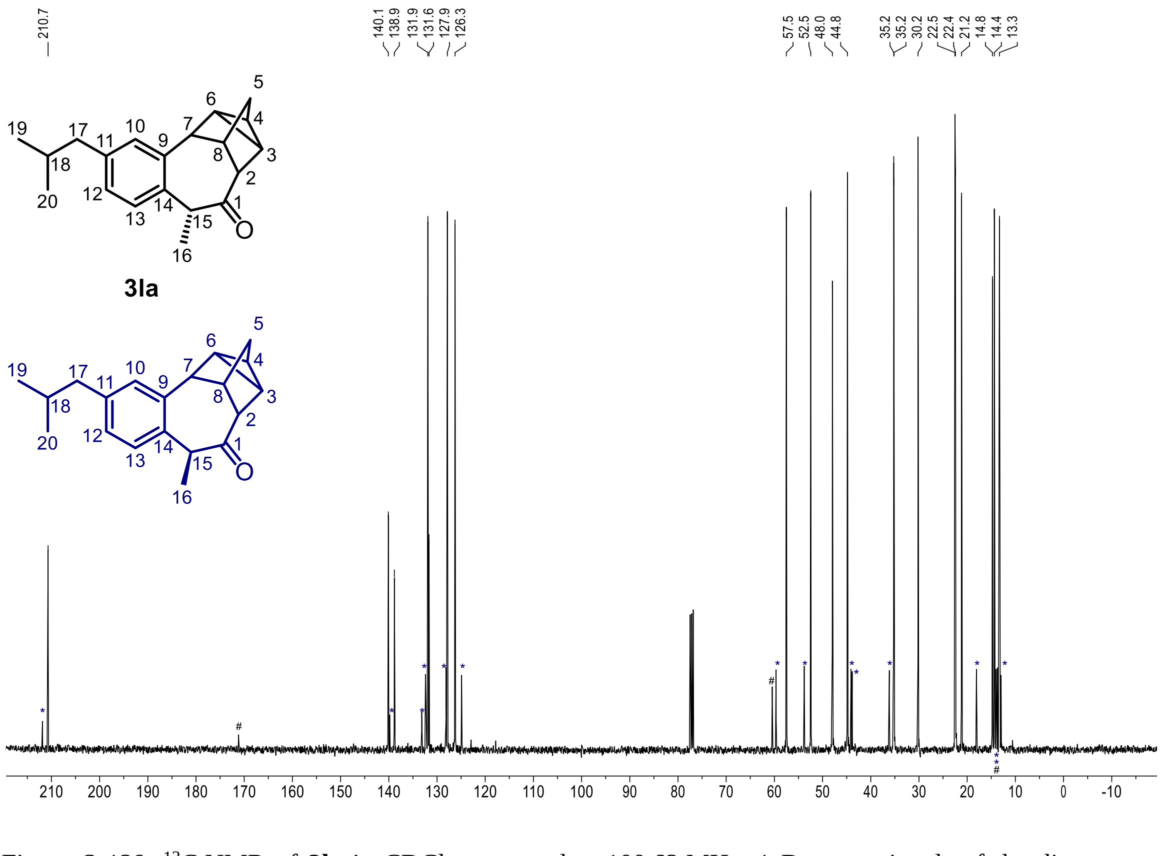

Figure S 129. ^{13}C NMR of **3la** in $CDCl_3$ measured at 100.63 MHz. * Denotes signals of the diastereomer. # Denotes residual EA.

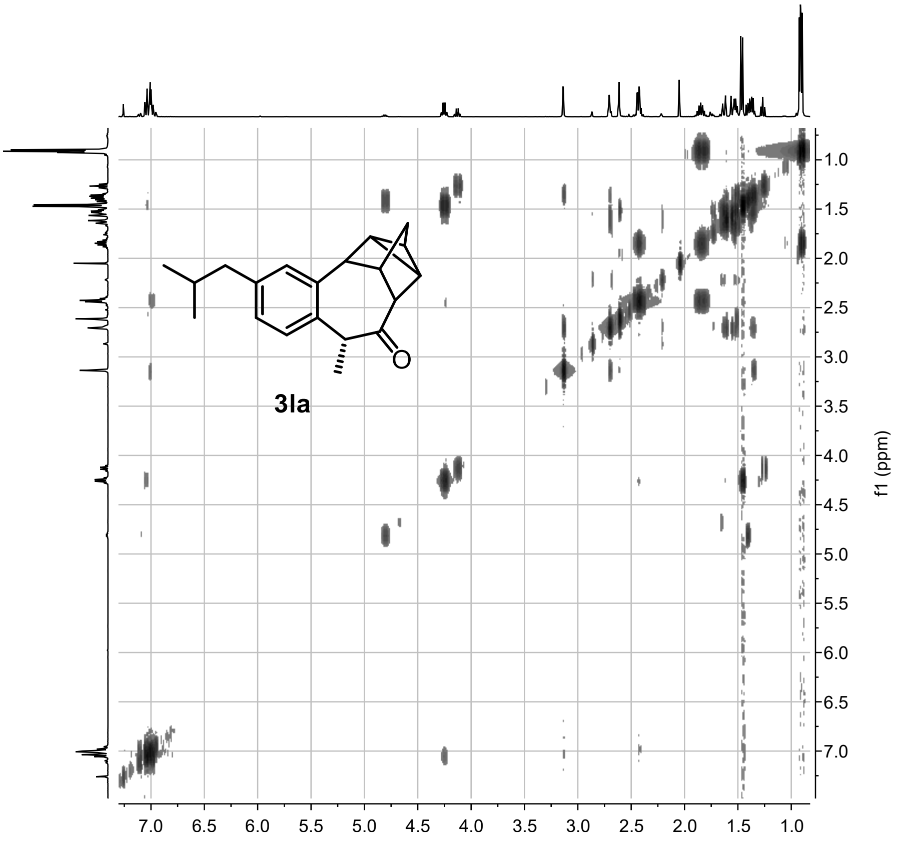

Figure S 130. $^{1}H,^{1}H$-COSY of **3la** and its respective diastereomer in $CDCl_3$ measured at 400.16 MHz.

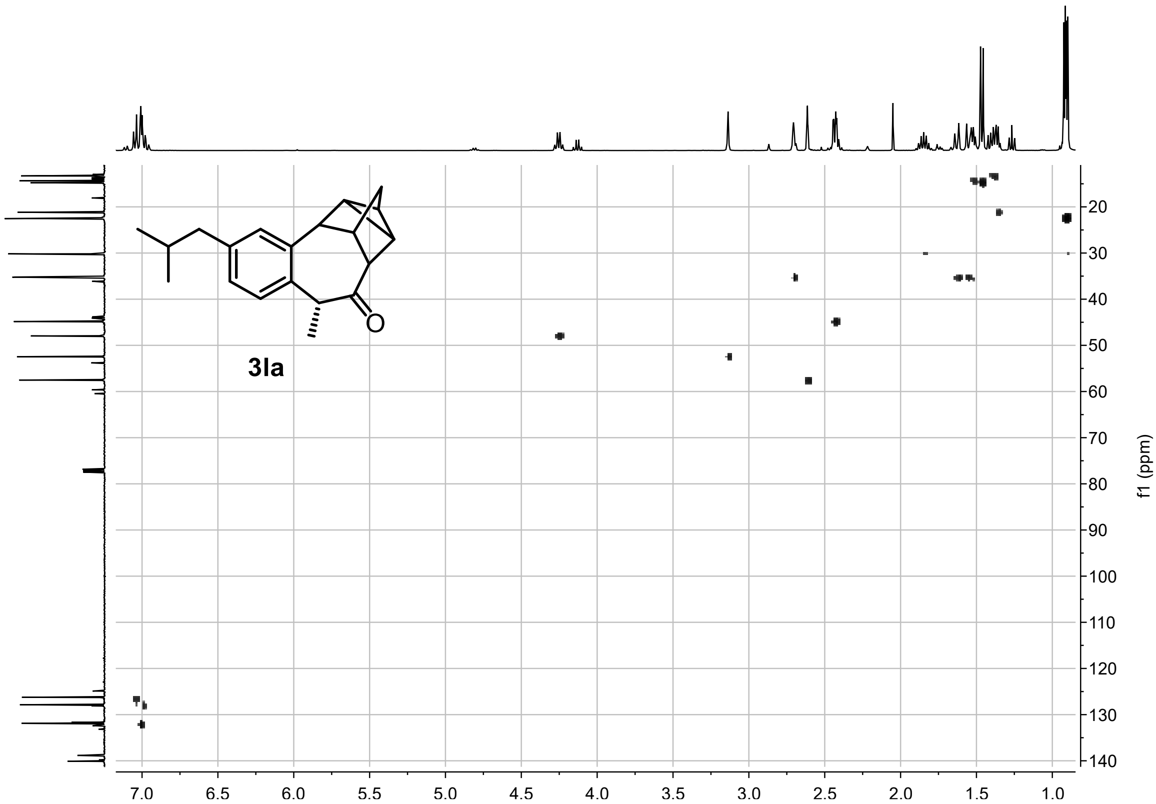

Figure S 131. $^{1}H,^{13}C$-HSQC of **3la** and its respective diastereomer in $CDCl_3$ measured at ^{1}H: 400.16 MHz; ^{13}C: 100.63 MHz.

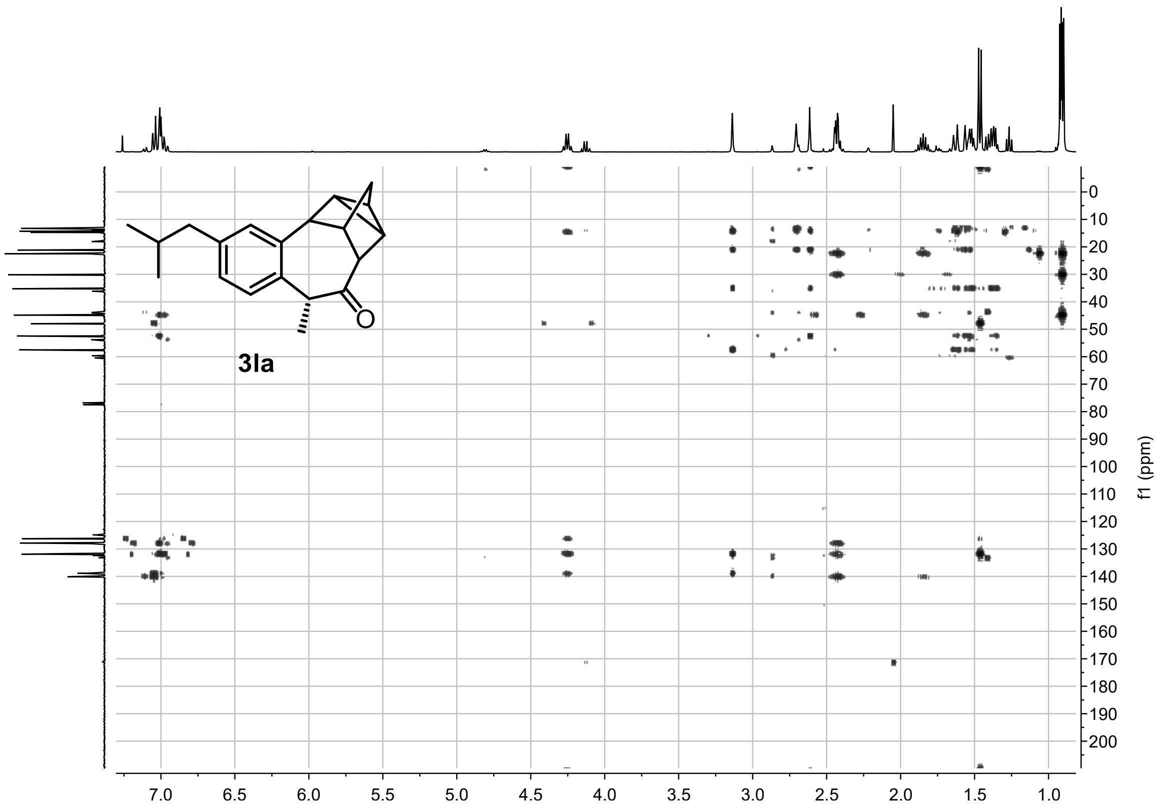

Figure S 132. $^{1}H,^{13}C$-HMBC of **3la** and its respective diastereomer in $CDCl_3$ measured at ^{1}H: 400.16 MHz; ^{13}C: 100.63 MHz.

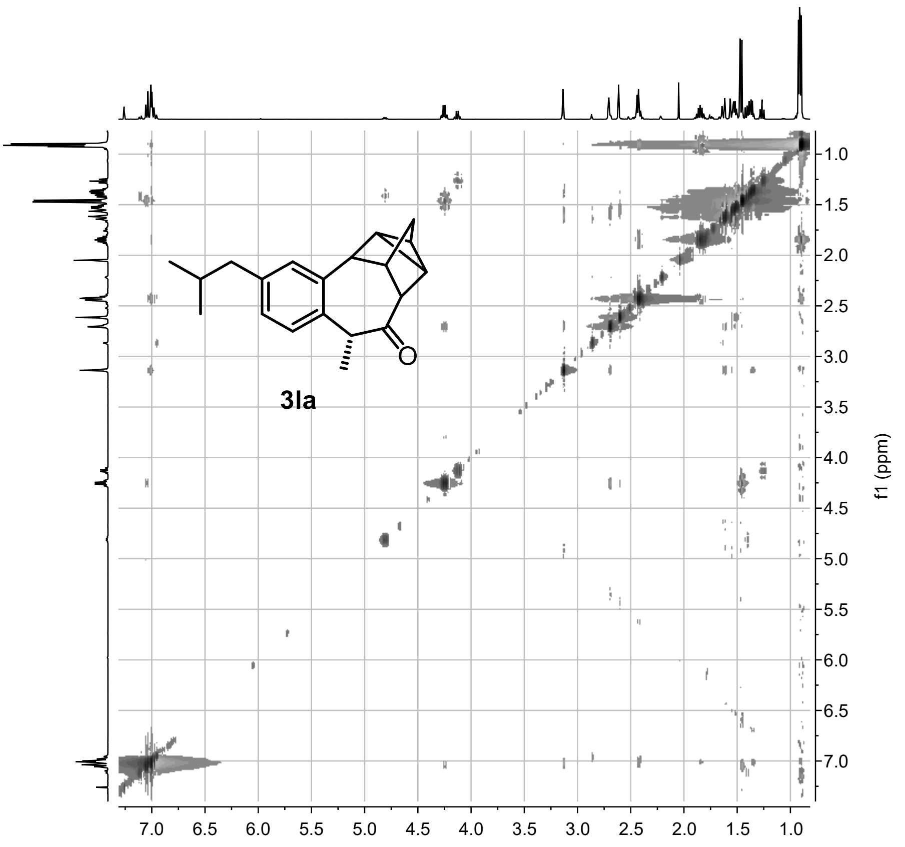

Figure S 133. $^{1}H,^{1}H$-NOESY of **3la** and its respective diastereomer in $CDCl_3$ measured at 400.16 MHz.

***rel*-(1*S*,2*R*,3*S*,3a*S*,4*R*,10a*S*,11*S*)-3-(*tert*-butoxy)-2,3,3a,4,9,10a-hexahydro-1,2,4-(epimethanetriyl)benzo[f]azulen-10(1H)-one**

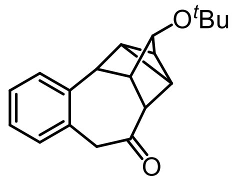

3ab

3ab was synthesized according to **GP-A** employing **1a** (118 mg, 1.00 mmol, 1.00 equiv.) and **2b** (214 mg, 1.30 mmol, 1.30 equiv.). Purification *via* flash chromatography (23 g SiO_2, gradient from 90:10 to 60:40 *n*-hexane/EA over 20 CV) afforded **3ab** (218 mg, 773 µmol, 77%) as a colorless solid.

d.r.: 87:13

$C_{19}H_{22}O_2$ (282.38 $\frac{g}{mol}$)

mp: 111.9 °C.

R$_f$: 0.53 (*n*-hexane/EA = 80:20) [anisaldehyde]

^{1}H NMR(400.16 MHz, $CDCl_3$): δ = 7.16 (m, 1H, H-12), 7.15 (m, 1H, H-11), 7.15 (m, 1H, H-10), 7.08 (m, 1H, H-13), 4.53 (dd, ^2J = 12.3 Hz, ^4J = 0.7 Hz, 1H, H-15b), 3.94 (m, 1H, H-5), 3.38 (dd, ^2J = 12.3 Hz, ^4J = 1.0 Hz, 1H, H-15a), 3.29 (s, 1H, H-2), 2.95 (s, 1H, H-7), 2.20 (s, 1H, H-8), 1.89 (m, 1H, H-3), 1.64 (m, 1H, H-6), 1.43 (m, 1H, H-4), 1.21 (s, 9H, H-17).

^{13}C NMR(100.62 MHz, $CDCl_3$): δ = 210.0 (C-1), 139.2 (C-9), 132.2 (C-14), 131.2 (C-10), 130.5 (C-13), 127.7 (C-12), 127.1 (C-11), 77.7 (C-5), 74.0 (16), 55.6 (C-2), 49.6 (C-15), 49.5 (C-7), 47.9 (C-8), 28.6 (C-17), 18.8 (C-4), 18.6 (C-3), 17.9 (C-6).

HRMS (ESI-TOF) m/z: $[M+H]^+$ Calcd for $C_{19}H_{22}O_2H$ 283.1693; Found 283.1693.

IR (ATR, ṽ): 1687 cm^{-1} (s, *CO*).

Diastereomer (anti):

$C_{19}H_{22}O_2$ (282.38 $\frac{g}{mol}$)

mp: 88.2 °C.

R$_f$: 0.55 (*n*-hexane/EA = 80:20) [anisaldehyde]

^{1}H NMR(700.21 MHz, CD_2Cl_2): δ = 7.30 (dm, ^3J = 7.0 Hz, 1H, H-10), 7.17 (m, 1H, H-11), 7.15 (m, 1H, H-12), 7.06 (d, ^3J = 6.9 Hz, 1H, H-13), 4.43 (d, ^2J = 12.2 Hz, 1H, H-15b), 3.80 (m, 1H, H-5), 3.63 (s, 1H, H-7), 3.33 (d, ^2J = 12.4 Hz, 1H, H-15a), 2.65 (s, 1H, H-2), 2.16 (s, 1H, H-8), 1.78 (m, 1H, H-3), 1.60 (m, 1H, H-6), 1.38 (m, 1H, H-4), 1.22 (s, 9H, H-17).

^{13}C NMR(176.08 MHz, CD_2Cl_2): δ = 208.2 (C-1), 140.0 (C-9), 132.3 (C-10), 132.3 (C-14), 130.4 (C-13), 127.7 (C-12), 127.3 (C-11), 78.0 (C-5), 74.2 (C-16), 56.1 (C-2), 49.3 (C-15), 48.4 (C-7), 47.7 (C-8), 28.6 (C-17), 19.7 (C-2), 18.6 (C-4), 16.4 (C-6).

HRMS (ESI-TOF) m/z: $[M+H]^+$ Calcd for $C_{19}H_{22}O_2H$ 283.1693; Found 283.1692.

IR (ATR, ṽ): 1694 cm^{-1} (s, *CO*).

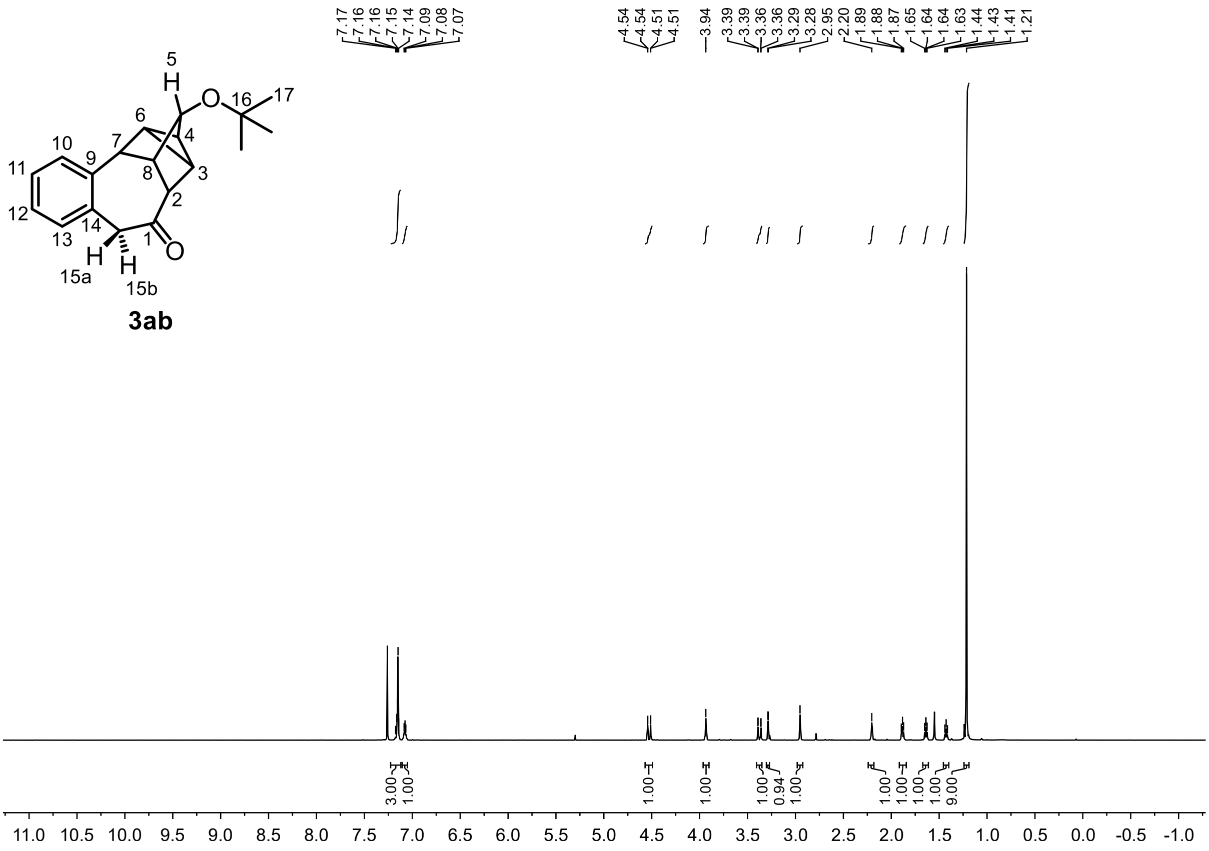

Figure S 134. ^{1}H NMR of **3ab** in $CDCl_3$ measured at 400.16 MHz.

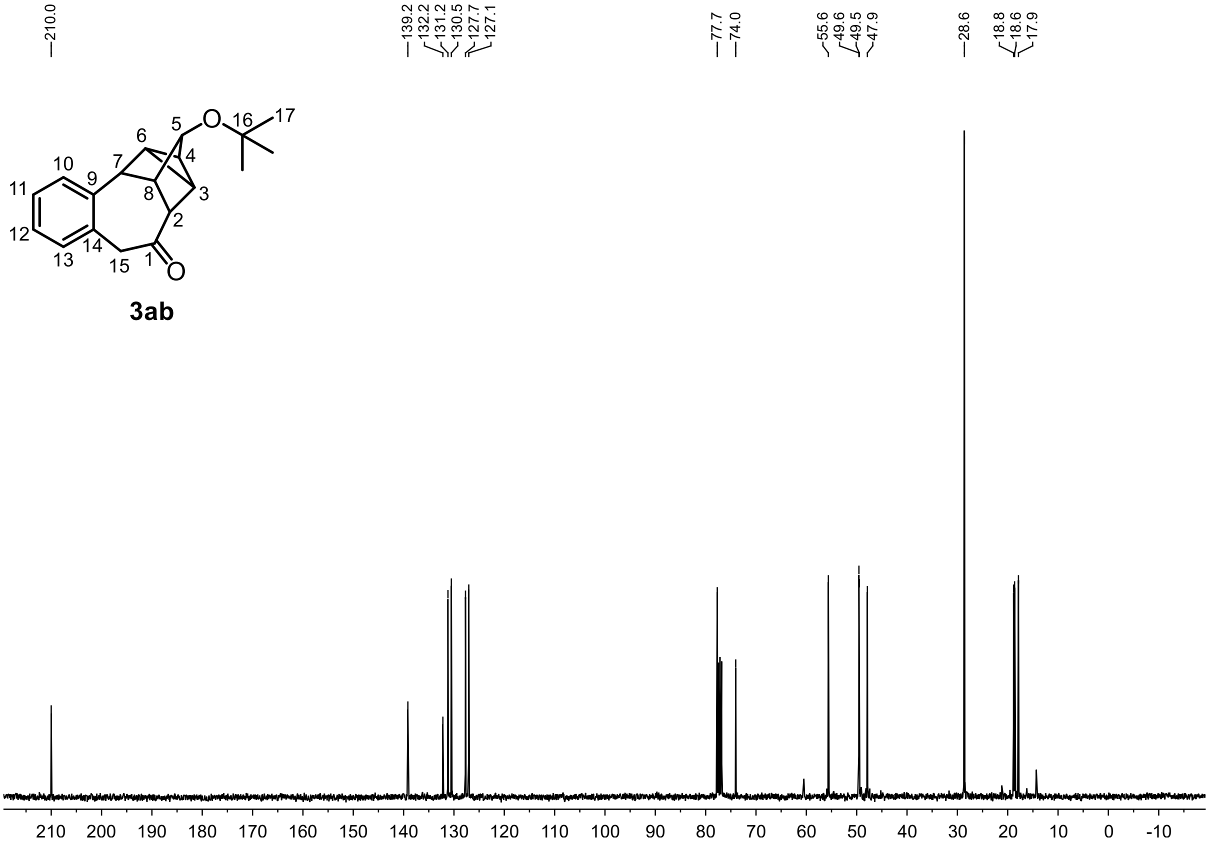

Figure S 135. ^{13}C NMR of **3ab** in $CDCl_3$ measured at 100.63 MHz.

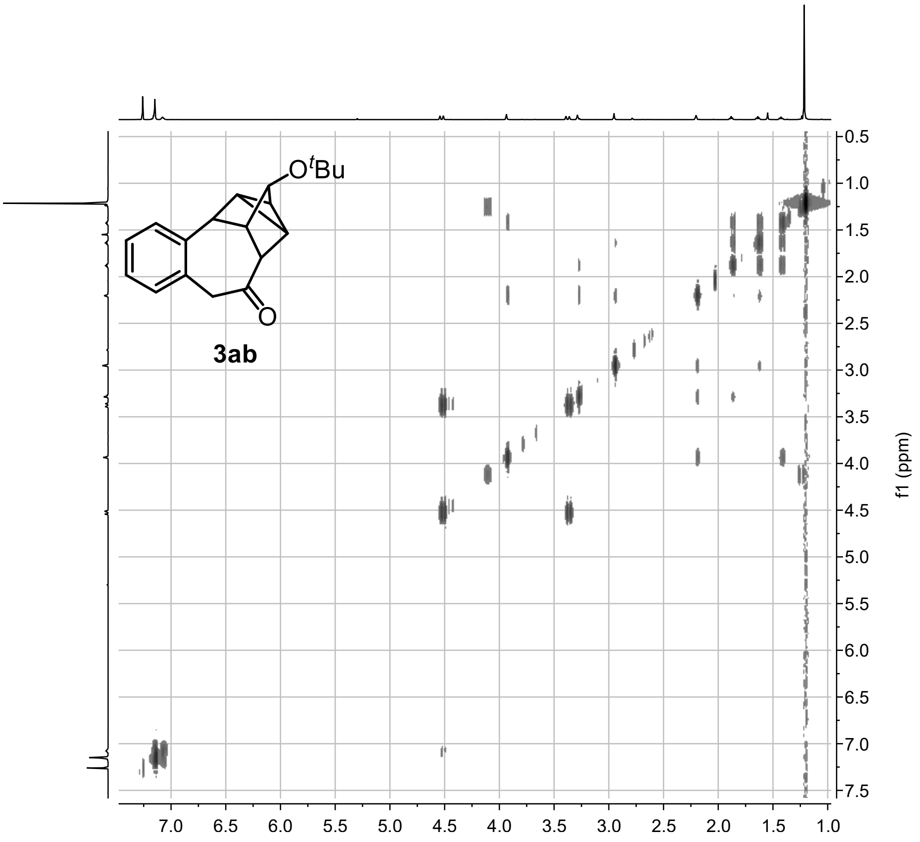

Figure S 136. ^{1}H,^{1}H-COSY of **3ab** in $CDCl_3$ measured at 400.16 MHz.

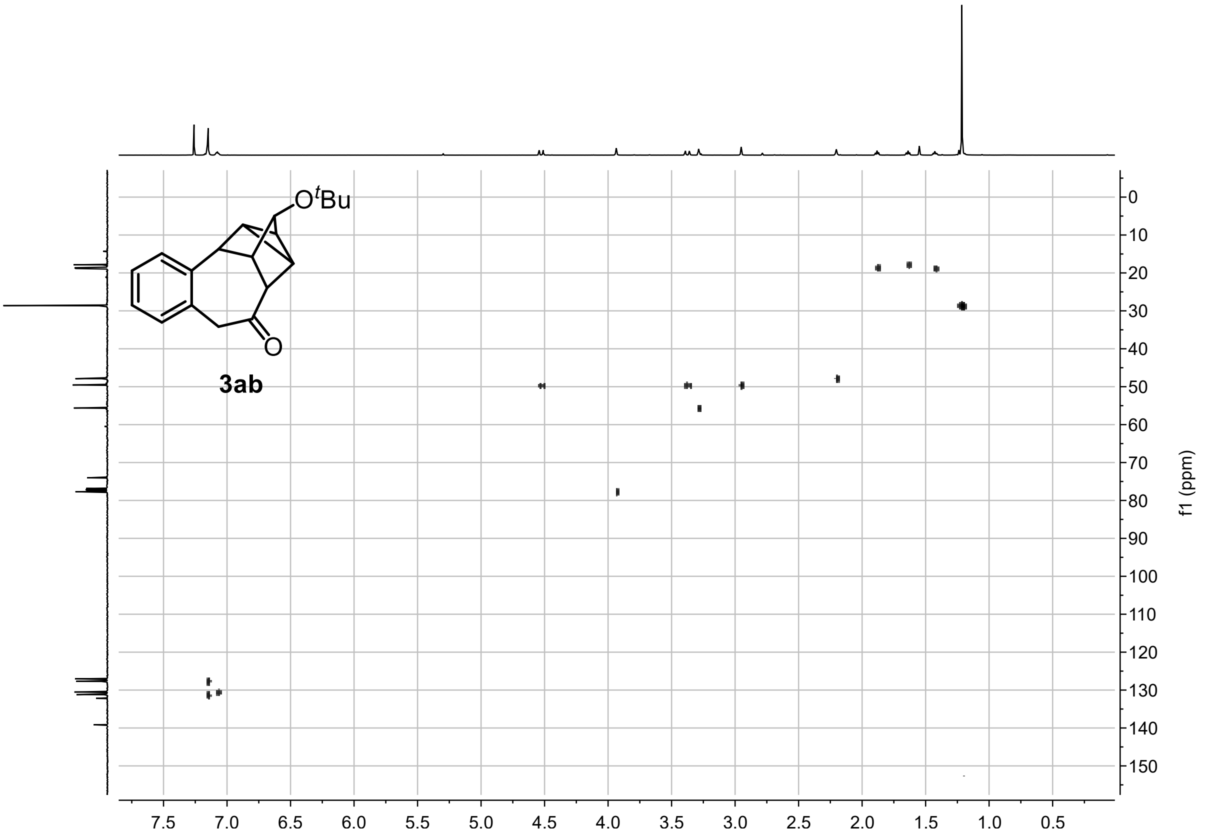

Figure S 137. ^{1}H,^{13}C-HSQC of **3ab** in $CDCl_3$ measured at ^{1}H: 400.16 MHz; ^{13}C: 100.63 MHz.

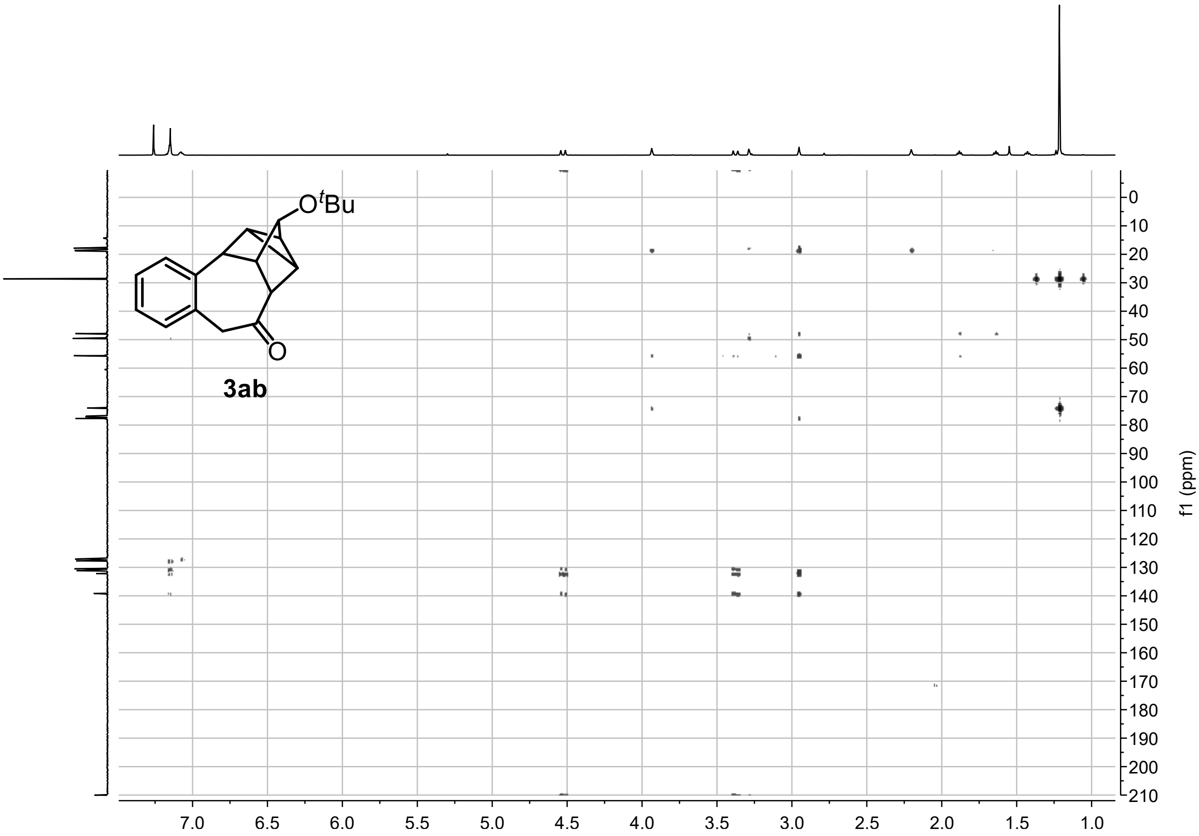

Figure S 138. $^{1}H,^{13}C$-HMBC of **3ab** in $CDCl_3$ measured at ^{1}H: 400.16 MHz; ^{13}C: 100.63 MHz.

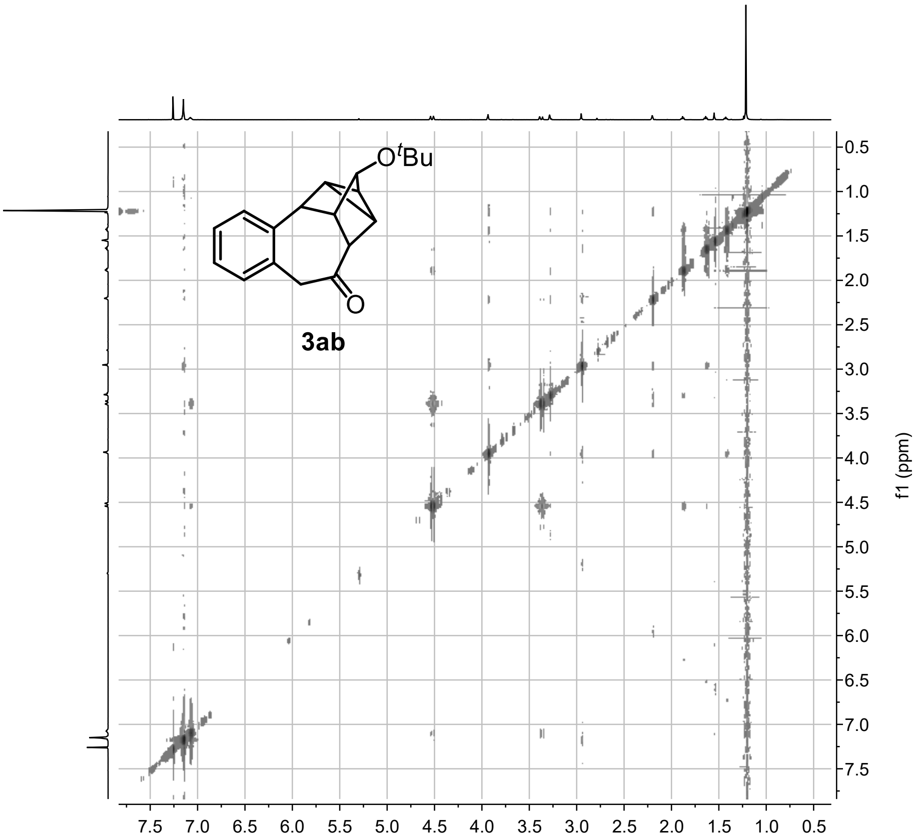

Figure S 139. $^{1}H,^{1}H$-NOESY of **3ab** in $CDCl_3$ measured at 400.16 MHz.

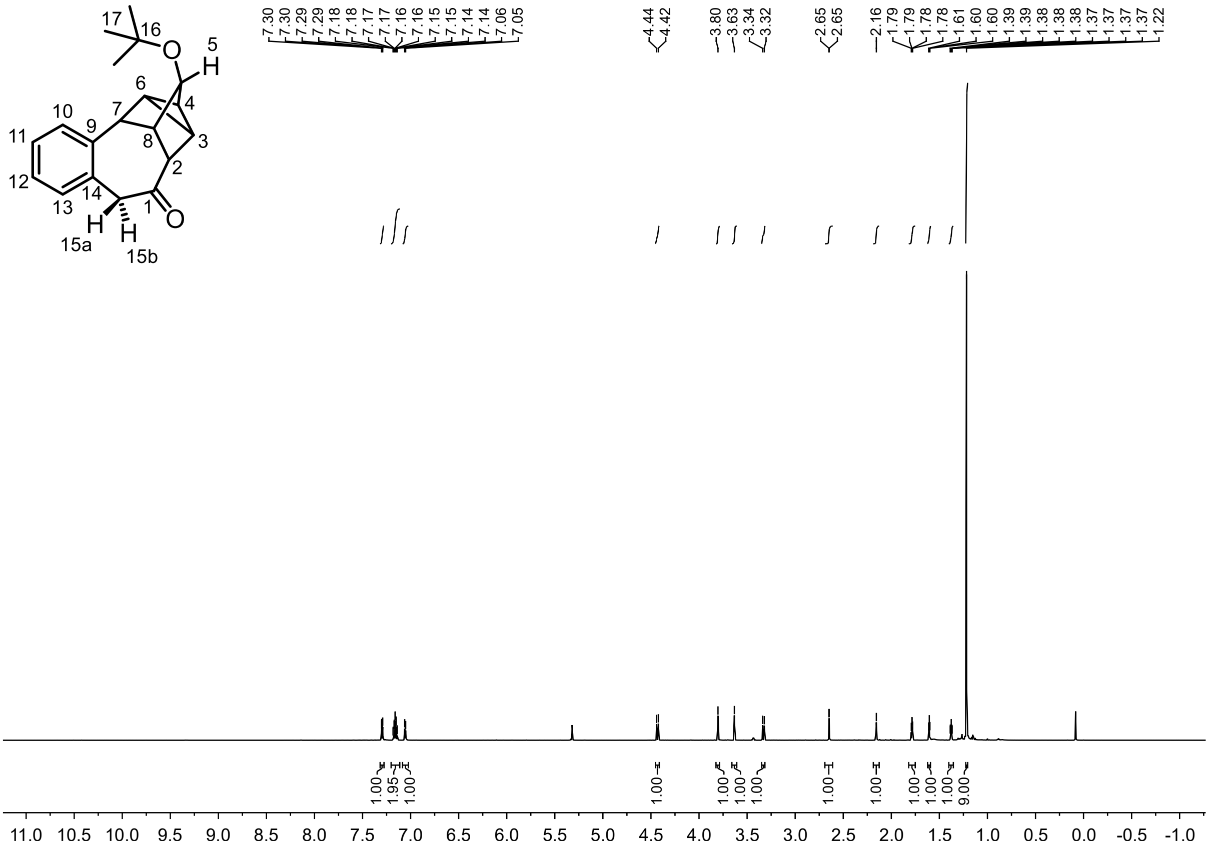

Figure S 140. ^{1}H NMR of the diastereomer of **3ab** in CD_2Cl_2 measured at 700.26 MHz.

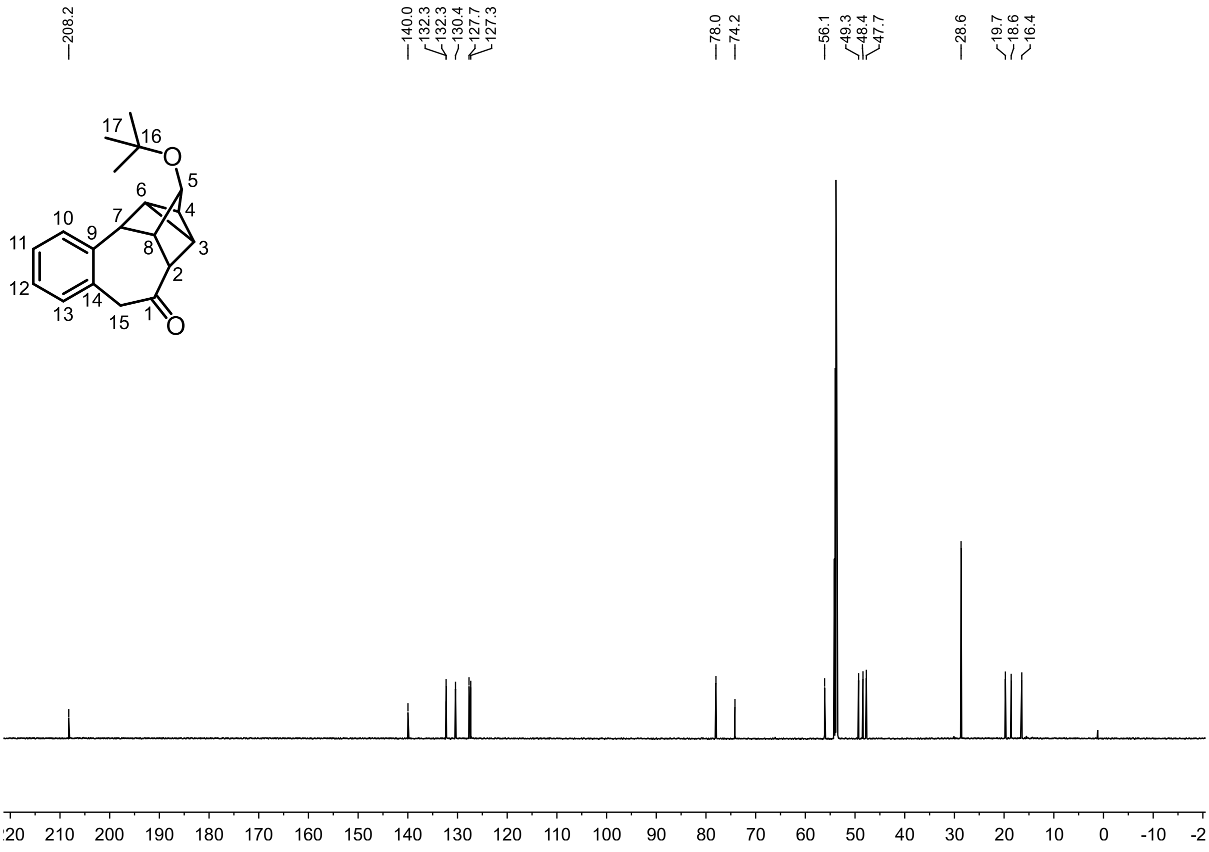

Figure S 141. ^{13}C NMR of the diastereomer of **3ab** in CD_2Cl_2 measured at 176.08 MHz.

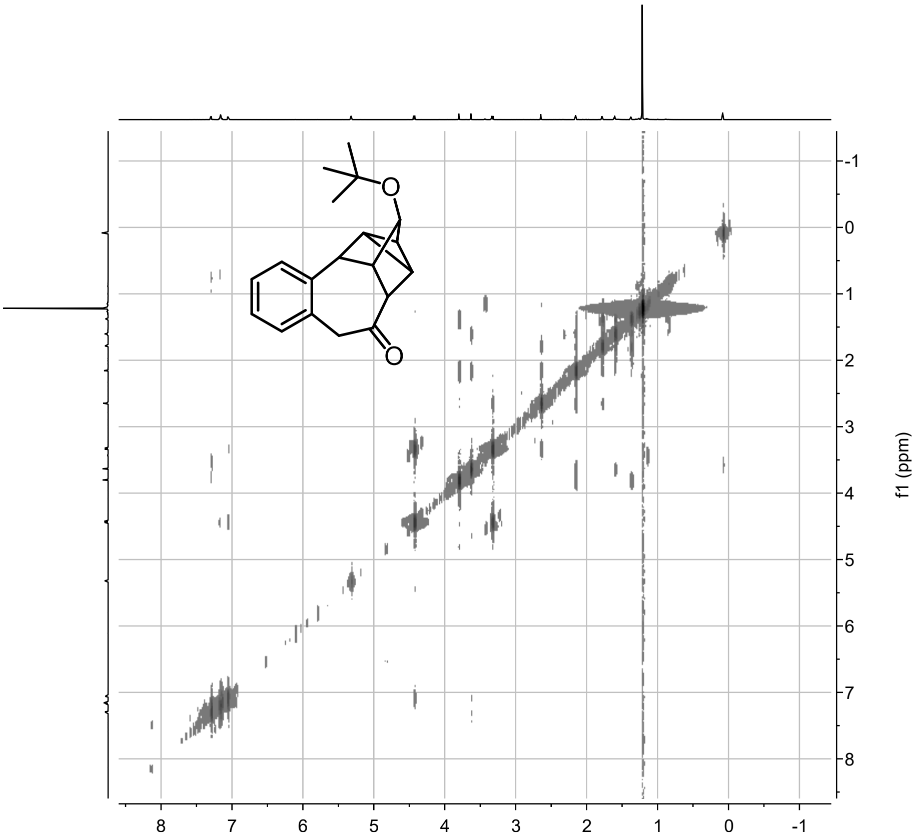

Figure S 142. $^{1}H,^{1}H$-COSY of the diastereomer of **3ab** in CD_2Cl_2 measured at 700.26 MHz.

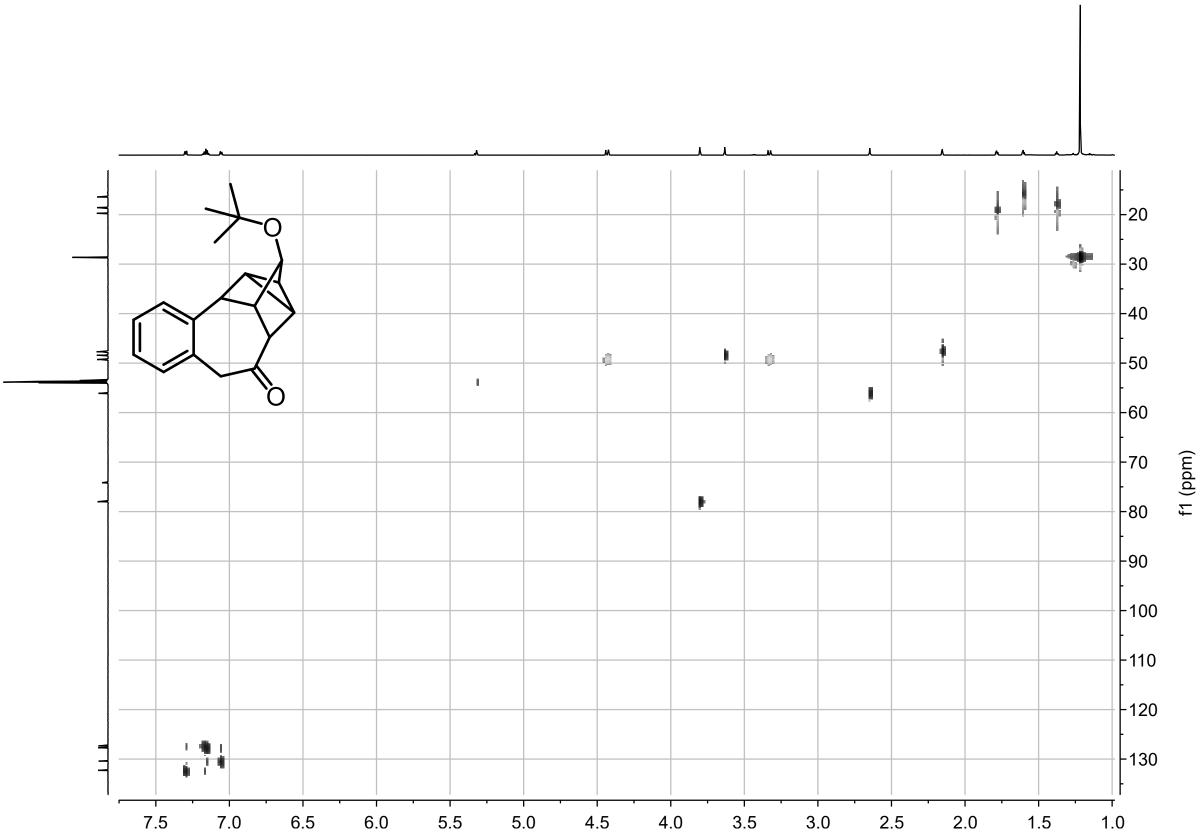

Figure S 143. $^{1}H,^{13}C$-HSQC of the diastereomer of **3ab** in CD_2Cl_2 measured at ^{1}H: 700.26 MHz; ^{13}C: 176.08 MHz.

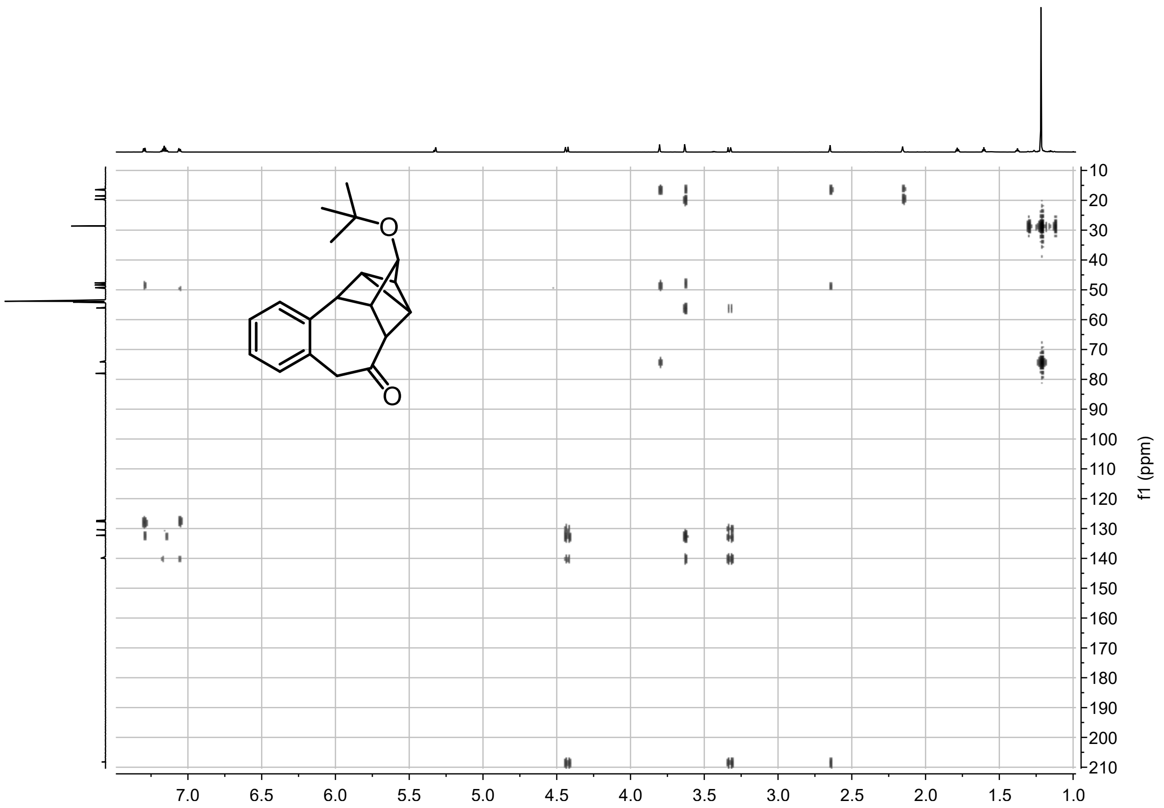

Figure S 144. $^{1}H,^{13}C$-HMBC of the diastereomer of **3ab** in CD_2Cl_2 measured at ^{1}H: 700.26 MHz; ^{13}C: 176.08 MHz.

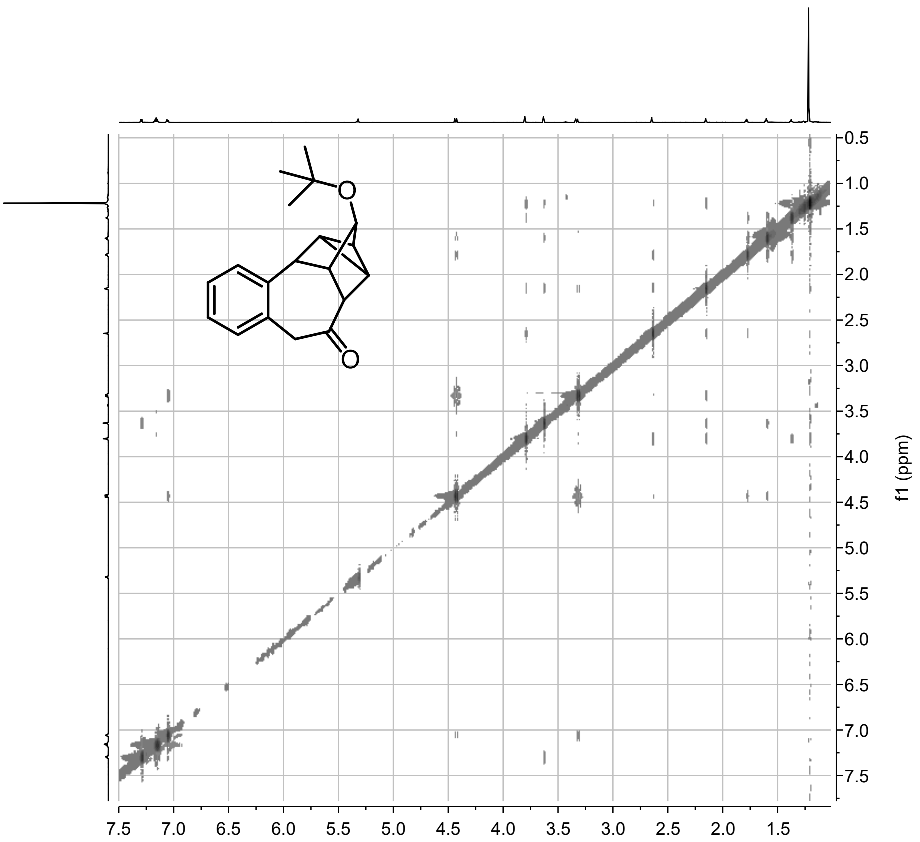

Figure S 145. $^{1}H,^{1}H$-NOESY of the diastereomer of **3ab** in CD_2Cl_2 measured at 700.26 MHz.

***rel*-(1*S*,2*R*,3*S*,3a*S*,4*R*,10a*S*,11*S*)-3-isopropyl-2,3,3a,4,9,10a-hexahydro-1,2,4-(epimethanetriyl)benzo[f]azulen-10(1H)-one**

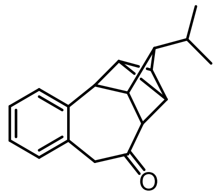

3ac

3ac was synthesized according to **GP-A** employing **1a** (118 mg, 1.00 mmol, 1.00 equiv.) and **2c** (175 mg, 1.30 mmol, 1.30 equiv.) at 50 °C instead of 80 °C. Purification *via* flash chromatography (23 g SiO_2, gradient from 98:02 to 80:20 *n*-hexane/EA over 20 CV) afforded **3ac** 205 mg, 812 µmol, 81%, mixture of isomers) as a pale yellow solid.

d.r.: 60:40

$C_{18}H_{20}O$ (252.36 $\frac{g}{mol}$)

mp: 64.1 °C.

R$_f$: 0.66 (*n*-hexane/EA = 80:20) [anisaldehyde]

^{1}H NMR(400.16 MHz, $CDCl_3$): *syn* δ = 7.15 (m, 3H, H-10 – 12), 7.07 (m, 1H, H-13), 4.58 (d, ^2J = 12.2 Hz, 1H, H-15b), 3.32 (dd, ^2J = 12.3 Hz, ^4J = 0.9 Hz, 1H, H-15a), 2.85 (s, 1H, H-7), 2.80 (m, 1H, H-2), 2.21 (m, 1H, H-8), 1.70 (m, 1H, H-6), 1.56 (m, 1H, H-4), 1.49 (m, 1H, H-3), 1.48 (m, 1H, H-5), 1.33 (m, 1H, H-16), 0.98 (d, ^3J = 6.5 Hz, 3H, H-18), 0.85 (d, ^3J = 6.5 Hz, 3H, H-17); *anti* δ = 7.20 (m, 1H, H-10), 7.15 (m, 2H, H-11/12), 7.07 (m, 1H, H-13), 4.44 (d, ^2J = 12.2 Hz, 1H, H-15b), 3.40 (d, ^2J = 12.2 Hz, 1H, H-15a), 3.15 (s, 1H, H-7), 2.61 (m, 1H, H-2), 2.26 (m, 1H, H-8), 1.74 (m, 1H, H-6), 1.63 (m, 1H, H-16), 1.47 (m, 1H, H-4), 1.46 (m, 1H, H-3), 1.32 (m, 1H, H-5), 1.01 (d, ^3J = 6.6 Hz, 3H, H-18), 0.89 (d, ^3J = 6.6 Hz, 3H, H-17).

^{13}C NMR(100.62 MHz, $CDCl_3$): *syn* δ = 210.1 (C-1), 140.0 (C-9), 132.1 (C-14), 131.4 (C-10), 130.2 (C-13), 127.6 (C-12), 127.0 (C-11), 56.8 (C-5), 55.6 (C-2), 54.1 (C-7), 49.1 (C-15), 44.7 (C-8), 26.9 (C-16), 21.5 (C-18), 21.0 (C-17), 17.9 (C-6), 17.1 (C-3), 16.7 (C-4); *anti* δ = 209.3 (C-1), 139.7 (C-9), 131.9 (C-10), 131.7 (C-14), 130.4 (C-13), 127.6 (C-12), 127.1 (C-11), 60.2 (C-2), 56.7 (C-5), 49.2 (C-15), 48.4 (C-7), 44.3 (C-8), 27.1 (C-16), 21.8 (C-18), 21.1 (C-17), 19.6 (C-6), 16.4 (C-3), 15.5 (C-4).

HRMS (ESI-TOF) m/z: $[M+H]^+$ Calcd for $C_{18}H_{20}OH$ 253.1587; Found 253.1588.

IR (ATR, ṽ): 1690 cm^{-1} (s, *CO*).

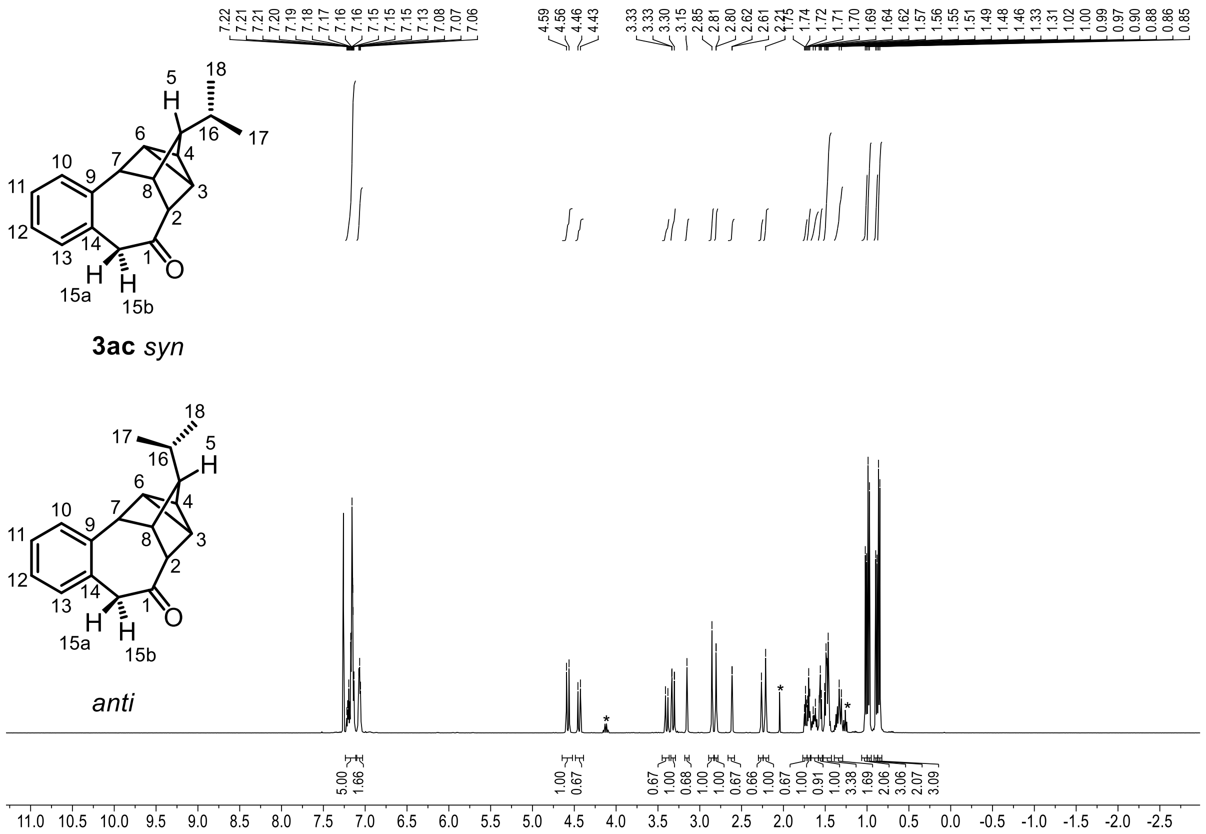

Figure S 146. ^{1}H NMR of **3ac** in $CDCl_3$ measured at 400.16 MHz. * denotes residual EA.

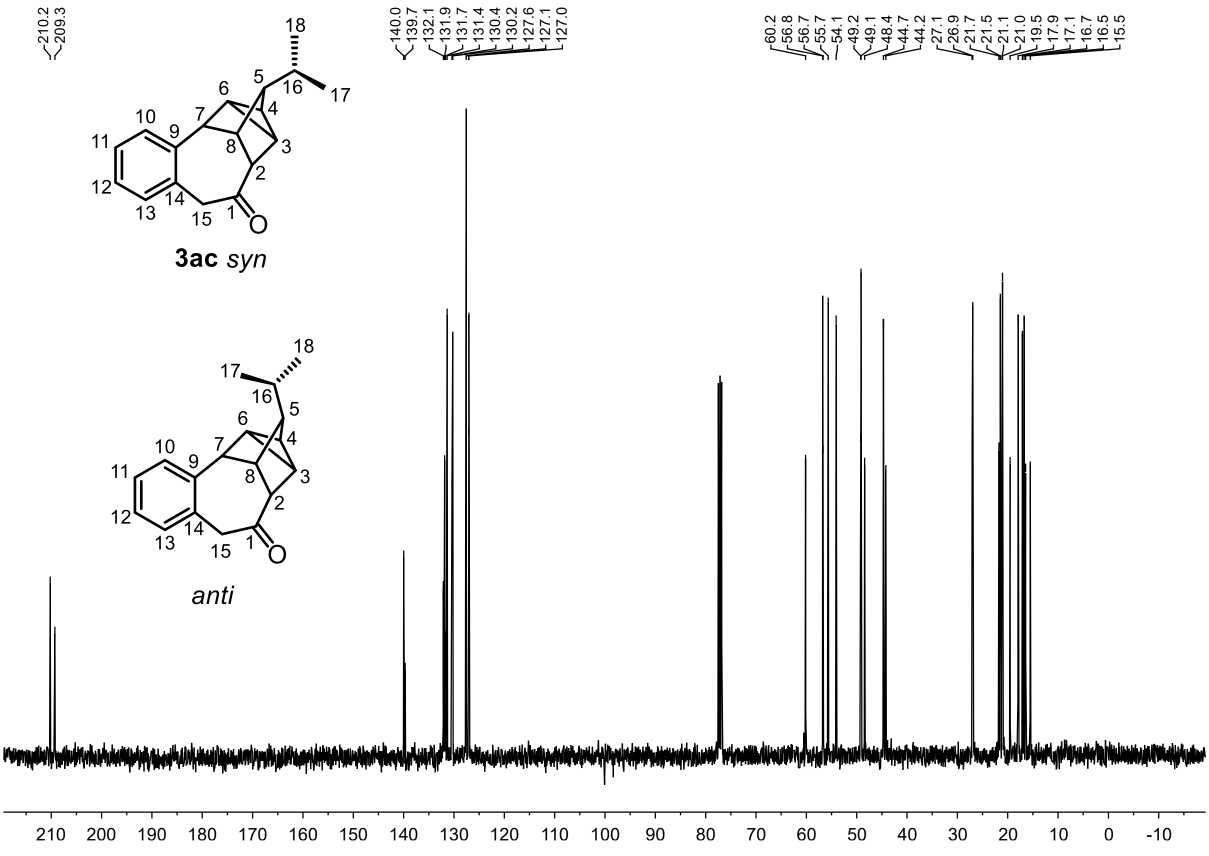

Figure S 147. ^{13}C NMR of **3ac** in $CDCl_3$ measured at 100.63 MHz.

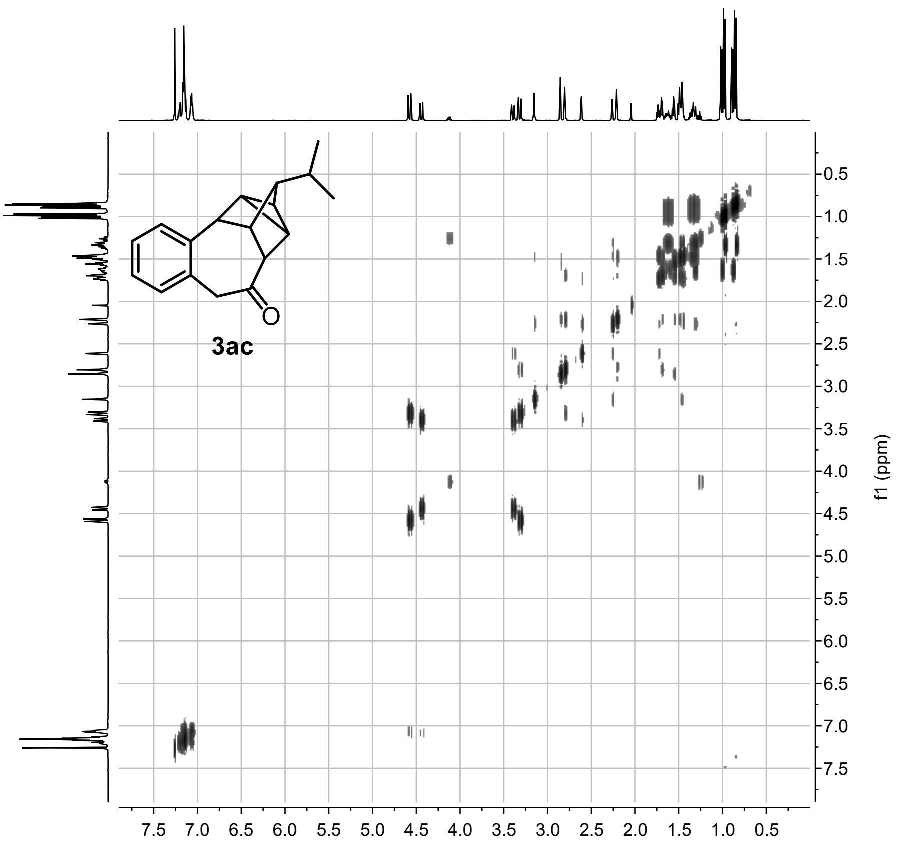

Figure S 148. $^{1}H,^{1}H$-COSY of **3ac** in $CDCl_3$ measured at 400.16 MHz.

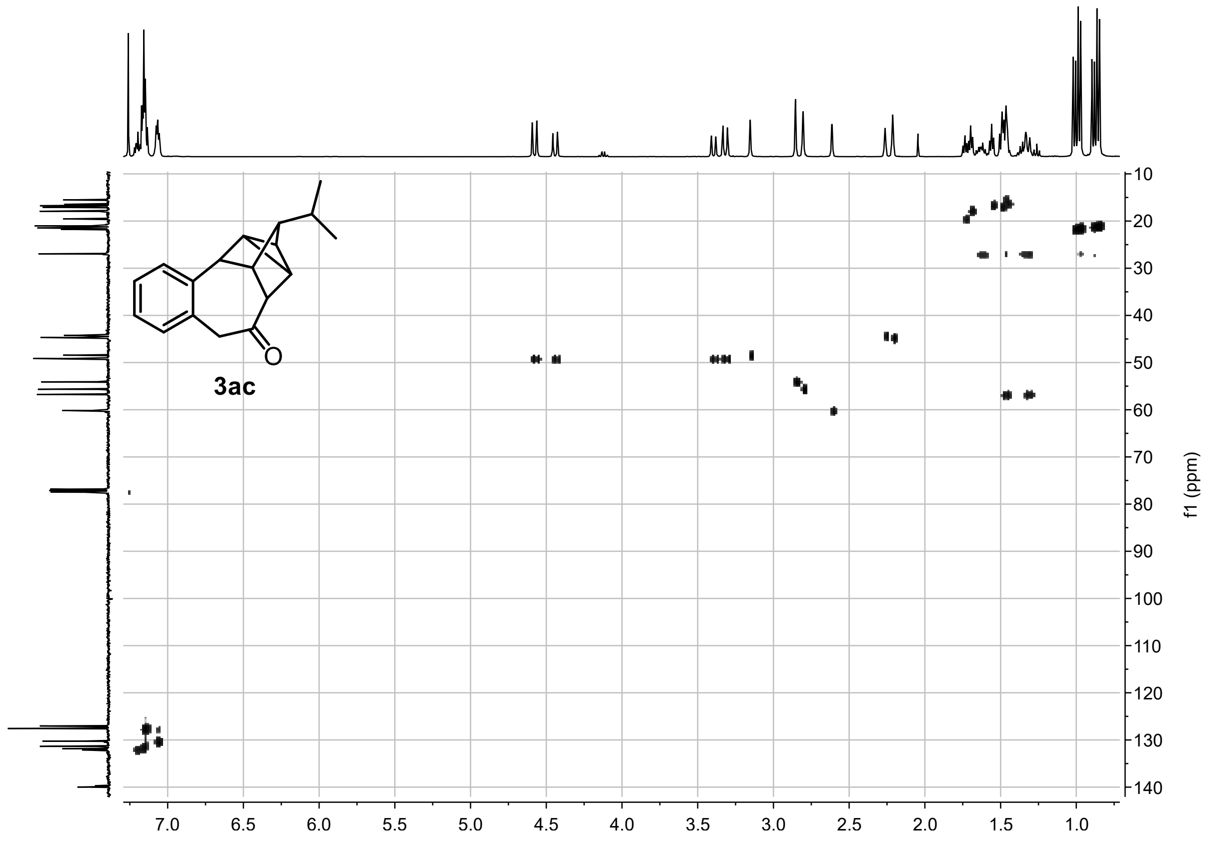

Figure S 149. $^{1}H,^{13}C$-HSQC of **3ac** in $CDCl_3$ measured at ^{1}H: 400.16 MHz; ^{13}C: 100.63 MHz.

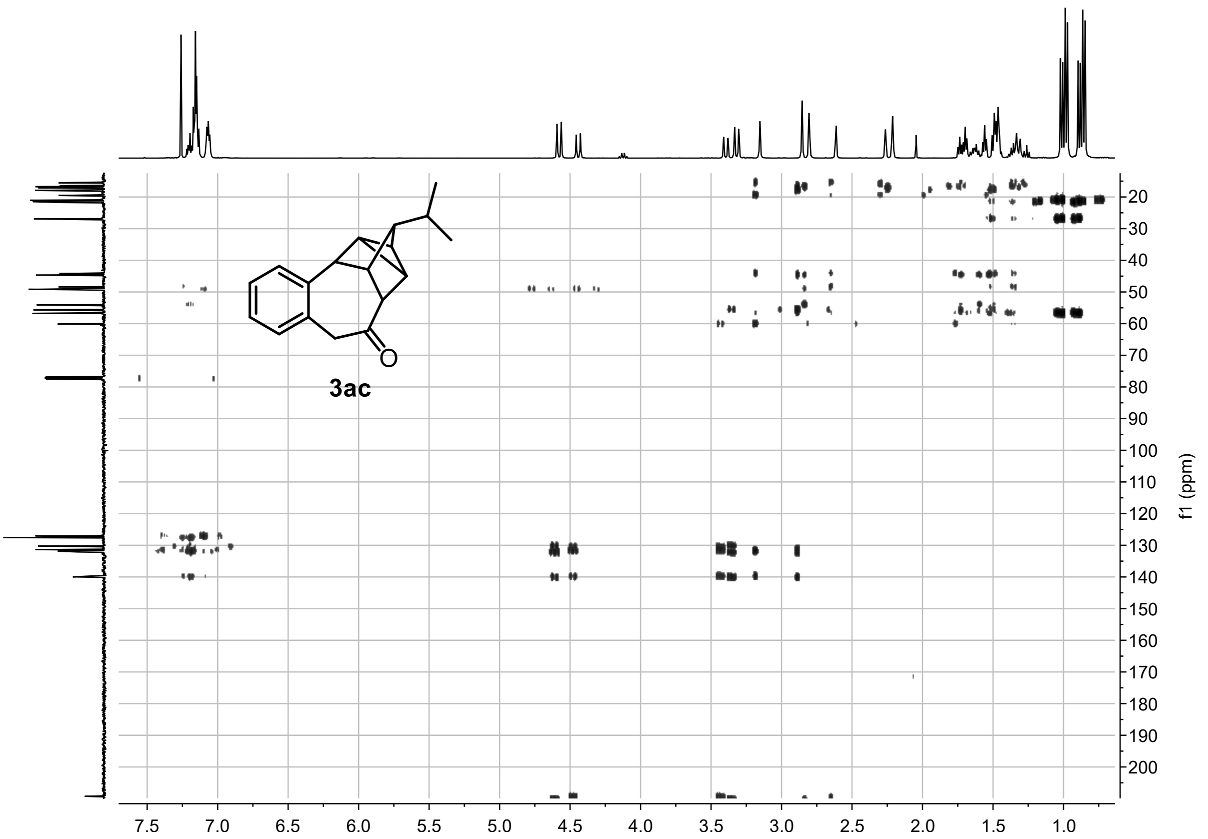

Figure S 150. $^{1}H,^{13}C$-HMBC of **3ac** in $CDCl_3$ measured at ^{1}H: 400.16 MHz; ^{13}C: 100.63 MHz.

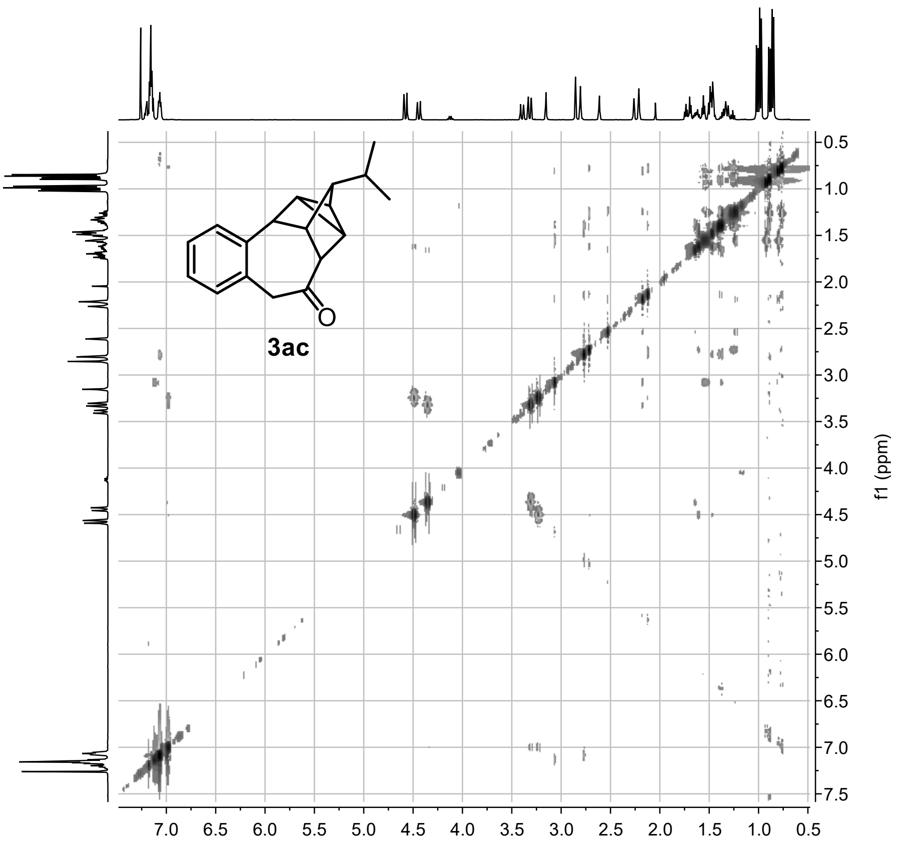

Figure S 151. $^{1}H,^{1}H$-NOESY of **3ac** in $CDCl_3$ measured at 400.16 MHz.

***rel*-(1*R*,2*R*,3*R*,3a*S*,10a*S*,11*S*)-3-phenyl-2,3,3a,4,9,10a-hexahydro-1,2,4-(epimethanetriyl) benzo[f]azulen-10(1H)-one**

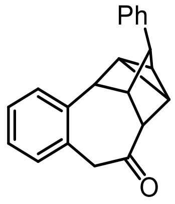

3ad

3ad was synthesized according to **GP-A** employing **1a** (118 mg, 1.00 mmol, 1.00 equiv.) and **2d** (219 mg, 1.30 mmol, 1.30 equiv.). Purification *via* flash chromatography (23 g SiO_2, gradient from 98:02 to 80:20 *n*-hexane/EA over 15 CV) afforded **3ad** (249 mg, 870 µmol, 87%) as a colorless solid.

$C_{21}H_{18}O$ (286.37 $\frac{g}{mol}$)

mp: 156.5 °C.

R$_f$: 0.64 (*n*-hexane/EA = 80:20) [anisaldehyde]

^{1}H NMR(400.16 MHz, $CDCl_3$): δ = 7.38 (m, 1H, H-19), 7.36 (m, 1H, H-17), 7.34 (m, 1H, H-10), 7.30 (m, 1H, H-18), 7.26 (m, 1H, H-11), 7.13 (m, 1H, H-12), 7.07 (m, 2H, H-13, H-20), 6.94 (m, 1H, H-21), 4.50 (d, ^2J = 12.2 Hz, 1H, H-15b), 3.44 (d, ^2J = 12.2 Hz, 1H, H-15a), 3.14 (s, 1H, H-6), 2.95 (s, 1H, H-5), 2.94 (s, 1H, H-2), 2.43 (m, 1H, H-8), 1.93 (ddm, ^3J = 5.8 Hz, ^3J = 4.8 Hz, 1H, H-3), 1.81 (ddm, ^3J = 5.3 Hz, ^3J = 5.3 Hz, 1H, H-3), 1.71 (ddm, ^3J = 4.8 Hz, ^3J = 4.9 Hz, 1H, H-3).

^{13}C NMR(100.62 MHz, $CDCl_3$): δ = 208.6 (C-1), 140.2 (C-16), 138.9 (C-9), 131.9 (C-21), 131.8 (C-14), 130.3 (C-13), 128.4 (3C, C-10, C-18, C-19), 127.6 (C-12), 127.0 (3C, C-11, C-20), 126.7 (C-17), 60.3 (C-2), 52.0 (C-7), 49.2 (C-15), 47.9 (C-5), 47.2 (C-8), 19.4 (C-6), 19.2 (C-3), 16.3 (C-4).

HRMS (ESI-TOF) m/z: $[M+H]^+$ Calcd for $C_{21}H_{18}OH$ 287.1430; Found 287.1433.

IR (ATR, ṽ): 1694 cm^{-1} (s, *CO*).

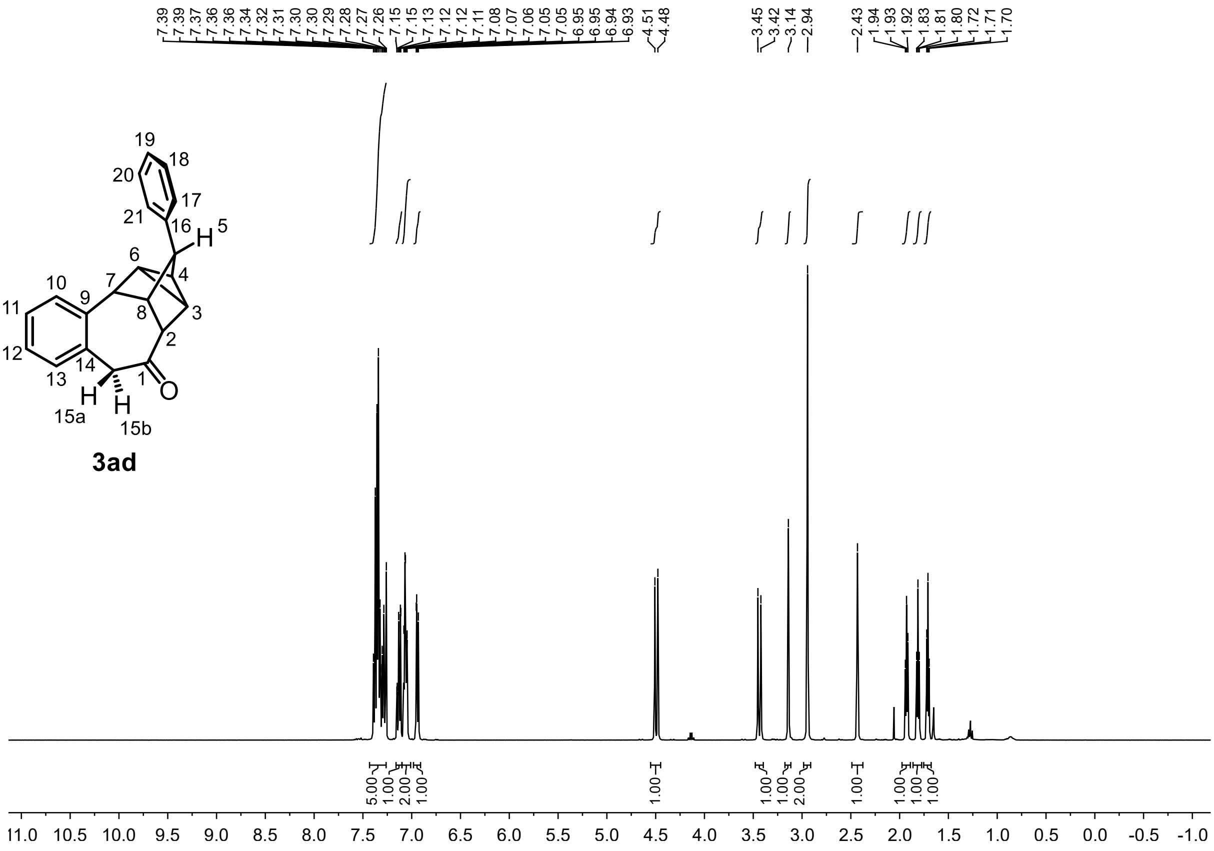

Figure S 152. ^{1}H NMR of **3ad** in $CDCl_3$ measured at 400.16 MHz.

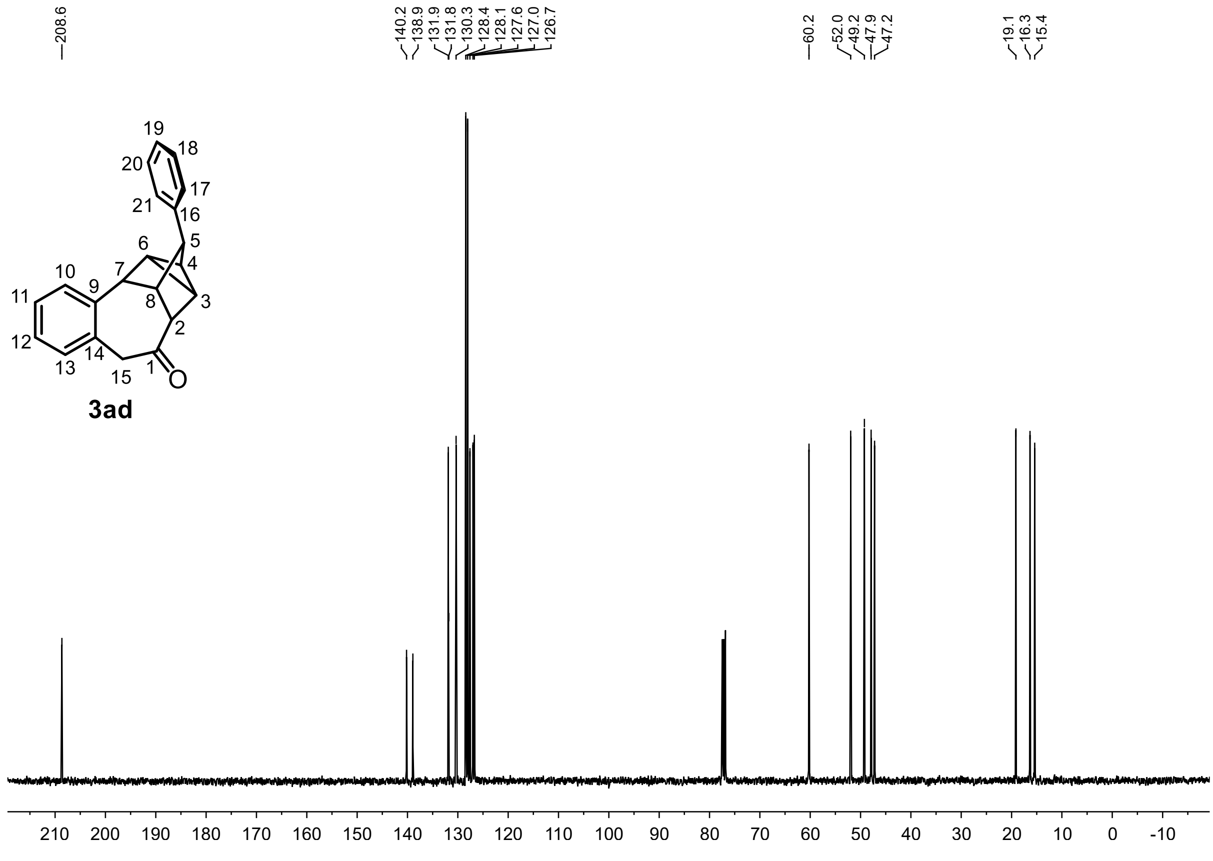

Figure S 153. ^{13}C NMR of **3ad** in $CDCl_3$ measured at 100.63 MHz.

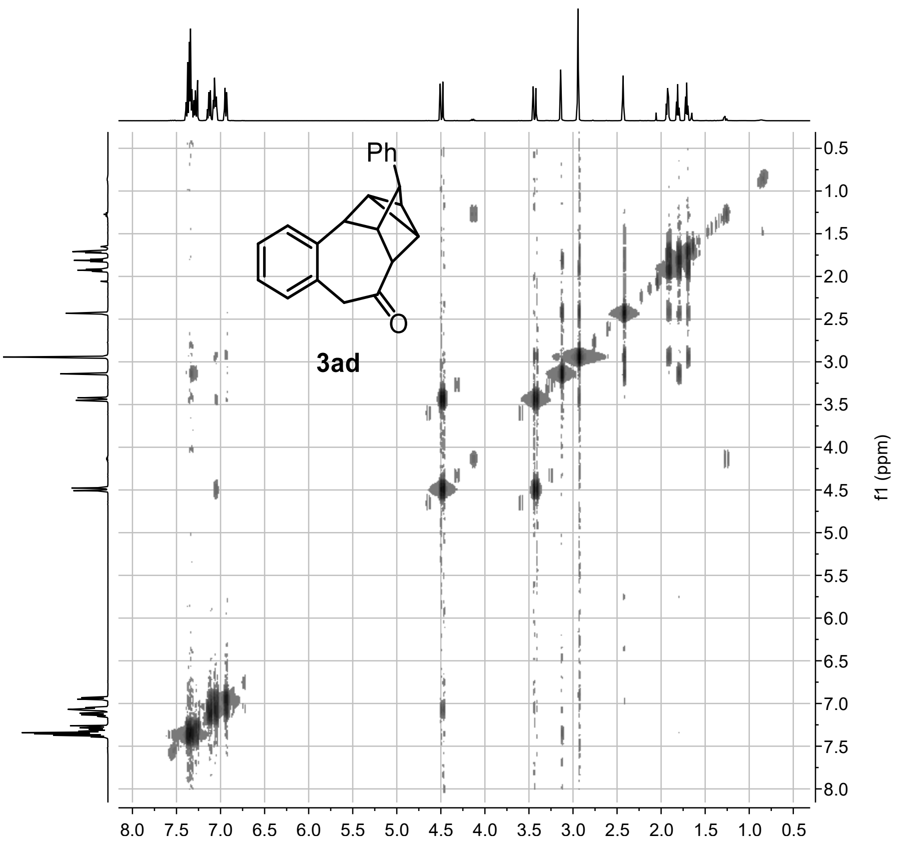

Figure S 154. ^{1}H,^{1}H-COSY of **3ad** in $CDCl_3$ measured at 400.16 MHz.

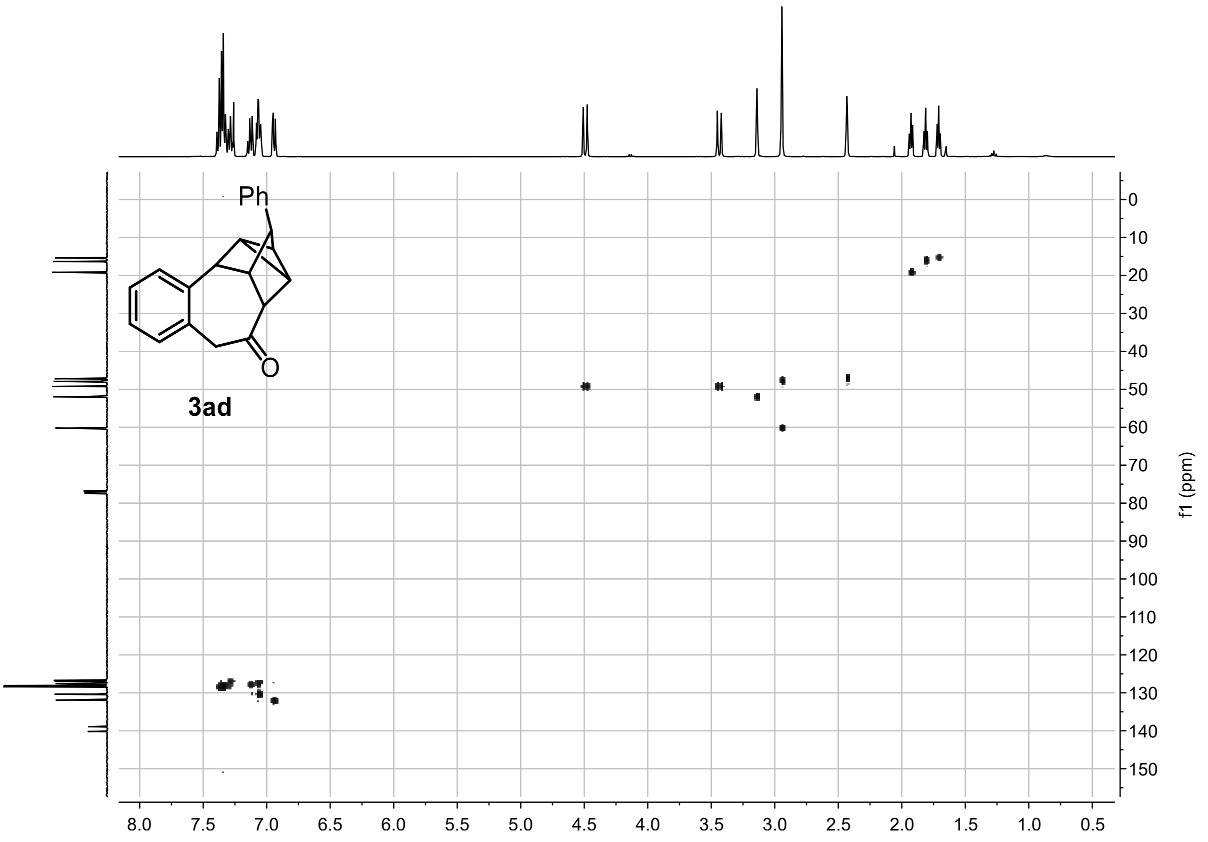

Figure S 155. ^{1}H,^{13}C-HSQC of **3ad** in $CDCl_3$ measured at ^{1}H: 400.16 MHz; ^{13}C: 100.63 MHz.

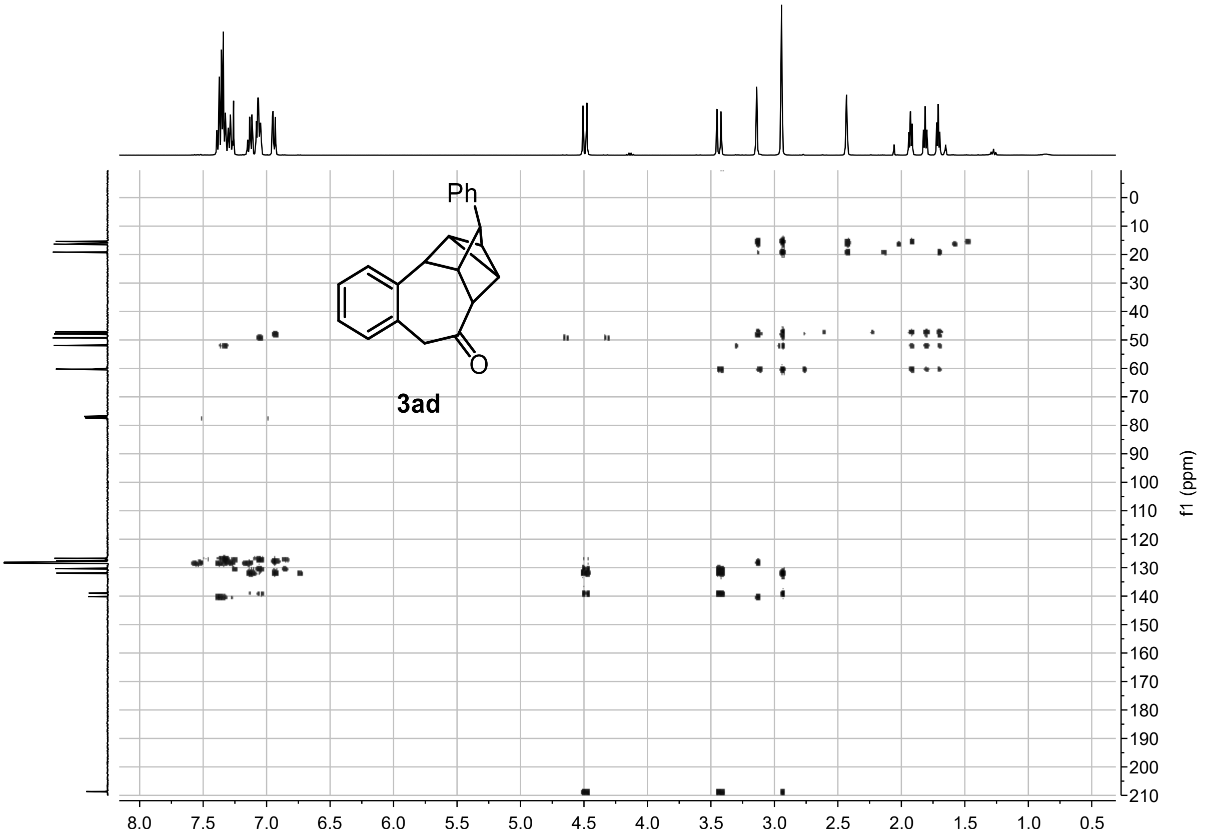

Figure S 156. $^{1}H,^{13}C$-HMBC of **3ad** in $CDCl_3$ measured at ^{1}H: 400.16 MHz; ^{13}C: 100.63 MHz.

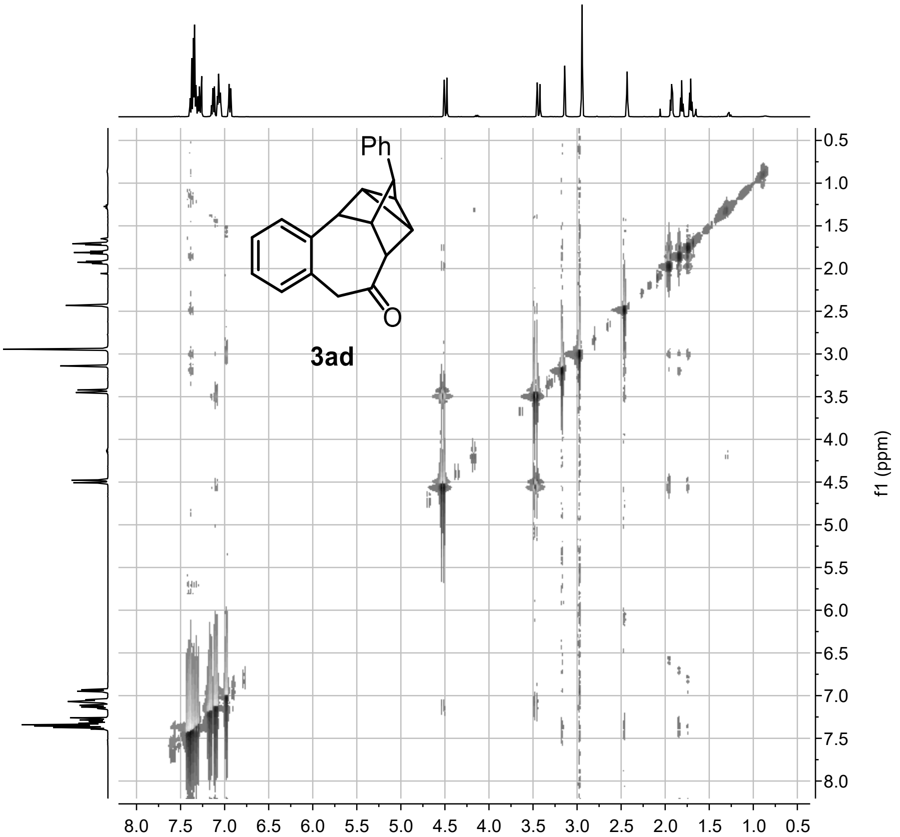

Figure S 157. $^{1}H,^{1}H$-NOESY of **3ad** in $CDCl_3$ measured at 400.16 MHz.

***rel*-(1*R*,2*R*,3a*S*,4*S*,10a*R*,11*R*)-1-hexyl-2,3,3a,4,9,10a-hexahydro-1,2,4-(epimethanetriyl) benzo[f]azulen-10(1H)-one**

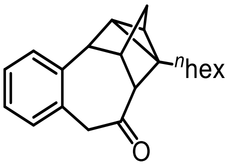

3ae

3ae was synthesized according to **GP-A** employing **1a** (118 mg, 1.00 mmol, 1.00 equiv.) and **2e** (229 mg, 1.30 mmol, 1.30 equiv.). PhCN (206 µL, 2.00 mmol, 2.00 equiv.) was added prior to **1a** and **2e**. Purification *via* flash chromatography (23 g SiO_2, gradient from 100:00 to 85:15 *n*-hexane/EA over 15 CV) afforded **3ae** and the respective isomer (147 mg, 499 µmol, 50%) as a pale yellow oil.

constitumeric ratio: 58:42

$C_{21}H_{26}O$ (294.44 $\frac{g}{mol}$)

R_f: 0.63 (*n*-hexane/EA = 80:20) [anisaldehyde]

^{1}H NMR(400.16 MHz, $CDCl_3$): δ = 7.15 (m, 3H, H-10, H-11, H-12), 7.07 (m, 1H, H-13), 4.46 (d, ^2J = 12.3 Hz, 1H, H-15b), 3.27 (d, ^2J = 12.3 Hz, 1H, H-15a), 2.81 (s, 1H, H-7), 2.66 (m, 1H, H-2), 2.26 (s, 1H, H-8), 1.71 (dm, ^2J = 10.5 Hz, 1H, H-5a), 1.71 (m, 1H, H-16a), 1.51 (s, 1H, H-6), 1.51 (dm, ^2J = 10.5 Hz, H-5b), 1.35 (dm, ^3J = 5.2 Hz, H-4), 1.29 (m, 1H, H-16b), 1.26 (m, 2H, H-17), 1.23 (m, 2H, H-18), 1.20 (m, 4H, H-19, H-20), 0.85 (t, ^3J = 6.8 Hz, 3H, H-21).

^{13}C NMR(100.62 MHz, $CDCl_3$): δ = 209.9 (C-1), 138.8 (C-9), 132.2 (C-10), 131.9 (C-14), 130.3 (C-13), 127.6 (C-12), 127.0 (C-11), 60.3 (C-2), 55.4 (C-7), 49.3 (C-15), 44.5 (C-8), 36.5 (C-5), 31.9 (C-19), 29.6 (C-20), 27.9 (C-17), 27.7 (C-16), 25.8 (C-3), 23.9 (C-6), 22.7 (C-18), 18.4 (C-4), 14.2 (C-21).

HRMS (ESI-TOF) m/z: $[M+H]^+$ Calcd for $C_{21}H_{26}OH$ 295.2055; Found 295.2056.

IR (ATR, ṽ): 1693 cm^{-1} (s, *CO*).

Constitumer:

$C_{21}H_{26}O$ (294.44 $\frac{g}{mol}$)

R_f: 0.66 (*n*-hexane/EA = 80:20) [anisaldehyde]

^{1}H NMR(400.16 MHz, $CDCl_3$): δ = 7.55 (dd, ^3J = 8.1 Hz, ^4J = 0.7 Hz, 1H, H-10), 7.23 (ddm, ^3J = 7.6 Hz, ^3J = 7.6 Hz, 1H, H-11), 7.13 (ddd, ^3J = 7.5 Hz, ^3J = 7.3 Hz, ^4J = 1.3 Hz, 1H, H-12), 6.97 (dm, ^3J = 7.6 Hz, 1H, H-13), 4.49 (dm, ^2J = 11.5 Hz, 1H, H-15b), 3.20 (d, ^2J = 11.5 Hz, 1H, H-15a), 2.66 (s, 1H, H-8), 2.49 (m, 1H, H-2), 2.12 (m, 1H, H-16b), 1.99 (dm, ^2J = 11.3 Hz, 1H, H-5a), 1.65 (m, 1H, H-16a), 1.49 (m, 1H, H-4), 1.40 (dm, ^2J = 11.3 Hz, H-5b), 1.38 (m, 1H, H-6), 1.32 (m, 1H, H-3), 1.19 (m, 3H, H-17a/b, H-18), 1.18 (m, 4H, H-19, H-20), 0.90 (m, 1H, H-17b/a), 0.83 (t, ^3J = 6.8 Hz, 3H, H-21).

^{13}C NMR(100.62 MHz, $CDCl_3$): δ = 207.0 (C-1), 142.3 (C-9), 130.7 (C-13), 130.5 (C-14), 130.0 (C-10), 127.4 (C-11), 127.1 (C-12), 57.3 (C-2), 57.3 (C-7), 50.8 (C-15), 37.5 (C-8), 36.3 (C-16), 33.5 (C-5), 31.8 (C-19), 30.0 (C-20), 26.9 (C-3), 25.6 (C-17), 22.7 (C-18), 16.6 (C-4), 14.2 (C-21), 13.9 (C-6).

HRMS (ESI-TOF) m/z: $[M+H]^+$ Calcd for $C_{21}H_{26}OH$ 295.2056; Found 295.2057.

IR (ATR, ṽ): 1709 cm^{-1} (s, *CO*).

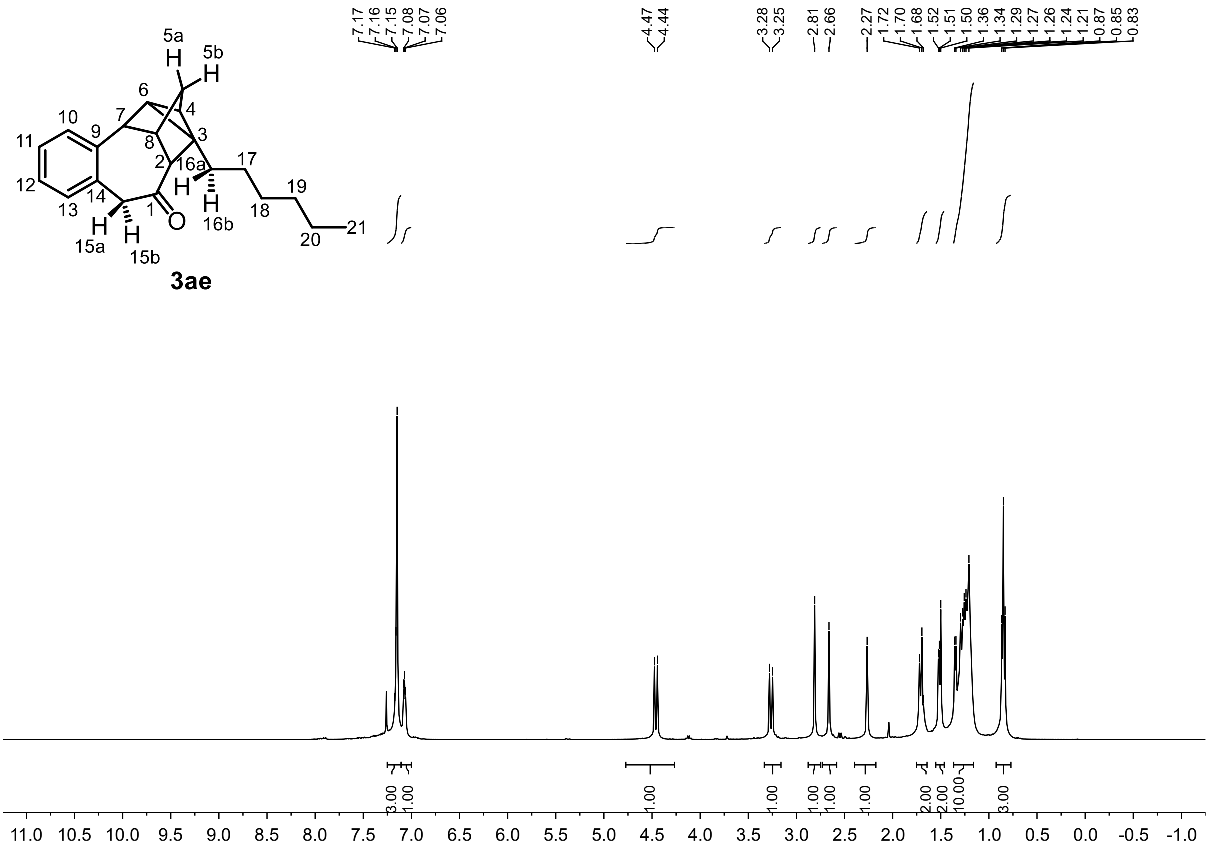

Figure S 158. ^{1}H NMR of **3ae** in $CDCl_3$ measured at 400.16 MHz.

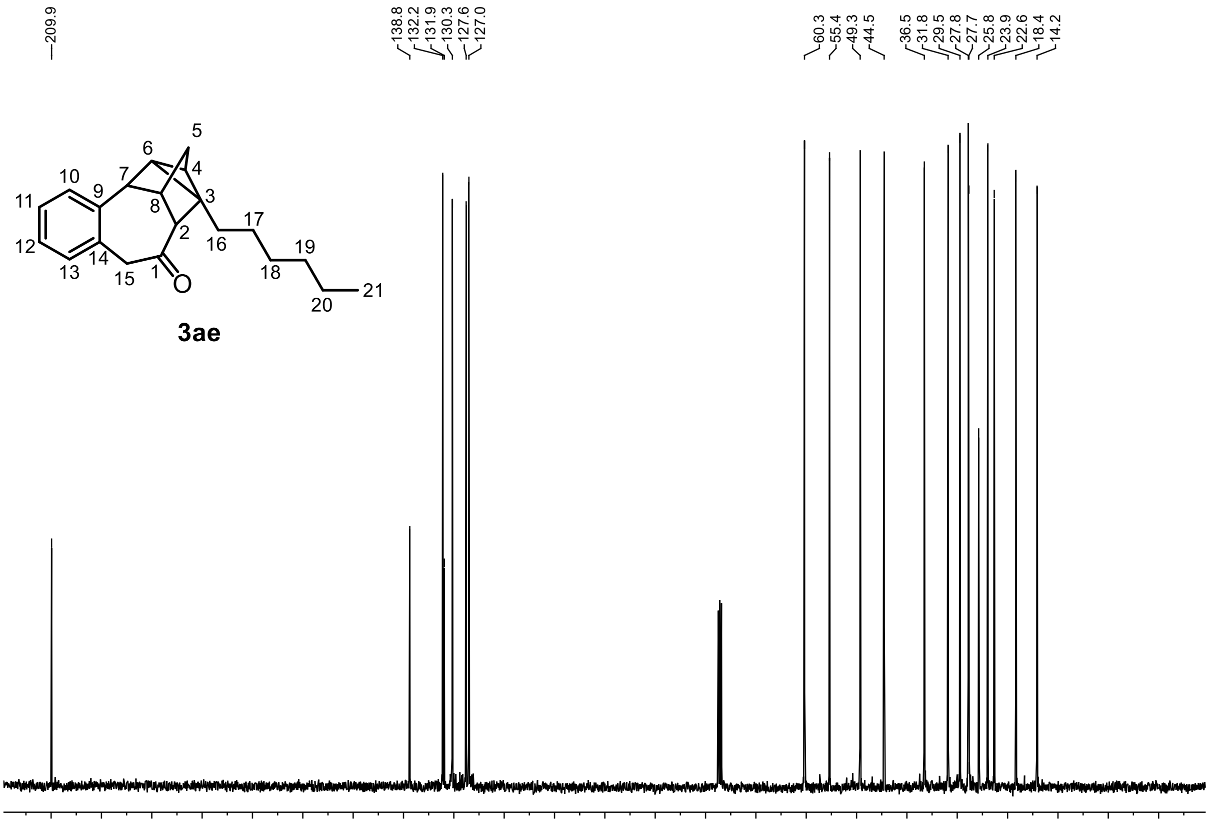

Figure S 159. ^{13}C NMR of **3ae** in $CDCl_3$ measured at 100.63 MHz.

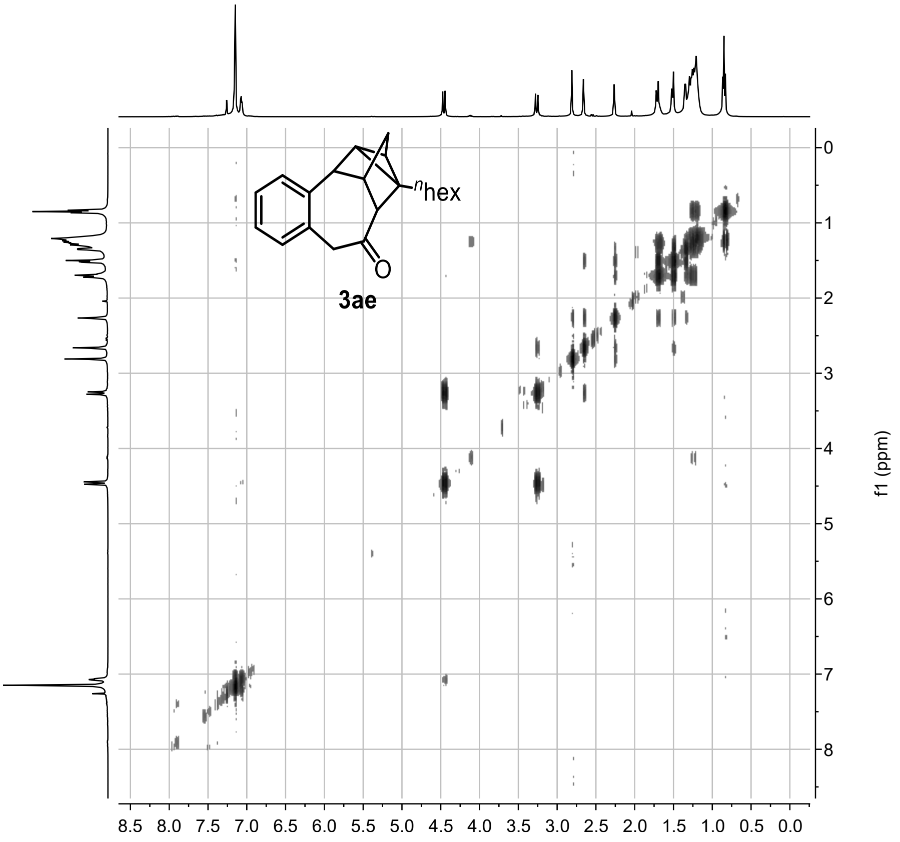

Figure S 160. $^{1}H,^{1}H$-COSY of **3ae** in $CDCl_3$ measured at 400.16 MHz.

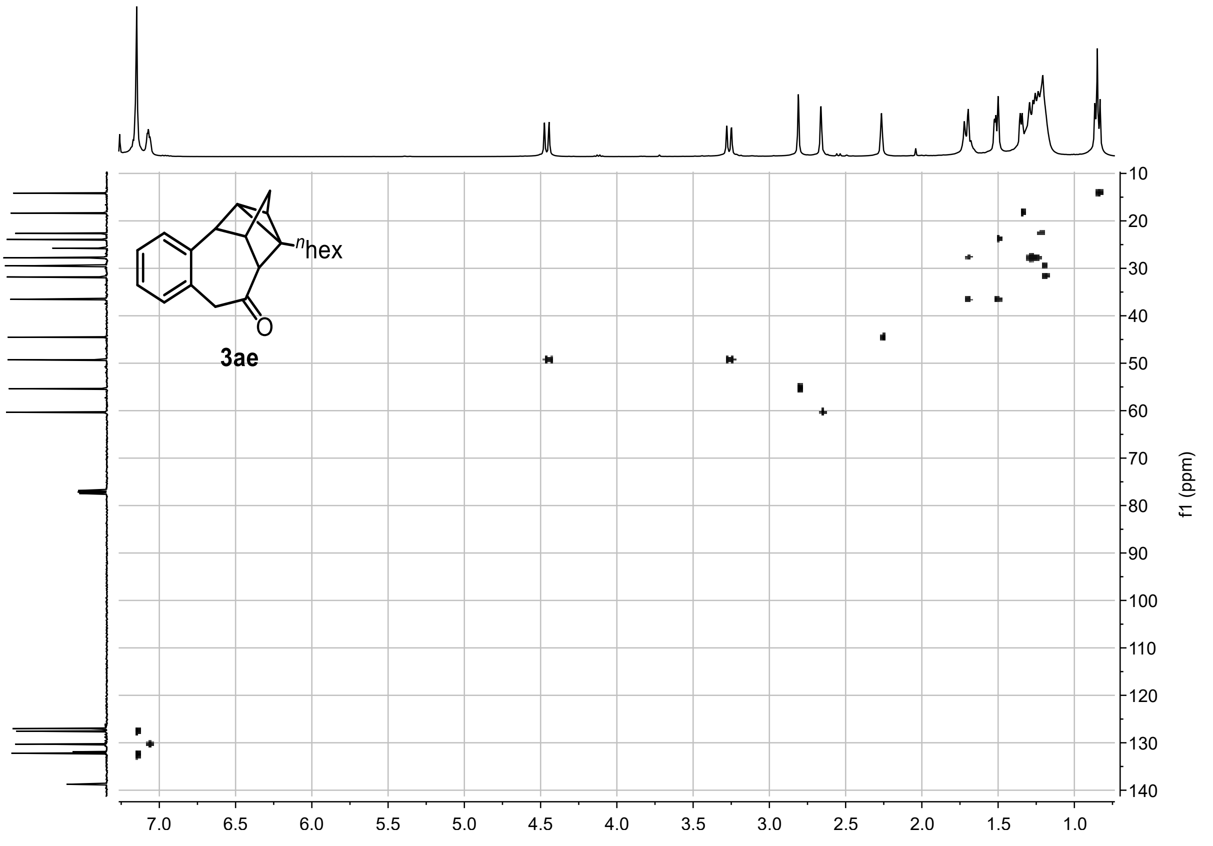

Figure S 161. $^{1}H,^{13}C$-HSQC of **3ae** in $CDCl_3$ measured at ^{1}H: 400.16 MHz; ^{13}C: 100.63 MHz.

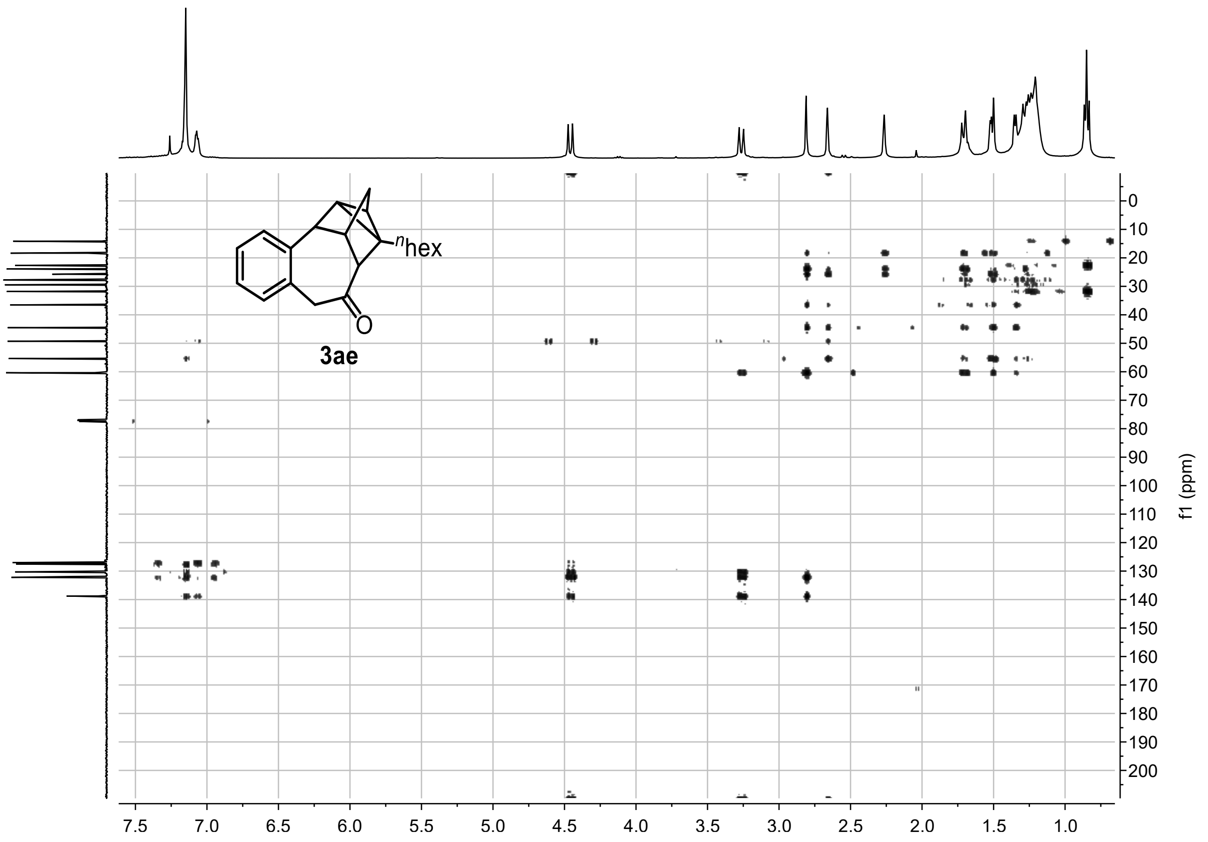

Figure S 162. ^{1}H,^{13}C-HMBC of **3ae** in $CDCl_3$ measured at ^{1}H: 400.16 MHz; ^{13}C: 100.63 MHz.

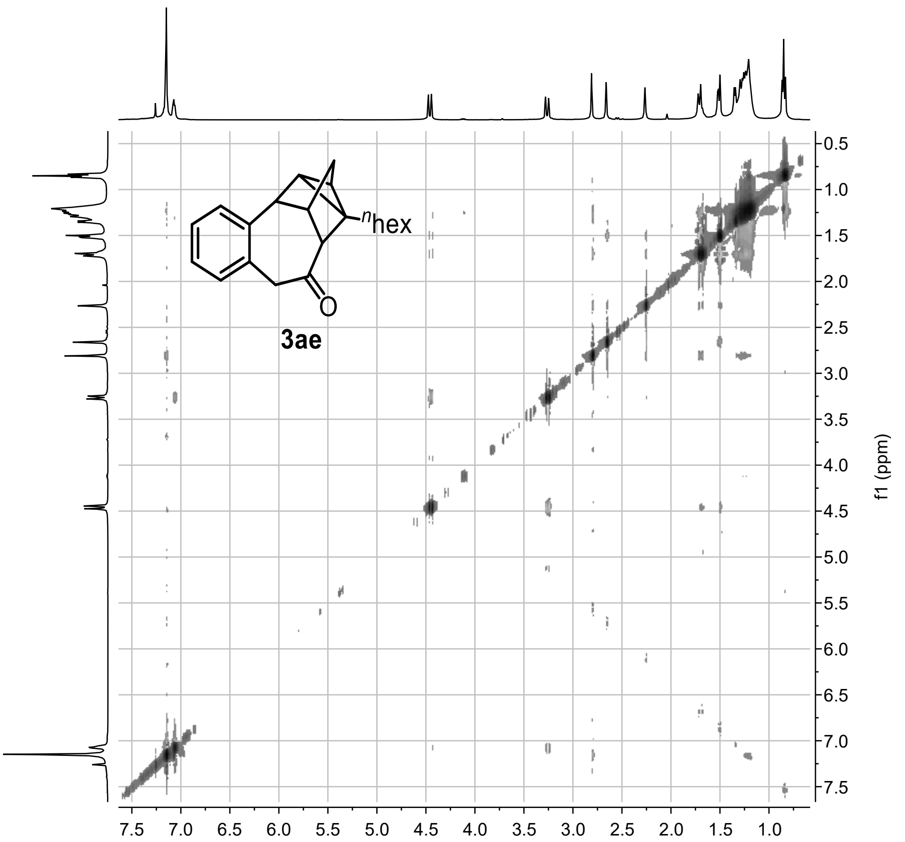

Figure S 163. ^{1}H,^{1}H-NOESY of **3ae** in $CDCl_3$ measured at 400.16 MHz.

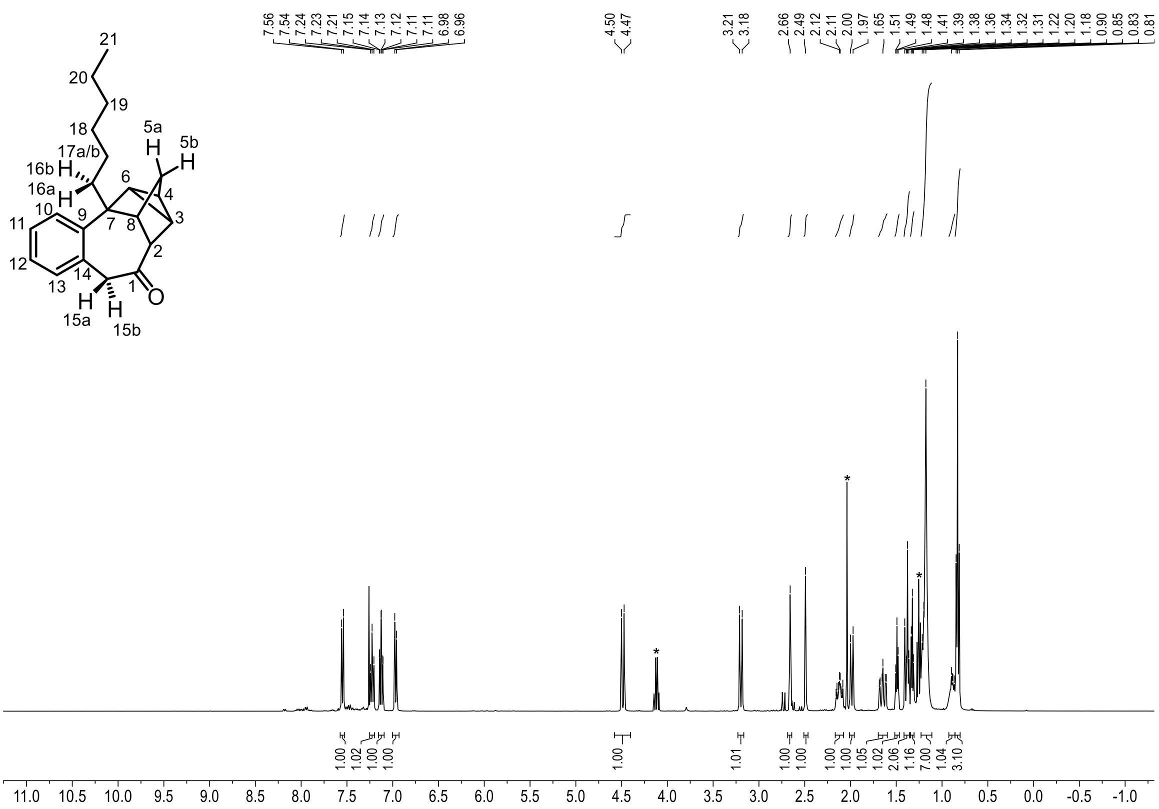

Figure S 164. ^{1}H NMR of the constitutional isomer of **3ae** in $CDCl_3$ measured at 400.16 MHz. * denotes residual EA.

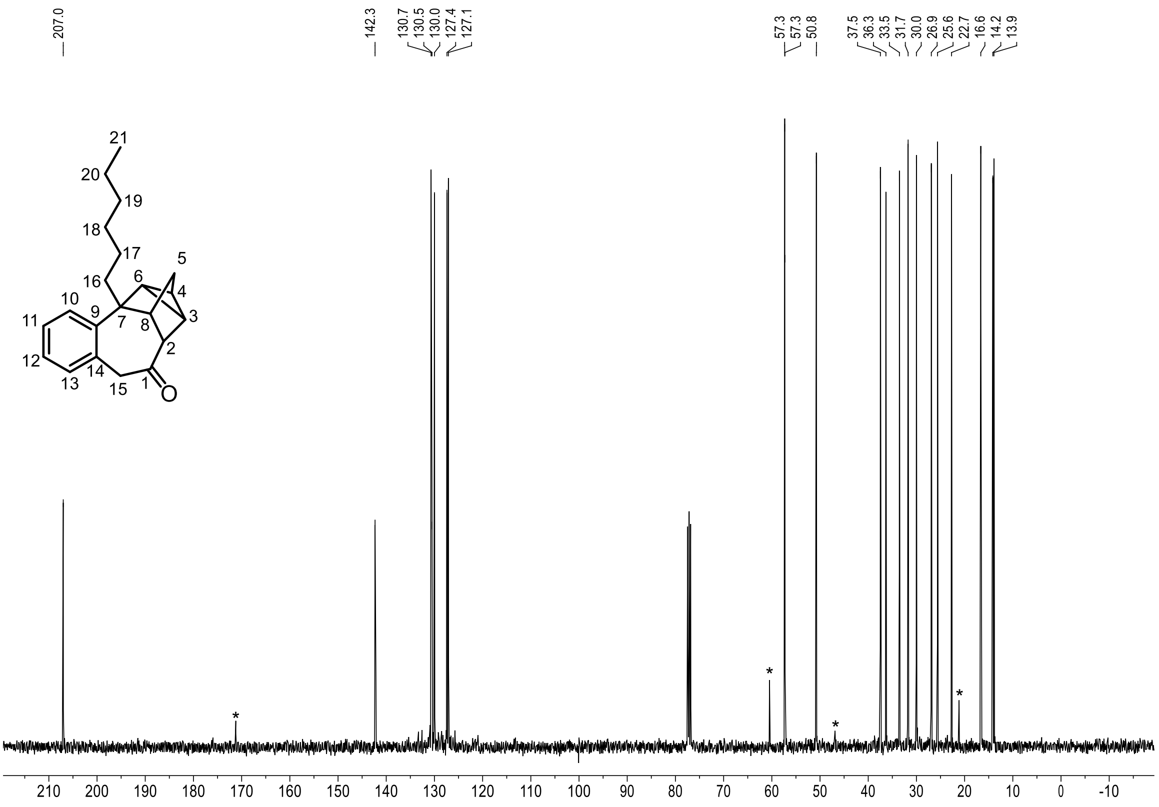

Figure S 165. ^{1}H NMR of the constitutional isomer of **3ae** in $CDCl_3$ measured at 400.16 MHz. * denotes residual EA.

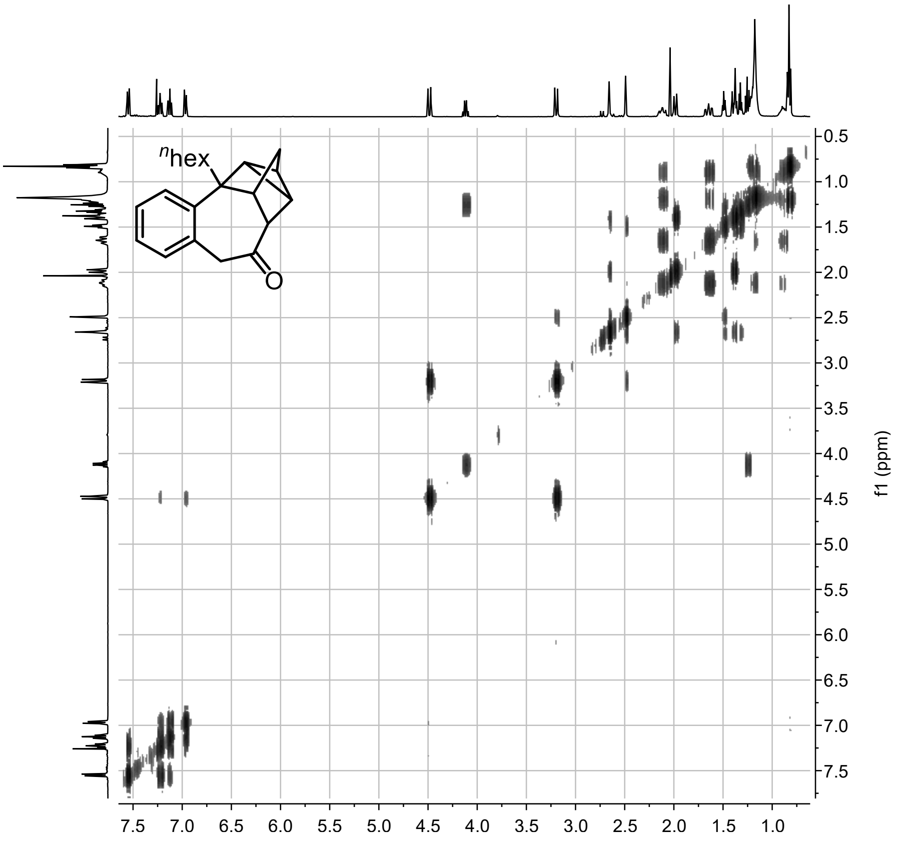

Figure S 166. $^{1}H,^{1}H$-COSY of the constitutional isomer of **3ae** in $CDCl_3$ measured at 400.16 MHz.

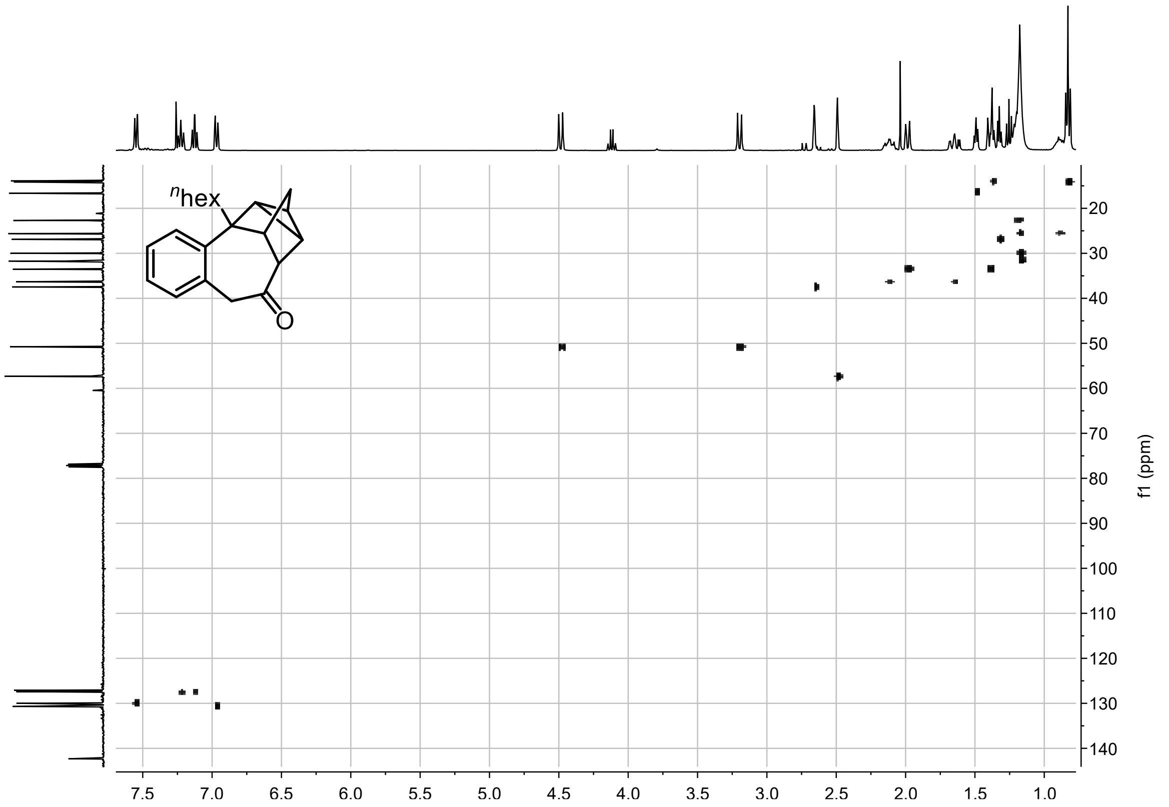

Figure S 167. $^{1}H,^{13}C$-HSQC of the constitutional isomer of **3ae** in $CDCl_3$ measured at ^{1}H: 400.16 MHz; ^{13}C: 100.63 MHz.

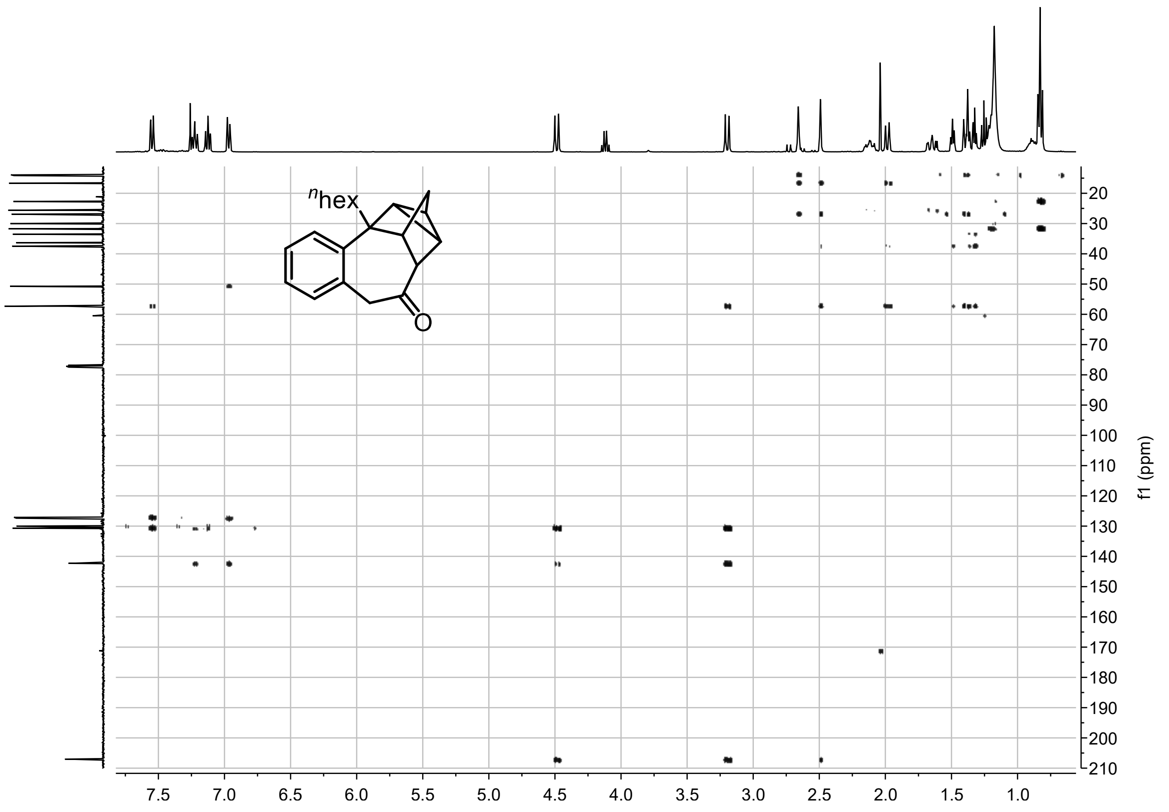

Figure S 168. $^{1}H,^{13}C$-HMBC of the constitutional isomer of **3ae** in $CDCl_3$ measured at ^{1}H: 400.16 MHz; ^{13}C: 100.63 MHz.

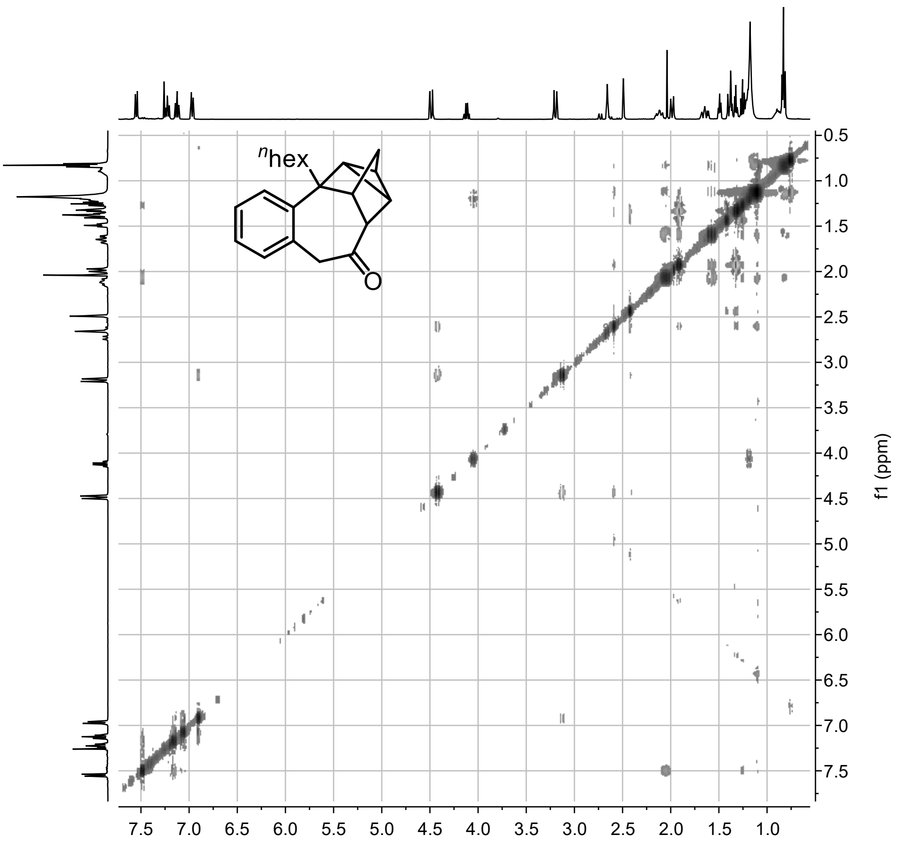

Figure S 169. $^{1}H,^{1}H$-NOESY of the constitutional isomer of **3ae** in $CDCl_3$ measured at 400.16 MHz.

***rel*-(2*S*,3*S*,3a*S*,10*R*,10a*R*,11*S*)-5-methyl-2,3,3a,9,10,10a-hexahydro-2,3,10-(epimethanetriyl)benzo[f]azulen-4(1H)-one**

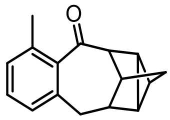

3ma

3ma was synthesized according to **GP-A** employing **1m** (132 mg, 1.00 mmol, 1.00 equiv.) and NBD (132 µL, 1.30 mmol, 1.30 equiv.). Purification *via* flash chromatography (23 g SiO_2, gradient from 100:00 to 85:15 *n*-hexane/EA over 15 CV) afforded **3ma** (167 mg, 745 µmol, 74%, 16% *ee*, $[\alpha]_D^{20}$ +1 (*c* 1.0, $CHCl_3$)) as a pale yellow oil.

isomer ratio: 81:19

$C_{16}H_{16}O$ (224.30 $\frac{g}{mol}$)

R_f: 0.84 (*n*-hexane/EA = 80:20) [UV]

^{1}H NMR(400.16 MHz, $CDCl_3$): δ = 7.13 (t, ^3J = 7.6 Hz, 1H, H-12,), 7.04 (d, ^3J = 7.7 Hz, 1H, H-13), 6.92 (d, ^3J = 7.4 Hz, 1H, H-11), 3.03 (d, ^2J = 15.7 Hz, 1H, H-9a), 2.90 (dd, ^2J = 15.7 Hz, ^3J = 7.7 Hz, 1H, H-9b), 2.57 (m, 1H, H-2), 2.27 (s, 3H, H-16), 2.19 (s, 1H, H-8), 2.11 (dm, ^3J = 7.7 Hz, 1H, H-7), 1.50 (dm, ^2J = 10.6 Hz, 1H, H-5b), 1.45 (dm, ^2J = 10.6 Hz, 1H, H-5a), 1.24 (m, 1H, H-4), 1.19 (m, 1H, H-3), 1.04 (m, 1H, H-6).

^{13}C NMR(100.62 MHz, $CDCl_3$): δ = 211.9 (C-1), 140.5 (C-15), 135.5 (C-10), 134.4 (C-14), 129.2 (C-12), 128.9 (C-13), 127.2 (C-11), 57.1 (C-2), 41.9 (C-8), 41.3 (C-7), 36.5 (C-5), 35.7 (C-9), 19.7 (C-16), 15.4 (C-6), 14.9 (C-3), 13.1 (C-4).

HRMS (ESI-TOF) m/z: $[M+H]^+$ Calcd for $C_{16}H_{16}OH$ 225.1274; Found 225.1278.

IR (ATR, ṽ): 1679 cm^{-1} (s, *CO*).

Regioisomer:

mp: 131.6 °C.

R_f: 0.60 (*n*-hexane/EA = 80:20) [anisaldehyde]

^{1}H NMR(600.13 MHz, $CDCl_3$): δ = 7.08 (d, ^3J = 6.9 Hz, 1H, H-11,), 7.03 (t, ^3J = 7.5 Hz, 1H, H-12), 6.92 (d, ^3J = 7.3 Hz, 1H, H-13), 4.06 (d, ^2J = 12.2 Hz, 1H, H-15b), 3.73 (d, ^2J = 12.2 Hz, 1H, H-15a), 3.27 (s, 1H, H-7), 2.62 (m, 1H, H-2), 2.43 (m, 1H, H-8), 2.41 (s, 3H, H-16), 1.66

(dm, ^2J =10.7 Hz, 1H, H-5a), 1.63 (m, 1H, H-3), 1.55 (dm, ^2J = 10.7 Hz, 1H, H-5b), 1.47 (m, 1H, H-4), 1.44 (m, 1H, H-6).

^{13}C NMR(150.90 MHz, $CDCl_3$): δ = 209.5 (C-1), 137.5 (C-9), 137.4 (C-14), 134.7 (C-10), 129.7 (C-11), 128.9 (C-13), 127.0 (C-12), 58.0 (C-2), 49.8 (C-15), 46.8 (C-7), 38.7 (C-8), 35.9 (C-5), 21.2 (C-16), 16.6 (C-3), 15.8 (C-6), 13.1 (C-4).

HRMS (ESI-TOF) m/z: $[M+H]^+$ Calcd for $C_{16}H_{16}OH$ 225.1274; Found 225.1277.

IR (ATR, ṽ): 1698 cm^{-1} (s, *CO*).

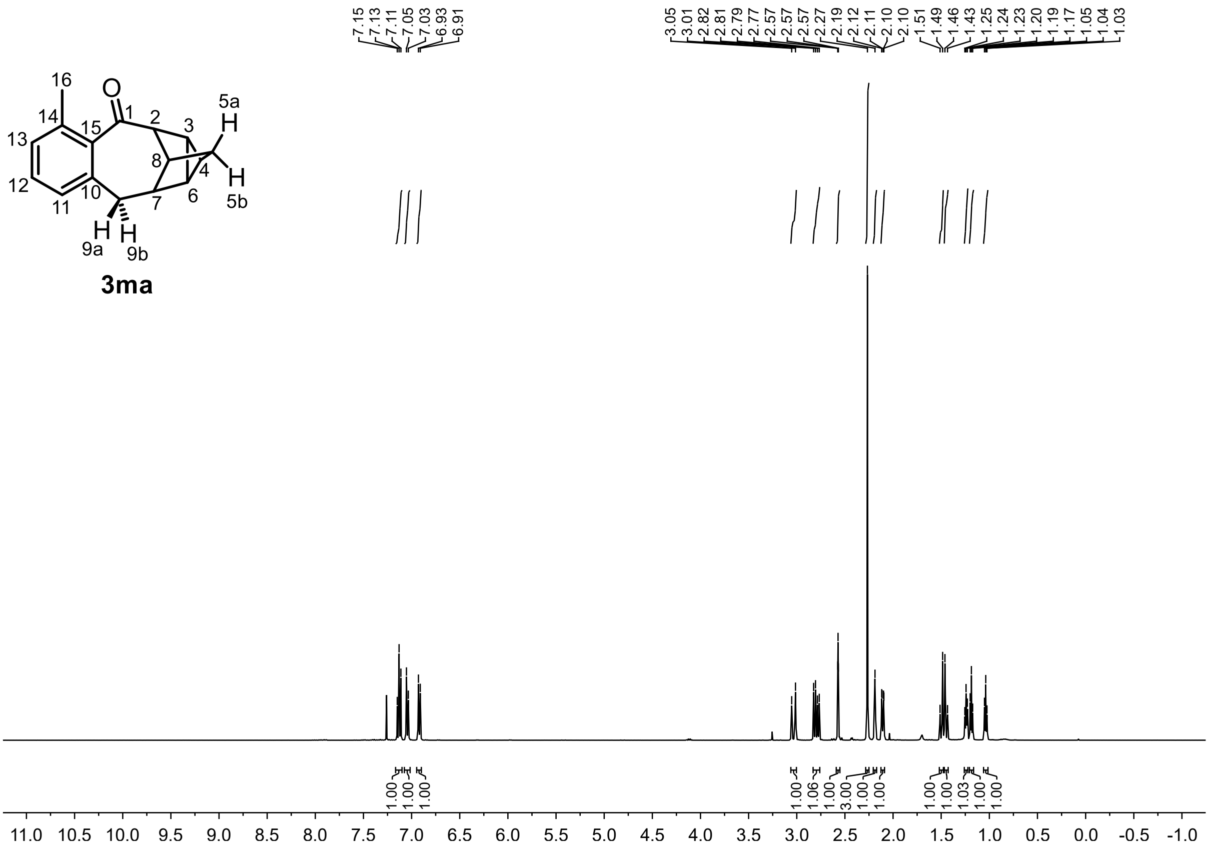

Figure S 170. ^{1}H NMR of **3ma** in $CDCl_3$ measured at 400.16 MHz.

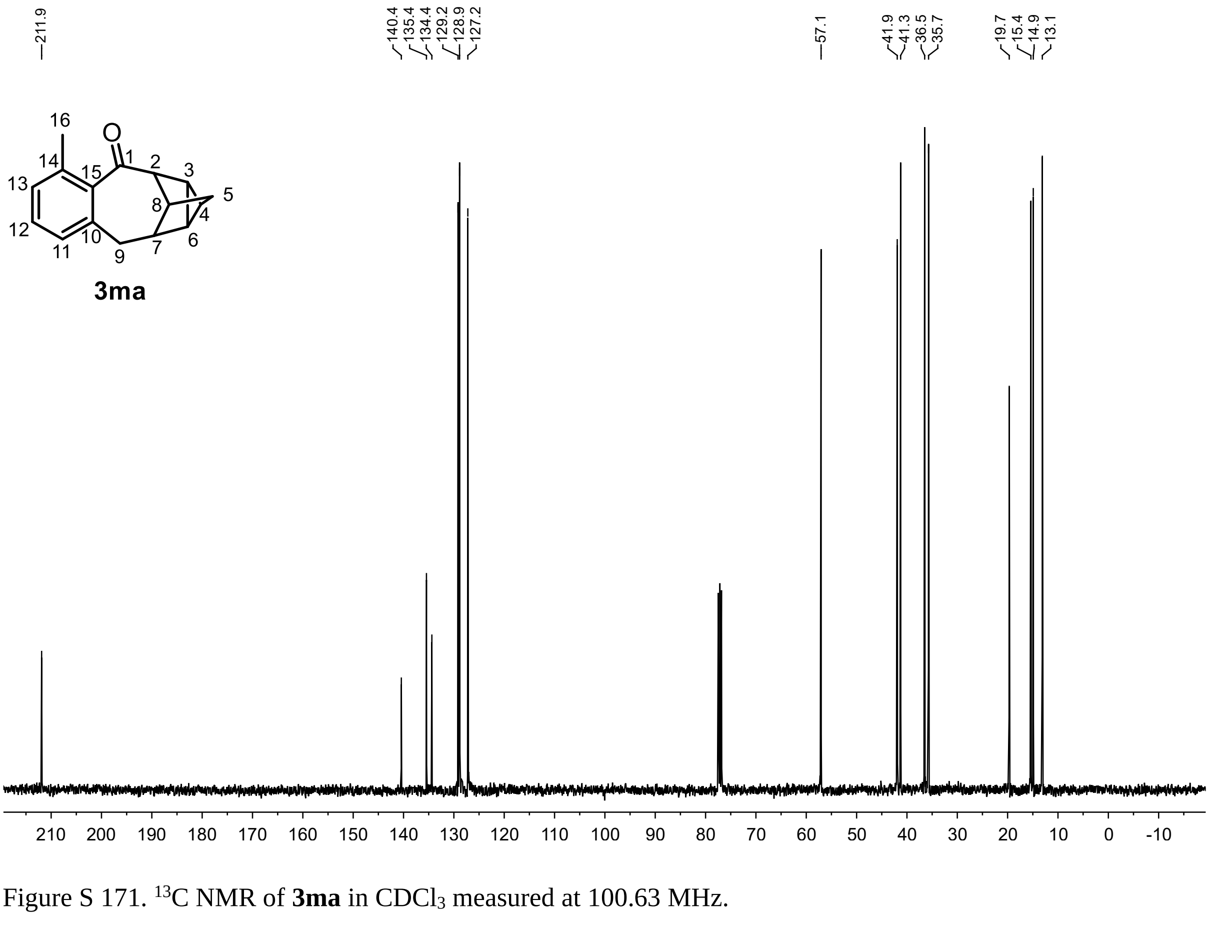

Figure S 171. ^{13}C NMR of **3ma** in $CDCl_3$ measured at 100.63 MHz.

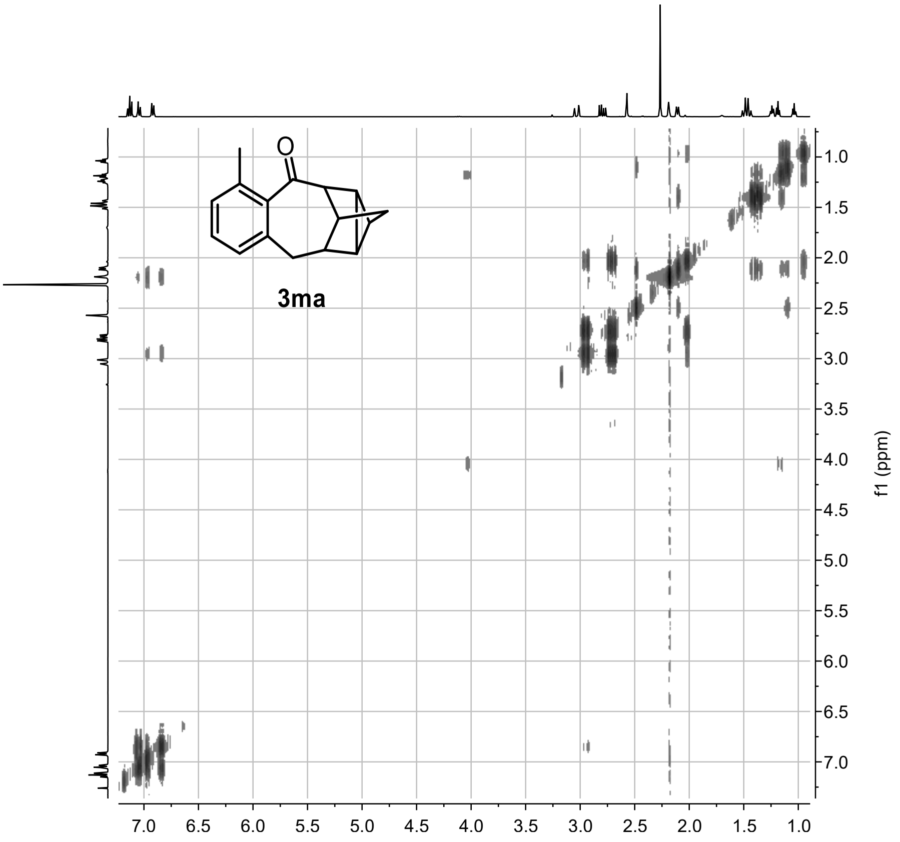

Figure S 172. $^{1}H,^{1}H$-COSY of **3ma** in $CDCl_3$ measured at 400.16 MHz.

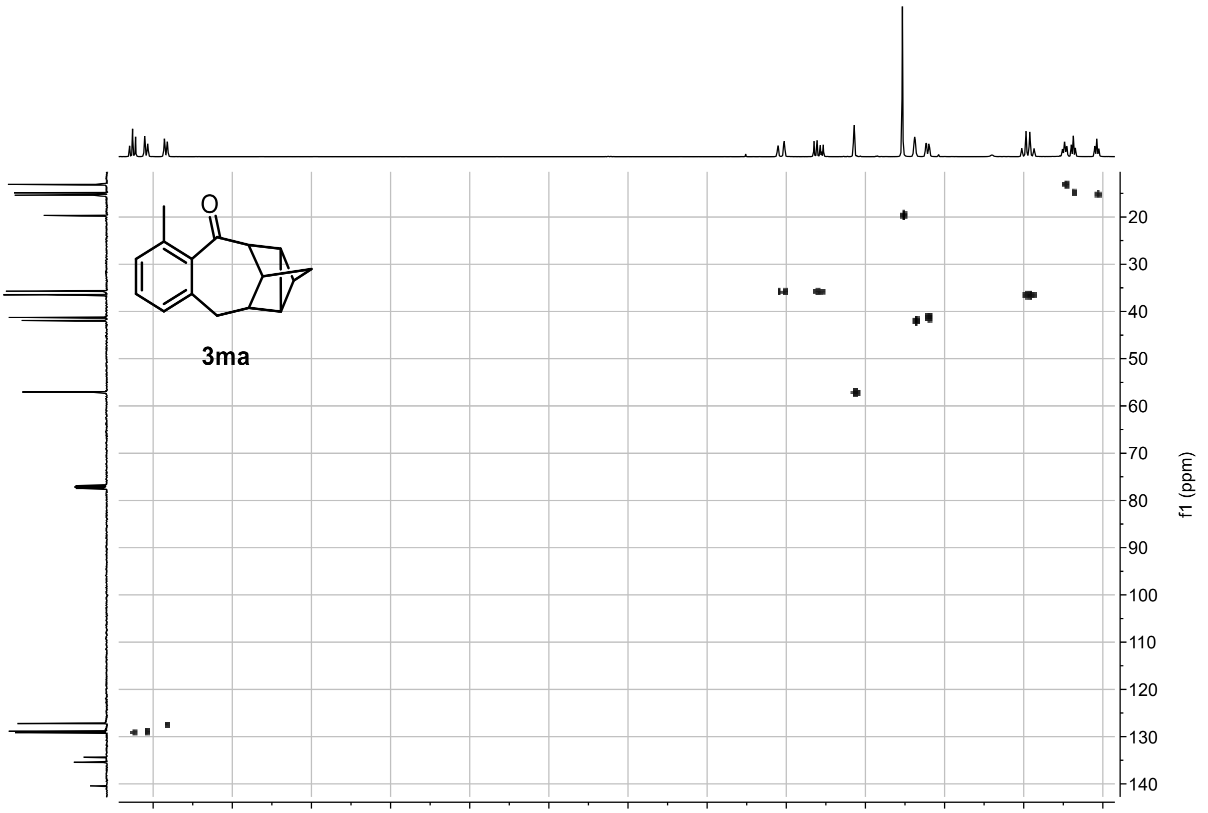

Figure S 173. $^{1}H,^{13}C$-HSQC of **3ma** in $CDCl_3$ measured at ^{1}H: 400.16 MHz; ^{13}C: 100.63 MHz.

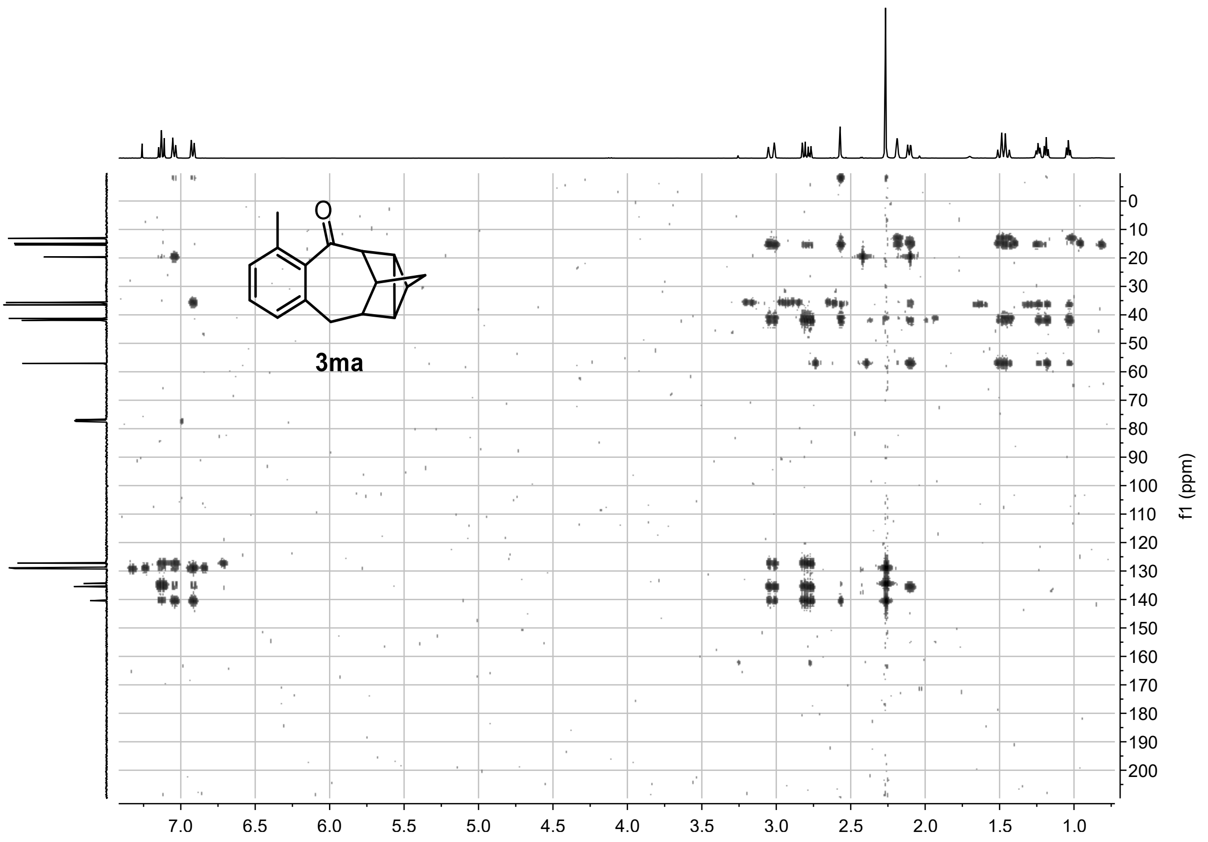

Figure S 174. $^{1}H,^{13}C$-HMBC of **3ma** in $CDCl_3$ measured at ^{1}H: 400.16 MHz; ^{13}C: 100.63 MHz.

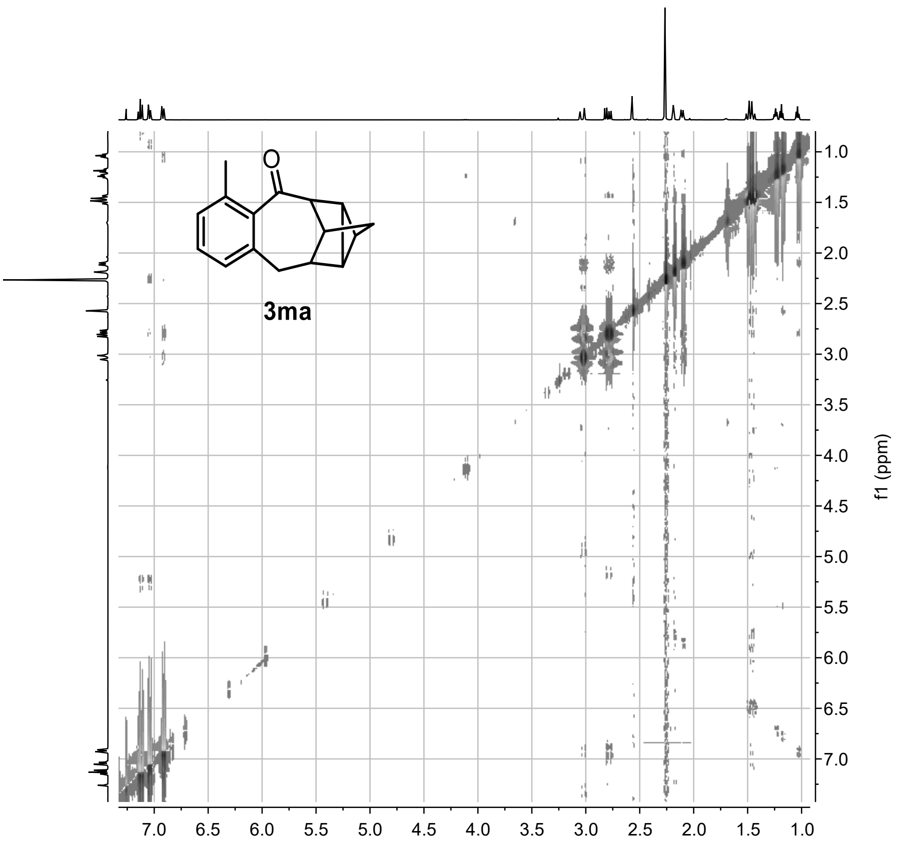

Figure S 175. $^{1}H,^{1}H$-NOESY of **3ma** in $CDCl_3$ measured at 400.16 MHz.

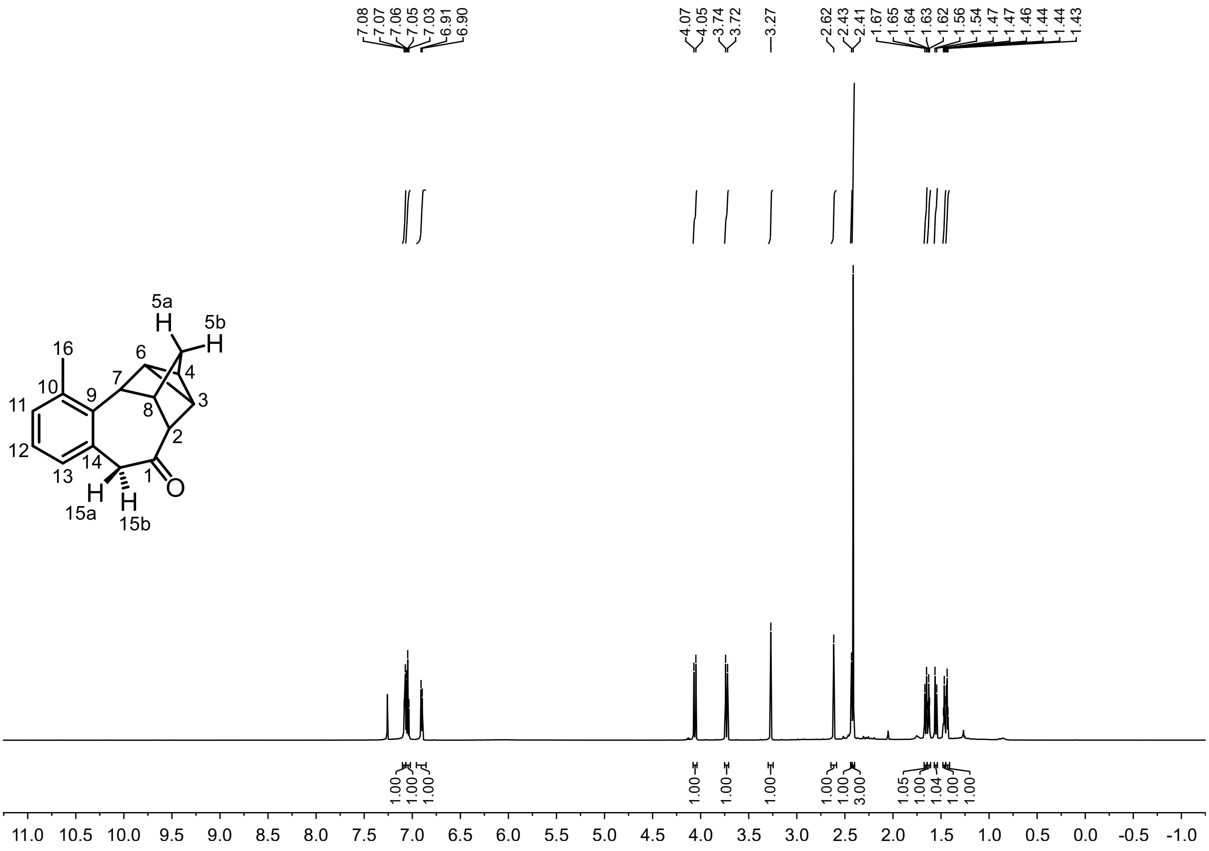

Figure S 176. ^{1}H NMR of the regioisomer of **3ma** in $CDCl_3$ measured at 600.13 MHz.

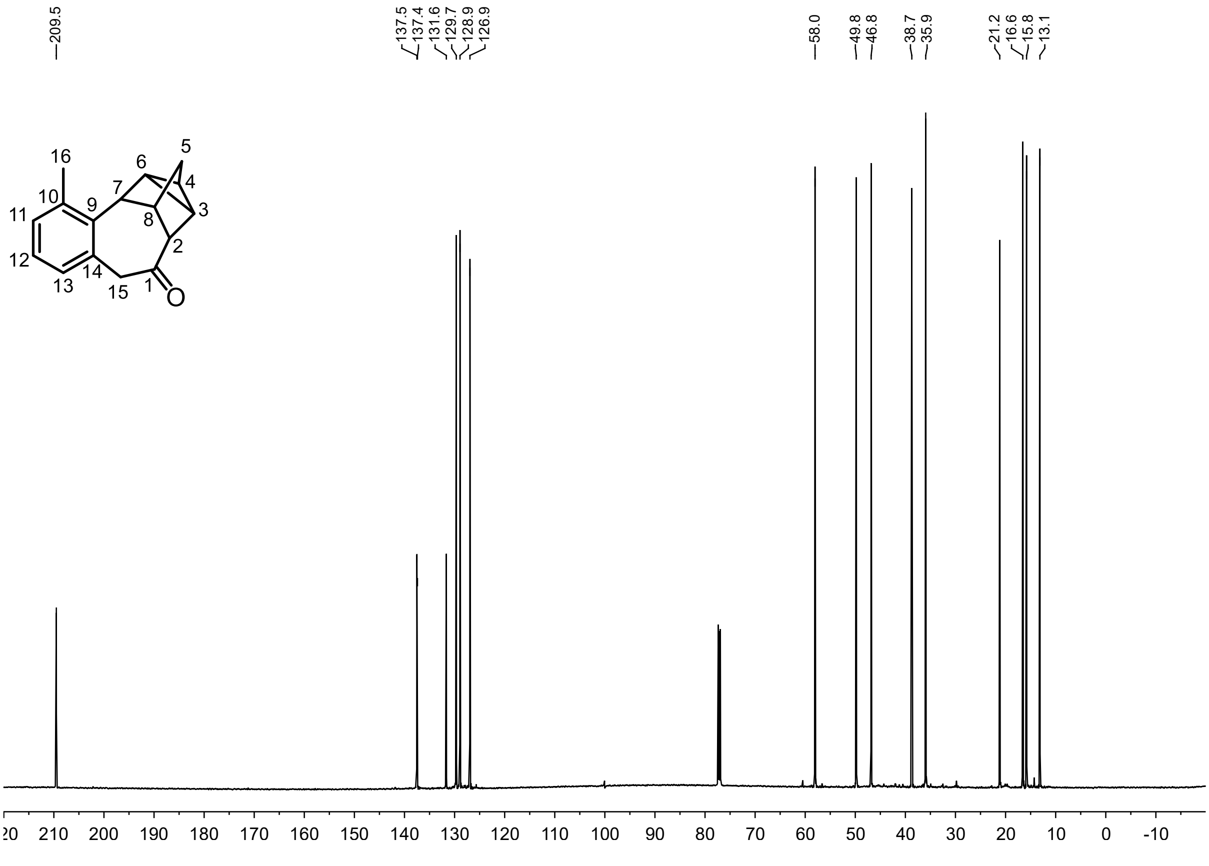

Figure S 177. ^{13}C NMR of the regioisomer of **3ma** in $CDCl_3$ measured at 150.92 MHz.

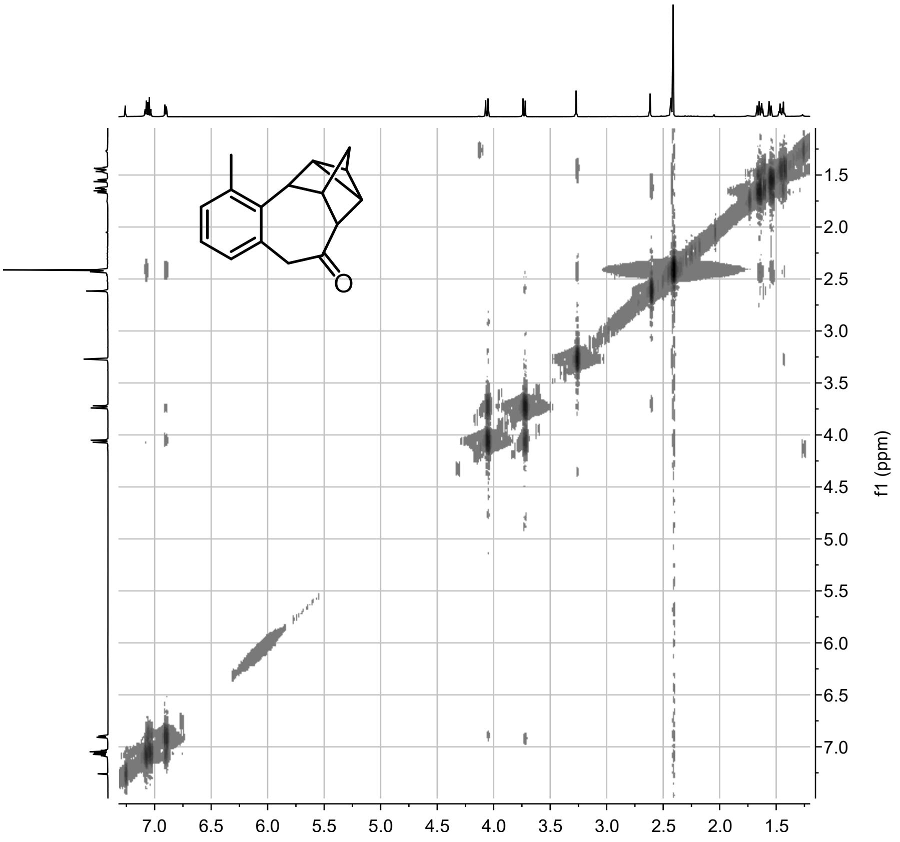

Figure S 178. ^{1}H,^{1}H-COSY of the regioisomer of **3ma** in $CDCl_3$ measured at 600.13 MHz.

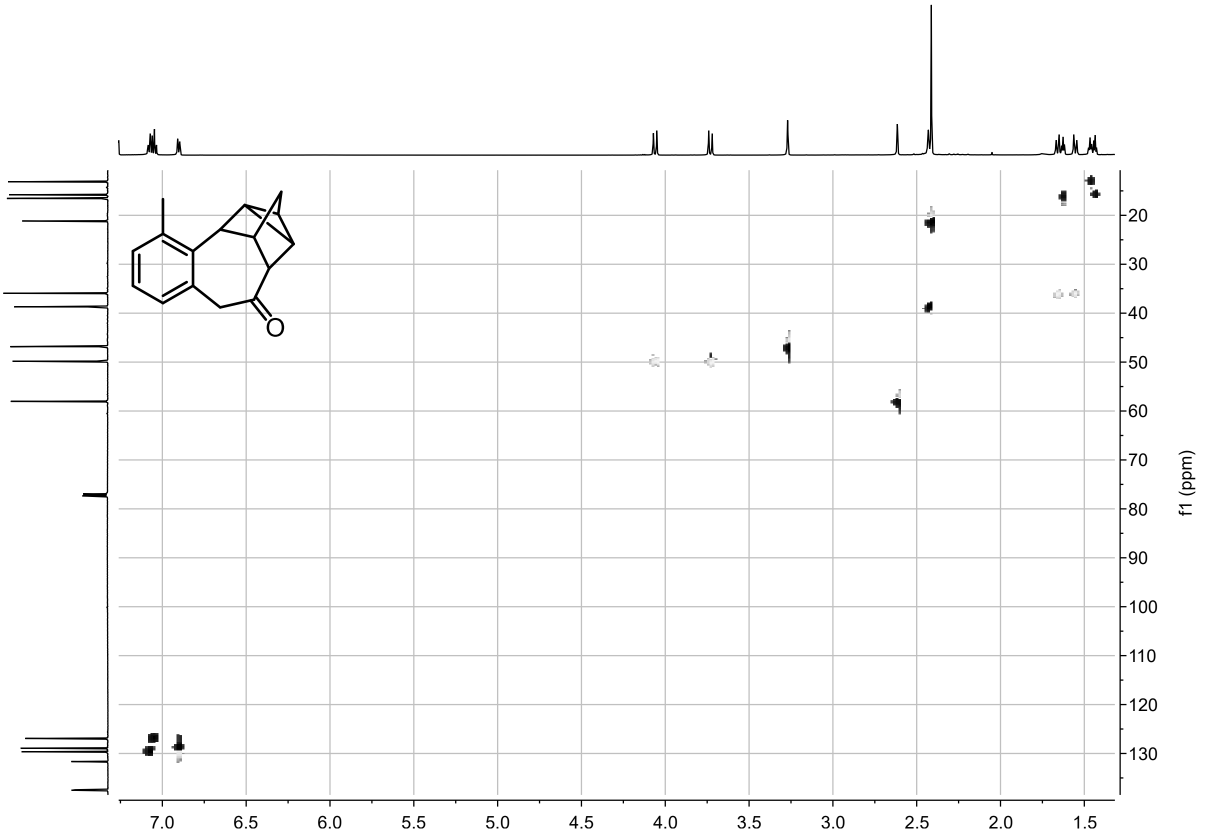

Figure S 179. ^{1}H,^{13}C-HSQC of the regioisomer of **3ma** in $CDCl_3$ measured at ^{1}H: 600.13 MHz; ^{13}C: 150.92 MHz.

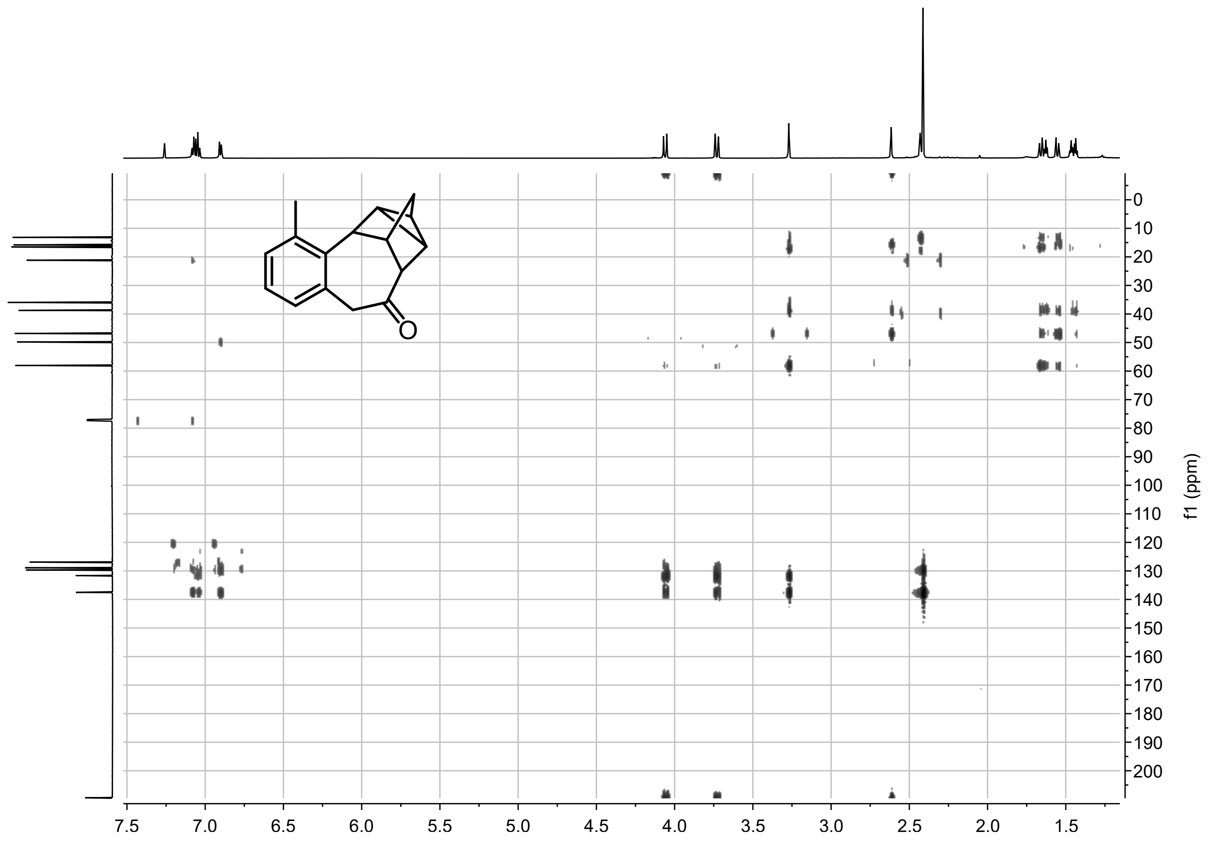

Figure S 180. $^{1}H,^{13}C$-HMBC of the regioisomer of **3ma** in $CDCl_3$ measured at ^{1}H: 600.13 MHz; ^{13}C: 150.92 MHz.

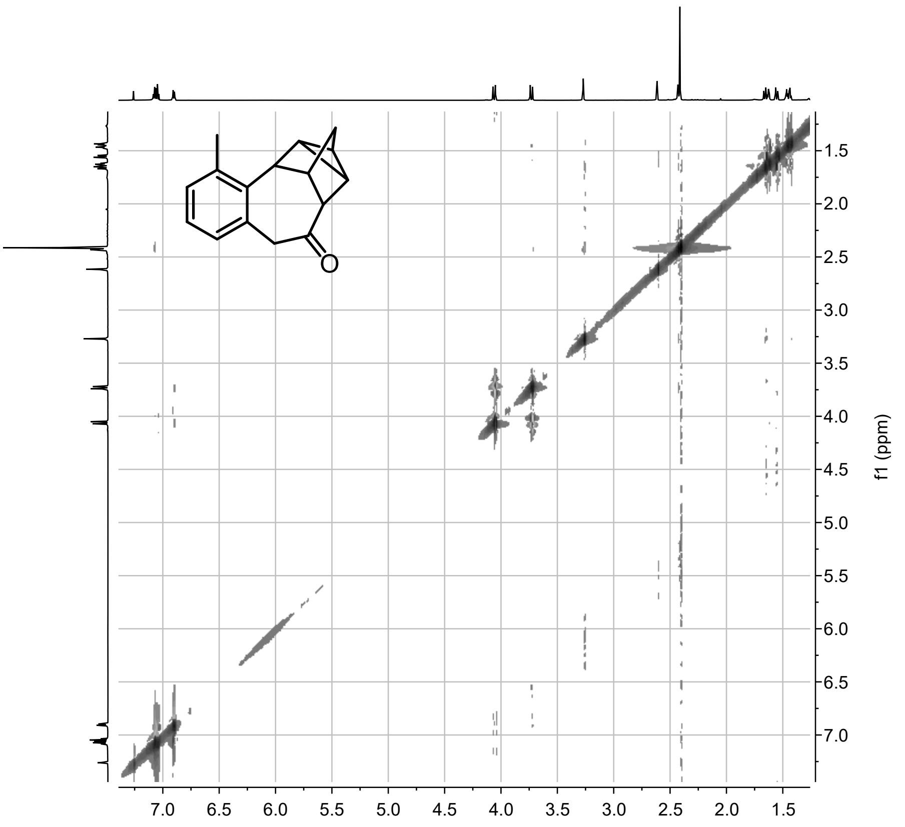

Figure S 181. $^{1}H,^{1}H$-NOESY of the regioisomer of **3ma** in $CDCl_3$ measured at 600.13 MHz.

***rel*-(2*S*,3*S*,3a*S*,10*R*,10a*R*,11*S*)-5-methoxy-2,3,3a,9,10,10a-hexahydro-2,3,10-(epimethanetriyl)benzo[f]azulen-4(1H)-one**

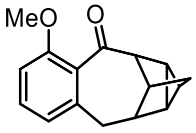

3na

3na was synthesized according to **GP-B** employing **1n** (148 mg, 1.00 mmol, 1.00 equiv.) and NBD (132 µL, 1.30 mmol, 1.30 equiv.). Purification *via* flash chromatography (23 g SiO_2, gradient from 100:00 to 80:20 *n*-hexane/EA over 15 CV) afforded **3na** (240 mg, 663 µmol, 66%, mixture of isomers) as a pale yellow oil.

isomeric ratio: 93:7 ([4+2+2]:[4+2])

$C_{16}H_{16}O_2$ (240.30 $\frac{g}{mol}$)

R$_f$: 0.52 (*n*-hexane/EA = 80:20) [UV]

^{1}H NMR(400.16 MHz, $CDCl_3$): δ = 7.18 (t, ^3J = 7.9 Hz, 1H, H-12,), 6.78 (d, ^3J = 8.4 Hz, 1H, H-13), 6.68 (d, ^3J = 7.6 Hz, 1H, H-11), 3.78 (s, 3H, H-16), 3.01 (d, ^2J = 15.8 Hz, 1H, H-9a), 2.78 (dd, ^2J = 15.8 Hz, ^3J = 7.7 Hz, 1H, H-9b), 2.59 (m, 1H, H-2), 2.16 (s, 1H, H-8), 2.08 (dm, ^3J = 7.7 Hz, 1H, H-7), 1.48 (dm, ^2J = 10.6 Hz, 1H, H-5b), 1.43 (dm, ^2J = 10.6 Hz, 1H, H-5a), 1.25 (m, 1H, H-3), 1.22 (m, 1H, H-4), 1.01 (m, 1H, H-6).

^{13}C NMR(100.62 MHz, $CDCl_3$): δ = 208.7 (C-1), 155.7 (C-14), 137.0 (C-10), 130.3 (C-13), 129.9 (C-15), 122.0 (C-11), 109.7 (C-12), 57.0 (C-2), 56.1 (C-16), 41.9 (C-8), 41.1 (C-7), 36.5 (C-5), 35.4 (C-9), 15.1 (C-6), 14.9 (C-3), 12.9 (C-4).

HRMS (ESI-TOF) m/z: $[M+H]^+$ Calcd for $C_{16}H_{16}O_2H$ 241.1223; Found 241.1224.

IR (ATR, ṽ): 1684 cm^{-1} (s, *CO*), 1583 cm^{-1} (m).

[2+2] insertion product:

^{1}H NMR(400.16 MHz, $CDCl_3$): δ = 7.31 (dd, ^3J = 8.2 Hz, ^3J = 7.7 Hz, 1H, H-12,), 6.80 (d, ^3J = 8.2 Hz, 1H, H-13), 6.75 (d, ^3J = 7.7 Hz, 1H, H-11), 6.18 (m, 2H, H-4, H-6), 3.83 (s, 3H, H-16), 3.48 (m, 1H, H-3), 2.96 (dd, ^2J = 14.8 Hz, ^3J = 6.5 Hz, 1H, H-9), 2.69 (m, 1H, H-7), 2.42 (dd, ^2J = 14.8 Hz, ^3J = 10.8 Hz, 1H, H-9), 2.31 (m, 1H, H-2), 2.25 (dm, ^3J = 10.8 Hz, 1H, H-7), 1.41 (m, 2H, H-5).

^{13}C NMR(100.62 MHz, $CDCl_3$): δ = 201.6 (C-1), 157.1 (C-14), 144.0 (C-15), 137.2 (C-4/6), 136.8 (C-6/4), 133.1 (C-12), 126.1 (C-10), 120.1 (C-11), 109.9 (C-13), 55.9 (C-16), 53.3 (C-2), 47.7 (C-7), 43.5 (C-3), 37.8 (C-8), 34.3 (C-9), 14.3 (C-5).

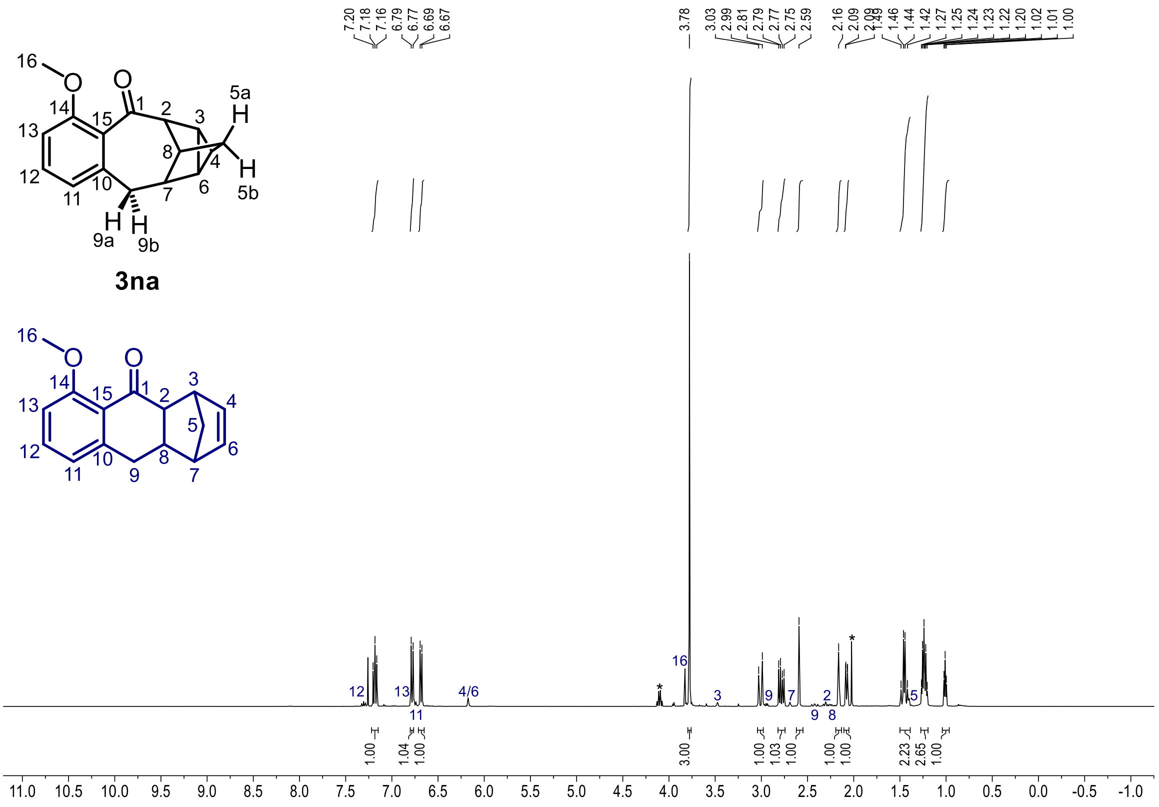

Figure S 182. ^{1}H NMR of **3na** and alken insertion product (blue) in $CDCl_3$ measured at 400.16 MHz. * denotes residual EA.

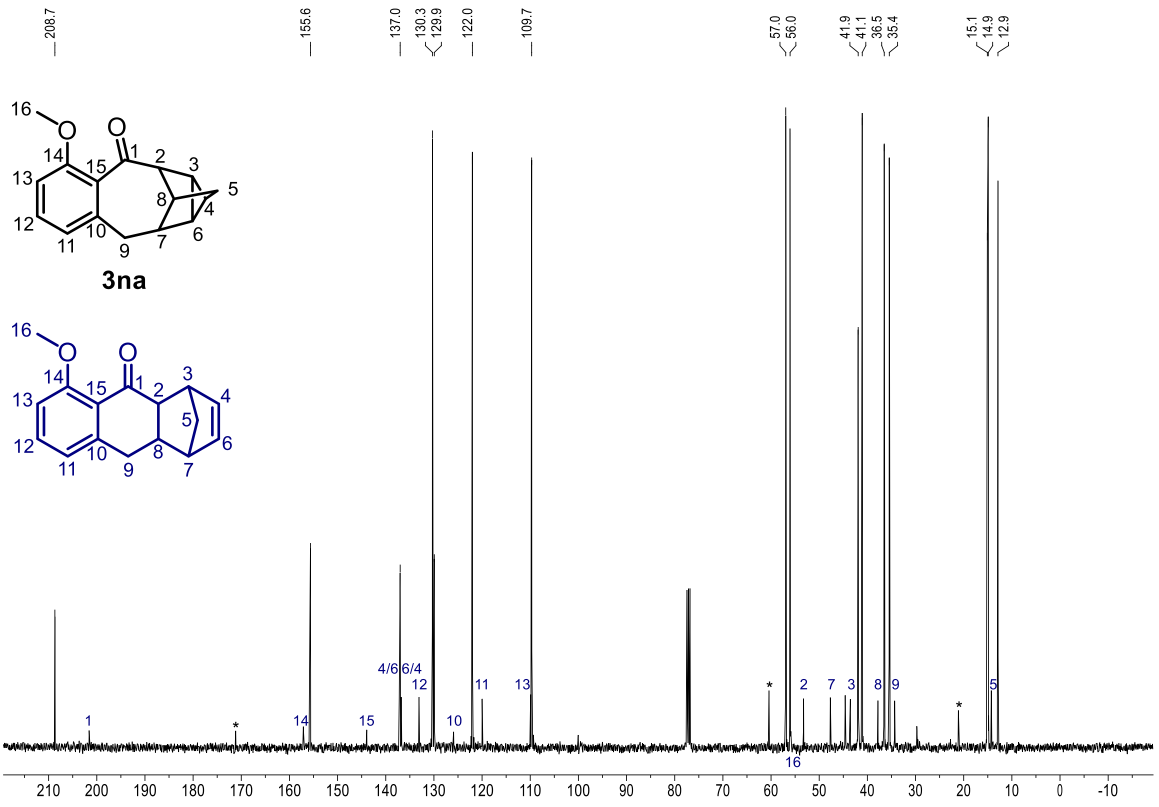

Figure S 183. ^{13}C NMR of **3na** and alken insertion product (blue) in $CDCl_3$ measured at 100.63 MHz. * denotes residual EA.

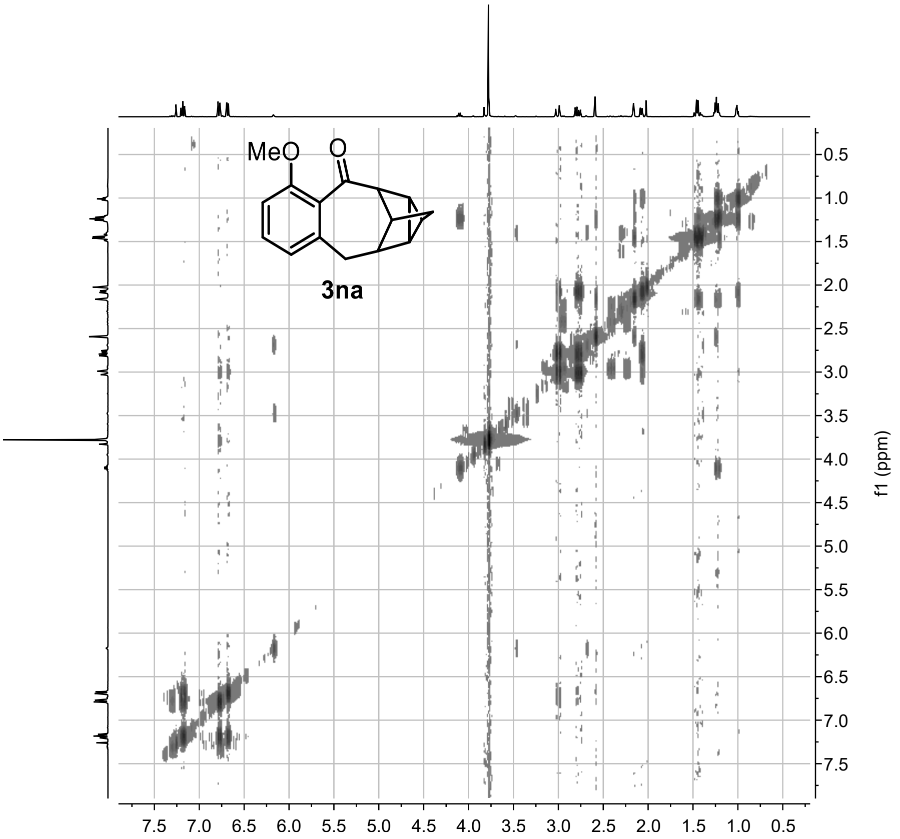

Figure S 184. ^{1}H,^{1}H-COSY of **3na** in $CDCl_3$ measured at 400.16 MHz.

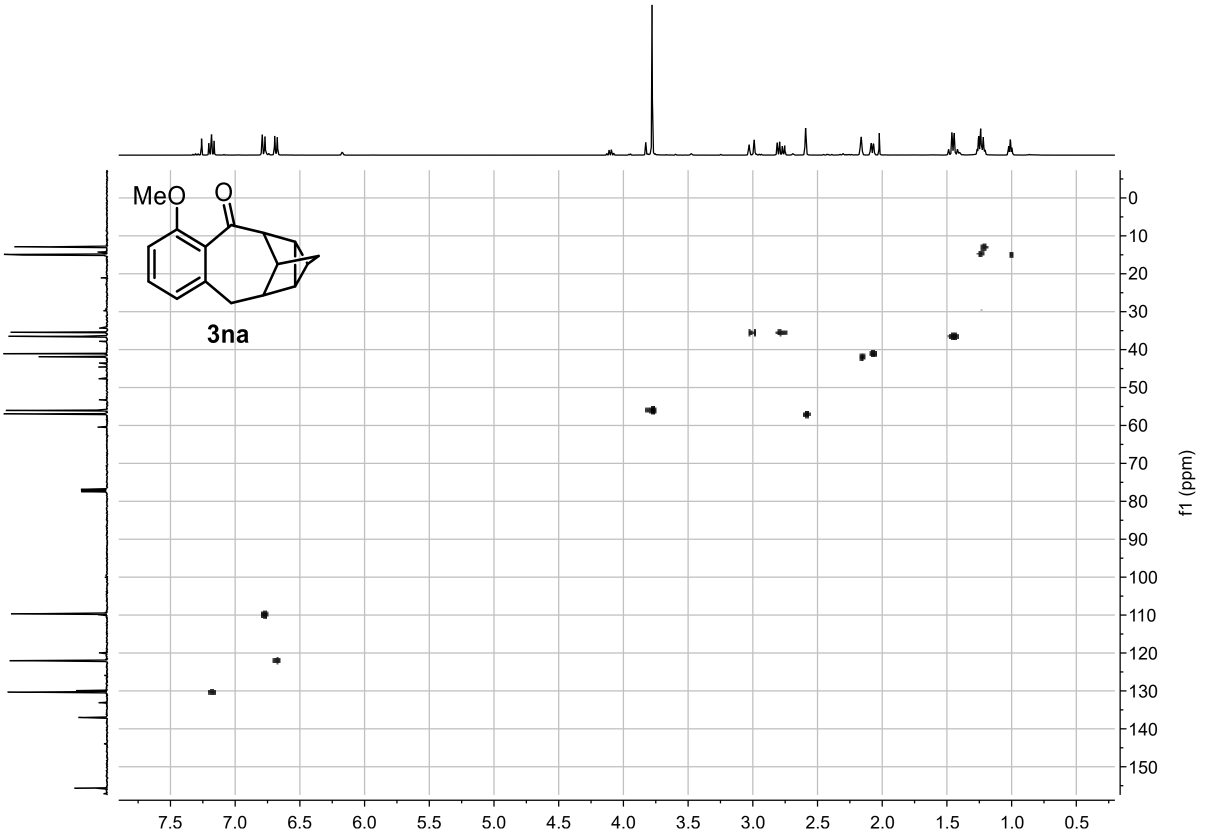

Figure S 185. ^{1}H,^{13}C-HSQC of **3na** in $CDCl_3$ measured at ^{1}H: 400.16 MHz; ^{13}C: 100.63 MHz.

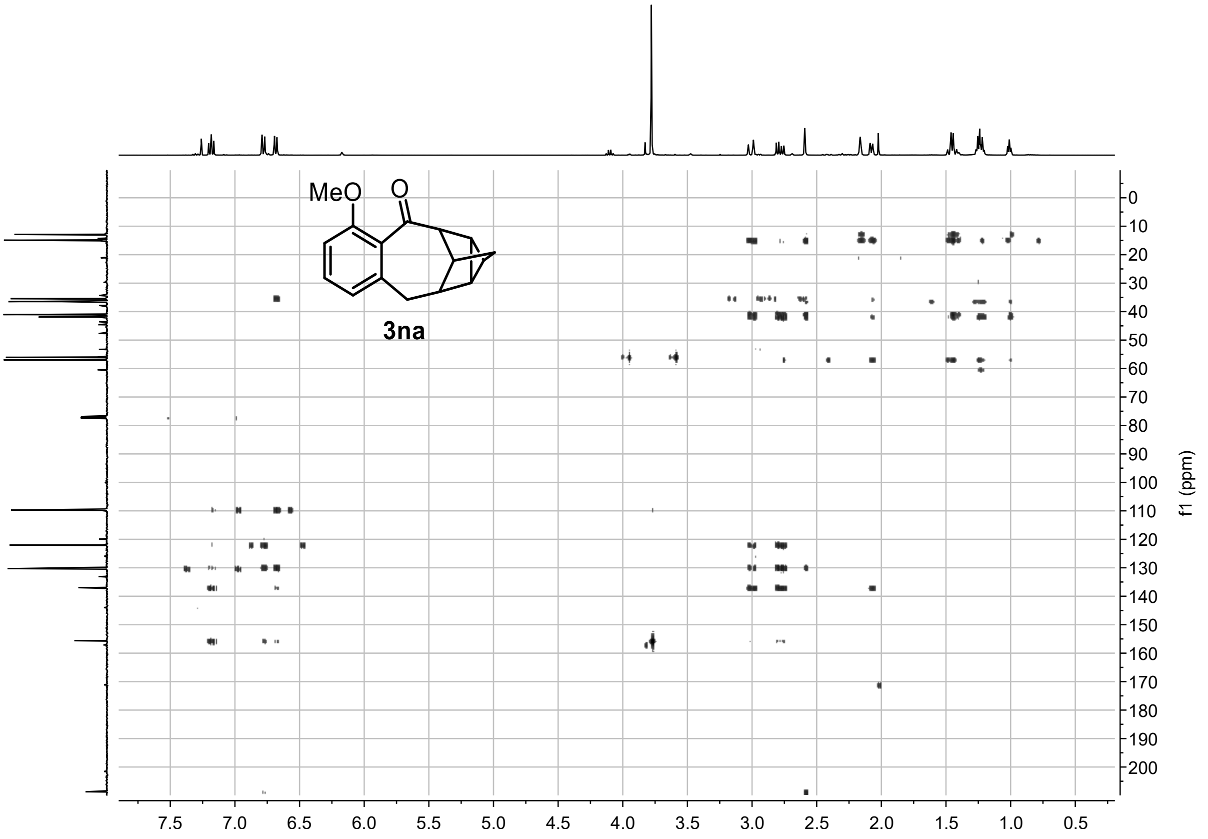

Figure S 186. $^{1}H,^{13}C$-HMBC of **3na** in $CDCl_3$ measured at ^{1}H: 400.16 MHz; ^{13}C: 100.63 MHz.

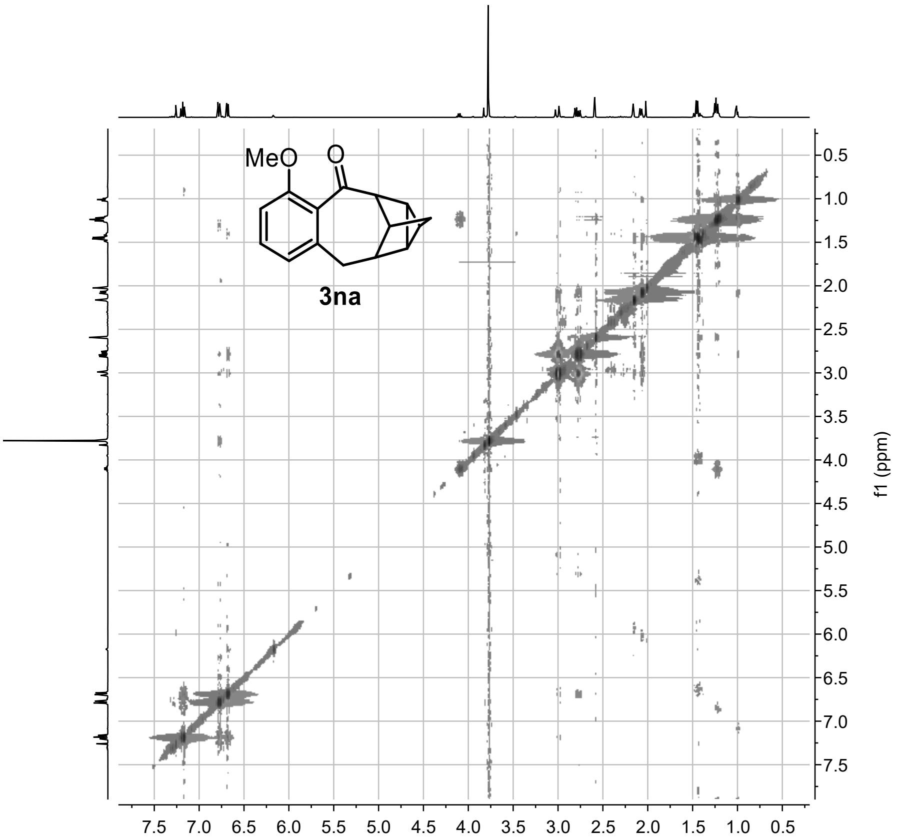

Figure S 187. $^{1}H,^{1}H$-NOESY of **3na** in $CDCl_3$ measured at 400.16 MHz.

***rel*-(1*R*,2*S*,3*R*,3a*R*,10*S*,10a*R*,11*S*)-1-(*tert*-butoxy)-5-methoxy-2,3,3a,9,10,10a-hexahydro-2,3,10-(epimethanetriyl)benzo[f]azulen-4(1H)-one**

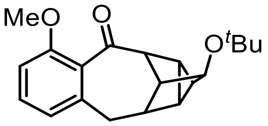

3nb

3nb was synthesized according to **GP-B** employing **1n** (148 mg, 1.00 mmol, 1.00 equiv.) and **2b** (214 mg, 1.30 mmol, 1.30 equiv.). Purification *via* flash chromatography (23 g SiO_2, gradient from 100:00 to 80:20 *n*-hexane/EA over 20 CV) afforded **3nb** as a mixture of isomers (240 mg, 663 µmol, 84%). Further purification by preparative HPLC (Kinetex C18; H_2O/MeOH + 0.1% FA = 32/68, for 10 min, then gradient to 20/80 over 25 min, flow rate = 14.8 mL/min, l = 220 nm) furnished: t_R = 13.5 min (**3nb** + diastereomer (1S)), 18.2 min (regioisomer), 27.1 min (*endo* [2+2] insertion product), and 31.2 min (*exo* [2+2] insertion product). A second preparative HPLC of the mixture of **3nb** and its diastereomer (1S) (Kinetex C18; H_2O/MeOH + 0.1% FA = 70:30 gradient to 0:100, over 30 min, flow rate = 7.5 mL/min, l = 220 nm) furnished: t_R = 31.1 min (**3nb**) and 31.5 min (diastereomer (1S)).

d.r.: 52:48

$C_{20}H_{24}O_3$ (312.41 $\frac{g}{mol}$)

R$_f$: 0.40 (*n*-hexane/EA = 80:20) [anisaldehyde]

^{1}H NMR(600.13 MHz, $CDCl_3$): δ = 7.18 (t, ^3J = 7.8 Hz, 1H, H-12,), 6.79 (d, ^3J = 8.4 Hz, 1H, H-13), 6.67 (d, ^3J = 7.6 Hz, 1H, H-11), 3.79 (s, 3H, H-16), 3.76 (m, 1H, H-5), 3.26 (m, 1H, H-2), 2.96 (d, ^2J = 15.7 Hz, 1H, H-9a), 2.75 (dd, ^2J = 15.8 Hz, ^3J = 7.7 Hz, 1H, H-9b), 2.16 (m, 1H, H-7), 2.08 (s, 1H, H-8), 1.45 (m, 1H, H-3), 1.19 (s, 9H, H-18), 1.19 (m, 1H, H-4), 1.13 (m, 1H, H-6).

^{13}C NMR(150.90 MHz, $CDCl_3$): δ = 209.6 (C-1), 155.8 (C-14), 136.6 (C-10), 130.7 (C-15), 130.2 (C-12), 122.1 (C-11), 109.9 (C-13), 77.9 (C-5), 73.7 (C-17), 56.1 (C-16), 53.7 (C-2), 47.8 (C-8), 37.9 (C-7), 35.9 (C-9), 28.6 (C-18), 18.8 (C-4), 17.7 (C-6), 16.0 (C-3).

HRMS (ESI-TOF) m/z: $[M+H]^+$ Calcd for $C_{20}H_{24}O_3H$ 313.1804; Found 313.1804.

IR (ATR, ṽ): 1686 cm^{-1} (s, *CO*), 1585 cm^{-1} (m).

Diastereomer (1*S*):

^{1}H NMR(600.13 MHz, $CDCl_3$): δ = 7.21 (t, ^3J = 7.8 Hz, 1H, H-12), 6.79 (d, ^3J = 8.4 Hz, 1H, H-13), 6.75 (d, ^3J = 7.5 Hz, 1H, H-11), 3.80 (s, 3H, H-16), 3.75 (m, 1H, H-5), 3.02 (d, ^2J = 15.7 Hz, 1H, H-9a), 2.90 (dd, ^2J = 15.7 Hz, ^3J = 7.7 Hz, 1H, H-9b), 2.76 (m, 1H, H-7), 2.66 (s, 1H, H-2), 2.08 (s, 1H, H-8), 1.35 (m, 1H, H-3), 1.25 (m, 1H, H-6), 1.20 (s, 9H, H-18), 1.19 (m, 1H, H-4).

^{13}C NMR(150.90 MHz, $CDCl_3$): δ = 207.8 (C-1), 155.9 (C-14), 137.6 (C-10), 130.6 (C-12), 129.5 (C-15), 122.3 (C-11), 109.7 (C-13), 78.2 (C-5), 74.8 (C-17), 56.1 (C-16), 54.0 (C-2), 47.2 (C-8), 37.6 (C-7), 35.2 (C-9), 28.7 (C-18), 18.2 (C-4), 17.1 (C-3), 16.3 (C-6).

HRMS (ESI-TOF) m/z: $[M+H]^+$ Calcd for $C_{20}H_{24}O_3H$ 313.1804; Found 313.1804.

IR (ATR, ṽ): 1683 cm^{-1} (s, *CO*), 1584 cm^{-1} (m).

regioisomer:

regioisomeric ratio: 82:18

mp: 137.8 °C.

^{1}H NMR(600.13 MHz, $CDCl_3$): δ = 7.11 (t, ^3J = 8.0 Hz, 1H, H-12), 6.78 (d, ^3J = 8.1 Hz, 1H, H-13), 6.66 (d, ^3J = 7.6 Hz, 1H, H-11), 4.05 (d, ^2J = 12.3 Hz, 1H, H-15b), 3.98 (m, 1H, H-5), 3.83 (s, 3H, H-16), 3.73 (d, ^2J = 12.3 Hz, 1H, H-15a), 3.69 (s, 1H, H-2), 3.25 (s, 1H, H-7), 2.26 (s, 1H, H-8), 1.75 (m, 1H, H-3), 1.55 (m, 1H, H-6), 1.38 (m, 1H, H-4), 1.22 (s, 9H, H-18).

^{13}C NMR(150.90 MHz, $CDCl_3$): δ = 209.9 (C-1), 157.8 (C-14), 133.3 (C-10), 127.9 (C-12), 127.4 (C-15), 123.0 (C-13), 109.4 (C-11), 77.9 (C-5), 73.9 (C-17), 55.9 (C-16), 55.0 (C-2), 49.9 (C-15), 44.8 (C-8), 38.8 (C-7), 28.7 (C-18), 19.0 (C-4), 18.9 (C-6), 17.3 (C-3).

HRMS (ESI-TOF) m/z: $[M+H]^+$ Calcd for $C_{20}H_{24}O_3H$ 313.1804; Found 313.1801.

IR (ATR, ṽ): 1702 cm^{-1} (s, *CO*), 1582 cm^{-1} (m).

***endo* [2+2] insertion product:**

d.r.: 95:5 (*endo*:*exo*)

mp: 145.1 °C.

^{1}H NMR(600.13 MHz, $CDCl_3$): δ = 7.28 (t, ^3J = 7.9 Hz, 1H, H-12), 6.76 (d, ^3J = 8.4 Hz, 1H, H-13), 6.70 (d, ^3J = 7.5 Hz, 1H, H-11), 6.24 (dd, ^3J = 6.2 Hz, ^3J = 3.3 Hz, 1H, H-5), 5.90 (dd, ^3J = 6.3 Hz, ^3J = 3.5 Hz, 1H, H-6), 3.83 (s, 3H, H-16), 3.54 (m, 1H, H-4), 3.36 (dd,

^3J = 10.8 Hz, ^3J = 3.8 Hz, 1H, H-2), 3.23 (dddd, ^3J = 11.3 Hz, ^3J = 10.8 Hz, 1H, ^3J = 6.9 Hz, ^3J = 3.8 Hz, 1H, H-8), 2.99 (m, 1H, H-3), 2.79 (dd, ^2J = 15.0 Hz, ^3J = 6.9 Hz, 1H, H-9b), 2.67 (m, 1H, H-7), 2.45 (dd, ^2J = 15.0 Hz, ^3J = 11.3 Hz, 1H, H-9a), 1.15 (s, 9H, H-18).

^{13}C NMR(150.90 MHz, $CDCl_3$): δ = 202.2 (C-1), 157.1 (C-14), 145.5 (C-10), 137.0 (C-5), 133.4 (C-6), 133.0 (C-12), 125.4 (C-15), 120.1 (C-11), 109.7 (C-13), 84.0 (C-4), 73.6 (C-17), 55.9 (C-16), 52.8 (C-2), 50.7 (C-7), 48.5 (C-3), 36.7 (C-8), 31.6 (C-9), 28.4 (C-18).

HRMS (ESI-TOF) m/z: $[M+H]^+$ Calcd for $C_{20}H_{24}O_3H$ 313.1804; Found 313.1797.

IR (ATR, ṽ): 1694 cm^{-1} (s, *CO*), 1587 cm^{-1} (m).

***exo* [2+2] insertion product:**

^{1}H NMR(600.13 MHz, $CDCl_3$): δ = 7.32 (t, ^3J = 8.1 Hz, 1H, H-12), 6.79 (d, ^3J = 8.4 Hz, 1H, H-11), 6.77 (d, ^3J = 7.5 Hz, 1H, H-13), 6.09 (ddd, ^3J = 6.2 Hz, ^3J = 3.3 Hz, ^4J = 0.9 Hz, 1H, H-5), 6.08 (ddd, ^3J = 6.2 Hz, ^3J = 3.4 Hz, ^4J = 0.9 Hz, 1H, H-6), 3.86 (s, 3H, H-16), 3.67 (dd, ^2J = 15.3 Hz, ^3J = 11.3 Hz, 1H, H-9a), 3.55 (m, 1H, H-4), 3.46 (m, 1H, H-7), 2.73 (dd, ^2J = 15.0 Hz, ^3J = 7.5 Hz, 1H, H-9b), 2.48 (m, 1H, H-3), 2.34 (dm, ^3J = 10.7 Hz, 1H, H-2), 2.28 (dddm, ^3J = 11.3 Hz, ^3J = 10.3 Hz, ^3J = 7.5 Hz, 1H, H-8), 1.07 (s, 9H, H-18).

^{13}C NMR(150.90 MHz, $CDCl_3$): δ = 198.9 (C-1), 157.9 (C-14), 145.6 (C-10), 135.5 (C-5), 134.4 (C-6), 132.9 (C-12), 125.4 (C-15), 120.3 (C-13), 109.3 (C-11), 84.3 (C-4), 74.2 (C-17), 56.1 (C-16), 51.0 (C-2), 50.2 (C-3), 47.2 (C-7), 37.6 (C-8), 30.7 (C-9), 28.0 (C-18).

HRMS (ESI-TOF) m/z: $[M+H]^+$ Calcd for $C_{20}H_{24}O_3H$ 313.1804; Found 313.1801.

IR (ATR, ṽ): 1732 cm^{-1} (m, *CO*), 1681 cm^{-1} (m), 1664 cm^{-1} (m), 1590 cm^{-1} (m).

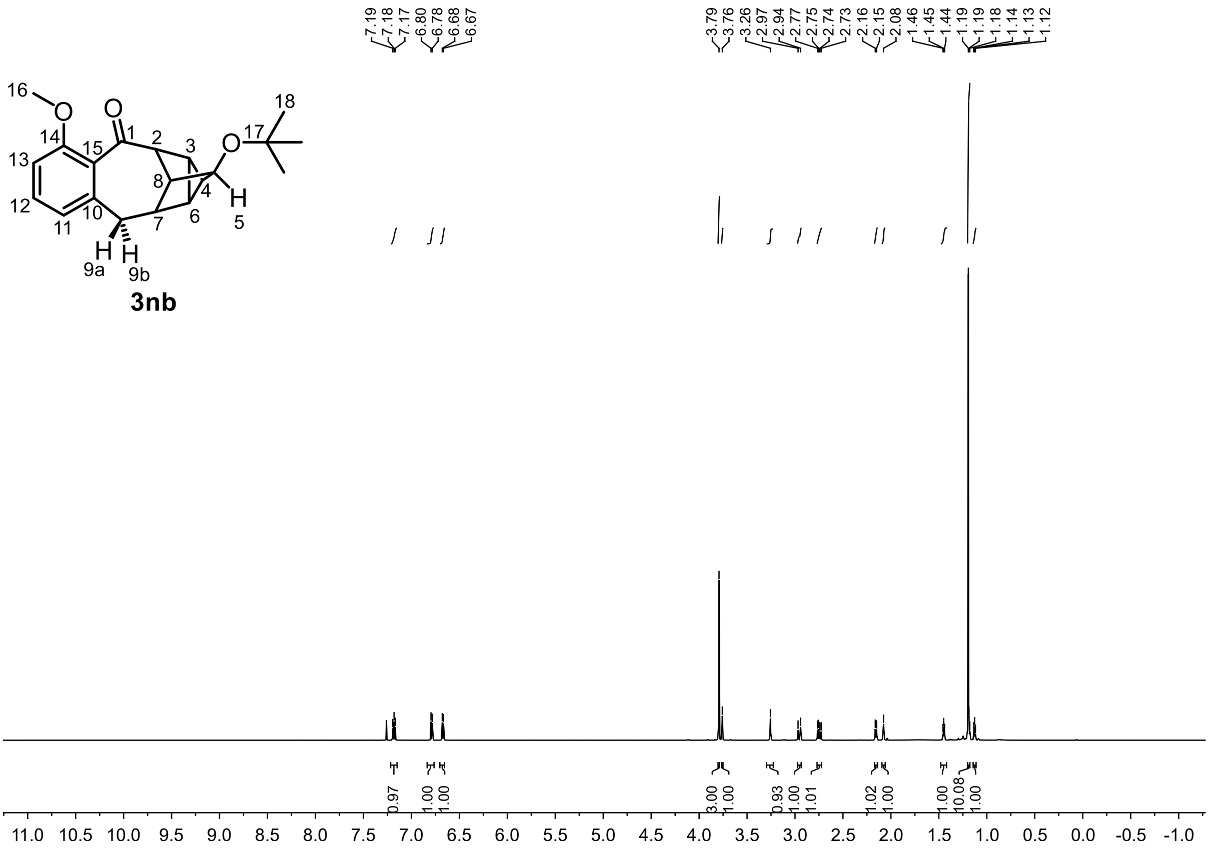

Figure S 188. ^{1}H NMR of **3nb** in $CDCl_3$ measured at 600.13 MHz.

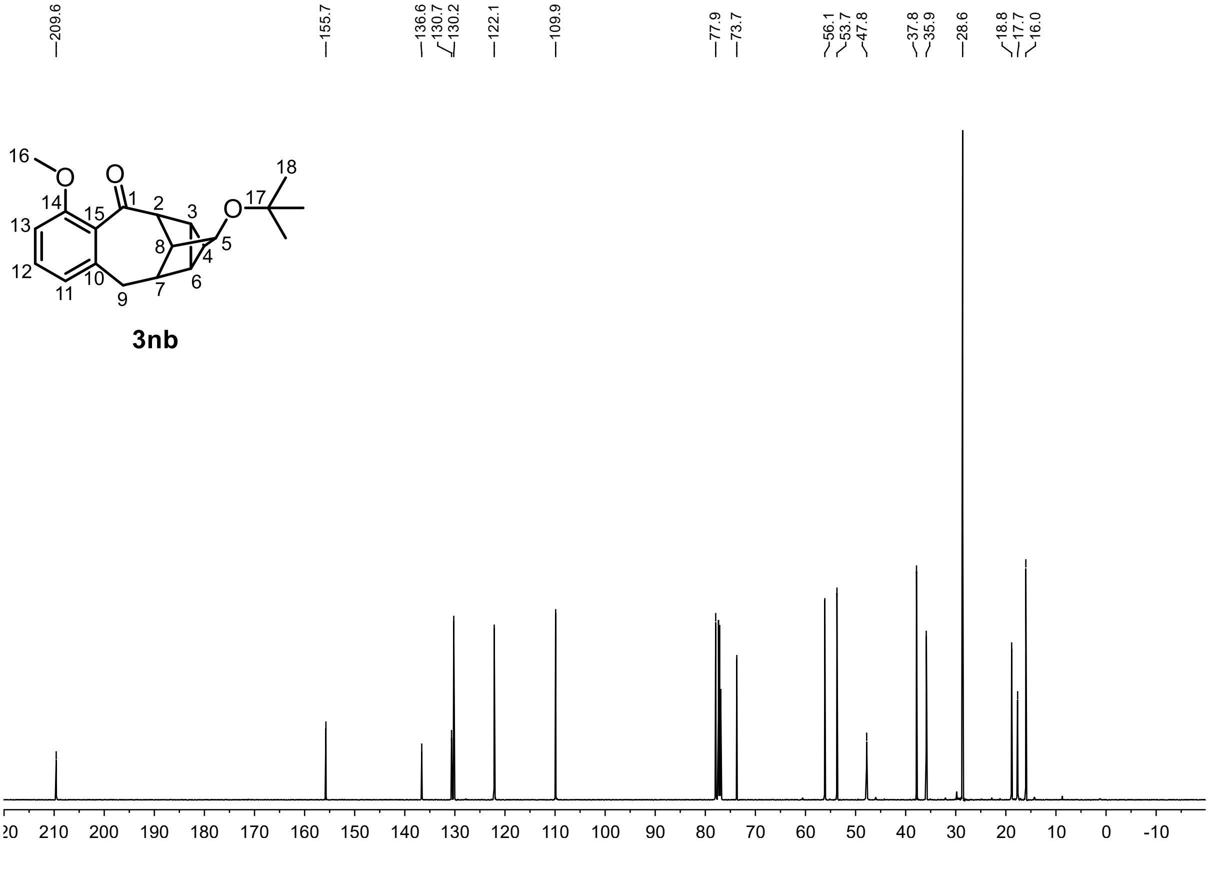

Figure S 189. ^{13}C NMR of **3nb** in $CDCl_3$ measured at 150.92 MHz.

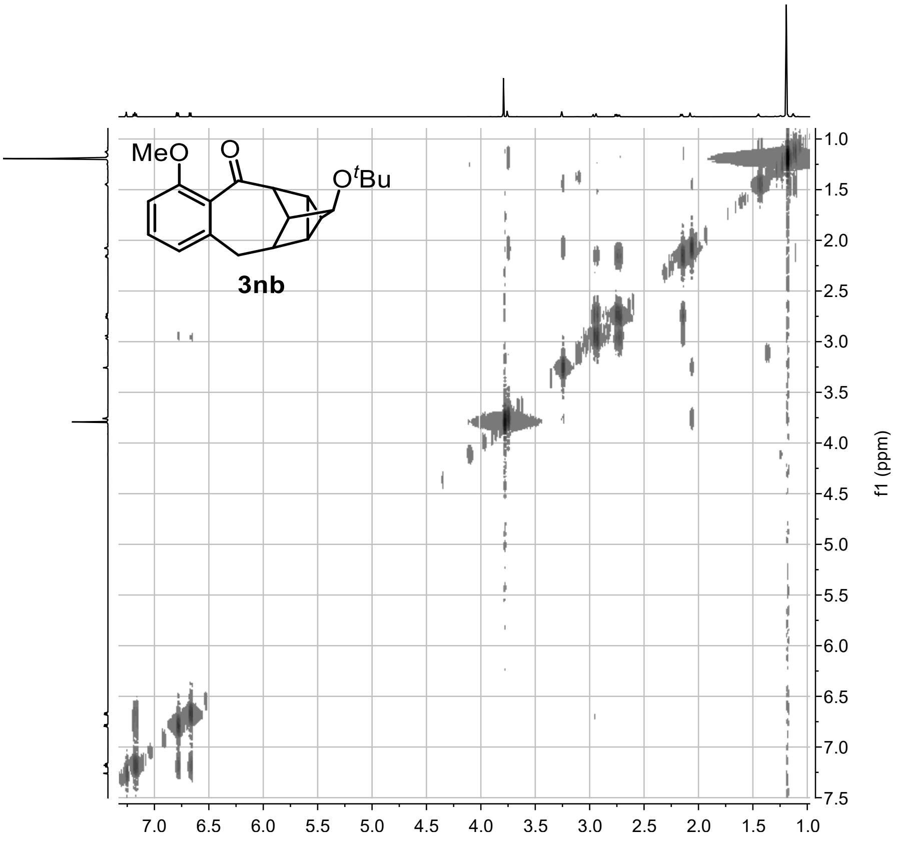

Figure S 190. $^1H,^1H$-COSY of **3nb** in $CDCl_3$ measured at 600.13 MHz.

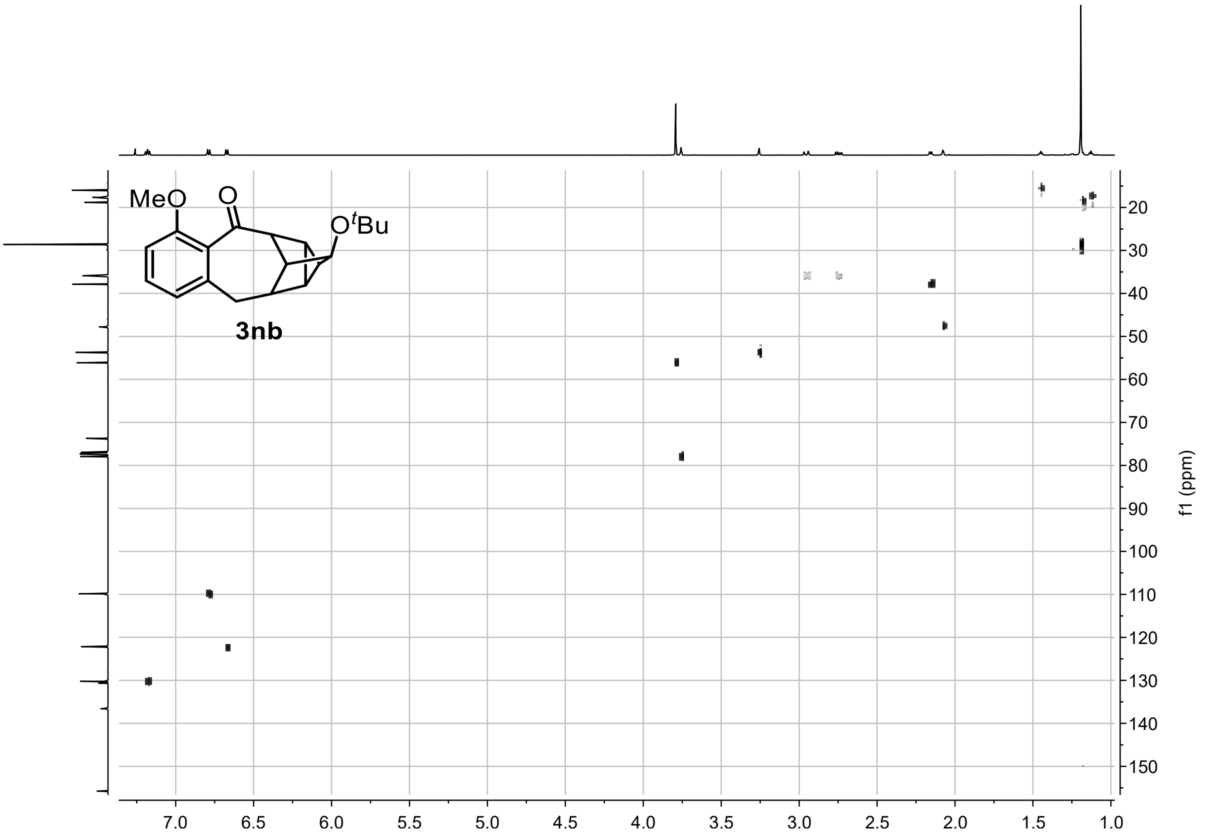

Figure S 191. $^1H,^{13}C$-HSQC of **3nb** in $CDCl_3$ measured at ^1H: 600.13 MHz; ^{13}C: 150.92 MHz.

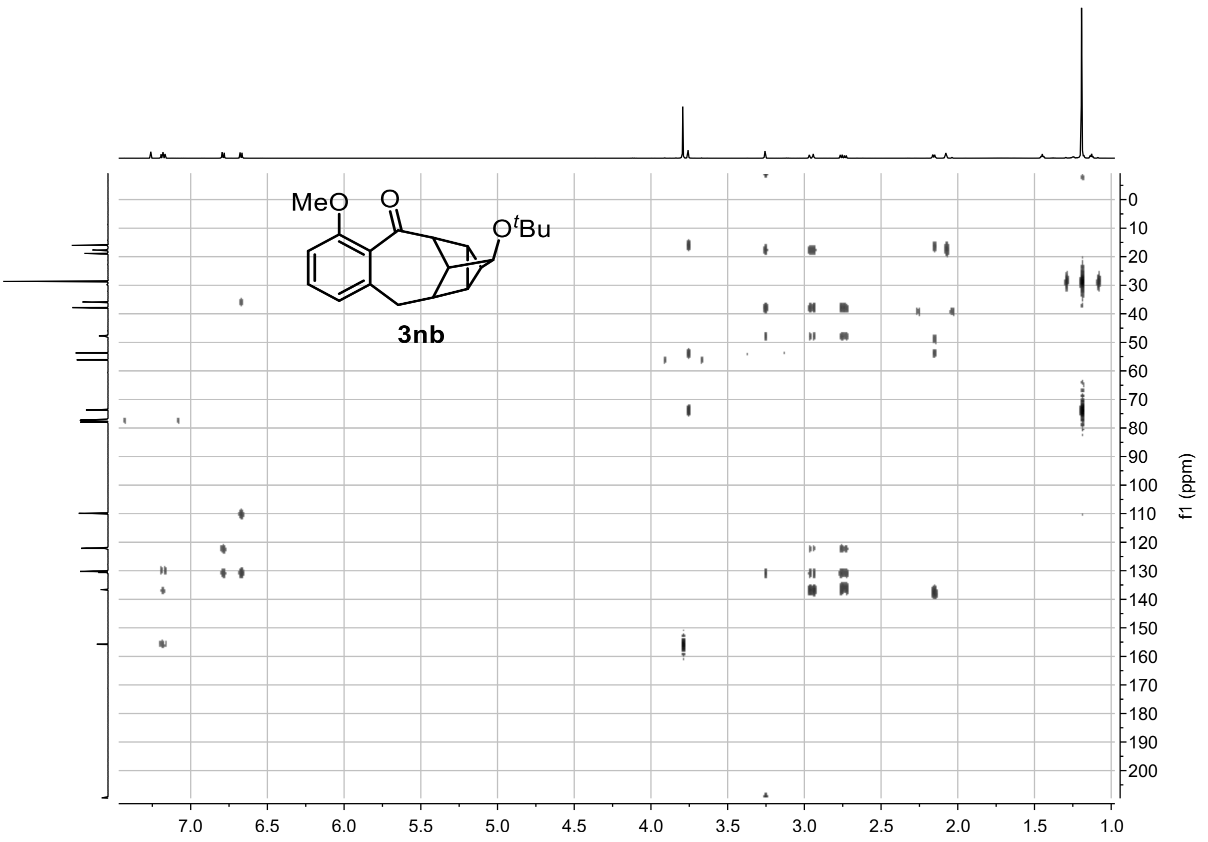

Figure S 192. ^{1}H,^{13}C-HMBC of **3nb** in $CDCl_3$ measured at ^{1}H: 600.13 MHz; ^{13}C: 150.92 MHz.

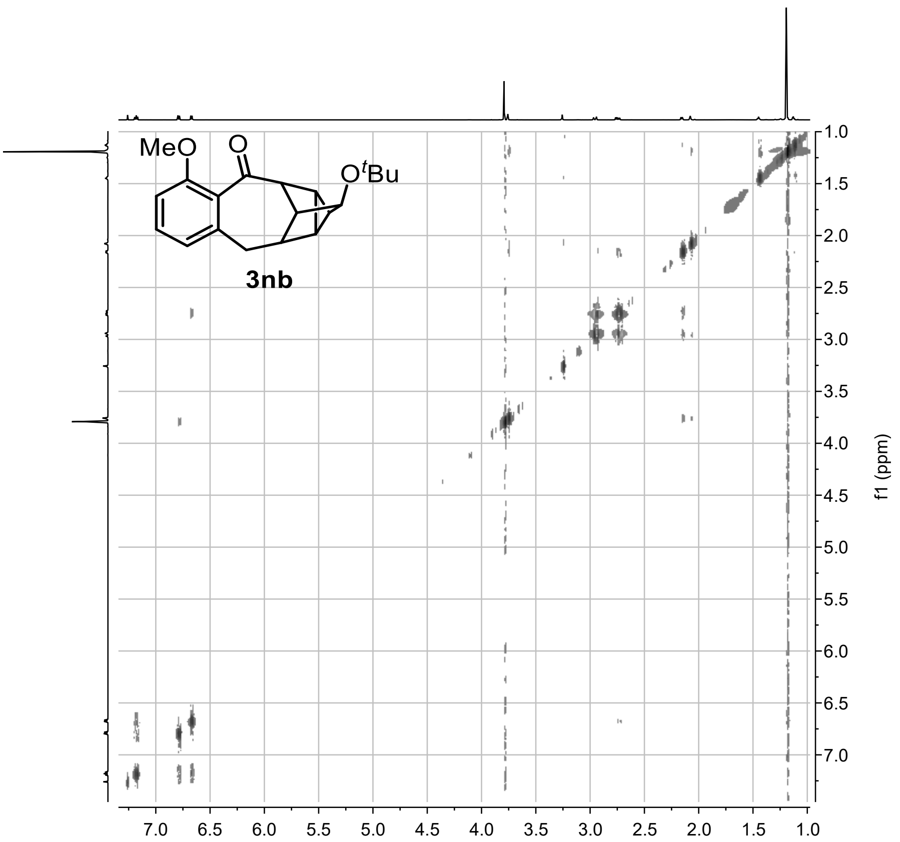

Figure S 193. ^{1}H,^{1}H-NOESY of **3nb** in $CDCl_3$ measured at 600.13 MHz.

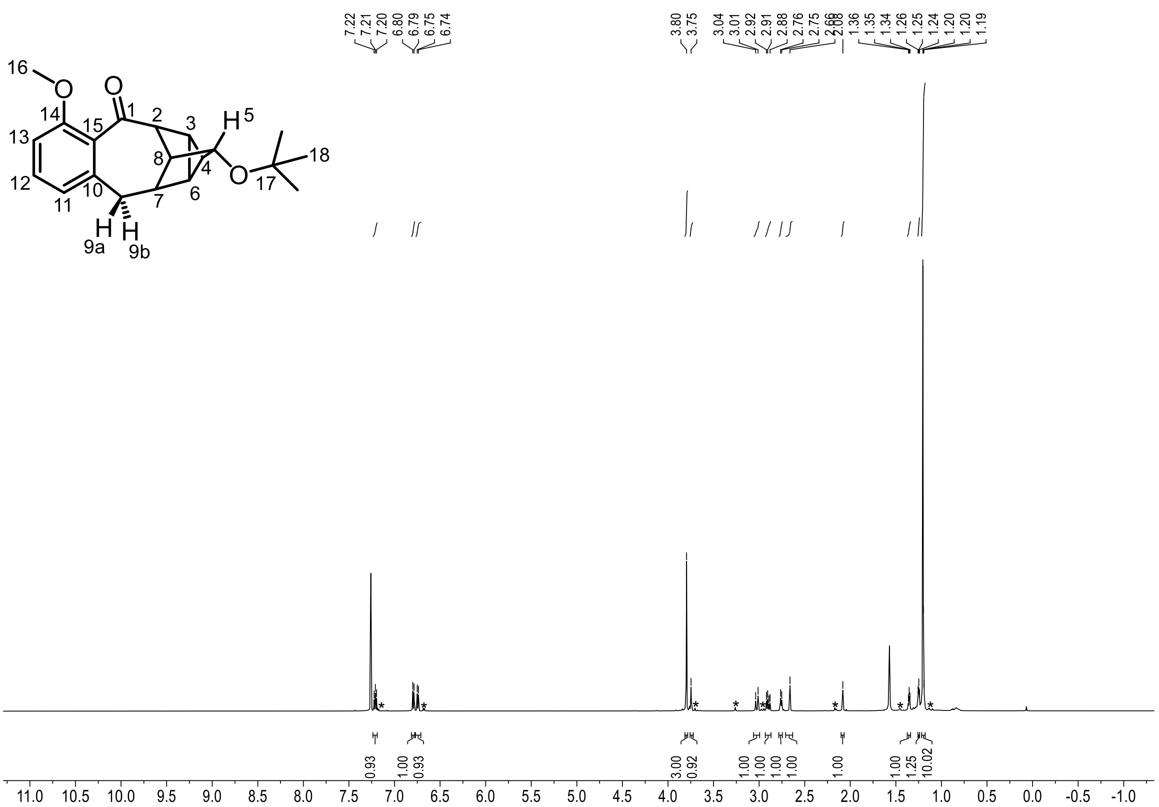

Figure S 194. ^{1}H NMR of the diastereomer (1*S*) of **3nb** in $CDCl_3$ measured at 600.13 MHz. * denotes signals of the *syn* diastereomer.

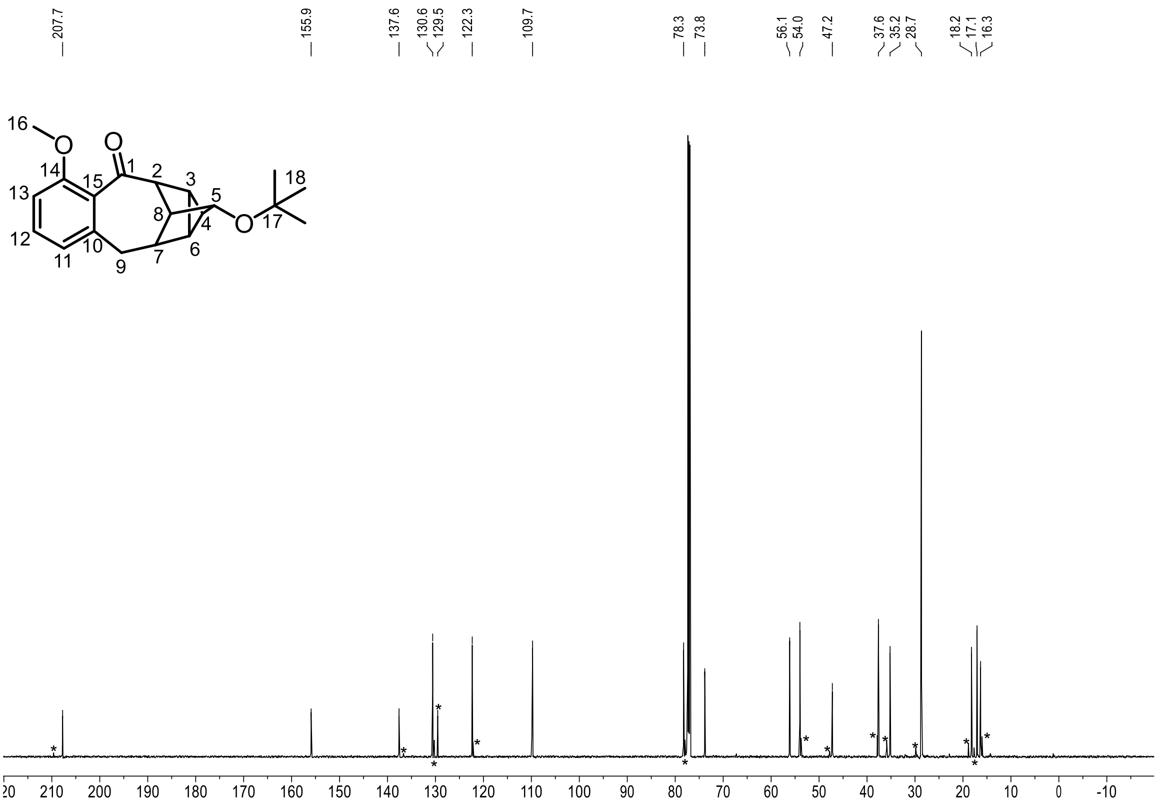

Figure S 195. ^{13}C NMR of the diastereomer (1*S*) of **3nb** in $CDCl_3$ measured at 150.92 MHz. * denotes signals of the *syn* diastereomer.

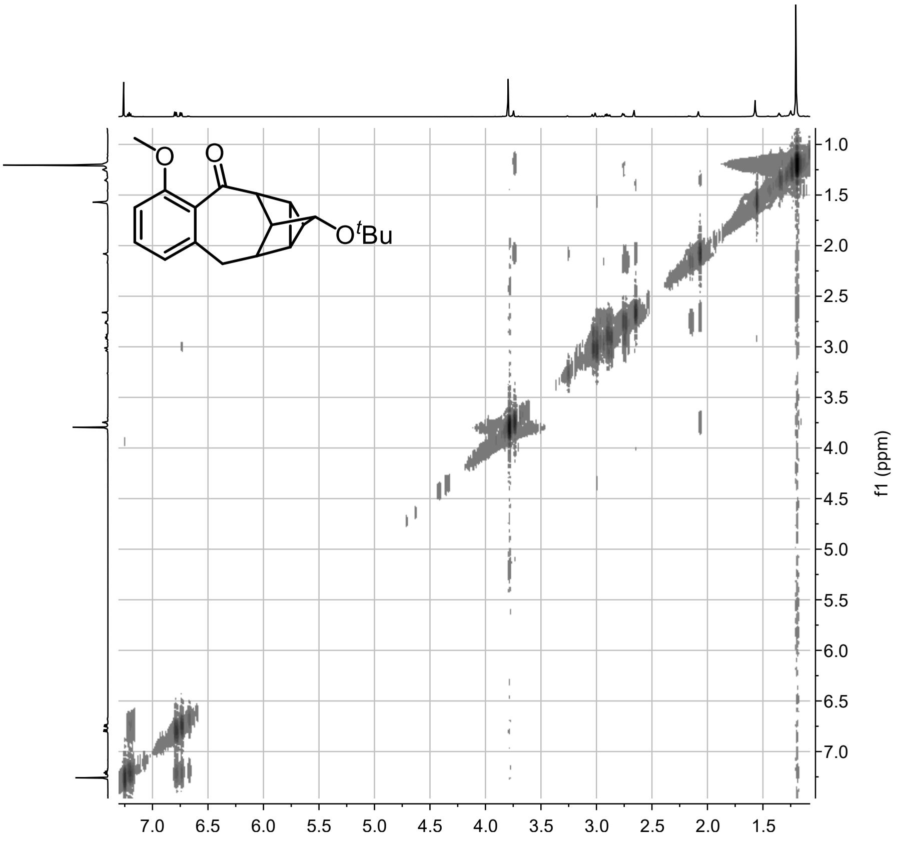

Figure S 196. $^{1}H,^{1}H$-COSY of the diastereomer (1*S*) of **3nb** in $CDCl_3$ measured at 600.13 MHz

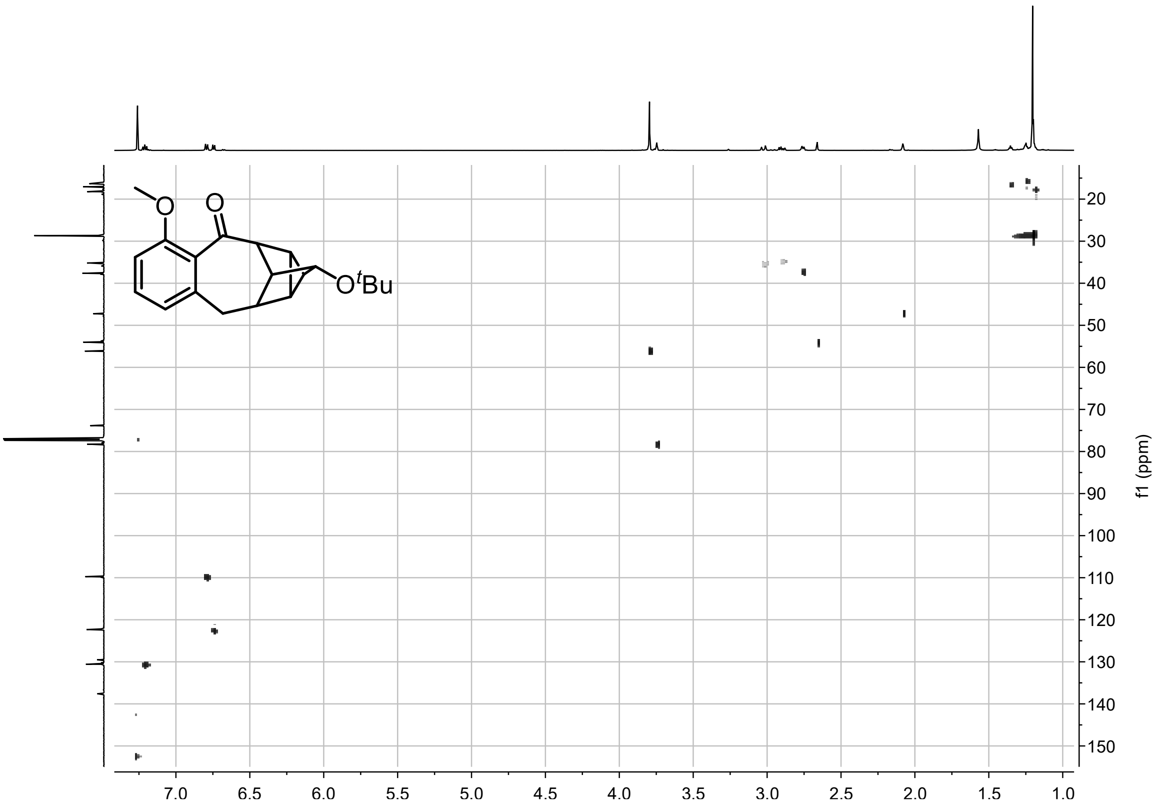

Figure S 197. $^{1}H,^{13}C$-HSQC of the diastereomer (1*S*) of **3nb** in $CDCl_3$ measured at ^{1}H: 600.13 MHz; ^{13}C: 150.92 MHz.

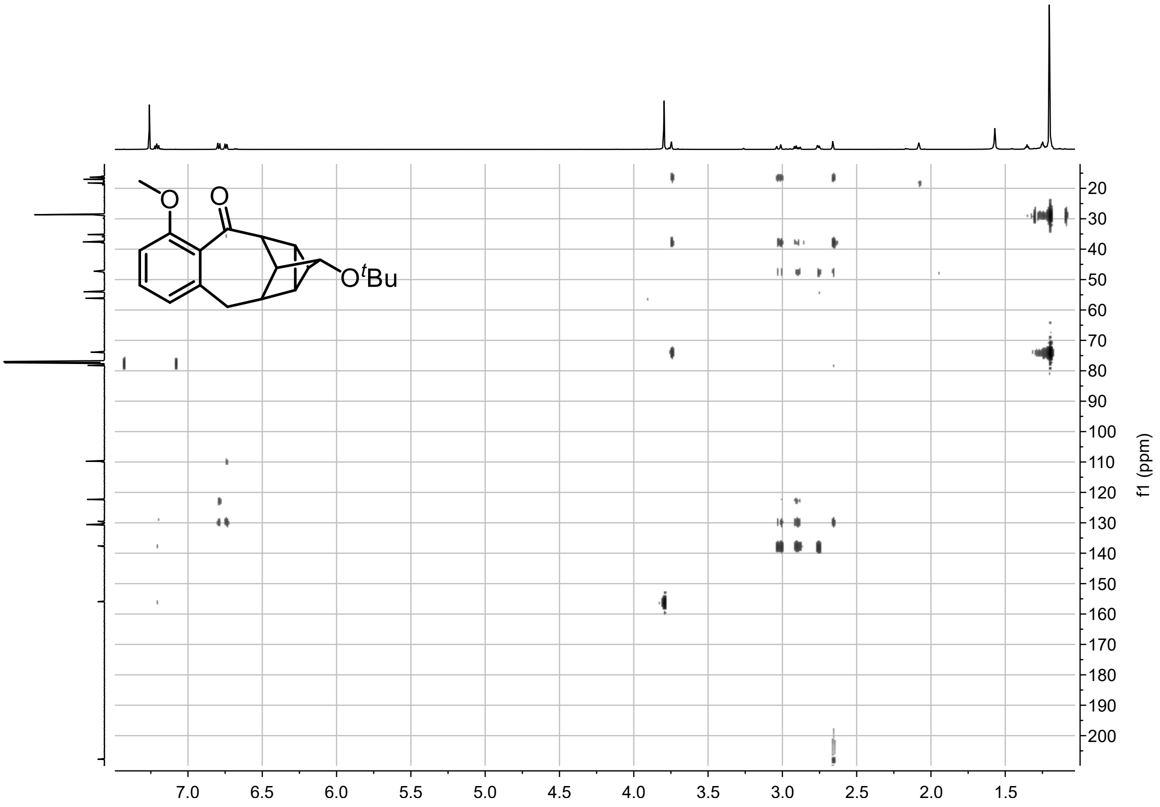

Figure S 198. ^{1}H,^{13}C-HMBC of the diastereomer (1*S*) of **3nb** in $CDCl_3$ measured at ^{1}H: 600.13 MHz; ^{13}C: 150.92 MHz.

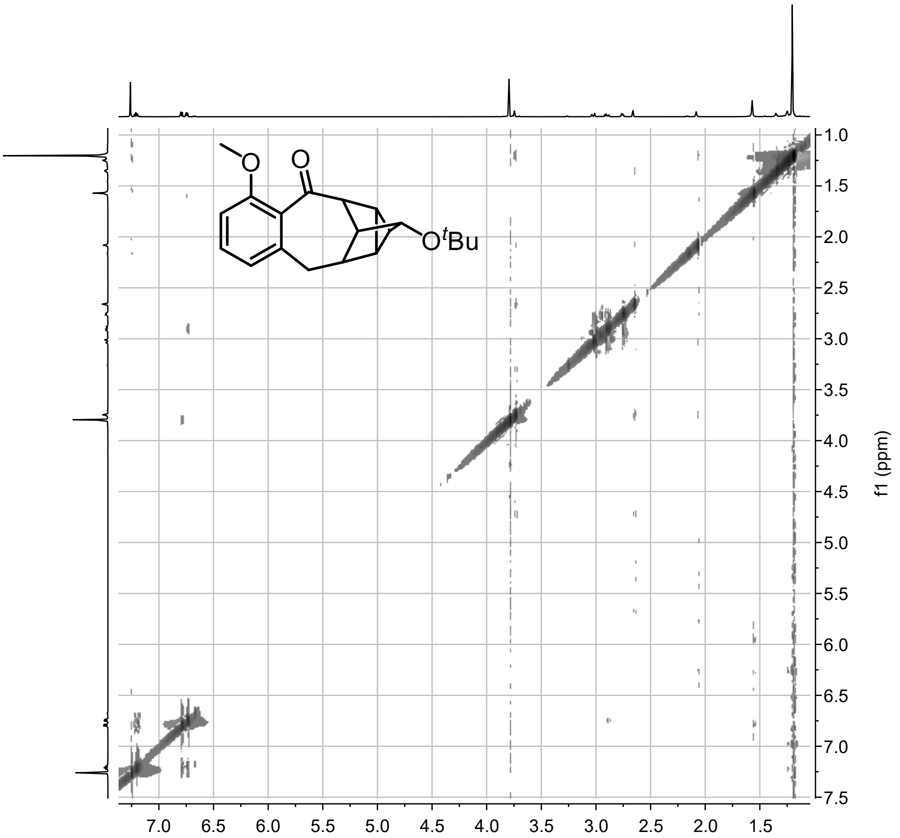

Figure S 199. ^{1}H,^{1}H-NOESY of the diastereomer (1*S*) of **3nb** in $CDCl_3$ measured at 600.13 MHz.

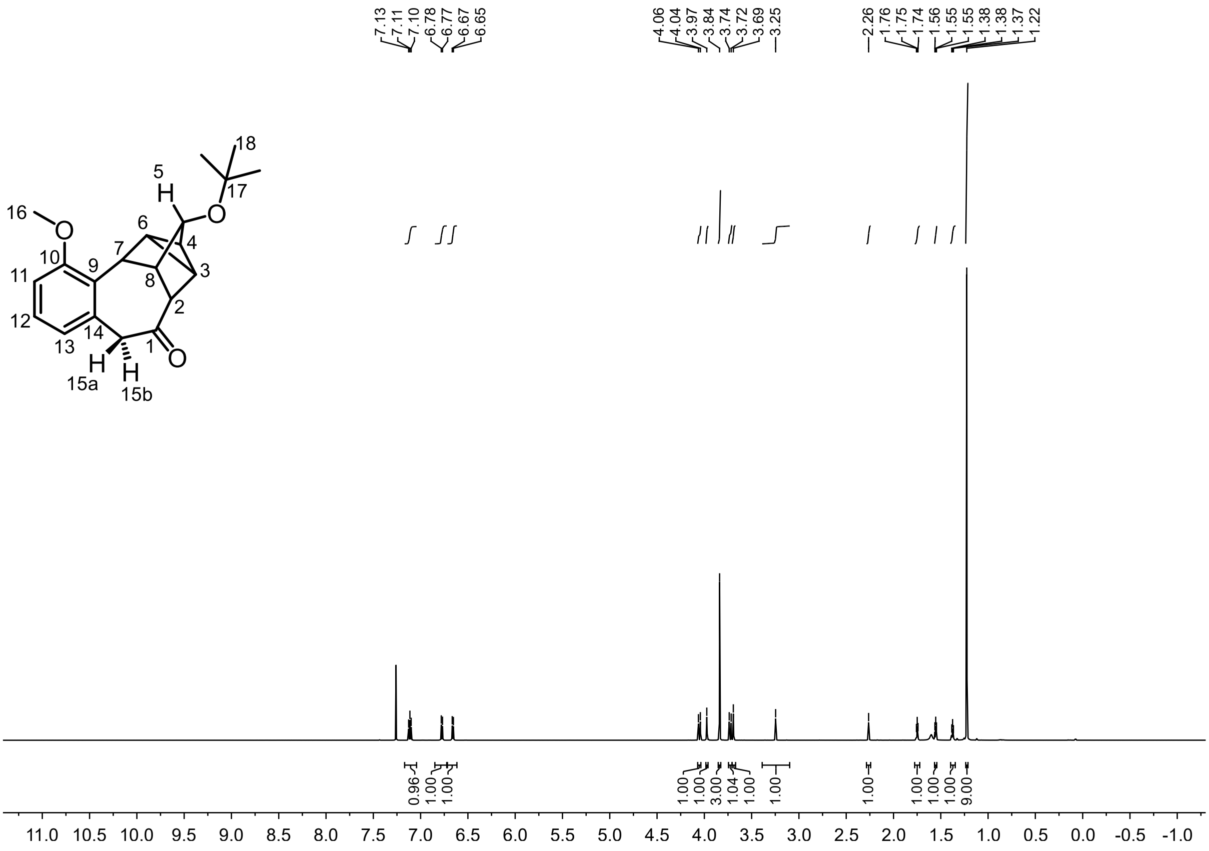

Figure S 200. ^{1}H NMR of the regioisomer of **3nb** in $CDCl_3$ measured at 600.13 MHz.

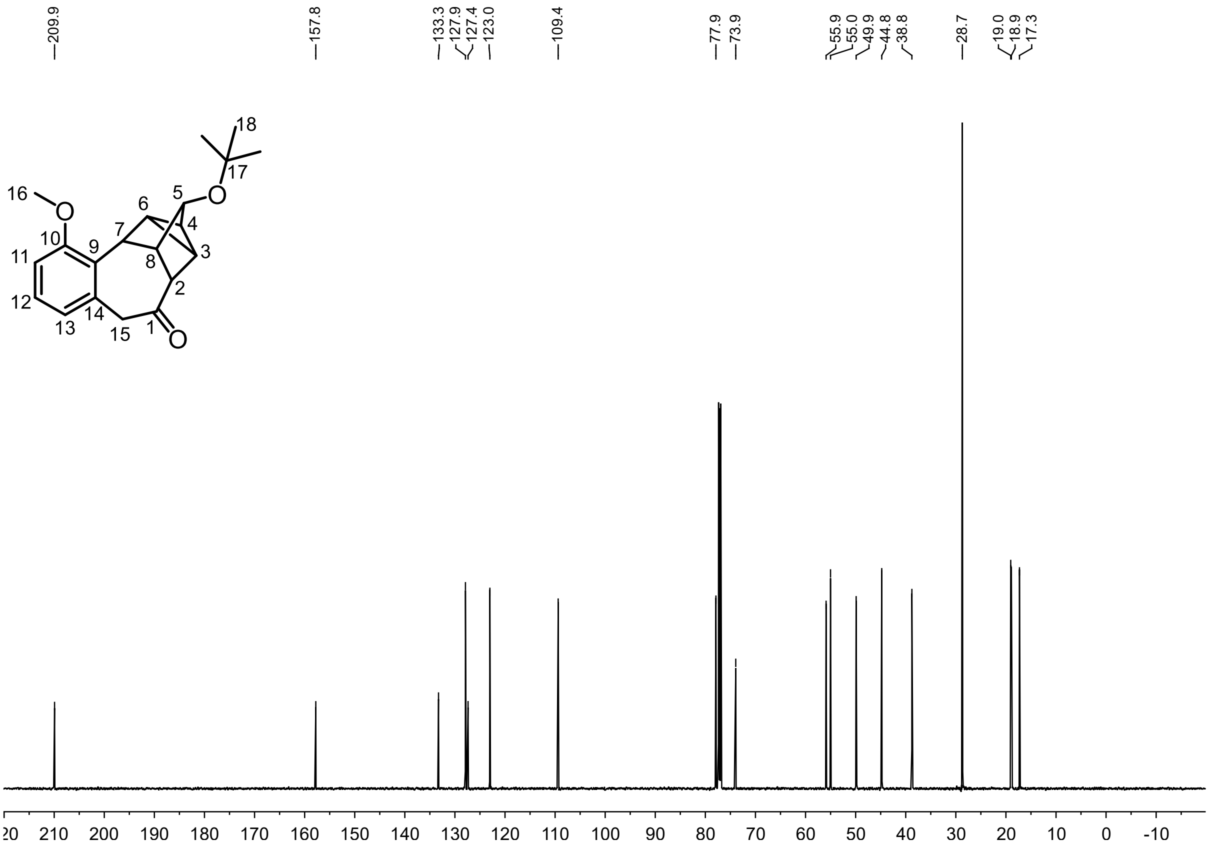

Figure S 201. ^{13}C NMR of the regioisomer of **3nb** in $CDCl_3$ measured at 150.92 MHz.

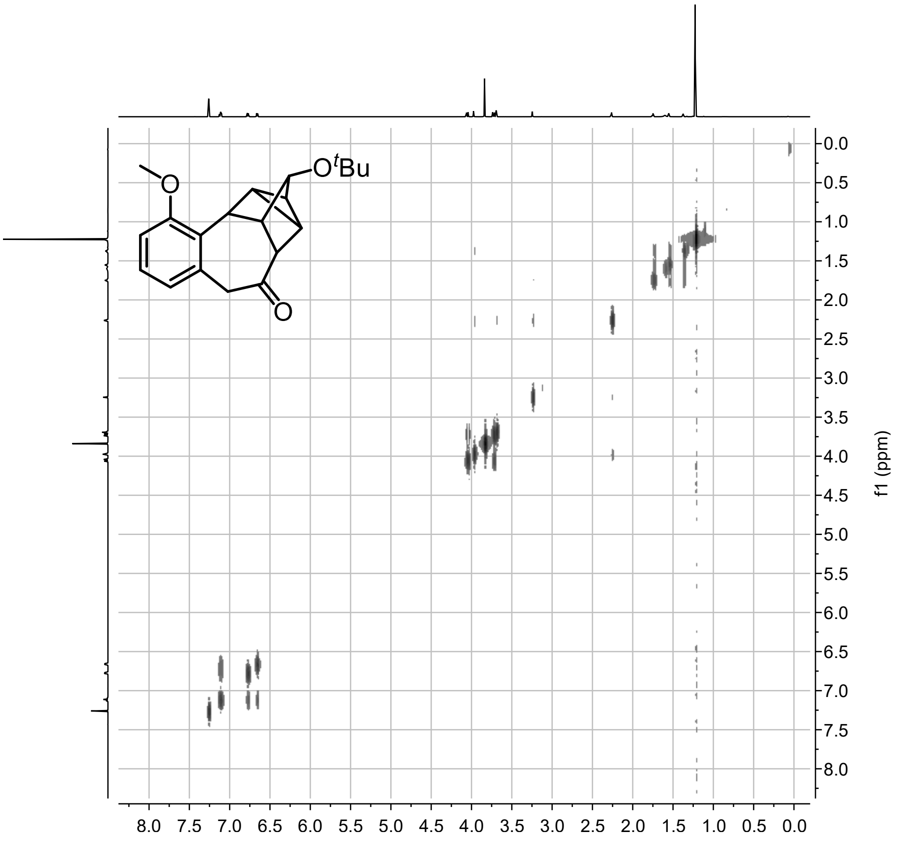

Figure S 202. ^{1}H,^{1}H-COSY of the regioisomer of **3nb** in $CDCl_3$ measured at 600.13 MHz.

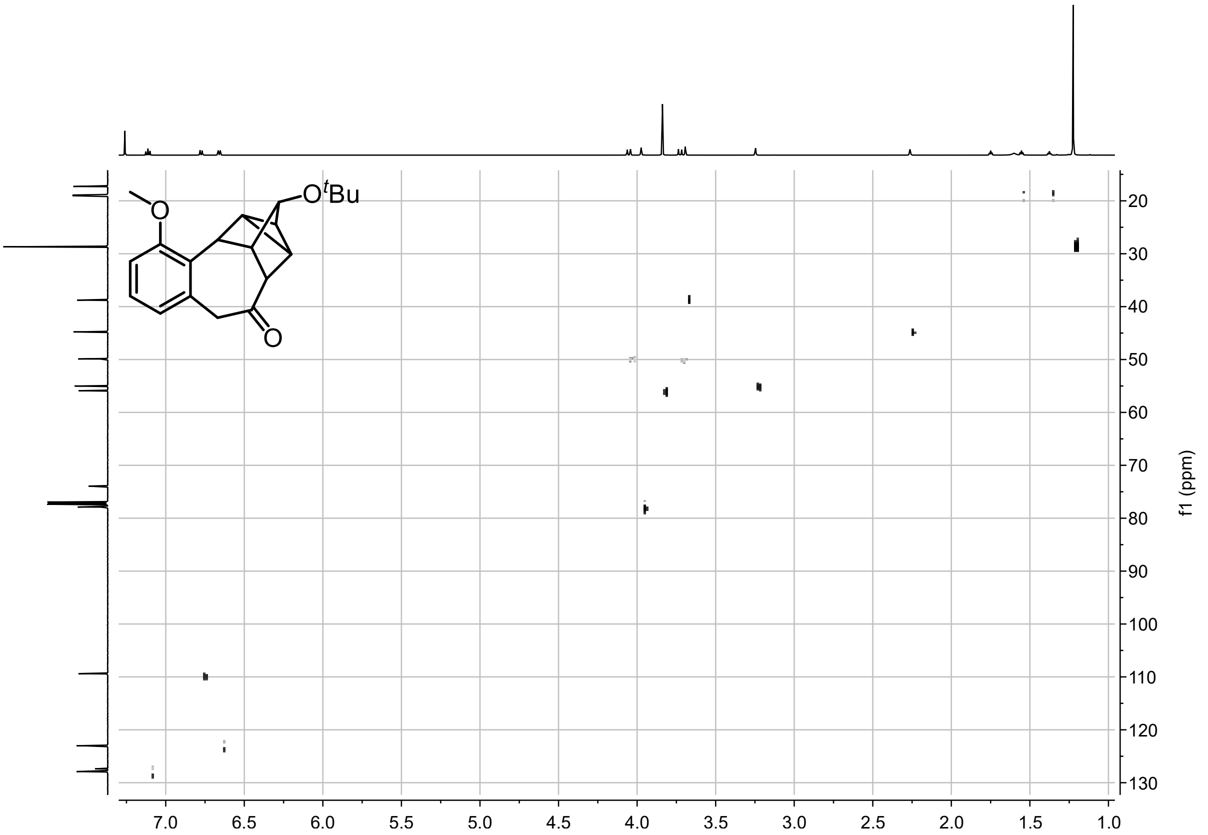

Figure S 203. ^{1}H,^{13}C-HSQC of the regioisomer of **3nb** in $CDCl_3$ measured at ^{1}H: 600.13 MHz; ^{13}C: 150.92 MHz.

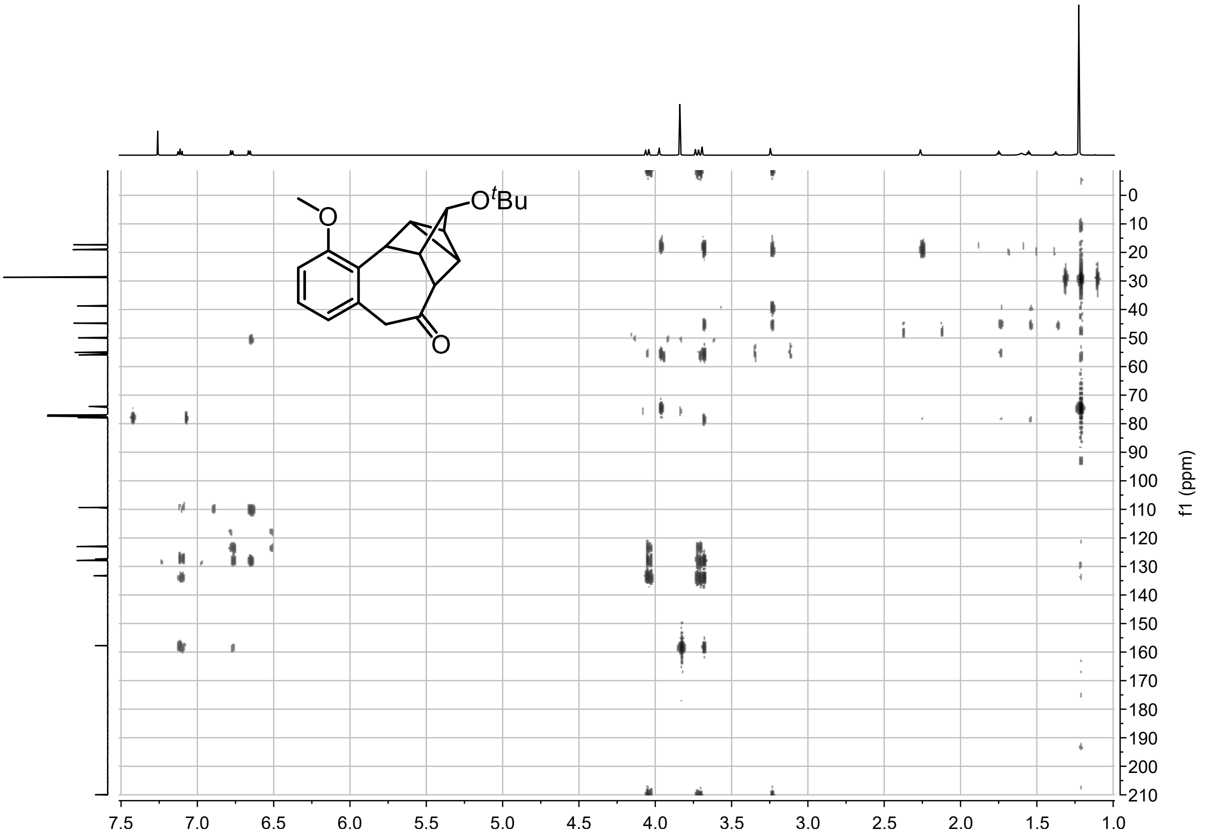

Figure S 204. $^{1}H,^{13}C$-HMBC of the regioisomer of **3nb** in $CDCl_3$ measured at ^{1}H: 600.13 MHz; ^{13}C: 150.92 MHz.

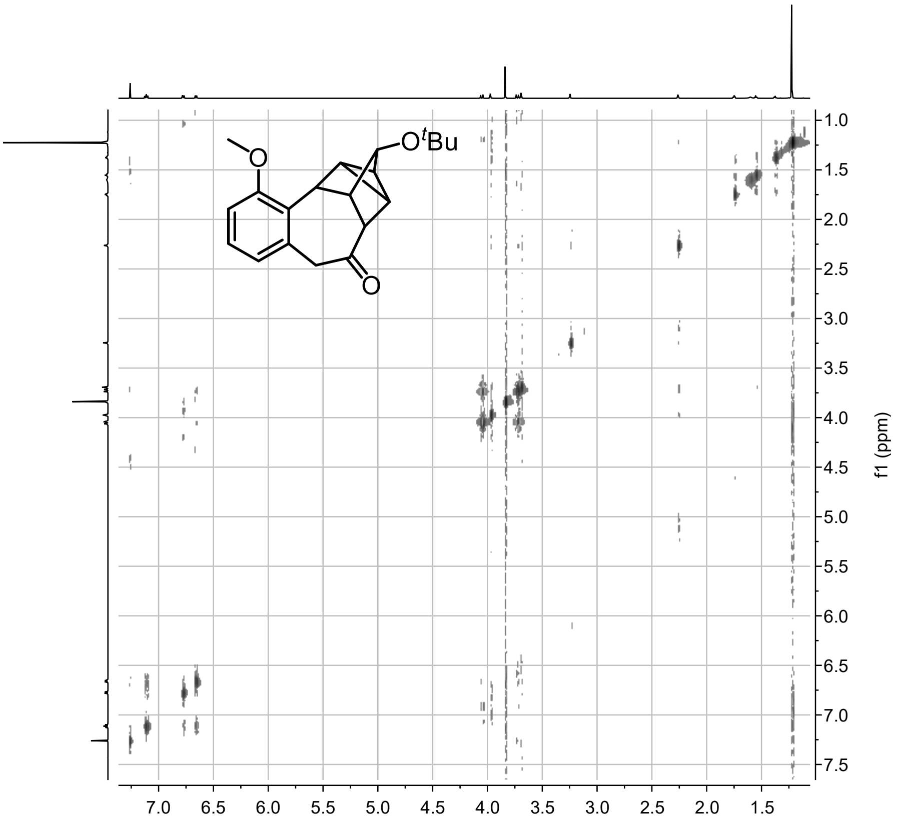

Figure S 205. $^{1}H,^{1}H$-NOESY of the regioisomer of **3nb** in $CDCl_3$ measured at 600.13 MHz.

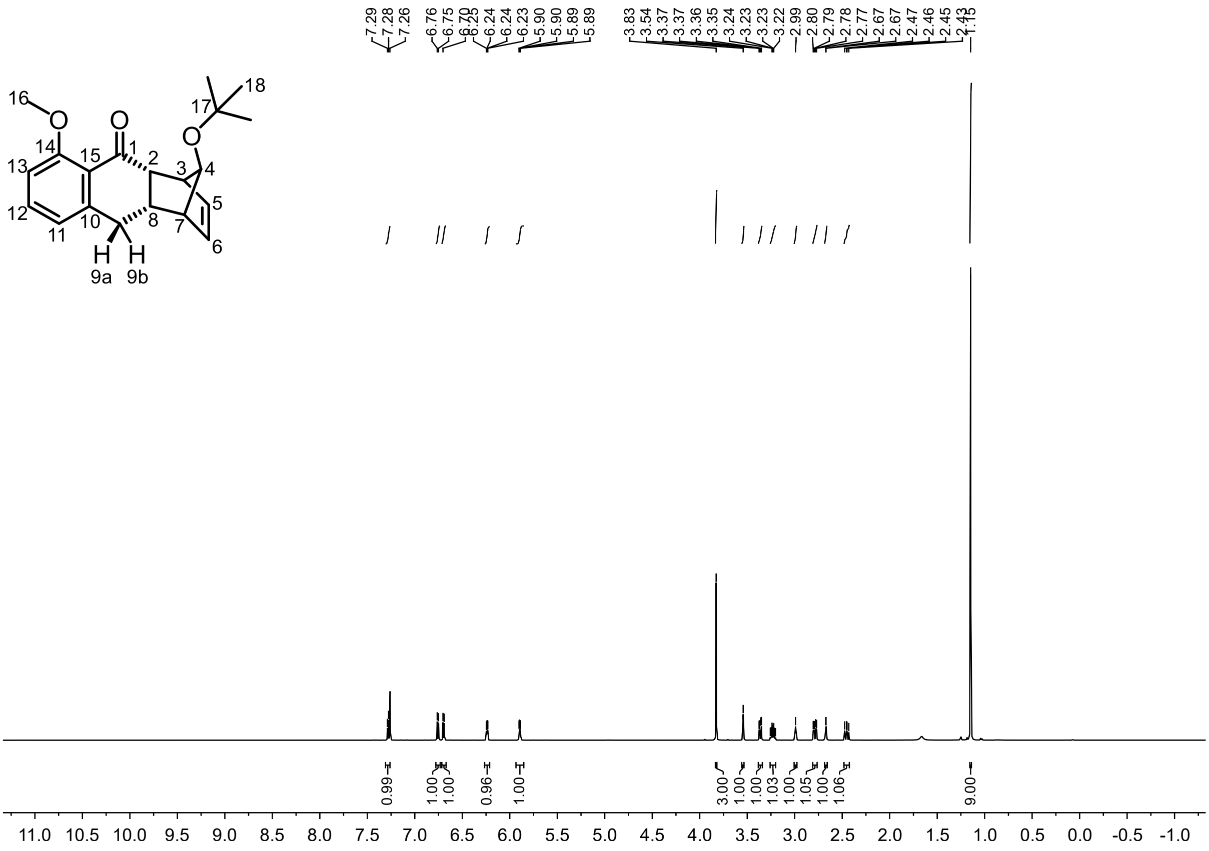

Figure S 206. ^{1}H NMR of the *endo* [2+2] insertion product of **3nb** in $CDCl_3$ measured at 600.13 MHz.

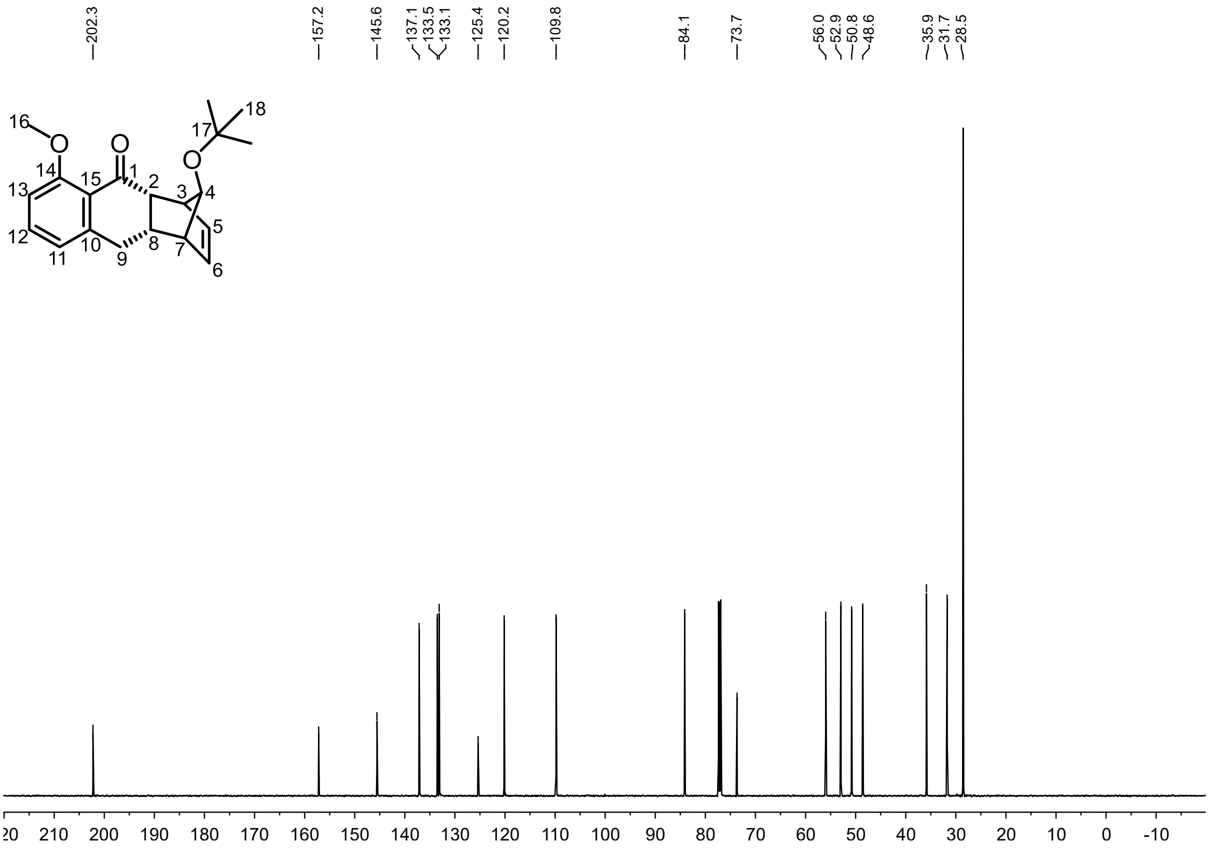

Figure S 207. ^{13}C NMR of the *endo* [2+2] insertion product of **3nb** in $CDCl_3$ measured at 150.92 MHz.

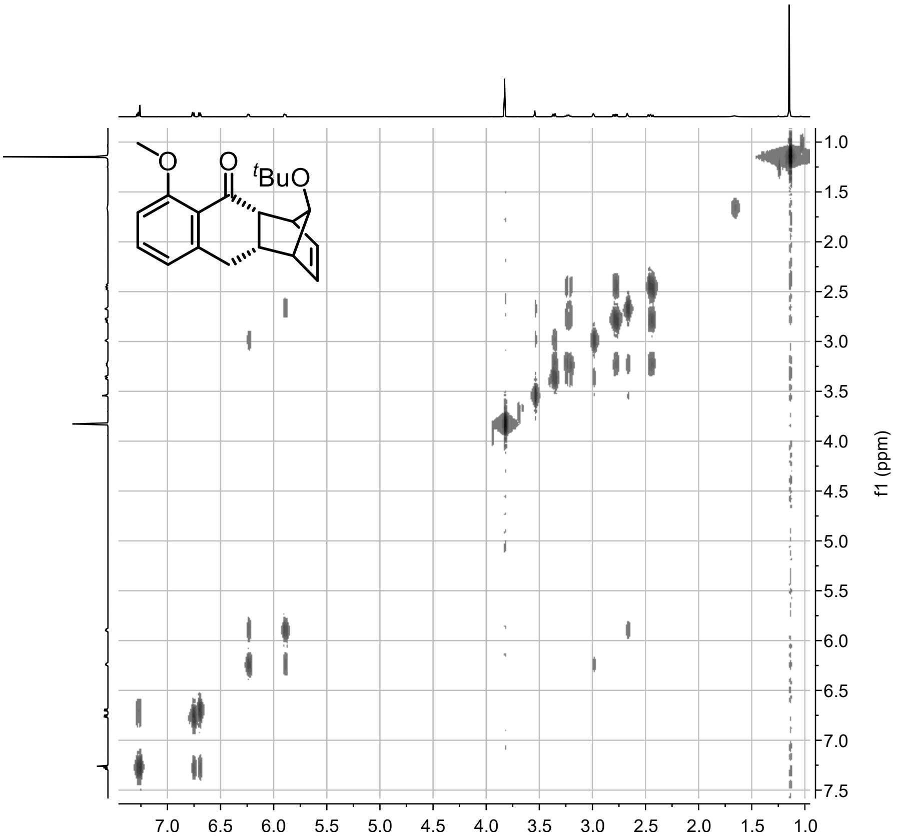

Figure S 208. ^{1}H,^{1}H-COSY of the *endo* [2+2] insertion product of **3nb** in $CDCl_3$ measured at 600.13 MHz.

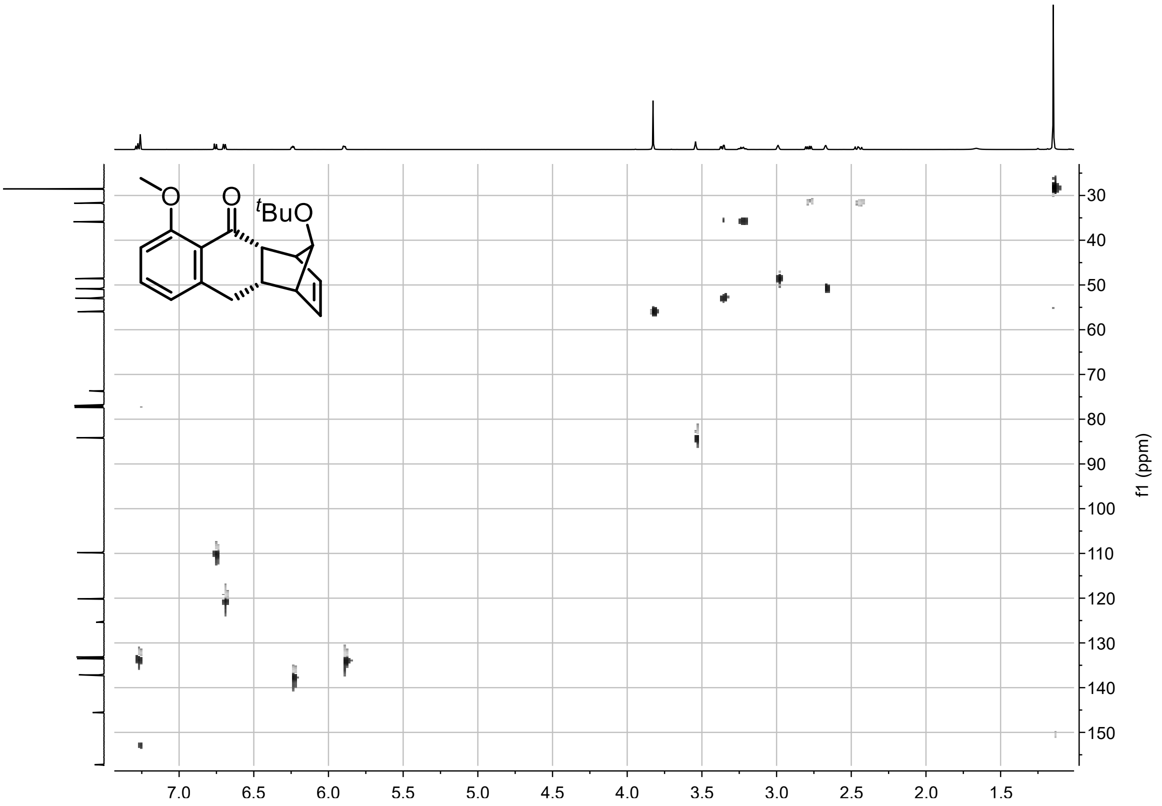

Figure S 209. ^{1}H,^{13}C-HSQC of the *endo* [2+2] insertion product of **3nb** in $CDCl_3$ measured at ^{1}H: 600.13 MHz; ^{13}C: 150.92 MHz.

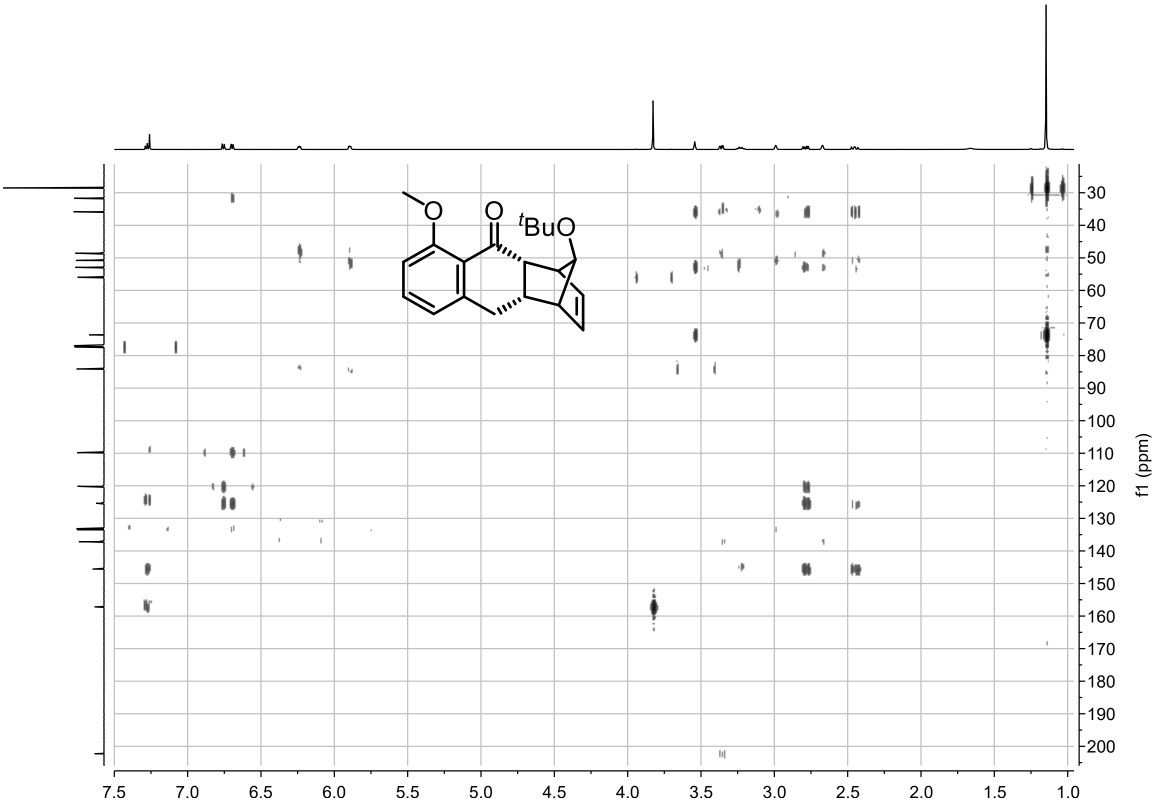

Figure S 210. $^{1}H,^{13}C$-HMBC of the *endo* [2+2] insertion product of **3nb** in $CDCl_3$ measured at ^{1}H: 600.13 MHz; ^{13}C: 150.92 MHz.

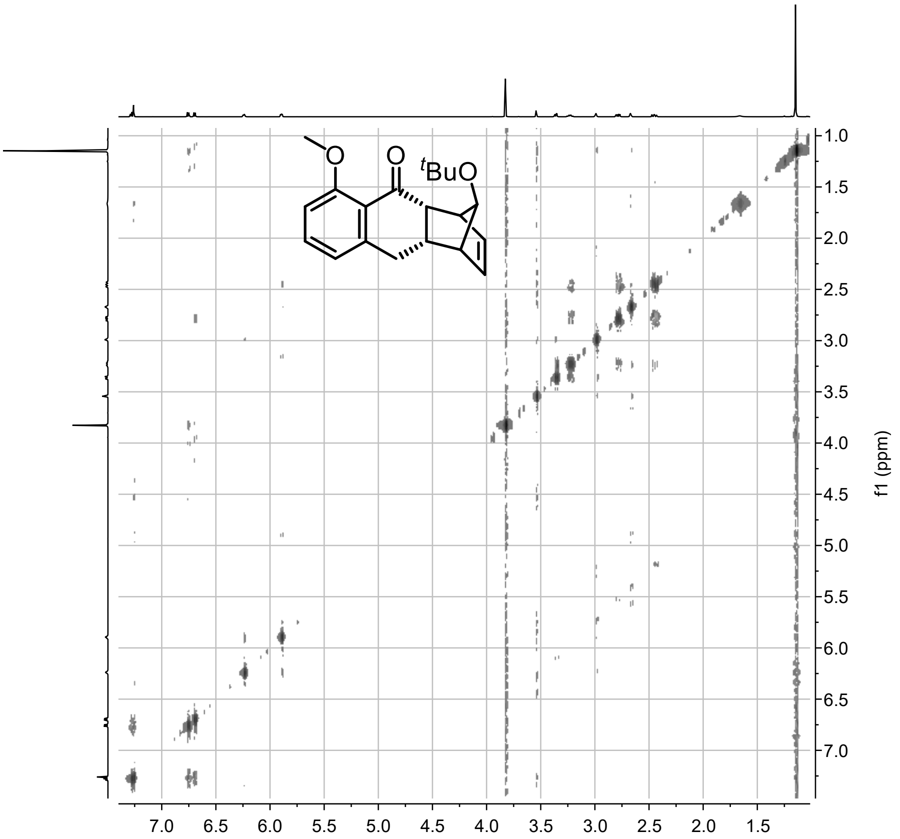

Figure S 211. $^{1}H,^{1}H$-NOESY of the *endo* [2+2] insertion product of **3nb** in $CDCl_3$ measured at 600.13 MHz.

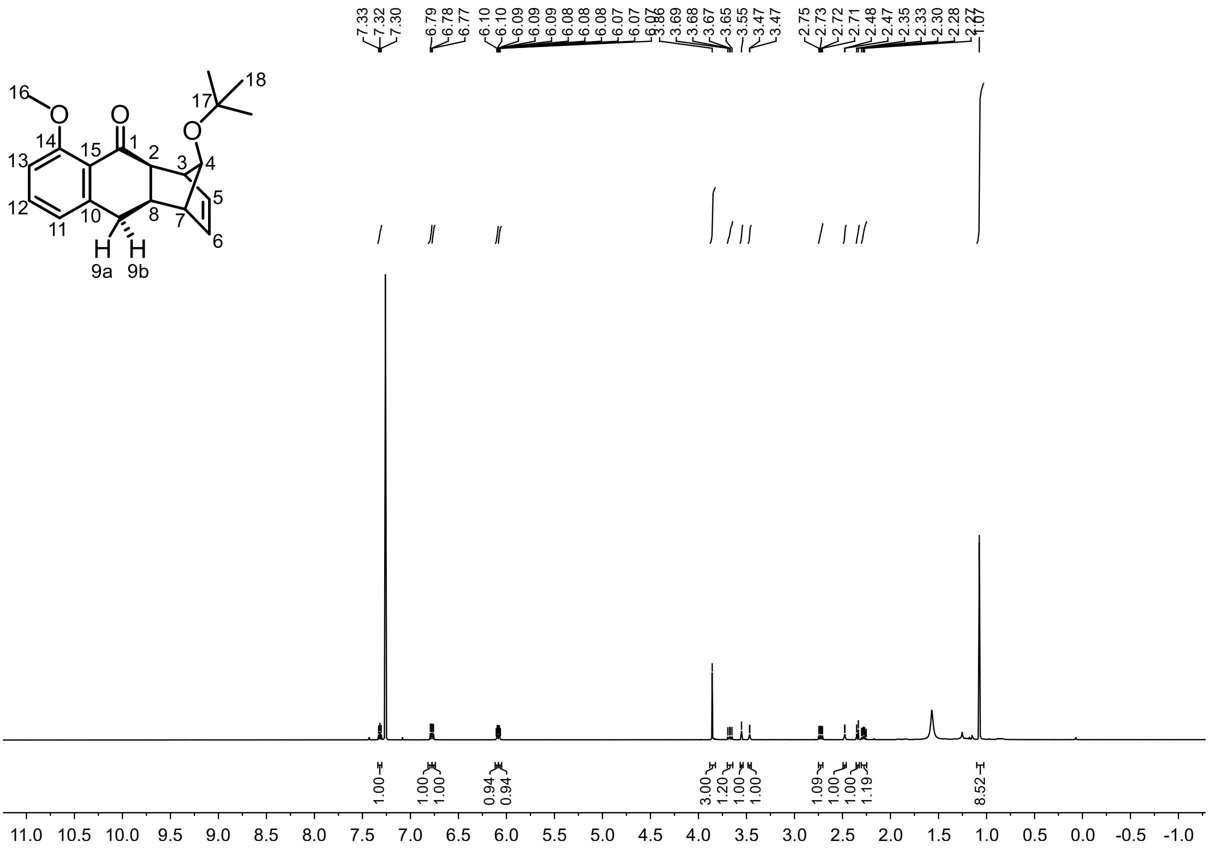

Figure S 212. ^{1}H NMR of the *exo* [2+2] insertion product of **3nb** in $CDCl_3$ measured at 600.13 MHz.

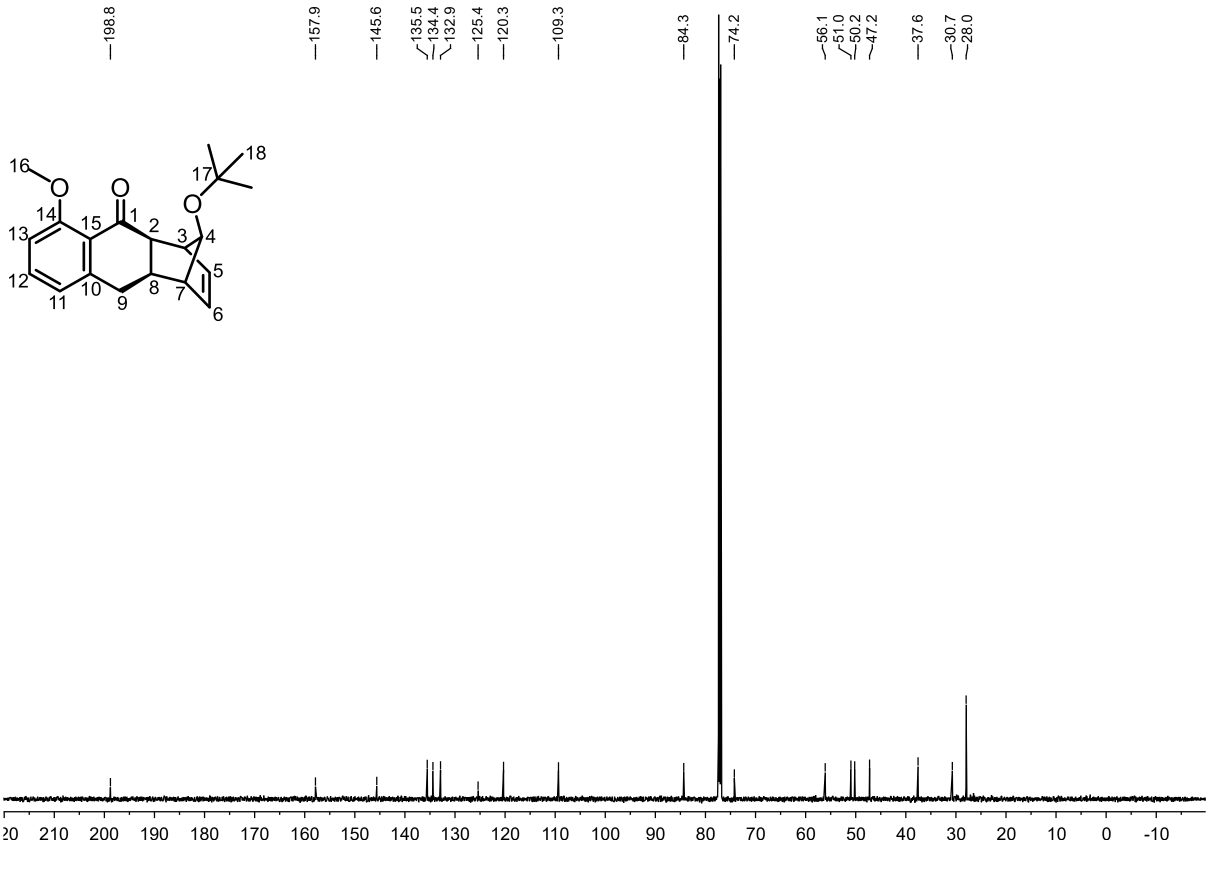

Figure S 213. ^{13}C NMR of the *exo* [2+2] insertion product of **3nb** in $CDCl_3$ measured at 150.92 MHz.

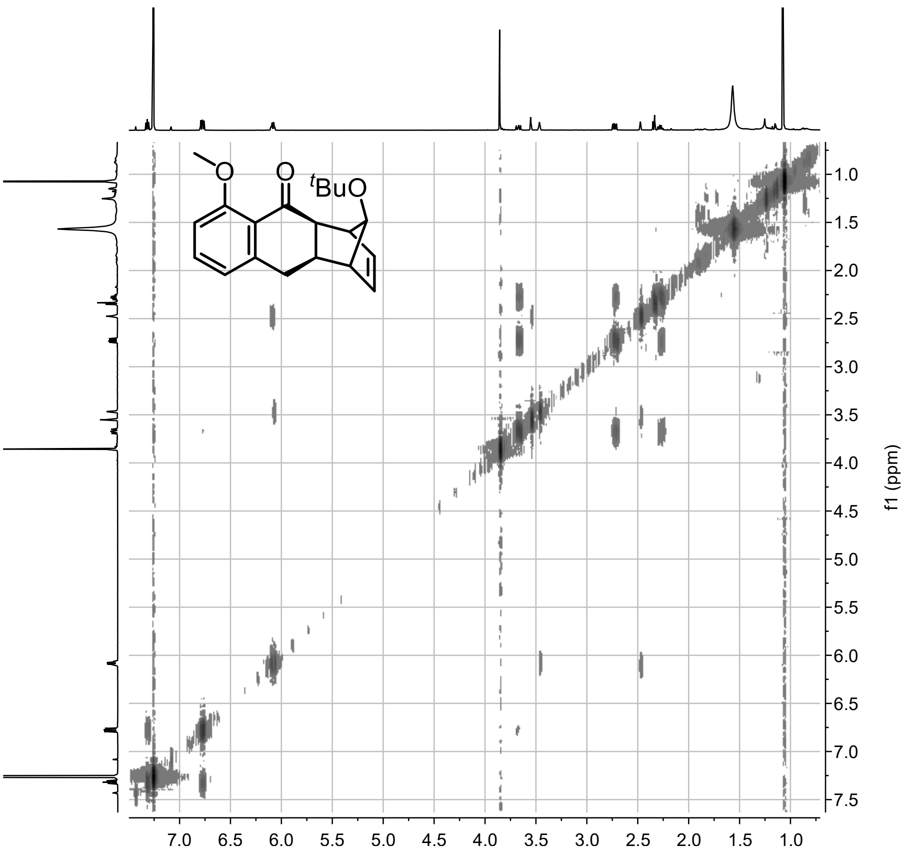

Figure S 214. ^{1}H,^{1}H-COSY of the *exo* [2+2] insertion product of **3nb** in $CDCl_3$ measured at 600.13 MHz.

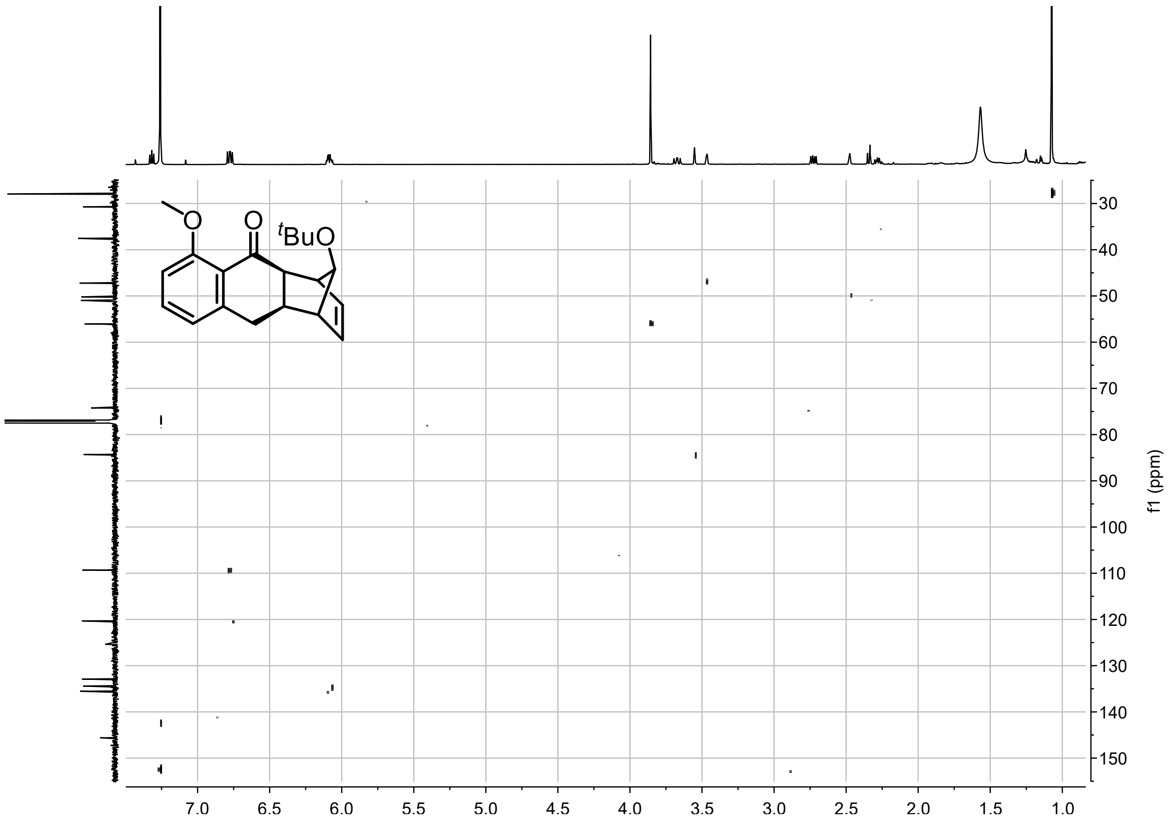

Figure S 215. ^{1}H,^{13}C-HSQC of the *exo* [2+2] insertion product of **3nb** in $CDCl_3$ measured at ^{1}H: 600.13 MHz; ^{13}C: 150.92 MHz.

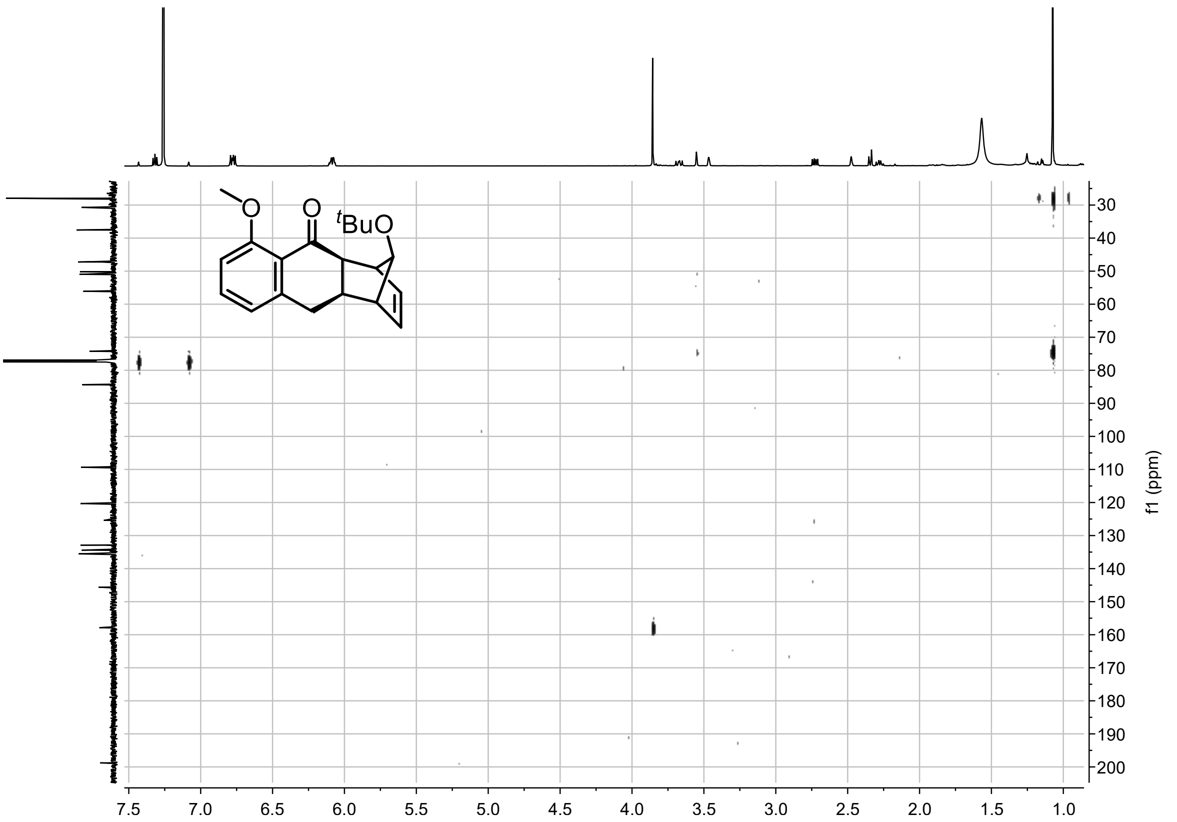

Figure S 216. $^{1}H,^{13}C$-HMBC of the *exo* [2+2] insertion product of **3nb** in $CDCl_3$ measured at ^{1}H: 600.13 MHz; ^{13}C: 150.92 MHz.

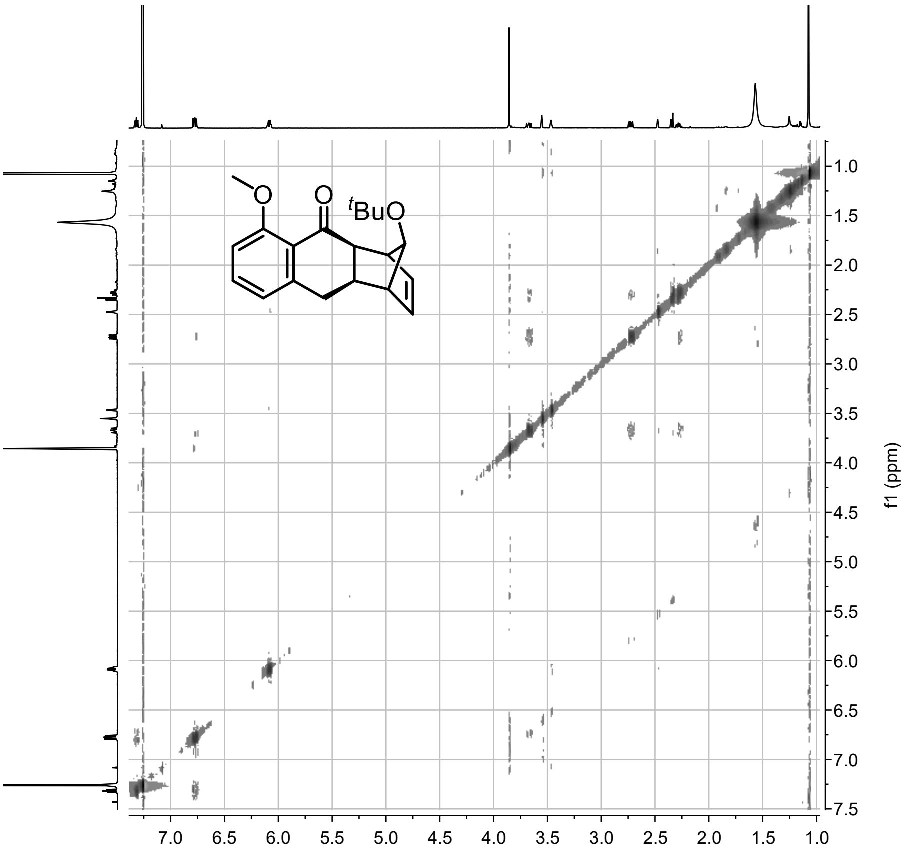

Figure S 217. $^{1}H,^{1}H$-NOESY of the *exo* [2+2] insertion product of **3nb** in $CDCl_3$ measured at 600.13 MHz.

4.6 Derivatisation products

***rel*-1*R*,2*R*,3,3a*S*,4*S*,9,10*S*,10a*R*,11*S*-octahydro-1,2,4-(epimethanetriyl)benzo[f]azulen-10-ol**

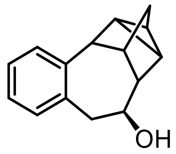

4

4 was synthesized according to a similar literature procedure.[23] $NaBH_4$ (22.7 mg, 600 µmol, 1.20 equiv.) was added to a solution of **3aa** (105 mg, 500 µmol, 1.0 equiv.) in MeOH (5 mL) at rt. The reaction was stirred overnight, quenched with H_2O (5 mL) and the organic phase was extracted with EA (3 × 5 mL). The combined organic phases were dried over Na_2SO_4 and the solvent was removed under reduced pressure. Purification *via* flash chromatography (23 g SiO_2, gradient from 100:00 to 50:50 *n*-hexane/EA over 15 CV) afforded **4** (94.6 mg, 446 µmol, 89%) as a colorless solid.

$C_{15}H_{16}O$ (212.29 $\frac{g}{mol}$)

mp: 103.6 °C.

R$_f$: 0.33 (*n*-hexane/EA = 80:20) [anisaldehyde]

^{1}H NMR(400.16 MHz, $CDCl_3$): δ = 7.17 (m, 1H, H-10), 7.13 (m, 2H, H-11/H-12), 7.10 (m, 1H, H-13), 4.24 (m, 1H, H-1), 3.59 (dm, ^2J = 14.9 Hz, 1H, H-15b), 2.73 (dd, ^2J = 14.9 Hz, ^3J = 5.7 Hz, 1H, H-15a), 2.69 (m, 1H, H-7), 2.29 (dm, ^3J = 6.2 Hz, 1H, H-2), 1.99 (m, 1H, H-8), 1.52 (dm, ^2J = 10.3 Hz, 1H, H-5a), 1.39 (dm, ^2J = 10.3 Hz, 1H, H-5b), 1.36 (m, 1H, H-4), 1.29 (m, 1H, H-6), 1.28 (m, 1H, H-3).

^{13}C NMR(100.62 MHz, $CDCl_3$): δ = 142.1 (C-9), 135.9 (C-14), 132.4 (C-13), 131.0 (C-10), 126.9 (C-11/C-12), 126.6 (C-12/C-11), 68.9 (C-1), 53.2 (C-7), 50.8 (C-2), 39.8 (C-15), 35.9 (C-5), 35.1 (C-8), 16.2 (C-3), 13.6 (C-6), 12.9 (C-4).

HRMS (ESI-TOF) m/z: $[M+Na]^+$ Calcd for $C_{15}H_{16}ONa$ 235.1093; Found 235.1093.

IR (ATR, ṽ): 3313 cm^{-1} (w, *OH*).

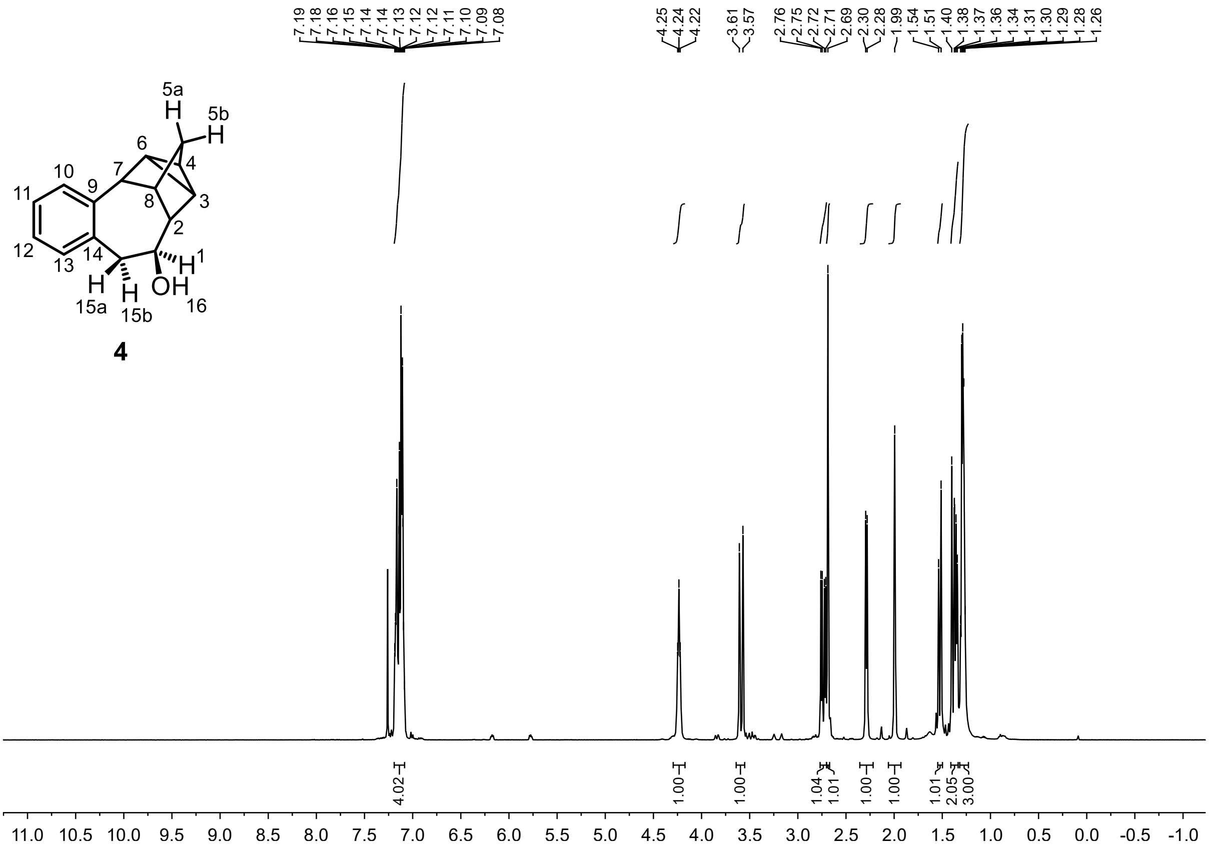

Figure S 218. ^{1}H NMR of **4** in $CDCl_3$ measured at 400.16 MHz.

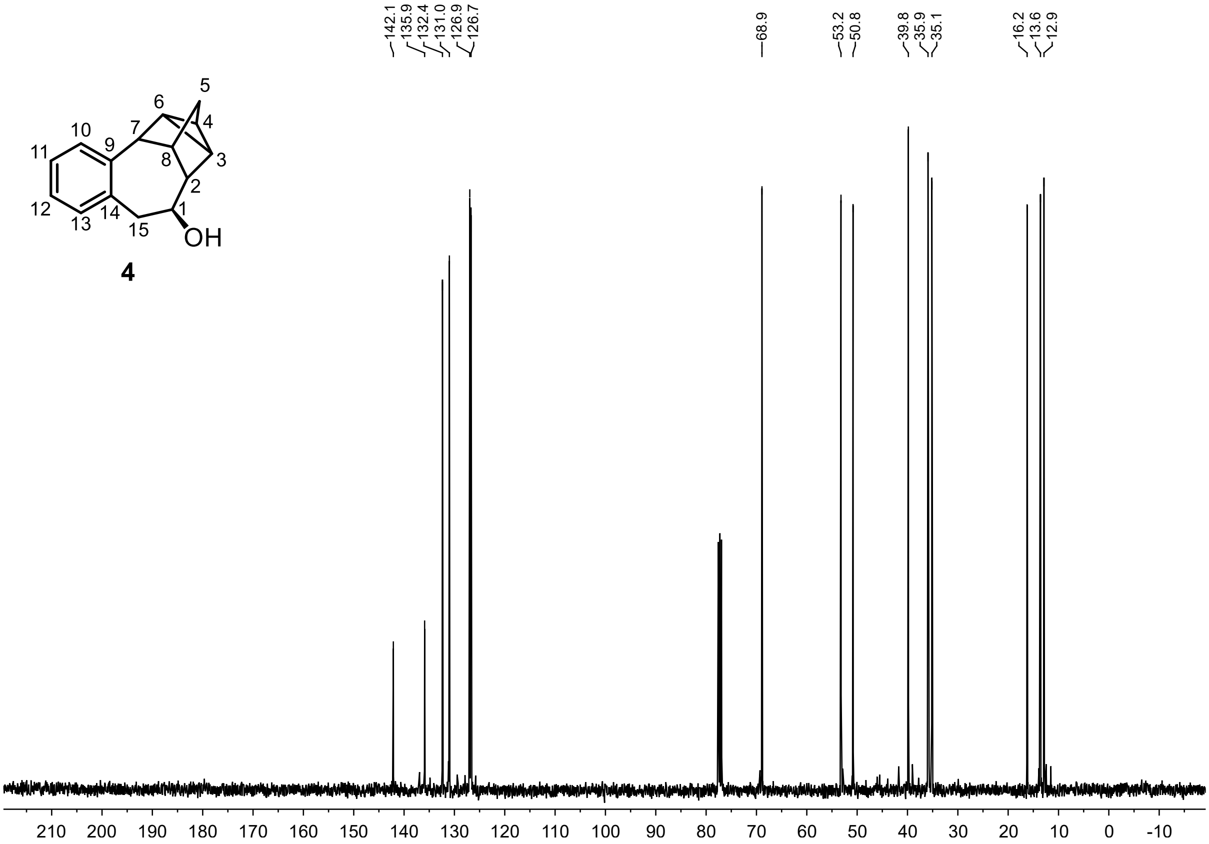

Figure S 219. ^{13}C NMR of **4** in $CDCl_3$ measured at 100.63 MHz.

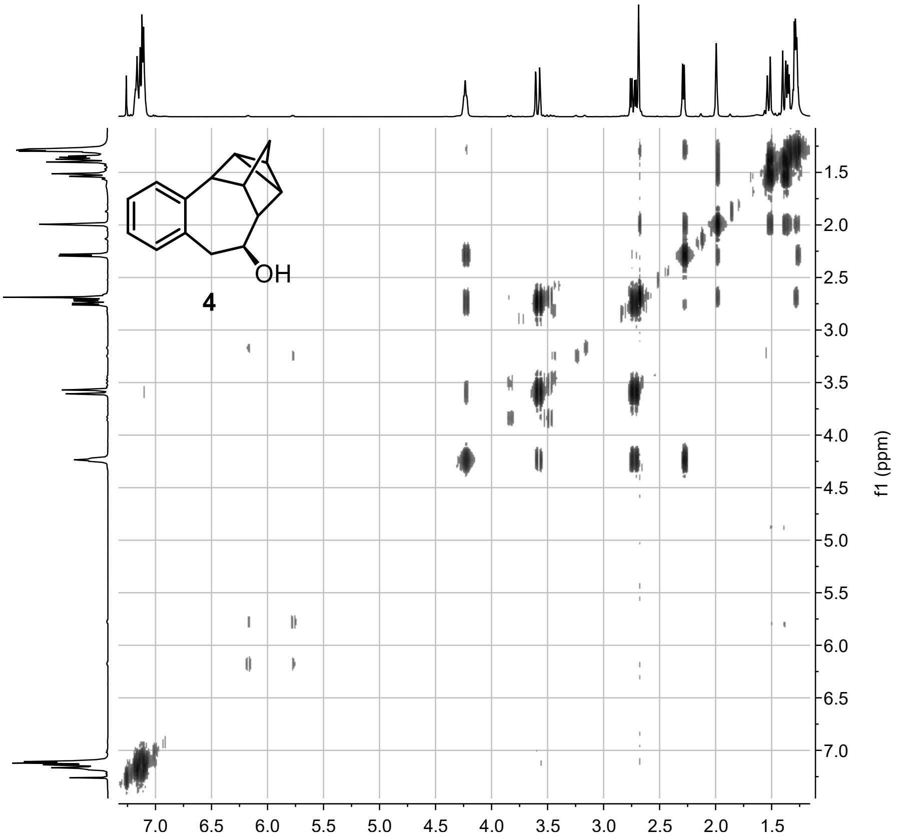

Figure S 220. ^{1}H,^{1}H-COSY of **4** in $CDCl_3$ measured at 400.16 MHz.

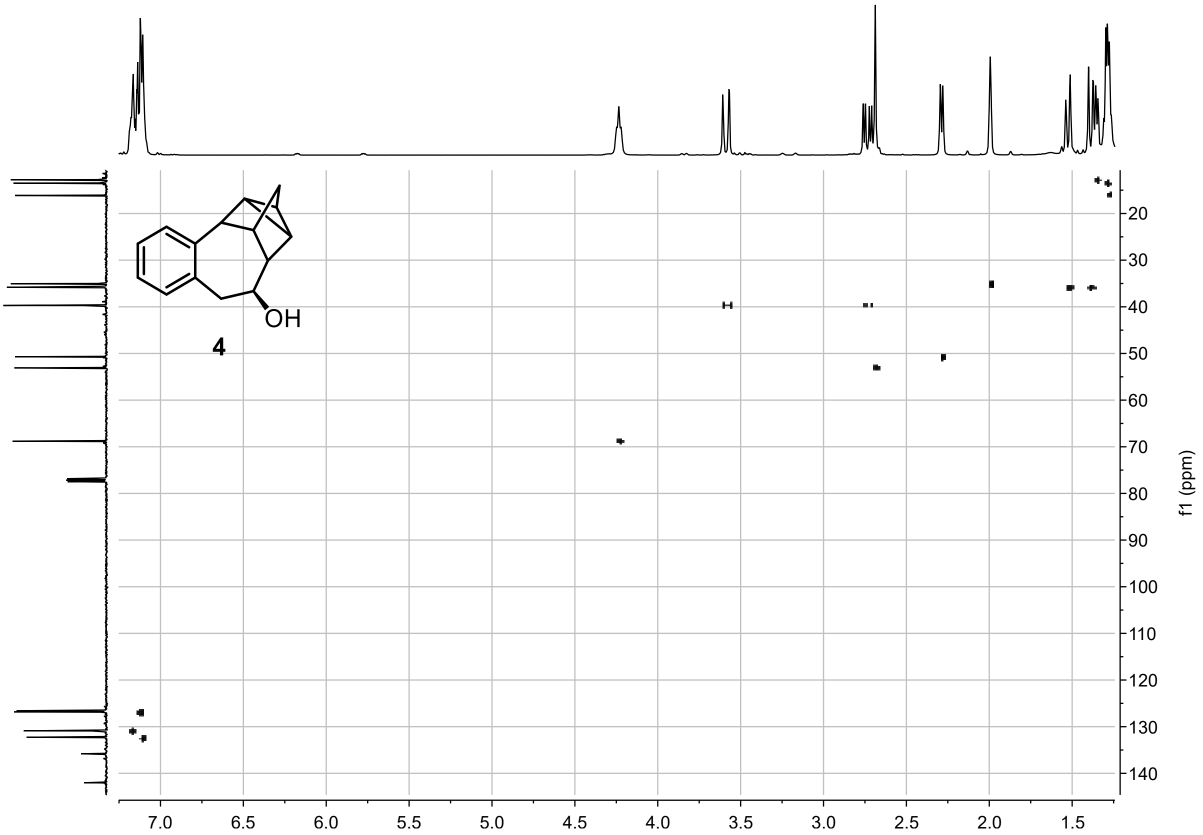

Figure S 221. ^{1}H,^{13}C-HSQC of **4** in $CDCl_3$ measured at ^{1}H: 400.16 MHz; ^{13}C: 100.63 MHz.

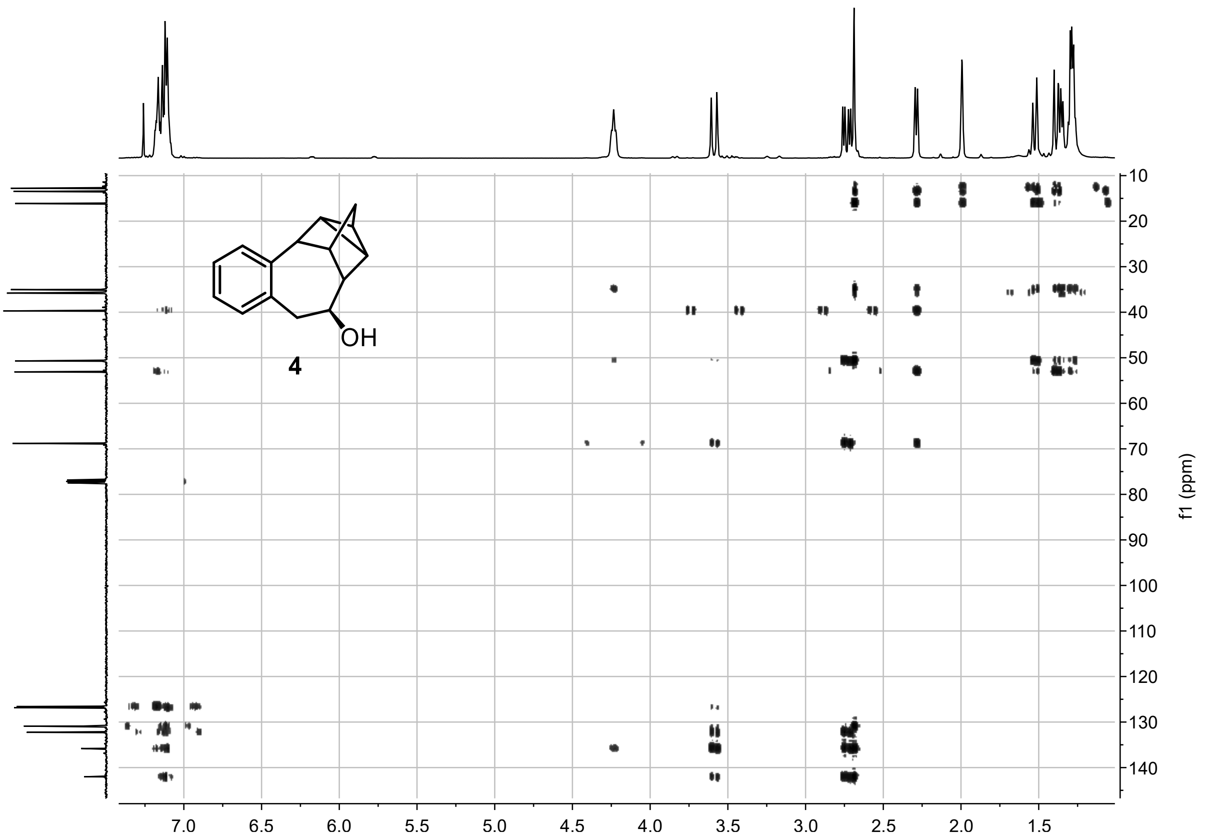

Figure S 222. $^{1}H,^{13}C$-HMBC of **4** in $CDCl_3$ measured at ^{1}H: 400.16 MHz; ^{13}C: 100.63 MHz.

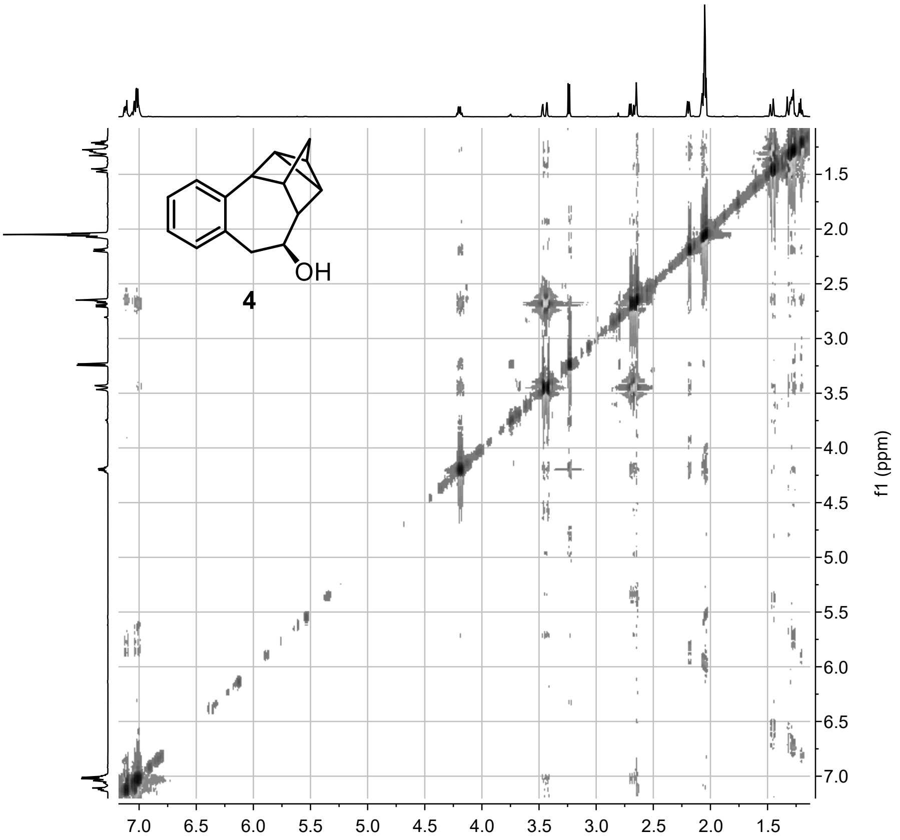

Figure S 223. $^{1}H,^{1}H$-NOESY of **4** in acetone-d6 measured at 400.16 MHz.

***rel*-1*R*,2*S*,2'*R*,3',3a'*S*,4'*S*,9',10a'*S*,11*S*-hexahydro-1'H-spiro[oxirane-2,10'-[1,2,4](epimethanetriyl)benzo[f]azulene]**

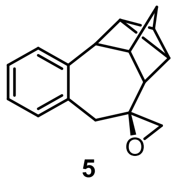

5

5 was synthesized according to a similar literature procedure with slight modifications.[24] In a dried Schlenk tube, a solution of **3aa** (105 mg, 500 µmol, 1.0 equiv.) in dry DMSO/THF (3 mL/1 mL) was slowly added to a cooled (0 °C) solution of NaH (36.0 mg, 1.50 mmol, 3.00 equiv.) in dry THF (1 mL). The reaction was allowed to warm to rt and stirred for 3 h, after which H_2O (5 mL) was added. The aqueous phase was extracted with EA (3 × 5 mL) and the combined organic extracts were washed with brine (3 × 5 mL), dried over $MgSO_4$ and the solvent was removed under reduced pressure. The crude product was recrystallized from *n*-hexane/toluene (98/2) furnishing **5** (91.3 mg, 407 µmol, 81%) as a colorless solid.

*Note: **5** decomposes in contact with silica.*

$C_{16}H_{16}O$ (224.30 $\frac{g}{mol}$)

mp: 102.6 °C.

^{1}H NMR(400.16 MHz, $CDCl_3$): δ = 7.19 (dm, ^3J = 7.1 Hz, 1H, H-10), 7.13 (m, 2H, H-11, H-12), 7.00 (d, ^3J = 6.7 Hz, 1H, H-13), 3.92 (d, ^2J = 15.0 Hz, 1H, H-15b), 2.78 (m, 1H, H-7), 2.77 (d, ^2J = 5.2 Hz, 1H, H-16a), 2.72 (d, ^2J = 5.2 Hz, 1H, H-16b), 2.15 (d, ^2J = 15.0 Hz, 1H, H-15a), 2.12 (m, 1H, H-8), 1.63 (m, 1H, H-2), 1.52 (dm, ^2J = 10.6 Hz, 1H, H-5a), 1.46 (m, 1H, H-3), 1.41 (m, 1H, H-6), 1.38 (dm, ^2J = 10.6 Hz, 1H, H-5b), 1.37 (m, 1H, H-4).

^{13}C NMR(100.62 MHz, $CDCl_3$): δ = 140.8 (C-9), 136.4 (C-14), 131.0 (C-10), 130.8 (C-13), 126.6 (C-11, C-12), 58.3 (C-1), 57.8 (C-16), 52.9 (C-7), 51.5 (C-2), 40.2 (C-15), 37.8 (C-8), 35.4 (C-5), 16.2 (C-3), 14.2 (C-6), 12.6 (C-4).

HRMS (ESI-TOF) m/z: $[M+Na]^+$ Calcd for $C_{16}H_{16}ONa$ 247.1093; Found 247.1093.

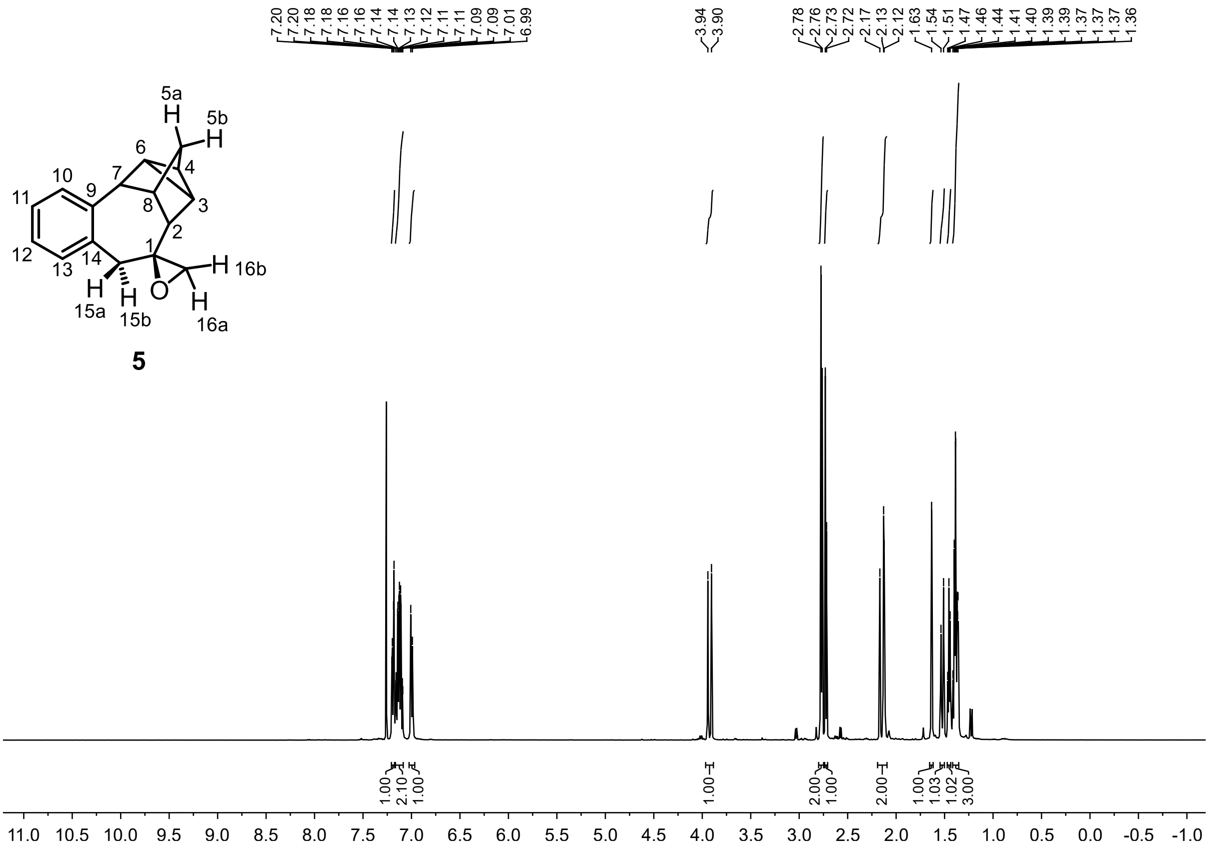

Figure S 224. ^{1}H NMR of **5** in $CDCl_3$ measured at 400.16 MHz.

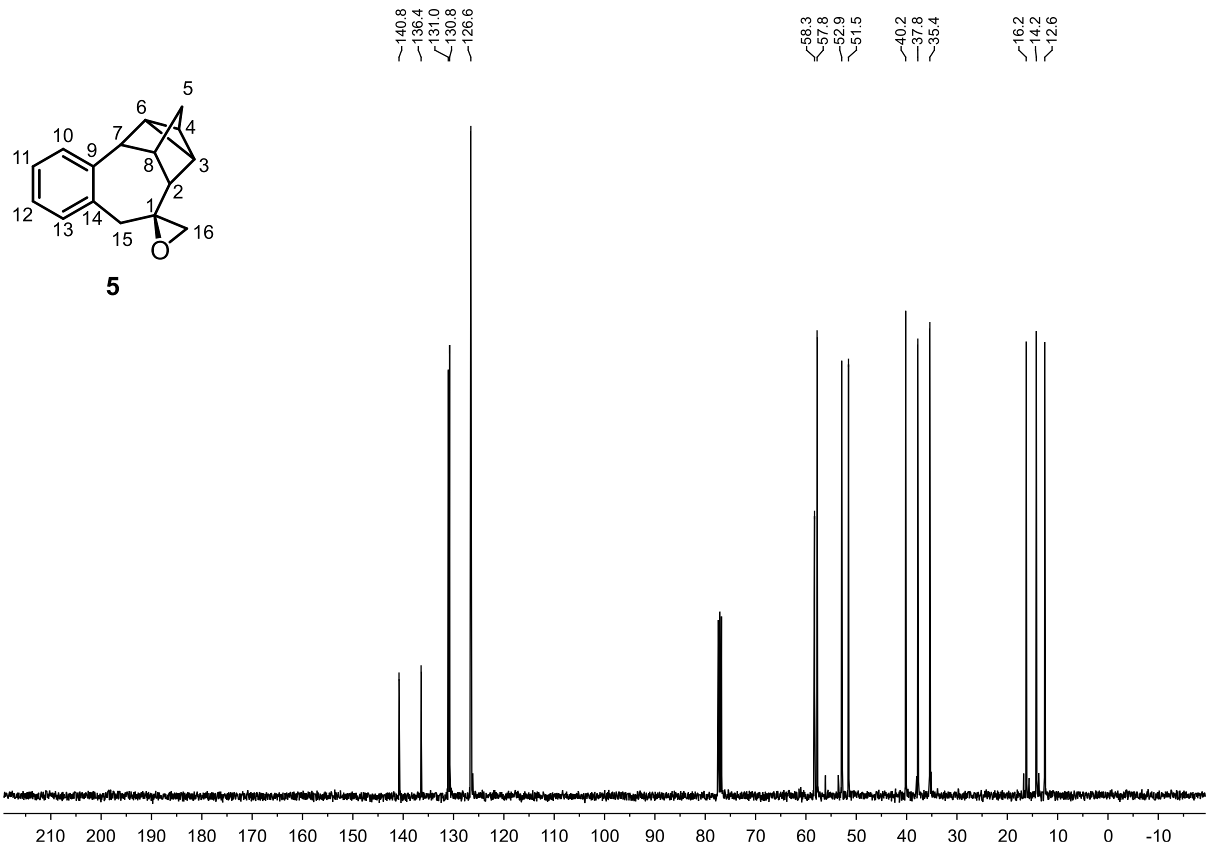

Figure S 225. ^{13}C NMR of **5** in $CDCl_3$ measured at 100.63 MHz.

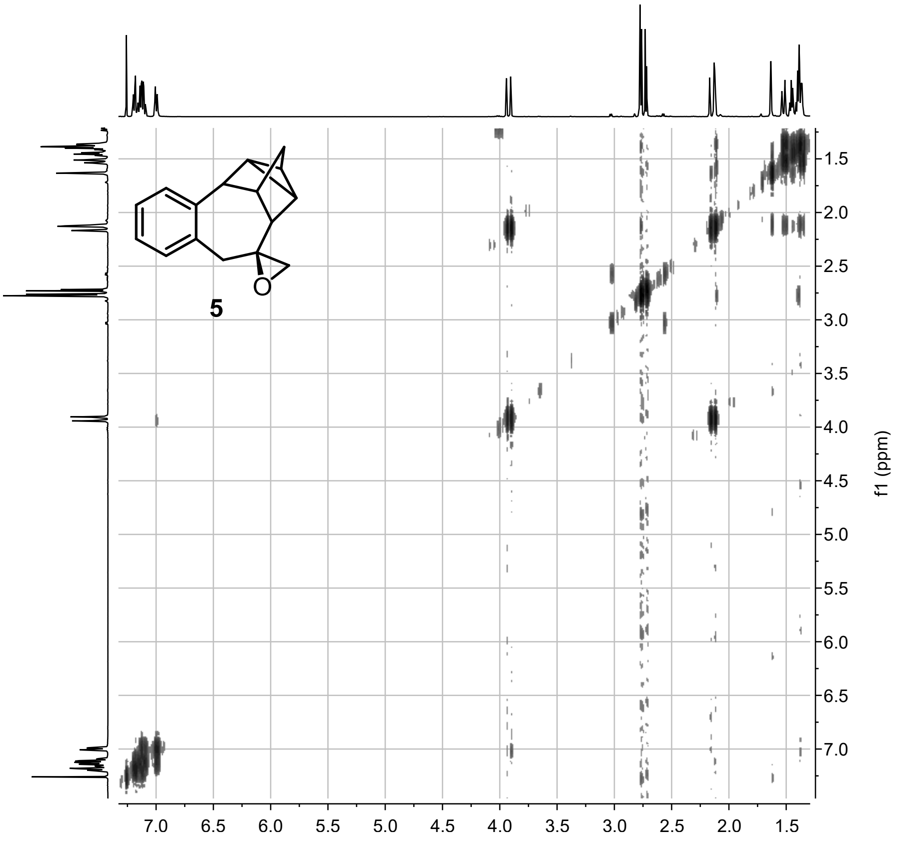

Figure S 226. $^{1}H,^{1}H$-COSY of **5** in $CDCl_3$ measured at 400.16 MHz.

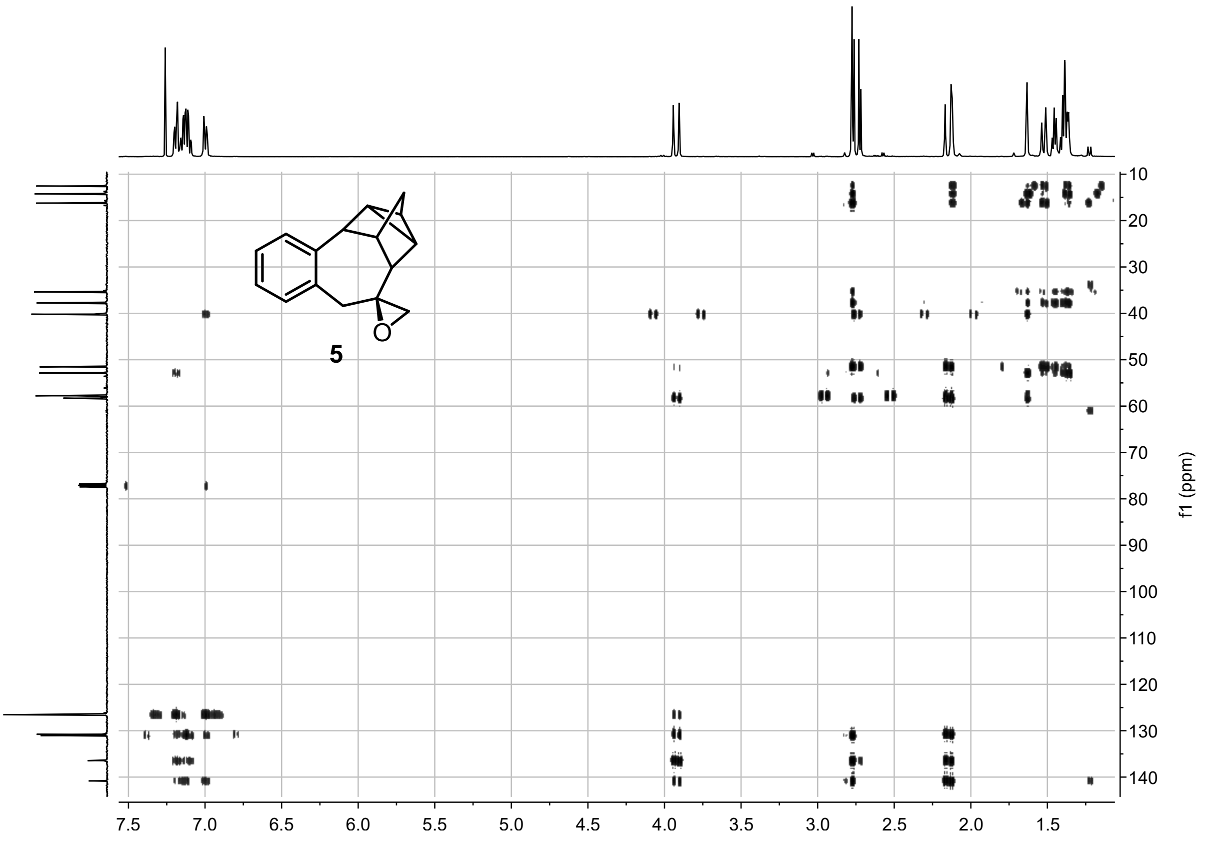

Figure S 227. $^{1}H,^{13}C$-HSQC of **5** in $CDCl_3$ measured at ^{1}H: 400.16 MHz; ^{13}C: 100.63 MHz.

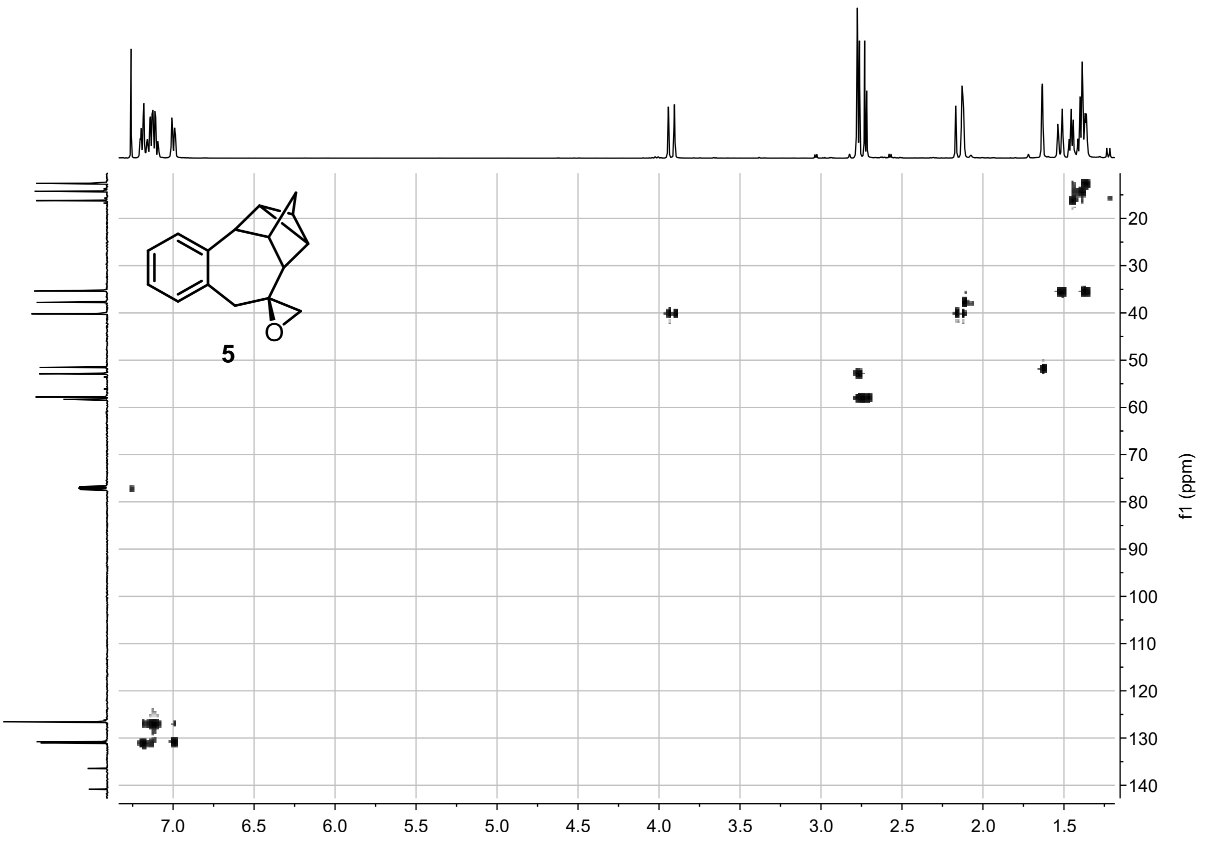

Figure S 228. $^{1}H,^{13}C$-HMBC of **5** in $CDCl_3$ measured at ^{1}H: 400.16 MHz; ^{13}C: 100.63 MHz.

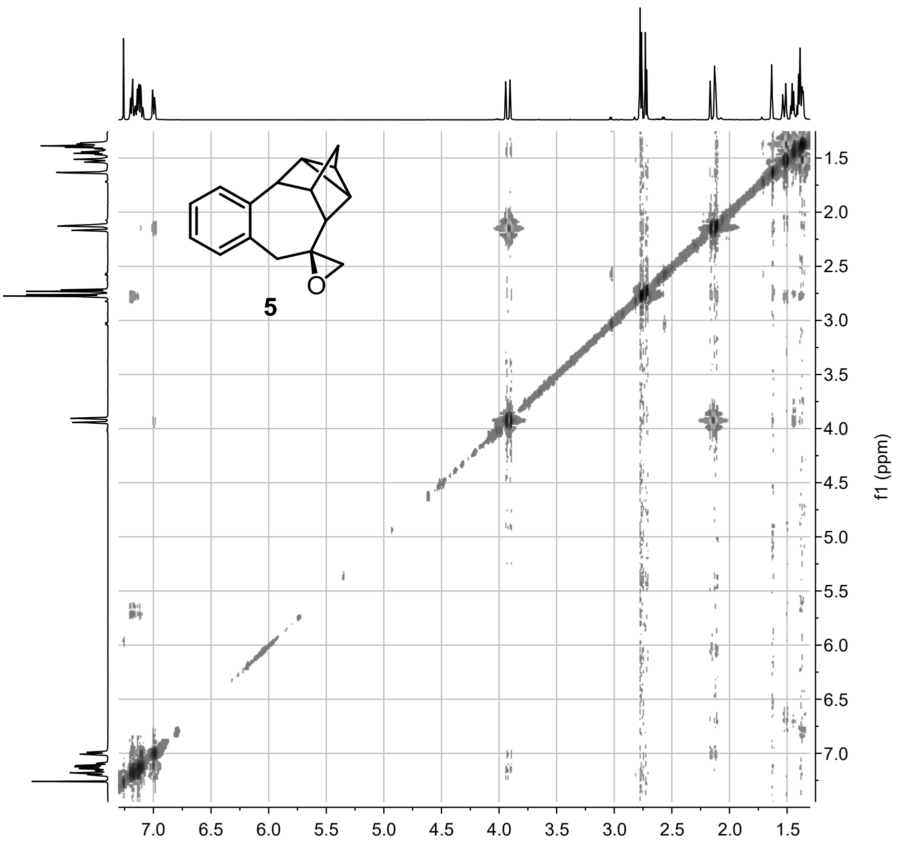

Figure S 229. $^{1}H,^{1}H$-NOESY of **5** in $CDCl_3$ measured at 400.16 MHz.

***rel*-10-phenyl-1*R*,2*R*,3,3a*S*,4*S*,9,10*S*,10a*R*,11*S*-octahydro-1,2,4-(epimethanetriyl)benzo[f]azulen-10-ol**

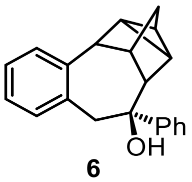

6

In a dried Schlenk tube, PhMgBr (750 µL, 1.00 M, 1.50 equiv.) was added to a solution of **3aa** (105 mg, 500 µmol, 1.0 equiv.) in THF (4 mL). The reaction was stirred for 4 h, after which sat. $NH_4Cl_{(aq.)}$ (2 mL) was added. The aqueous phase was extracted with EA (3 × 5 mL) and the combined organic phases were dried over Na_2SO_4. The solvent was removed *in vacuo*. Purification *via* flash chromatography (23 g SiO_2, gradient from 100:00 to 50:50 *n*-hexane/EA over 15 CV) afforded **5** (93.2 mg, 323 µmol, 65%) as a colorless solid.

$C_{21}H_{20}O$ (288.39 $\frac{g}{mol}$)

mp: 145.3 °C.

R_f: 0.70 (*n*-hexane/EA = 80:20) [anisaldehyde]

^{1}H NMR(600.13 MHz, $CDCl_3$): δ = 7.69 (d, ^3J = 7.7 Hz, 2H, H-17), 7.41 (m, 2H, H-18), 7.31 (m, 1H, H-19), 7.25 (dd, ^3J = 7.5 Hz, ^4J = 1.1 Hz, 1H, H-10), 7.21 (m, 1H, H-11), 7.17 (m, 1H, H-12), 7.11 (d, ^3J = 7.2 Hz, 1H, H-13), 4.26 (d, ^2J = 14.4 Hz, 1H, H-15b), 2.82 (d, ^2J = 14.4 Hz, 1H, H-15a), 2.79 (m, 1H, H-7), 2.21 (m, 1H, H-2), 2.13 (m, 1H, H-8), 1.63 (s, 1H, H-20), 1.55 (dm, ^2J = 10.5 Hz, 1H, H-5a), 1.41 (m, 1H, H-3), 1.37 (dm, ^2J = 10.5 Hz, 1H, H-5b), 1.37 (m, 1H, H-6), 1.32 (m, 1H, H-4).

^{13}C NMR(100.62 MHz, $CDCl_3$): δ = 150.1 (C-16), 141.9 (C-9), 135.5 (C-14), 132.6 (C-13), 131.1 (C-10), 128.3 (C-18), 127.2 (C-11), 126.9 (C-12), 126.9 (C-19), 125.0 (C-17), 73.5 (C-1), 58.0 (C-2), 53.1 (C-7), 44.8 (C-15), 36.9 (C-8), 35.8 (C-5), 16.0 (C-3), 13.4 (C-6), 13.1 (C-4).

HRMS (ESI-TOF) m/z: $[M+Na]^+$ Calcd for $C_{21}H_{20}ONa$ 311.1406; Found 311.1406.

IR (ATR, ṽ): 3577 cm^{-1} (w, *OH*).

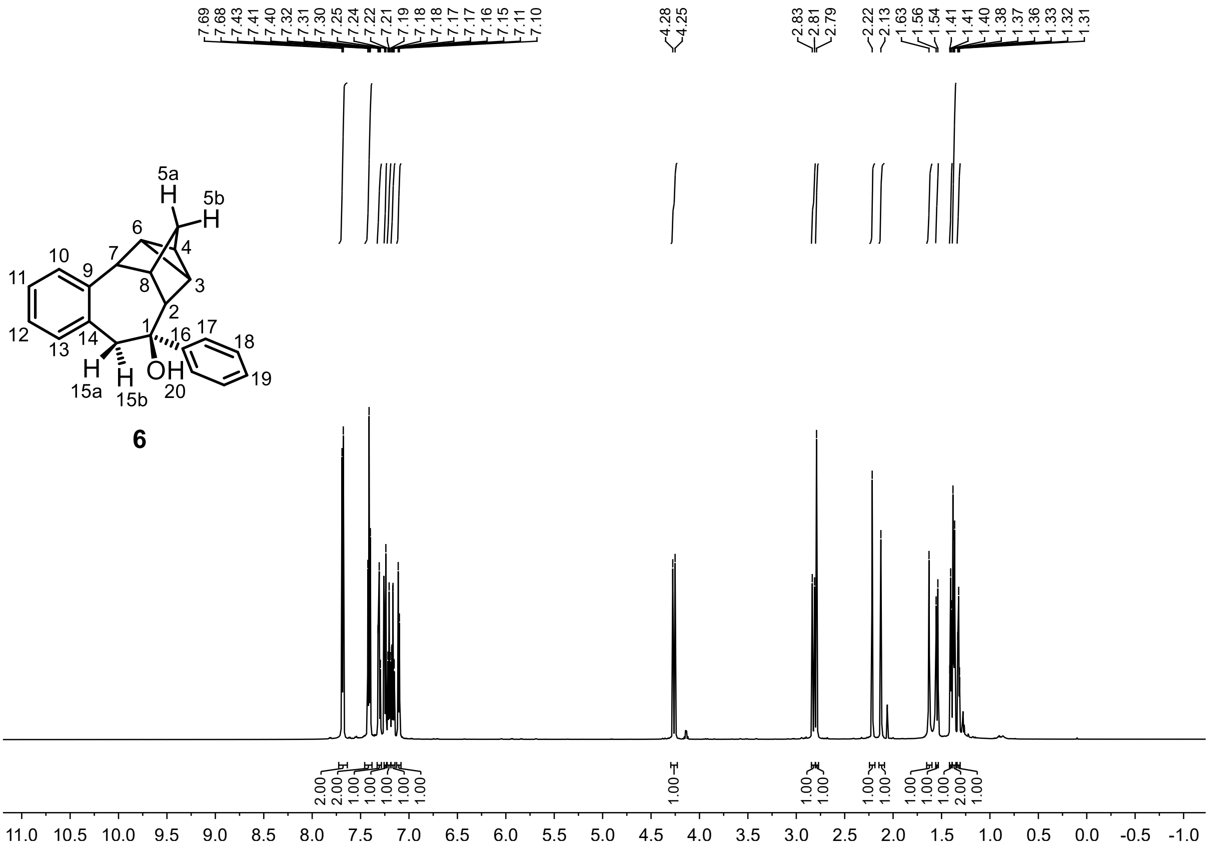

Figure S 230. ^{1}H NMR of **6** in $CDCl_3$ measured at 400.16 MHz.

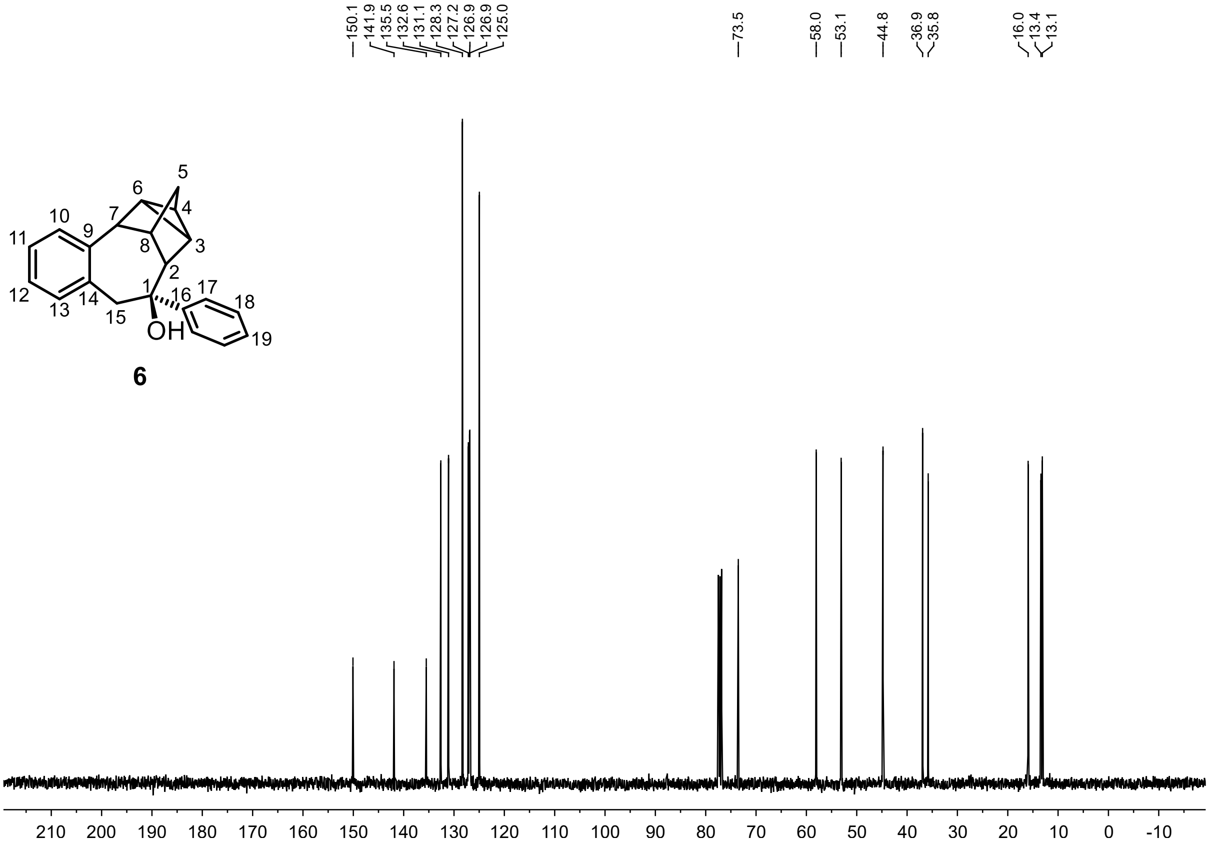

Figure S 231. ^{13}C NMR of **6** in $CDCl_3$ measured at 100.63 MHz.

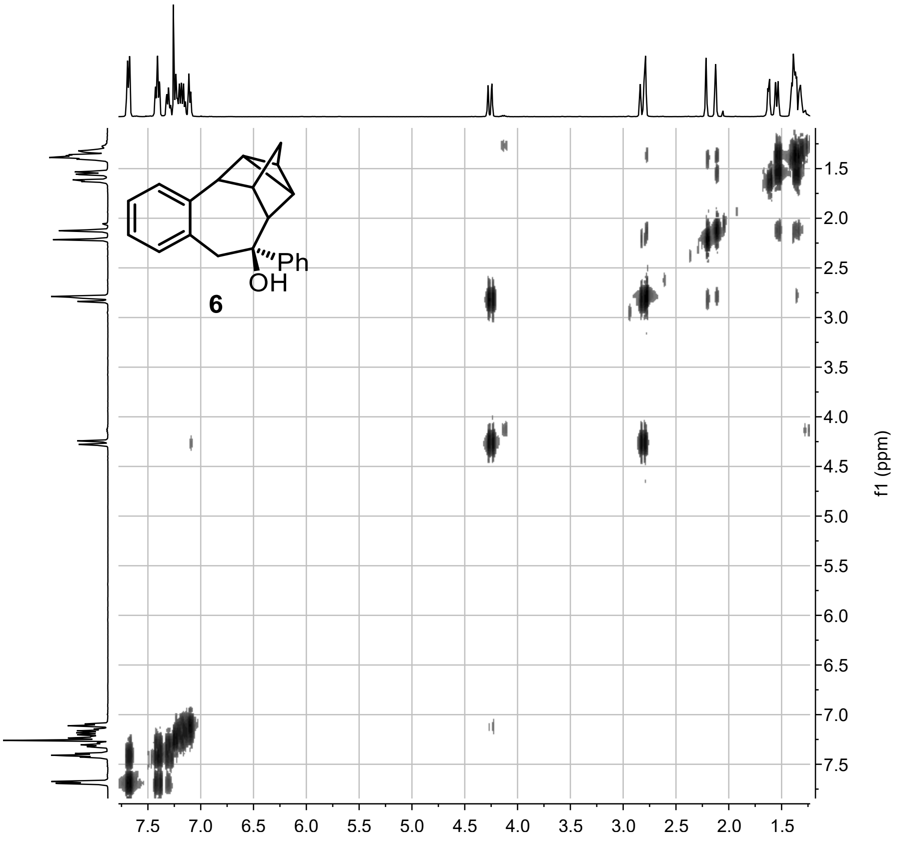

Figure S 232. $^{1}H,^{1}H$-COSY of **6** in $CDCl_3$ measured at 400.16 MHz.

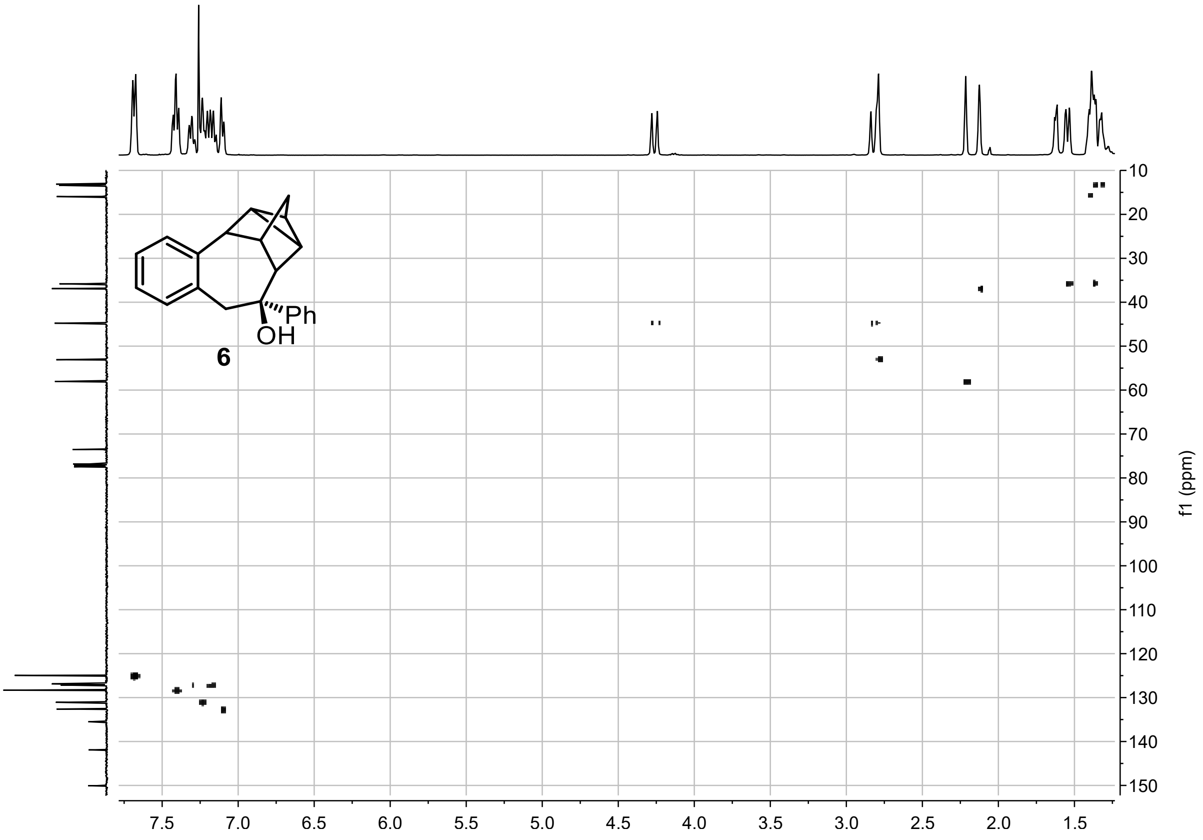

Figure S 233. $^{1}H,^{13}C$-HSQC of **6** in $CDCl_3$ measured at ^{1}H: 400.16 MHz; ^{13}C: 100.63 MHz.

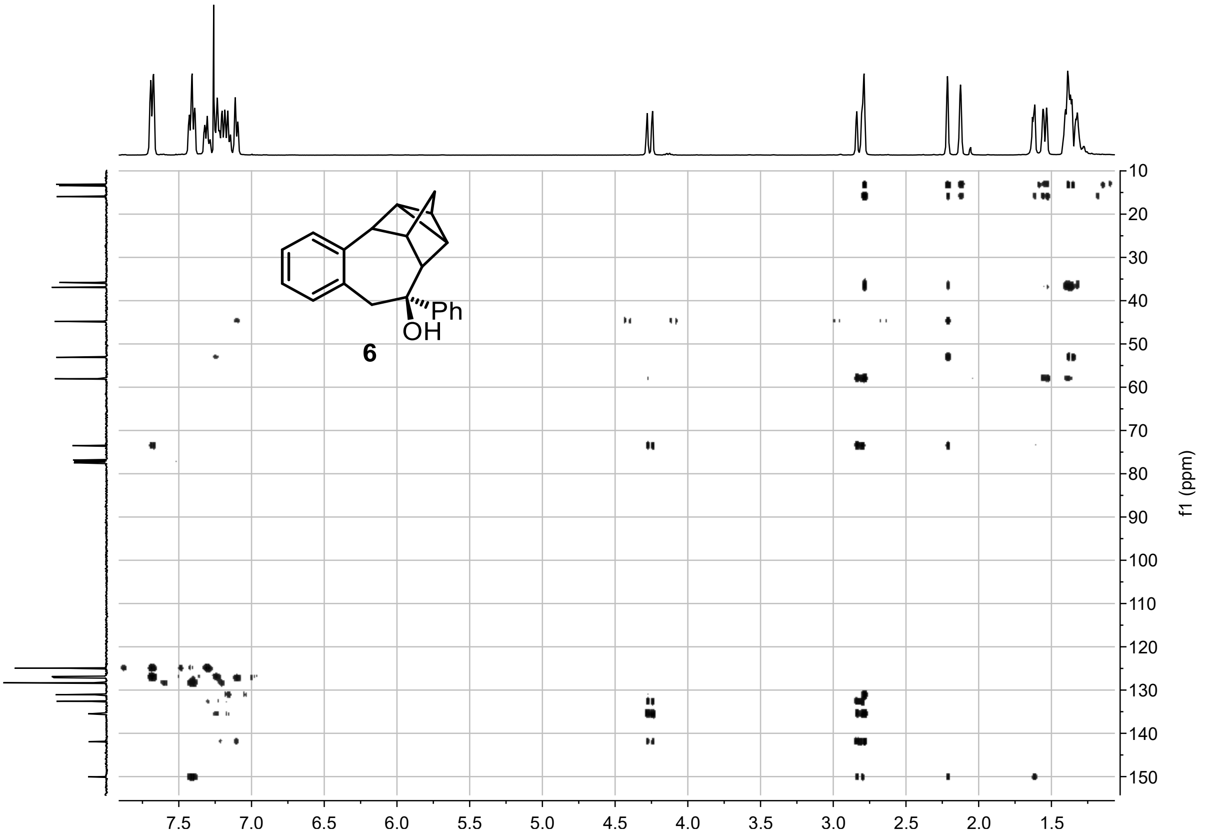

Figure S 234. $^{1}H,^{13}C$-HMBC of **6** in $CDCl_3$ measured at ^{1}H: 400.16 MHz; ^{13}C: 100.63 MHz.

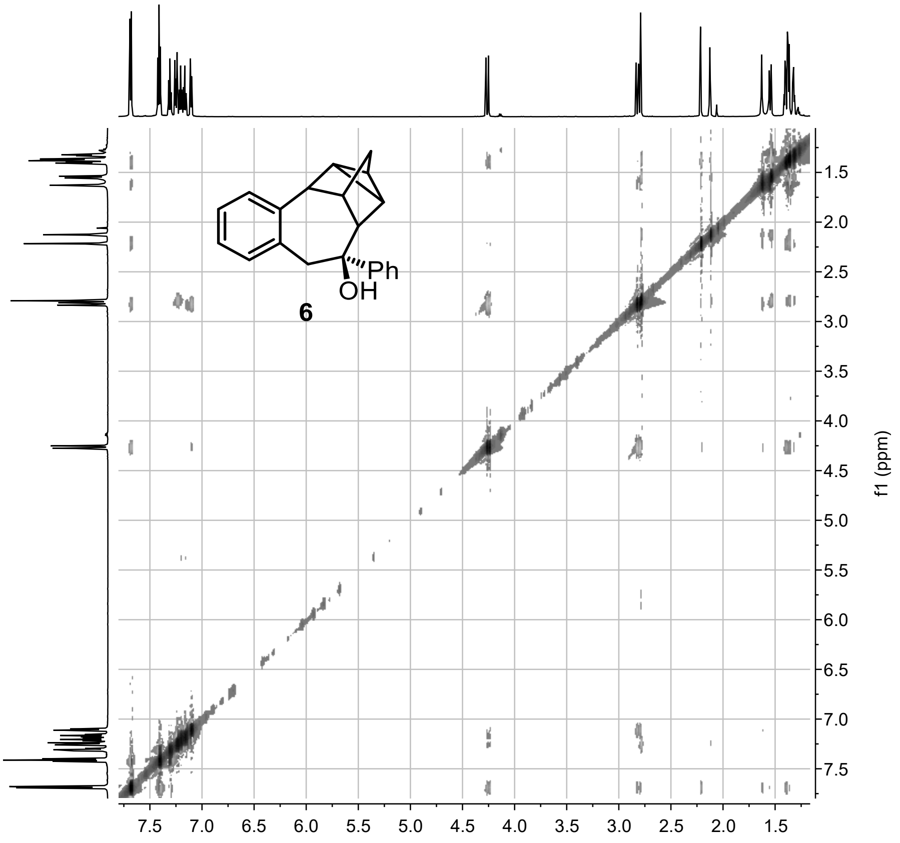

Figure S 235. $^{1}H,^{1}H$-NOESY of **6** in $CDCl_3$ measured at 400.16 MHz.

***rel*-1*S*,2*R*,3,3a*S*,4*R*,7,11b*S*,12*R*-hexahydro-1,2,4-(epimethanetriyl)benzo[c]cyclopenta[e]oxocin-5(1H)-one**

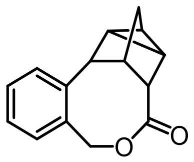

7

7 was synthesized according to a similar literature procedure with slight modifications.[25] **3aa** (105 mg, 500 µmol, 1.0 equiv.) and *m*-CPBA (140 mg, 1.50 mmol, 1.50 equiv.) were dissolved in DCM (2 mL) at 0 °C. The reaction was allowed to warm to rt and stirred for 4 d. Subsequently *m*-CPBA (140 mg, 1.50 mmol, 1.50 equiv.) was added and the reaction was stirred for 1 d. Reaction completion was confirmed by TLC, after which the precipitated was filtered. The organic phase was washed with sat. $NaHCO_{3(aq.)}$, dried over Na_2SO_4 and the solvent was removed *in vacuo*. Purification *via* flash chromatography (23 g SiO_2, gradient from 100:00 to 63:37 *n*-hexane/EA over 18 CV) afforded **7** (60.7 mg, 268 µmol, 54%) as a colorless solid.

$C_{15}H_{14}O_2$ (226.28 $\frac{g}{mol}$)

mp: 110.9 °C.

R$_f$: 0.25 (*n*-hexane/EA = 80:20) [anisaldehyde]

^{1}H NMR(400.16 MHz, $CDCl_3$): δ = 7.26 (m, 4H, H-10, H-11, H-12, H-13), 6.60 (d, ^2J = 12.4 Hz, 1H, H-15b), 4.80 (d, ^2J = 12.4 Hz, 1H, H-15a), 3.02 (s, 1H, H-7), 2.88 (s, 1H, H-2), 2.37 (m, 1H, H-8), 1.85 (m, 1H, H-3), 1.61 (m, 1H, H-4, H-4), 1.60 (m, 1H, H-6), 1.58 (dm, ^2J = 10.7 Hz, 1H, H-5a), 1.50 (dm, ^2J = 10.7 Hz, 1H, H-5b).

^{13}C NMR(100.62 MHz, $CDCl_3$): δ = 175.0 (C-1), 139.9, 135.4, 132.9, 132.5, 129.6, 127.5, 68.7 (C-15), 53.4 (C-7), 51.9 (C-2), 40.9 (C-8), 34.7 (C-5), 16.9 (C-6), 15.0 (C-3), 14.7 (C-3).

HRMS (ESI-TOF) m/z: $[M+H]^+$ Calcd for $C_{15}H_{14}O_2H$ 227.1067; Found 227.1069.

IR (ATR, ṽ): 1701 cm^{-1} (s, *CO*OR).

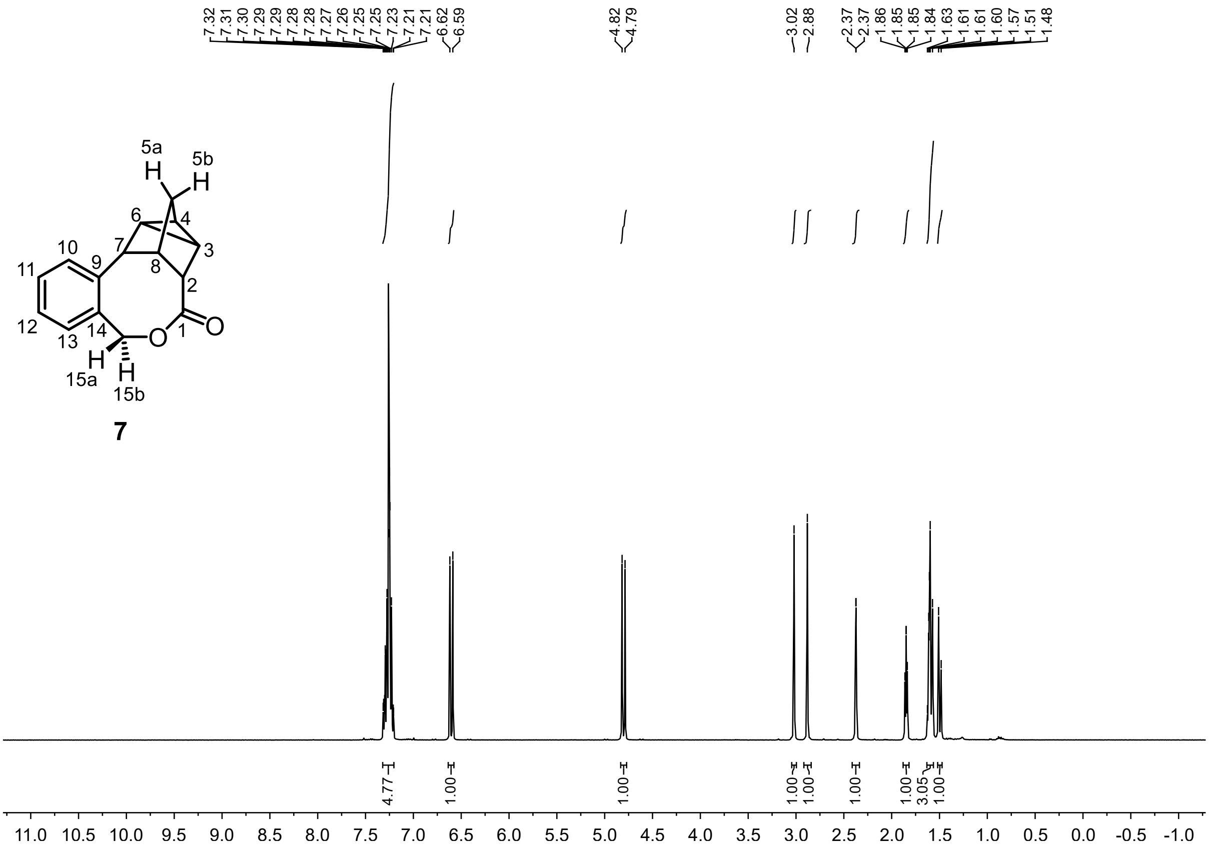

Figure S 236. ^{1}H NMR of **7** in $CDCl_3$ measured at 400.16 MHz.

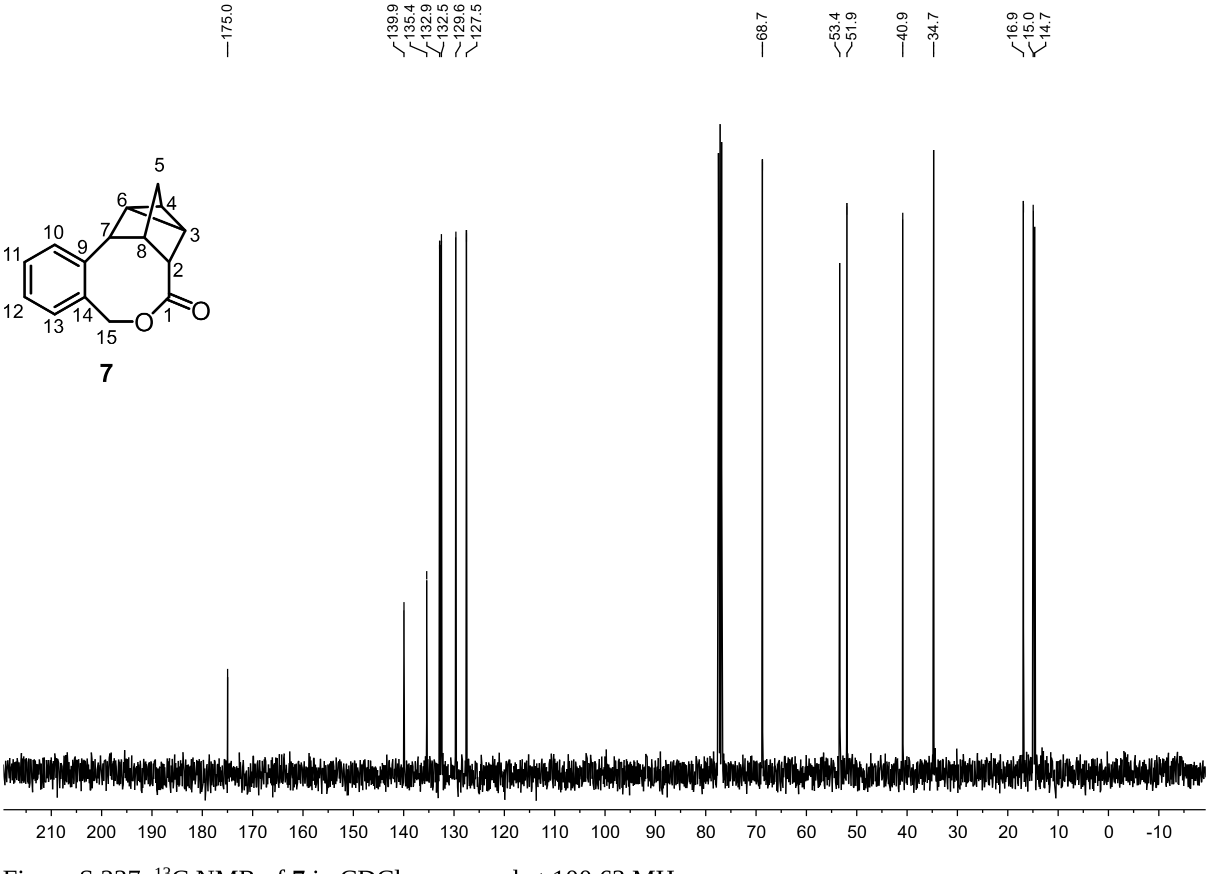

Figure S 237. ^{13}C NMR of **7** in $CDCl_3$ measured at 100.63 MHz.

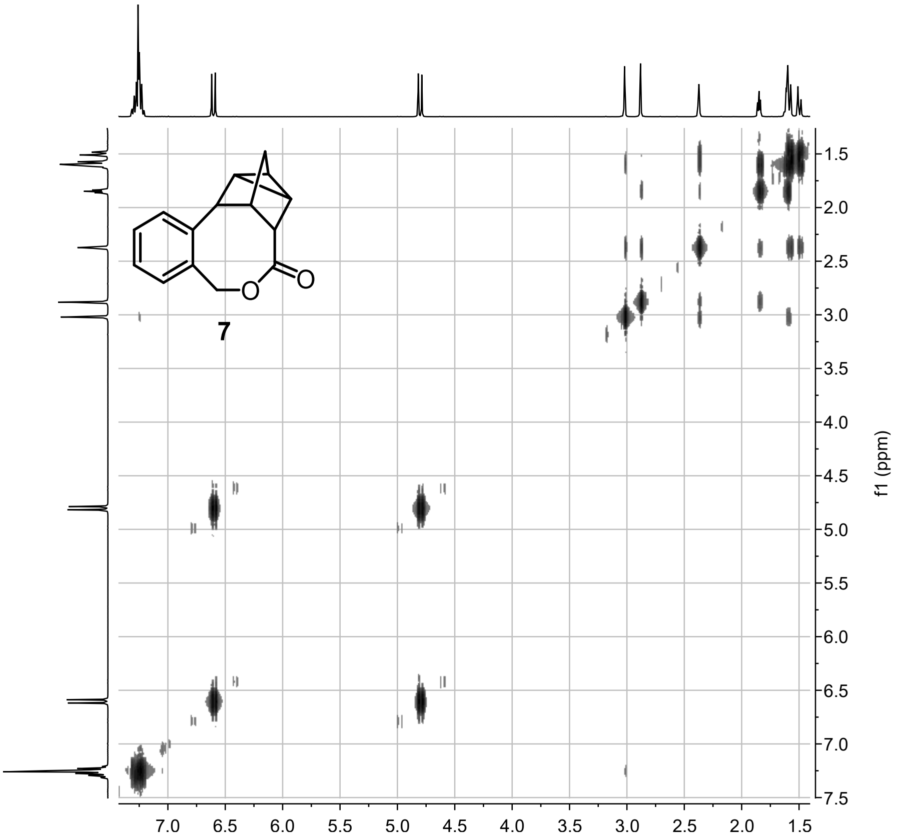

Figure S 238. $^{1}H,^{1}H$-COSY of **7** in $CDCl_3$ measured at 400.16 MHz.

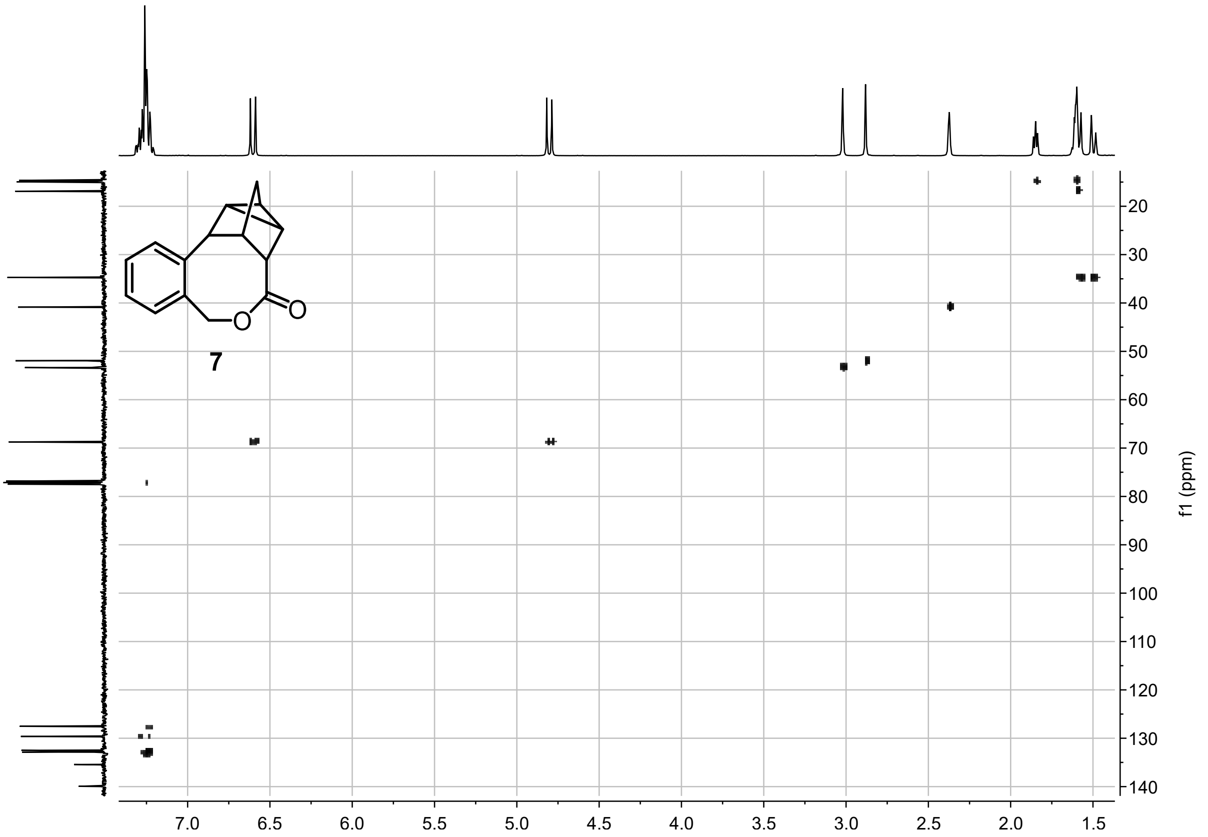

Figure S 239. $^{1}H,^{13}C$-HSQC of **7** in $CDCl_3$ measured at ^{1}H: 400.16 MHz; ^{13}C: 100.63 MHz.

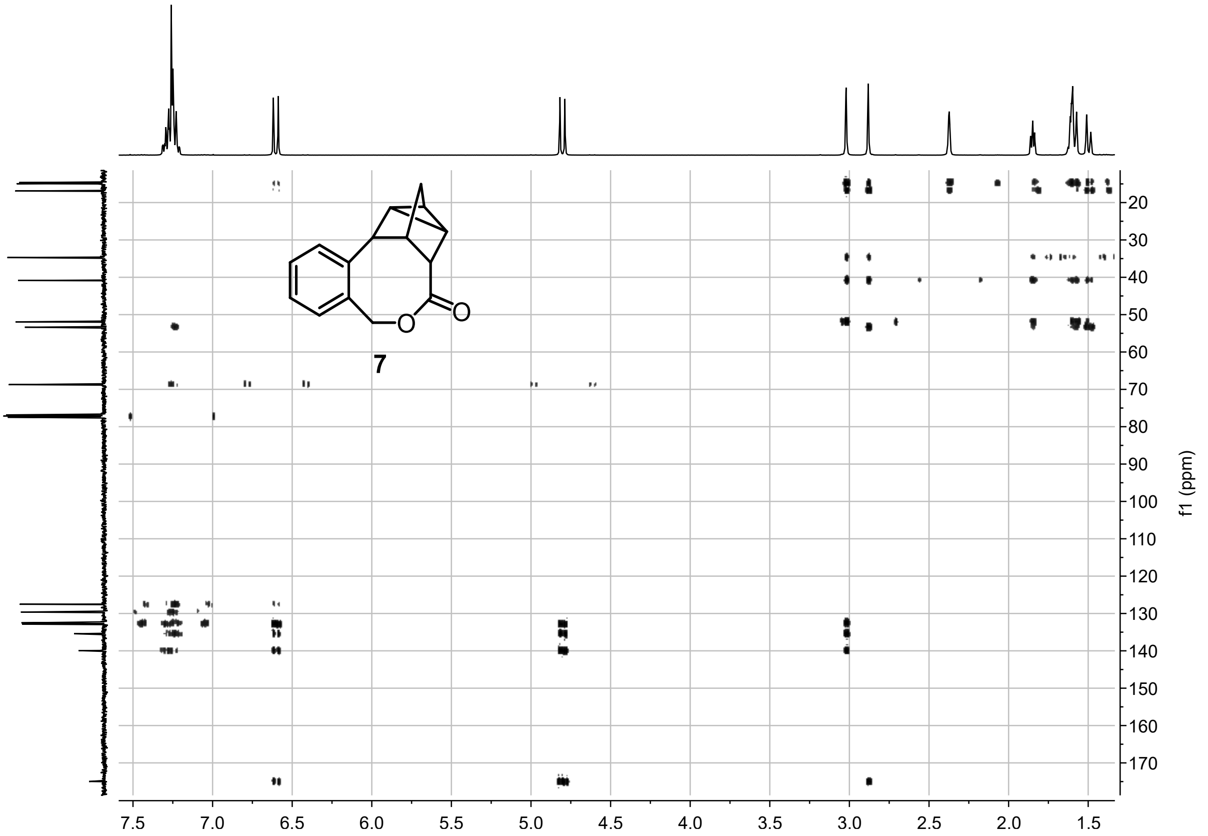

Figure S 240. $^{1}H,^{13}C$-HMBC of **7** in $CDCl_3$ measured at ^{1}H: 400.16 MHz; ^{13}C: 100.63 MHz.

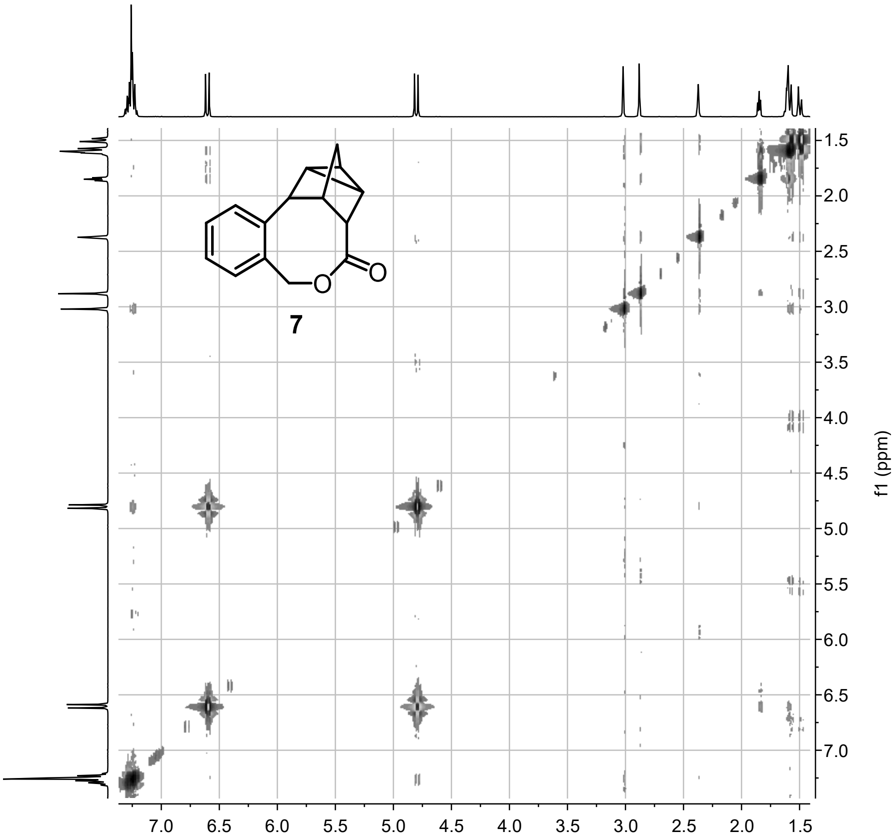

Figure S 241. $^{1}H,^{1}H$-NOESY of **7** in $CDCl_3$ measured at 400.16 MHz.

5 ^{31}P NMR studies

To gain insight into the mechanism of the Ni catalyzed NBD insertion into **1a**, a ^{31}P NMR study was conducted. Hereby, ^{31}P NMR spectra were measured in DMI without locking or shimming the sample.

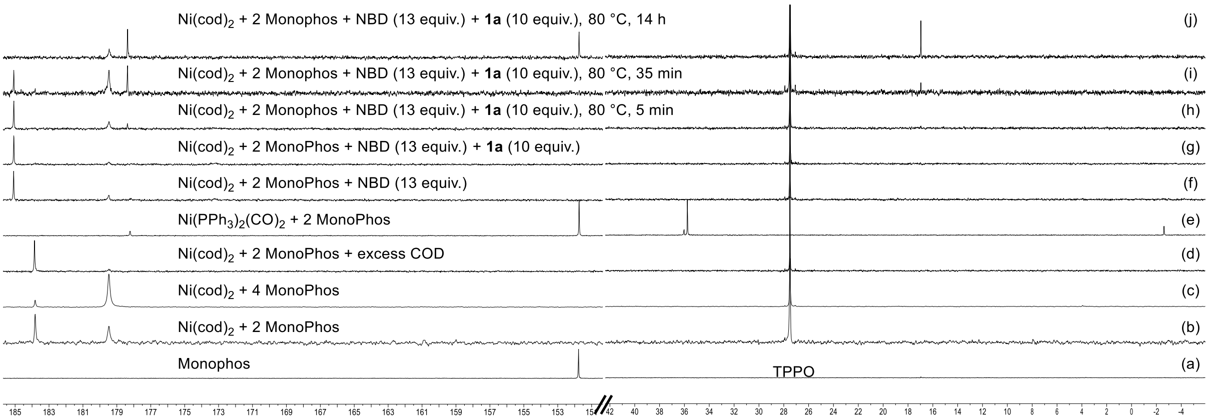

Figure S 242. Mechanistic ^{31}P NMR study of the Ni catalyzed NBD insertion into **1a**. All ^{31}P NMR spectra were measured in DMI without locking or shimming the sample at 300.13 MHz. To all reaction triphenylphosphine oxide (TPPO) was added as an internal standard.

In order to assign measured signals, reference spectra of MonoPhos and the employed catalyst consisting of 1 equiv. of $Ni(COD)_2$ and 2 equiv. of MonoPhos were recorded. Hereby, two distinct signals were observed (Figure S 242, b), which could be assigned to a Ni-species with most likely four MonoPhos ligands (Figure S 242, c) accompanied by a Ni-species with most likely two MonoPhos ligands and one COD ligand (Figure S 242, d), by adding an excess of MonPhos or COD respectively. Additionally, Ni(PPh3)2(CO)2 was mixed with 2 equiv. of MonoPhos giving rise to a signal corresponding to a Ni-carbonyl species with one or more MonoPhos ligands (Figure S 242, e).

To mimic the standard conditions, initially NBD (13 equiv.) was added. As expected, the signals corresponding to the Ni-species with most likely four MonoPhos ligands depleted, while the signal corresponding to the Ni-species with most likely two MonoPhos ligands and one COD ligand was shifted, as COD was replaced by NBD. Upon addition of **1a** (10 equiv.)

6 Kinetic measurements

To gain insight into the reaction kinetics, preliminary experiments were conducted at 50 °C and 80 °C.

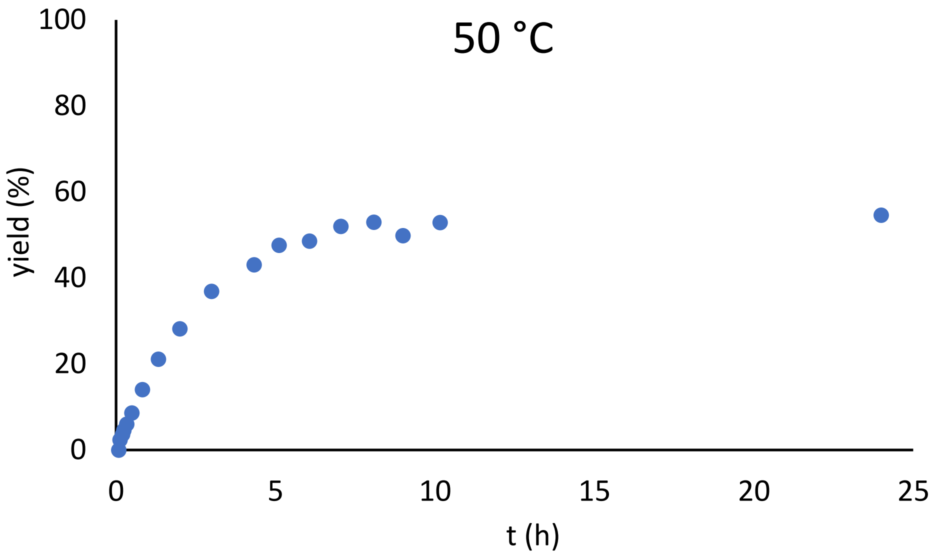

Figure S 243. GC-FID kinetic measurement of the Ni catalyzed NBD insertion into **1a** at 50 °C.

At 50 °C, the reaction achieved only ~50% yield after 10 h (Figure S 243), which was unexpected given the isolated yield of 90% under these conditions. However, the reproducibility of the kinetic measurements suggests that sampling during the reaction adversely affects the outcome. Furthermore, an induction period of approximately 10 min was observed at 50 °C.

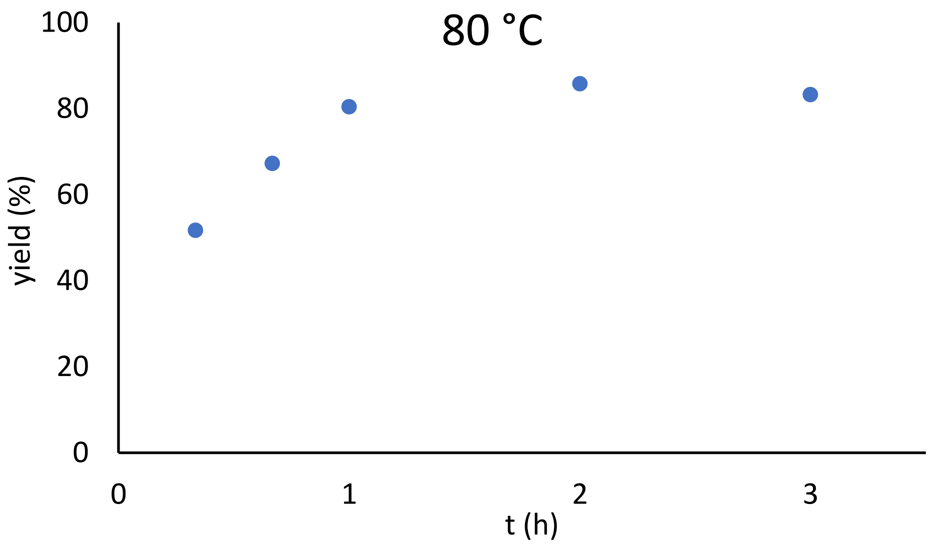

Figure S 244. GC-FID kinetic measurement of the Ni catalyzed NBD insertion into **1a** at 80 °C.

In contrast, the reaction at 80 °C proceeded significantly faster, reaching completion within approximately 2 h. Notably, the overall yield was not affected during this experiment, likely due to the reduced frequency of sampling.

7 Crystal structures

Crystals for X-ray structure analyses, were crystallized from *n*-hexane/EA unless denoted otherwise by slow evaporation of the solvent. Data collection was conducted on a Rigaku XtaLAB Synergy-S single-crystal X-ray diffractometer equipped with HyPix-6000HE detector and monochromated Cu-K_{α} radiation (λ=1.54184 Å) at 100 K. Absorption corrections for X-ray intensities were executed through a numerical method employing CrysAlisPro 1.171.42.49 (Rigaku Oxford Diffraction, 2022). The structure was solved by direct methods (SHELXS),[26] followed by full-matrix least-squares structure refinements, performed with SHELXL-2018,[27] implemented in Olex2 1.5.[28] The graphical representations were generated utilizing CCDC Mercury 2022.2.0.[29] Additional details pertaining to refinement procedures and crystallographic data for each crystal structure are listed below and in their respective CIF file.

***rel*-(1*S*,2*S*,3a*R*,4*R*,12a*S*,13*R*)-2,3,3a,4,11,12a-hexahydro-1,2,4-(epimethanetriyl) naphtho[2,3-f]azulen-12(1H)-one (3ba)**

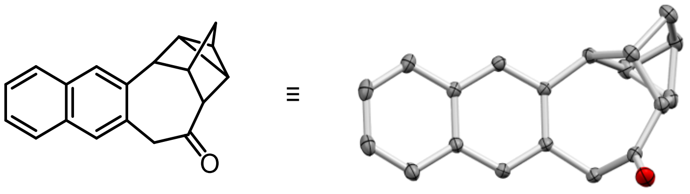

Figure S 245. Crystal structure of **3ba**. Hydrogen atoms are omitted for clarity and the thermal ellipsoids are drawn at 50% probability level.

Table S 7. Crystal data and structure refinement for **3ba**.

**Compound**	**3ba**		
Formula	$C_{19}H_{16}O$	Z'	1
CCDC	2310325	Wavelength/Å	1.54184
$D_{calc.}$/ g cm^{-3}	1.331	Radiation type	Cu K$_{\alpha}$
μ/mm^{-1}	0.623	Θ_{min}/°	4.40
Formula Weight	260.338	Θ_{max}/°	79.61
Colour	clear colourless	Measured Refl's.	13687
Shape	plate-shaped	Indep't Refl's	2759
Size/mm^3	0.14×0.13×0.06	Refl's I≥2 *s*(I)	2618
T/K	149.99(10)	R_{int}	0.0145
Crystal System	monoclinic	Parameters	326
Space Group	$P2_1/c$	Restraints	0
a/Å	10.5666(3)	Largest Peak	0.0855
b/Å	16.0194(3)	Deepest Hole	-0.0781
c/Å	8.0757(2)	GooF	1.2241
α/°	90	wR_2 (all data)	0.0257
β/°	108.119(3)	wR_2	0.0255
γ/°	90	R_1 (all data)	0.0119
V/Å^3	1299.19(6)	R_1	0.0108
Z	4		

rel-5-fluoro-(1*S*,2*S*,3a*R*,4*R*,10a*S*,11*R*)-2,3,3a,4,9,10a-hexahydro-1,2,4-(epimethanetriyl) benzo[f]azulen-10(1H)-one (3fa)

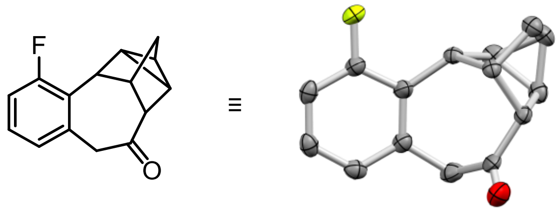

Figure S 246. Crystal structure of **3fa**. Hydrogen atoms are omitted for clarity and the thermal ellipsoids are drawn at 50% probability level.

Table S 8. Crystal data and structure refinement for **3fa**.

**Compound**	**3fa**		
Formula	$C_{15}H_{13}FO$	*Z*	4
CCDC	2376680	*Z'*	2
$D_{calc.}$/ g cm^{-3}	1.409	Wavelength/Å	1.54184
μ/mm^{-1}	0.802	Radiation type	Cu K□
Formula Weight	228.25	Θ_{min}/°	5.253
Colour	clear colourless	Θ_{max}/°	78.844
Shape	plate-shaped	Measured Refl's.	25937
Size/mm^3	0.16×0.10×0.05	Indep't Refl's	4360
T/K	149.99(10)	Refl's I≥2 □(I)	4272
Crystal System	monoclinic	R_{int}	0.0129
Flack Parameter	0.08(14)	Parameters	465
Hooft Parameter	-0.07(2)	Restraints	43
Space Group	$P2_1$	Largest Peak	0.194
a/Å	8.4337(2)	Deepest Hole	-0.163
b/Å	15.1543(2)	GooF	1.036
c/Å	9.2579(2)	wR_2 (all data)	0.0721
α/°	90	wR_2	0.0715
β/°	114.552(2)	R_1 (all data)	0.0265
γ/°	90	R_1	0.0259
V/Å^3	1076.24(4)		

***rel*-(1*R*,2*R*,3a*S*,4*S*,9*R*,10a*R*,11*S*)-9-methyl-2,3,3a,4,9,10a-hexahydro-1,2,4-(epimethanetriyl)benzo[f]azulen-10(1H)-one (mixture of enantiomers) (3ga)**

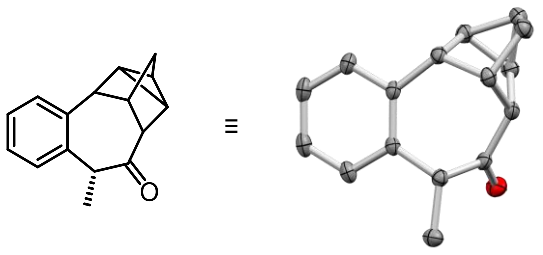

Figure S 247. Crystal structure of **3ga**. Hydrogen atoms are omitted for clarity and the thermal ellipsoids are drawn at 50% probability level.

Table S 9. Crystal data and structure refinement for **3ga**.

**Compound**	**3ga**		
Formula	$C_{16}H_{16}O$	*Z'*	2
CCDC	2378469	Wavelength/Å	1.54184
$D_{calc.}$/ g cm^{-3}	1.312	Radiation type	Cu K□
μ/mm^{-1}	0.618	Θ_{min}/°	3.27
Formula Weight	224.305	Θ_{max}/°	79.74
Colour	clear colourless	Measured Refl's.	43228
Shape	plate-shaped	Indep't Refl's	4855
Size/mm^3	0.17×0.12×0.05	Refl's I≥2 *s*(I)	4550
T/K	150.00(10)	R_{int}	0.0175
Crystal System	triclinic	Parameters	596
Space Group	*P*-1	Restraints	0
a/Å	9.2433(3)	Largest Peak	0.1920
b/Å	9.3665(3)	Deepest Hole	-0.0984
c/Å	13.9650(5)	GooF	1.0867
α/°	78.015(3)	wR_2 (all data)	0.0407
β/°	79.186(3)	wR_2	0.0401
γ/°	75.965(3)	R_1 (all data)	0.0194
V/Å^3	1135.33(7)	R_1	0.0176
Z	4		

***rel*-(1*R*,2*R*,3a*S*,4*S*,9*R*,10a*R*,11*S*)-9-ethyl-2,3,3a,4,9,10a-hexahydro-1,2,4-(epimethanetriyl)benzo[f]azulen-10(1H)-one (mixture of enantiomers) (3ha)**

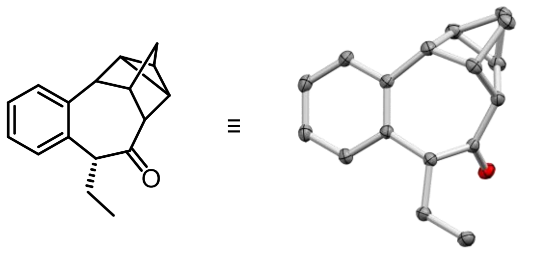

Figure S 248. Crystal structure of **3ha**. Hydrogen atoms are omitted for clarity and the thermal ellipsoids are drawn at 50% probability level.

Table S 10. Crystal data and structure refinement for **3ha**.

**Compound**	**3ha**		
Formula	$C_{17}H_{18}O$	*Z'*	1
CCDC	2375605	Wavelength/Å	1.54184
$D_{calc.}$/ g cm^{-3}	1.275	Radiation type	Cu K□
μ/mm^{-1}	0.594	Θ_{min}/°	4.84
Formula Weight	238.332	Θ_{max}/°	79.74
Colour	clear colourless	Measured Refl's.	22390
Shape	block-shaped	Indep't Refl's	2643
Size/mm^3	0.22×0.17×0.07	Refl's I≥2 *s*(I)	2494
T/K	149.99(10)	R_{int}	0.0160
Crystal System	triclinic	Parameters	326
Space Group	*P*-1	Restraints	0
a/Å	8.2890(4)	Largest Peak	0.2601
b/Å	8.9537(4)	Deepest Hole	-0.1055
c/Å	9.8110(3)	GooF	1.6571
α/°	76.935(3)	wR_2 (all data)	0.0424
β/°	69.386(4)	wR_2	0.0421
γ/°	66.263(4)	R_1 (all data)	0.0176
V/Å^3	620.84(5)	R_1	0.0163
Z	2		

***rel*-(1*R*,2*R*,3*R*,3a*S*,10a*S*,11*S*)-3-phenyl-2,3,3a,4,9,10a-hexahydro-1,2,4-(epimethanetriyl) benzo[f]azulen-10(1H)-one (3ad)**

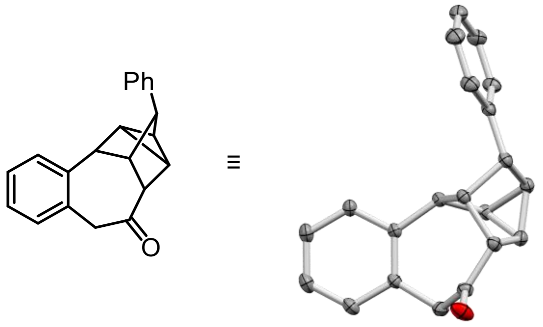

Figure S 249. Crystal structure of **3ad**. Hydrogen atoms are omitted for clarity and the thermal ellipsoids are drawn at 50% probability level.

Table S 11. Crystal data and structure refinement for **3ad**.

**Compound**	**3ad**		
Formula	$C_{21}H_{18}O$	*Z'*	1
CCDC	2374054	Wavelength/Å	1.54184
$D_{calc.}$/ g cm^{-3}	1.303	Radiation type	Cu K_{α}
μ/mm^{-1}	0.604	$\Theta_{min}/^{\circ}$	4.70
Formula Weight	286.376	$\Theta_{max}/^{\circ}$	79.84
Colour	clear colourless	Measured Refl's.	51022
Shape	block-shaped	Indep't Refl's	3177
Size/mm^3	0.23×0.21×0.13	Refl's I≥2 *s*(I)	3118
T/K	149.99(10)	R_{int}	0.0186
Crystal System	monoclinic	Parameters	362
Space Group	$P2_1/n$	Restraints	0
a/Å	11.1297(1)	Largest Peak	0.0763
b/Å	7.7608(1)	Deepest Hole	-0.0753
c/Å	16.9009(2)	GooF	1.2584
$\alpha/^{\circ}$	90	wR_2 (all data)	0.0291
$\beta/^{\circ}$	91.540(1)	wR_2	0.0289
$\gamma/^{\circ}$	90	R_1 (all data)	0.0127
V/Å^3	1459.29(3)	R_1	0.0123
Z	4		

rel-5-methyl-2,3,3a,4,9,10a-hexahydro-1,2,4-(epimethanetriyl)benzo[f]azulen-10(1H)-one (X)

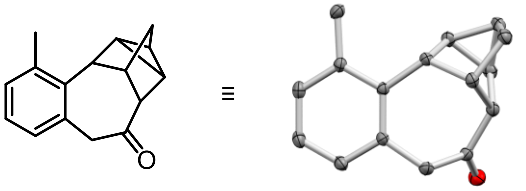

Figure S 250. Crystal structure of the regioisomer of **3ma**. Hydrogen atoms are omitted for clarity and the thermal ellipsoids are drawn at 50% probability level.

Table S 12. Crystal data and structure refinement for the regioisomer of **3ma**.

**Compound**	regioisomer of **3ma**		
Formula	$C_{16}H_{16}O$	*Z'*	1
CCDC	2383238	Wavelength/Å	1.54184
$D_{calc.}$/ g cm^{-3}	1.338	Radiation type	Cu K□
μ/mm^{-1}	0.630	Θ_{min}/°	5.59
Formula Weight	224.305	Θ_{max}/°	79.93
Colour	clear colourless	Measured Refl's.	40072
Shape	block-shaped	Indep't Refl's	2415
Size/mm^3	0.28×0.14×0.13	Refl's I≥2 *s*(I)	2368
T/K	150.00(10)	R_{int}	0.0307
Crystal System	monoclinic	Parameters	299
Space Group	$P2_1/c$	Restraints	0
a/Å	8.7741(2)	Largest Peak	0.1678
b/Å	15.5263(2)	Deepest Hole	-0.0718
c/Å	9.0616(2)	GooF	1.1718
α/°	90	wR_2 (all data)	0.0341
β/°	115.591(2)	wR_2	0.0339
γ/°	90	R_1 (all data)	0.0152
V/Å^3	1113.36(4)	R_1	0.0148
Z	4		

rel-(2S)-2',3',3a',4',9',10a'-hexahydro-1'H-spiro[oxirane-2,10'-[1,2,4](epimethanetriyl)benzo[f]azulene]

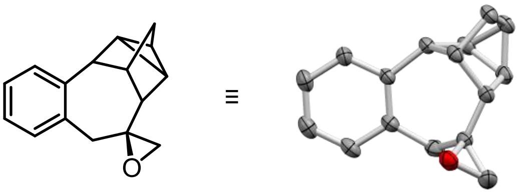

Figure S 251. Crystal structure of **5**. Hydrogen atoms are omitted for clarity and the thermal ellipsoids are drawn at 50% probability level.

Table S 13. Crystal data and structure refinement for **5**.

**Compound**	**5**		
Formula	$C_{16}H_{16}O$	*Z'*	1
CCDC	2412014	Wavelength/Å	1.54184
$D_{calc.}$/ g cm^{-3}	1.294	Radiation type	Cu K□
μ/mm^{-1}	0.609	$\Theta_{min}/^{\circ}$	5.00
Formula Weight	224.305	$\Theta_{max}/^{\circ}$	79.89
Colour	clear colourless	Measured Refl's.	17017
Shape	block-shaped	Indep't Refl's	2444
Size/mm^3	0.09×0.07×0.06	Refl's I≥2 *s*(I)	2369
T/K	150.01(19)	R_{int}	0.0275
Crystal System	monoclinic	Parameters	299
Space Group	-0.06(8)	Restraints	2
a/Å	-0.06(8)	Largest Peak	0.3031
b/Å	$P2_1$	Deepest Hole	-0.1671
c/Å	6.6983(4)	GooF	1.5325
$\alpha/^{\circ}$	9.7213(4)	wR_2 (all data)	0.0442
$\beta/^{\circ}$	9.4188(5)	wR_2	0.0440
$\gamma/^{\circ}$	90	R_1 (all data)	0.0197
V/Å^3	110.205(6)	R_1	0.0187
Z	90		

rel-2,3,3a,4,7,11b-hexahydro-1,2,4-(epimethanetriyl)benzo[c]cyclopenta[e]oxocin-5(1H)-one (7)

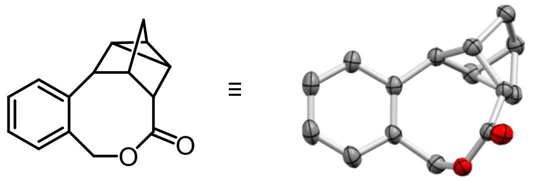

Figure S 252. Crystal structure of **7**. Hydrogen atoms are omitted for clarity and the thermal ellipsoids are drawn at 50% probability level.

Table S 14. Crystal data and structure refinement for **7**.

**Compound**	**7**		
Formula	$C_{15}H_{14}O_2$	*Z'*	1
CCDC	2409660	Wavelength/Å	1.54184
$D_{calc.}$/ g cm^{-3}	1.378	Radiation type	Cu K□
μ/mm^{-1}	0.721	$\Theta_{min}/^{\circ}$	5.16
Formula Weight	226.277	$\Theta_{max}/^{\circ}$	81.02
Colour	clear colourless	Measured Refl's.	38923
Shape	block-shaped	Indep't Refl's	2378
Size/mm^3	0.08×0.06×0.03	Refl's I≥2 *s*(I)	2125
T/K	150.01(18)	R_{int}	0.0339
Crystal System	monoclinic	Parameters	280
Space Group	*C*2/*c*	Restraints	0
a/Å	18.0665(12)	Largest Peak	0.2126
b/Å	8.2079(5)	Deepest Hole	-0.1295
c/Å	15.5222(11)	GooF	1.0650
$\alpha/^{\circ}$	90	wR_2 (all data)	0.0611
$\beta/^{\circ}$	108.558(7)	wR_2	0.0594
$\gamma/^{\circ}$	90	R_1 (all data)	0.0296
V/Å^3	2182.1(3)	R_1	0.0255
Z	8		

8 GC on chiral stationary phase

GC-MS spectra were measured on a 8890 GC and 5977B MSD system from Agilent, utilizing a chiral stationary phase: MEGA-DEX DET Beta (film thickness: 0.25 micron, Internal diameter: 0.25 mm; length: 25 m). All measurements were conducted under isocratic conditions with the respective temperature denoted under each measurement.

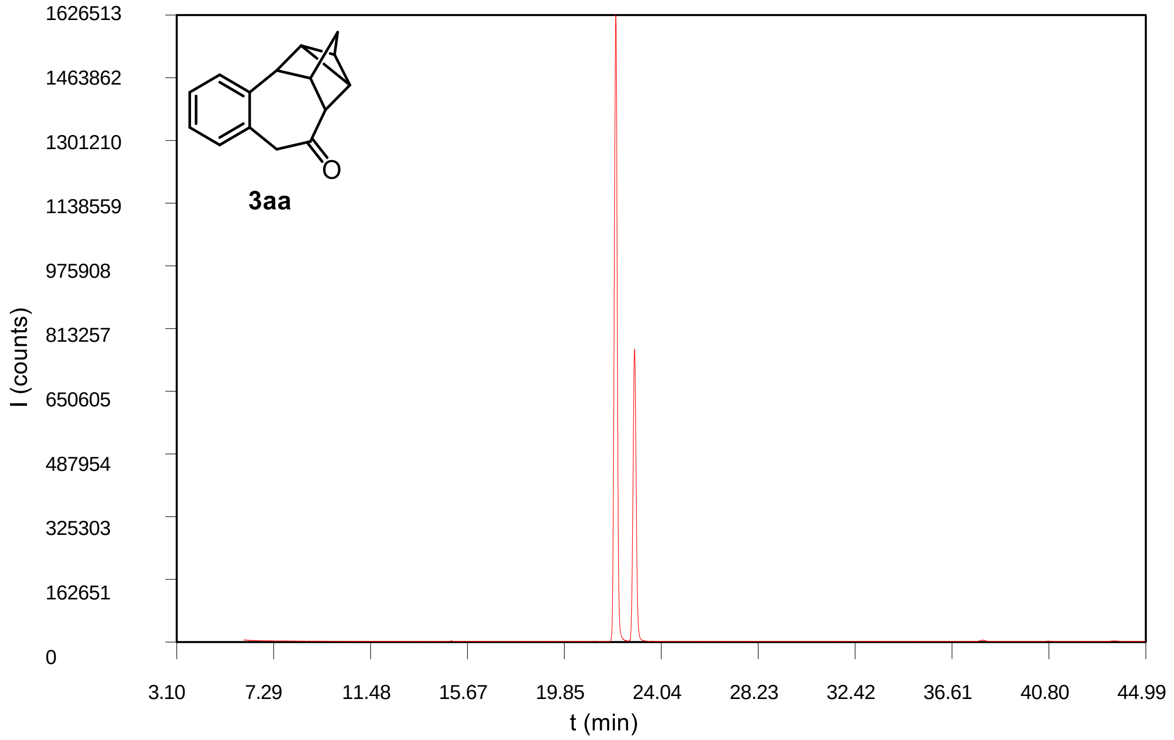

Figure S 253. GC-MS chromatogram measured with a chiral stationary phase of **3aa** (isocratic, 170 °C).

GC-MS (MEGA-DEX DET Beta, He, flow rate = 1.2 mL/min, EI) t_R = 20.39 min (**3aa**, 1); t_R = 21.26 min (**3aa**, 2).

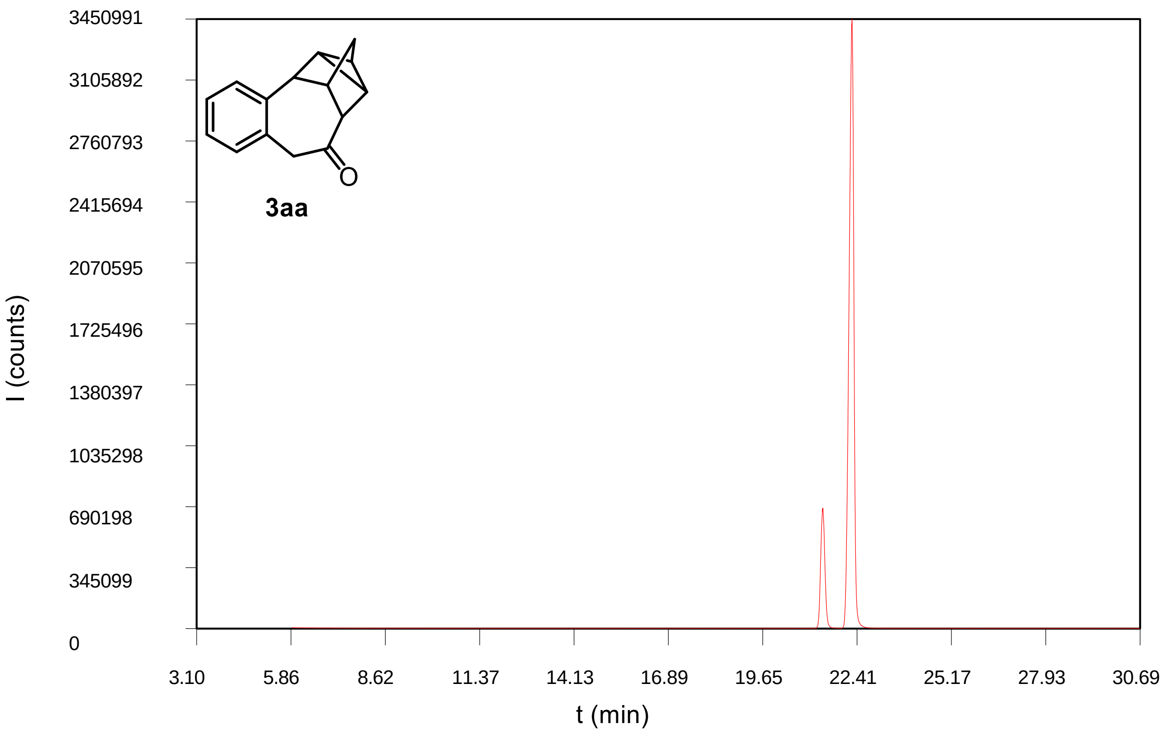

Figure S 254. GC-MS chromatogram measured with a chiral stationary phase of **3aa** (isocratic, 170 °C).

GC-MS (MEGA-DEX DET Beta, He, flow rate = 1.2 mL/min, EI) t_R = 20.37 min (**3aa**, 1); t_R = 21.31 min (**3aa**, 2).

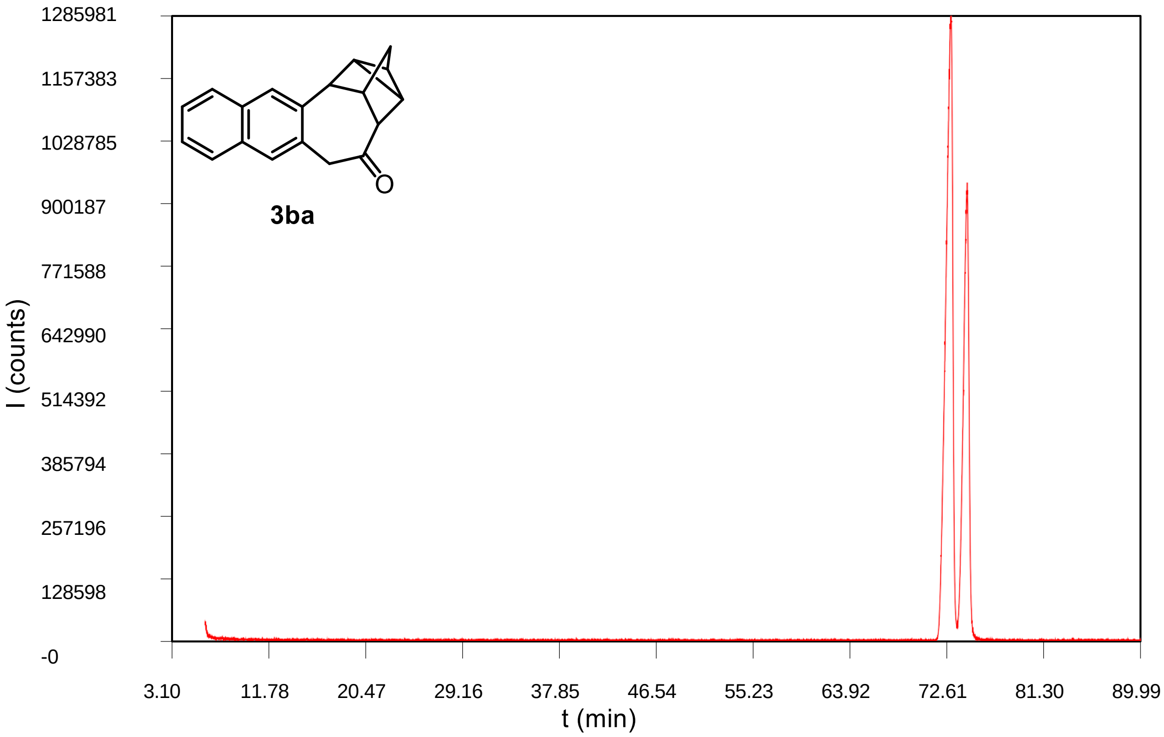

Figure S 255. GC-MS chromatogram measured with a chiral stationary phase of **3ba** (isocratic, 195 °C).

GC-MS (MEGA-DEX DET Beta, He, flow rate = 1.2 mL/min, EI) t_R = 72.36 min (**3ba**, 1); t_R = 73.87 min (**3ba**, 2).

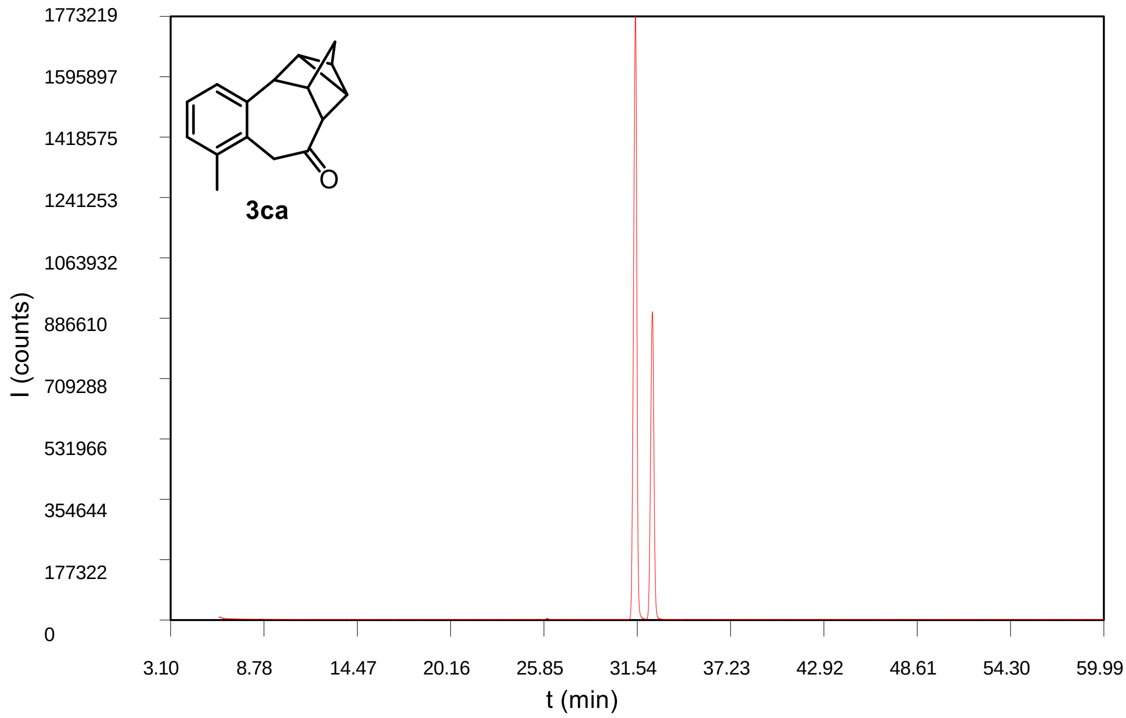

Figure S 256. GC-MS chromatogram measured with a chiral stationary phase of **3ca** (isocratic, 170 °C).

GC-MS (MEGA-DEX DET Beta, He, flow rate = 1.2 mL/min, EI) t_R = 29.88 min (**3ca**, 1); t_R = 30.97 min (**3ca**, 2).

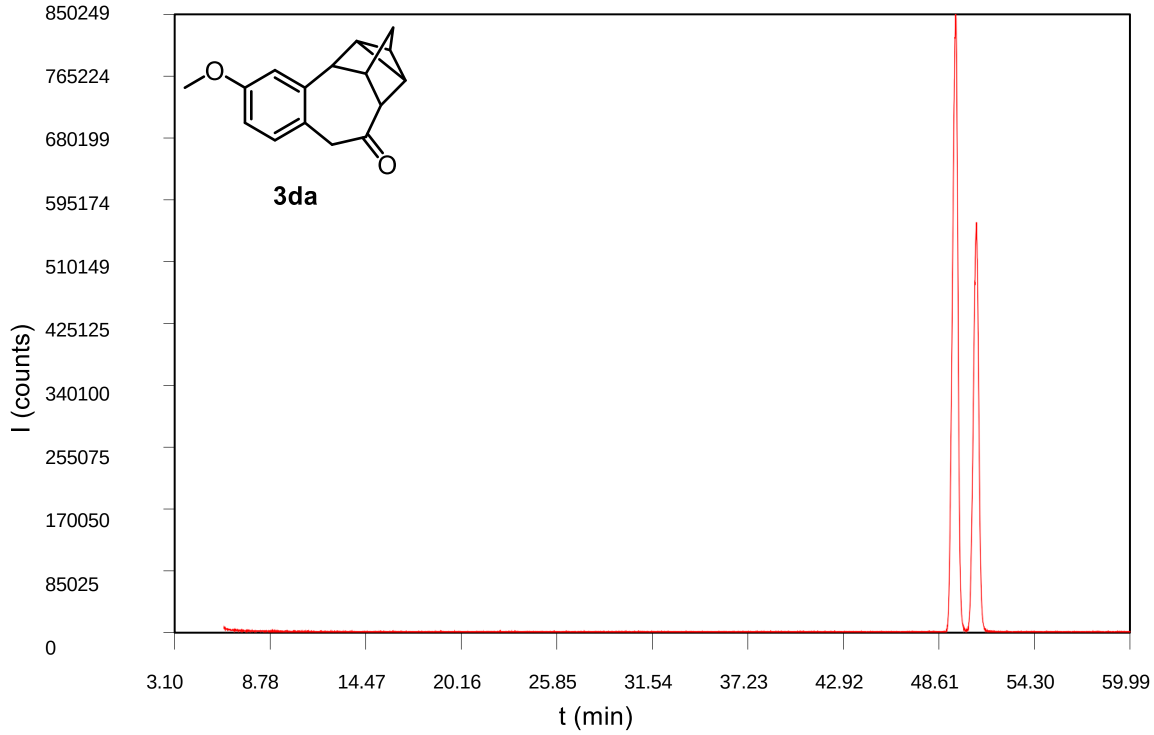

Figure S 257. GC-MS chromatogram measured with a chiral stationary phase of **3da** (isocratic, 175 °C).

GC-MS (MEGA-DEX DET Beta, He, flow rate = 1.2 mL/min, EI) t_R = 49.03 min (**3da**, 1); t_R = 50.33 min (**3da**, 2).

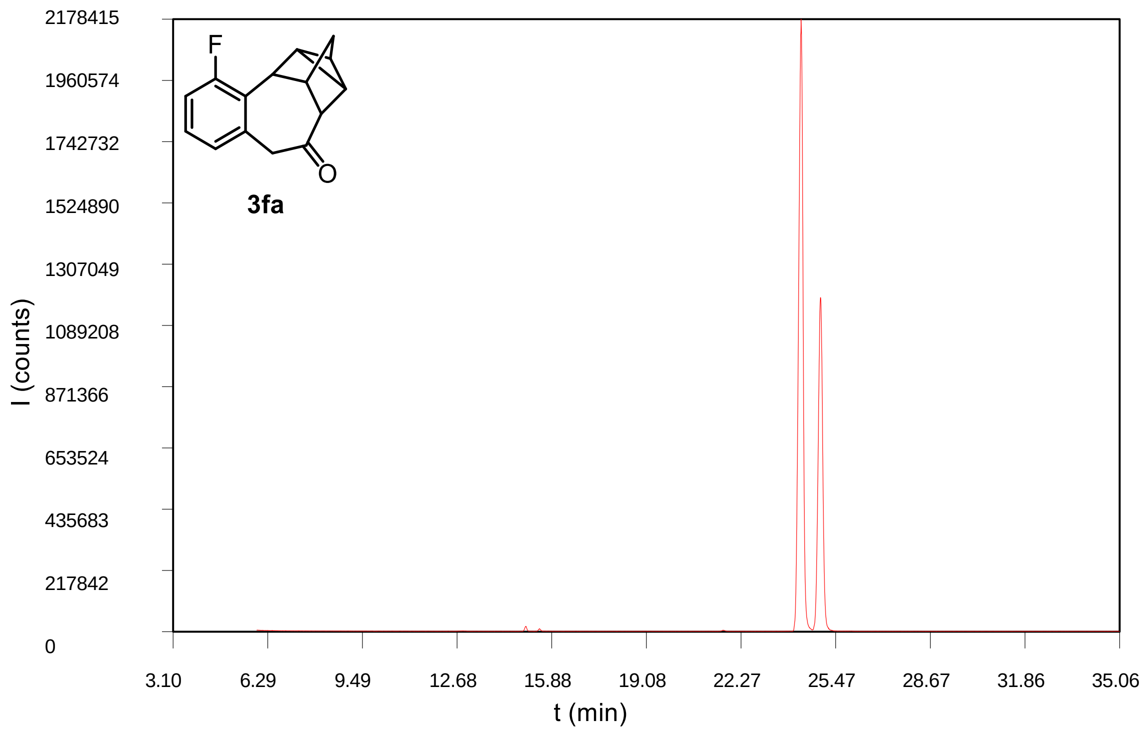

Figure S 258. GC-MS chromatogram measured with a chiral stationary phase of **3fa** (isocratic, 165 °C).

GC-MS (MEGA-DEX DET Beta, He, flow rate = 1.2 mL/min, EI) t_R = 23.26 min (**3fa**, 1); t_R = 23.98 min (**3fa**, 2).

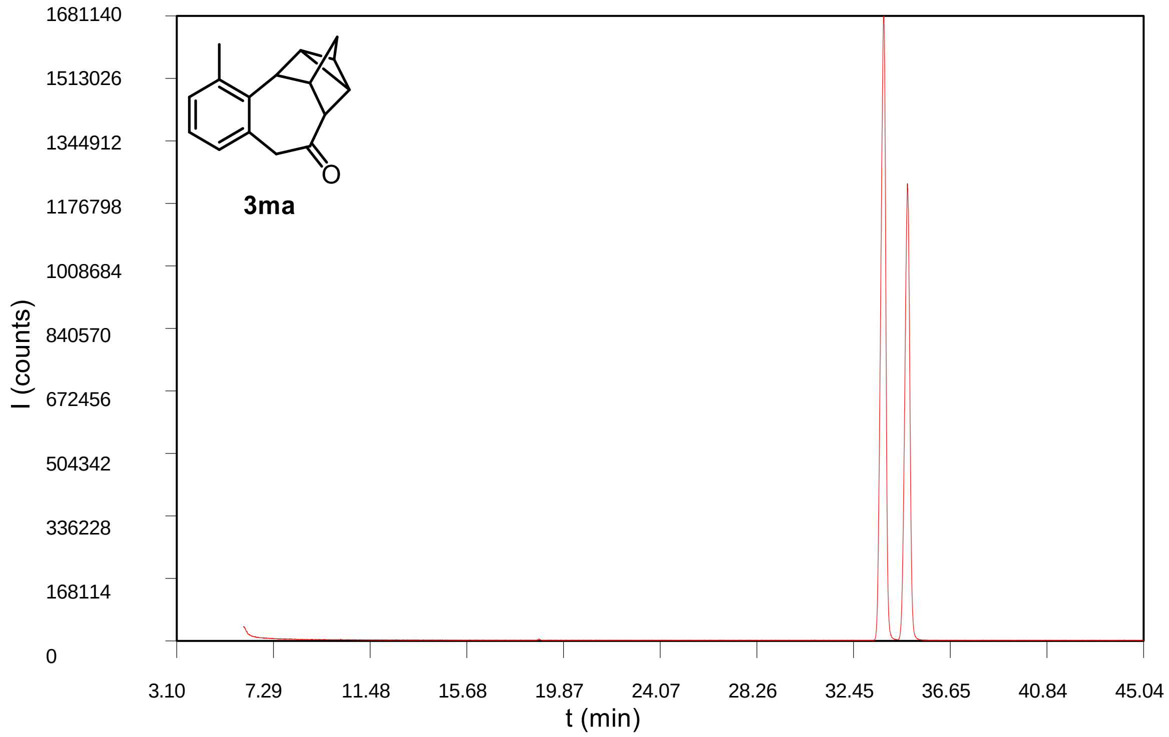

Figure S 259. GC-MS chromatogram measured with a chiral stationary phase of **3ma** (isocratic, 170 °C).

GC-MS (MEGA-DEX DET Beta, He, flow rate = 1.2 mL/min, EI) t_R = 32.93 min (**3ma**, 1); t_R = 34.04 min (**3ma**, 2).

9 GC-FID calibration

GC-FID yields were quantified by addition of *n*-pentadecane as an internal standard, applying the general equation:

$$\frac{A(compound)}{A(n\text{-}pentadecane)} = R \cdot \frac{m(compound)}{m(n\text{-}pentadecane)}$$

R: response factor
A: peak area determined by GC-FID
m: mass of compound

The response factors were determined by GC-FID calibration in EA.

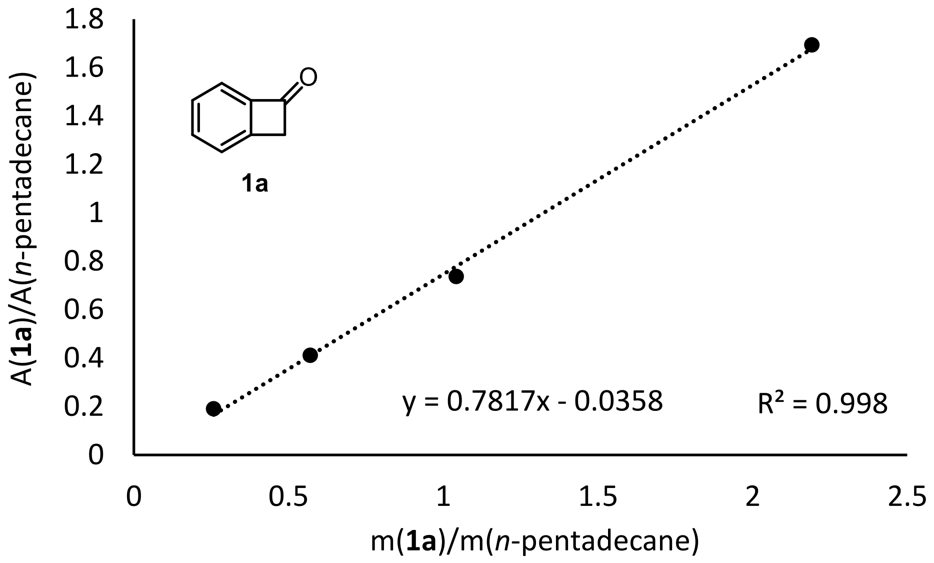

Figure S 260. GC-FID calibration of **1a** with *n*-pentadecane as an internal standard.

Table S 15. Values for the calibration of **1a** with *n*-pentadecane as an internal standard.

Entry	m(1a) (mg)	m(*n*-pentadecane) (mg)	A(1a)	A(*n*-pentadecane)
**1**	14.9	6.8	9419	5560
**2**	7.4	7.1	4445	6037
**3**	7.7	13.5	4575	11114
**4**	7.1	27.5	3354	17669

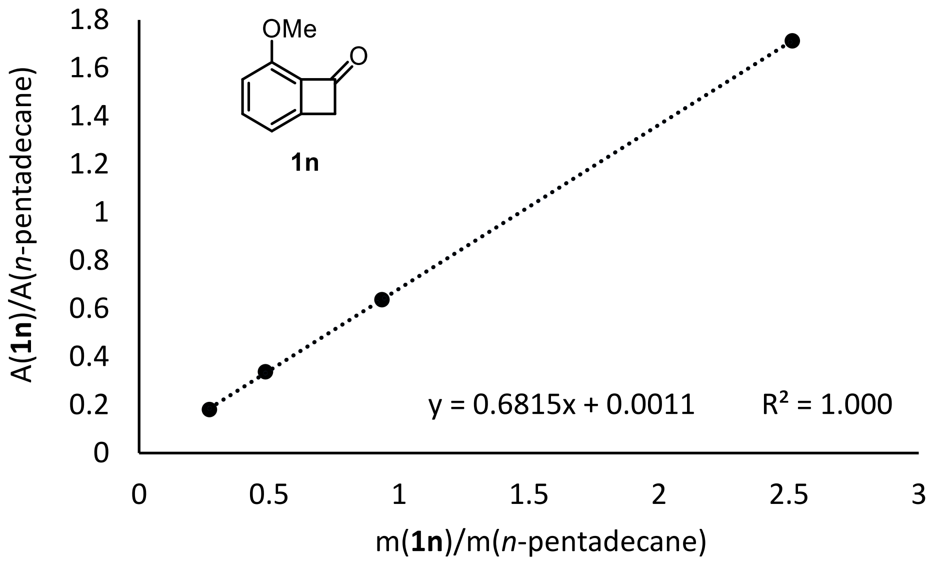

Figure S 261. GC-FID calibration of **1n** with *n*-pentadecane as an internal standard.

Table S 16. Values for the calibration of **1n** with *n*-pentadecane as an internal standard.

Entry	m(1n) (mg)	m(*n*-pentadecane) (mg)	A(1n)	A(*n*-pentadecane)
**1**	19.1	7.6	10923	6375
**2**	10.0	10.7	5248	8239
**3**	8.4	17.3	4564	13524
**4**	7.6	28.1	2848	15733

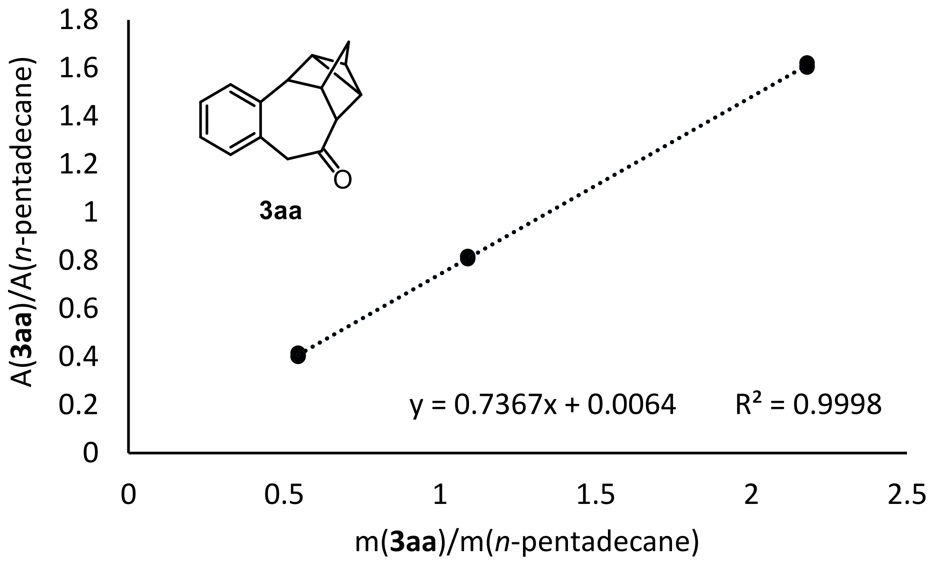

Figure S 262. GC-FID calibration of **3aa** with *n*-pentadecane as an internal standard.

Table S 17. Values for the calibration of **3aa** with *n*-pentadecane as an internal standard.

Entry	m(3aa) (mg)	m(*n*-pentadecane) (mg)	A(3aa)	A(*n*-pentadecane)
**1**	1.4	2.5	3118	7521
**2**	1.4	2.5	3100	7716
**3**	1.4	2.5	3084	7657
**4**	1.4	1.2	2983	3692
**5**	1.4	1.2	3214	3936
**6**	2.7	1.2	6112	3769
**7**	2.7	1.2	6095	3791
**8**	2.7	1.2	5759	3590

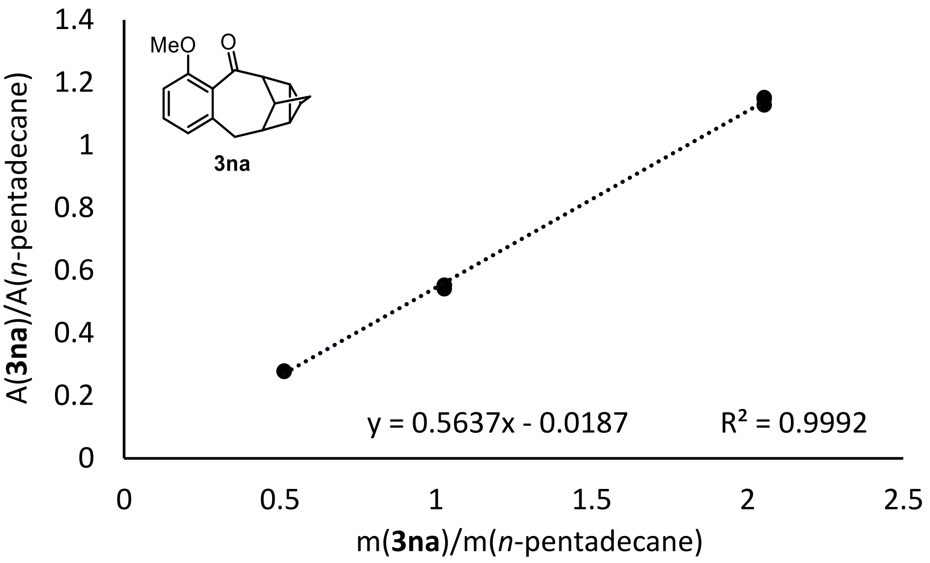

Figure S 263. GC-FID calibration of **3na** with *n*-pentadecane as an internal standard.

Table S 18. Values for the calibration of **3na** with *n*-pentadecane as an internal standard.

Entry	m(3na) (mg)	m(*n*-pentadecane) (mg)	A(3na)	A(*n*-pentadecane)
**1**	3.2	1.6	3722	3296
**2**	3.2	1.6	3723	3232
**3**	3.2	1.6	3751	3267
**4**	1.6	1.6	1839	3393
**5**	1.6	1.6	1854	3356
**6**	1.6	1.6	1837	3323
**7**	1.6	3.1	1889	6778
**8**	1.6	3.1	1865	6710
**9**	1.6	3.1	1862	6686

10 Literature

[1] J. A. Werra, K. Wurst, L. B. Wilm, P. Löwe, M. B. Röthel, F. Dielmann, *Organometallics* **2023**, *42*, 597-605.

[2] M. J. O'Neil, 14th ed., The Merck index : an encyclopedia of chemicals, drugs, and biologicals, Merck Whitehouse Station, N.J., **2006**.

[3] P.-h. Chen, J. Sieber, C. H. Senanayake, G. Dong, *Chem. Sci.* **2015**, *6*, 5440-5445.

[4] T.-S. Mei, D.-H. Wang, J.-Q. Yu, *Org. Lett.* **2010**, *12*, 3140-3143.

[5] J. A. Cadge, P. J. Gates, J. F. Bower, C. A. Russell, *J. Am. Chem. Soc.* **2022**, *144*, 19719-19725.

[6] R. Li, B. Li, H. Zhang, C.-W. Ju, Y. Qin, X.-S. Xue, D. Zhao, *Nat. Chem.* **2021**, *13*, 1006-1016.

[7] L. S. Liebeskind, L. J. Lescosky, C. M. McSwain, Jr., *J. Org. Chem.* **1989**, *54*, 1435-1439.

[8] P. R. Story, S. R. Fahrenholtz, *J. Org. Chem.* **1963**, *28*, 1716-1717.

[9] L. Liu, N. Ishida, M. Murakami, *Angew. Chem. Int. Ed.* **2012**, *51*, 2485-2488.

[10] M. Šámal, S. Chercheja, J. Rybáček, J. Vacek Chocholoušová, J. Vacek, L. Bednárová, D. Šaman, I. G. Stará, I. Starý, *J. Am. Chem. Soc.* **2015**, *137*, 8469-8474.

[11] H. Wang, C. Zhou, Z. Gao, S. Li, G. Li, *Angew. Chem. Int. Ed.* **2023**, *62*, e202300905.

[12] F. De Wael, G. G. Muccioli, D. M. Lambert, T. Sergent, Y.-J. Schneider, J.-F. Rees, J. Marchand-Brynaert, *European Journal of Medicinal Chemistry* **2010**, *45*, 3564-3574.

[13] A. Leclair, Q. Wang, J. Zhu, *ACS Catal.* **2022**, *12*, 1209-1215.

[14] T. Kobayashi, T. Hosoya, S. Yoshida, *J. Org. Chem.* **2020**, *85*, 4448-4462.

[15] K. Nishikawa, H. Fukuda, M. Abe, K. Nakanishi, Y. Tazawa, C. Yamaguchi, S. Hiradate, Y. Fujii, K. Okuda, M. Shindo, *Phytochemistry* **2013**, *96*, 223-234.

[16] T. Yano, T. Kawasaki, T. Yuhki, N. Ishida, M. Murakami, *Org. Lett.* **2018**, *20*, 1224-1227.

[17] R. V. Stevens, G. S. Bisacchi, *J. Org. Chem.* **1982**, *47*, 2393-2396.

[18] P. R. Story, *J. Org. Chem.* **1961**, *26*, 287-290.

[19] A. A. Nagarkar, M. Yasir, A. Crochet, K. M. Fromm, A. F. M. Kilbinger, *Angew. Chem. Int. Ed.* **2016**, *55*, 12343-12346.

[20] W. Maudez, C. Roy, P. D. Tran, C. Thurier, F. Karmous, P. Doppelt, *Chem. Vap. Deposition* **2014**, *20*, 59-68.

[21] H. D. Verkruijsse, L. Brandsma, *Recl. Trav. Chim. Pays-Bas* **1986**, *105*, 66-68.

[22] B. S. Pilgrim, D. A. Roberts, T. G. Lohr, T. K. Ronson, J. R. Nitschke, *Nat. Chem.* **2017**, *9*, 1276-1281.

[23] P. Liu, H. Dong, B. Gong, S. Gao, A. Lin, H. Yao, *Org. Lett.* **2024**, *26*, 8244-8248.

[24] J. A. Ciaccio, A. L. Drahus, R. M. Meis, C. T. Tingle, M. Smrtka, R. Geneste, *Synth. Commun.* **2003**, *33*, 2135-2143.

[25] J. Duan, Y.-F. Du, X. Pang, X.-Z. Shu, *Chem. Sci.* **2019**, *10*, 8706-8712.

[26] G. M. Sheldrick, *Acta Crystallogr., Sect. A* **2008**, *64*, 112-122.

[27] G. Sheldrick, *Acta Crystallogr., Sect. C* **2015**, *71*, 3-8.

[28] O. V. Dolomanov, L. J. Bourhis, R. J. Gildea, J. A. K. Howard, H. Puschmann, *J. Appl. Crystallogr.* **2009**, *42*, 339-341.

[29] C. F. Macrae, I. Sovago, S. J. Cottrell, P. T. A. Galek, P. McCabe, E. Pidcock, M. Platings, G. P. Shields, J. S. Stevens, M. Towler, P. A. Wood, *J. Appl. Crystallogr.* **2020**, *53*, 226-235.

11 IR-Spectra

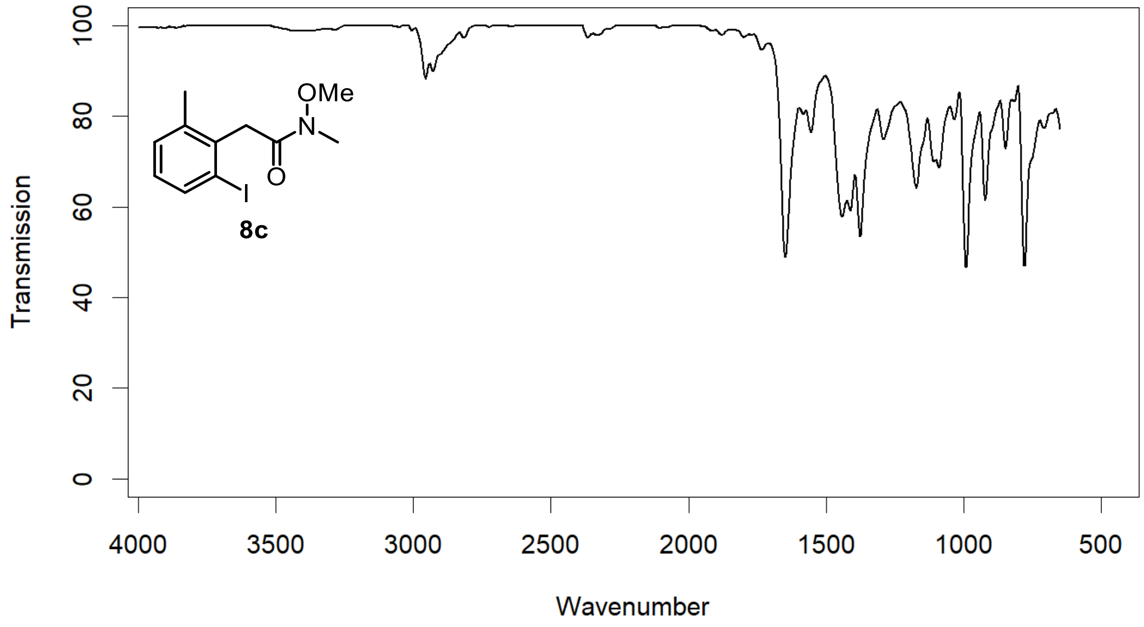

Figure S 264. IR spectrum of **8c**.

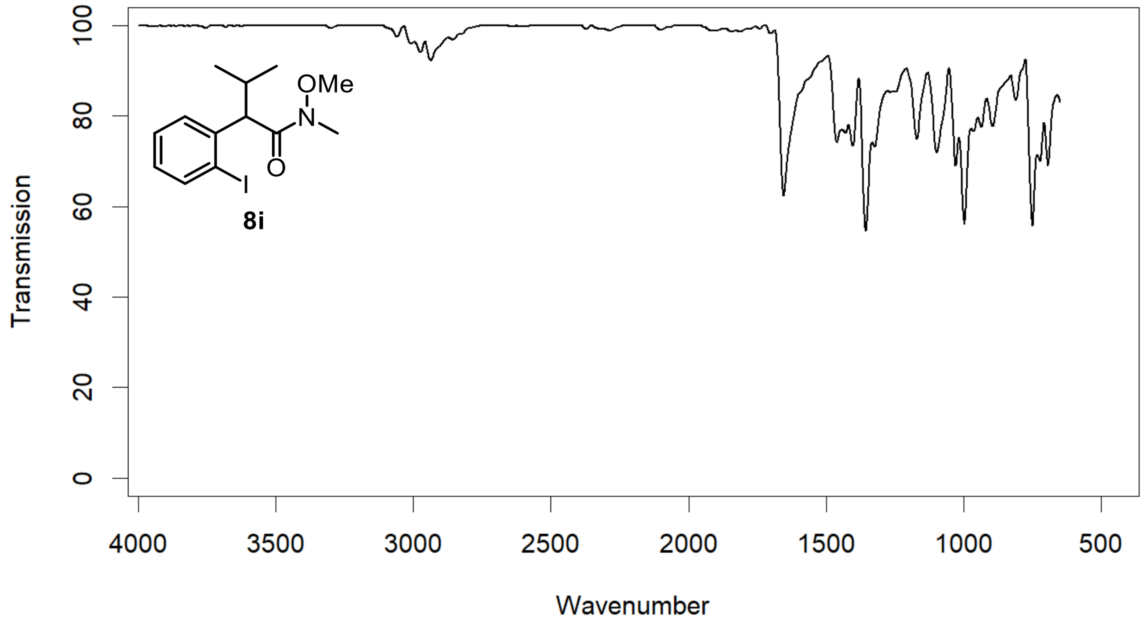

Figure S 265. IR spectrum of **8i**.

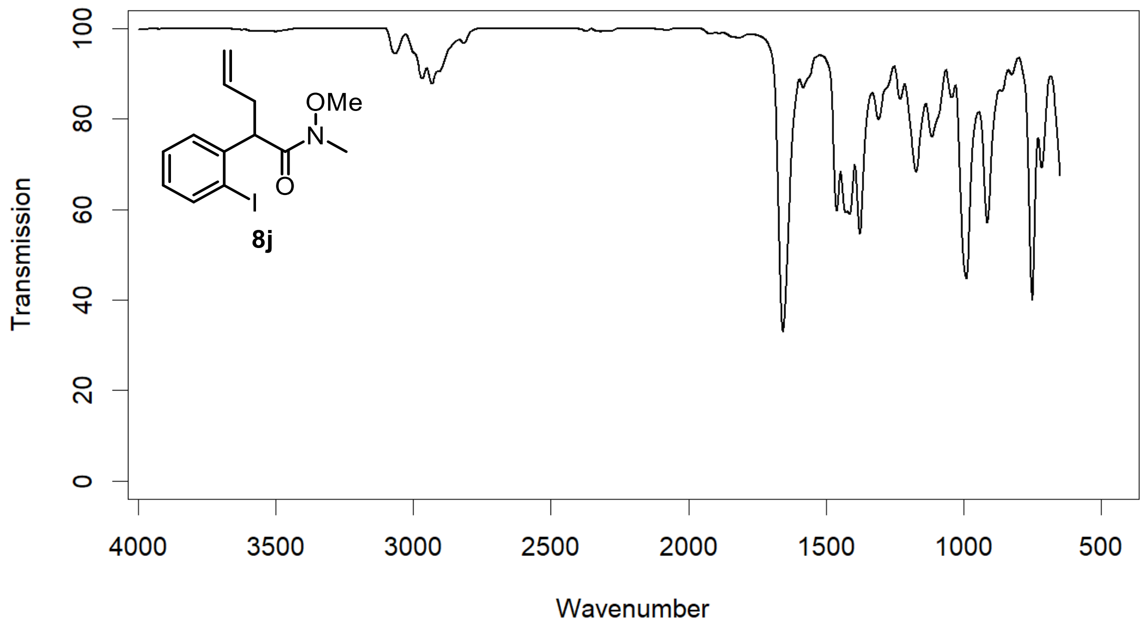

Figure S 266. IR spectrum of **8j**.

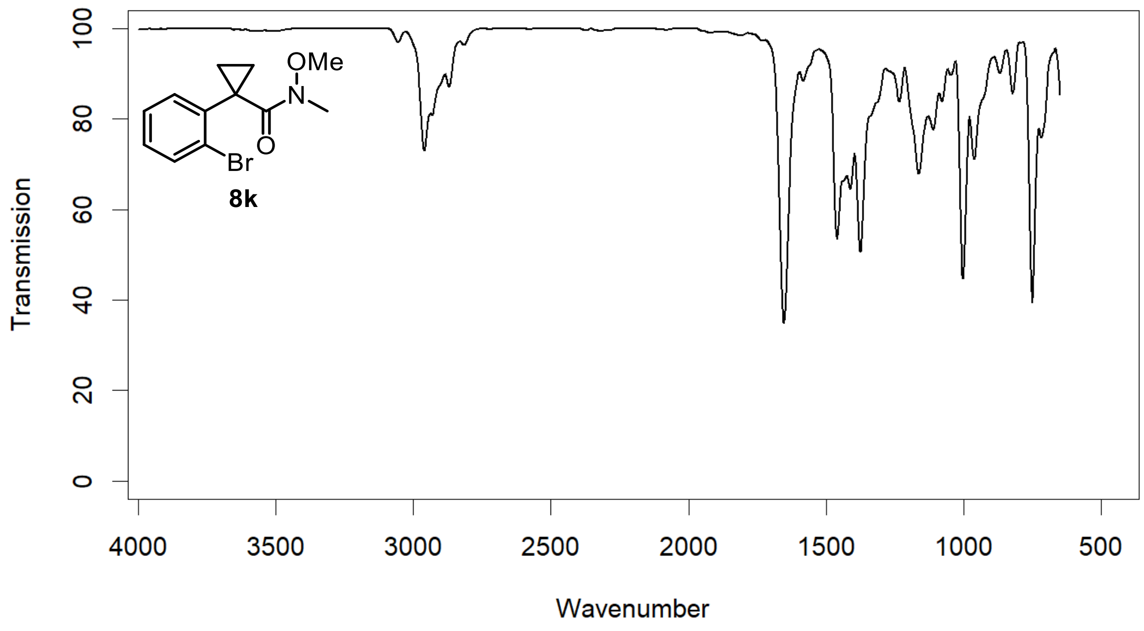

Figure S 267. IR spectrum of **8k**.

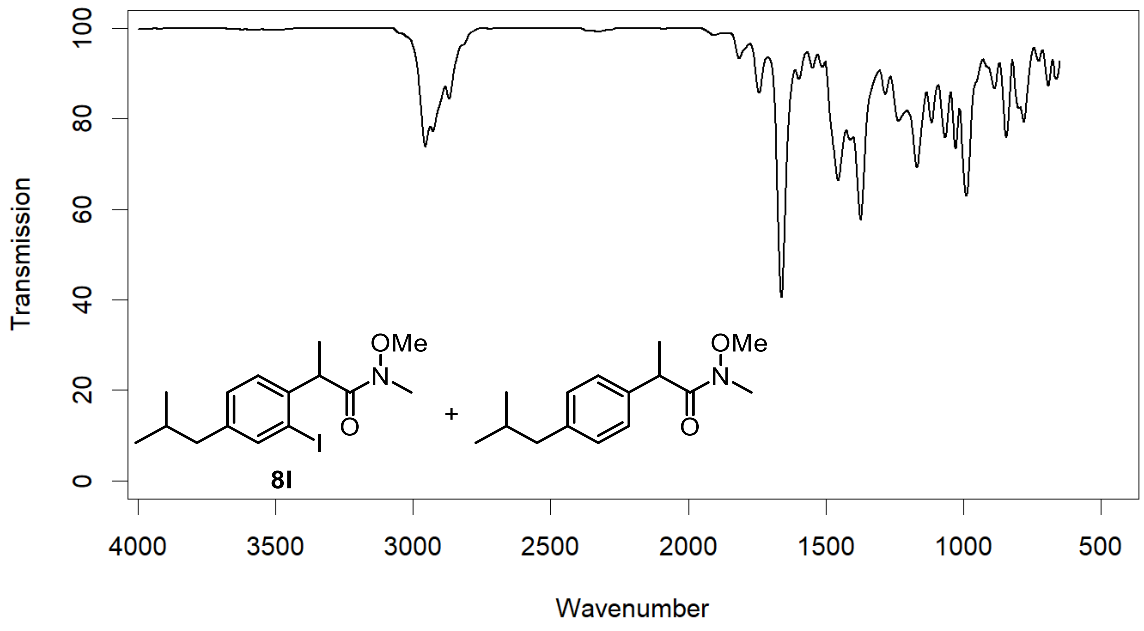

Figure S 268. IR spectrum of a mixture of **8l** and N-Methoxy-N-methyl-2-(4-isobutylphenyl)propenamide (78:22).

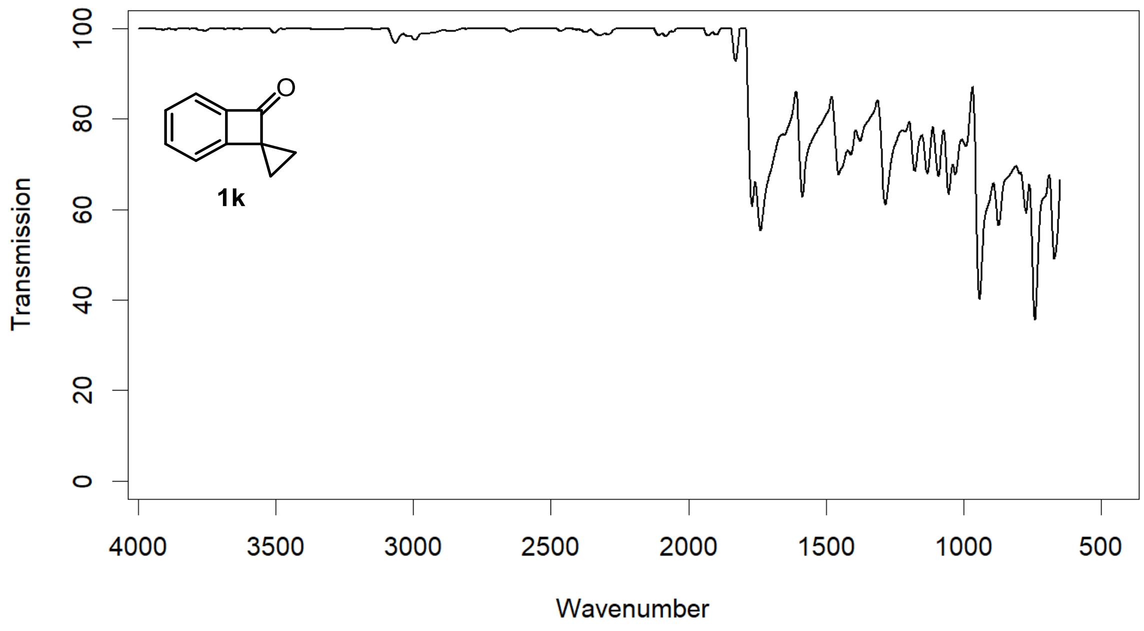

Figure S 269. IR spectrum of **1k**.

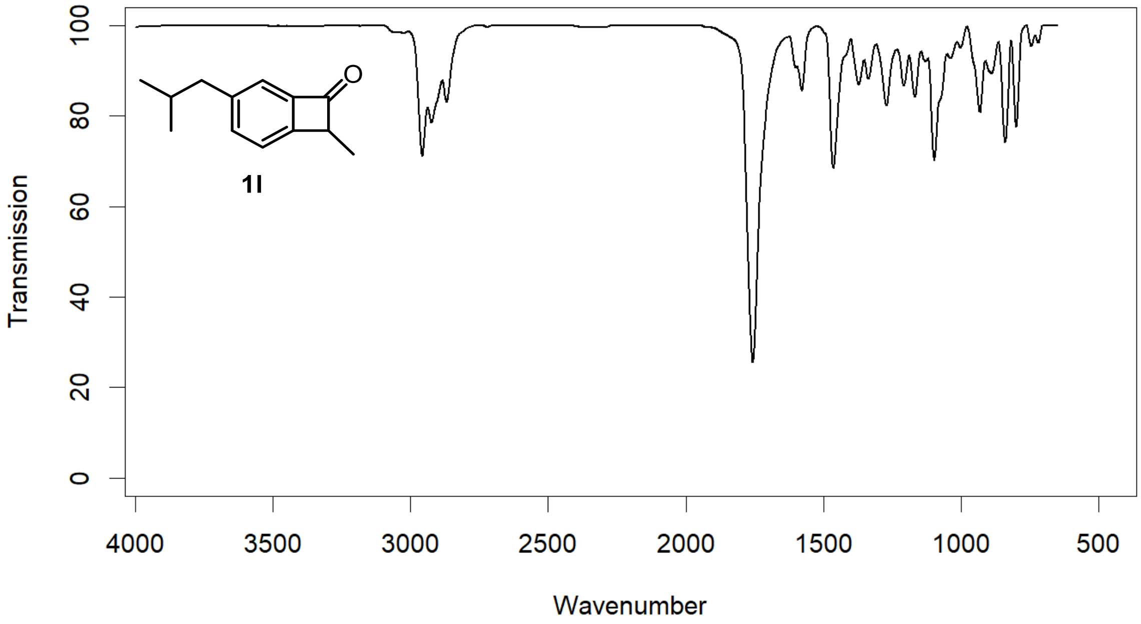

Figure S 270. IR spectrum of **1l**.

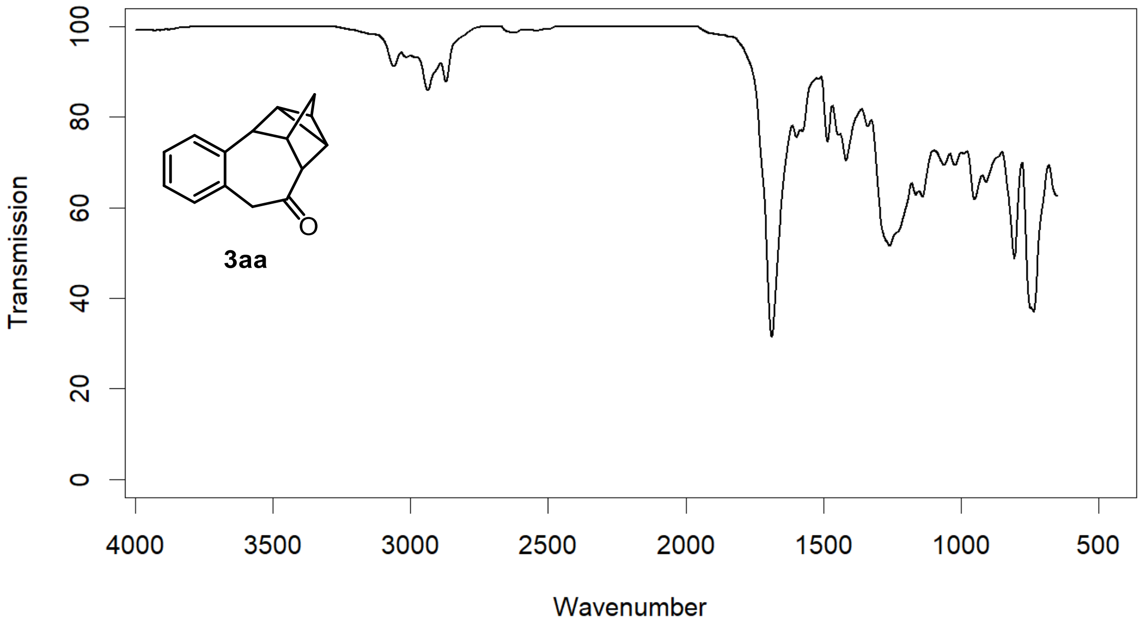

Figure S 271. IR spectrum of **3aa**.

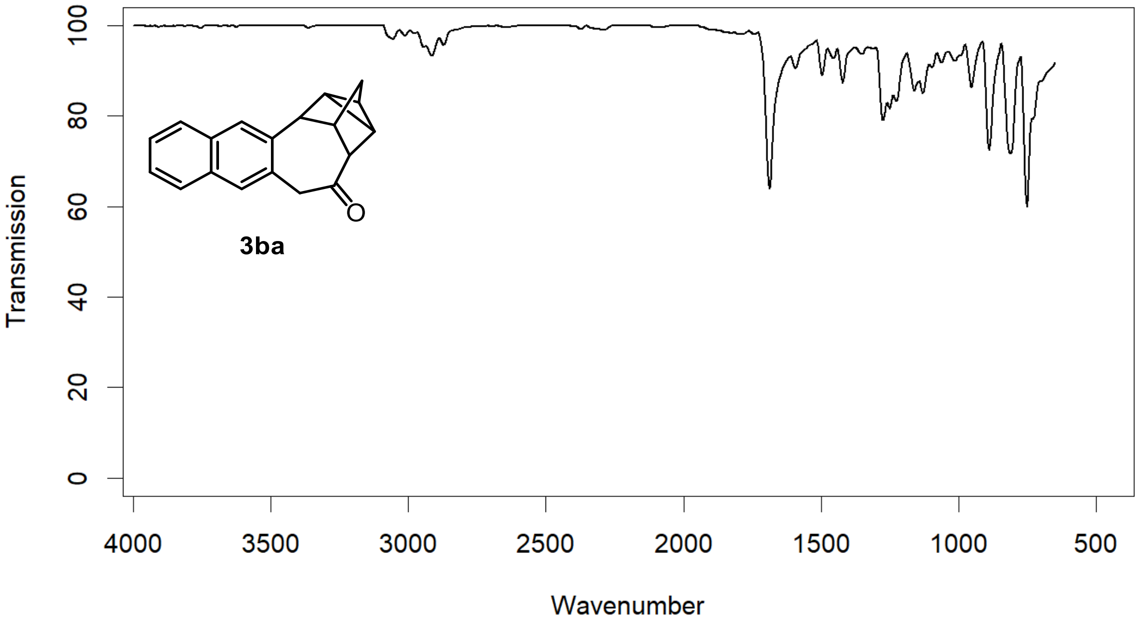

Figure S 272. IR spectrum of **3ba**.

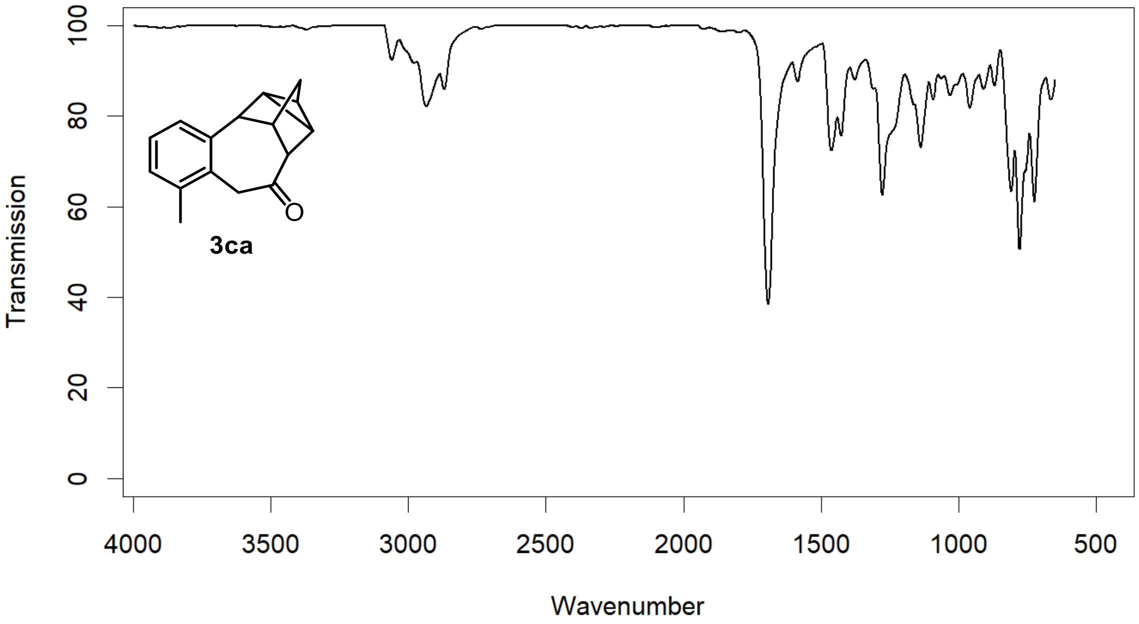

Figure S 273. IR spectrum of **3ca**.

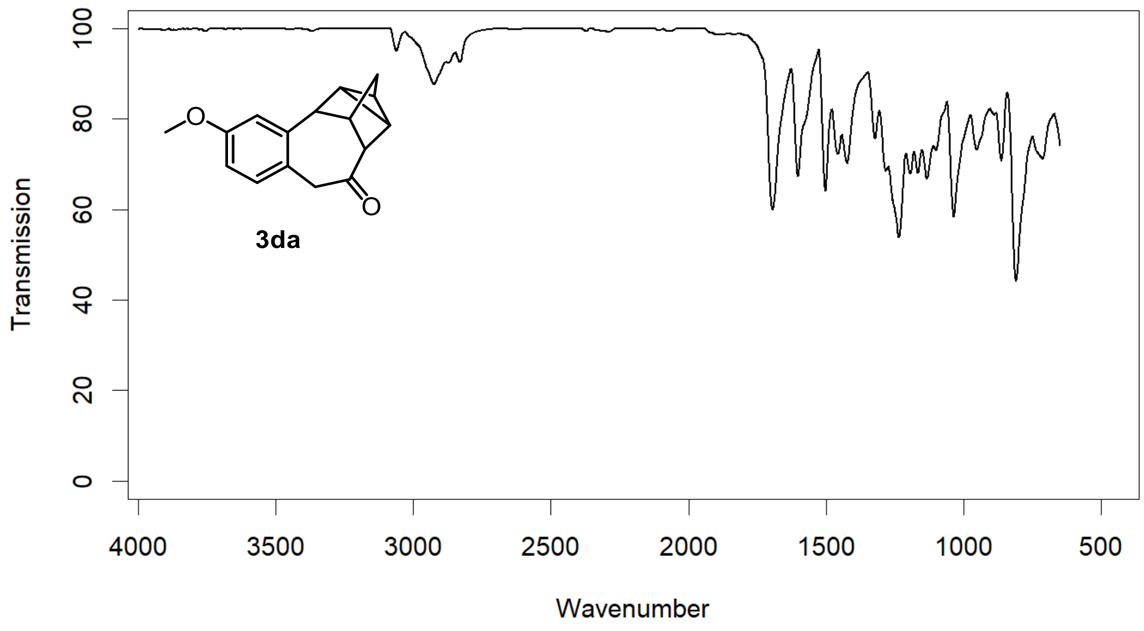

Figure S 274. IR spectrum of **3da**.

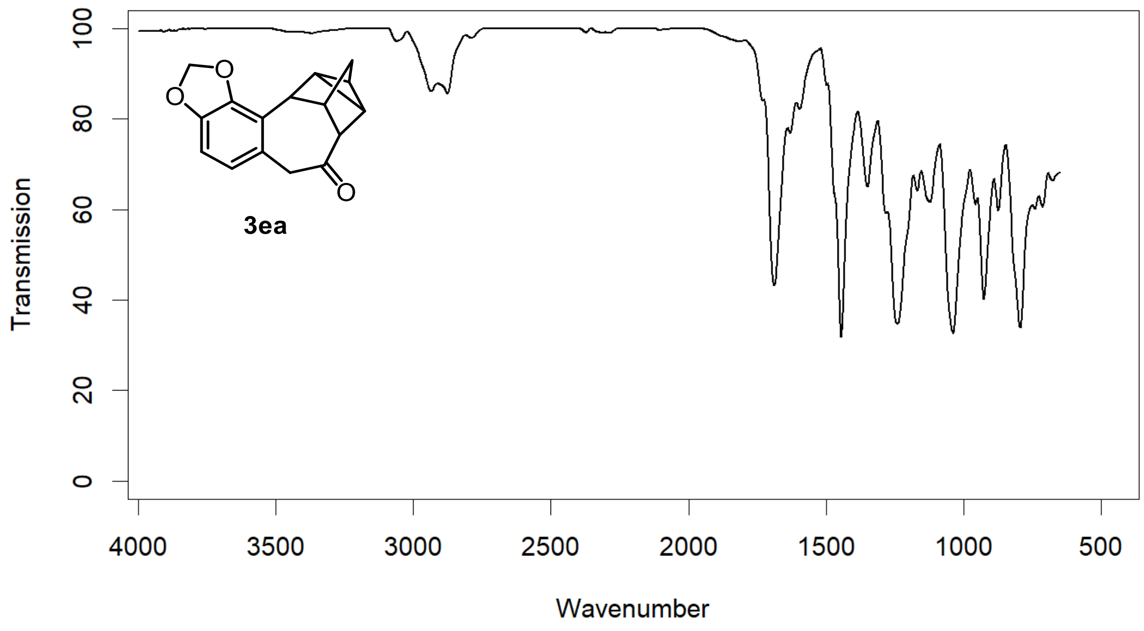

Figure S 275. IR spectrum of **3ea**.

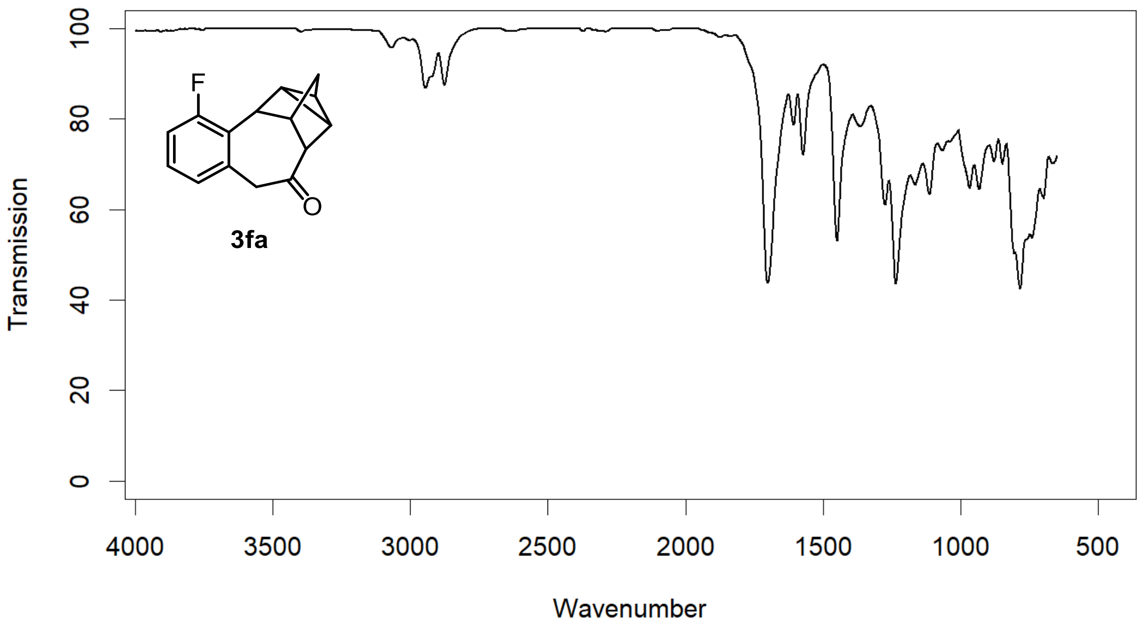

Figure S 276. IR spectrum of **3fa**.

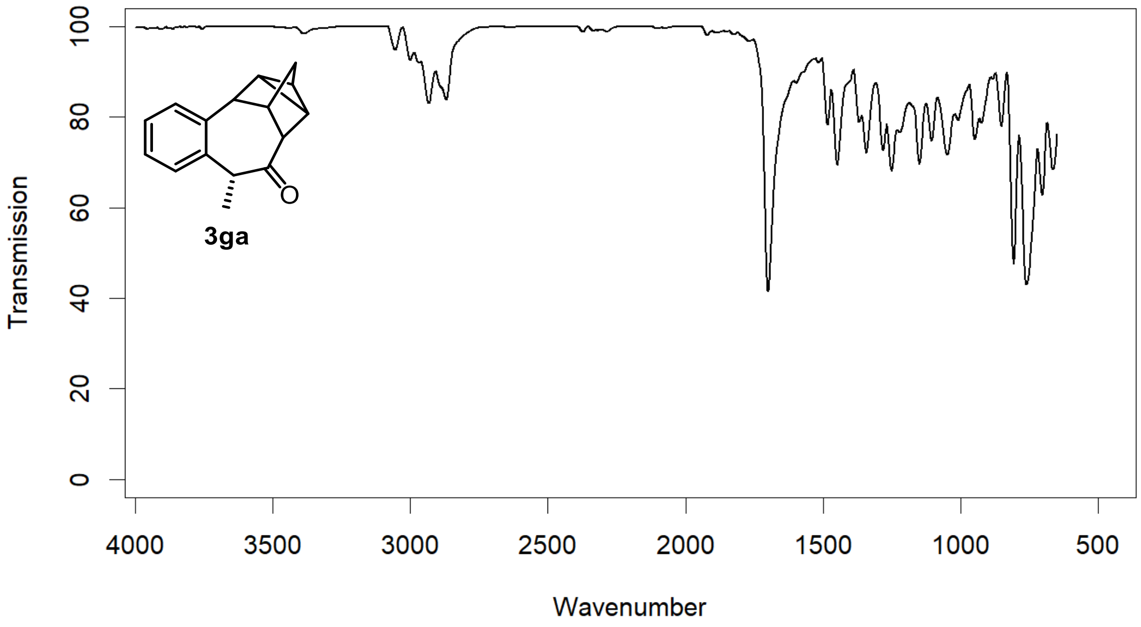

Figure S 277. IR spectrum of **3ga**.

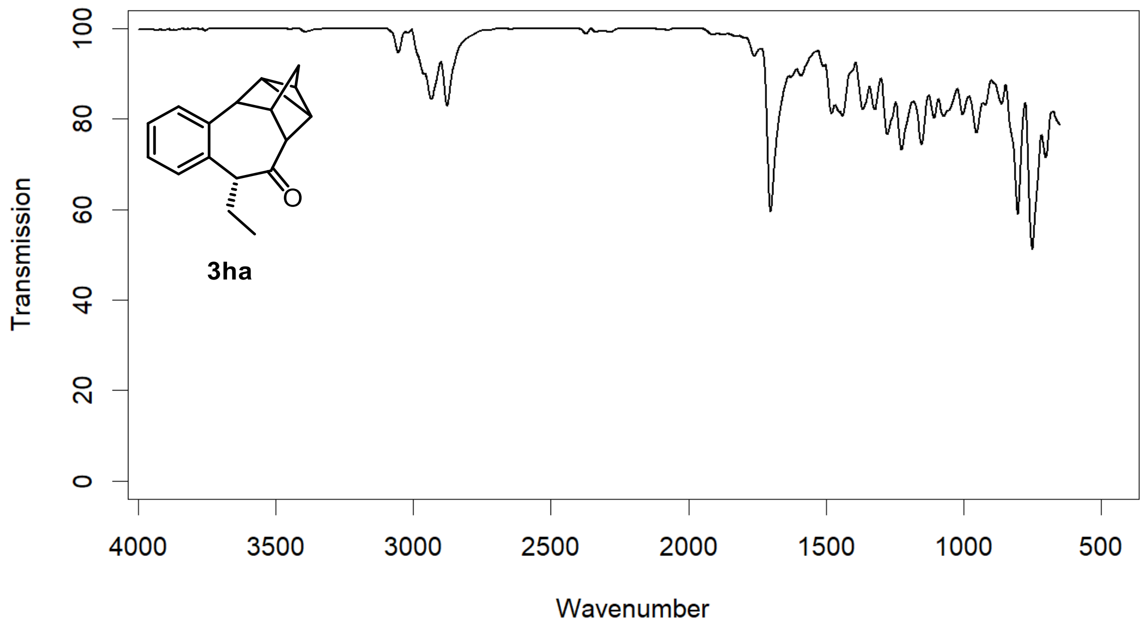

Figure S 278. IR spectrum of **3ha**.

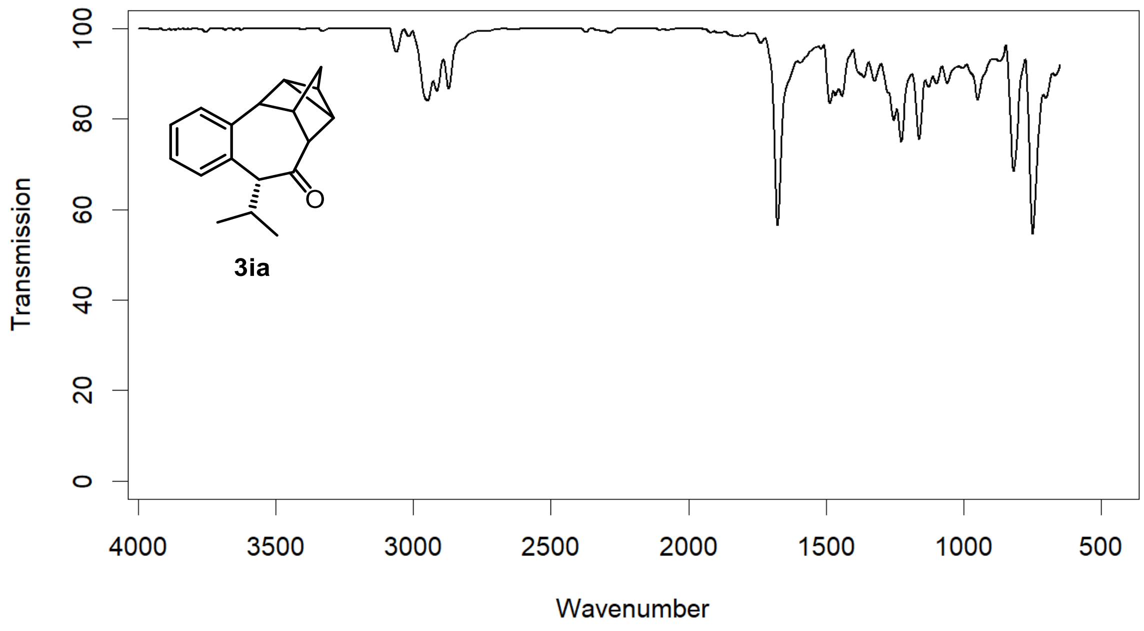

Figure S 279. IR spectrum of **3ia**.

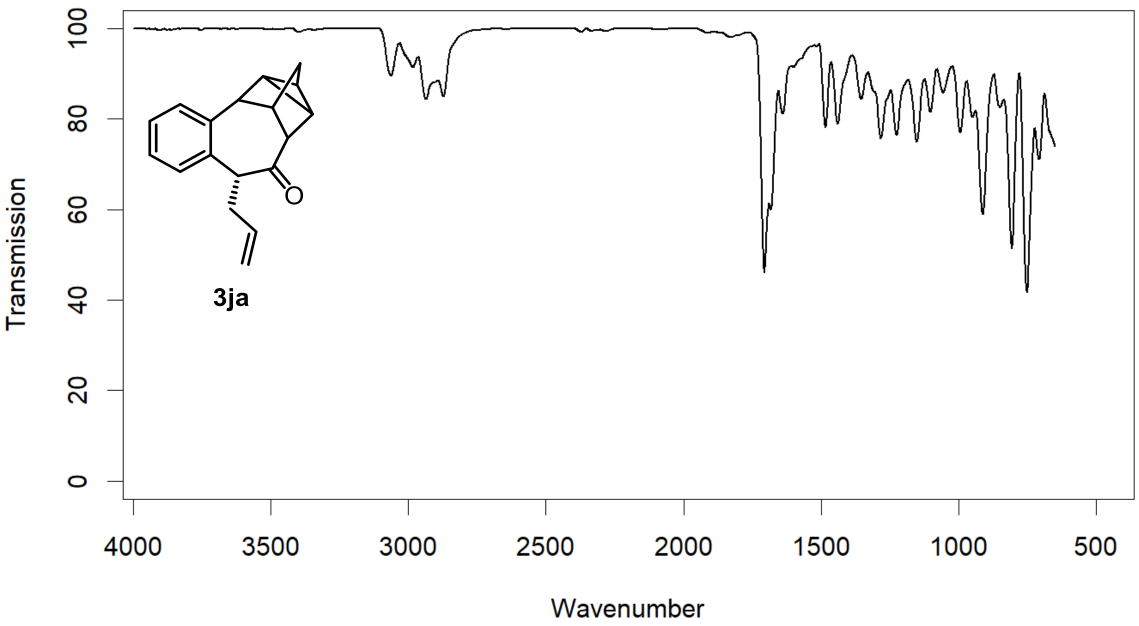

Figure S 280. IR spectrum of **3ja**.

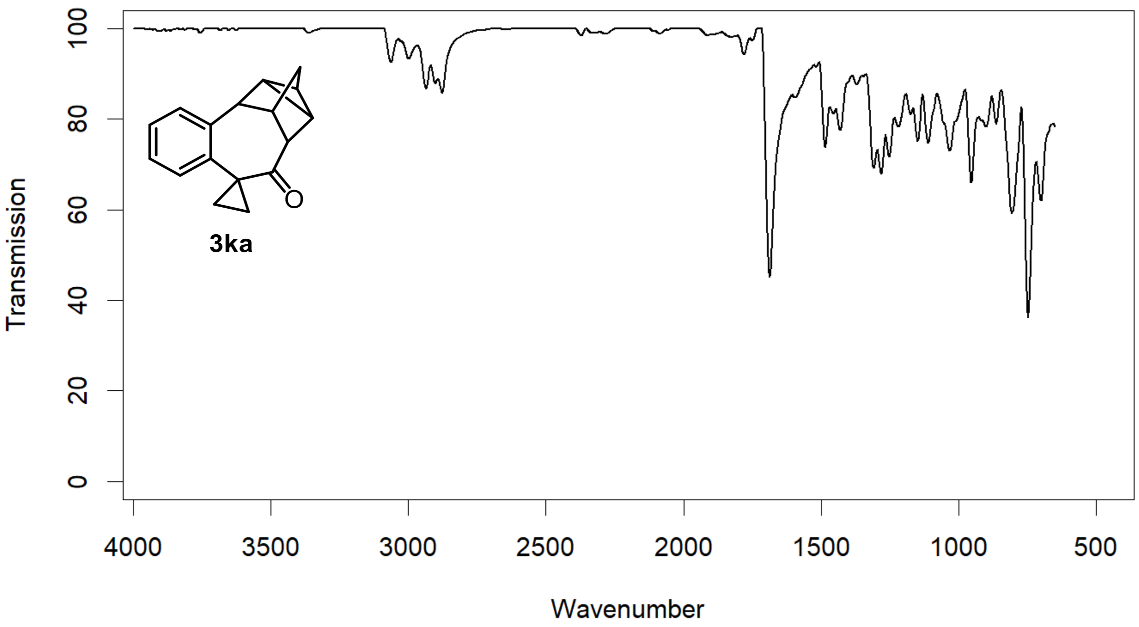

Figure S 281. IR spectrum of **3ka**.

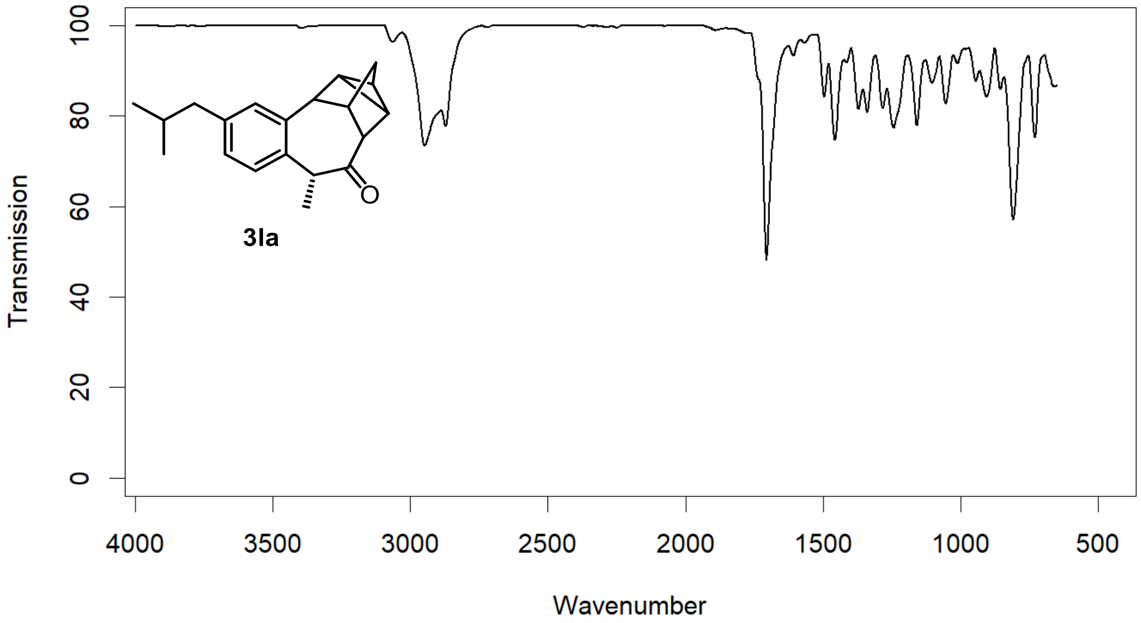

Figure S 282. IR spectrum of **3la**.

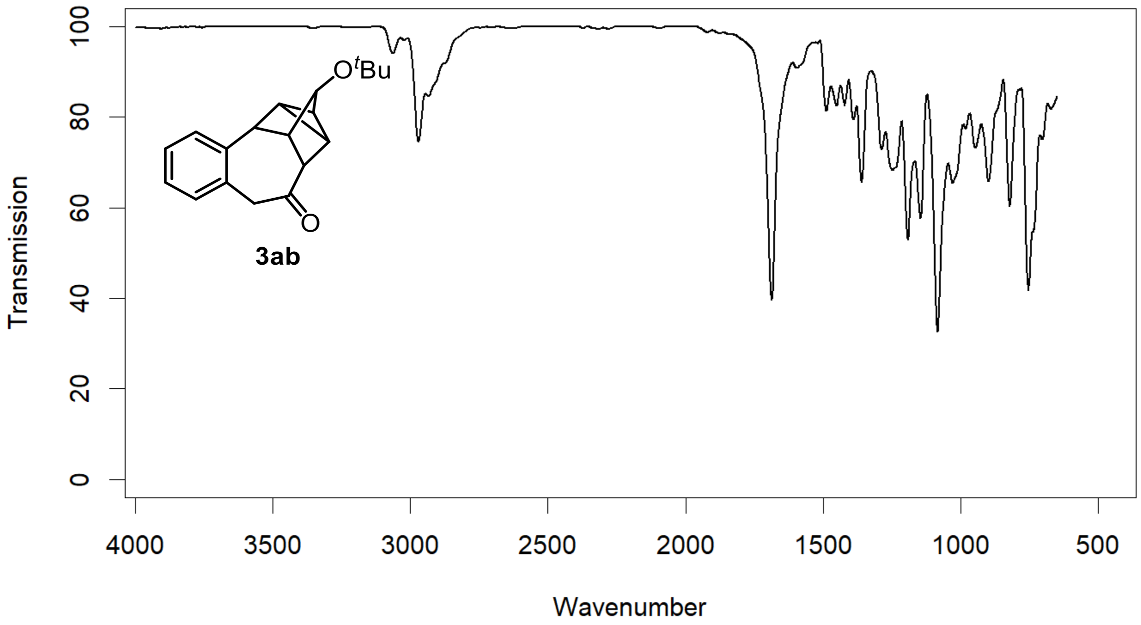

Figure S 283. IR spectrum of **3ab**.

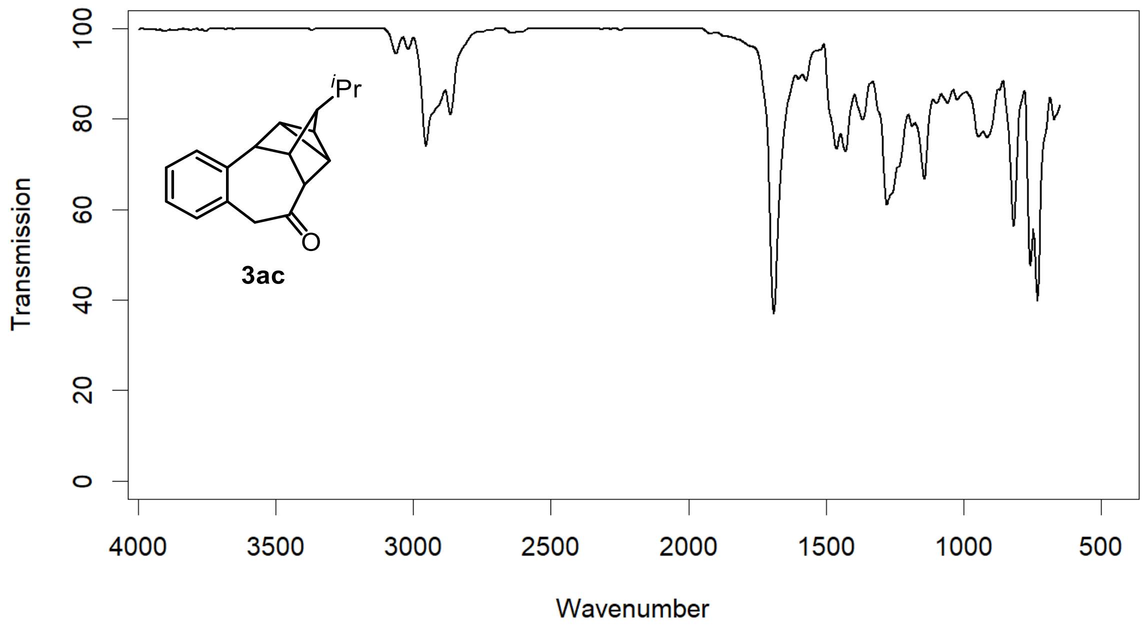

Figure S 284. IR spectrum of **3ac**.

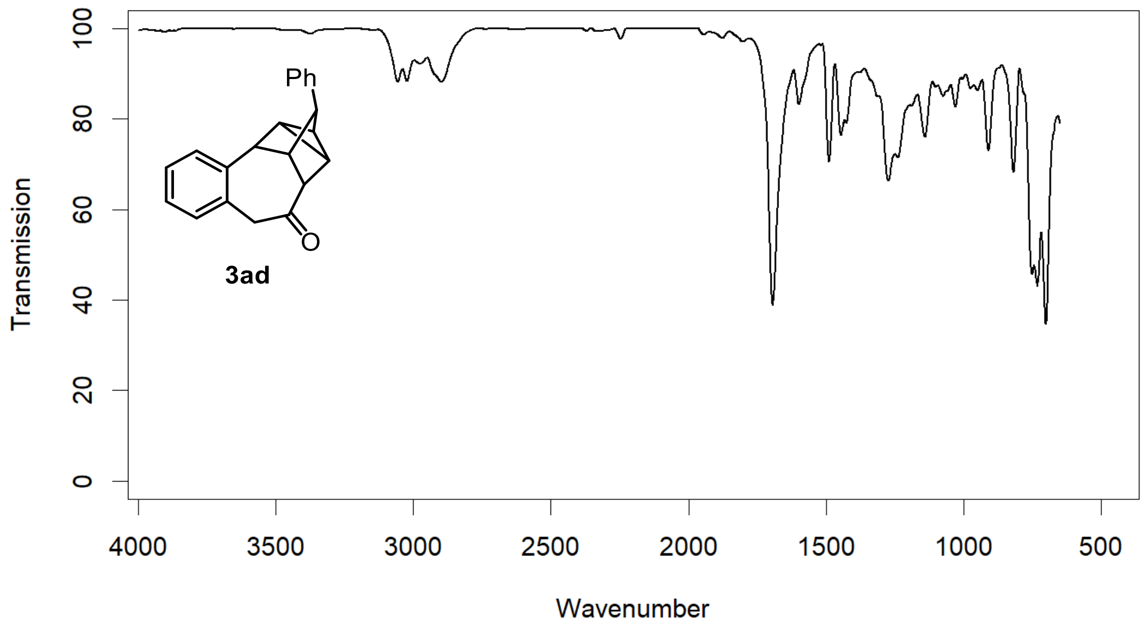

Figure S 285. IR spectrum of **3ad**.

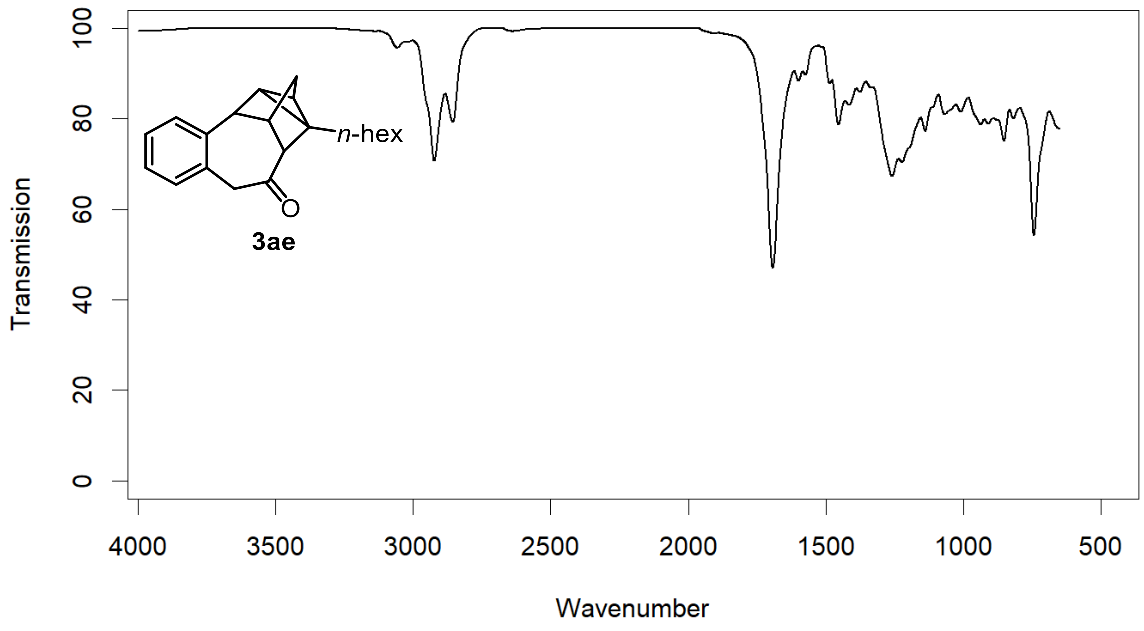

Figure S 286. IR spectrum of **3ae**.

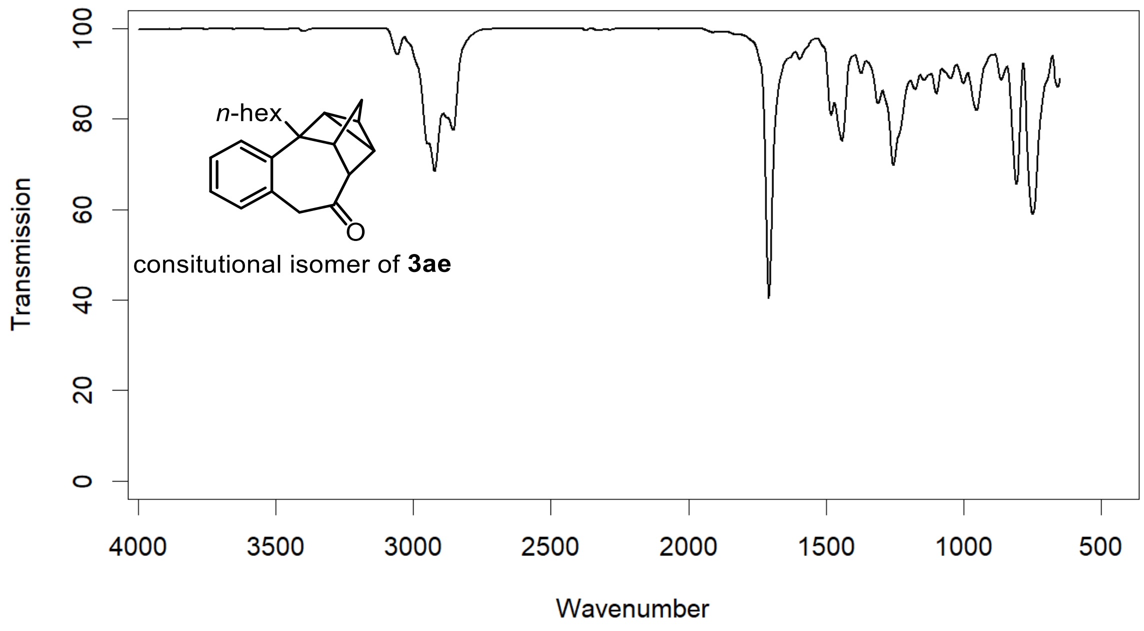

Figure S 287. IR spectrum of the constitutional isomer of **3ae**.

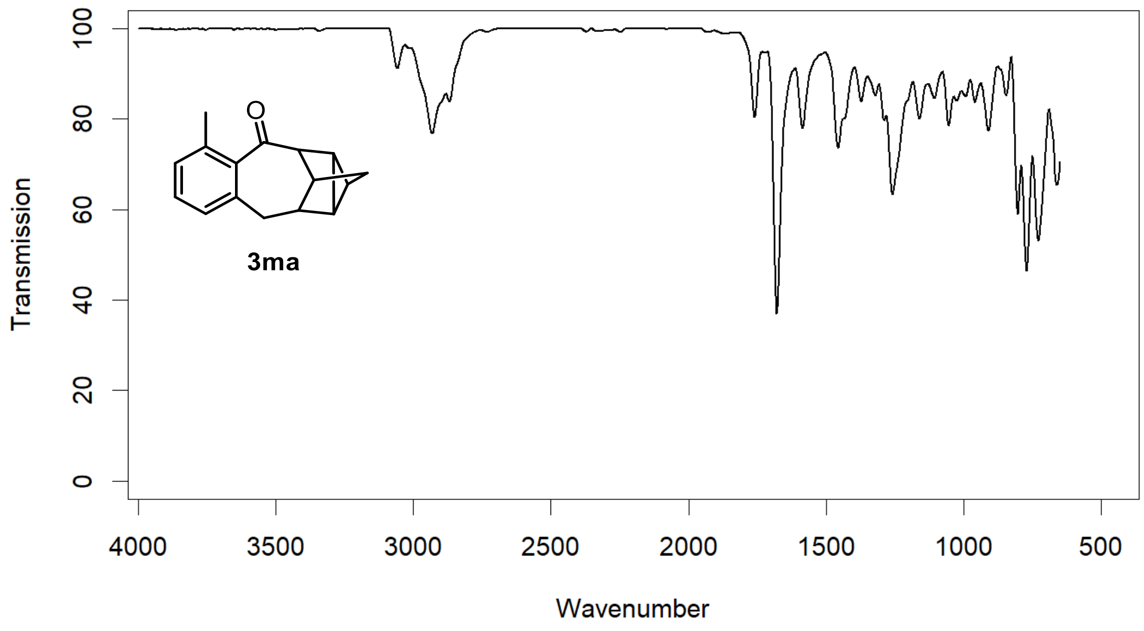

Figure S 288. IR spectrum of **3ma**.

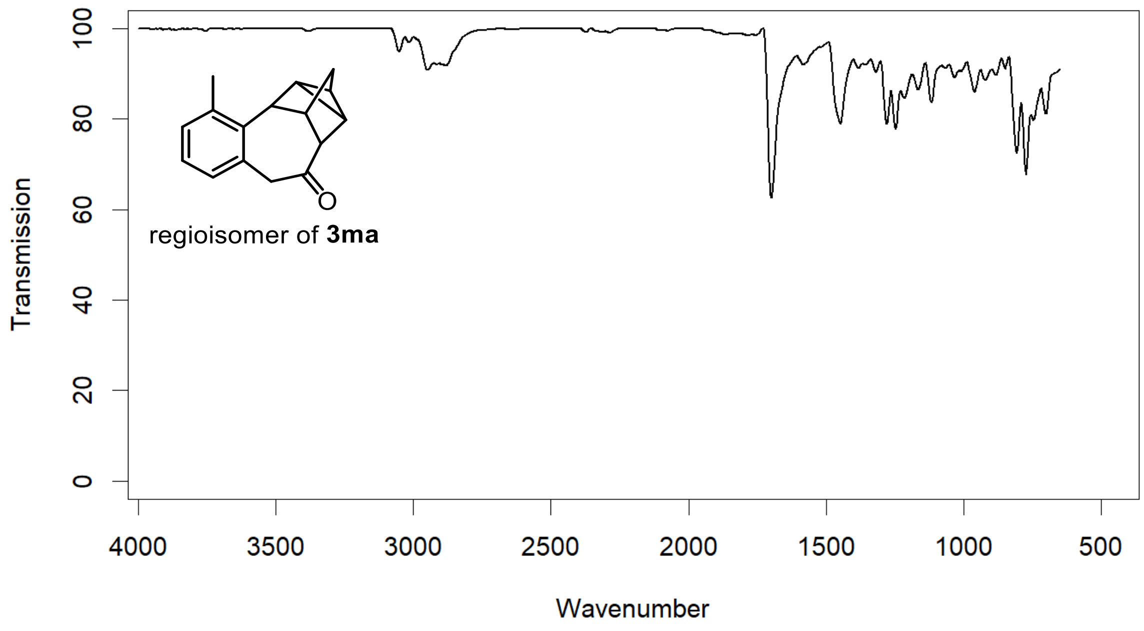

Figure S 289. IR spectrum of the regioisomer of **3ma**.

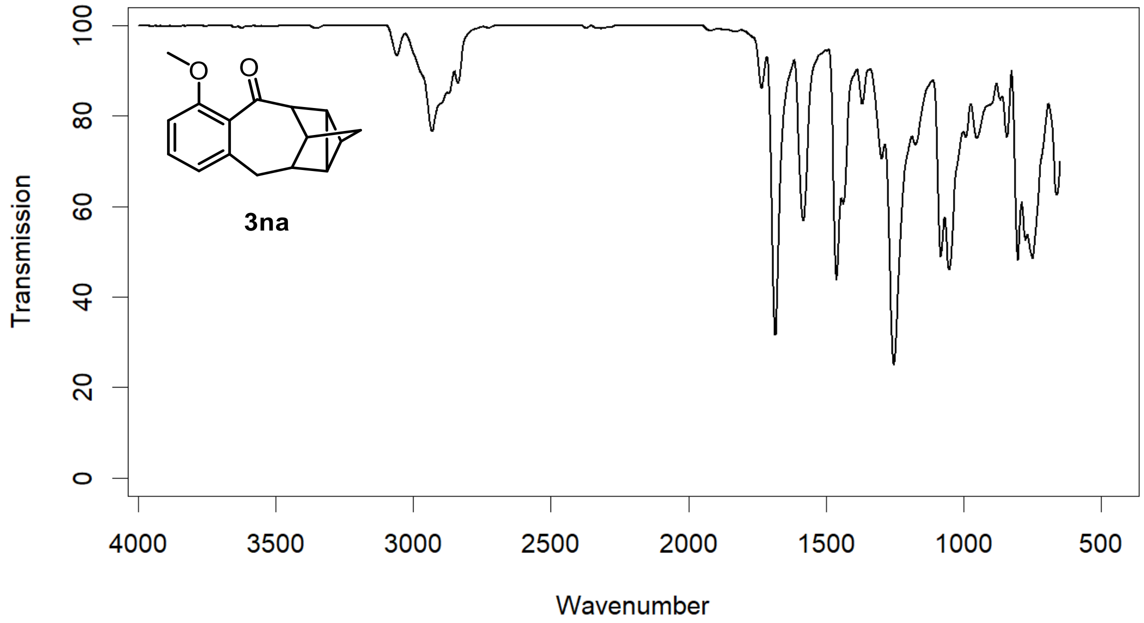

Figure S 290. IR spectrum of **3na**.

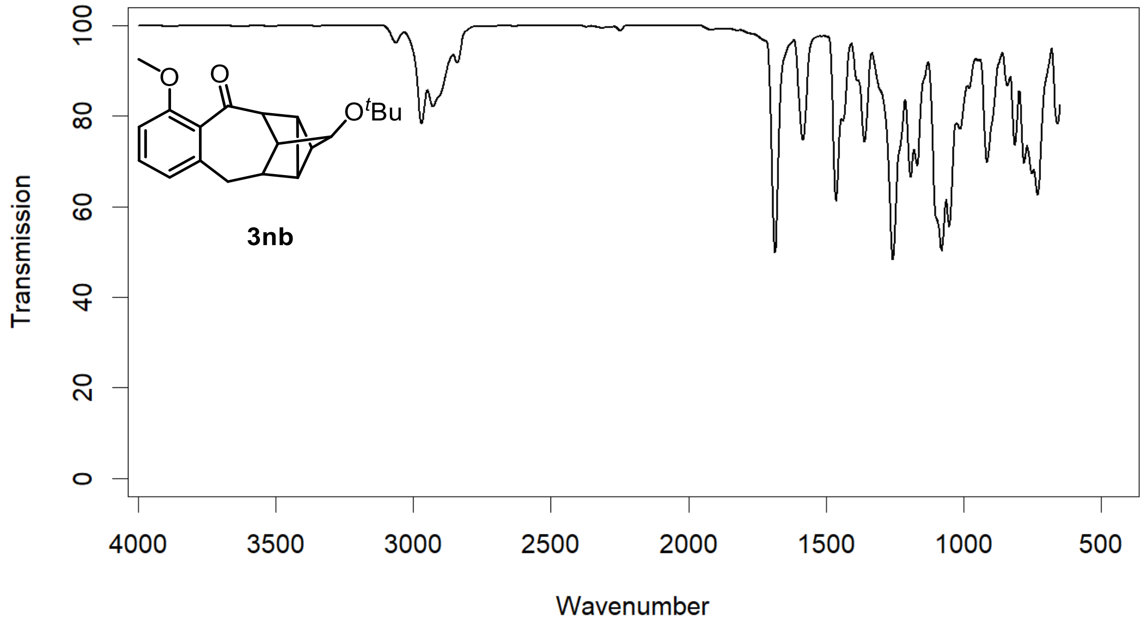

Figure S 291. IR spectrum of **3nb**.

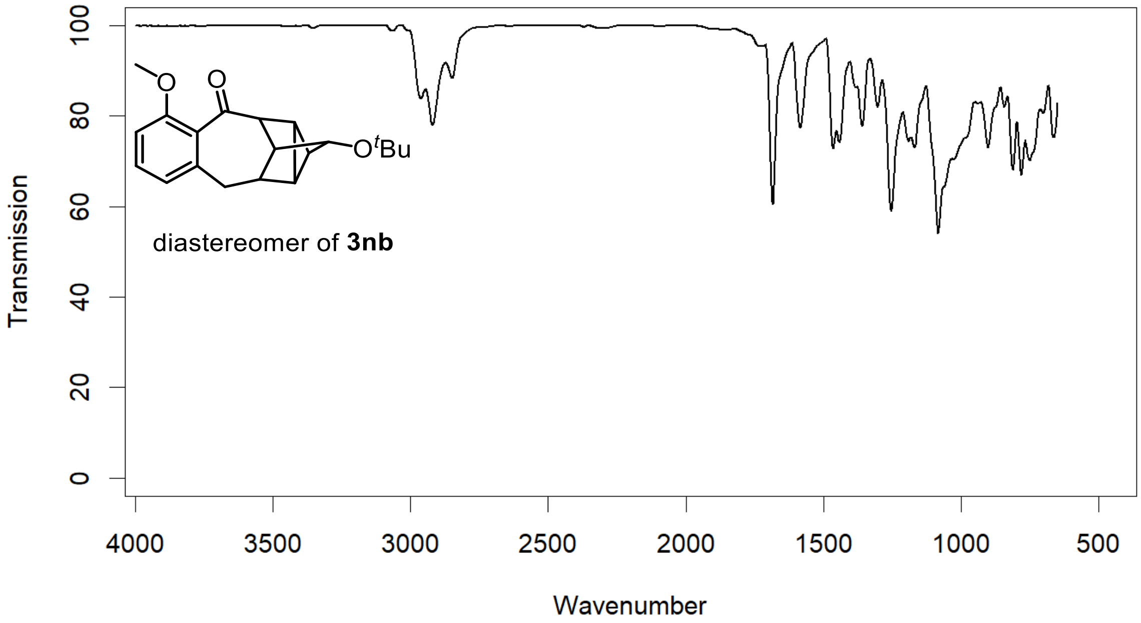

Figure S 292. IR spectrum of the diastereomer of **3nb**.

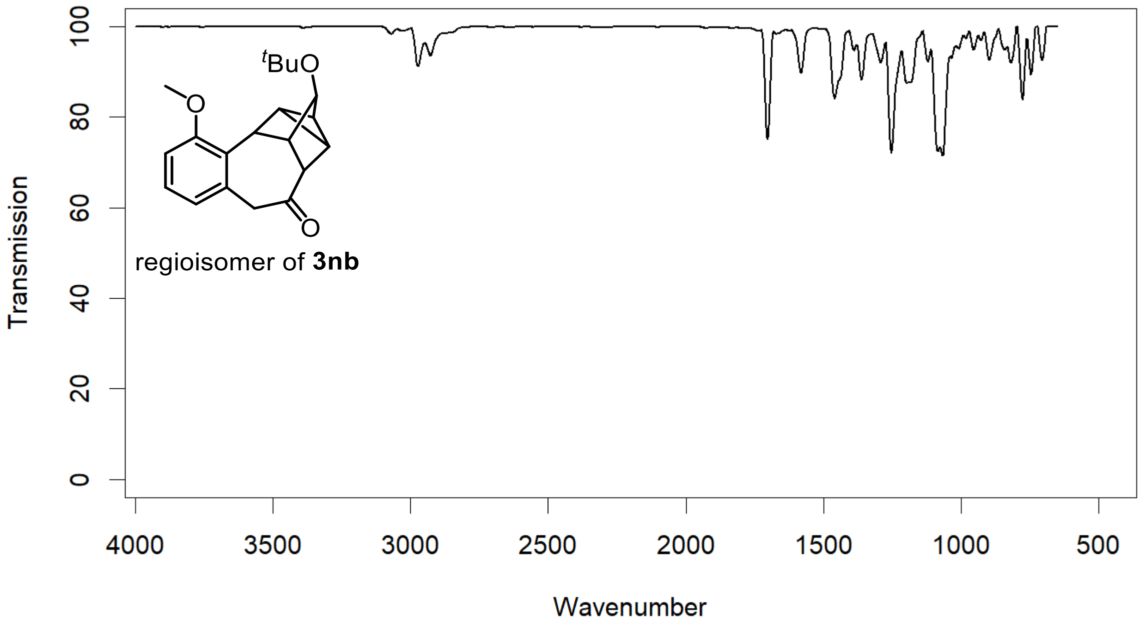

Figure S 293. IR spectrum of the regioisomer of **3nb**.

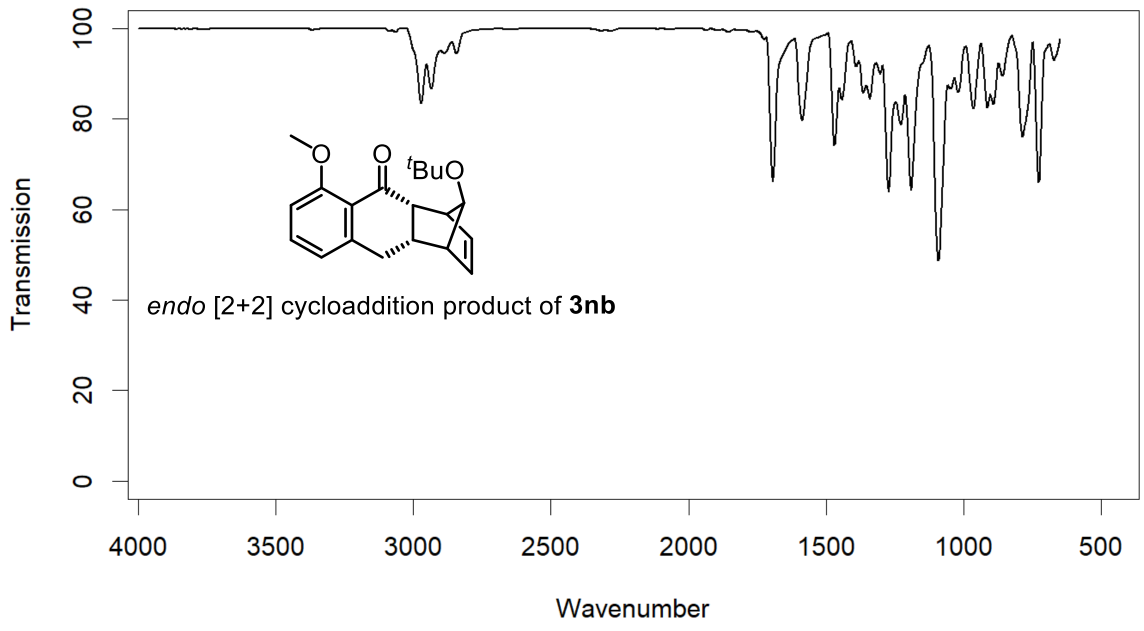

Figure S 294. IR spectrum of the endo [2+2] cycloaddition product of **3nb**.

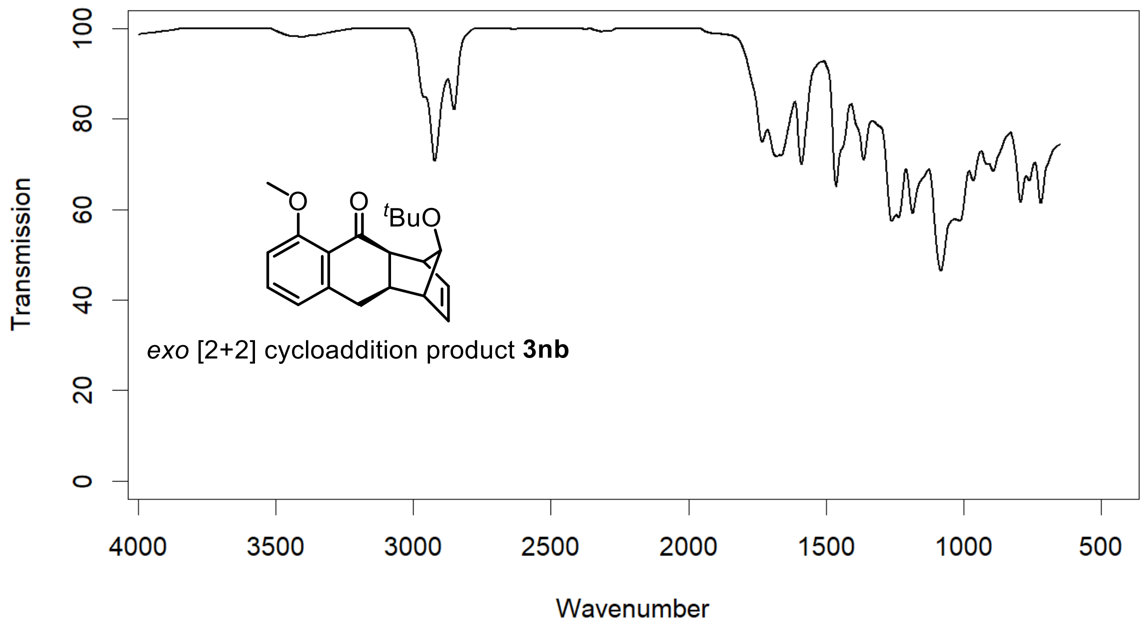

Figure S 295. IR spectrum of exo [2+2] cycloaddition product of **3nb**.

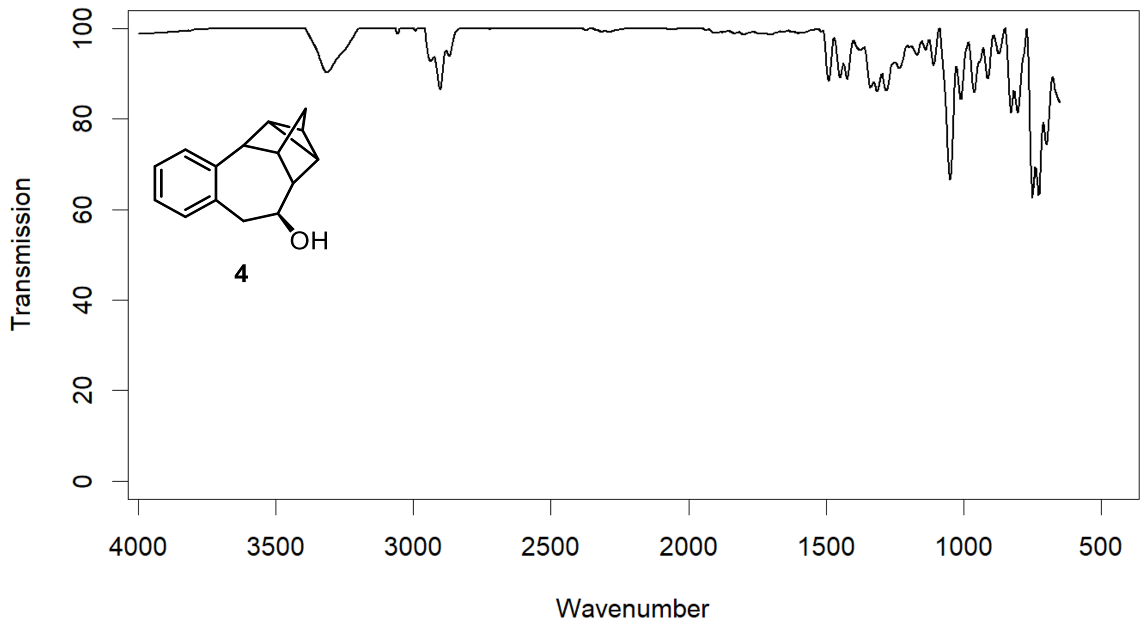

Figure S 296. IR spectrum of **4**.

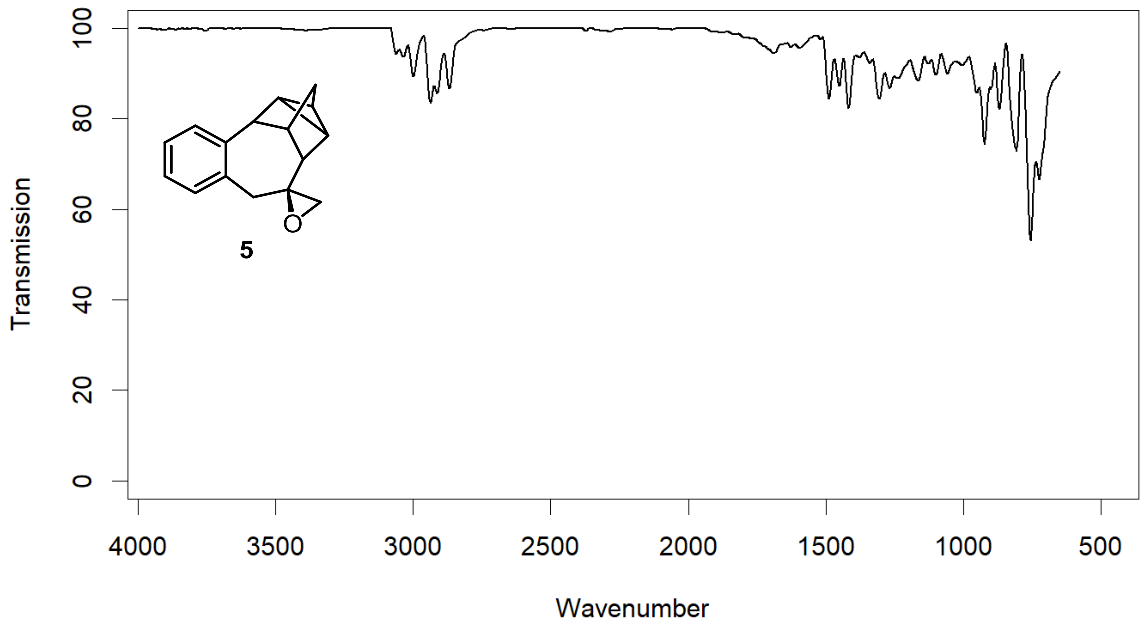

Figure S 297. IR spectrum of **5**.

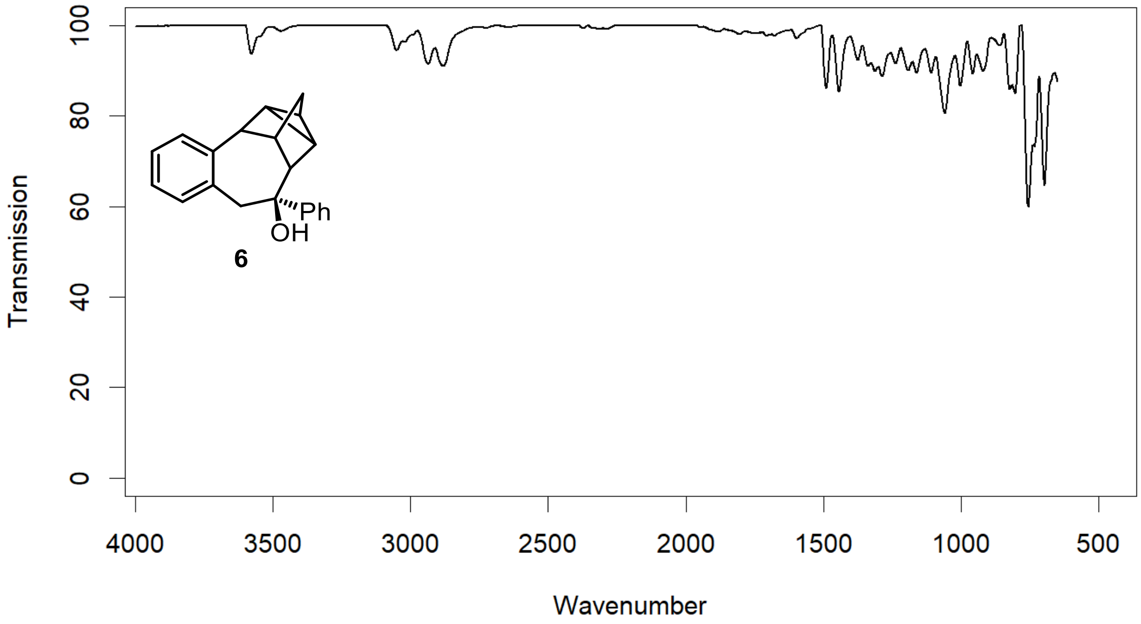

Figure S 298. IR spectrum of **6**.

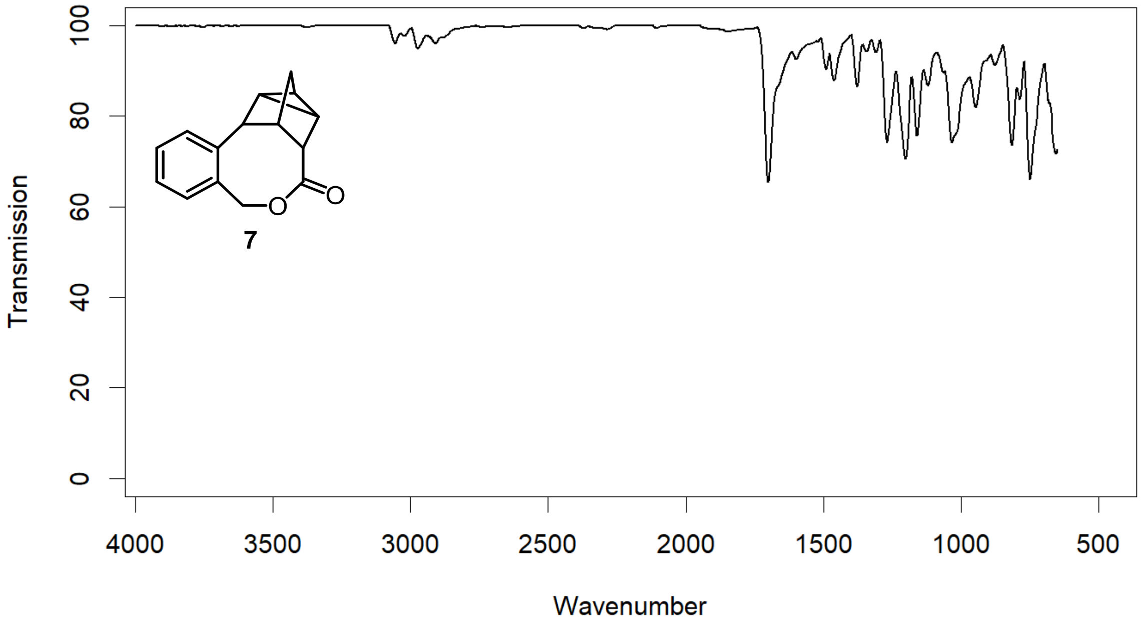

Figure S 299. IR spectrum of **7**.
